# Tumor Intrinsic Subtypes and Gene Expression Signatures in Early-Stage *ERBB2/HER2*-Positive Breast Cancer: A Pooled Analysis of CALGB 40601, NeoALTTO, and NSABP B-41 Trials

**DOI:** 10.1001/jamaoncol.2023.7304

**Published:** 2024-03-28

**Authors:** Aranzazu Fernandez-Martinez, Mattia Rediti, Gong Tang, Tomás Pascual, Katherine A. Hoadley, David Venet, Naim U. Rashid, Patricia A. Spears, Md N. Islam, Sarra El-Abed, Judith Bliss, Matteo Lambertini, Serena Di Cosimo, Jens Huobe, David Goerlitz, Rong Hu, Peter C. Lucas, Sandra M. Swain, Christos Sotiriou, Charles M. Perou, Lisa A. Carey

**Affiliations:** 1 Lineberger Comprehensive Center, University of North Carolina, Chapel Hill; 2 Department of Genetics, University of North Carolina, Chapel Hill; 3 Breast Cancer Translational Research Laboratory, Institut Jules Bordet, Hôpital Universitaire de Bruxelles (H.U.B), Université Libre de Bruxelles (ULB), Brussels, Belgium; 4 NSABP Foundation Inc., Pittsburgh, Pennsylvania; 5 Department of Biostatistics, University of Pittsburgh, Pittsburgh, Pennsylvania; 6 Department of Medical Oncology, Hospital Clínic de Barcelona, Spain; 7 Translational Genomics and Targeted Therapeutics in Solid Tumors, August Pi i Sunyer Biomedical Research Institute (IDIBAPS), Barcelona, Spain; 8 SOLTI Breast Cancer Cooperative Group, Barcelona, Spain; 9 Department of Biostatistics, University of North Carolina, Chapel Hill; 10 Genomics and Epigenomics Shared Resource (GESR), Georgetown University Medical Center, Washington, DC; 11 Breast International Group, Brussels, Belgium; 12 The Institute of Cancer Research, Clinical Trials & Statistics Unit, London, United Kingdom; 13 Department of Internal Medicine and Medical Specialties (DiMI), School of Medicine, University of Genova, Genova, Italy; 14 Department of Medical Oncology, UOC Clinica di Oncologia Medica, IRCCS Ospedale Policlinico San Martino, Genova, Italy; 15 Integrated Biology Platform, Fondazione IRCCS Istituto Nazionale dei Tumori, Milano, Italy; 16 Kantonsspital St. Gallen, Brustzentrum, Departement Interdisziplinäre medizinische Dienste, St. Gallen, Switzerland; 17 Lombardi Comprehensive Cancer Center, Georgetown University Medical Center, Washington, DC; 18 Department of Pathology, School of Medicine, University of Pittsburgh, Pittsburgh, Pennsylvania; 19 Division of Hematology-Oncology, Department of Medicine, School of Medicine, University of North Carolina at Chapel Hill, Chapel Hill, North Carolina

## Abstract

**Importance:**

Biologic features may affect pathologic complete response (pCR) and event-free survival (EFS) after neoadjuvant chemotherapy plus *ERBB2/HER2* blockade in *ERBB2/HER2*-positive early breast cancer (EBC).

**Objective:**

To define the quantitative association between pCR and EFS by intrinsic subtype and by other gene expression signatures in a pooled analysis of 3 phase 3 trials: CALGB 40601, NeoALTTO, and NSABP B-41.

**Design, Setting, and Participants:**

In this retrospective pooled analysis, 1289 patients with EBC received chemotherapy plus either trastuzumab, lapatinib, or the combination, with a combined median follow-up of 5.5 years. Gene expression profiling by RNA sequencing was obtained from 758 samples, and intrinsic subtypes and 618 gene expression signatures were calculated. Data analyses were performed from June 1, 2020, to January 1, 2023.

**Main Outcomes and Measures:**

The association of clinical variables and gene expression biomarkers with pCR and EFS were studied by logistic regression and Cox analyses.

**Results:**

In the pooled analysis, of 758 women, median age was 49 years, 12% were Asian, 6% Black, and 75% were White. Overall, pCR results were associated with EFS in the ERBB2-enriched (hazard ratio [HR], 0.45; 95% CI, 0.29-0.70; *P* < .001) and basal-like (HR, 0.19; 95% CI, 0.04-0.86; *P* = *.*03) subtypes but not in luminal A or B tumors. Dual trastuzumab plus lapatinib blockade over trastuzumab alone had a trend toward EFS benefit in the intention-to-treat population; however, in the ERBB2-enriched subtype there was a significant and independent EFS benefit of trastuzumab plus lapatinib vs trastuzumab alone (HR, 0.47; 95% CI, 0.27-0.83; *P* = .009). Overall, 275 of 618 gene expression signatures (44.5%) were significantly associated with pCR and 9 of 618 (1.5%) with EFS. The *ERBB2/HER2* amplicon and multiple immune signatures were significantly associated with pCR. Luminal-related signatures were associated with lower pCR rates but better EFS, especially among patients with residual disease and independent of hormone receptor status. There was significant adjusted HR for pCR ranging from 0.45 to 0.81 (higher pCR) and 1.21-1.94 (lower pCR rate); significant adjusted HR for EFS ranged from 0.71 to 0.94.

**Conclusions and relevance:**

In patients with *ERBB2/HER2*-positive EBC, the association between pCR and EFS differed by tumor intrinsic subtype, and the benefit of dual *ERBB2/HER2* blockade was limited to ERBB2-enriched tumors. Immune-activated signatures were concordantly associated with higher pCR rates and better EFS, whereas luminal signatures were associated with lower pCR rates.

## Key Points

**Question:**

What is the quantitative association between pathologic complete response (pCR) and event-free survival (EFS) by intrinsic subtype and other gene expression signatures in patients with *ERBB2*/*HER2*-positive early breast cancer (EBC) treated in the neoadjuvant setting?

**Findings:**

In this retrospective pooled analysis of 3 randomized clinical trials including 1289 patients with *ERBB2/HER2*-positive EBC, the association between pCR and EFS differed by tumor intrinsic subtype, and the benefit of dual *ERBB2/HER2*-blockade was limited to ERBB2-enriched tumors. Immune-activated signatures were associated with higher pCR rates and better EFS, whereas luminal signatures were associated with lower pCR rates.

**Meaning:**

Intrinsic subtype and immune gene expression biomarkers may help guide personalized treatment in patients with *ERBB2/HER2*-postive EBC.

## Teaser

This retrospective analysis of data from 3 randomized clinical trials examines the association of pathologic complete response with event-free survival by intrinsic subtype and other gene expression signatures in patients with *ERBB2*/*HER2*-positive early breast cancer treated in the neoadjuvant setting

## Introduction

The highly aggressive *ERBB2*/*HER2*-positive breast cancer accounts for 20% of all breast tumors. However, the success of multiple *ERBB2/HER2*-targeting drugs has markedly improved outcomes. In *ERBB2/HER2*-positive early breast cancer (EBC), neoadjuvant treatment is now the standard of care given the surgical benefits of tumor downstaging^1^ and the benefits of tailoring adjuvant anti-*ERBB2/HER2* drugs based on the presence of residual disease at surgery.^2^ Pathologic complete response (pCR) at surgery has been associated with improved survival,^3^ but a predictable quantitative relationship between pCR benefit and survival outcomes has been elusive.

In randomized neoadjuvant trials, dual *ERBB2/HER2*-targeting has been associated with higher pCR rates than single *ERBB2/HER2* targeting.^4,5,6,7^ However, the magnitude of this effect has differed across trial populations and drugs, and neoadjuvant trials are typically underpowered for long-term outcomes. This was true of 3 phase 3 trials investigating dual (trastuzumab and lapatinib) vs single (trastuzumab or lapatinib) anti-*ERBB2/HER2* drugs added to chemotherapy: NeoALTTO (NCT00553358), CALGB 40601 (NCT00770809), and NSABP B-41 (NCT00486668). All 3 studies demonstrated higher pCR rates and better survival with dual therapy, which was statistically significant in NeoALTTO for pCR,^4^ and CALGB 40601 for relapse-free survival.^8^ In a trial-level meta-analysis including these 3 phase 3 studies and a similar phase 2 trial, Cher-LOB, dual blockade with trastuzumab plus lapatinib in combination with neoadjuvant chemotherapy significantly prolonged relapse-free and overall survival.^9^ This result contrasts with the findings of the phase 3 adjuvant trial ALTTO,^10^ in which patients with *ERBB2/HER2*-positive EBC treated with trastuzumab plus lapatinib blockade as adjuvant therapy had a 16% reduction in disease-free survival (DFS) with a hazard ratio (HR) that did not meet statistical significance. Dual therapy with trastuzumab plus pertuzumab in the phase 3 APHINITY trial resulted in a significant 24% improvement in invasive DFS HR and is the current standard of care in patients with high-risk disease.^11^

Differences in the effect of dual *ERBB2/HER2* blockade and robustness of the association of pCR with EFS could be partly explained by differences in tumor and microenvironment biology. *ERBB2/HER2*-positive EBC is not a singular biological entity; instead, it is characterized by heterogeneity of both cancer and immune cell components. At a tumor level, all the intrinsic molecular subtypes (ie, luminal A, luminal B, *ERBB2/HER2*-enriched, and basal-like) can be found within *ERBB2/HER2*-positive breast cancer tumors.^12^ This intrinsic tumor heterogeneity has clinical implications. *ERBB2/HER2*-positive/*ERBB2*-enriched tumors have been systematically associated with higher pCR rates for *ERBB2/HER2*-targeted therapies,^13^ and *ERBB2/HER2*-positive/ERBB2-enriched and *ERBB2/HER2*-positive/basal-like tumors have been associated with worse prognoses compared with *ERBB2/HER2*-positive/luminal tumors, particularly in those with residual disease.^8,14^

Similarly, the immune microenvironment is both predictive and prognostic. Tumor-infiltrating lymphocytes (TILs), which correlate strongly with T-cell expression signatures, have been significantly associated with higher pCR rates and EFS in multiple *ERBB2/HER2*-positive neoadjuvant studies.^7,8,15,16,17,18,19,20,21,22,23,24,25^ In a recent analysis, immune gene signatures appear more valuable than TILs in pCR prediction.^26^ Another important finding is that immune signatures and TILs are associated with both higher pCR rates and longer EFS, unlike intrinsic subtypes, which often work in opposite directions for predicting response and survival.^8^

We hypothesized that the biological tumor and immune heterogeneity of *ERBB2/HER2*-positive EBC contribute to the inconsistent results coming from different neoadjuvant and adjuvant clinical trials. In this analysis, we examined how intrinsic subtype, immune activation status, and other gene expression signatures contribute to pCR and EFS, and the benefit of dual therapy with trastuzumab and lapatinib compared with single-agent trastuzumab, by performing individual patient-level biomarker analysis of 3 phase 3 clinical trials with similar designs: NeoALTTO,^4,15,21,27,28,29,30^ CALGB 40601,^7,8,26^ and NSABP B-41.^6,14,31,32^

## Methods

### Neoadjuvant Trials: Study Designs and Patients

All 3 phase 3 trials have had pCR, correlative, and/or survival end points published previously (NeoALTTO,^4,15,21,27,28,29,30^ CALGB 40601,^7,8,26^ NSABP B-41^6,14,31,32^). All trials involved previously untreated patients with early *ERBB2/HER2*-positive breast cancer randomized to chemotherapy with single trastuzumab, single lapatinib, or dual trastuzumab and lapatinib anti-*ERBB2/HER2* therapy (eFigure 1A in Supplement 1). Trial differences included a 6-week lead-in phase of the randomized anti-*ERBB2/HER2* agent(s) in the NeoALTTO trial,^4,15,21,27,28,29,30^ all chemotherapy given as neoadjuvant therapy in B-41, neoadjuvant durations varying from 16 weeks (CALGP 40601) to 28 weeks (B-41), and that the adjuvant anti-*ERBB2/HER2* therapy was as randomized in the NeoALTTO trial but was single-agent trastuzumab in the other 2 trials.

To homogenize the clinical outcomes from the 3 clinical trials, for this patient-level pooled analysis, pCR was defined as the absence of invasive tumor cells in the breast (ypT0/is). We used EFS for long-term outcome, defined as the time from randomization to the event (ie, local recurrence, regional recurrence, distant recurrence, nonbreast second primary tumors, contralateral invasive breast cancer, and death of any cause). A slight difference in the event definitions between the 3 clinical trials included progressions during the neoadjuvant phase, which were regarded as events in the CALGB 40601 and NeoALTTO trials but not in the B-41 trial. However, only 3 patients from the NeoALTTO and 5 from the B-41 trials progressed during the neoadjuvant phase. In the intention-to-treat (ITT) cohort, 10 patients from NSABP B-41 did not have EFS events and/or time information collected.

Ethics committee and relevant health authorities at each participating site approved the NeoALTTO, CALGB 40601, and NSABP B-41 studies, and all patients provided written informed consent, including future biomarker research.

### Tumor Gene Expression Analyses

Gene expression profiles from pretreatment core biopsies were obtained from 249 of 455 participants (54.7%) in the NeoALTTO, 264 of 305 participants (86.6%) in the CALGB 40601, and 245 of 529 participants (46.3%) in the NSABP B-41 trials, respectively (CONSORT diagram, eFigure 1B in Supplement 1). The tumor preservation methods, RNA extraction, RNAseq library preparation, sequencing parameters, bioinformatic algorithms, and data preprocessing are summarized in the eMethods in Supplement 1. A principal component analysis (PCA) plot before and after the batch effect correction can be found in eFigure 2 in Supplement 1.

For the 3 studies, intrinsic subtypes and a collection of 618 gene expression signatures (GES) representing diverse cell types and biologic pathways were obtained from RNAseq gene expression data as described previously^8,26^ (eMethods, eTable 1 in Supplement 1).

### Statistical Analysis

Comparisons of differences in baseline clinicopathologic variables among the trials were made using a Wilcoxon rank-sum for continuous variables and χ^2^ or Fisher exact tests for categorical variables. Proportions and *P* values are provided. For the survival analyses, the 5-year EFS proportions for each group were estimated using the Kaplan-Meier method. The association between clinical and genomic biomarkers with pCR and EFS was assessed using univariable and multivariable logistic and Cox regression models, respectively. Clinical variables considered for multivariable models included clinical trial (ie, CALGB 40601, NeoALTTO, and NSABP B-41, where CALGB 40601 was the reference group), treatment arm (ie, trastuzumab, trastuzumab plus lapatinib, or lapatinib, where trastuzumab was the reference arm), hormone-receptor status (hormone receptor positive vs hormone receptor negative), clinical tumor size (T1-T2 vs T3-T4a-c), and clinical node involvement (node positive vs node negative). Inflammatory breast cancer was excluded in all trials. All Cox models were stratified by clinical trial. Odds ratios (ORs), hazard ratios (HRs), and 95% CIs were calculated for each variable. The significance level was set to a 2-sided α of .05, and *P* values were adjusted for multiple testing using the Benjamini and Hochberg method to control the false discovery rate. To avoid a potential guarantee time bias in the multivariable EFS models including pCR status, we performed a 30-week landmark analysis. The landmark subpopulation included only patients without events and being followed up at 30 weeks after randomization.^27,33^

All the analyses were based on the study clinical database frozen on May 26, 2016, in the NeoALTTO trial, on June 10, 2021, in the CALGB 40601 trial, and on December 31, 2016, in the NSABP B-41 trial, and were performed using R (version 3.5.2; R Foundation for Statistical Computing) and Python statistical software (version 3.6; Python Software Foundation). Data analyses were performed from June 1, 2020, to January 1, 2023.

## Results

### Clinicopathologic Characteristics and Efficacy Analysis in the ITT Cohort

There were 1289 patients with *ERBB2/HER2*-positive EBC included. Although generally similar, several baseline clinicopathologic features differed among the 3 trials, including larger tumors, more node-positive, and a higher proportion of Asian participants in the NeoALTTO trial (eTable 2 in Supplement 1).

In the ITT population, a multivariable analysis for pCR prediction found that patients treated with trastuzumab plus lapatinib had significantly higher pCR rates than those treated with trastuzumab (adjusted OR [aOR], 1.80; 95% CI, 1.36-2.39; *P* < .001), with no significant differences between the lapatinib and trastuzumab arms (eTable 3 in Supplement 1). The Kaplan-Meier estimates of 5-year EFS by treatment were 83%, 79%, and 73% for the lapatinib and trastuzumab, trastuzumab, and lapatinib arms, respectively (eFigure 3 in Supplement 1), a difference that was not significant in a multivariable Cox analysis (lapatinib and trastuzumab vs trastuzumab: adjusted HR [aHR], 0.74; 95% CI, 0.54-1.01; *P* = .056) (eTable 4 in Supplement 1).

When comparing the EFS among the 3 studies, the NeoALTTO trial had significantly worse EFS outcomes than the CALGB 40601 trial (HR, 1.92; 95% CI, 1.39-2.66; *P* < .001) (eFigure 4 in Supplement 1). In the overall cohort of patients that were treated with trastuzumab in the neoadjuvant setting (n = 887), patients with pCR had a significantly better EFS outcome than patients with residual disease in a multivariable model stratified by clinical trial and adjusted by treatment arm, hormone receptor status, tumor size, and node status (aHR for pCR vs residual disease: 0.47; 95% CI, 0.34-0.66; *P* < .001). The Kaplan-Meier estimates of 5-year EFS were 88% for pCR and 74% for patients with residual disease (eFigure 5A in Supplement 1). Similar results were observed when the landmark analysis was performed (n = 856) (eFigure 5B in Supplement 1).

Similar distributions of local, distant, and organ sites of recurrence or death comprising the EFS events were seen across the 3 trials (eFigure 6 in Supplement 1). Patients treated only with lapatinib generally experienced more distant recurrences than patients treated with trastuzumab or trastuzumab plus lapatinib, except for brain metastasis, which was more frequent in the trastuzumab arm, suggesting activity of lapatinib in preventing brain relapses as has been noted with other anti-*ERBB2/HER2* small molecules (eFigure 7 in Supplement 1).^34^

### Clinical Implications of the Intrinsic Subtypes in the RNAseq Cohort

The CALGB 40601 trial had a higher proportion of patients represented in the RNAseq cohort, whereas the NSABP B-41 trial was more represented in the ITT cohort. Otherwise, there were no significant differences among the clinicopathologic characteristics, response, and EFS survival outcomes of the parent ITT and the RNAseq cohorts (eTable 5 in Supplement 1). In the RNAseq cohort, several baseline clinicopathologic features differed among the 3 trials, including larger tumors, more node positive, and a higher proportion of Asian participants in the NeoALTTO trial (Table).

**Table.** Comparison of Baseline Clinicopathologic Characteristics of the Patients From the NSABP B-41, CALGB 40601, and NeoALTTO Trials in the 758 Patients in the RNAseq Cohort

Variable	No. (%)			*P* value^a^
	NSABP B-41 (n = 245)	C40601 (n = 264)	NeoALTTO (n = 249)	
Age, median (IQR), y	48 (42-56)	49 (41-56)	50 (40-55)	.85
Menopause status				
Postmenopausal	110 (44.9)	102 (38.6)	117 (47.0)	.14
Premenopausal	135 (55.1)	162 (61.4)	132 (53.0)	
Race				
Asian	10 (4.1)	16 (6.1)	68 (27.3)	**<**.001
Black	24 (9.8)	21 (8.0)	4 (1.6)	
Other	7 (2.9)	14 (5.3)	22 (8.8)	
White	204 (83.3)	213 (80.7)	155 (62.2)	
HR status				
HR negative	104 (42.4)	110 (41.7)	115 (46.2)	.55
HR positive	141 (57.6)	154 (58.3)	134 (53.8)	
Clinical tumor size				
T1-T2	168 (68.6)	181 (68.6)	148 (59.4)	<.001
T3-T4	77 (31.4)	61 (23.1)	101 (40.6)	
Unknown	0	22 (8.3)	0	
Tumor size by physical examination, median (IQR), cm	4 (3-6)	4 (3-5)	4 (3-8)	.07
Clinical status of lymph nodes				
N positive	129 (52.7)	136 (51.5)	181 (72.7)	<.001
N negative	116 (47.3)	113 (42.8)	67 (26.9)	
Unknown	0	15 (5.7)	1 (0.4)	
Treatment arm				
Trastuzumab	86 (35.1)	104 (39.4)	77 (30.9)	.004
Trastuzumab and lapatinib	75 (30.6)	103 (39.0)	84 (33.7)	
Lapatinib	84 (34.3)	57 (21.6)	88 (35.3)	

Abbreviations: HR, hormone receptor; T1-T2, tumor size ≤50 mm; T3-T4, tumor size >50 mm; N, lymph nodes infiltration.

^a^ Kruskal-Wallis rank sum test; Pearson χ^2^ test.

When analyzed for RNAseq-based tumor intrinsic subtype on the combined cohort, most tumors were *ERBB2* enriched (57.9%), followed by luminal B (15.0%), luminal A (9.9%), basal-like (8.8%), and normal-like (8.3%). There were no significant differences in the intrinsic subtype distribution by study. Subtype distribution significantly differed by hormone-receptor status (eTable 6 in Supplement 1). There were no significant differences in the hormone-receptor status distribution by locoregional and distant relapse location (eFigure 8A in Supplement 1). However, there were significant differences in the intrinsic subtype distribution by the site of relapse: only patients with *ERBB2/HER2*-positive/ERBB2-enriched and *ERBB2/HER2*-positive/basal-like tumors had brain metastases. Importantly, 5 of the 55 brain metastases were from hormone-receptor–positive tumors; all had a nonluminal subtype (1 basal-like and 4 *ERBB2* enriched). *ERBB2/HER2*-positive/luminal tumors developed more bone and visceral metastasis (eFigure 8B in Supplement 1).

**Figure 1.**
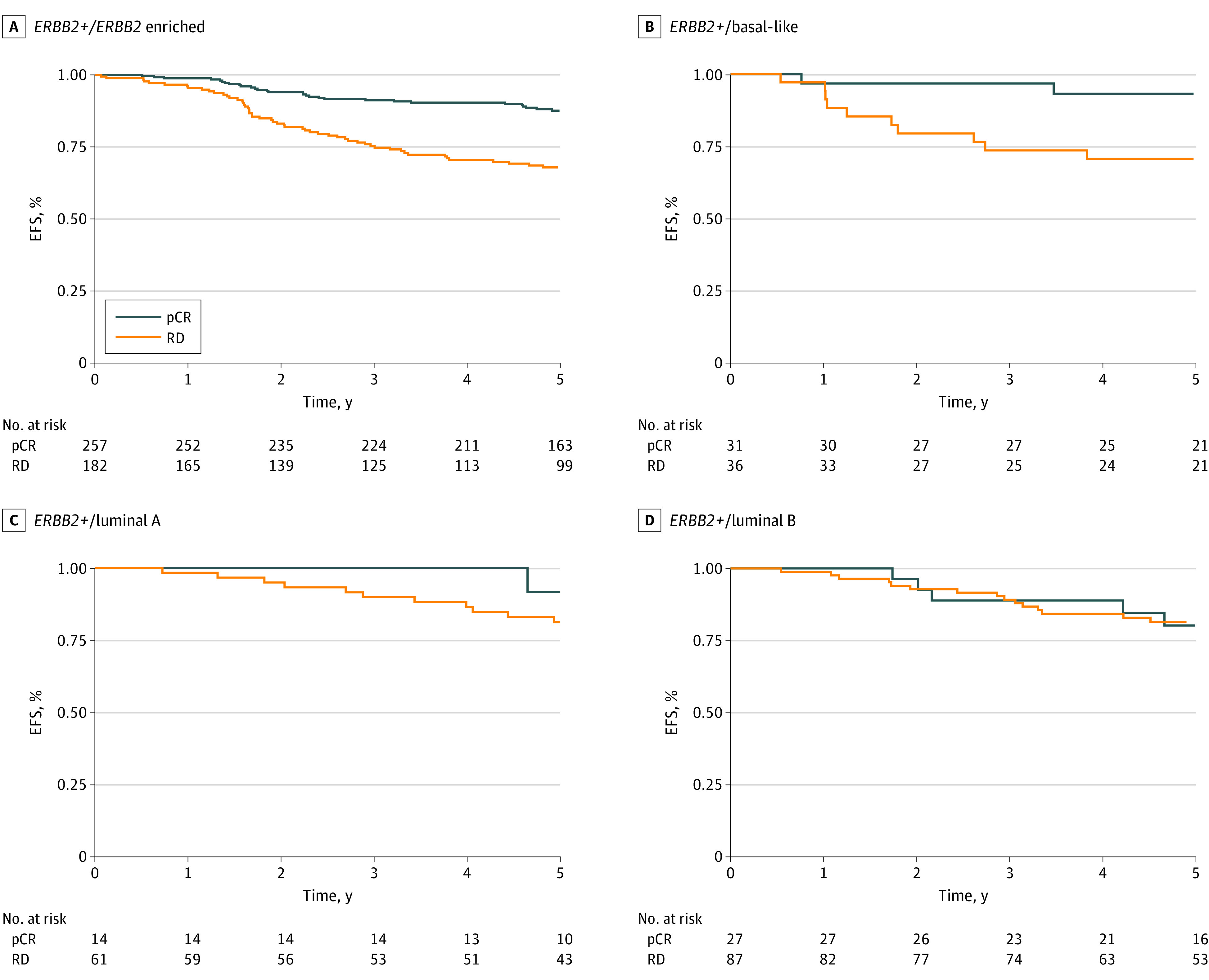
Kaplan-Meier Curves of the Association of Pathologic Complete Response (pCR) With Event-Free Survival by Tumor Intrinsic Subtype Kaplan-Meier event-free survival (EFS) proportions at 5 years are provided. Cox regression models were stratified by clinical trial. Patients with normal-like tumors were removed from the analysis. RD indicates residual disease.

**Figure 2.**
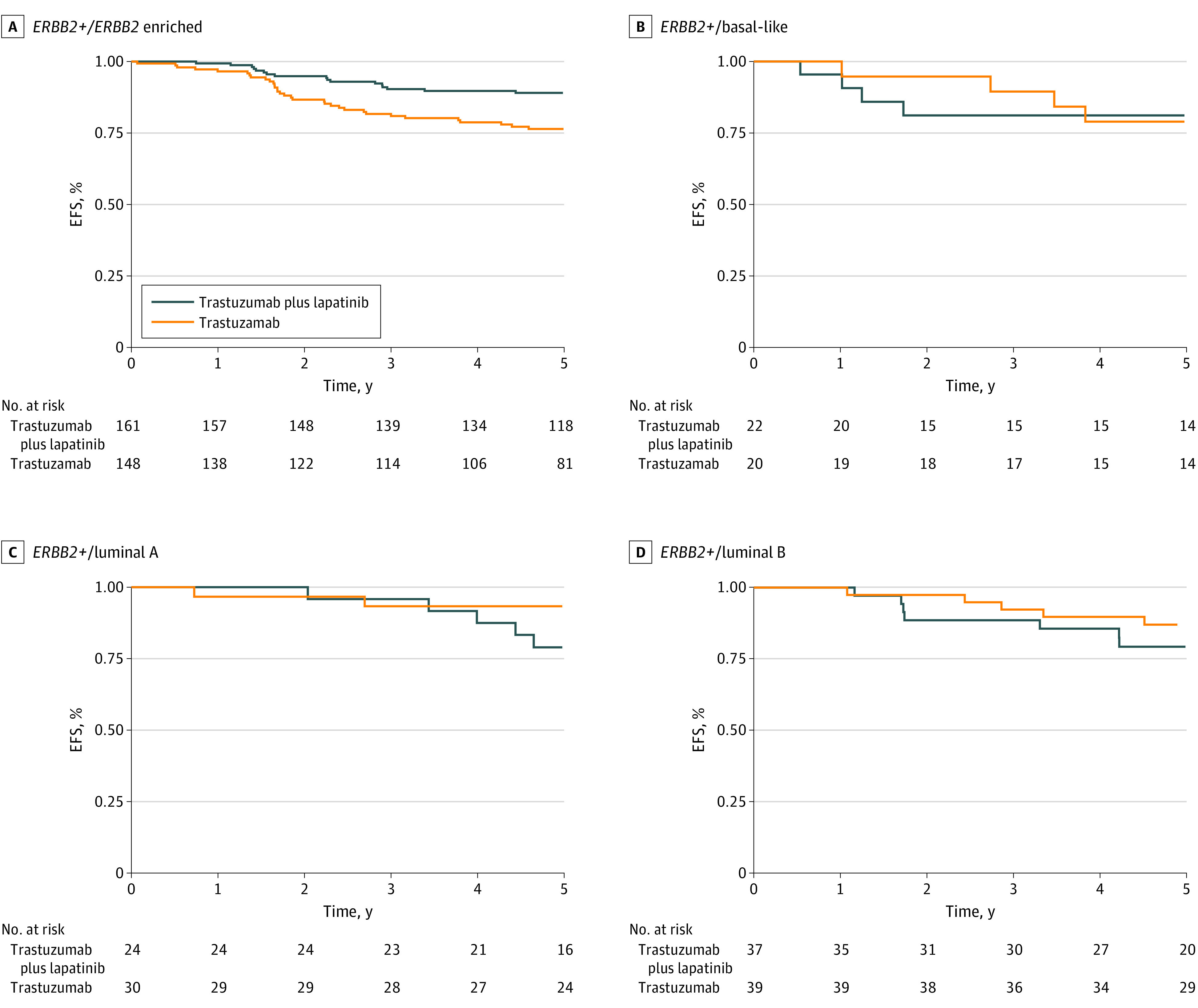
Kaplan-Meier Curves of the Association of the Treatment Arm With Event-Free Survival by Tumor Intrinsic Subtype Kaplan-Meier event-free survival (EFS) proportions at 5 years are provided. Cox regression models were stratified by clinical trial. Patients with normal-like tumors and treated with lapatinib only were removed from the analysis.

The association between pCR and EFS was different by tumor intrinsic subtype. In a multivariable Cox model stratified by study and adjusted by treatment arm, pCR status was significantly associated with EFS in patients with *ERBB2/HER2*-positive/*ERBB2*-enriched (aHR, 0.45; 95% CI, 0.29-0.70; *P* < .001) and *ERBB2/HER2*-positive/basal-like tumors (aHR, 0.19; 95% CI, 0.04-0.86; *P* = .03), but not in patients with *ERBB2/HER2*-positive/luminal A or B disease (Figure 1). Similar results were obtained when performing a landmark analysis (eTable 7 in Supplement 1). In a stratified univariable Cox model, a significant EFS benefit of dual trastuzumab and lapatinib vs single trastuzumab *ERBB2/HER2*-blockade was found only in patients with *ERBB2/HER2*-positive/*ERBB2*-enriched disease (aHR for trastuzumab and lapatinib vs trastuzumab alone, 0.48; 95% CI, 0.27-0.83; *P* = .009) but not in patients with basal-like or luminal *ERBB2/HER2*-positive EBC (Figure 2).

### pCR and EFS Biomarkers Across Individual Trials and in the Combined Cohort

**Figure 3.**
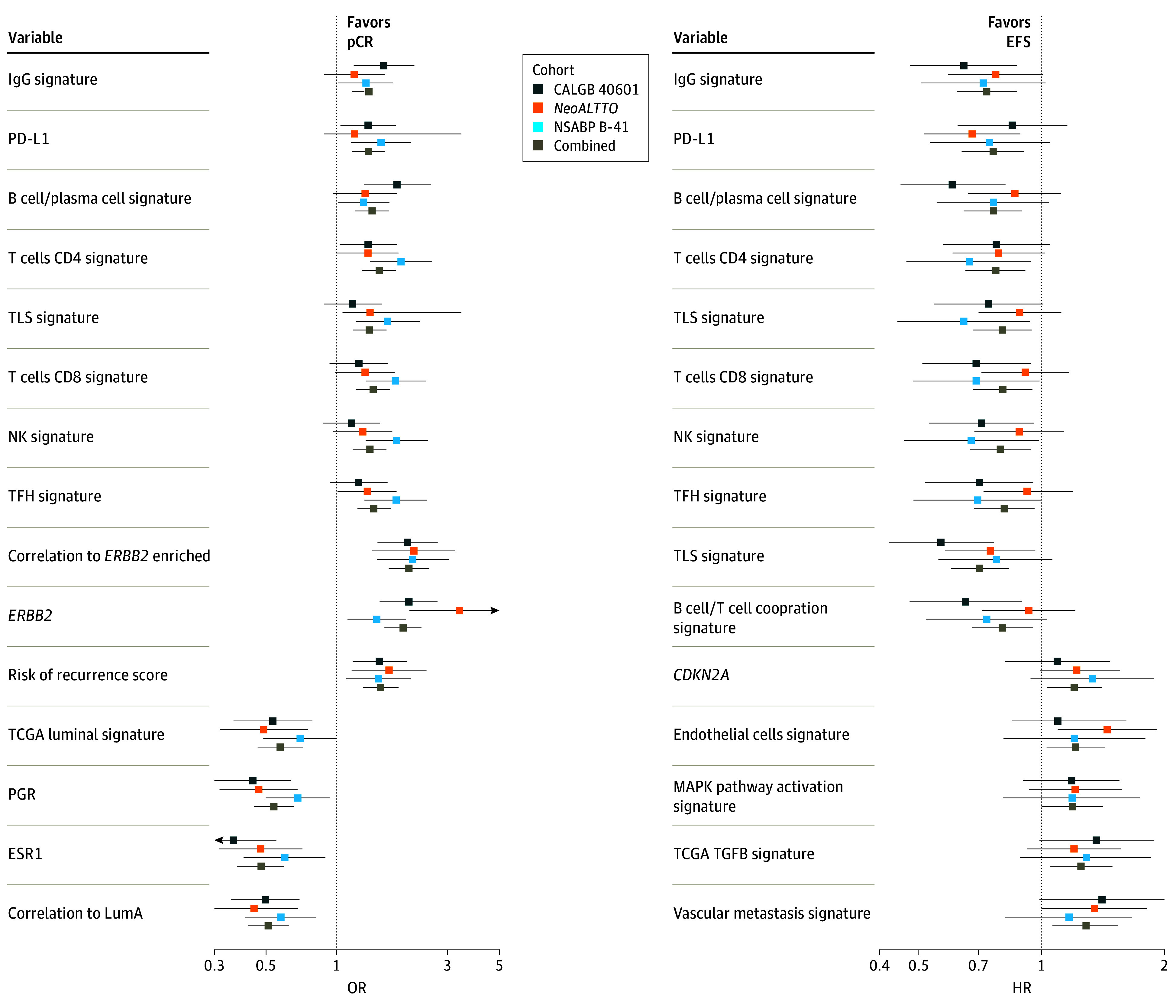
Forest Plots Showing the Association of Gene Expression Biomarker Levels at Baseline With Pathologic Complete Response (PCR) and Event-Free Survival (EFS) A selection of the most interesting and consistent biomarkers across the 3 studies and the combined cohort is shown. All the models have been stratified by clinical trial and adjusted by tumor size, hormone receptor status, and clinical node involvement. The whiskers indicate the 95% CIs. HR indicates hazard ratio; IgG, immunoglobulin G; OR, odds ratio; PD-L1, programmed death-ligand 1.

Using the combined cohort, 275 of 618 gene expression signatures (44.5%) were significantly associated with pCR, and 8 biomarkers were significantly associated with pCR individually in each trial (eTable 8 in Supplement 1). A selection of the signatures more consistently and strongly associated with pCR is shown in Figure 3A. In general, *ERBB2/HER2*, proliferation, and immune-related signatures were associated with higher pCR rates. Some differences were observed between the studies at an immune signature level; for example, B-cell–related signatures were more associated with pCR in the CALGB 40601 trial, whereas T-cell–related signatures were more associated with pCR in the NeoALTTO trial. As expected, luminal-related signatures were associated with lower pCR rates across the 3 studies and the combined cohort.

In multivariable Cox regression analysis, only 9 of 618 signatures (1.5%), all immune, were significantly associated with EFS in the combined cohort when *P* values were adjusted by multiplicity (eTable 9 in Supplement 1). Some of the signatures strongly associated with EFS are represented in Figure 3B. Concordant with their association with pCR, B-cell–related immune gene expression signatures were significantly associated with longer EFS, whereas vascular, proliferation, and metastasis signatures were associated with worse prognosis, although these associations were no longer significant when adjusted for multiple comparisons (eTable 9 in Supplement 1).

Significantly better prognosis was seen in the 409 patients with residual disease if these tumors were HR-positive at baseline (aHR, 0.50; 95% CI, 0.34-0.74; *P*<.001) or luminal expression subtypes (luminal vs HER2-enriched aHR, 0.55; 95% CI, 0.35-0.86; *P* = .01) (eFigure 9A and 9B in Supplement 1). Among patients with residual disease, those with higher immune infiltration at baseline showed a significantly better prognosis. In contrast, high MAPK activation pathway signature levels and the gene expression of *ERBB3* were significantly associated with worse outcomes, although these associations were not significant when adjusted for multiple comparisons (eFigure 9C and eTable 10 in Supplement 1).

## Discussion

This pooled analysis of 3 phase 3 clinical trials with similar designs (ie, NSABP B-41, CALGB 40601, and NeoALTTO) illustrates how the association between pCR and survival in *ERBB2/HER2*-positive EBC varies by genomic intrinsic subtype and is only significant in patients with *ERBB2/HER2*-positive/*ERBB2*-enriched and *ERBB2/HER2*-positive/basal-like tumors. We also found a significant pCR and EFS benefit of dual anti-*ERBB2/HER2* therapy limited to patients with *ERBB2*-enriched tumors, which comprise approximately 60% of *ERBB2/HER2*-positive EBC.

Prognostic and predictive biomarkers are needed in *ERBB2/HER2*-positive EBC to guide the future tailoring of treatment strategies. However, one of the biggest obstacles in biomarker research is the lack of validation. In this individual-level pooled analysis of 758 patients, we were able to test hundreds of gene expression signatures for pCR prediction and outcome prognostication, even though these studies were not powered individually for survival outcomes. *ERBB2* amplicon genes, proliferation, and immune signature levels at baseline were significantly associated with pCR rates in each trial and the combined cohort. In contrast, luminal genes and signatures were significantly associated with lower pCR rates but did not appear to have EFS implications in this setting. Immune signatures were also significantly associated with better EFS outcomes in the combined cohort. These results support our previous findings,^8^ suggesting that the combination of tumor (ie, *ERBB2* amplicon genes, proliferation, and luminal signatures) and immune-related biomarkers provide essential prognostic information to stratify patients with *ERBB2/HER2*-positive EBC in different groups that could benefit from different treatment strategies; some newer commercially available predictors already integrate these elements into a single assay.^24^

### Limitations

This study has several limitations. First, lapatinib is only approved in the metastatic setting but not for *ERBB2/HER2*-positive EBC treatment. Second, even though all 3 trials aimed to test a common hypothesis (ie, if dual *ERBB2/HER2*-blockade with trastuzumab plus lapatinib was better thantrastuzumab alone in terms of pCR), the designs were slightly different: in the NSABP B-41 trial, all chemotherapy was administered before surgery, whereas in the NeoALTTO and CALGB 40601 trials only the taxane component was preoperative, whereas the anthracycline-based regimen was administered after surgery. However, several associations with pCR were consistent across the 3 cohorts. Third, in the NeoALTTO trial, there was a brief induction phase of 6 weeks with only *ERBB2/HER2*-targeted drugs, and T was administered for 12 weeks, compared with 16 weeks in NSABP B-41 and CALGB 40601. Moreover, in the NeoALTTO adjuvant phase, the *ERBB2/HER2*-blockade treatment was the same as in the induction phase and not the standard-of-care trastuzumab for 1 year through most of the trial. The variations in treatment as well as the higher proportion of high clinical risk patients may have contributed to the worse EFS seen in NeoALTTO compared with the other trials. All the models performed in our study have been adjusted and/or stratified by the clinical trial to mitigate these differences and adjusted for key clinical variables. Fourth, the proportions of T4a-c tumors within each study were not studied in this pooled analysis. However, the tumor size assessed by physical examination was not significantly different among the 3 studies. Fifth, even when pooling together patients from 3 trials, the number of EFS events is limited when dividing the study cohort by subgroups, which may result in inadequate statistical power for certain statistical comparisons. Finally, there was a slight difference in the EFS event definition in NSABP B-41, in which progression during the neoadjuvant phase was not counted as an event; however, this was an uncommon occurrence, and we did not find variation in local and distant event proportions across the 3 studies.

## Conclusions

This analysis shows for the first time 2 main clinical implications of tumor intrinsic subtype differences within *ERBB2/HER2*-positive EBC, demonstrating that the association of EFS with pCR after chemotherapy plus *ERBB2/HER2* targeting is seen only in patients with ERBB2-enriched and basal-like tumors and only ERBB2-enriched patients benefit from dual neoadjuvant *ERBB2/HER2*-blockade with trastuzumab and lapatinib. Common biomarkers of pCR and EFS included *ERBB2* amplicon genes and immune gene signatures, whereas in luminal tumors, pCR was less common but had little prognostic implication. Our results highlight the need to incorporate intrinsic subtype and immune gene expression biomarkers to guide personalized treatment in *ERBB2/HER2*-positive EBC.

## Notes

Supplement 1. eFigure 1. Clinical trial designs and CONSORT diagrams eFigure 2. Principal Component Analysis (PCA) plot eFigure 3. Kaplan-Meier (KM) curves eFigure 4. Kaplan-Meier (KM) curves eFigure 5. Kaplan-Meier (KM) curves eFigure 6. Distribution of the types of event-free survival (EFS) events eFigure 7. Distribution of the locoregional and distant relapse events eFigure 8. Distribution of the locoregional and distant relapse events eFigure 9. Association of hormone receptor (HR), intrinsic subtype and gene expression signatures with event-free survival eMethods eReferences eTables

Supplement 2. Data Sharing Statement

## References

[1] Pilewskie M, Morrow M. Axillary nodal management following neoadjuvant chemotherapy: a review. JAMA Oncol. 2017;3(4):549-555. doi:10.1001/jamaoncol.2016.4163 27918753 PMC5580251

[2] von Minckwitz G, Huang CS, Mano MS, ; KATHERINE Investigators. Trastuzumab emtansine for residual invasive her2-positive breast cancer. N Engl J Med. 2019;380(7):617-628. doi:10.1056/NEJMoa1814017 30516102

[3] Cortazar P, Zhang L, Untch M, . Pathological complete response and long-term clinical benefit in breast cancer: the CTNeoBC pooled analysis. Lancet. 2014;384(9938):164-172. doi:10.1016/S0140-6736(13)62422-8 24529560

[4] Baselga J, Bradbury I, Eidtmann H, ; NeoALTTO Study Team. Lapatinib with trastuzumab for HER2-positive early breast cancer (NeoALTTO): a randomised, open-label, multicentre, phase 3 trial. Lancet. 2012;379(9816):633-640. doi:10.1016/S0140-6736(11)61847-3 22257673 PMC5705192

[5] Gianni L, Pienkowski T, Im YH, . Efficacy and safety of neoadjuvant pertuzumab and trastuzumab in women with locally advanced, inflammatory, or early HER2-positive breast cancer (NeoSphere): a randomised multicentre, open-label, phase 2 trial. Lancet Oncol. 2012;13(1):25-32. doi:10.1016/S1470-2045(11)70336-9 22153890

[6] Robidoux A, Tang G, Rastogi P, . Lapatinib as a component of neoadjuvant therapy for HER2-positive operable breast cancer (NSABP protocol B-41): an open-label, randomised phase 3 trial. Lancet Oncol. 2013;14(12):1183-1192. doi:10.1016/S1470-2045(13)70411-X 24095300

[7] Carey LA, Berry DA, Cirrincione CT, . Molecular heterogeneity and response to neoadjuvant human epidermal growth factor receptor 2 targeting in CALGB 40601, a randomized phase III Trial of paclitaxel plus trastuzumab with or without lapatinib. J Clin Oncol. 2016;34(6):542-549. doi:10.1200/JCO.2015.62.1268 26527775 PMC4980567

[8] Fernandez-Martinez A, Krop IE, Hillman DW, . Survival, pathologic response, and genomics in CALGB 40601 (Alliance), a neoadjuvant phase III trial of paclitaxel-trastuzumab with or without lapatinib in HER2-positive breast cancer. J Clin Oncol. 2020;38(35):4184-4193. doi:10.1200/JCO.20.01276 33095682 PMC7723687

[9] Guarneri V, Griguolo G, Miglietta F, Conte PF, Dieci MV, Girardi F. Survival after neoadjuvant therapy with trastuzumab-lapatinib and chemotherapy in patients with HER2-positive early breast cancer: a meta-analysis of randomized trials. ESMO Open. 2022;7(2):100433. doi:10.1016/j.esmoop.2022.100433 35276440 PMC8917305

[10] Piccart-Gebhart M, Holmes E, Baselga J, . Adjuvant lapatinib and trastuzumab for early human epidermal growth factor receptor 2-positive breast cancer: results from the randomized phase III adjuvant lapatinib and/or trastuzumab treatment optimization trial. J Clin Oncol. 2016;34(10):1034-1042. doi:10.1200/JCO.2015.62.1797 26598744 PMC4872016

[11] Piccart M, Procter M, Fumagalli D, ; APHINITY Steering Committee and Investigators. Adjuvant pertuzumab and trastuzumab in early HER2-positive breast cancer in the APHINITY Trial: 6 years’ follow-up. J Clin Oncol. 2021;39(13):1448-1457. doi:10.1200/JCO.20.01204 33539215

[12] Prat A, Carey LA, Adamo B, . Molecular features and survival outcomes of the intrinsic subtypes within HER2-positive breast cancer. J Natl Cancer Inst. 2014;106(8):dju152. doi:10.1093/jnci/dju152 25139534 PMC4151853

[13] Schettini F, Pascual T, Conte B, . HER2-enriched subtype and pathological complete response in HER2-positive breast cancer: a systematic review and meta-analysis. Cancer Treat Rev. 2020;84:101965. doi:10.1016/j.ctrv.2020.101965 32000054 PMC7230134

[14] Swain SM, Tang G, Lucas PC, . Pathologic complete response and outcomes by intrinsic subtypes in NSABP B-41, a randomized neoadjuvant trial of chemotherapy with trastuzumab, lapatinib, or the combination. Breast Cancer Res Treat. 2019;178(2):389-399. doi:10.1007/s10549-019-05398-3 31428908 PMC6797698

[15] Salgado R, Denkert C, Campbell C, . Tumor-infiltrating lymphocytes and associations with pathological complete response and event-free survival in HER2-positive early-stage breast cancer treated with lapatinib and trastuzumab: a secondary analysis of the NeoALTTO Trial. JAMA Oncol. 2015;1(4):448-454. doi:10.1001/jamaoncol.2015.0830 26181252 PMC5551492

[16] Nuciforo P, Pascual T, Cortés J, . A predictive model of pathologic response based on tumor cellularity and tumor-infiltrating lymphocytes (CelTIL) in HER2-positive breast cancer treated with chemo-free dual HER2 blockade. Ann Oncol. 2018;29(1):170-177. doi:10.1093/annonc/mdx647 29045543

[17] Dieci MV, Prat A, Tagliafico E, . Integrated evaluation of PAM50 subtypes and immune modulation of pCR in HER2-positive breast cancer patients treated with chemotherapy and HER2-targeted agents in the CherLOB trial. Ann Oncol. 2016;27(10):1867-1873. doi:10.1093/annonc/mdw262 27484801

[18] Denkert C, von Minckwitz G, Darb-Esfahani S, . Tumour-infiltrating lymphocytes and prognosis in different subtypes of breast cancer: a pooled analysis of 3771 patients treated with neoadjuvant therapy. Lancet Oncol. 2018;19(1):40-50. doi:10.1016/S1470-2045(17)30904-X 29233559

[19] Chic N, Luen SJ, Nuciforo P, . Tumor cellularity and infiltrating lymphocytes as a survival surrogate in HER2-positive breast cancer. J Natl Cancer Inst. 2022;114(3):467-470. doi:10.1093/jnci/djab057 33787900 PMC8902438

[20] Barroso-Sousa R, Barry WT, Guo H, . The immune profile of small HER2-positive breast cancers: a secondary analysis from the APT trial. Ann Oncol. 2019;30(4):575-581. doi:10.1093/annonc/mdz047 30753274 PMC8033534

[21] Fumagalli D, Venet D, Ignatiadis M, . RNA sequencing to predict response to neoadjuvant anti-HER2 therapy: a secondary analysis of the NeoALTTO randomized clinical trial. JAMA Oncol. 2017;3(2):227-234. doi:10.1001/jamaoncol.2016.3824 27684533 PMC5374044

[22] Ignatiadis M, Singhal SK, Desmedt C, . Gene modules and response to neoadjuvant chemotherapy in breast cancer subtypes: a pooled analysis. J Clin Oncol. 2012;30(16):1996-2004. doi:10.1200/JCO.2011.39.5624 22508827

[23] Iglesia MD, Vincent BG, Parker JS, . Prognostic B-cell signatures using mRNA-seq in patients with subtype-specific breast and ovarian cancer. Clin Cancer Res. 2014;20(14):3818-3829. doi:10.1158/1078-0432.CCR-13-3368 24916698 PMC4102637

[24] Prat A, Guarneri V, Pascual T, . Development and validation of the new HER2DX assay for predicting pathological response and survival outcome in early-stage HER2-positive breast cancer. EBioMedicine. 2022;75:103801. doi:10.1016/j.ebiom.2021.103801 34990895 PMC8741424

[25] Solinas C, Ceppi M, Lambertini M, . Tumor-infiltrating lymphocytes in patients with HER2-positive breast cancer treated with neoadjuvant chemotherapy plus trastuzumab, lapatinib or their combination: A meta-analysis of randomized controlled trials. Cancer Treat Rev. 2017;57:8-15. doi:10.1016/j.ctrv.2017.04.005 28525810

[26] Fernandez-Martinez A, Pascual T, Singh B, . Prognostic and predictive value of immune-related gene expression signatures vs tumor-infiltrating lymphocytes in early-stage ERBB2/HER2-positive breast cancer: a correlative analysis of the CALGB 40601 and PAMELA trials. JAMA Oncol. 2023;9(4):490-499. doi:10.1001/jamaoncol.2022.6288 36602784 PMC9857319

[27] de Azambuja E, Holmes AP, Piccart-Gebhart M, . Lapatinib with trastuzumab for HER2-positive early breast cancer (NeoALTTO): survival outcomes of a randomised, open-label, multicentre, phase 3 trial and their association with pathological complete response. Lancet Oncol. 2014;15(10):1137-1146. doi:10.1016/S1470-2045(14)70320-1 25130998

[28] Venet D, Rediti M, Maetens M, . Copy number aberration analysis to predict response to neoadjuvant Anti-HER2 Therapy: results from the NeoALTTO phase III clinical trial. Clin Cancer Res. 2021;27(20):5607-5618. doi:10.1158/1078-0432.CCR-21-1317 34321278

[29] Powles RL, Redmond D, Sotiriou C, . Association of T-cell receptor repertoire use with response to combined trastuzumab-lapatinib treatment of HER2-positive breast cancer: secondary analysis of the NeoALTTO randomized clinical trial. JAMA Oncol. 2018;4(11):e181564. doi:10.1001/jamaoncol.2018.1564 29902299 PMC6224305

[30] Huober J, Holmes E, Baselga J, . Survival outcomes of the NeoALTTO study (BIG 1-06): updated results of a randomised multicenter phase III neoadjuvant clinical trial in patients with HER2-positive primary breast cancer. Eur J Cancer. 2019;118:169-177. doi:10.1016/j.ejca.2019.04.038 31377477

[31] Swain SM, Tang G, Brauer HA, . NSABP B-41, a randomized neoadjuvant trial: genes and signatures associated with pathologic complete response. Clin Cancer Res. 2020;26(16):4233-4241. doi:10.1158/1078-0432.CCR-20-0152 32371537 PMC7724952

[32] Rastogi P, Tang G, Hassan S, . Long-term outcomes of dual vs single HER2-directed neoadjuvant therapy in NSABP B-41. Breast Cancer Res Treat. 2023;199(2):243-252. doi:10.1007/s10549-023-06881-8 36944848 PMC11225589

[33] Giobbie-Hurder A, Gelber RD, Regan MM. Challenges of guarantee-time bias. J Clin Oncol. 2013;31(23):2963-2969. doi:10.1200/JCO.2013.49.5283 23835712 PMC3732313

[34] Lin NU, Borges V, Anders C, . Intracranial efficacy and survival with tucatinib plus trastuzumab and capecitabine for previously treated HER2-positive breast cancer with brain metastases in the HER2CLIMB Trial. J Clin Oncol. 2020;38(23):2610-2619. doi:10.1200/JCO.20.00775 32468955 PMC7403000

