# Supplementary material for: Tumor Intrinsic Subtypes and Gene Expression Signatures in Early-Stage ERBB2/HER2-Positive Breast Cancer: A Pooled Analysis of CALGB 40601, NeoALTTO, and NSABP B-41 Trials
Source: JAMA Oncol. 2024 Mar 28;10(5):603–11. doi: 10.1001/jamaoncol.2023.7304 (PMC10979363; doi:10.1001/jamaoncol.2023.7304)
Supplement: Supplement 1. — eFigure 1. Clinical trial designs and CONSORT diagrams eFigure 2. Principal Component Analysis (PCA) plot eFigure 3. Kaplan-Meier (KM) curves eFigure 4. Kaplan-Meier (KM) curves eFigure 5. Kaplan-Meier (KM) curves eFigure 6. Distribution of the types of event-free survival (EFS) events eFigure 7. Distribution of the locoregional and distant relapse events eFigure 8. Distribution of the locoregional and distant relapse events eFigure 9. Association of hormone receptor (HR), intrinsic subtype and gene expression signatures with event-free survival eMethods eReferences eTables [file jamaoncol-e237304-s001.pdf]

Supplemental Online Content

Fernandez-Martinez A, Rediti M, Tang G, et al. Clinical relevance of tumor intrinsic subtypes and gene expression signatures in early-stage *ERBB2/HER2*-positive breast cancer: a pooled analysis of CALGB 40601, NeoALTTO, and NSABP B-41 Trials. *JAMA Oncol*. Published online March 28, 2024. doi:10.1001/jamaoncol.2023.7304

eFigure 1. Clinical trial designs and CONSORT diagrams
eFigure 2. Principal Component Analysis (PCA) plot
eFigure 3. Kaplan-Meier (KM) curves
eFigure 4. Kaplan-Meier (KM) curves
eFigure 5. Kaplan-Meier (KM) curves
eFigure 6. Distribution of the types of event-free survival (EFS) events
eFigure 7. Distribution of the locoregional and distant relapse events
eFigure 8. Distribution of the locoregional and distant relapse events
eFigure 9. Association of hormone receptor (HR), intrinsic subtype and gene expression signatures with event-free survival
eMethods
eReferences
eTables

This supplemental material has been provided by the authors to give readers additional information about their work.

Online-only Figures

eFigure 1. Clinical trial designs and CONSORT diagrams

1A: Clinical trial schemas. EBC: early breast cancer; H: trastuzumab; L: lapatinib; wT: weekly paclitaxel; dd: dose-dense; AC: adriamycin and cyclophosphamide; FEC: fluorouracil, epirubicin, cyclophosphamide. 1B: CONSORT diagram of the intention-to-treat (ITT), RNA-sequencing (RNA-seq), and Landmark cohorts. RNAseq: RNA sequencing; pCR: pathologic complete response; EFS: event-free survival; 30w: 30 weeks.

A

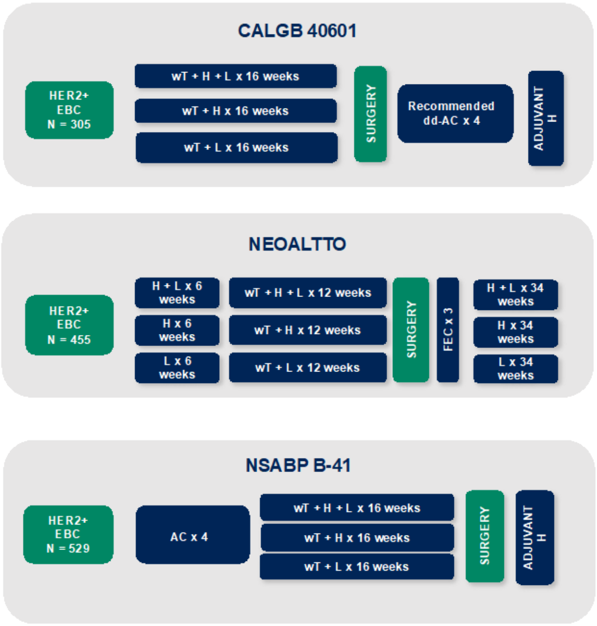

B

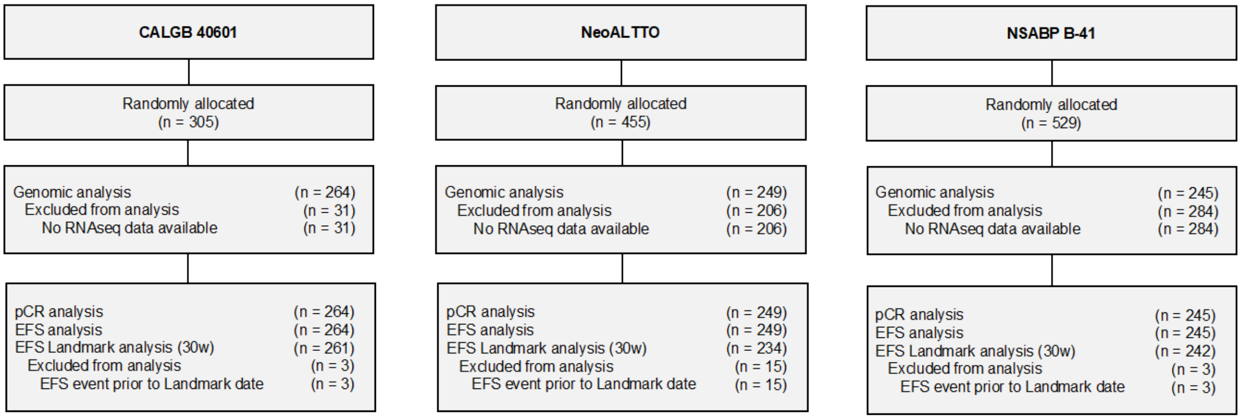

© 2024 Fernandez-Martinez et al. *JAMA Oncology.*

eFigure 2. Principal Component Analysis (PCA) plot

Principal Component Analysis (PCA) plot showing the batch effect among the gene expression data from the three clinical trials (A, B, C) and the batch effect correction (D, E, F) after applying the Distance Weight Discrimination (DWD) method to the RNA sequencing cohort (N = 758). Only PC 1 vs. PC 2 is shown. Samples were colored by clinical trial (A, D), estrogen-receptor (ER) status (B, E), and intrinsic subtype (C, F). PC: principal component; ER: estrogen receptor; Basal: Basal-like; HER2-E: HER2-Enriched; LumA: Luminal A; LumB: Luminal B; Normal: Normal-like.

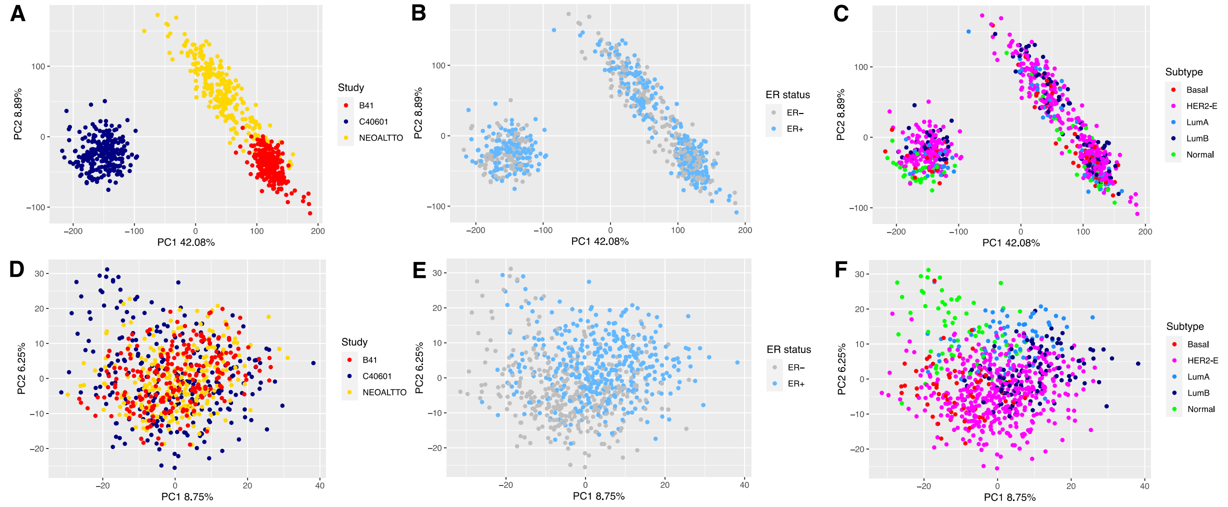

© 2024 Fernandez-Martinez et al. *JAMA Oncology.*

eFigure 3. Kaplan-Meier (KM) curves

Kaplan-Meier (KM) curves showing the association of the treatment arm with event-free survival (EFS) in the intention-to-treat (ITT) cohort with EFS information (N = 1,279). KM estimates of EFS at 5 years are provided. H: trastuzumab; L: lapatinib; EFS: event-free survival; N: number; 5-yr: 5 years; CI: confident interval.

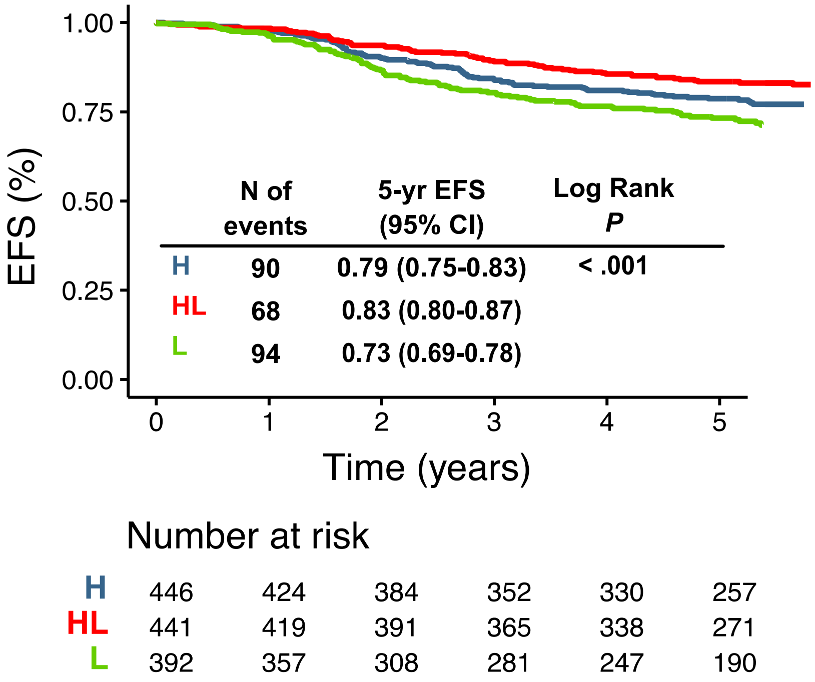

© 2024 Fernandez-Martinez et al. *JAMA Oncology.*

eFigure 4. Kaplan-Meier (KM) curves

Kaplan-Meier (KM) curves showing the differences in event-free survival (EFS) between the three clinical trials in the intention to treat (ITT) population with EFS data (N = 1,279). KM EFS proportions at 5 years are provided. A univariable Cox regression model results is shown. EFS: event-free survival; N: number of events; 5-yr: 5 years; HR: hazard ratio; CI: confidence interval.

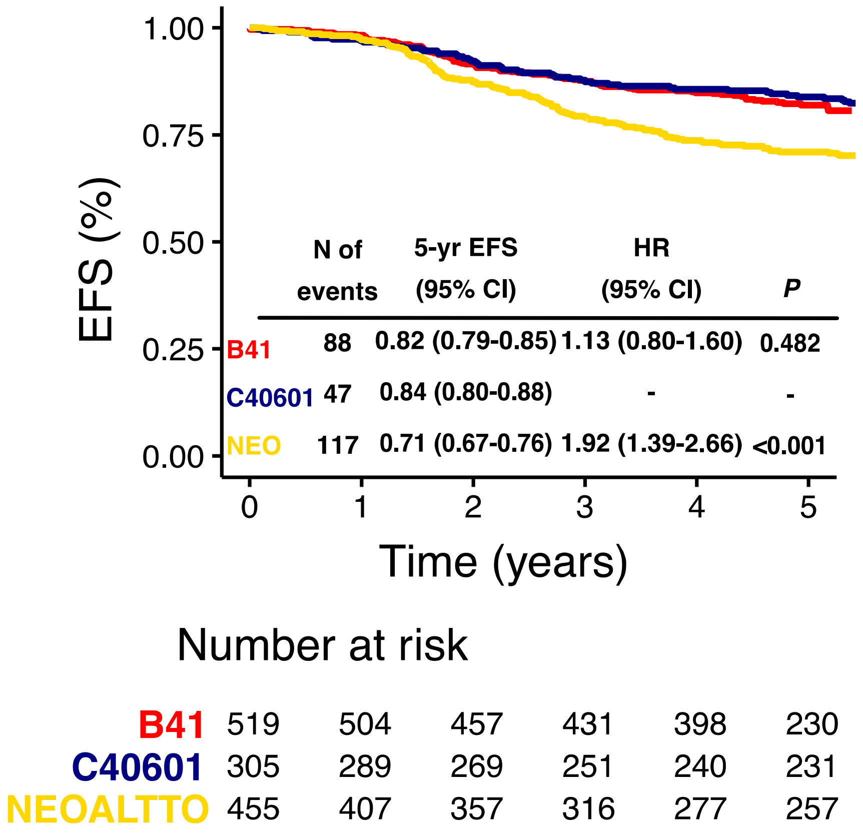

© 2024 Fernandez-Martinez et al. *JAMA Oncology.*

eFigure 5. Kaplan-Meier (KM) curves

Kaplan-Meier (KM) curves showing the differences in event-free survival (EFS) between patients with pathologic complete response (pCR) and patients with residual disease (RD). Patients treated only with lapatinib in the neoadjuvant setting have been removed for this analysis. **5A**: intention to treat population with EFS data (N = 887). 5B: Landmark population at 30 weeks with EFS data (N = 856). KM EFS proportions at 5 years are provided. The adjusted hazard ratio (HR) from a multivariable Cox regression model stratified by clinical trial and adjusted by treatment arm, hormone receptor status, tumor size and node status is provided. EFS: event-free survival; N: number; 5-yr: 5 years; HR: hazard ratio; CI: confidence interval.

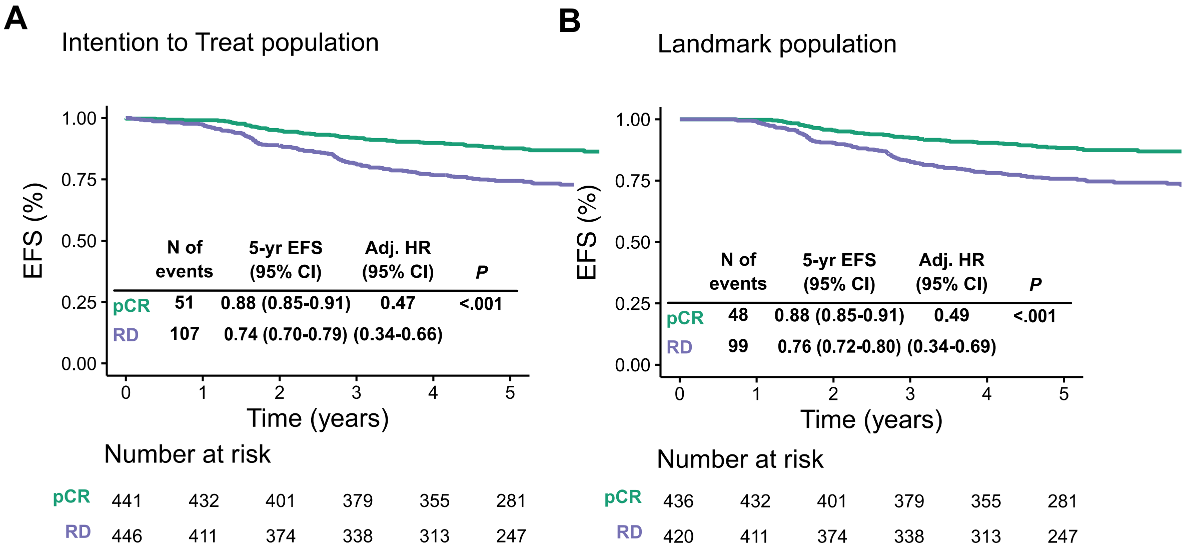

© 2024 Fernandez-Martinez et al. *JAMA Oncology.*

eFigure 6. Distribution of the types of event-free survival (EFS) events

Distribution of the types of event-free survival (EFS) events in the intention-to-treat (ITT) cohort by clinical trial. Distribution of the EFS events in the ITT cohort from **6A**: NSABP B-41, **6B**: CALGB 40601, and **6C**: NeoALTTO. **6D**: bar plot showing the proportion of events grouped into three categories (i.e., distant recurrence, local-regional recurrence, and others). The *P* from a Chi-square test comparing proportions across the three trials is provided. M1: metastasis.

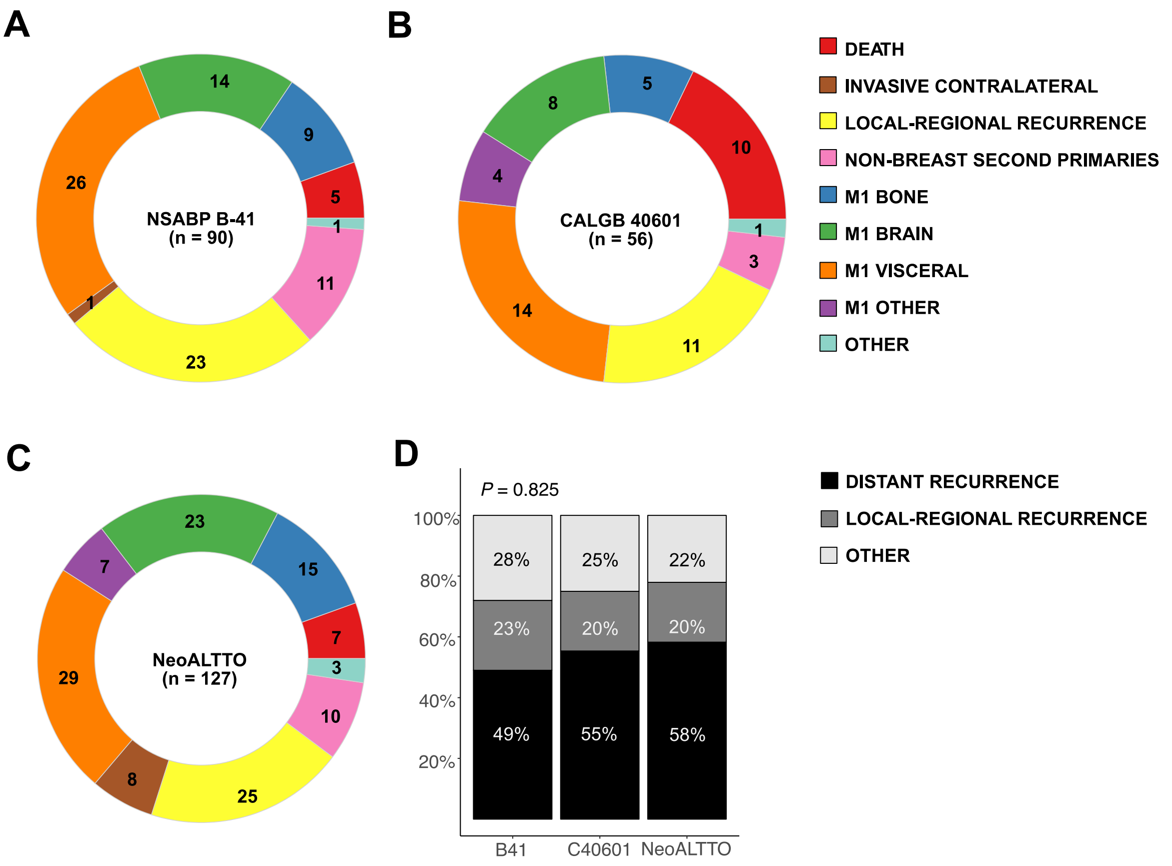

© 2024 Fernandez-Martinez et al. *JAMA Oncology.*

eFigure 7. Distribution of the locoregional and distant relapse events

Distribution of the locoregional and distant relapse events in the intention-to-treat (ITT) cohort by treatment arm. For distant relapses, patients with visceral relapses and other metastasis were considered “M1 visceral”; patients with brain and other metastasis were classified as “M1 brain”; the rest of the patients with distant relapses were considered “M1 bone” if they had bone metastasis or “M1 other” if they had a different metastatic relapse (i.e., soft tissue). M1: metastasis; H: trastuzumab; L: lapatinib.

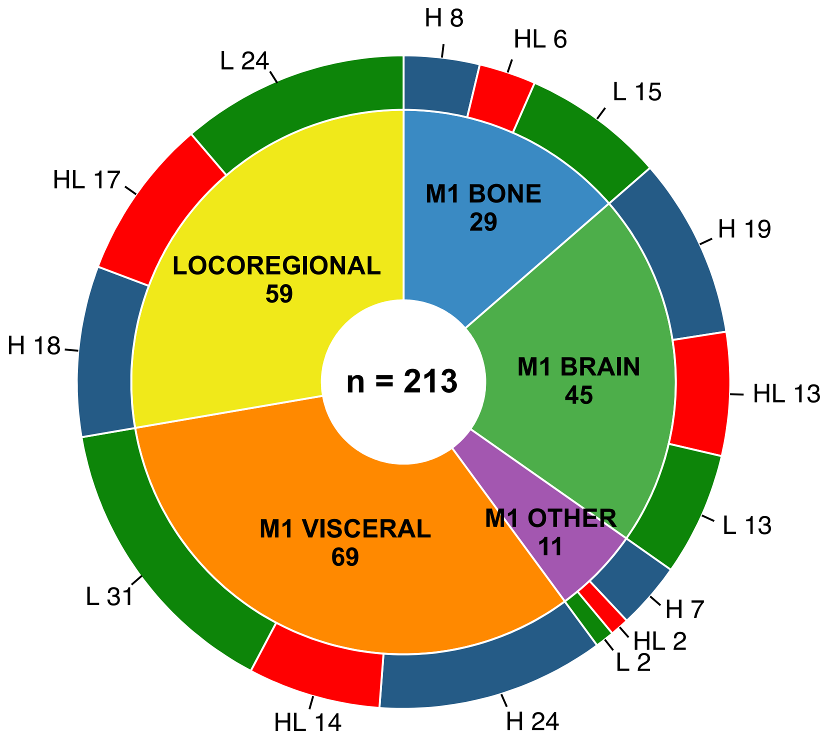

	M1 BONE<br>N = 29	M1 BRAIN<br>N = 45	M1 OTHER<br>N = 11	M1 VISCERAL<br>N = 69	LOCOREGIONAL<br>N = 59	*P*[1]
**Treatment**						0.32
H	8 (27.6%)	19 (42.2%)	7 (63.6%)	24 (34.8%)	18 (30.5%)	
HL	6 (20.7%)	13 (28.9%)	2 (18.2%)	14 (20.3%)	17 (28.8%)	
L	15 (51.7%)	13 (28.9%)	2 (18.2%)	31 (44.9%)	24 (40.7%)	

[1]Fisher's exact test

© 2024 Fernandez-Martinez et al. *JAMA Oncology.*

eFigure 8. Distribution of the locoregional and distant relapse events

Distribution of the locoregional and distant relapse events by hormone receptor (HR) status and tumor intrinsic subtype in the intention to treat (ITT) and RNAseq cohorts, respectively. **8A**: Distribution of distant relapse events by HR status in the intention-to-treat (ITT) cohort. **8B**: Distribution of distant relapse events by intrinsic subtype in the RNA sequencing (RNAseq) cohort. For panel B, Normal-like tumors were removed. For distant relapses, patients with visceral relapses and other metastasis were considered "M1 visceral"; patients with brain metastasis and other metastasis were classified as "M1 brain"; the rest of the patients with distant relapses were considered "M1 bone" if they had bone metastasis or "M1 other" if they had another metastatic relapse (i.e., soft tissue). M1: metastasis; HR-pos: hormone receptor-positive; HR-neg: hormone receptor-negative; LumA: Luminal A; LumB: Luminal B; HER2-E: HER2-Enriched; Basal: Basal-like.

A

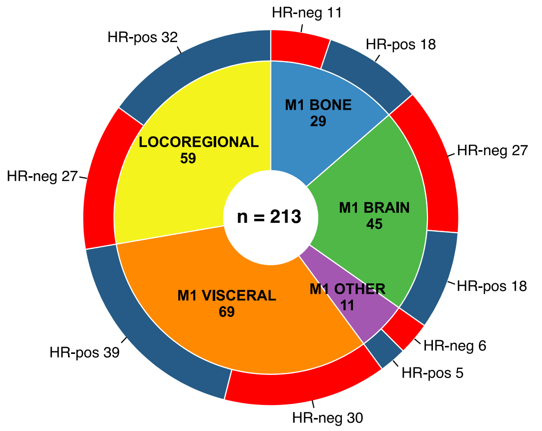

	M1 BONE N = 29	M1 BRAIN N = 45	M1 OTHER N = 11	M1 VISCERAL N = 69	LOCOREGIONAL N = 59	*P*[1]
**HR status**						0.33
HR-neg	11 (37.9%)	27 (60.0%)	6 (54.5%)	30 (43.5%)	27 (45.8%)	
HR-pos	18 (62.1%)	18 (40.0%)	5 (45.5%)	39 (56.5%)	32 (54.2%)	

[1]Fisher's exact test

B

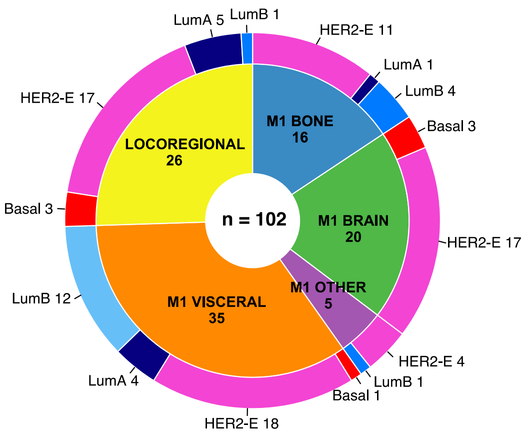

	M1 BONE N = 16	M1 BRAIN N = 20	M1 OTHER N = 5	M1 VISCERAL N = 35	LOCOREGIONAL N = 26	*P*[1]
**Subtype**						0.004
Basal	0 (0.0%)	3 (15.0%)	0 (0.0%)	1 (2.9%)	3 (11.5%)	
HER2-E	11 (68.8%)	17 (85.0%)	4 (80.0%)	18 (51.4%)	17 (65.4%)	
LumA	1 (6.2%)	0 (0.0%)	0 (0.0%)	4 (11.4%)	5 (19.2%)	
LumB	4 (25.0%)	0 (0.0%)	1 (20.0%)	12 (34.3%)	1 (3.8%)	

[1]Fisher's exact test

© 2024 Fernandez-Martinez et al. *JAMA Oncology.*

eFigure 9. Association of hormone receptor (HR), intrinsic subtype and gene expression signatures with event-free survival

Association of hormone receptor (HR), intrinsic subtype and gene expression signatures with event-free survival (EFS) in the cohort of patients with residual disease. **9A:** Kaplan-Meier (KM) curves of the association of HR status at baseline with EFS in the cohort of patients with residual disease and RNA sequencing (RNAseq) data (N = 409). KM EFS proportions at 5 years are provided. The Cox model have been stratified by study. **9B**: Kaplan-Meier (KM) curves of the association of intrinsic subtype at baseline with EFS in the cohort of patients with residual disease and RNAseq data. Normal-like tumors have been removed for this analysis (N = 363) KM EFS proportions at 5 years are provided. The Cox model have been stratified by study. **9C**: Forest plot showing the association of gene expression biomarker levels at baseline with EFS in the cohort of patients with residual disease and RNAseq data (N = 409). A selection of the most strong and consistent biomarkers is shown. All the Cox models have been stratified by clinical trial and adjusted by tumor size, HR status, and clinical node involvement. EFS: event-free survival; *N*: number of events; 5-yr: 5 years; HR: hazard ratio; CI: confident interval. HR-pos: HR-positive; HR-neg: HR-negative; Basal: HER2-E: HER2-Enriched; Lum: Luminal A and Luminal B; IgG: immunoglobulin G; NK Natural Killer.

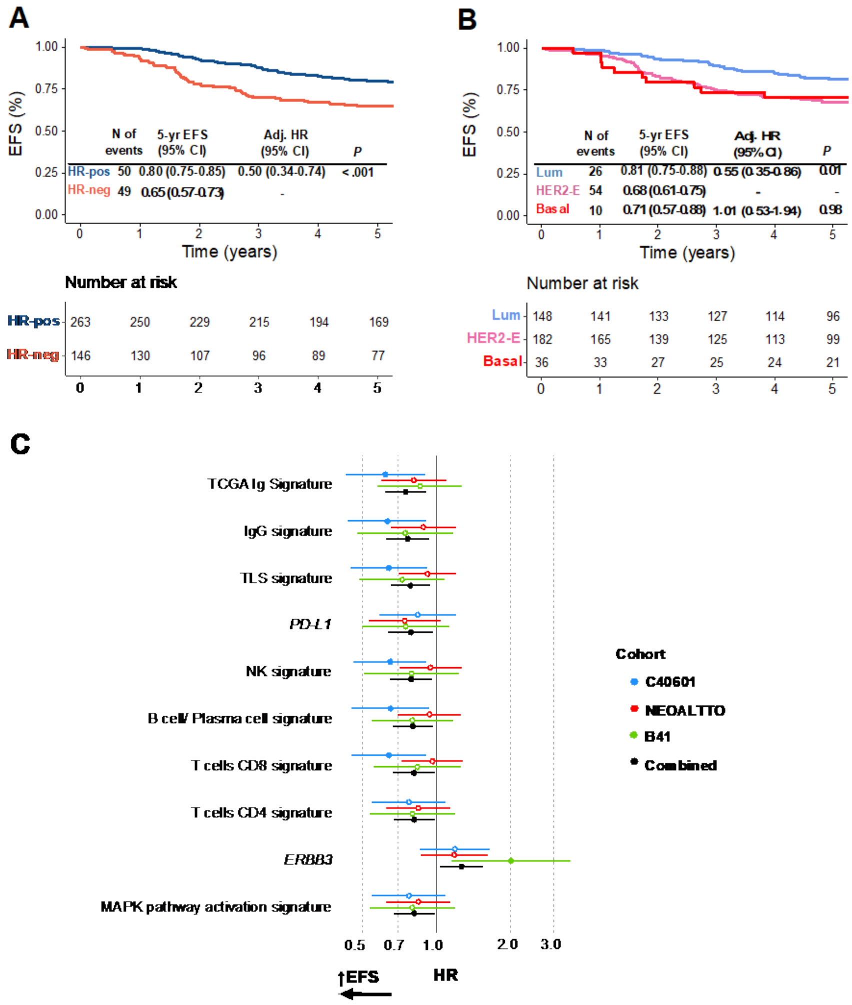

© 2024 Fernandez-Martinez et al. *JAMA Oncology.*

eMethods

Biomarker versus intention-to-treat (ITT) subpopulation.

Gene expression profiles from pretreatment core biopsies were obtained from 249/455 (54.7%) NeoALTTO, 264/305 (86.6%) CALGB 40601, and 245/529 (46.3%) of NSABP B-41 participants, respectively. Due to the different proportions of patients included in the RNAseq biomarker analysis in each clinical trial, CALGB 40601 was the trial more represented in the RNAseq cohort, while NSABP B-41 was more represented in the ITT cohort. Apart from this, there were no significant differences between the clinicopathologic characteristics, response, and EFS survival outcomes of the parent ITT and the RNA sequencing (RNAseq) cohorts (**Supplementary Methods Table 1**).

RNA sequencing and expression quantification.

For CALGB 40601, RNAseq was performed at the University of North Carolina (UNC) High-Throughput Sequencing Facility. Briefly, RNA was extracted from fresh-frozen baseline tumor samples, RNAseq libraries were made using Illumina TruSeq® mRNA kit, and sequenced on an Illumina HiSeq 2000 system using a 2x50bp configuration. For NeoALTTO, RNAseq was performed at GATC Biotech AG. Briefly, RNA was extracted from fresh-frozen baseline tumor samples, Ribo-Zero RNAseq libraries were constructed using the NEBNext Ultra directional RNA library preparation kit and sequenced on the HiSeq 2500 system using a 2x100bp configuration. For NSABP B-41, RNA extraction and RNAseq libraries were made at Georgetown University Genomics and Epigenomics Share Resource (GESR), and the sequencing was performed at Intermountain Precision Genomics. Briefly, RNA was extracted from paraffin-embedded baseline tumor samples, ribo-zero RNAseq libraries were made using an Illumina TruSeq Stranded Total RNA kit and sequenced on an Illumina NovaSeq6000 system using a 2x100bp configuration.

All three trials' RNAseq fastq files were processed by the UNC Lineberger Comprehensive Cancer Center Bioinformatics Core following the same algorithm. Purity-filtered reads were aligned to the human reference GRCh38/hg38 genome using Spliced Transcripts Aligned to a Reference (STAR) version 2.4.2a[1]. Transcript abundance estimates were generated by Salmon version 0.6.0[2] in '-quant' mode, based on the STAR alignments. Raw read counts for all RNAseq samples were normalized to a fixed upper quartile[3]. RNAseq normalized gene counts were then log2 transformed, and genes were filtered for those expressed in 70% of samples for each data set. Genes in common were found for the three studies, and the batch effect between the three studies was corrected by applying the Distance Weight Discrimination (DWD) method[4, 5] version 1.0.2[6], using CALGB 40601 as reference (**Supplementary Methods Figure 1**).

Intrinsic subtypes.

For the three studies, intrinsic subtypes were obtained from RNAseq gene expression data as described before[7]. The Python code to apply the PAM50 gene normalization can be found at https://github.com/afernan4/PAM50_ER_HER2_normalization.

Gene expression signatures.

© 2024 Fernandez-Martinez et al. *JAMA Oncology.*

A collection of 618 previously published gene expression signatures was calculated. The signature scores were computed by extracting the median expression of all the genes within a signature. The immune signatures are summarized in **Supplementary Methods Table 2**. The R code to calculate the signatures can be found at https://github.com/afernan4/iGES_scores.

© 2024 Fernandez-Martinez et al. *JAMA Oncology.*

eReferences

1 Dobin A, Davis CA, Schlesinger F et al. STAR: ultrafast universal RNA-seq aligner. Bioinformatics 2013; 29 (1): 15-21.

2 Patro R, Duggal G, Love MI et al. Salmon provides fast and bias-aware quantification of transcript expression. Nat Methods 2017; 14 (4): 417-419.

3 Bullard JH, Purdom E, Hansen KD et al. Evaluation of statistical methods for normalization and differential expression in mRNA-Seq experiments. BMC Bioinformatics 2010; 11: 94.

4 Marron JS, Todd MJ, Ahn J. Distance-Weighted Discrimination. Journal of the American Statistical Association 2007; 102 (480): 1267-1271.

5 Wang B, Hui Zou. Another look at distance-weighted discrimination. Journal of the Royal Statistical Society: Series B (Statistical Methodology) 2018; 80 (1): 177-198.

6 Carmichael I. DWD. GitHub repository 2021.

7 Fernandez-Martinez A, Krop IE, Hillman DW et al. Survival, Pathologic Response, and Genomics in CALGB 40601 (Alliance), a Neoadjuvant Phase III Trial of Paclitaxel-Trastuzumab With or Without Lapatinib in HER2-Positive Breast Cancer. J Clin Oncol 2020: JCO2001276.

© 2024 Fernandez-Martinez et al. *JAMA Oncology.*

eTable 1
List of gene expression signatures and publication PubMed Unique Identifier (PMID).

Signaturename_Journal.Year|PMID	Module type
Activate.Endothelium_Clin.Exp.Metastasis.2014|PMID.23975155	median
Activated.B.cell_CellRep.2017|PMID.28052254	median
Activated.Blood.Neutrophil.Signature_Nat.Cell.Biol.2019|PMID.31263265	median
Activated.Cancer.Cell.Signature_Nat.Cell.Biol.2019|PMID.31263265	median
Activated.CD4.T.cell_CellRep.2017|PMID.28052254	median
Activated.CD8.T.cell_CellRep.2017|PMID.28052254	median
Activated.dendritic.cell_CellRep.2017|PMID.28052254	median
Activated.Lung.MSC.Signature_Nat.Cell.Biol.2019|PMID.31263265	median
Activated.Lung.Neutrophil.Signature_Nat.Cell.Biol.2019|PMID.31263265	median
aDC_Immunity.2013_PMID.24138885|PMID.24138885	median
ADM.S100A10.A110NDGR1.Cluster_BMC.Med.Genomics.2011|PMID.21214954	median
African.and.European.Ancestry.TCGA.Negative_JAMA.Oncol.2017|PMID.28472234	median
African.and.European.Ancestry.TCGA.Positive_JAMA.Oncol.2017|PMID.28472234	median
Age.associated.signature_Genome.Biol.2015|PMID.26343147	median
aMaSC_BCR.2010|PMID.20346151	median
aMaSC.HsEnriched_BCR.2015|PMID.25575446	median
aMaSC.HsEnriched.Refined1_BCR.2015|PMID.25575446	median
aMaSC.Lim09_BCR.2015|PMID.25575446	median
aMaSC.Prat_BCR.2015|PMID.25575446	median
aMaSC.Shehata_BCR.2015|PMID.25575446	median
aMaSC.Signature_Cell.Stem.Cell.2012|PMID.22305568	median
AMPH.EPIREGULIN.Cluster_BMC.Med.Genomics.2011|PMID.21214954	median
Amplification.50_Genome.Biol.2014|PMID.25164602	median
Amplification.50.better.than._Genome.Biol.2015|PMID.25164602	median
Apocrine.Features_J.Pathol.2017|PMID.27861902	median
aStr.HsEnriched_BCR.2015|PMID.25575446	median
aStr.HsEnriched.Refined1_BCR.2015|PMID.25575446	median
aStr.HsEnriched.Refined2_BCR.2015|PMID.25575446	median
aStr.Lim09_BCR.2015|PMID.25575446	median
aStr.Prat_BCR.2015|PMID.25575446	median
aStr.Shehata_BCR.2015|PMID.25575446	median
BASAL.Cluster_BMC.Med.Genomics.2011|PMID.21214954	median
Bcell.cluster_CCR.2014|PMID.24916698	median
Bcell.IL10.MINUS_Immunol.2014|PMID.25080484	median

Bcell.IL10.PLUS_Immunol.2014|PMID.25080484	median
Bcell.lineage.MCP_Nature.2020|PMID.31942077	median
Bcell.Plasma.52gene_Genome.Biol.2013|PMID.23618380	median
Bcell.Plasma.Metagene_Genome.Biol.2013|PMID.23618380	median
Bcell.Tcell.Cooperation_Cell.2019|PMID.31730857	median
Bcells_CancerImmunolRes.2018|PMID.30266715	median
Bcells_Immunity.2013|PMID.24138885	median
Bcells.Centroblast_JCO.2015|PMID.25800755	median
Bcells.Centrocyte_JCO.2015|PMID.25800755	median
Bcells.Memory_JCO.2015|PMID.25800755	median
Bcells.memory_Nat.Methods.2015|PMID.25822800	median
Bcells.Naive_JCO.2015|PMID.25800755	median
Bcells.naive_Nat.Methods.2015|PMID.25822800	median
Bcells.Plasmablast_JCO.2015|PMID.25800755	median
Blood.vessels_Immunity.2013|PMID.24138885	median
bMYB.Signature_Oncogene.2009|PMID.19043454	median
C3TAG.Responding_CCR.2013|PMID.23780888	median
C3TAG.Untreated_CCR.2013|PMID.23780888	median
CD103.Negative_Cancer.Cell.2014|PMID.25446897	median
CD103.Positive_Cancer.Cell.2014|PMID.25446897	median
CD103.Ratio_Cancer.Cell.2014|PMID.25446897	median
CD274_Single gene|Single	single gene
CD34.CD36.Cluster_BMC.Med.Genomics|PMID.21214954	median
CD44.downregulated.genes_Cancer.Cell.2007|PMID.17349583	median
CD44.upregulated.genes_Cancer.Cell.2007|PMID.17349583	median
CD56bright.natural.killer.cell_CellRep.2017|PMID.28052254	median
CD56dim.natural.killer.cell_CellRep.2017|PMID.28052254	median
CD68.cluster_CCR.2014|PMID.24916698	median
CD8.cluster_CCR.2014|PMID.24916698	median
CDKN2A_Single gene|Single	single gene
Central.memory.CD4.T.cell_CellRep.2017|PMID.28052254	median
Central.memory.CD8.T.cell_CellRep.2017|PMID.28052254	median
CES.Score_CCR.2017|PMID.27903675	special
Chromogramin_BMC.Med.Genomics.2011|PMID.21214954	median
CIN70_Nat.Genet.2006|PMID.16921376	median
Claudin.High_Genome.Biol.2007|PMID.17493263	median
Claudin.Low_Genome.Biol.2007|PMID.17493263	median
Claudin.Low.29_Cancer.Res.2009|PMID.19435916	median

cMYB.Signature_PLoS.One.2010|PMID.20949095	median
CORE.Bcell.signature.Garber_Cell.Mol.Gastroenterol.Hepatol.2017|PMID.28508029	median
CTLA4_Single gene|Single	single gene
Cytolytic.activity_Cell.2015|PMID.25594174	median
Cytotoxic.cells_Immunity.2013|PMID.24138885	median
Day7.Downregulated_Nat.Cell.Biol.2014|PMID.25173976	median
Day7.Upregulated_Nat.Cell.Biol.2014|PMID.25173976	median
DC_Immunity.2013|PMID.24138885	median
DCIS.HGF.down_BCR.2013|PMID.24025166	median
DCIS.HGF.up_BCR.2014|PMID.24025166	median
Delection.50_Genome.Biol.2016|PMID.25164602	median
Delection.50.better.than_Genome.Biol.2017|PMID.25164602	median
Dendritic.cells.activated_Nat.Methods.2015|PMID.25822800	median
Dendritic.cells.resting_Nat.Methods.2015|PMID.25822800	median
Down.Basal.High_Nat.Cell.Biol.2014|PMID.25173976	median
Down.CLOW.High_Nat.Cell.Biol.2014|PMID.25173976	median
Downregulated.upon.NRAS.repression.basal_Cell.Rep.2015|PMID.26166574	median
Ductal.Carcinoma.In.Situ_J.Pathol.2017|PMID.27861902	median
Duke.Module01.acidosis_PNASUSA.2010|PMID.20335537	median
Duke.Module02.akt_PNASUSA.2010|PMID.20335537	median
Duke.Module03.betacatenin_PNASUSA.2010|PMID.20335537	median
Duke.Module04.E2F1_PNASUSA.2010|PMID.20335537	median
Duke.Module05.EGFR_PNASUSA.2010|PMID.20335537	median
Duke.Module06.ER_PNASUSA.2010|PMID.20335537	median
Duke.Module07.glucosedepletion_PNASUSA.2010|PMID.20335537	median
Duke.Module08.HER2_PNASUSA.2010|PMID.20335537	median
Duke.Module09.hypoxia_PNASUSA.2010|PMID.20335537	median
Duke.Module10.IFNA_PNASUSA.2010|PMID.20335537	median
Duke.Module11.IFNG_PNASUSA.2010|PMID.20335537	median
Duke.Module12.lacticacidosis_PNASUSA.2010|PMID.20335537	median
Duke.Module13.myc_PNASUSA.2010|PMID.20335537	median
Duke.Module14.p53_PNASUSA.2010|PMID.20335537	median
Duke.Module15.p63_PNASUSA.2010|PMID.20335537	median
Duke.Module16.pi3k_PNASUSA.2010|PMID.20335537	median
Duke.Module17.PR_PNASUSA.2010|PMID.20335537	median
Duke.Module18.ras_PNASUSA.2010|PMID.20335537	median
Duke.Module19.src_PNASUSA.2010|PMID.20335537	median
Duke.Module20.STAT3_PNASUSA.2010|PMID.20335537	median

Duke.Module21.TGFB_PNASUSA.2010|PMID.20335537	median
Duke.Module22.TNFA_PNASUSA.2010|PMID.20335537	median
Durvalumab.signature_CCR.2018|PMID.29716923	median
Early.IRS.1_PLoS.One.2016|PMID.26991655	median
Early.IRS.2_PLoS.One.2016|PMID.26991655	median
Early.Relapse.ERPos.33genes_JAMA.2011|PMID.21558518	median
Early.Response.ERNeg.27genes_JAMA.2011|PMID.21558518	median
Effector.memeory.CD4.T.cell_CellRep.2017|PMID.28052254	median
Effector.memeory.CD8.T.cell_CellRep.2017|PMID.28052254	median
EGFR_Single gene|Single	single gene
EMT.down.Taube_PNAS.2010|PMID.20713713	median
EMT.down.Weingberg_PNAS.2010|PMID.20713713	median
EMT.up.Taube_PNAS.2010|PMID.20713713	median
EMT.up.Weinberg_PNAS.2010|PMID.20713713	median
Endothelial.cells.MCP_Nature.2020|PMID.31942077	median
Endothelial.Normal_Angiogenesis.2014|PMID.24257808	median
Endothelial.Tumor_Angiogenesis.2014|PMID.24257808	median
Eosinophil_CellRep.2017|PMID.28052254	median
Eosinophils_Immunity.2013|PMID.24138885	median
Eosinophils_Nat.Methods.2015|PMID.25822800	median
Epithelial.Tubule.Formation_J.Pathol.2017|PMID.27861902	median
ERBB2_Single gene|Single	single gene
ERBB3_Single gene|Single	single gene
ESR1_Single gene|Single	single gene
ESTIMATE.Immune_Nat.Commun.2013|PMID.24113773	median
ESTIMATE.Stromal_Nat.Commun.2013|PMID.24113773	median
Euclidean.Distance.CLOW_BCR.2010|PMID.20813035	special
EXTENDED.Bcell.signature.Garber_Cell.Mol.Gastroenterol.Hepatol.2017|PMID.28508029	median
FGFR4_Single gene|Single	single gene
FGFR4.Induced_JCI.2020|PMID.32573490	median
FGFR4.Repressed_JCI.2020|PMID.32573490	median
Fibrinogen.Cluster_BMC.Med.Genomics.2011|PMID.21214954	median
Fibroblast.Cluster_BMC.Med.Genomics.2011|PMID.21214954	median
Fibroblasts.MCP_Nature.2020|PMID.31942077	median
Fibromatosis_Lab.Invest.2008|PMID.18414401	median
fMaSC.Metab_CellRep.2018|PMID.30089273	median
fMaSC.Metab8_CellRep.2018|PMID.30089273	median
fMaSC.refined1_BCR.2015|PMID.25575446	median

fMasC.Signature_Cell.Stem.Cell.2012|PMID.22305568	median
fMaSC.Signature_CellRep.2018|PMID.30089273	median
FOS.JUN_Cluster_BMC.Med.Genomics.2011|PMID.21214954	median
FOXC1.Hair.Follicles.P30C.LO.vs.WT.Negative_Science.2016|PMID.26912704	median
FOXC1.Hair.Follicles.P30C.LO.vs.WT.Positive_Science.2016|PMID.26912704	median
fSTR.Signature_Cell.Stem.Cell.2012|PMID.22305568	median
Gamma.delta.T.cell_CellRep.2017|PMID.28052254	median
GATA3.induced.genes_JCO.2006|PMID.16505416	median
GATA3.induced.genes_Oncogene.2004|PMID.15361840	median
GDF11.TGFBR3_Nat.Cell.Biol.2014|PMID.24658685	median
Glycolysis_BMC.Med.2009|PMID.19291283	median
GO.DOWN.with.SOX10.OE_Cell.Rep.2015|PMID.26365194	median
GO.UP.with.SOX10.OE_Cell.Rep.2015|PMID.26365194	median
GSEA_BIOCARTA_ALK_PATHWAY|PMID.16199517	median
GSEA_BIOCARTA.AKT.PATHWAY|PMID.16199517	median
GSEA_BIOCARTA.BRCA.ATR.PATHWAY.ATRBRCA|PMID.16199517	median
GSEA_BIOCARTA.CASPASE.PATHWAY|PMID.16199517	median
GSEA_BIOCARTA.CTLA4.PATHWAY|PMID.16199517	median
GSEA_BIOCARTA.IGF1R.PATHWAY|PMID.16199517	median
GSEA_BIOCARTA.MTOR.PATHWAY|PMID.16199517	median
GSEA_BIOCARTA.PTEN.PATHWAY|PMID.16199517	median
GSEA_BIOCARTA.RAS.PATHWAY|PMID.16199517	median
GSEA_BIOCARTA.RB.PATHWAY|PMID.16199517	median
GSEA_BIOCARTA.VEGF.PATHWAY|PMID.16199517	median
GSEA_HALLMARK.MYC.TARGETS.V1|PMID.16199517	median
GSEA_HELLER.HDAC.TARGETS.DOWN|PMID.16199517	median
GSEA_NELSON.RESPONSE.TO.ANDROGEN.UP|PMID.16199517	median
GSEA_REACTOME.PD1.SIGNALING|PMID.16199517	median
GSEA_REACTOME.PI3K.CASCADE|PMID.16199517	median
GSEA_RETINOL.METABOLISM.KEGG|PMID.16199517	median
GSEA.GP1_Proliferation.DNA.repair..PUJANA.CHEK2.PCC.NETWORK|PMID.25109877	median
GSEA.GP1_Proliferation.DNA.repair.REACTOME.CELL.CYCLE.MITOTIC|PMID.25109877	median
GSEA.GP10_Fatty.acid.oxidation.CARBOXYLIC.ACID.METABOLIC.PROCESS|PMID.25109877	median
GSEA.GP11_Immune.IFN.PerouLab|PMID.25109877	median
GSEA.GP12_Hypoxia.glycolosis.SEMENZA.HIF1.TARGETS|PMID.25109877	median
GSEA.GP13_Neural.signailng.MODULE100|PMID.25109877	median
GSEA.GP13_Neural.signailng.NERVOUS.SYSTEM.DEVELOPMENT|PMID.25109877	median
GSEA.GP14_Plasma.membrane.cell.cell.signaling.MORF.CNTN1|PMID.25109877	median

GSEA.GP15_EGF.signaling.NAGASHIMA.EGF.SIGNALING.UP|PMID.25109877	median
GSEA.GP16_Protein.kinase.signaling.MAPKs.INTRACELLULAR.SIGNALING.CASCADE|PMID.25109877	median
GSEA.GP16_Protein.kinase.signaling.MAPKs.REGULATION.OF.KINASE.ACTIVITY|PMID.25109877	median
GSEA.GP17_Basal.signaling.SMID.BREAST.CANCER.BASAL.UP|PMID.25109877	median
GSEA.GP18_Vesicle.EPR.MEMBRANE.COAT|PMID.25109877	median
GSEA.GP19_1Q.amplicon.PerouLab|PMID.25109877	median
GSEA.GP2_Immune.Tcell.Bcell.KEGG.HEMATOPOIETIC.CELL.LINEAGE|PMID.25109877	median
GSEA.GP2_Immune.Tcell.Bcell.PerouLab|PMID.25109877	median
GSEA.GP20_TAL1.Leukemia.erythropoiesis.GNF2.TAL1|PMID.25109877	median
GSEA.GP21_Anti.apoptosis.DNA.stability.MORF.BCL2|PMID.25109877	median
GSEA.GP21_Anti.apoptosis.DNA.stability.MORF.MT4|PMID.25109877	median
GSEA.GP21_Anti.apoptosis.DNA.stability.MORF.STK17A|PMID.25109877	median
GSEA.GP22_16Q22.24.amplicon.PerouLab|PMID.25109877	median
GSEA.GP3_Tumo.suppressing.miRNA.targets.GTTTGTT.MIR.495|PMID.25109877	median
GSEA.GP3_Tumor.suppressing.miRNA.targets.DACOSTA.UV.RESPONSE.VIA.ERCC3.DN|PMID.25109877	median
GSEA.GP3_Tumor.suppressing.miRNA.targets.TGCTTTG.MIR.330|PMID.25109877	median
GSEA.GP4_MES.ECM.PerouLab|PMID.25109877	median
GSEA.GP5_MYC.targets.TERT.PerouLab|PMID.25109877	median
GSEA.GP6_Squamous.differentiation.development.RICKMAN.TUMOR.DIFFERENTIATED.WELL.VS.POORLY.DN|PMID.25109877	median
GSEA.GP7_Estrogen.signaling.SMID.BREAST.CANCER.BASAL.DN|PMID.25109877	median
GSEA.GP8_FOXO.stemness.MORF.PTPRB|PMID.25109877	median
GSEA.GP8_FOXO.stemness.TTGTTT.VSFOXO4.01|PMID.25109877	median
GSEA.GP9_Cell.cell.adhesion.PerouLab|PMID.25109877	median
HCK_BCR.2008|PMID.19272155	median
HER1.Cluster1_BMC.Genomics.2007|PMID.17663798	median
HER1.Cluster2_BMC.Genomics.2007|PMID.17663798	median
HER1.Cluster3_BMC.Genomics.2007|PMID.17663798	median
HER2.Amplicon.PerouLab_BMC.Med.Genomic.2011|PMID.21214954	median
Histological.Grade_J.Pathol.2017|PMID.27861902	median
HouseKeeping_Genome.Biol.2004|PMID.15287981	median
iDC.Median_Immunity.2013|PMID.24138885	median
IFN.Cluster_BMC.Med.Genomics.2011|PMID.21214954	median
IgG_BCR.2008|PMID.19272155	median
IGG.Cluster_BMC.Med.Genomics.2011|PMID.21214954	median
Immature..B.cell_CellRep.2017|PMID.28052254	median
Immature.dendritic.cell_CellRep.2017|PMID.28052254	median
ImmLandscape_Macro.mono.CSF1.core.response_CCR.2009|PMID.29628290	median
ImmLandscape_Wound.Healing_Immunity.2018|PMID.29628290	median

ImmLandscape.IFN3_Plos.One.2014|PMID.24516633	median
ImmLandscape.IFNG5_Plos.One.2014|PMID.24516633	median
ImmLandscape.lymphocyte.Infil.T.B|PMID.18592372	median
Immune.Hot.CD8.vs.Cold_Nature.2020|PMID.31942071	median
Immune.Perez.14_JCO.2015|PMID.25605861	median
Immune.Perez.87_JCO.2015|PMID.25605861	median
Immune.Suppression_JCI.Insight.2016|PMID.27699256	median
ImmuneActive_Cell.2019|PMID.31730857	median
Immunosuppression|PMID.31942077	median
IMS.Score_CCR.2018|PMID.29921729	special
Induced.in.Bcells_PNAS.2013|PMID.23382184	median
Induced.in.DC_PNAS.2013|PMID.23382184	median
Induced.in.GN_PNAS.2013|PMID.23382184	median
Induced.in.HSC_PNAS.2013|PMID.23382184	median
Induced.in.MOs_PNAS.2013|PMID.23382184	median
Induced.in.NKcells_PNAS.2013|PMID.23382184	median
Induced.in.Tcells_PNAS.2013|PMID.23382184	median
Inflammatory.breast.cancer.491genes_CCR.2013|PMID.23396049	median
Inflammatory.breast.cancer.79genes_CCR.2013|PMID.23396049	median
Inflammatory.breast.cancer.expressed.noIBC_79genes_CCR.2013|PMID.23396049	median
Inflammatory.breast.cancer.expressed.noIBC.491genes_CCR.2013|PMID.23396049	median
Influenza.11genes.Metasignature_Immunity.2015|PMID.26682989	median
Interferon_BCR.2008|PMID.19272155	median
Interferon.Pathway_CancerImmunolRes.2018|PMID.30266715	median
JUND.KRT5_Nat.Cell.Biol.2014|PMID.24658685	median
Keller2012.CD10.Adam_BCR.2015|PMID.25575446	median
KRAS.amplicon_Genome.Biology.2007|PMID.17493263	median
Late.IRS.1_PLoS.One.2016|PMID.26991655	median
Late.IRS.2_PLoS.One.2016|PMID.26991655	median
LCK_BCR.2008|PMID.19272155	median
Lim2009.LumProg.Adam_BCR.2015|PMID.25575446	median
Lim2009.MaSC.Adam_BCR.2015|PMID.25575446	median
Lim2009.MatureLum.Adam_BCR.2015|PMID.25575446	median
Lim2009.Stroma.Adam_BCR.2015|PMID.25575446	median
Lim2010.LumProg.Adam_BCR.2015|PMID.25575446	median
Lim2010.MaSC.Adam_BCR.2015|PMID.25575446	median
Lim2010.MatureLum.Adam_BCR.2015|PMID.25575446	median
Lim2010.Stroma.Adam_BCR.2015|PMID.25575446	median

Lobular.Carcinoma.In.Situ_J.Pathol.2017|PMID.27861902	median
LOBULAR.TCGA.SIGNATURE.ImmuneCell.2015|PMID.26451490	median
LOBULAR.TCGA.SIGNATURE.Reactive_Cell.2015|PMID.26451490	median
LOBULAR.TCGA.SUBTYPE.Immune_Cell.2015|PMID.26451490	median
LOBULAR.TCGA.SUBTYPE.Proliferative_Cell.2015|PMID.26451490	median
LOBULAR.TCGA.SUBTYPE.Reactive_Cell.2015|PMID.26451490	median
LTS.score_JCI.2020|PMID.32573490	special
Luminal_Progenitor_Up_Nat.Med.2009|PMID.19648928	median
Luminal.cluster_BMC.Med.Genomics.2011|PMID.21214954	median
Luminal.Progenitor_BCR.2010|PMID.20346151	median
Luminal.Progenitor.Down_Nat.Med.2009|PMID.19648928	median
LumProg.HsEnriched_BCR.2015|PMID.25575446	median
LumProg.HsEnriched.Refined1_BCR.2015|PMID.25575446	median
LumProg.Lim09_BCR.2015|PMID.25575446	median
LumProg.Prat_BCR.2015|PMID.25575446	median
LumProg.Shehata_BCR.2015|PMID.25575446	median
Lums.HER2E.DOWN.metastatic.signature_JCI.2020|PMID.32573490	median
Lums.HER2E.UP.metastatic.signature_JCI.2020|PMID.32573490	median
Lung.WNT_Cancer.Res.2009|PMID.19549913	median
Lymph.vessels_Immunity.2013|PMID.24138885	median
Lymphovascular.Invasion_J.Pathol.2017|PMID.27861902	median
M.D.Metagene_Genome.Biol.2013|PMID.23618380	median
M2.Macrophage_Blood.2006|PMID.16556895	median
Macrophage_CellRep.2017|PMID.28052254	median
Macrophages_CancerImmunolRes.2018|PMID.30266715	median
Macrophages_Immunity.2013|PMID.24138885	median
Macrophages.M0_Nat.Methods.2015|PMID.25822800	median
Macrophages.M1_Nat.Methods.2015|PMID.25822800	median
Macrophages.M2_Nat.Methods.2015|PMID.25822800	median
MacTh1.cluster_CCR.2014|PMID.24916698	median
MammaPrint_Nature.2002|PMID.11823860	special
MAPK.pathway.activation_NPJ.Precis.Oncol.2018|PMID.29872725	median
MASC.Down_Nat.Med.2009|PMID.19648928	median
MASC.Up_Nat.Med.2009|PMID.19648928	median
Mast.cell_CellRep.2017|PMID.28052254	median
Mast.cells_Immunity.2013|PMID.24138885	median
Mast.cells.activated_Nat.Methods.2015|PMID.25822800	median
Mast.cells.resting_Nat.Methods.2015|PMID.25822800	median

Mature.luminal_BCR.2010|PMID.20346151	median
Mature.Luminal.Down_Nat.Med.2009|PMID.19648928	median
Mature.LuminaUp_Nat.Med.2009|PMID.19648928	median
MatureLum.HsEnriched_BCR.2015|PMID.25575446	median
MatureLum.HsEnriched.Refined1_BCR.2015|PMID.25575446	median
MatureLum.Lim09_BCR.2015|PMID.25575446	median
MatureLum.Prat_BCR.2015|PMID.25575446	median
MatureLum.Shehata_BCR.2015|PMID.25575446	median
MBasal.Cluster_BMC.Med.Genomics.2011|PMID.21214954	median
MCD3.CD8_BMC.Med.Genomics.2011|PMID.21214954	median
MCF7.E2.induced.genes_JCO.2006|PMID.16505416	median
MCF7.E2.repressed.genes_JCO.2006|PMID.16505416	median
MDSC_CellRep.2017|PMID.28052254	median
MDSC.Granulocytic_Leukoc.Biol.2012|PMID.21954284	median
MDSC.Neutrophi_Leukoc.Biol.2012|PMID.21954284	median
MDSC.tumor_J.Immunol.2012|PMID.23152559	median
MDSC.tumor.MO_J.Immunol.2012|PMID.23152559	median
MECM_BMC.Med.Genomics.2011|PMID.21214954	median
Memory.B.cell_CellRep.2017|PMID.28052254	median
MET.DOWN.RNAseq.Significant.Genes_JCI.2018|PMID.29480819	median
MET.DOWN.Significant.Genes.Low.Basal.1_JCI.2018|PMID.29480819	median
MET.DOWN.Significant.Genes.Low.Basal.2_JCI.2018|PMID.29480819	median
MET.UP.RNAseq.Significant.Genes_JCI.2018|PMID.29480819	median
MET.UP.Significant.Genes.HIGH.BASALS.Genes_JCI.2018|PMID.29480819	median
Metaplastic.Up_CanRes.2009|PMID.19435916	median
Metastasis.predictor.TNBC_BCR.2010|PMID.20946665	median
MFGFR2_BMC.Med.Genomics.2011|PMID.21214954	median
MHC.Forero.11_Cancer.Immunol.Res.2016|PMID.26980599	median
MHC.Forero.24_Cancer.Immunol.Res.2016|PMID.26980599	median
MHC.I_BCR.2008|PMID.19272155	median
MHC.II_BCR.2008|PMID.19272155	median
MHCI.coreGenes_Nat.Commun.2017|PMID29170503	median
MIR200c.Induced_ONCO.2015|PMID.25746005	median
MIR200c.Repressed_ONCO.2015|PMID.25746005	median
miRNA.138.signature_Cancer.Res.2014|PMID.25339353	median
MITO1_BMC.Med.Genomics.2011|PMID.21214954	median
MITO2_BMC.Med.Genomics.2011|PMID.21214954	median
Mitotic.Count_J.Pathol.2017|PMID.27861902	median

MK14.K17_BMC.Med.Genomics.2011|PMID.21214954	median
MKRAS.amplicon_BMC.Med.Genomics.2011|PMID.21214954	median
MM.BRCAwnt.1pFDR.UP_Genome.Biology.2007|PMID.17493263	median
MM.C3Tag.1pFDR.UP_Genome.Biology.2007|PMID.17493263	median
MM.C3Tag.2012_Genome.Biol.2013|PMID.24220145	median
MM.Class3_Genome.Biol.2013|PMID.24220145	median
MM.Class8_Genome.Biol.2013|PMID.24220145	median
MM.Claudinlow_Genome.Biol.2013|PMID.24220145	median
MM.DMBAwnt.1pFDR.UP.Genome.Biology.2007|PMID.17493263	median
MM.Erbb2.like_Genome.Biol.2013|PMID.24220145	median
MM.Myc.2012_Genome.Biol.2013|PMID.24220145	median
MM.Myoepithelioma.like_Genome.Biol.2013|PMID.24220145	median
MM.Neu.2012_Genome.Biol.2013|PMID.24220145	median
MM.NeuPyMT.1pFDR.UP_Genome.Biology.2007|PMID.17493263	median
MM.Normal.1pFDR.UP_Genome.Biology.2007|PMID.17493263	median
MM.Normal.like_Genome.Biol.2013|PMID.24220145	median
MM.p53null.1pFDR.UP_Genome.Biology.2007|PMID.17493263	median
MM.p53null.Basal_Genome.Biol.2013|PMID.24220145	median
MM.p53null.Luminal_Genome.Biol.2013|PMID.24220145	median
MM.Potluck_1pFDR_UP_Genome.Biology.2007_PMID.17493263|PMID.24220145	median
MM.PyMT.2012_Genome.Biol.2013|PMID.24220145	median
MM.Squamous.like_Genome.Biol.2013|PMID.24220145	median
MM.Stat1_Genome.Biol.2013|PMID.24220145	median
MM.WapINT3.1pFDR.UP_Genome.Biology.2007|PMID.17493263	median
MM.WapINT3.2012_Genome.Biol.2013|PMID.24220145	median
MM.WAPTag.1pFDR.UP_Genome.Biology.2007|PMID.17493263	median
MM.Wnt1.Early_Genome.Biol.2013|PMID.24220145	median
MM.Wnt1.Late_Genome.Biol.2013|PMID.24220145	median
Mmyosin_BMC.Med.Genomics.2011|PMID.21214954	median
MNADH_CYTochrome_BMC.Med.Genomics.2011|PMID.21214954	median
MNB1_BMC.Med.Genomics.2011|PMID.21214954	median
MNB2_BMC.Med.Genomics.2011|PMID.21214954	median
MNB3_BMC.Med.Genomics.2011|PMID.21214954	median
MNOtch4_BMC.Med.Genomics.2011|PMID.21214954	median
Monocyte_CellRep.2017|PMID.28052254	median
Monocyte..DC.25gene_Genome.Biol.2013|PMID.23618380	median
Monocytes_CancerImmunolRes.2018|PMID.30266715	median
Monocytes_Nat.Methods.2015|PMID.25822800	median

Monocytic.lineage.MCP_Nature.2020|PMID.31942075	median
MProliferation_BMC.Med.Genomics.2011|PMID.21214954	median
MProtocadherin_BMC.Med.Genomics.2011|PMID.21214954	median
MPYMT_NEU_Cluster_BMC.Med.Genomics.2011|PMID.21214954	median
MRibosomal_BMC.Med.Genomics.2011|PMID.21214954	median
MS.CD44.DOWN_PNAS.2009|PMID.19666588	median
MS.CD44.UP_PNAS.2009|PMID.19666588	median
MSquamous_BMC.Med.Genomics.2011|PMID.21214954	median
Murat.G07_JCO.2008|PMID.18565887	median
Murat.G18_JCO.2008|PMID.18565887	median
Murat.G24_JCO.2008|PMID.18565887	median
MVEGFC_BMC.Med.Genomics.2011|PMID.21214954	median
Myeloid.cell.chemotaxis.1gene_Nature.2020|PMID.31942077	median
Myeloid.dendritic.cells.MCP_Nature.2020|PMID.31942077	median
Natural.killer.cell_CellRep.2017|PMID.28052254	median
Natural.killer.T.cell_CellRep.2017|PMID.28052254	median
Necrosis_J.Pathol.2017|PMID.27861902	median
Neutrophil_CellRep.2017|PMID.28052254	median
Neutrophils_CancerImmunolRes.2018|PMID.30266715	median
Neutrophils_Immunity.2013|PMID.24138885	median
Neutrophils_Nat.Methods.2015|PMID.25822800	median
Neutrophils.MCP_Nature.2020|PMID.31942077	median
NK_Immunity.2013|PMID.24138885	median
NK.activated_Nat.Methods.2015|PMID.25822800	median
NK.CD56bright_Immunity.2013|PMID.24138885	median
NK.CD56dim_Immunity.2013|PMID.24138885	median
NK.resting_Nat.Methods.2015|PMID.25822800	median
NKcells_CancerImmunolRes.2018|PMID.30266715	median
NKcells.MCP_Nature.2020|PMID.31942077	median
No.Response.Immunotherapy.TLS.Melanoma_Nature.2020|PMID.31942075	median
Normal.mucosa_Immunity.2013|PMID.24138885	median
Nuclear.Pleomorphism_J.Pathol.2017|PMID.27861902	median
Oncotype_NEJM.2004|PMID.15591335	special
P53.ERPos.MDACC_CCR.2011|PMID.21248301	median
Parity.signature.251genes_BCR.2014|PMID.25005139	median
Parity.signature.40genes_BCR.2014|PMID.25005139	median
PARPi.Resistance_BCRT_2012|PMID.22875744	median
PARPi.Sensitivity_BCRT_2012|PMID.22875744	median

PARPi.Sensitivity.MDACC_NPJ.Syst.Biol.Appl.2017|PMID.28649435	median
PARPi.Sensitivity.Negative_Sci.Adv.2017|PMID.28439535	median
PARPi.Sensitivity.Positive_Sci.Adv.2017|PMID.28439535	median
Pcorr.Breast2Lung.LM2.Correlation_Nature.2005|PMID.16049480	special
Pcorr.Breast2Lung.Parental.Correlation_Nature.2005|PMID.16049480	special
Pcorr.dasatinib.resistant_Cancer.Res.2007|PMID.17332353	special
Pcorr.dasatinib.sensitive_Cancer.Res.2007|PMID.17332353	special
Pcorr.Hypoxia.High.Correlation_PLoS.Med.2006|PMID.16417408	special
Pcorr.Hypoxia.Low.Correlation_PLoS.Med.2006|PMID.16417408	special
Pcorr.IGS_Invasiveness_NJEM.2007|PMID.17229949	special
Pcorr.wound.response.activated_PNAS.2005|PMID.15701700	special
pCR.predictor.ERNeg.55genes_JAMA.2011|PMID.21558518	median
pCR.predictor.ERPos.39genes_JAMA.2011|PMID.21558518	median
PDCD1_Single gene|Single	single gene
Pfefferle2012.LumProg_BCR.2015|PMID.25575446	median
Pfefferle2012.MaSC_BCR.2015|PMID.25575446	median
Pfefferle2012.MatureLum_BCR.2015|PMID.25575446	median
Pfefferle2012.Stroma_BCR.2015|PMID.25575446	median
PGR_Single gene|Single	single gene
PI3Ki.Down_CancerCell.2017|PMID.28528867	median
PI3Ki.Up_CancerCell.2017|PMID.28528867	median
PIK3CA.Pathway_Ann.Oncol.2017|PMID.28177460	median
PIK3CAmt.signature_Cancer.Res.2012|PMID.22552288	median
Plasma.cells_Nat.Methods.2015|PMID.25822800	median
PlasmaCells_CancerImmunolRes.2018|PMID.30266715	median
Plasmacytoid.dendritic.cell_CellRep.2017|PMID.28052254	median
PR.Isoform.Ratio.Up.in.PRA.H_JNCI.2017|PMID.28376177	median
PR.Isoform.Ratio.Up.in.PRB.H_JNCI.2017|PMID.28376177	median
Proliferation.Cluster_BMC.Med.Genomics.2011|PMID.21214954	median
Proliferation.Metagene_Genome.Biol.2013|PMID.23618380	median
Proliferation.score.PAM50_JCO.2009|PMID.19204204	special
ProliferationPathway_CancerImmunolRes.2018|PMID.30266715	median
Prosigna.Proliferation.18_BMC.Med.Genomics.2015|PMID.26297356	median
Race.LuminalA.MRE.score_BCRT.2015|PMID.26109344	special
Radiation.induced.genes_Radoat.Res.2014|PMID.24527691	median
RB.LOH_BCR.2008|PMID.18782450	median
RB.LOSS_JCI.2007|PMID.17160137	median
Regulatory.T.cell_CellRep.2017|PMID.28052254	median

Replication.Stress.Down.set_Cell.Rep.2018|PMID.29768207	median
Replication.Stress.Model_Cell.Rep.2018_PMID.29768207|PMID.29768207	special
Replication.Stress.Neg_Cell.Rep.2018_PMID.29768207|PMID.29768207	median
Replication.Stress.Pos_Cell.Rep.2018_PMID.29768207|PMID.29768207	median
Replication.Stress.Up_Set_Cell.Rep.2018_PMID.29768207|PMID.29768207	median
Residual.disease.predictor.ERNeg.54genes_JAMA.2011|PMID.21558518	median
Residual.disease.predictor.ERPos.73genes_JAMA.2011|PMID.21558518	median
Response.Immunotherapy.MCP.TLS.Melanoma_Nature.2020|PMID.31942075	median
Response.Immunotherapy.signature_Science.2018|PMID.30309915	median
Response.Neo.Chemo_common_CCR.2014|PMID.25047707	median
Response.Neo.Chemo_ERNeg_CCR.2014|PMID.25047707	median
Response.Neo.Chemo_ERPos_CCR.2014|PMID.25047707	median
RHOA.pathway_Ann.Oncol.2017|PMID.28177460	median
Ribosomal.Cluster_BMC.Med.Genomics.2011|PMID.21214954	median
ROR.subtype.PAM50_JCO.2009|PMID.19204204	special
ROR.subtype.proliferation.PAM50_JCO.2009|PMID.19204204	special
RSS.Score_CCR.2018|PMID.29921729	special
S100A9.A8_BMC.Med.Genomics.2011|PMID.21214954	median
Scorr.EMAT1.Correlation_BCR.2020|PMID.32641077	special
Scorr.EMAT2.Correlation_BCR.2020|PMID.32641077	special
Scorr.EMAT3.Correlation_BCR.2020|PMID.32641077	special
Scorr.EMAT4.Correlation_BCR.2020|PMID.32641077	special
Scorr.IE.Correlation_JCO.2006|PMID.16505416	special
Scorr.IIE.Correlation_JCO.2006|PMID.16505416	special
Scorr.PAM50.Basal_JCO.2009|PMID.19204204	special
Scorr.PAM50.Her2_JCO.2009|PMID.19204204	special
Scorr.PAM50.LumA_JCO.2009|PMID.19204204	special
Scorr.PAM50.LumB_JCO.2009|PMID.19204204	special
Scorr.PAM50.Normal_JCO.2009|PMID.19204204	special
Scorr.S329.L_Br.J.Cancer.2008|PMID.18382427	special
Scorr.S329.R_Br.J.Cancer.2008|PMID.18382427	median
Secretoglobin_BMC.Med.Genomics.2011|PMID.21214954	median
Shehata2012.ALDHneg_BCR.2015|PMID.25575446	median
Shehata2012.ALDHpos_BCR.2015|PMID.25575446	median
Shehata2012.Basal_BCR.2015|PMID.25575446	median
Shehata2012.Erbb3neg_BCR.2015|PMID.25575446	median
Shehata2012.LumProg_BCR.2015|PMID.25575446	median
Shehata2012.NCL_BCR.2015|PMID.25575446	median

Shehata2012.Stroma_BCR.2015|PMID.25575446	median
Spike2012.aMaSC_BCR.2015|PMID.25575446	median
Spike2012.fMaSC_BCR.2015|PMID.25575446	median
Spike2012.fStr_BCR.2015|PMID.25575446	median
STAT1_BCR.2008|PMID.19272155	median
STAT3.Basal_PNAS.2014|PMID.25139989	median
STAT3.Basal.short_PNAS.2014|PMID.25139989	median
Stroma.FNA.MDACC.1_JCO.2010|PMID.20805453	median
Stroma.FNA.MDACC.2_JCO.2010|PMID.20805453	median
Stromal.Central.Fibrotic.Focus_J.Pathol.2017|PMID.27861902	median
Stromal.Down_Nat.Med.2009|PMID.19648928	median
Stromal.Inflammation_J.Pathol.2017|PMID.27861902	median
Stromal.Signature_Nat.Med.2008|PMID.18438415	median
Stromal.Up_Nat.Med.2009|PMID.19648928	median
SW480.cancer.cells_Immunity.2013|PMID.24138885	median
T.follicular.helper.cell_CellRep.2017|PMID.28052254	median
Tcell.activation_Nature.2020|PMID.31942077	median
Tcell.CD8.Effector.vs.naive.2_Science.2016|PMID27789795	median
Tcell.CD8.Exhausted.vs.antiPDL1.2_Science.2016|PMID27789795	median
Tcell.CD8.Exhausted.vs.naive.2_Science.2016|PMID27789795	median
Tcell.CD8.Memory.vs.naive.1_Science.2016|PMID27789795	median
Tcell.cluster_CCR.2014|PMID.24916698	median
Tcell.EXH.Anti.PDL1.vs.control.treated.exhausted.CD8.Tcell.Metagene.1.Science.2016|PMID.27789795	median
Tcell.EXH.Effector.CD8.T.cell.at.day.8.p.i.Armstrong.vs.Naive.CD8.Tcell.Metagene.1_Science.2016|PMID.27789795	median
Tcell.EXH.Exhausted.CD8.T.cell.vs.Naive.CD8.T.cell.Metagene.1_Science.2016|PMID.27789795	median
Tcell.EXH.Exhausted.CD8.T.cell.vs.Naive.CD8.T.cell.Metagene.3_Science.2016|PMID.27789795	median
Tcell.EXH.Memory.CD8.T.cell.a.vs.Naive.CD8.T.cell.Metagene.1_Science.2016|PMID.27789795	median
Tcell.EXH.Memory.CD8.T.cell.a.vs.Naive.CD8.T.cell.Metagene.2_Science.2016|PMID.27789795	median
Tcell.EXH.Memory.CD8.T.cell.a.vs.Naive.CD8.T.cell.Metagene.3.Science_2016|PMID.27789795	median
Tcell.NK.51gene_Genome.Biol.2013|PMID.23618380	median
Tcell.NK.Metagene_Genome.Biol.2013|PMID.23618380	median
Tcell.RM_Nat_Med.2018|PMID.29942092	median
Tcell.survival.2gene_Nature.2020|PMID.31942077	median
Tcells_CancerImmunolRes.2018|PMID.30266715	median
Tcells_Immunity.2013|PMID.24138885	median
Tcells_TFH_Nat.Methods.2015|PMID.25822800	median
Tcells.CD4.memory.activated_Nat.Methods.2015|PMID.25822800	median
Tcells.CD4.memory.resting_Nat.Methods.2015|PMID.25822800	median

Tcells.CD4.naive_Nat.Methods.2015|PMID.25822800	median
Tcells.CD8_Immunity.2013|PMID.24138885	median
Tcells.CD8_Nat.Methods.2015|PMID.25822800	median
Tcells.CD8.MCP_Nature.2020|PMID.31942075	median
Tcells.Cytotoxic.MCP_Nature.2020|PMID.31942075	median
Tcells.gammadelta_Nat.Methods.2015|PMID.25822800	median
Tcells.helper_Immunity.2013|PMID.24138885	median
Tcells.MCP_Nature.2020|PMID.31942077	median
Tcells.regulatory.2gene_Nature.2020|PMID.31942077	median
Tcells.Tregs_Nat.Methods.2015|PMID.25822800	median
TCGA.BRCA.1198_BASAL_JCI.2020|PMID.32573490	median
TCGA.BRCA.1198_Chromogranin_JCI.2020|PMID.32573490	median
TCGA.BRCA.1198_COLLAGEN11A_JCI.2020|PMID.32573490	median
TCGA.BRCA.1198_EN1_FDZ9_JCI.2020|PMID.32573490	median
TCGA.BRCA.1198_FGFR4_EGF_JCI.2020|PMID.32573490	median
TCGA.BRCA.1198_HISTONES_JCI.2020|PMID.32573490	median
TCGA.BRCA.1198_HOXC11_HOTAIR_SIX1_JCI.2020|PMID.32573490	median
TCGA.BRCA.1198_IL8_CCL_JCI.2020|PMID.32573490	median
TCGA.BRCA.1198_immune_CD19_JCI.2020|PMID.32573490	median
TCGA.BRCA.1198_immune_CD34_TIE1_JCI.2020|PMID.32573490	median
TCGA.BRCA.1198_immune_CD4_CD53_CD84_BTK_JCI.2020|PMID.32573490	median
TCGA.BRCA.1198_immune_CD8_GZMK_JCI.2020|PMID.32573490	median
TCGA.BRCA.1198_immune_CTLA4_CXCL_FOXP3_JCI.2020|PMID.32573490	median
TCGA.BRCA.1198_immune_FOS_JUN_IL6_JCI.2020|PMID.32573490	median
TCGA.BRCA.1198_immune_GIMAP_IL16_JCI.2020|PMID.32573490	median
TCGA.BRCA.1198_immune_HLA_A_F_JCI.2020|PMID.32573490	median
TCGA.BRCA.1198_immune_HLA_D_JCI.2020|PMID.32573490	median
TCGA.BRCA.1198_immune_INTERFERON_JCI.2020|PMID.32573490	median
TCGA.BRCA.1198_IMMUNE1_JCI.2020|PMID.32573490	median
TCGA.BRCA.1198_LUMINAL_JCI.2020|PMID.32573490	median
TCGA.BRCA.1198_MYBL2_APOBEC3B_JCI.2020|PMID.32573490	median
TCGA.BRCA.1198_NORMAL_JCI.2020|PMID.32573490	median
TCGA.BRCA.1198_NORMAL2_JCI.2020|PMID.32573490	median
TCGA.BRCA.1198_PDCHA_MANY_JCI.2020|PMID.32573490	median
TCGA.BRCA.1198_S100A7_8_9_JCI.2020|PMID.32573490	median
TCGA.BRCA.1198_TP63_JCI.2020|PMID.32573490	median
TCGA.BRCA.1198.IMMUNOGLOBULIN_JCI.2020|PMID.32573490	median
TCGA.CSF1.response_Immunity.2018|PMID.29628290	median

TCGA.IFN.score_Immunity.2018|PMID.29628290	median
TCGA.Liexpression.score_Immunity.2018|PMID.29628290	median
TCGA.Serum.response.up_Immunity.2018|PMID.29628290	median
TCGA.TFH_Immunity.2018|PMID.29628290	median
TCGA.Tgd_Immunity.2018|PMID.29628290	median
TCGA.TGFB.score_Immunity.2018|PMID.29628290	median
Tcm_Immunity.2013|PMID.24138885	median
Tem_Immunity.2013|PMID.24138885	median
TFH_Immunity.2013|PMID.24138885	median
Tgd_Immunity.2013|PMID.24138885	median
Th1_cells_Immunity.2013|PMID.24138885	median
Th17_cells_Immunity.2013|PMID.24138885	median
Th2_cells_Immunity.2013|PMID.24138885	median
TLS.9Gene.Signature_Nature.2020|PMID.31942071	median
TLS.CXCL13.Single.Gene_Nature.2020|PMID.31942077	median
TLS.Hallmark.Gene.Signature_Nature.2020|PMID.31942071	median
TLS.Known.Markers_Nature.2020|PMID.31942071	median
TLS.Structure.12chemokine_FrontImmunol.2017|PMID.28713385	median
TLS.tumors.wTLS.and.CD8.vs.CD8alone_Nature.2020|PMID.31942071	median
TNBC.good.prognosis.TNBC.230genes_BCR.2011|PMID.21978456	median
TNBC.good.prognosis.TNBC.26genes_BCR.2011|PMID.21978456	median
TNBC.metastasis.free.survival_PLoS.One.2013|PMID.24349199	median
TNBC.poor.prognosis.TNBC.26genes_BCR.2011|PMID.21978456	median
Translation.Pathway_CancerImmunolRes.2018|PMID.30266715	median
Tumour.hypoxia.causes.DNA.hypermethylation_Nature.2016|PMID.27533040	median
Type.1.T.helper.cell_CellRep.2017|PMID.28052254	median
Type.17.T.helper.cell_CellRep.2017|PMID.28052254	median
Type.2.T.helper.cell_CellRep.2017|PMID.28052254	median
Up.Basal.High_Nat.Cell.Biol.2014|PMID.25173976	median
Up.Proliferation_Nat.Cell.Biol.2014|PMID.25173976	median
Upregulated.by.oncogenic.NRAS.basal_Cell.Rep.2016|PMID.26166574	median
Upregulated.upon.NRAS.repression.basal_Cell.Rep.2017|PMID.26166574	median
Vascular.Content_Clin.Exp.Metastasis.2014|PMID.23975155	median
VEGF.13genes_BMC.Med.2009|PMID.19291283	median
Wirapati.Proliferation_BCR.2008|PMID.18662380	median
Wound.Signature_CCR.2009|PMID.19887484	median
X11q13.Amplicon_BMC.Med.Genomics.2011|PMID.21214954	median
X12qMDM4.BMC.Med.Genomics.2011|PMID.21214954	median

X13q14.Amplicon_BMC.Med.Genomics.2011|PMID.21214954	median
X15q25.Amplicon_BMC.Med.Genomics.2011|PMID.21214954	median
X16.13.Amplicon_BMC.Med.Genomics.2011|PMID.21214954	median
X16q23.Amplicon_BMC.Med.Genomics.2011|PMID.21214954	median
X17PP13.Amplicon_BMC.Med.Genomics.2011|PMID.21214954	median
X17q25x.BMC.Med.Genomics.2011|PMID.21214954	median
X19p13.Amplicon_BMC.Med.Genomics.2011|PMID.21214954	median
X1p36.Amplicon_BMC.Med.Genomics.2011|PMID.21214954	median
X3p21.Amplicon_BMC.Med.Genomics.2011|PMID.21214954	median
X4p16.Amplicon_BMC.Med.Genomics.2011|PMID.21214954	median
X5Q_BCRT.2012|PMID.22048815	median
X8p.Amplicon_BMC.Med.Genomics.2011|PMID.21214954	median
X8p22.Amplicon_BMC.Med.Genomics.2011|PMID.21214954	median
XBP1.Signature_Nature.2014|PMID.24670641	median

eTable 2
Comparison of baseline clinico-pathologic characteristics of the patients from NSABP B-41, CALGB 40601, and NeoALTTO in the intention-to-treat (ITT) cohort (N = 1,298)
IQR: interquartile range; HR: hormone receptor; N: lymph node involvement; H: trastuzumab; L: lapatinib.

Variable	C40601, *N* = 305	NeoALTTO, *N* = 455	B41, *N* = 529
**Age** Median (IQR)	49 (42, 56)	50 (45, 55)	49 (42, 56)
**Menopause status**			
Postmenopausal	123 (40.3%)	222 (48.8%)	232 (43.9%)
Premenopausal	182 (59.7%)	233 (51.2%)	297 (56.1%)
**Race**			
Asian	17 (5.6%)	108 (23.7%)	22 (4.2%)
Black	27 (8.9%)	8 (1.8%)	42 (7.9%)
Other	18 (5.9%)	43 (9.5%)	13 (2.5%)
White	243 (79.7%)	296 (65.1%)	452 (85.4%)
**HR status**			
HR negative	127 (41.6%)	223 (49.0%)	198 (37.4%)
HR positive	178 (58.4%)	232 (51.0%)	331 (62.6%)
**Clinical tumor size**			
T1-T2	204 (66.9%)	274 (60.2%)	362 (68.4%)
T3-T4	75 (24.6%)	181 (39.8%)	167 (31.6%)
Unknown	26 (8.5%)	0 (0.0%)	0 (0.0%)
**Clinical status of lymph nodes**			
N positive	158 (51.8%)	326 (71.6%)	268 (50.7%)
N negative	131 (43.0%)	123 (27.0%)	261 (49.3%)
Unknown	16 (5.2%)	6 (1.3%)	0 (0.0%)
**Treatment arm**			
H	120 (39.3%)	149 (32.7%)	181 (34.2%)
HL	118 (38.7%)	152 (33.4%)	174 (32.9%)
L	67 (22.0%)	154 (33.8%)	174 (32.9%)

[1]Kruskal-Wallis rank sum test; Pearson's Chi-squared test; Fisher's exact test

eTable 3
Association of treatment arm with pathologic complete response (pCR) in the intention-to-treat (ITT) cohort.
HR: hormone receptor; N: lymph node involvement; H: trastuzumab; L: lapatinib; OR: odds ratio; CI: confidence interval.

Characteristic	OR[1]	95% CI[1]	*P*
**Study**			
C40601	—	—	
B-41	1.52	1.12, 2.06	***0.007***
NEOALTTO	0.62	0.45, 0.86	***0.004***
**Treatment arm**			
H	—	—	
HL	1.8	1.36, 2.39	***<0.001***
L	0.79	0.59, 1.05	*0.1*
**HR status**			
HR negative	—	—	
HR positive	0.48	0.37, 0.60	***<0.001***
**Clinical tumor size**			
T1-T2	—	—	
T3-T4	0.81	0.63, 1.04	*0.09*
**Clinical status of lymph nodes**			
N negative	—	—	
N positive	0.89	0.70, 1.14	*0.37*

[1]OR = Odds Ratio, CI = Confidence Interval

eTable 4

Association of treatment arm with event-free survival (EFS) in the intention-to-treat (ITT) cohort.

The Cox model has been stratified by clinical trial

HR: hormone receptor; N: lymph node involvement; H: trastuzumab; L: lapatinib;

HR: hazard ratio; CI: confidence interval.

Characteristic	HR[1]	95% CI[1]	*P*
**Treatment arm**			
H	—	—	
HL	0.74	0.54, 1.01	*0.06*
L	1.22	0.92, 1.62	*0.16*
**HR status**			
HR negative	—	—	
HR positive	0.81	0.64, 1.04	*0.09*
**Clinical tumor size**			
T1-T2	—	—	
T3-T4	1.44	1.12, 1.84	***0.004***
**Clinical status of lymph nodes**			
N negative	—	—	
N positive	1.87	1.42, 2.48	***<0.001***

[1]HR = Hazard Ratio, CI = Confidence Interval

eTable 5
Comparison of baseline clinicopathologic characteristics of the patients from the intention-to-treat (ITT) and the RNA sequencing (RNAseq) cohorts.
HR: hormone receptor; N: lymph node involvement; H: trastuzumab; L: lapatinib.

Variable	ITT, *N* = 1,289	RNAseq, *N* = 758	*P*[1]
**Study**			**<0.001**
B-41	529 (41.0%)	245 (32.3%)	
C40601	305 (23.7%)	264 (34.8%)	
NeoALTTO	455 (35.3%)	249 (32.8%)	
**Age** Median (IQR)	50 (42, 56)	49 (41, 55)	0.43
**Menopause status**			0.55
Postmenopausal	577 (44.8%)	329 (43.4%)	
Premenopausal	712 (55.2%)	429 (56.6%)	
**Race**			0.87
Asian	147 (11.4%)	94 (12.4%)	
Black	77 (6.0%)	49 (6.5%)	
Other	74 (5.7%)	43 (5.7%)	
White	991 (76.9%)	572 (75.5%)	
**HR status**			0.65
HR-negative	548 (42.5%)	330 (43.5%)	
HR-positive	741 (57.5%)	428 (56.5%)	
**Clinical tumor size**			0.4
T1-T2	840 (65.2%)	497 (65.6%)	
T3-T4	423 (32.8%)	239 (31.5%)	
Unknown	26 (2.0%)	22 (2.9%)	
**Clinical status of lymph nodes**			0.76
N positive	752 (58.3%)	446 (58.8%)	
N negative	515 (40.0%)	296 (39.1%)	
Unknown	22 (1.7%)	16 (2.1%)	
**Treatment arm**			0.98
H	450 (34.9%)	267 (35.2%)	
HL	444 (34.4%)	262 (34.6%)	
L	395 (30.6%)	229 (30.2%)	
**Global pCR rates**			0.9
pCR	588 (46.3%)	349 (46.0%)	
RD	681 (53.7%)	409 (54.0%)	
**5-year rate of EFS**	79%	81%	0.35

[1]Pearson's Chi-squared test; Wilcoxon rank sum test; Fisher's exact test; Log Rank test

eTable 6

Distribution of tumor intrinsic subtype by hormone receptor (HR) status and study.

HR: hormone receptor; HER2-E: HER2-Enriched; LumA: Luminal A; LumB: Luminal B.

All RNAseq, *N* = 758					*P*[1] Distribution of subtypes by study
**Subtype**	HR positive, *N* = 428	HR negative, *N* = 330	*P*[1] Distribution of subtypes by HR status	Total, *N* = 758	0.11
			**<0.001**		
Basal	12 (2.8%)	55 (16.7%)		67 (8.8%)	
HER2-E	186 (43.5%)	253 (76.7%)		439 (57.9%)	
LumA	73 (17.1%)	2 (0.6%)		75 (9.9%)	
LumB	112 (26.2%)	2 (0.6%)		114 (15.0%)	
Normal	45 (10.5%)	18 (5.5%)		63 (8.3%)	
C40601, *N* = 264					
**Subtype**	HR positive, *N* = 153	HR negative, *N* = 111	*P*[1] Distribution of subtypes by HR status	Total, *N* = 264	
			**<0.001**		
Basal	2 (1.3%)	20 (18.0%)		22 (8.3%)	
HER2-E	66 (43.1%)	80 (72.1%)		146 (55.3%)	
LumA	28 (18.3%)	0 (0.0%)		28 (10.6%)	
LumB	35 (22.9%)	0 (0.0%)		35 (13.3%)	
Normal	22 (14.4%)	11 (9.9%)		33 (12.5%)	
NeoALTTO, *N* = 249					
**Subtype**	HR positive, *N* = 134	HR negative, *N* = 115	*P*[1] Distribution of subtypes by HR status	Total, *N* = 249	
			**<0.001**		
Basal	5 (3.7%)	15 (13.0%)		20 (8.0%)	
HER2-E	57 (42.5%)	92 (80.0%)		149 (59.8%)	
LumA	19 (14.2%)	2 (1.7%)		21 (8.4%)	
LumB	38 (28.4%)	2 (1.7%)		40 (16.1%)	
Normal	15 (11.2%)	4 (3.5%)		19 (7.6%)	
B-41, *N* = 245					
**Subtype**	HR positive, *N* = 141	HR negative, *N* = 104	*P*[1] Distribution of subtypes by HR status	Total, *N* = 245	
			**<0.001**		
Basal	5 (3.5%)	20 (19.2%)		25 (10.2%)	
HER2-E	63 (44.7%)	81 (77.9%)		144 (58.8%)	
LumA	26 (18.4%)	0 (0.0%)		26 (10.6%)	
LumB	39 (27.7%)	0 (0.0%)		39 (15.9%)	
Normal	8 (5.7%)	3 (2.9%)		11 (4.5%)	
1Pearson's Chi-squared test					

eTable 7

Association of pCR with EFS by tumor intrinsic subtype in multivariable Cox analysis stratified by clinical trials and adjusted by treatment arm. Result from a Landmark analysis at 30 weeks

Cox models has been stratified by clinical trial and adjusted by treatment arm

HR: Hazard ratio; H: trastuzumab; L: lapatinib; pCR: pathologic complete response; RD: residual disease; CI: confident interval.

Luminal A			
Variables	HR[1]	95% CI[1]	*P*
Treatment arm			
H	—	—	
HL	5.12	0.96, 27.3	0.06
L	1.96	0.35, 11.1	0.45
pCR status			
RD	—	—	
pCR	0.26	0.03, 2.24	0.22

[1]HR = Hazard Ratio, CI = Confidence Interval

Luminal B			
Variables	HR[1]	95% CI[1]	*P*
Treatment arm			
H	—	—	
HL	1.21	0.42, 3.50	0.72
L	1.24	0.43, 3.58	0.69
pCR status			
RD	—	—	
pCR	0.89	0.32, 2.48	0.83

[1]HR = Hazard Ratio, CI = Confidence Interval

HER2-Enriched			
Variables	HR[1]	95% CI[1]	*P*
Treatment arm			
H	—	—	
HL	0.55	0.31, 0.96	0.04
L	1.15	0.70, 1.89	0.58
pCR status			
RD	—	—	
pCR	0.46	0.29, 0.73	**<0.001**

[1]HR = Hazard Ratio, CI = Confidence Interval

Basal-like			
Variables	HR[1]	95% CI[1]	*P*
Treatment arm			
H	—	—	
HL	0.87	0.21, 3.57	0.85
L	0.54	0.13, 2.28	0.4
pCR status			
RD	—	—	
pCR	0.21	0.05, 0.97	**0.045**

[1]HR = Hazard Ratio, CI = Confidence Interval

eTable 8

Association of gene expression signatures at baseline with pCR in the combined cohort, CALGB 40601, NeoALTTO, and NSABP B-41.

Logistic regression multivariable models adjusted by treatment arm, stage, node status,
HR status and study (combined cohort) have been built for each gene expression biomarker.
Adjusted p-values for multiple testing using a Benjamini & Hochberg method to control the False Discovery Rate are provided
OR: odds ratio; CI: confident interval.
The biomarkers significantly associated with pCR in the combined cohort are higlighted in blue.
The biomarkers significantly associated with pCR in the three cohorts are highlighted in green.

Combined cohort					
Signature	OR	95% CI		*P*	adjusted *P*
Activate.Endothelium_Clin.Exp.Metastasis.2014.PMID.23975155	1.32	1.12	1.55	0.001	0.003
Activated.B.cell_CellRep.2017.PMID.28052254	1.32	1.12	1.56	0.001	0.003
Activated.Blood.Neutrophil.Signature_Nat.Cell.Biol.2019.PMID.31263265	1.01	0.87	1.18	0.89	0.93
Activated.Cancer.Cell.Signature_Nat.Cell.Biol.2019.PMID.31263265	0.89	0.76	1.04	0.13	0.22
Activated.CD4.T.cell_CellRep.2017.PMID.28052254	1.66	1.41	1.97	<0.001	<0.001
Activated.CD8.T.cell_CellRep.2017.PMID.28052254	1.46	1.24	1.72	<0.001	<0.001
Activated.dendritic.cell_CellRep.2017.PMID.28052254	1.37	1.17	1.62	<0.001	0.001
Activated.Lung.MSC.Signature_Nat.Cell.Biol.2019.PMID.31263265	0.81	0.69	0.95	0.01	0.03
Activated.Lung.Neutrophil.Signature_Nat.Cell.Biol.2019.PMID.31263265	1.29	1.10	1.52	0.002	0.005
aDC_Immunity.2013_PMID.24138885.PMID.24138885	1.44	1.23	1.70	<0.001	<0.001
ADM.S100A10.A110NDGR1.Cluster_BMC.Med.Genomics.2011.PMID.21214954	1.07	0.90	1.27	0.43	0.56
African.and.European.Ancestry.TCGA.Negative_JAMA.Oncol.2017.PMID.28472234	0.90	0.77	1.06	0.21	0.31
African.and.European.Ancestry.TCGA.Positive_JAMA.Oncol.2017.PMID.28472234	0.92	0.79	1.08	0.32	0.44
Age.associated.signature_Genome.Biol.2015.PMID.26343147	0.83	0.71	0.98	0.02	0.05
aMaSC_BCR.2010.PMID.20346151	0.87	0.74	1.02	0.08	0.15
aMaSC.HsEnriched_BCR.2015.PMID.25575446	0.92	0.79	1.08	0.30	0.42
aMaSC.HsEnriched.Refined1_BCR.2015.PMID.25575446	0.84	0.72	0.98	0.03	0.06
aMaSC.Lim09_BCR.2015.PMID.25575446	0.97	0.83	1.14	0.73	0.81
aMaSC.Prat_BCR.2015.PMID.25575446	1.00	0.85	1.17	0.95	0.98
aMaSC.Shehata_BCR.2015.PMID.25575446	0.97	0.83	1.14	0.72	0.80
aMaSC.Signature_Cell.Stem.Cell.2012.PMID.22305568	0.98	0.84	1.15	0.82	0.88
AMPH.EPIREGULIN.Cluster_BMC.Med.Genomics.2011.PMID.21214954	0.87	0.74	1.01	0.08	0.14
Amplification.50_Genome.Biol.2014.PMID.25164602	0.99	0.85	1.16	0.92	0.96
Amplification.50.better.than._Genome.Biol.2015.PMID.25164602	0.85	0.73	1.00	0.06	0.11
Apocrine.Features_J.Pathol.2017.PMID.27861902	1.39	1.18	1.65	<0.001	0.001
aStr.HsEnriched_BCR.2015.PMID.25575446	0.97	0.82	1.13	0.68	0.78
aStr.HsEnriched.Refined1_BCR.2015.PMID.25575446	0.85	0.72	0.99	0.04	0.09
aStr.HsEnriched.Refined2_BCR.2015.PMID.25575446	0.93	0.79	1.08	0.34	0.46
aStr.Lim09_BCR.2015.PMID.25575446	0.91	0.77	1.06	0.22	0.32
aStr.Prat_BCR.2015.PMID.25575446	0.96	0.82	1.13	0.61	0.72
aStr.Shehata_BCR.2015.PMID.25575446	0.97	0.83	1.14	0.73	0.81
BASAL.Cluster_BMC.Med.Genomics.2011.PMID.21214954	0.90	0.77	1.06	0.21	0.32
Bcell.cluster_CCR.2014.PMID.24916698	1.33	1.14	1.57	0.001	0.002
Bcell.IL10.MINUS_Immunol.2014.PMID.25080484	1.12	0.96	1.31	0.16	0.26
Bcell.IL10.PLUS_Immunol.2014.PMID.25080484	1.29	1.10	1.52	0.002	0.007

Bcell.lineage.MCP_Nature.2020.PMID.31942077	1.27	1.08	1.50	0.004	0.01
Bcell.Plasma.52gene_Genome.Biol.2013.PMID.23618380	1.44	1.22	1.70	<0.001	<0.001
Bcell.Plasma.Metagene_Genome.Biol.2013.PMID.23618380	1.43	1.21	1.69	<0.001	<0.001
Bcell.Tcell.Cooperation_Cell.2019.PMID.31730857	1.23	1.05	1.45	0.01	0.03
Bcells_CancerImmunolRes.2018.PMID.30266715	1.33	1.13	1.57	0.001	0.002
Bcells_Immunity.2013.PMID.24138885	1.26	1.07	1.48	0.006	0.02
Bcells.Centroblast_JCO.2015.PMID.25800755	1.40	1.19	1.65	<0.001	<0.001
Bcells.Centrocyte_JCO.2015.PMID.25800755	1.35	1.16	1.59	<0.001	0.001
Bcells.Memory_JCO.2015.PMID.25800755	1.03	0.88	1.21	0.69	0.78
Bcells.memory_Nat.Methods.2015.PMID.25822800	1.32	1.12	1.56	0.001	0.003
Bcells.Naive_JCO.2015.PMID.25800755	1.19	1.01	1.40	0.04	0.08
Bcells.naive_Nat.Methods.2015.PMID.25822800	1.27	1.08	1.49	0.004	0.01
Bcells.Plasmablast_JCO.2015.PMID.25800755	1.47	1.24	1.74	<0.001	<0.001
Blood.vessels_Immunity.2013.PMID.24138885	0.96	0.81	1.12	0.58	0.69
bMYB.Signature_Oncogene.2009.PMID.19043454	1.33	1.14	1.57	0.001	0.002
C3TAG.Responding_CCR.2013.PMID.23780888	0.98	0.84	1.15	0.84	0.90
C3TAG.Untreated_CCR.2013.PMID.23780888	1.29	1.10	1.52	0.002	0.006
CD103.Negative_Cancer.Cell.2014.PMID.25446897	1.09	0.93	1.28	0.30	0.41
CD103.Positive_Cancer.Cell.2014.PMID.25446897	1.20	1.03	1.41	0.02	0.05
CD103.Ratio_Cancer.Cell.2014.PMID.25446897	1.31	1.12	1.54	0.001	0.003
CD274_Single_Gene.Single	1.38	1.17	1.62	<0.001	0.001
CD34.CD36.Cluster_BMC.Med.Genomics.PMID.21214954	0.93	0.79	1.09	0.35	0.47
CD44.downregulated.genes_Cancer.Cell.2007.PMID.17349583	1.42	1.21	1.67	<0.001	<0.001
CD44.upregulated.genes_Cancer.Cell.2007.PMID.17349583	1.06	0.90	1.23	0.49	0.62
CD56bright.natural.killer.cell_CellRep.2017.PMID.28052254	1.22	1.05	1.43	0.01	0.03
CD56dim.natural.killer.cell_CellRep.2017.PMID.28052254	1.32	1.13	1.56	0.001	0.002
CD68.cluster_CCR.2014.PMID.24916698	1.12	0.96	1.31	0.16	0.25
CD8.cluster_CCR.2014.PMID.24916698	1.45	1.24	1.72	<0.001	<0.001
CDKN2A_Single_Gene.Single	0.75	0.63	0.88	0.001	0.002
Central.memory.CD4.T.cell_CellRep.2017.PMID.28052254	0.94	0.81	1.10	0.43	0.56
Central.memory.CD8.T.cell_CellRep.2017.PMID.28052254	1.01	0.87	1.18	0.89	0.94
CES.Score_CCR.2017.PMID.27903675	0.52	0.42	0.65	<0.001	<0.001
Chromogramin_BMC.Med.Genomics.2011.PMID.21214954	0.87	0.74	1.01	0.07	0.13
CIN70_Nat.Genet.2006.PMID.16921376	1.38	1.18	1.63	<0.001	0.001
Claudin.High_Genome.Biol.2007.PMID.17493263	1.32	1.13	1.56	0.001	0.003
Claudin.Low_Genome.Biol.2007.PMID.17493263	1.01	0.86	1.19	0.88	0.93
Claudin.Low.29_Cancer.Res.2009.PMID.19435916	1.16	0.99	1.38	0.07	0.13
cMYB.Signature_PLoS.One.2010.PMID.20949095	0.92	0.79	1.08	0.30	0.42
CORE.Bcell.signature.Garber_Cell.Mol.Gastroenterol.Hepatol.2017.PMID.28508029	1.20	1.02	1.41	0.02	0.05
CTLA4_Single_Gene.Single	1.54	1.31	1.83	<0.001	<0.001
Cytolytic.activity_Cell.2015.PMID.25594174	1.43	1.21	1.69	<0.001	<0.001
Cytotoxic.cells_Immunity.2013.PMID.24138885	1.40	1.19	1.65	<0.001	<0.001
Day7.Downregulated_Nat.Cell.Biol.2014.PMID.25173976	0.93	0.79	1.10	0.39	0.52
Day7.Upregulated_Nat.Cell.Biol.2014.PMID.25173976	1.27	1.08	1.49	0.004	0.01
DC_Immunity.2013.PMID.24138885	1.22	1.04	1.43	0.02	0.04
DCIS.HGF.down_BCR.2013.PMID.24025166	0.88	0.75	1.04	0.13	0.22
DCIS.HGF.up_BCR.2014.PMID.24025166	1.03	0.88	1.21	0.69	0.78

Delection.50_Genome.Biol.2016.PMID.25164602	0.80	0.67	0.94	0.006	0.02
Delection.50.better.than_Genome.Biol.2017.PMID.25164602	0.94	0.80	1.10	0.41	0.54
Dendritic.cells.activated_Nat.Methods.2015.PMID.25822800	1.40	1.18	1.65	<0.001	<0.001
Dendritic.cells.resting_Nat.Methods.2015.PMID.25822800	1.32	1.12	1.55	0.001	0.003
Down.Basal.High_Nat.Cell.Biol.2014.PMID.25173976	0.91	0.78	1.07	0.28	0.39
Down.CLOW.High_Nat.Cell.Biol.2014.PMID.25173976	0.84	0.71	0.98	0.03	0.06
Downregulated.upon.NRAS.repression.basal_Cell.Rep.2015.PMID.26166574	1.21	1.03	1.42	0.02	0.05
Ductal.Carcinoma.In.Situ_J.Pathol.2017.PMID.27861902	1.02	0.88	1.20	0.76	0.83
Duke.Module01.acidosis_PNASUSA.2010.PMID.20335537	0.84	0.72	0.99	0.04	0.08
Duke.Module02.akt_PNASUSA.2010.PMID.20335537	1.09	0.93	1.27	0.29	0.41
Duke.Module03.betacatenin_PNASUSA.2010.PMID.20335537	1.17	1.00	1.37	0.05	0.09
Duke.Module04.E2F1_PNASUSA.2010.PMID.20335537	1.37	1.17	1.60	<0.001	0.001
Duke.Module05.EGFR_PNASUSA.2010.PMID.20335537	1.19	1.02	1.40	0.03	0.06
Duke.Module06.ER_PNASUSA.2010.PMID.20335537	1.04	0.87	1.24	0.70	0.79
Duke.Module07.glucosedepletion_PNASUSA.2010.PMID.20335537	1.25	1.07	1.47	0.005	0.01
Duke.Module08.HER2_PNASUSA.2010.PMID.20335537	1.79	1.50	2.14	<0.001	<0.001
Duke.Module09.hypoxia_PNASUSA.2010.PMID.20335537	1.04	0.89	1.22	0.62	0.73
Duke.Module10.IFNA_PNASUSA.2010.PMID.20335537	1.05	0.90	1.23	0.53	0.65
Duke.Module11.IFNG_PNASUSA.2010.PMID.20335537	1.13	0.96	1.32	0.13	0.22
Duke.Module12.lacticacidosis_PNASUSA.2010.PMID.20335537	1.16	0.98	1.36	0.08	0.14
Duke.Module13.myc_PNASUSA.2010.PMID.20335537	1.13	0.97	1.32	0.12	0.20
Duke.Module14.p53_PNASUSA.2010.PMID.20335537	0.73	0.61	0.88	0.001	0.003
Duke.Module15.p63_PNASUSA.2010.PMID.20335537	1.16	0.99	1.36	0.06	0.12
Duke.Module16.pi3k_PNASUSA.2010.PMID.20335537	1.25	1.07	1.47	0.006	0.02
Duke.Module17.PR_PNASUSA.2010.PMID.20335537	0.62	0.51	0.76	<0.001	<0.001
Duke.Module18.ras_PNASUSA.2010.PMID.20335537	1.31	1.12	1.54	0.001	0.003
Duke.Module19.src_PNASUSA.2010.PMID.20335537	1.01	0.86	1.18	0.93	0.96
Duke.Module20.STAT3_PNASUSA.2010.PMID.20335537	1.28	1.08	1.53	0.005	0.02
Duke.Module21.TGFB_PNASUSA.2010.PMID.20335537	1.15	0.99	1.34	0.08	0.14
Duke.Module22.TNFA_PNASUSA.2010.PMID.20335537	1.30	1.11	1.54	0.001	0.004
Durvalumab.signature_CCR.2018.PMID.29716923	1.39	1.19	1.64	<0.001	<0.001
Early.IRS.1_PLoS.One.2016.PMID.26991655	1.23	1.05	1.45	0.01	0.03
Early.IRS.2_PLoS.One.2016.PMID.26991655	1.05	0.89	1.23	0.57	0.68
Early.Relapse.ERPos.33genes_JAMA.2011.PMID.21558518	0.99	0.85	1.15	0.88	0.93
Early.Response.ERNeg.27genes_JAMA.2011.PMID.21558518	0.97	0.83	1.13	0.69	0.78
Effector.memeory.CD4.T.cell_CellRep.2017.PMID.28052254	1.13	0.96	1.32	0.14	0.22
Effector.memeory.CD8.T.cell_CellRep.2017.PMID.28052254	1.41	1.19	1.66	<0.001	<0.001
EGFR_Single_Gene.Single	0.87	0.73	1.02	0.10	0.17
EMT.down.Taube_PNAS.2010.PMID.20713713	1.37	1.16	1.62	<0.001	0.001
EMT.down.Weingberg_PNAS.2010.PMID.20713713	1.15	0.98	1.35	0.09	0.16
EMT.up.Taube_PNAS.2010.PMID.20713713	0.88	0.75	1.03	0.10	0.17
EMT.up.Weinberg_PNAS.2010.PMID.20713713	0.92	0.78	1.07	0.29	0.40
Endothelial.cells.MCP_Nature.2020..PMID.31942077	0.95	0.81	1.12	0.55	0.67
Endothelial.Normal_Angiogenesis.2014.PMID.24257808	1.29	1.10	1.52	0.002	0.006
Endothelial.Tumor_Angiogenesis.2014.PMID.24257808	1.08	0.92	1.26	0.38	0.50
Eosinophil_CellRep.2017.PMID.28052254	1.11	0.95	1.30	0.18	0.27
Eosinophils_Immunity.2013.PMID.24138885	1.15	0.98	1.36	0.08	0.14

Eosinophils_Nat.Methods.2015.PMID.25822800	1.23	1.05	1.44	0.01	0.03
Epithelial.Tubule.Formation_J.Pathol.2017.PMID.27861902	0.67	0.56	0.79	<0.001	<0.001
ERBB2_Single_Gene.Single	1.94	1.63	2.34	<0.001	<0.001
ERBB3_Single_Gene.Single	1.11	0.94	1.31	0.24	0.34
ESR1_Single_Gene.Single	0.48	0.38	0.60	<0.001	<0.001
ESTIMATE.Immune_Nat.Commun.2013.PMID.24113773	1.41	1.20	1.67	<0.001	<0.001
ESTIMATE.Stromal_Nat.Commun.2013.PMID.24113773	1.03	0.87	1.20	0.76	0.83
Euclidean.Distance.CLOW_BCR.2010.PMID.20813035	1.16	0.99	1.36	0.07	0.14
EXTENDED.Bcell.signature.Garber_Cell.Mol.Gastroenterol.Hepatol.2017.PMID.28508029	1.12	0.96	1.31	0.16	0.25
FGFR4_Single_Gene.Single	1.45	1.24	1.71	<0.001	<0.001
FGFR4.Induced_JCI.2020.PMID.32573490	1.41	1.19	1.67	<0.001	<0.001
FGFR4.Repressed_JCI.2020.PMID.32573490	0.82	0.69	0.97	0.02	0.05
Fibrinogen.Cluster_BMC.Med.Genomics.2011.PMID.21214954	0.75	0.64	0.88	0.001	0.002
Fibroblast.Cluster_BMC.Med.Genomics.2011.PMID.21214954	0.87	0.75	1.02	0.09	0.16
Fibroblasts.MCP_Nature.2020.PMID.31942077	0.91	0.78	1.06	0.24	0.35
Fibromatosis_Lab.Invest.2008.PMID.18414401	0.88	0.76	1.03	0.13	0.21
fMaSC.Metab_CellRep.2018.PMID.30089273	0.81	0.69	0.96	0.01	0.03
fMaSC.Metab8_CellRep.2018.PMID.30089273	1.21	1.03	1.43	0.02	0.05
fMaSC.refined1_BCR.2015.PMID.25575446	1.02	0.87	1.20	0.77	0.83
fMasC.Signature_Cell.Stem.Cell.2012.PMID.22305568	0.94	0.80	1.10	0.45	0.57
fMaSC.Signature_CellRep.2018.PMID.30089273	1.23	1.05	1.44	0.01	0.03
FOS.JUN_Cluster_BMC.Med.Genomics.2011.PMID.21214954	0.97	0.83	1.14	0.74	0.81
FOXC1.Hair.Follicles.P30C.LO.vs.WT.Negative_Science.2016.PMID.26912704	0.90	0.77	1.06	0.21	0.31
FOXC1.Hair.Follicles.P30C.LO.vs.WT.Positive_Science.2016.PMID.26912704	1.05	0.90	1.23	0.51	0.64
fSTR.Signature_Cell.Stem.Cell.2012.PMID.22305568	0.75	0.63	0.88	<0.001	0.002
Gamma.delta.T.cell_CellRep.2017.PMID.28052254	1.08	0.92	1.26	0.35	0.47
GATA3.induced.genes_JCO.2006.PMID.16505416	1.48	1.26	1.76	<0.001	<0.001
GATA3.induced.genes_Oncogene.2004.PMID.15361840	1.36	1.15	1.61	<0.001	0.001
GDF11.TGFBR3_Nat.Cell.Biol.2014.PMID.24658685	1.01	0.86	1.18	0.94	0.97
Glycolysis_BMC.Med.2009.PMID.19291283	1.32	1.12	1.57	0.001	0.003
GO.DOWN.with.SOX10.OE_Cell.Rep.2015.PMID.26365194	1.26	1.07	1.49	0.006	0.02
GO.UP.with.SOX10.OE_Cell.Rep.2015.PMID.26365194	0.96	0.81	1.12	0.57	0.69
GSEA_BIOCARTA_ALK_PATHWAY.PMID.16199517	0.88	0.75	1.03	0.10	0.17
GSEA_BIOCARTA.AKT.PATHWAY.PMID.16199517	1.17	1.00	1.37	0.05	0.10
GSEA_BIOCARTA.BRCA.ATR.PATHWAY.ATRBRCA.PMID.16199517	1.11	0.94	1.30	0.22	0.32
GSEA_BIOCARTA.CASPASE.PATHWAY.PMID.16199517	1.38	1.17	1.62	<0.001	0.001
GSEA_BIOCARTA.CTLA4.PATHWAY.PMID.16199517	1.47	1.25	1.73	<0.001	<0.001
GSEA_BIOCARTA.IGF1R.PATHWAY.PMID.16199517	0.81	0.69	0.95	0.01	0.03
GSEA_BIOCARTA.MTOR.PATHWAY.PMID.16199517	1.14	0.98	1.34	0.10	0.17
GSEA_BIOCARTA.PTEN.PATHWAY.PMID.16199517	0.90	0.77	1.06	0.20	0.31
GSEA_BIOCARTA.RAS.PATHWAY.PMID.16199517	1.01	0.86	1.19	0.90	0.94
GSEA_BIOCARTA.RB.PATHWAY.PMID.16199517	1.47	1.25	1.74	<0.001	<0.001
GSEA_BIOCARTA.VEGF.PATHWAY.PMID.16199517	1.12	0.96	1.31	0.15	0.25
GSEA_HALLMARK.MYC.TARGETS.V1.PMID.16199517	0.95	0.81	1.11	0.51	0.64
GSEA_HELLER.HDAC.TARGETS.DOWN.PMID.16199517	1.39	1.18	1.65	<0.001	0.001
GSEA_NELSON.RESPONSE.TO.ANDROGEN.UP.PMID.16199517	1.34	1.14	1.58	<0.001	0.001
GSEA_REACTOME.PD1.SIGNALING.PMID.16199517	1.40	1.19	1.65	<0.001	<0.001

GSEA_REACTOME.PI3K.CASCADE.PMID.16199517	0.89	0.76	1.04	0.15	0.25
GSEA_RETINOL.METABOLISM.KEGG.PMID.16199517	0.96	0.82	1.12	0.59	0.70
GSEA.GP1_Proliferation.DNA.repair..PUJANA.CHEK2.PCC.NETWORK.PMID.25109877	1.25	1.07	1.47	0.005	0.01
GSEA.GP1_Proliferation.DNA.repair.REACTOME.CELL.CYCLE.MITOTIC.PMID.25109877	1.23	1.05	1.44	0.01	0.03
GSEA.GP10_Fatty.acid.oxidation.CARBOXYLIC.ACID.METABOLIC.PROCESS.PMID.25109877	0.89	0.76	1.04	0.15	0.24
GSEA.GP11_Immune.IFN.PerouLab.PMID.25109877	1.10	0.94	1.28	0.25	0.36
GSEA.GP12_Hypoxia.glycolosis.SEMENZA.HIF1.TARGETS.PMID.25109877	1.08	0.92	1.26	0.34	0.46
GSEA.GP13_Neural.signailng.MODULE100.PMID.25109877	0.93	0.79	1.08	0.33	0.45
GSEA.GP13_Neural.signailng.NERVOUS.SYSTEM.DEVELOPMENT.PMID.25109877	0.83	0.71	0.97	0.02	0.05
GSEA.GP14_Plasma.membrane.cell.cell.signaling.MORF.CNTN1.PMID.25109877	0.97	0.83	1.13	0.68	0.78
GSEA.GP15_EGF.signaling.NAGASHIMA.EGF.SIGNALING.UP.PMID.25109877	1.00	0.85	1.16	0.96	0.99
GSEA.GP16_Protein.kinase.signaling.MAPKs.INTRACELLULAR.SIGNALING.CASCADE.PMID.25109877	1.43	1.21	1.70	<0.001	<0.001
GSEA.GP16_Protein.kinase.signaling.MAPKs.REGULATION.OF.KINASE.ACTIVITY.PMID.25109877	1.32	1.12	1.56	0.001	0.004
GSEA.GP17_Basal.signaling.SMID.BREAST.CANCER.BASAL.UP.PMID.25109877	1.17	0.97	1.40	0.10	0.17
GSEA.GP18_Vesicle.EPR.MEMBRANE.COAT.PMID.25109877	1.23	1.05	1.44	0.01	0.03
GSEA.GP19_1Q.amplicon.PerouLab.PMID.25109877	0.94	0.81	1.10	0.46	0.58
GSEA.GP2_Immune.Tcell.Bcell.KEGG.HEMATOPOIETIC.CELL.LINEAGE.PMID.25109877	1.33	1.13	1.56	<0.001	0.002
GSEA.GP2_Immune.Tcell.Bcell.PerouLab.PMID.25109877	1.40	1.19	1.65	<0.001	<0.001
GSEA.GP20_TAL1.Leukemia.erythropoiesis.GNF2.TAL1.PMID.25109877	0.86	0.73	1.00	0.05	0.10
GSEA.GP21_Anti.apoptosis.DNA.stability.MORF.BCL2.PMID.25109877	0.82	0.70	0.97	0.02	0.04
GSEA.GP21_Anti.apoptosis.DNA.stability.MORF.MT4.PMID.25109877	0.95	0.81	1.11	0.53	0.65
GSEA.GP21_Anti.apoptosis.DNA.stability.MORF.STK17A.PMID.25109877	0.83	0.70	0.97	0.02	0.04
GSEA.GP22_16Q22.24.amplicon.PerouLab.PMID.25109877	1.04	0.89	1.21	0.66	0.76
GSEA.GP3_Tumo.suppressing.miRNA.targets.GTTTGTT.MIR.495.PMID.25109877	1.09	0.93	1.28	0.27	0.38
GSEA.GP3_Tumor.suppressing.miRNA.targets.DACOSTA.UV.RESPONSE.VIA.ERCC3.DN.PMID.25109877	1.08	0.92	1.27	0.33	0.45
GSEA.GP3_Tumor.suppressing.miRNA.targets.TGCTTTG.MIR.330.PMID.25109877	1.06	0.91	1.24	0.46	0.59
GSEA.GP4_MES.ECM.PerouLab.PMID.25109877	0.88	0.75	1.03	0.12	0.21
GSEA.GP5_MYC.targets.TERT.PerouLab.PMID.25109877	1.11	0.95	1.30	0.21	0.31
GSEA.GP6_Squamous.differentiation.development.RICKMAN.TUMOR.DIFFERENTIATED.WELL.VS.POORLY.DN.PMID.25109877	1.30	1.10	1.54	0.002	0.007
GSEA.GP7_Estrogen.signaling.SMID.BREAST.CANCER.BASAL.DN.PMID.25109877	0.83	0.68	1.00	0.05	0.09
GSEA.GP8_FOXO.stemness.MORF.PTPRB.PMID.25109877	0.86	0.73	1.00	0.05	0.10
GSEA.GP8_FOXO.stemness.TTGTTT.VSFOXO4.01.PMID.25109877	1.01	0.86	1.19	0.88	0.93
GSEA.GP9_Cell.cell.adhesion.PerouLab.PMID.25109877	1.17	0.99	1.38	0.07	0.12
HCK_BCR.2008.PMID.19272155	1.33	1.13	1.57	0.001	0.002
HER1.Cluster1_BMC.Genomics.2007.PMID.17663798	1.06	0.91	1.25	0.44	0.57
HER1.Cluster2_BMC.Genomics.2007.PMID.17663798	1.12	0.95	1.31	0.18	0.27
HER1.Cluster3_BMC.Genomics.2007.PMID.17663798	1.19	1.02	1.39	0.03	0.07
HER2.Amplicon.PerouLab_BMC.Med.Genomic.2011.PMID.21214954	1.52	1.29	1.79	<0.001	<0.001
Histological.Grade_J.Pathol.2017.PMID.27861902	1.40	1.19	1.66	<0.001	<0.001
HouseKeeping_Genome.Biol.2004.PMID.15287981	1.05	0.90	1.23	0.52	0.65
iDC.Median_Immunity.2013.PMID.24138885	1.17	1.00	1.37	0.06	0.11
IFN.Cluster_BMC.Med.Genomics.2011.PMID.21214954	0.92	0.79	1.07	0.30	0.41
IgG_BCR.2008.PMID.19272155	1.42	1.20	1.68	<0.001	<0.001
IGG.Cluster_BMC.Med.Genomics.2011.PMID.21214954	1.38	1.18	1.63	<0.001	0.001
Immature..B.cell_CellRep.2017.PMID.28052254	1.31	1.12	1.54	0.001	0.003
Immature.dendritic.cell_CellRep.2017.PMID.28052254	0.96	0.82	1.12	0.57	0.68
ImmLandscape_Macro.mono.CSF1.core.response_CCR.2009.PMID.29628290	1.41	1.20	1.67	<0.001	<0.001

ImmLandscape_Wound.Healing_Immunity.2018.PMID.29628290	1.19	1.02	1.40	0.03	0.06
ImmLandscape.IFN3_Plos.One.2014.PMID.24516633	0.90	0.77	1.05	0.16	0.26
ImmLandscape.IFNG5_Plos.One.2014.PMID.24516633	1.50	1.28	1.78	<0.001	<0.001
ImmLandscape.lymphocyte.Infil.T.B.PMID.18592372	1.40	1.19	1.65	<0.001	<0.001
Immune.Hot.CD8.vs.Cold_Nature.2020.PMID.31942071	1.40	1.19	1.65	<0.001	<0.001
Immune.Perez.14_JCO.2015.PMID.25605861	1.27	1.08	1.49	0.004	0.01
Immune.Perez.87_JCO.2015.PMID.25605861	1.45	1.24	1.72	<0.001	<0.001
Immune.Suppression_JCI.Insight.2016.PMID.27699256	1.42	1.21	1.67	<0.001	<0.001
ImmuneActive_Cell.2019.PMID.31730857	1.48	1.26	1.75	<0.001	<0.001
Immunosuppression.PMID.31942077	1.03	0.89	1.21	0.68	0.78
IMS.Score_CCR.2018.PMID.29921729	0.91	0.78	1.07	0.26	0.37
Induced.in.Bcells_PNAS.2013.PMID.23382184	1.25	1.07	1.48	0.006	0.02
Induced.in.DC_PNAS.2013.PMID.23382184	1.41	1.19	1.67	<0.001	<0.001
Induced.in.GN_PNAS.2013.PMID.23382184	1.22	1.03	1.44	0.02	0.05
Induced.in.HSC_PNAS.2013.PMID.23382184	1.09	0.94	1.28	0.25	0.37
Induced.in.MOs_PNAS.2013.PMID.23382184	1.25	1.06	1.48	0.007	0.02
Induced.in.NKcells_PNAS.2013.PMID.23382184	1.27	1.08	1.50	0.004	0.01
Induced.in.Tcells_PNAS.2013.PMID.23382184	1.20	1.02	1.40	0.03	0.06
Inflammatory.breast.cancer.491genes_CCR.2013.PMID.23396049	1.48	1.26	1.76	<0.001	<0.001
Inflammatory.breast.cancer.79genes_CCR.2013.PMID.23396049	1.43	1.21	1.69	<0.001	<0.001
Inflammatory.breast.cancer.expressed.noIBC_79genes_CCR.2013.PMID.23396049	0.91	0.77	1.06	0.21	0.32
Inflammatory.breast.cancer.expressed.noIBC.491genes_CCR.2013.PMID.23396049	0.80	0.67	0.95	0.01	0.03
Influenza.11genes.Metasignature_Immunity.2015.PMID.26682989	0.97	0.83	1.13	0.73	0.81
Interferon_BCR.2008.PMID.19272155	0.87	0.74	1.02	0.08	0.14
Interferon.Pathway_CancerImmunolRes.2018.PMID.30266715	0.99	0.85	1.15	0.89	0.94
JUND.KRT5_Nat.Cell.Biol.2014.PMID.24658685	0.87	0.74	1.02	0.09	0.15
Keller2012.CD10.Adam_BCR.2015.PMID.25575446	1.15	0.97	1.35	0.10	0.18
KRAS.amplicon_Genome.Biology.2007.PMID.17493263	1.18	1.01	1.39	0.04	0.07
Late.IRS.1_PLoS.One.2016.PMID.26991655	1.23	1.05	1.44	0.009	0.02
Late.IRS.2_PLoS.One.2016.PMID.26991655	0.97	0.83	1.13	0.68	0.78
LCK_BCR.2008.PMID.19272155	1.41	1.20	1.66	<0.001	<0.001
Lim2009.LumProg.Adam_BCR.2015.PMID.25575446	1.13	0.95	1.35	0.16	0.25
Lim2009.MaSC.Adam_BCR.2015.PMID.25575446	0.94	0.80	1.10	0.45	0.58
Lim2009.MatureLum.Adam_BCR.2015.PMID.25575446	0.88	0.73	1.06	0.19	0.28
Lim2009.Stroma.Adam_BCR.2015.PMID.25575446	0.88	0.75	1.03	0.11	0.18
Lim2010.LumProg.Adam_BCR.2015.PMID.25575446	1.36	1.16	1.59	<0.001	0.001
Lim2010.MaSC.Adam_BCR.2015.PMID.25575446	1.00	0.85	1.18	0.98	> 0.99
Lim2010.MatureLum.Adam_BCR.2015.PMID.25575446	0.80	0.68	0.93	0.005	0.01
Lim2010.Stroma.Adam_BCR.2015.PMID.25575446	0.86	0.73	1.01	0.06	0.12
Lobular.Carcinoma.In.Situ_J.Pathol.2017.PMID.27861902	0.86	0.73	1.01	0.06	0.11
LOBULAR.TCGA.SIGNATURE.ImmuneCell.2015.PMID.26451490	1.38	1.17	1.63	<0.001	0.001
LOBULAR.TCGA.SIGNATURE.Reactive_Cell.2015.PMID.26451490	0.88	0.75	1.03	0.12	0.21
LOBULAR.TCGA.SUBTYPE.Immune_Cell.2015.PMID.26451490	0.98	0.83	1.14	0.76	0.82
LOBULAR.TCGA.SUBTYPE.Proliferative_Cell.2015.PMID.26451490	0.95	0.82	1.12	0.56	0.68
LOBULAR.TCGA.SUBTYPE.Reactive_Cell.2015.PMID.26451490	0.93	0.80	1.09	0.38	0.51
LTS.score_JCI.2020.PMID.32573490	0.71	0.60	0.83	<0.001	<0.001
Luminal_Progenitor_Up_Nat.Med.2009.PMID.19648928	1.32	1.11	1.59	0.002	0.007

Luminal.cluster_BMC.Med.Genomics.2011.PMID.21214954	0.82	0.68	1.00	0.05	0.09
Luminal.Progenitor_BCR.2010.PMID.20346151	1.26	1.06	1.51	0.009	0.02
Luminal.Progenitor.Down_Nat.Med.2009.PMID.19648928	1.03	0.88	1.20	0.72	0.80
LumProg.HsEnriched_BCR.2015.PMID.25575446	1.22	1.02	1.46	0.03	0.06
LumProg.HsEnriched.Refined1_BCR.2015.PMID.25575446	0.94	0.80	1.11	0.48	0.62
LumProg.Lim09_BCR.2015.PMID.25575446	1.24	1.06	1.47	0.01	0.03
LumProg.Prat_BCR.2015.PMID.25575446	1.20	1.02	1.42	0.03	0.06
LumProg.Shehata_BCR.2015.PMID.25575446	1.08	0.92	1.27	0.33	0.45
Lums.HER2E.DOWN.metastatic.signature_JCI.2020.PMID.32573490	0.68	0.57	0.80	<0.001	<0.001
Lums.HER2E.UP.metastatic.signature_JCI.2020.PMID.32573490	1.32	1.12	1.57	0.001	0.004
Lung.WNT_Cancer.Res.2009.PMID.19549913	1.08	0.92	1.26	0.37	0.49
Lymph.vessels_Immunity.2013.PMID.24138885	1.02	0.87	1.20	0.79	0.85
Lymphovascular.Invasion_J.Pathol.2017.PMID.27861902	1.18	1.00	1.38	0.04	0.09
M.D.Metagene_Genome.Biol.2013.PMID.23618380	1.37	1.17	1.62	<0.001	0.001
M2.Macrophage_Blood.2006.PMID.16556895	1.18	1.01	1.38	0.04	0.08
Macrophage_CellRep.2017.PMID.28052254	0.95	0.81	1.12	0.57	0.68
Macrophages_CancerImmunolRes.2018.PMID.30266715	1.29	1.10	1.52	0.002	0.006
Macrophages_Immunity.2013.PMID.24138885	1.24	1.05	1.46	0.01	0.03
Macrophages.M0_Nat.Methods.2015.PMID.25822800	1.50	1.27	1.77	<0.001	<0.001
Macrophages.M1_Nat.Methods.2015.PMID.25822800	1.46	1.24	1.72	<0.001	<0.001
Macrophages.M2_Nat.Methods.2015.PMID.25822800	1.26	1.07	1.49	0.005	0.02
MacTh1.cluster_CCR.2014.PMID.24916698	1.42	1.20	1.67	<0.001	<0.001
MammaPrint_Nature.2002.PMID.11823860	0.68	0.57	0.80	<0.001	<0.001
MAPK.pathway.activation_NPJ.Precis.Oncol.2018.PMID.29872725	1.17	0.99	1.37	0.06	0.12
MASC.Down_Nat.Med.2009.PMID.19648928	0.98	0.83	1.15	0.76	0.83
MASC.Up_Nat.Med.2009.PMID.19648928	0.91	0.77	1.06	0.23	0.33
Mast.cell_CellRep.2017.PMID.28052254	1.09	0.93	1.28	0.26	0.38
Mast.cells_Immunity.2013.PMID.24138885	0.81	0.69	0.95	0.01	0.03
Mast.cells.activated_Nat.Methods.2015.PMID.25822800	1.01	0.86	1.18	0.95	0.98
Mast.cells.resting_Nat.Methods.2015.PMID.25822800	1.03	0.88	1.20	0.75	0.82
Mature.luminal_BCR.2010.PMID.20346151	0.98	0.82	1.17	0.83	0.89
Mature.Luminal.Down_Nat.Med.2009.PMID.19648928	1.04	0.87	1.23	0.68	0.78
Mature.LuminaUp_Nat.Med.2009.PMID.19648928	0.88	0.74	1.06	0.19	0.28
MatureLum.HsEnriched_BCR.2015.PMID.25575446	0.88	0.73	1.06	0.18	0.28
MatureLum.HsEnriched.Refined1_BCR.2015.PMID.25575446	1.00	0.83	1.20	0.98	> 0.99
MatureLum.Lim09_BCR.2015.PMID.25575446	0.90	0.76	1.08	0.27	0.39
MatureLum.Prat_BCR.2015.PMID.25575446	1.05	0.89	1.25	0.55	0.67
MatureLum.Shehata_BCR.2015.PMID.25575446	0.99	0.84	1.18	0.94	0.97
MBasal.Cluster_BMC.Med.Genomics.2011.PMID.21214954	0.91	0.78	1.07	0.26	0.37
MCD3.CD8_BMC.Med.Genomics.2011.PMID.21214954	1.38	1.17	1.62	<0.001	0.001
MCF7.E2.induced.genes_JCO.2006.PMID.16505416	0.95	0.82	1.11	0.53	0.66
MCF7.E2.repressed.genes_JCO.2006.PMID.16505416	0.92	0.77	1.09	0.32	0.44
MDSC_CellRep.2017.PMID.28052254	1.50	1.27	1.78	<0.001	<0.001
MDSC.Granulocytic_Leukoc.Biol.2012.PMID.21954284	1.12	0.96	1.30	0.17	0.26
MDSC.Neutrophi_Leukoc.Biol.2012.PMID.21954284	1.35	1.15	1.59	<0.001	0.001
MDSC.tumor_J.Immunol.2012.PMID.23152559	1.16	0.99	1.37	0.07	0.13
MDSC.tumor.MO_J.Immunol.2012.PMID.23152559	1.32	1.12	1.56	0.001	0.004

MECM_BMC.Med.Genomics.2011.PMID.21214954	0.90	0.77	1.05	0.18	0.28
Memory.B.cell_CellRep.2017.PMID.28052254	1.02	0.88	1.20	0.77	0.83
MET.DOWN.RNAseq.Significant.Genes_JCI.2018.PMID.29480819	1.05	0.90	1.23	0.56	0.68
MET.DOWN.Significant.Genes.Low.Basal.1_JCI.2018.PMID.29480819	0.95	0.82	1.11	0.55	0.67
MET.DOWN.Significant.Genes.Low.Basal.2_JCI.2018.PMID.29480819	0.96	0.82	1.13	0.60	0.71
MET.UP.RNAseq.Significant.Genes_JCI.2018.PMID.29480819	0.82	0.70	0.96	0.01	0.03
MET.UP.Significant.Genes.HIGH.BASALS.Genes_JCI.2018.PMID.29480819	0.91	0.77	1.06	0.22	0.32
Metaplastic.Up_CanRes.2009.PMID.19435916	0.95	0.80	1.12	0.53	0.65
Metastasis.predictor.TNBC_BCR.2010.PMID.20946665	1.22	1.04	1.43	0.02	0.04
MFGFR2_BMC.Med.Genomics.2011.PMID.21214954	1.07	0.91	1.25	0.45	0.57
MHC.Forero.11_Cancer.Immunol.Res.2016.PMID.26980599	1.36	1.16	1.60	<0.001	0.001
MHC.Forero.24_Cancer.Immunol.Res.2016.PMID.26980599	1.15	0.98	1.34	0.08	0.14
MHC.I_BCR.2008.PMID.19272155	1.16	0.99	1.35	0.07	0.13
MHC.II_BCR.2008.PMID.19272155	1.44	1.23	1.70	<0.001	<0.001
MHCI.coreGenes_Nat.Commun.2017.PMID29170503	1.27	1.09	1.49	0.003	0.008
MIR200c.Induced_ONCO.2015.PMID.25746005	1.22	1.04	1.43	0.02	0.04
MIR200c.Repressed_ONCO.2015.PMID.25746005	1.00	0.85	1.18	0.97	0.99
miRNA.138.signature_Cancer.Res.2014.PMID.25339353	1.23	1.05	1.44	0.01	0.03
MITO1_BMC.Med.Genomics.2011.PMID.21214954	1.16	0.99	1.36	0.06	0.12
MITO2_BMC.Med.Genomics.2011.PMID.21214954	1.03	0.88	1.20	0.71	0.80
Mitotic.Count_J.Pathol.2017.PMID.27861902	1.21	1.03	1.42	0.02	0.05
MK14.K17_BMC.Med.Genomics.2011.PMID.21214954	1.03	0.88	1.20	0.74	0.82
MKRAS.amplicon_BMC.Med.Genomics.2011.PMID.21214954	1.20	1.03	1.41	0.02	0.05
MM.BRCAwnt.1pFDR.UP_Genome.Biology.2007.PMID.17493263	1.05	0.89	1.23	0.55	0.67
MM.C3Tag.1pFDR.UP_Genome.Biology.2007.PMID.17493263	1.24	1.06	1.46	0.009	0.02
MM.C3Tag.2012_Genome.Biol.2013.PMID.24220145	1.34	1.15	1.58	<0.001	0.001
MM.Class3_Genome.Biol.2013.PMID.24220145	1.21	1.04	1.42	0.02	0.04
MM.Class8_Genome.Biol.2013.PMID.24220145	0.87	0.74	1.01	0.07	0.13
MM.Claudinlow_Genome.Biol.2013.PMID.24220145	1.00	0.85	1.18	>0.99	> 0.99
MM.DMBAwnt.1pFDR.UP.Genome.Biology.2007.PMID.17493263	1.00	0.85	1.17	0.97	0.99
MM.Erbb2.like_Genome.Biol.2013.PMID.24220145	1.05	0.90	1.23	0.52	0.64
MM.Myc.2012_Genome.Biol.2013.PMID.24220145	1.16	0.99	1.36	0.07	0.13
MM.Myoepithelioma.like_Genome.Biol.2013.PMID.24220145	0.97	0.83	1.14	0.73	0.81
MM.Neu.2012_Genome.Biol.2013.PMID.24220145	1.17	1.00	1.38	0.05	0.09
MM.NeuPyMT.1pFDR.UP_Genome.Biology.2007.PMID.17493263	1.07	0.91	1.26	0.39	0.52
MM.Normal.1pFDR.UP_Genome.Biology.2007.PMID.17493263	0.97	0.83	1.14	0.74	0.81
MM.Normal.like_Genome.Biol.2013.PMID.24220145	0.99	0.85	1.16	0.95	0.98
MM.p53null.1pFDR.UP_Genome.Biology.2007.PMID.17493263	1.06	0.90	1.24	0.50	0.63
MM.p53null.Basal_Genome.Biol.2013.PMID.24220145	1.00	0.85	1.17	0.97	0.99
MM.p53null.Luminal_Genome.Biol.2013.PMID.24220145	1.00	0.85	1.17	>0.99	> 0.99
MM.Potluck_1pFDR_UP_Genome.Biology.2007_PMID.17493263.PMID.24220145	1.09	0.92	1.28	0.33	0.45
MM.PyMT.2012_Genome.Biol.2013.PMID.24220145	1.26	1.08	1.49	0.004	0.01
MM.Squamous.like_Genome.Biol.2013.PMID.24220145	1.10	0.93	1.29	0.26	0.37
MM.Stat1_Genome.Biol.2013.PMID.24220145	1.00	0.86	1.17	>0.99	> 0.99
MM.WapINT3.1pFDR.UP_Genome.Biology.2007.PMID.17493263	1.15	0.98	1.35	0.08	0.14
MM.WapINT3.2012_Genome.Biol.2013.PMID.24220145	0.99	0.85	1.15	0.87	0.93
MM.WAPTag.1pFDR.UP_Genome.Biology.2007.PMID.17493263	1.34	1.14	1.58	<0.001	0.002

MM.Wnt1.Early_Genome.Biol.2013.PMID.24220145	1.11	0.95	1.31	0.19	0.29
MM.Wnt1.Late_Genome.Biol.2013.PMID.24220145	1.05	0.90	1.23	0.55	0.67
Mmyosin_BMC.Med.Genomics.2011.PMID.21214954	0.79	0.68	0.93	0.004	0.01
MNADH_CYTochrome_BMC.Med.Genomics.2011.PMID.21214954	1.23	1.05	1.44	0.01	0.03
MNB1_BMC.Med.Genomics.2011.PMID.21214954	1.04	0.89	1.21	0.66	0.76
MNB2_BMC.Med.Genomics.2011.PMID.21214954	1.05	0.89	1.23	0.57	0.68
MNB3_BMC.Med.Genomics.2011.PMID.21214954	0.84	0.69	1.01	0.08	0.14
MNOtch4_BMC.Med.Genomics.2011.PMID.21214954	1.06	0.91	1.25	0.43	0.56
Monocyte_CellRep.2017.PMID.28052254	1.00	0.85	1.18	0.96	0.99
Monocyte..DC.25gene_Genome.Biol.2013.PMID.23618380	1.38	1.18	1.63	<0.001	0.001
Monocytes_CancerImmunolRes.2018.PMID.30266715	1.19	1.01	1.39	0.03	0.07
Monocytes_Nat.Methods.2015.PMID.25822800	1.29	1.10	1.53	0.002	0.006
Monocytic.lineage.MCP_Nature.2020.PMID.31942075	1.22	1.04	1.44	0.02	0.04
MProliferation_BMC.Med.Genomics.2011.PMID.21214954	1.36	1.16	1.60	<0.001	0.001
MProtocadherin_BMC.Med.Genomics.2011.PMID.21214954	1.04	0.89	1.22	0.65	0.75
MPYMT_NEU_Cluster_BMC.Med.Genomics.2011.PMID.21214954	1.12	0.96	1.32	0.15	0.24
MRibosomal_BMC.Med.Genomics.2011.PMID.21214954	0.96	0.82	1.12	0.60	0.71
MS.CD44.DOWN_PNAS.2009.PMID.19666588	1.04	0.89	1.23	0.60	0.71
MS.CD44.UP_PNAS.2009.PMID.19666588	1.03	0.88	1.22	0.68	0.78
MSquamous_BMC.Med.Genomics.2011.PMID.21214954	0.93	0.79	1.08	0.34	0.46
Murat.G07_JCO.2008.PMID.18565887	0.95	0.81	1.11	0.50	0.63
Murat.G18_JCO.2008.PMID.18565887	0.88	0.75	1.04	0.13	0.21
Murat.G24_JCO.2008.PMID.18565887	1.47	1.24	1.74	<0.001	<0.001
MVEGFC_BMC.Med.Genomics.2011.PMID.21214954	0.84	0.72	0.99	0.03	0.07
Myeloid.cell.chemotaxis.1gene_Nature.2020.PMID.31942077	1.05	0.90	1.23	0.55	0.67
Myeloid.dendritic.cells.MCP_Nature.2020.PMID.31942077	1.17	1.00	1.37	0.05	0.09
Natural.killer.cell_CellRep.2017.PMID.28052254	0.91	0.77	1.06	0.22	0.32
Natural.killer.T.cell_CellRep.2017.PMID.28052254	1.12	0.96	1.32	0.15	0.25
Necrosis_J.Pathol.2017.PMID.27861902	1.33	1.13	1.58	0.001	0.003
Neutrophil_CellRep.2017.PMID.28052254	1.00	0.86	1.17	0.99	> 0.99
Neutrophils_CancerImmunolRes.2018.PMID.30266715	1.40	1.18	1.68	<0.001	0.001
Neutrophils_Immunity.2013.PMID.24138885	1.13	0.96	1.33	0.13	0.22
Neutrophils_Nat.Methods.2015.PMID.25822800	1.21	1.03	1.43	0.02	0.05
Neutrophils.MCP_Nature.2020.PMID.31942077	0.90	0.77	1.05	0.18	0.28
NK_Immunity.2013.PMID.24138885	0.97	0.83	1.13	0.70	0.79
NK.activated_Nat.Methods.2015.PMID.25822800	1.40	1.19	1.65	<0.001	<0.001
NK.CD56bright_Immunity.2013.PMID.24138885	1.19	1.02	1.39	0.03	0.06
NK.CD56dim_Immunity.2013.PMID.24138885	1.56	1.32	1.85	<0.001	<0.001
NK.resting_Nat.Methods.2015.PMID.25822800	1.40	1.19	1.65	<0.001	<0.001
NKcells_CancerImmunolRes.2018.PMID.30266715	1.41	1.20	1.66	<0.001	<0.001
NKcells.MCP_Nature.2020.PMID.31942077	1.01	0.86	1.18	0.90	0.94
No.Response.Immunotherapy.TLS.Melanoma_Nature.2020.PMID.31942075	0.81	0.69	0.96	0.01	0.03
Normal.mucosa_Immunity.2013.PMID.24138885	0.88	0.75	1.02	0.10	0.17
Nuclear.Pleomorphism_J.Pathol.2017.PMID.27861902	0.97	0.83	1.14	0.75	0.82
Oncotype_NEJM.2004.PMID.15591335	1.77	1.49	2.11	<0.001	<0.001
P53.ERPos.MDACC_CCR.2011.PMID.21248301	1.37	1.17	1.61	<0.001	0.001
Parity.signature.251genes_BCR.2014.PMID.25005139	1.28	1.09	1.51	0.003	0.009

Parity.signature.40genes_BCR.2014.PMID.25005139	1.34	1.14	1.58	<0.001	0.002
PARPi.Resistance_BCRT_2012.PMID.22875744	1.20	1.03	1.40	0.02	0.05
PARPi.Sensitivity_BCRT_2012.PMID.22875744	0.92	0.78	1.07	0.28	0.40
PARPi.Sensitivity.MDACC_NPJ.Syst.Biol.Appl.2017.PMID.28649435	1.19	1.02	1.39	0.03	0.06
PARPi.Sensitivity.Negative_Sci.Adv.2017.PMID.28439535	1.13	0.96	1.32	0.14	0.22
PARPi.Sensitivity.Positive_Sci.Adv.2017.PMID.28439535	1.18	1.00	1.38	0.04	0.09
Pcorr.Breast2Lung.LM2.Correlation_Nature.2005.PMID.16049480	1.01	0.86	1.18	0.91	0.95
Pcorr.Breast2Lung.Parental.Correlation_Nature.2005.PMID.16049480	1.03	0.89	1.21	0.67	0.77
Pcorr.dasatinib.resistant_Cancer.Res.2007.PMID.17332353	0.96	0.81	1.13	0.61	0.71
Pcorr.dasatinib.sensitive_Cancer.Res.2007.PMID.17332353	1.02	0.86	1.22	0.80	0.86
Pcorr.Hypoxia.High.Correlation_PLoS.Med.2006.PMID.16417408	0.95	0.81	1.11	0.54	0.66
Pcorr.Hypoxia.Low.Correlation_PLoS.Med.2006.PMID.16417408	1.00	0.85	1.17	0.98	> 0.99
Pcorr.IGS_Invasiveness_NJEM.2007.PMID.17229949	1.39	1.18	1.64	<0.001	<0.001
Pcorr.wound.response.activated_PNAS.2005.PMID.15701700	1.25	1.07	1.46	0.005	0.02
pCR.predictor.ERNeg.55genes_JAMA.2011.PMID.21558518	1.18	1.01	1.38	0.04	0.08
pCR.predictor.ERPos.39genes_JAMA.2011.PMID.21558518	1.07	0.92	1.26	0.37	0.49
PDCD1_Single_Gene.Single	1.44	1.22	1.70	<0.001	<0.001
Pfefferle2012.LumProg_BCR.2015.PMID.25575446	1.21	1.02	1.45	0.03	0.07
Pfefferle2012.MaSC_BCR.2015.PMID.25575446	0.97	0.83	1.14	0.71	0.80
Pfefferle2012.MatureLum_BCR.2015.PMID.25575446	1.06	0.89	1.27	0.50	0.63
Pfefferle2012.Stroma_BCR.2015.PMID.25575446	0.89	0.76	1.04	0.15	0.25
PGR_Single_Gene.Single	0.54	0.44	0.66	<0.001	<0.001
PI3Ki.Down_CancerCell.2017.PMID.28528867	1.30	1.11	1.53	0.001	0.004
PI3Ki.Up_CancerCell.2017.PMID.28528867	1.00	0.85	1.18	>0.99	> 0.99
PIK3CA.Pathway_Ann.Oncol.2017.PMID.28177460	1.15	0.98	1.36	0.09	0.15
PIK3CAmt.signature_Cancer.Res.2012.PMID.22552288	1.22	1.04	1.44	0.01	0.04
Plasma.cells_Nat.Methods.2015.PMID.25822800	1.34	1.14	1.57	<0.001	0.002
PlasmaCells_CancerImmunolRes.2018.PMID.30266715	1.49	1.26	1.76	<0.001	<0.001
Plasmacytoid.dendritic.cell_CellRep.2017.PMID.28052254	0.89	0.76	1.04	0.14	0.23
PR.Isoform.Ratio.Up.in.PRA.H_JNCI.2017.PMID.28376177	0.84	0.72	0.98	0.03	0.06
PR.Isoform.Ratio.Up.in.PRB.H_JNCI.2017.PMID.28376177	1.30	1.11	1.53	0.001	0.004
Proliferation.Cluster_BMC.Med.Genomics.2011.PMID.21214954	1.37	1.17	1.62	<0.001	0.001
Proliferation.Metagene_Genome.Biol.2013.PMID.23618380	1.38	1.17	1.63	<0.001	0.001
Proliferation.score.PAM50_JCO.2009.PMID.19204204	1.41	1.19	1.66	<0.001	<0.001
ProliferationPathway_CancerImmunolRes.2018.PMID.30266715	1.38	1.17	1.63	<0.001	0.001
Prosigna.Proliferation.18_BMC.Med.Genomics.2015.PMID.26297356	1.33	1.13	1.57	0.001	0.002
Race.LuminalA.MRE.score_BCRT.2015.PMID.26109344	1.03	0.88	1.21	0.69	0.78
Radiation.induced.genes_Radoat.Res.2014.PMID.24527691	1.21	1.00	1.46	0.05	0.09
RB.LOH_BCR.2008.PMID.18782450	1.27	1.08	1.49	0.003	0.01
RB.LOSS_JCI.2007.PMID.17160137	1.37	1.17	1.61	<0.001	0.001
Regulatory.T.cell_CellRep.2017.PMID.28052254	1.27	1.08	1.50	0.005	0.01
Replication.Stress.Down.set_Cell.Rep.2018.PMID.29768207	1.05	0.89	1.23	0.57	0.68
Replication.Stress.Model_Cell.Rep.2018_PMID.29768207.PMID.29768207	1.00	0.85	1.17	>0.99	> 0.99
Replication.Stress.Neg_Cell.Rep.2018_PMID.29768207.PMID.29768207	1.11	0.94	1.30	0.22	0.32
Replication.Stress.Pos_Cell.Rep.2018_PMID.29768207.PMID.29768207	0.94	0.80	1.10	0.42	0.55
Replication.Stress.Up_Set_Cell.Rep.2018_PMID.29768207.PMID.29768207	0.96	0.82	1.12	0.61	0.71
Residual.disease.predictor.ERNeg.54genes_JAMA.2011.PMID.21558518	1.03	0.88	1.21	0.71	0.80

Residual.disease.predictor.ERPos.73genes_JAMA.2011.PMID.21558518	1.04	0.89	1.21	0.65	0.76
Response.Immunotherapy.MCP.TLS.Melanoma_Nature.2020.PMID.31942075	1.58	1.34	1.88	<0.001	<0.001
Response.Immunotherapy.signature_Science.2018.PMID.30309915	1.45	1.23	1.70	<0.001	<0.001
Response.Neo.Chemo_common_CCR.2014.PMID.25047707	1.33	1.13	1.56	0.001	0.002
Response.Neo.Chemo_ERNeg_CCR.2014.PMID.25047707	0.96	0.82	1.11	0.56	0.68
Response.Neo.Chemo_ERPos_CCR.2014.PMID.25047707	1.00	0.85	1.17	0.97	0.99
RHOA.pathway_Ann.Oncol.2017.PMID.28177460	0.90	0.77	1.05	0.19	0.29
Ribosomal.Cluster_BMC.Med.Genomics.2011.PMID.21214954	0.96	0.82	1.12	0.57	0.68
ROR.subtype.PAM50_JCO.2009.PMID.19204204	1.92	1.58	2.36	<0.001	<0.001
ROR.subtype.proliferation.PAM50_JCO.2009.PMID.19204204	1.55	1.31	1.86	<0.001	<0.001
RSS.Score_CCR.2018.PMID.29921729	1.17	1.00	1.37	0.04	0.09
S100A9.A8_BMC.Med.Genomics.2011.PMID.21214954	1.85	1.52	2.26	<0.001	<0.001
Scorr.EMAT1.Correlation_BCR.2020.PMID.32641077	1.14	0.97	1.34	0.11	0.19
Scorr.EMAT2.Correlation_BCR.2020.PMID.32641077	0.74	0.63	0.86	<0.001	0.001
Scorr.EMAT3.Correlation_BCR.2020.PMID.32641077	0.91	0.78	1.08	0.28	0.40
Scorr.EMAT4.Correlation_BCR.2020.PMID.32641077	1.34	1.12	1.61	0.001	0.004
Scorr.IE.Correlation_JCO.2006.PMID.16505416	0.60	0.50	0.71	<0.001	<0.001
Scorr.IIE.Correlation_JCO.2006.PMID.16505416	1.72	1.45	2.05	<0.001	<0.001
Scorr.PAM50.Basal_JCO.2009.PMID.19204204	1.50	1.22	1.86	<0.001	0.001
Scorr.PAM50.Her2_JCO.2009.PMID.19204204	2.05	1.70	2.50	<0.001	<0.001
Scorr.PAM50.LumA_JCO.2009.PMID.19204204	0.51	0.41	0.63	<0.001	<0.001
Scorr.PAM50.LumB_JCO.2009.PMID.19204204	1.18	1.01	1.39	0.04	0.07
Scorr.PAM50.Normal_JCO.2009.PMID.19204204	0.71	0.60	0.84	<0.001	<0.001
Scorr.S329.L_Br.J.Cancer.2008.PMID.18382427	0.99	0.84	1.17	0.90	0.94
Scorr.S329.R_Br.J.Cancer.2008.PMID.18382427	0.99	0.83	1.17	0.88	0.93
Secretoglobin_BMC.Med.Genomics.2011.PMID.21214954	1.15	0.99	1.35	0.08	0.14
Shehata2012.ALDHneg_BCR.2015.PMID.25575446	1.12	0.96	1.30	0.16	0.26
Shehata2012.ALDHpos_BCR.2015.PMID.25575446	1.24	1.04	1.48	0.02	0.04
Shehata2012.Basal_BCR.2015.PMID.25575446	0.89	0.76	1.05	0.17	0.26
Shehata2012.Erbb3neg_BCR.2015.PMID.25575446	0.95	0.82	1.11	0.54	0.66
Shehata2012.LumProg_BCR.2015.PMID.25575446	1.21	1.03	1.43	0.02	0.05
Shehata2012.NCL_BCR.2015.PMID.25575446	1.00	0.84	1.18	0.97	0.99
Shehata2012.Stroma_BCR.2015.PMID.25575446	0.94	0.80	1.10	0.45	0.57
Spike2012.aMaSC_BCR.2015.PMID.25575446	0.89	0.76	1.04	0.15	0.24
Spike2012.fMaSC_BCR.2015.PMID.25575446	0.93	0.80	1.09	0.40	0.53
Spike2012.fStr_BCR.2015.PMID.25575446	0.88	0.75	1.04	0.13	0.21
STAT1_BCR.2008.PMID.19272155	1.40	1.19	1.65	<0.001	<0.001
STAT3.Basal_PNAS.2014.PMID.25139989	1.29	1.10	1.51	0.002	0.007
STAT3.Basal.short_PNAS.2014.PMID.25139989	1.31	1.11	1.55	0.001	0.004
Stroma.FNA.MDACC.1_JCO.2010.PMID.20805453	1.44	1.23	1.70	<0.001	<0.001
Stroma.FNA.MDACC.2_JCO.2010.PMID.20805453	0.90	0.77	1.06	0.21	0.31
Stromal.Central.Fibrotic.Focus_J.Pathol.2017.PMID.27861902	0.87	0.75	1.03	0.10	0.17
Stromal.Down_Nat.Med.2009.PMID.19648928	1.08	0.92	1.27	0.33	0.45
Stromal.Inflammation_J.Pathol.2017.PMID.27861902	1.53	1.29	1.80	<0.001	<0.001
Stromal.Signature_Nat.Med.2008.PMID.18438415	1.22	1.04	1.43	0.01	0.04
Stromal.Up_Nat.Med.2009.PMID.19648928	0.89	0.76	1.05	0.17	0.26
SW480.cancer.cells_Immunity.2013.PMID.24138885	0.94	0.80	1.10	0.43	0.56

T.follicular.helper.cell_CellRep.2017.PMID.28052254	1.23	1.05	1.45	0.01	0.03
Tcell.activation_Nature.2020.PMID.31942077	1.36	1.16	1.61	<0.001	0.001
Tcell.CD8.Effector.vs.naive.2_Science.2016.PMID27789795	1.38	1.17	1.63	<0.001	0.001
Tcell.CD8.Exhausted.vs.antiPDL1.2_Science.2016.PMID27789795	1.48	1.25	1.75	<0.001	<0.001
Tcell.CD8.Exhausted.vs.naive.2_Science.2016.PMID27789795	1.38	1.17	1.63	<0.001	0.001
Tcell.CD8.Memory.vs.naive.1_Science.2016.PMID27789795	1.53	1.29	1.81	<0.001	<0.001
Tcell.cluster_CCR.2014.PMID.24916698	1.46	1.24	1.73	<0.001	<0.001
Tcell.EXH.Anti.PDL1.vs.control.treated.exhausted.CD8.Tcell.Metagene.1.Science.2016.PMID.27789795	1.10	0.94	1.29	0.22	0.32
Tcell.EXH.Effector.CD8.T.cell.at.day.8.p.i.Armstrong.vs.Naive.CD8.Tcell.Metagene.1_Science.2016.PMID.27789795	1.42	1.20	1.68	<0.001	<0.001
Tcell.EXH.Exhausted.CD8.T.cell.vs.Naive.CD8.T.cell.Metagene.1_Science.2016.PMID.27789795	1.26	1.07	1.49	0.005	0.01
Tcell.EXH.Exhausted.CD8.T.cell.vs.Naive.CD8.T.cell.Metagene.3_Science.2016.PMID.27789795	1.42	1.20	1.68	<0.001	<0.001
Tcell.EXH.Memory.CD8.T.cell.a.vs.Naive.CD8.T.cell.Metagene.1_Science.2016.PMID.27789795	1.53	1.29	1.81	<0.001	<0.001
Tcell.EXH.Memory.CD8.T.cell.a.vs.Naive.CD8.T.cell.Metagene.2_Science.2016.PMID.27789795	1.36	1.15	1.61	<0.001	0.001
Tcell.EXH.Memory.CD8.T.cell.a.vs.Naive.CD8.T.cell.Metagene.3.Science_2016.PMID.27789795	1.38	1.17	1.64	<0.001	0.001
Tcell.NK.51gene_Genome.Biol.2013.PMID.23618380	1.41	1.20	1.66	<0.001	<0.001
Tcell.NK.Metagene_Genome.Biol.2013.PMID.23618380	1.40	1.20	1.65	<0.001	<0.001
Tcell.RM_Nat_Med.2018.PMID.29942092	1.40	1.19	1.65	<0.001	<0.001
Tcell.survival.2gene_Nature.2020.PMID.31942077	1.40	1.19	1.66	<0.001	<0.001
Tcells_CancerImmunolRes.2018.PMID.30266715	1.43	1.21	1.68	<0.001	<0.001
Tcells_Immunity.2013.PMID.24138885	1.44	1.23	1.71	<0.001	<0.001
Tcells_TFH_Nat.Methods.2015.PMID.25822800	1.46	1.24	1.72	<0.001	<0.001
Tcells.CD4.memory.activated_Nat.Methods.2015.PMID.25822800	1.53	1.30	1.81	<0.001	<0.001
Tcells.CD4.memory.resting_Nat.Methods.2015.PMID.25822800	1.39	1.18	1.63	<0.001	<0.001
Tcells.CD4.naive_Nat.Methods.2015.PMID.25822800	1.39	1.18	1.63	<0.001	<0.001
Tcells.CD8_Immunity.2013.PMID.24138885	1.02	0.88	1.20	0.76	0.83
Tcells.CD8_Nat.Methods.2015.PMID.25822800	1.44	1.23	1.70	<0.001	<0.001
Tcells.CD8.MCP_Nature.2020.PMID.31942075	1.29	1.10	1.52	0.002	0.006
Tcells.Cytotoxic.MCP_Nature.2020.PMID.31942075	1.42	1.21	1.68	<0.001	<0.001
Tcells.gammadelta_Nat.Methods.2015.PMID.25822800	1.44	1.23	1.70	<0.001	<0.001
Tcells.helper_Immunity.2013.PMID.24138885	1.16	0.99	1.36	0.07	0.13
Tcells.MCP_Nature.2020.PMID.31942077	1.46	1.24	1.72	<0.001	<0.001
Tcells.regulatory.2gene_Nature.2020.PMID.31942077	1.16	0.99	1.36	0.07	0.13
Tcells.Tregs_Nat.Methods.2015.PMID.25822800	1.36	1.16	1.60	<0.001	0.001
TCGA.BRCA.1198_BASAL_JCI.2020.PMID.32573490	0.87	0.74	1.02	0.08	0.14
TCGA.BRCA.1198_Chromogranin_JCI.2020.PMID.32573490	0.99	0.85	1.16	0.93	0.96
TCGA.BRCA.1198_COLLAGEN11A_JCI.2020.PMID.32573490	1.00	0.85	1.17	0.98	> 0.99
TCGA.BRCA.1198_EN1_FDZ9_JCI.2020.PMID.32573490	1.23	1.02	1.47	0.03	0.06
TCGA.BRCA.1198_FGFR4_EGF_JCI.2020.PMID.32573490	1.53	1.29	1.82	<0.001	<0.001
TCGA.BRCA.1198_HISTONES_JCI.2020.PMID.32573490	0.94	0.81	1.10	0.44	0.57
TCGA.BRCA.1198_HOXC11_HOTAIR_SIX1_JCI.2020.PMID.32573490	1.26	1.07	1.48	0.006	0.02
TCGA.BRCA.1198_IL8_CCL_JCI.2020.PMID.32573490	1.35	1.15	1.59	<0.001	0.001
TCGA.BRCA.1198_immune_CD19_JCI.2020.PMID.32573490	1.32	1.12	1.55	0.001	0.003
TCGA.BRCA.1198_immune_CD34_TIE1_JCI.2020.PMID.32573490	0.94	0.81	1.10	0.47	0.60
TCGA.BRCA.1198_immune_CD4_CD53_CD84_BTK_JCI.2020.PMID.32573490	1.44	1.22	1.70	<0.001	<0.001
TCGA.BRCA.1198_immune_CD8_GZMK_JCI.2020.PMID.32573490	1.44	1.22	1.69	<0.001	<0.001
TCGA.BRCA.1198_immune_CTLA4_CXCL_FOXP3_JCI.2020.PMID.32573490	1.49	1.27	1.76	<0.001	<0.001
TCGA.BRCA.1198_immune_FOS_JUN_IL6_JCI.2020.PMID.32573490	0.99	0.84	1.15	0.86	0.91

TCGA.BRCA.1198_immune_GIMAP_IL16_JCI.2020.PMID.32573490	1.26	1.08	1.48	0.005	0.01
TCGA.BRCA.1198_immune_HLA_A_F_JCI.2020.PMID.32573490	1.28	1.09	1.50	0.002	0.007
TCGA.BRCA.1198_immune_HLA_D_JCI.2020.PMID.32573490	1.36	1.16	1.61	<0.001	0.001
TCGA.BRCA.1198_immune_INTERFERON_JCI.2020.PMID.32573490	0.91	0.78	1.06	0.22	0.32
TCGA.BRCA.1198_IMMUNE1_JCI.2020.PMID.32573490	1.47	1.24	1.73	<0.001	<0.001
TCGA.BRCA.1198_LUMINAL_JCI.2020.PMID.32573490	0.58	0.46	0.72	<0.001	<0.001
TCGA.BRCA.1198_MYBL2_APOBEC3B_JCI.2020.PMID.32573490	1.59	1.35	1.89	<0.001	<0.001
TCGA.BRCA.1198_NORMAL_JCI.2020.PMID.32573490	0.92	0.78	1.07	0.29	0.40
TCGA.BRCA.1198_NORMAL2_JCI.2020.PMID.32573490	1.09	0.94	1.28	0.26	0.37
TCGA.BRCA.1198_PDCHA_MANY_JCI.2020.PMID.32573490	0.99	0.85	1.16	0.91	0.95
TCGA.BRCA.1198_S100A7_8_9_JCI.2020.PMID.32573490	1.21	1.01	1.45	0.03	0.07
TCGA.BRCA.1198_TP63_JCI.2020.PMID.32573490	0.97	0.83	1.13	0.69	0.78
TCGA.BRCA.1198.IMMUNOGLOBULIN_JCI.2020.PMID.32573490	1.49	1.27	1.77	<0.001	<0.001
TCGA.CSF1.response_Immunity.2018.PMID.29628290	1.41	1.20	1.67	<0.001	<0.001
TCGA.IFN.score_Immunity.2018.PMID.29628290	0.90	0.77	1.05	0.16	0.26
TCGA.Liexpression.score_Immunity.2018.PMID.29628290	1.40	1.19	1.65	<0.001	<0.001
TCGA.Serum.response.up_Immunity.2018.PMID.29628290	1.24	1.06	1.46	0.008	0.02
TCGA.TFH_Immunity.2018.PMID.29628290	1.36	1.16	1.60	<0.001	0.001
TCGA.Tgd_Immunity.2018.PMID.29628290	0.78	0.67	0.92	0.003	0.009
TCGA.TGFB.score_Immunity.2018.PMID.29628290	1.01	0.87	1.18	0.85	0.91
Tcm_Immunity.2013.PMID.24138885	1.17	1.00	1.36	0.05	0.10
Tem_Immunity.2013.PMID.24138885	0.88	0.75	1.04	0.13	0.21
TFH_Immunity.2013.PMID.24138885	1.36	1.16	1.60	<0.001	0.001
Tgd_Immunity.2013.PMID.24138885	0.78	0.67	0.92	0.003	0.009
Th1_cells_Immunity.2013.PMID.24138885	1.47	1.25	1.74	<0.001	<0.001
Th17_cells_Immunity.2013.PMID.24138885	0.83	0.71	0.97	0.02	0.05
Th2_cells_Immunity.2013.PMID.24138885	1.12	0.96	1.31	0.16	0.26
TLS.9Gene.Signature_Nature.2020.PMID.31942071	1.23	1.05	1.44	0.01	0.03
TLS.CXCL13.SingleGene_Nature.2020.PMID.31942077	1.40	1.20	1.66	<0.001	<0.001
TLS.Hallmark.Gene.Signature_Nature.2020.PMID.31942071	1.39	1.19	1.65	<0.001	<0.001
TLS.Known.Markers_Nature.2020.PMID.31942071	1.39	1.18	1.64	<0.001	<0.001
TLS.Structure.12chemokine_FrontImmunol.2017.PMID.28713385	1.45	1.23	1.71	<0.001	<0.001
TLS.tumors.wTLS.and.CD8.vs.CD8alone_Nature.2020.PMID.31942071	1.41	1.20	1.66	<0.001	<0.001
TNBC.good.prognosis.TNBC.230genes_BCR.2011.PMID.21978456	1.11	0.94	1.30	0.21	0.31
TNBC.good.prognosis.TNBC.26genes_BCR.2011.PMID.21978456	1.22	1.04	1.43	0.01	0.04
TNBC.metastasis.free.survival_PLoS.One.2013.PMID.24349199	1.31	1.11	1.53	0.001	0.004
TNBC.poor.prognosis.TNBC.26genes_BCR.2011.PMID.21978456	1.06	0.90	1.23	0.49	0.63
Translation.Pathway_CancerImmunolRes.2018.PMID.30266715	0.96	0.82	1.12	0.60	0.71
Tumour.hypoxia.causes.DNA.hypermethylation_Nature.2016.PMID.27533040	1.03	0.88	1.21	0.72	0.80
Type.1.T.helper.cell_CellRep.2017.PMID.28052254	1.36	1.15	1.60	<0.001	0.001
Type.17.T.helper.cell_CellRep.2017.PMID.28052254	1.46	1.24	1.72	<0.001	<0.001
Type.2.T.helper.cell_CellRep.2017.PMID.28052254	1.19	1.01	1.39	0.03	0.07
Up.Basal.High_Nat.Cell.Biol.2014.PMID.25173976	0.86	0.73	1.01	0.06	0.11
Up.Proliferation_Nat.Cell.Biol.2014.PMID.25173976	1.36	1.16	1.61	<0.001	0.001
Upregulated.by.oncogenic.NRAS.basal_Cell.Rep.2016.PMID.26166574	0.86	0.73	1.00	0.05	0.10
Upregulated.upon.NRAS.repression.basal_Cell.Rep.2017.PMID.26166574	0.74	0.63	0.87	<0.001	0.001
Vascular.Content_Clin.Exp.Metastasis.2014.PMID.23975155	1.03	0.87	1.21	0.76	0.83

VEGF.13genes_BMC.Med.2009.PMID.19291283	0.96	0.81	1.13	0.61	0.71
Wirapati.Proliferation_BCR.2008.PMID.18662380	1.36	1.16	1.60	<0.001	0.001
Wound.Signature_CCR.2009.PMID.19887484	0.92	0.78	1.07	0.27	0.39
X11q13.Amplicon_BMC.Med.Genomics.2011.PMID.21214954	0.92	0.78	1.07	0.29	0.41
X12qMDM4.BMC.Med.Genomics.2011.PMID.21214954	1.04	0.88	1.21	0.66	0.76
X13q14.Amplicon_BMC.Med.Genomics.2011.PMID.21214954	0.95	0.81	1.11	0.50	0.63
X15q25.Amplicon_BMC.Med.Genomics.2011.PMID.21214954	1.15	0.98	1.35	0.09	0.15
X16.13.Amplicon_BMC.Med.Genomics.2011.PMID.21214954	0.89	0.76	1.05	0.17	0.26
X16q23.Amplicon_BMC.Med.Genomics.2011.PMID.21214954	1.10	0.94	1.29	0.23	0.33
X17PP13.Amplicon_BMC.Med.Genomics.2011.PMID.21214954	1.15	0.99	1.35	0.08	0.14
X17q25x.BMC.Med.Genomics.2011.PMID.21214954	1.07	0.92	1.25	0.41	0.53
X19p13.Amplicon_BMC.Med.Genomics.2011.PMID.21214954	0.94	0.80	1.10	0.43	0.56
X1p36.Amplicon_BMC.Med.Genomics.2011.PMID.21214954	1.04	0.88	1.23	0.64	0.74
X3p21.Amplicon_BMC.Med.Genomics.2011.PMID.21214954	0.84	0.72	0.99	0.03	0.07
X4p16.Amplicon_BMC.Med.Genomics.2011.PMID.21214954	0.93	0.80	1.09	0.39	0.52
X5Q_BCRT.2012.PMID.22048815	0.84	0.71	0.99	0.04	0.08
X8p.Amplicon_BMC.Med.Genomics.2011.PMID.21214954	1.15	0.98	1.34	0.08	0.14
X8p22.Amplicon_BMC.Med.Genomics.2011.PMID.21214954	0.81	0.69	0.94	0.008	0.02
XBP1.Signature_Nature.2014.PMID.24670641	0.97	0.83	1.13	0.71	0.80

CALGB 40601					
Signature	OR	95% CI		*P*	adjusted *P*
Activate.Endothelium_Clin.Exp.Metastasis.2014.PMID.23975155	1.30	1.01	1.70	0.05	0.17
Activated.B.cell_CellRep.2017.PMID.28052254	1.25	0.94	1.67	0.12	0.26
Activated.Blood.Neutrophil.Signature_Nat.Cell.Biol.2019.PMID.31263265	0.77	0.57	1.05	0.10	0.24
Activated.Cancer.Cell.Signature_Nat.Cell.Biol.2019.PMID.31263265	0.79	0.59	1.04	0.09	0.23
Activated.CD4.T.cell_CellRep.2017.PMID.28052254	1.42	1.09	1.87	0.01	0.07
Activated.CD8.T.cell_CellRep.2017.PMID.28052254	1.26	0.95	1.69	0.11	0.24
Activated.dendritic.cell_CellRep.2017.PMID.28052254	1.07	0.81	1.40	0.63	0.75
Activated.Lung.MSC.Signature_Nat.Cell.Biol.2019.PMID.31263265	0.73	0.53	0.99	0.04	0.16
Activated.Lung.Neutrophil.Signature_Nat.Cell.Biol.2019.PMID.31263265	1.33	1.03	1.75	0.03	0.14
aDC_Immunity.2013_PMID.24138885.PMID.24138885	1.37	1.03	1.86	0.03	0.14
ADM.S100A10.A110NDGR1.Cluster_BMC.Med.Genomics.2011.PMID.21214954	0.99	0.74	1.32	0.93	0.96
African.and.European.Ancestry.TCGA.Negative_JAMA.Oncol.2017.PMID.28472234	0.81	0.64	1.02	0.08	0.21
African.and.European.Ancestry.TCGA.Positive_JAMA.Oncol.2017.PMID.28472234	0.92	0.70	1.20	0.55	0.68
Age.associated.signature_Genome.Biol.2015.PMID.26343147	0.83	0.62	1.10	0.20	0.34
aMaSC_BCR.2010.PMID.20346151	0.77	0.60	0.97	0.03	0.14
aMaSC.HsEnriched_BCR.2015.PMID.25575446	0.84	0.65	1.07	0.16	0.30
aMaSC.HsEnriched.Refined1_BCR.2015.PMID.25575446	0.88	0.70	1.10	0.27	0.42
aMaSC.Lim09_BCR.2015.PMID.25575446	0.92	0.70	1.21	0.56	0.69
aMaSC.Prat_BCR.2015.PMID.25575446	0.96	0.74	1.24	0.75	0.85
aMaSC.Shehata_BCR.2015.PMID.25575446	0.71	0.53	0.94	0.02	0.10
aMaSC.Signature_Cell.Stem.Cell.2012.PMID.22305568	0.84	0.64	1.09	0.20	0.34
AMPH.EPIREGULIN.Cluster_BMC.Med.Genomics.2011.PMID.21214954	0.86	0.68	1.07	0.17	0.31
Amplification.50_Genome.Biol.2014.PMID.25164602	1.01	0.77	1.33	0.93	0.96
Amplification.50.better.than._Genome.Biol.2015.PMID.25164602	0.80	0.60	1.04	0.11	0.24
Apocrine.Features_J.Pathol.2017.PMID.27861902	1.45	1.08	1.98	0.02	0.09
aStr.HsEnriched_BCR.2015.PMID.25575446	0.78	0.61	0.98	0.04	0.15
aStr.HsEnriched.Refined1_BCR.2015.PMID.25575446	0.69	0.54	0.88	0.003	0.04
aStr.HsEnriched.Refined2_BCR.2015.PMID.25575446	0.81	0.63	1.01	0.07	0.20
aStr.Lim09_BCR.2015.PMID.25575446	0.76	0.59	0.96	0.02	0.12
aStr.Prat_BCR.2015.PMID.25575446	0.79	0.62	1.01	0.06	0.19
aStr.Shehata_BCR.2015.PMID.25575446	0.80	0.62	1.02	0.07	0.20
BASAL.Cluster_BMC.Med.Genomics.2011.PMID.21214954	0.85	0.66	1.08	0.19	0.33
Bcell.cluster_CCR.2014.PMID.24916698	1.35	1.01	1.80	0.04	0.16
Bcell.IL10.MINUS_Immunol.2014.PMID.25080484	1.26	0.95	1.68	0.11	0.25
Bcell.IL10.PLUS_Immunol.2014.PMID.25080484	1.09	0.83	1.45	0.54	0.67

Bcell.lineage.MCP_Nature.2020.PMID.31942077	1.24	0.93	1.66	0.15	0.28
Bcell.Plasma.52gene_Genome.Biol.2013.PMID.23618380	2.09	1.50	2.99	<0.001	0.002
Bcell.Plasma.Metagene_Genome.Biol.2013.PMID.23618380	1.83	1.33	2.58	<0.001	0.008
Bcell.Tcell.Cooperation_Cell.2019.PMID.31730857	1.19	0.89	1.59	0.24	0.39
Bcells_CancerImmunolRes.2018.PMID.30266715	1.25	0.94	1.68	0.12	0.26
Bcells_Immunity.2013.PMID.24138885	1.15	0.87	1.52	0.33	0.48
Bcells.Centroblast_JCO.2015.PMID.25800755	1.50	1.17	1.95	0.002	0.03
Bcells.Centrocyte_JCO.2015.PMID.25800755	1.49	1.11	2.02	0.009	0.07
Bcells.Memory_JCO.2015.PMID.25800755	0.99	0.76	1.30	0.96	0.97
Bcells.memory_Nat.Methods.2015.PMID.25822800	1.21	0.91	1.63	0.19	0.33
Bcells.Naive_JCO.2015.PMID.25800755	0.97	0.74	1.27	0.81	0.88
Bcells.naive_Nat.Methods.2015.PMID.25822800	1.12	0.84	1.50	0.43	0.58
Bcells.Plasmablast_JCO.2015.PMID.25800755	1.53	1.14	2.08	0.006	0.05
Blood.vessels_Immunity.2013.PMID.24138885	0.81	0.64	1.02	0.07	0.21
bMYB.Signature_Oncogene.2009.PMID.19043454	1.39	1.09	1.79	0.009	0.07
C3TAG.Responding_CCR.2013.PMID.23780888	0.95	0.77	1.17	0.65	0.76
C3TAG.Untreated_CCR.2013.PMID.23780888	1.25	1.00	1.57	0.05	0.18
CD103.Negative_Cancer.Cell.2014.PMID.25446897	0.90	0.70	1.16	0.43	0.58
CD103.Positive_Cancer.Cell.2014.PMID.25446897	0.99	0.75	1.32	0.95	0.97
CD103.Ratio_Cancer.Cell.2014.PMID.25446897	1.04	0.81	1.32	0.78	0.86
CD274_Single_Gene.Single	1.37	1.05	1.81	0.02	0.12
CD34.CD36.Cluster_BMC.Med.Genomics.PMID.21214954	0.85	0.69	1.04	0.12	0.26
CD44.downregulated.genes_Cancer.Cell.2007.PMID.17349583	1.84	1.38	2.52	<0.001	0.002
CD44.upregulated.genes_Cancer.Cell.2007.PMID.17349583	1.14	0.84	1.55	0.41	0.57
CD56bright.natural.killer.cell_CellRep.2017.PMID.28052254	1.12	0.85	1.50	0.42	0.57
CD56dim.natural.killer.cell_CellRep.2017.PMID.28052254	1.16	0.88	1.52	0.29	0.44
CD68.cluster_CCR.2014.PMID.24916698	0.94	0.73	1.20	0.60	0.72
CD8.cluster_CCR.2014.PMID.24916698	1.28	0.98	1.71	0.08	0.21
CDKN2A_Single_Gene.Single	0.74	0.56	0.98	0.04	0.15
Central.memory.CD4.T.cell_CellRep.2017.PMID.28052254	0.80	0.60	1.04	0.10	0.24
Central.memory.CD8.T.cell_CellRep.2017.PMID.28052254	0.78	0.60	1.02	0.07	0.20
CES.Score_CCR.2017.PMID.27903675	0.45	0.30	0.66	<0.001	0.002
Chromogramin_BMC.Med.Genomics.2011.PMID.21214954	0.87	0.67	1.14	0.31	0.46
CIN70_Nat.Genet.2006.PMID.16921376	1.42	1.13	1.82	0.004	0.04
Claudin.High_Genome.Biol.2007.PMID.17493263	1.03	0.78	1.35	0.83	0.89
Claudin.Low_Genome.Biol.2007.PMID.17493263	1.33	1.04	1.74	0.03	0.12
Claudin.Low.29_Cancer.Res.2009.PMID.19435916	1.32	1.05	1.69	0.02	0.12
cMYB.Signature_PLoS.One.2010.PMID.20949095	0.92	0.68	1.26	0.62	0.74
CORE.Bcell.signature.Garber_Cell.Mol.Gastroenterol.Hepatol.2017.PMID.28508029	1.03	0.78	1.35	0.85	0.91
CTLA4_Single_Gene.Single	1.35	1.03	1.79	0.03	0.14
Cytolytic.activity_Cell.2015.PMID.25594174	1.21	0.90	1.64	0.22	0.36
Cytotoxic.cells_Immunity.2013.PMID.24138885	1.23	0.93	1.65	0.15	0.28
Day7.Downregulated_Nat.Cell.Biol.2014.PMID.25173976	0.81	0.62	1.05	0.11	0.25
Day7.Upregulated_Nat.Cell.Biol.2014.PMID.25173976	1.52	1.16	2.01	0.003	0.04
DC_Immunity.2013.PMID.24138885	1.06	0.84	1.34	0.62	0.74
DCIS.HGF.down_BCR.2013.PMID.24025166	0.80	0.60	1.05	0.11	0.25
DCIS.HGF.up_BCR.2014.PMID.24025166	1.07	0.81	1.42	0.62	0.74

Delection.50_Genome.Biol.2016.PMID.25164602	0.86	0.66	1.11	0.25	0.40
Delection.50.better.than_Genome.Biol.2017.PMID.25164602	0.97	0.72	1.29	0.82	0.89
Dendritic.cells.activated_Nat.Methods.2015.PMID.25822800	1.25	0.95	1.66	0.11	0.24
Dendritic.cells.resting_Nat.Methods.2015.PMID.25822800	1.04	0.80	1.34	0.78	0.86
Down.Basal.High_Nat.Cell.Biol.2014.PMID.25173976	0.88	0.69	1.12	0.31	0.46
Down.CLOW.High_Nat.Cell.Biol.2014.PMID.25173976	0.77	0.60	0.97	0.03	0.14
Downregulated.upon.NRAS.repression.basal_Cell.Rep.2015.PMID.26166574	0.97	0.73	1.29	0.83	0.89
Ductal.Carcinoma.In.Situ_J.Pathol.2017.PMID.27861902	1.12	0.86	1.48	0.41	0.56
Duke.Module01.acidosis_PNASUSA.2010.PMID.20335537	0.82	0.65	1.04	0.10	0.24
Duke.Module02.akt_PNASUSA.2010.PMID.20335537	0.75	0.56	0.99	0.05	0.17
Duke.Module03.betacatenin_PNASUSA.2010.PMID.20335537	1.27	0.97	1.66	0.08	0.21
Duke.Module04.E2F1_PNASUSA.2010.PMID.20335537	1.38	1.05	1.83	0.02	0.12
Duke.Module05.EGFR_PNASUSA.2010.PMID.20335537	1.23	0.98	1.56	0.08	0.21
Duke.Module06.ER_PNASUSA.2010.PMID.20335537	1.23	0.90	1.70	0.19	0.33
Duke.Module07.glucosedepletion_PNASUSA.2010.PMID.20335537	1.22	0.91	1.63	0.19	0.33
Duke.Module08.HER2_PNASUSA.2010.PMID.20335537	2.64	1.88	3.85	<0.001	<0.001
Duke.Module09.hypoxia_PNASUSA.2010.PMID.20335537	1.24	0.95	1.64	0.11	0.25
Duke.Module10.IFNA_PNASUSA.2010.PMID.20335537	1.08	0.83	1.41	0.58	0.71
Duke.Module11.IFNG_PNASUSA.2010.PMID.20335537	1.13	0.87	1.49	0.36	0.51
Duke.Module12.lacticacidosis_PNASUSA.2010.PMID.20335537	1.17	0.88	1.56	0.28	0.43
Duke.Module13.myc_PNASUSA.2010.PMID.20335537	1.29	1.00	1.69	0.06	0.19
Duke.Module14.p53_PNASUSA.2010.PMID.20335537	0.74	0.53	1.02	0.07	0.20
Duke.Module15.p63_PNASUSA.2010.PMID.20335537	1.48	1.13	1.95	0.005	0.05
Duke.Module16.pi3k_PNASUSA.2010.PMID.20335537	1.21	0.93	1.58	0.15	0.29
Duke.Module17.PR_PNASUSA.2010.PMID.20335537	0.62	0.43	0.88	0.01	0.07
Duke.Module18.ras_PNASUSA.2010.PMID.20335537	1.54	1.12	2.13	0.008	0.07
Duke.Module19.src_PNASUSA.2010.PMID.20335537	1.09	0.82	1.46	0.54	0.67
Duke.Module20.STAT3_PNASUSA.2010.PMID.20335537	1.22	0.91	1.65	0.19	0.33
Duke.Module21.TGFB_PNASUSA.2010.PMID.20335537	1.27	0.99	1.63	0.06	0.19
Duke.Module22.TNFA_PNASUSA.2010.PMID.20335537	1.26	0.97	1.67	0.09	0.22
Durvalumab.signature_CCR.2018.PMID.29716923	1.36	1.05	1.79	0.02	0.12
Early.IRS.1_PLoS.One.2016.PMID.26991655	1.11	0.87	1.43	0.41	0.56
Early.IRS.2_PLoS.One.2016.PMID.26991655	1.06	0.80	1.42	0.69	0.80
Early.Relapse.ERPos.33genes_JAMA.2011.PMID.21558518	1.03	0.79	1.34	0.83	0.89
Early.Response.ERNeg.27genes_JAMA.2011.PMID.21558518	0.95	0.72	1.25	0.72	0.82
Effector.memeory.CD4.T.cell_CellRep.2017.PMID.28052254	0.98	0.73	1.32	0.91	0.94
Effector.memeory.CD8.T.cell_CellRep.2017.PMID.28052254	1.10	0.84	1.45	0.49	0.63
EGFR_Single_Gene.Single	0.86	0.64	1.14	0.29	0.44
EMT.down.Taube_PNAS.2010.PMID.20713713	1.47	1.16	1.90	0.002	0.03
EMT.down.Weingberg_PNAS.2010.PMID.20713713	1.42	1.11	1.85	0.007	0.06
EMT.up.Taube_PNAS.2010.PMID.20713713	0.76	0.59	0.98	0.04	0.15
EMT.up.Weinberg_PNAS.2010.PMID.20713713	0.80	0.61	1.04	0.10	0.24
Endothelial.cells.MCP_Nature.2020..PMID.31942077	0.82	0.65	1.04	0.11	0.24
Endothelial.Normal_Angiogenesis.2014.PMID.24257808	1.32	1.03	1.70	0.03	0.14
Endothelial.Tumor_Angiogenesis.2014.PMID.24257808	1.00	0.75	1.35	0.98	0.98
Eosinophil_CellRep.2017.PMID.28052254	0.99	0.76	1.29	0.94	0.96
Eosinophils_Immunity.2013.PMID.24138885	1.11	0.86	1.45	0.42	0.58

Eosinophils_Nat.Methods.2015.PMID.25822800	0.93	0.72	1.20	0.59	0.72
Epithelial.Tubule.Formation_J.Pathol.2017.PMID.27861902	0.60	0.42	0.83	0.003	0.04
ERBB2_Single_Gene.Single	2.06	1.57	2.77	<0.001	<0.001
ERBB3_Single_Gene.Single	1.18	0.92	1.53	0.20	0.34
ESR1_Single_Gene.Single	0.36	0.23	0.55	<0.001	<0.001
ESTIMATE.Immune_Nat.Commun.2013.PMID.24113773	1.13	0.85	1.50	0.40	0.55
ESTIMATE.Stromal_Nat.Commun.2013.PMID.24113773	0.83	0.64	1.08	0.17	0.31
Euclidean.Distance.CLOW_BCR.2010.PMID.20813035	1.39	1.08	1.80	0.01	0.08
EXTENDED.Bcell.signature.Garber_Cell.Mol.Gastroenterol.Hepatol.2017.PMID.28508029	0.91	0.68	1.21	0.52	0.66
FGFR4_Single_Gene.Single	1.80	1.37	2.41	<0.001	0.002
FGFR4.Induced_JCI.2020.PMID.32573490	2.03	1.45	2.91	<0.001	0.002
FGFR4.Repressed_JCI.2020.PMID.32573490	0.70	0.50	0.96	0.03	0.14
Fibrinogen.Cluster_BMC.Med.Genomics.2011.PMID.21214954	0.76	0.58	0.98	0.04	0.15
Fibroblast.Cluster_BMC.Med.Genomics.2011.PMID.21214954	0.81	0.63	1.05	0.12	0.25
Fibroblasts.MCP_Nature.2020.PMID.31942077	0.80	0.61	1.05	0.11	0.24
Fibromatosis_Lab.Invest.2008.PMID.18414401	0.79	0.60	1.03	0.08	0.22
fMaSC.Metab_CellRep.2018.PMID.30089273	0.75	0.57	0.97	0.03	0.14
fMaSC.Metab8_CellRep.2018.PMID.30089273	1.53	1.16	2.06	0.003	0.04
fMaSC.refined1_BCR.2015.PMID.25575446	0.99	0.76	1.28	0.92	0.95
fMasC.Signature_Cell.Stem.Cell.2012.PMID.22305568	0.83	0.64	1.06	0.14	0.28
fMaSC.Signature_CellRep.2018.PMID.30089273	1.43	1.06	1.96	0.02	0.12
FOS.JUN_Cluster_BMC.Med.Genomics.2011.PMID.21214954	0.77	0.57	1.04	0.09	0.22
FOXC1.Hair.Follicles.P30C.LO.vs.WT.Negative_Science.2016.PMID.26912704	0.77	0.58	1.01	0.07	0.20
FOXC1.Hair.Follicles.P30C.LO.vs.WT.Positive_Science.2016.PMID.26912704	0.90	0.68	1.19	0.47	0.62
fSTR.Signature_Cell.Stem.Cell.2012.PMID.22305568	0.68	0.54	0.85	0.001	0.02
Gamma.delta.T.cell_CellRep.2017.PMID.28052254	1.07	0.81	1.41	0.64	0.76
GATA3.induced.genes_JCO.2006.PMID.16505416	1.72	1.32	2.29	<0.001	0.003
GATA3.induced.genes_Oncogene.2004.PMID.15361840	1.49	1.16	1.96	0.003	0.04
GDF11.TGFBR3_Nat.Cell.Biol.2014.PMID.24658685	1.07	0.80	1.43	0.66	0.77
Glycolysis_BMC.Med.2009.PMID.19291283	2.11	1.52	3.01	<0.001	0.001
GO.DOWN.with.SOX10.OE_Cell.Rep.2015.PMID.26365194	1.26	0.93	1.73	0.14	0.28
GO.UP.with.SOX10.OE_Cell.Rep.2015.PMID.26365194	0.78	0.59	1.02	0.07	0.21
GSEA_BIOCARTA_ALK_PATHWAY.PMID.16199517	0.91	0.71	1.18	0.49	0.63
GSEA_BIOCARTA.AKT.PATHWAY.PMID.16199517	1.21	0.91	1.62	0.19	0.33
GSEA_BIOCARTA.BRCA.ATR.PATHWAY.ATRBRCA.PMID.16199517	1.11	0.84	1.47	0.48	0.62
GSEA_BIOCARTA.CASPASE.PATHWAY.PMID.16199517	1.38	1.03	1.87	0.03	0.14
GSEA_BIOCARTA.CTLA4.PATHWAY.PMID.16199517	1.25	0.94	1.67	0.13	0.27
GSEA_BIOCARTA.IGF1R.PATHWAY.PMID.16199517	0.83	0.64	1.07	0.16	0.30
GSEA_BIOCARTA.MTOR.PATHWAY.PMID.16199517	1.23	0.95	1.61	0.12	0.26
GSEA_BIOCARTA.PTEN.PATHWAY.PMID.16199517	1.11	0.86	1.45	0.41	0.57
GSEA_BIOCARTA.RAS.PATHWAY.PMID.16199517	1.09	0.83	1.44	0.52	0.66
GSEA_BIOCARTA.RB.PATHWAY.PMID.16199517	1.71	1.28	2.33	<0.001	0.01
GSEA_BIOCARTA.VEGF.PATHWAY.PMID.16199517	1.21	0.90	1.64	0.21	0.35
GSEA_HALLMARK.MYC.TARGETS.V1.PMID.16199517	0.98	0.75	1.29	0.89	0.93
GSEA_HELLER.HDAC.TARGETS.DOWN.PMID.16199517	1.32	0.99	1.77	0.06	0.19
GSEA_NELSON.RESPONSE.TO.ANDROGEN.UP.PMID.16199517	1.47	1.11	1.99	0.009	0.07
GSEA_REACTOME.PD1.SIGNALING.PMID.16199517	1.25	0.94	1.67	0.13	0.26

GSEA_REACTOME.PI3K.CASCADE.PMID.16199517	0.85	0.64	1.11	0.23	0.37
GSEA_RETINOL.METABOLISM.KEGG.PMID.16199517	0.93	0.75	1.15	0.52	0.66
GSEA.GP1_Proliferation.DNA.repair..PUJANA.CHEK2.PCC.NETWORK.PMID.25109877	1.37	1.05	1.81	0.02	0.12
GSEA.GP1_Proliferation.DNA.repair.REACTOME.CELL.CYCLE.MITOTIC.PMID.25109877	1.33	1.02	1.75	0.04	0.15
GSEA.GP10_Fatty.acid.oxidation.CARBOXYLIC.ACID.METABOLIC.PROCESS.PMID.25109877	0.83	0.64	1.06	0.15	0.28
GSEA.GP11_Immune.IFN.PerouLab.PMID.25109877	1.10	0.85	1.43	0.48	0.62
GSEA.GP12_Hypoxia.glycolosis.SEMENZA.HIF1.TARGETS.PMID.25109877	1.20	0.93	1.56	0.16	0.30
GSEA.GP13_Neural.signailng.MODULE100.PMID.25109877	0.76	0.60	0.96	0.02	0.12
GSEA.GP13_Neural.signailng.NERVOUS.SYSTEM.DEVELOPMENT.PMID.25109877	0.63	0.47	0.82	0.001	0.01
GSEA.GP14_Plasma.membrane.cell.cell.signaling.MORF.CNTN1.PMID.25109877	0.97	0.74	1.27	0.82	0.89
GSEA.GP15_EGF.signaling.NAGASHIMA.EGF.SIGNALING.UP.PMID.25109877	0.93	0.68	1.25	0.62	0.75
GSEA.GP16_Protein.kinase.signaling.MAPKs.INTRACELLULAR.SIGNALING.CASCADE.PMID.25109877	1.31	0.99	1.74	0.06	0.19
GSEA.GP16_Protein.kinase.signaling.MAPKs.REGULATION.OF.KINASE.ACTIVITY.PMID.25109877	1.21	0.92	1.60	0.18	0.33
GSEA.GP17_Basal.signaling.SMID.BREAST.CANCER.BASAL.UP.PMID.25109877	1.13	0.81	1.58	0.47	0.62
GSEA.GP18_Vesicle.EPR.MEMBRANE.COAT.PMID.25109877	1.39	1.04	1.89	0.03	0.13
GSEA.GP19_1Q.amplicon.PerouLab.PMID.25109877	0.94	0.73	1.20	0.60	0.73
GSEA.GP2_Immune.Tcell.Bcell.KEGG.HEMATOPOIETIC.CELL.LINEAGE.PMID.25109877	1.10	0.84	1.45	0.48	0.62
GSEA.GP2_Immune.Tcell.Bcell.PerouLab.PMID.25109877	1.16	0.87	1.56	0.31	0.46
GSEA.GP20_TAL1.Leukemia.erythropoiesis.GNF2.TAL1.PMID.25109877	0.76	0.57	1.00	0.05	0.17
GSEA.GP21_Anti.apoptosis.DNA.stability.MORF.BCL2.PMID.25109877	0.77	0.57	1.04	0.09	0.23
GSEA.GP21_Anti.apoptosis.DNA.stability.MORF.MT4.PMID.25109877	1.04	0.79	1.38	0.76	0.85
GSEA.GP21_Anti.apoptosis.DNA.stability.MORF.STK17A.PMID.25109877	0.79	0.59	1.06	0.12	0.26
GSEA.GP22_16Q22.24.amplicon.PerouLab.PMID.25109877	1.28	0.97	1.70	0.08	0.21
GSEA.GP3_Tumo.suppressing.miRNA.targets.GTTTGTT.MIR.495.PMID.25109877	1.04	0.82	1.32	0.76	0.85
GSEA.GP3_Tumor.suppressing.miRNA.targets.DACOSTA.UV.RESPONSE.VIA.ERCC3.DN.PMID.25109877	1.08	0.86	1.36	0.50	0.64
GSEA.GP3_Tumor.suppressing.miRNA.targets.TGCTTTG.MIR.330.PMID.25109877	0.96	0.76	1.23	0.77	0.86
GSEA.GP4_MES.ECM.PerouLab.PMID.25109877	0.82	0.63	1.07	0.14	0.28
GSEA.GP5_MYC.targets.TERT.PerouLab.PMID.25109877	1.28	0.98	1.68	0.07	0.20
GSEA.GP6_Squamous.differentiation.development.RICKMAN.TUMOR.DIFFERENTIATED.WELL.VS.POORLY.DN.PMID.25109877	1.41	1.05	1.90	0.02	0.12
GSEA.GP7_Estrogen.signaling.SMID.BREAST.CANCER.BASAL.DN.PMID.25109877	0.87	0.62	1.22	0.42	0.57
GSEA.GP8_FOXO.stemness.MORF.PTPRB.PMID.25109877	0.72	0.53	0.96	0.03	0.12
GSEA.GP8_FOXO.stemness.TTGTTT.VSFOXO4.01.PMID.25109877	0.78	0.59	1.03	0.08	0.21
GSEA.GP9_Cell.cell.adhesion.PerouLab.PMID.25109877	1.48	1.16	1.93	0.002	0.03
HCK_BCR.2008.PMID.19272155	0.99	0.76	1.29	0.94	0.96
HER1.Cluster1_BMC.Genomics.2007.PMID.17663798	1.41	1.05	1.92	0.03	0.12
HER1.Cluster2_BMC.Genomics.2007.PMID.17663798	1.36	1.03	1.81	0.03	0.14
HER1.Cluster3_BMC.Genomics.2007.PMID.17663798	1.37	1.03	1.83	0.03	0.14
HER2.Amplicon.PerouLab_BMC.Med.Genomic.2011.PMID.21214954	1.75	1.31	2.38	<0.001	0.007
Histological.Grade_J.Pathol.2017.PMID.27861902	1.53	1.20	1.99	0.001	0.02
HouseKeeping_Genome.Biol.2004.PMID.15287981	1.15	0.86	1.53	0.35	0.50
iDC.Median_Immunity.2013.PMID.24138885	1.25	0.95	1.66	0.12	0.25
IFN.Cluster_BMC.Med.Genomics.2011.PMID.21214954	0.98	0.75	1.27	0.88	0.93
IgG_BCR.2008.PMID.19272155	2.04	1.46	2.93	<0.001	0.002
IGG.Cluster_BMC.Med.Genomics.2011.PMID.21214954	1.61	1.20	2.18	0.002	0.03
Immature..B.cell_CellRep.2017.PMID.28052254	1.15	0.87	1.53	0.32	0.47
Immature.dendritic.cell_CellRep.2017.PMID.28052254	1.23	0.94	1.62	0.13	0.27
ImmLandscape_Macro.mono.CSF1.core.response_CCR.2009.PMID.29628290	1.11	0.84	1.46	0.46	0.61

ImmLandscape_Wound.Healing_Immunity.2018.PMID.29628290	1.24	0.95	1.64	0.12	0.26
ImmLandscape.IFN3_Plos.One.2014.PMID.24516633	0.96	0.74	1.24	0.76	0.85
ImmLandscape.IFNG5_Plos.One.2014.PMID.24516633	1.37	1.02	1.84	0.04	0.15
ImmLandscape.lymphocyte.Infil.T.B.PMID.18592372	1.25	0.94	1.66	0.13	0.26
Immune.Hot.CD8.vs.Cold_Nature.2020.PMID.31942071	1.17	0.88	1.56	0.29	0.44
Immune.Perez.14_JCO.2015.PMID.25605861	1.00	0.78	1.30	0.98	0.98
Immune.Perez.87_JCO.2015.PMID.25605861	1.27	0.95	1.70	0.11	0.24
Immune.Suppression_JCI.Insight.2016.PMID.27699256	1.33	1.01	1.77	0.04	0.17
ImmuneActive_Cell.2019.PMID.31730857	1.32	1.00	1.75	0.05	0.18
Immunosuppression.PMID.31942077	0.89	0.67	1.18	0.43	0.58
IMS.Score_CCR.2018.PMID.29921729	1.01	0.77	1.33	0.94	0.96
Induced.in.Bcells_PNAS.2013.PMID.23382184	1.20	0.91	1.60	0.21	0.35
Induced.in.DC_PNAS.2013.PMID.23382184	1.40	1.05	1.88	0.02	0.12
Induced.in.GN_PNAS.2013.PMID.23382184	1.04	0.79	1.36	0.79	0.87
Induced.in.HSC_PNAS.2013.PMID.23382184	1.31	0.97	1.79	0.08	0.21
Induced.in.MOs_PNAS.2013.PMID.23382184	0.97	0.74	1.26	0.80	0.88
Induced.in.NKcells_PNAS.2013.PMID.23382184	1.10	0.85	1.44	0.48	0.62
Induced.in.Tcells_PNAS.2013.PMID.23382184	1.02	0.77	1.35	0.90	0.94
Inflammatory.breast.cancer.491genes_CCR.2013.PMID.23396049	1.31	0.97	1.77	0.08	0.21
Inflammatory.breast.cancer.79genes_CCR.2013.PMID.23396049	1.72	1.29	2.33	<0.001	0.008
Inflammatory.breast.cancer.expressed.noIBC_79genes_CCR.2013.PMID.23396049	0.96	0.73	1.27	0.80	0.88
Inflammatory.breast.cancer.expressed.noIBC.491genes_CCR.2013.PMID.23396049	0.86	0.64	1.15	0.32	0.46
Influenza.11genes.Metasignature_Immunity.2015.PMID.26682989	1.10	0.85	1.42	0.47	0.62
Interferon_BCR.2008.PMID.19272155	0.93	0.72	1.20	0.59	0.72
Interferon.Pathway_CancerImmunolRes.2018.PMID.30266715	1.03	0.79	1.33	0.83	0.89
JUND.KRT5_Nat.Cell.Biol.2014.PMID.24658685	0.66	0.49	0.89	0.007	0.06
Keller2012.CD10.Adam_BCR.2015.PMID.25575446	1.13	0.88	1.47	0.34	0.48
KRAS.amplicon_Genome.Biology.2007.PMID.17493263	1.28	0.96	1.72	0.10	0.24
Late.IRS.1_PLoS.One.2016.PMID.26991655	1.34	1.05	1.73	0.02	0.12
Late.IRS.2_PLoS.One.2016.PMID.26991655	0.87	0.65	1.14	0.31	0.46
LCK_BCR.2008.PMID.19272155	1.19	0.90	1.58	0.23	0.38
Lim2009.LumProg.Adam_BCR.2015.PMID.25575446	1.41	1.06	1.90	0.02	0.11
Lim2009.MaSC.Adam_BCR.2015.PMID.25575446	0.85	0.66	1.07	0.17	0.31
Lim2009.MatureLum.Adam_BCR.2015.PMID.25575446	1.07	0.78	1.46	0.68	0.80
Lim2009.Stroma.Adam_BCR.2015.PMID.25575446	0.76	0.60	0.95	0.02	0.11
Lim2010.LumProg.Adam_BCR.2015.PMID.25575446	1.35	1.02	1.81	0.04	0.15
Lim2010.MaSC.Adam_BCR.2015.PMID.25575446	0.93	0.73	1.18	0.54	0.68
Lim2010.MatureLum.Adam_BCR.2015.PMID.25575446	0.86	0.65	1.14	0.29	0.44
Lim2010.Stroma.Adam_BCR.2015.PMID.25575446	0.73	0.57	0.93	0.01	0.08
Lobular.Carcinoma.In.Situ_J.Pathol.2017.PMID.27861902	0.72	0.55	0.93	0.01	0.09
LOBULAR.TCGA.SIGNATURE.ImmuneCell.2015.PMID.26451490	1.07	0.83	1.37	0.62	0.74
LOBULAR.TCGA.SIGNATURE.Reactive_Cell.2015.PMID.26451490	0.82	0.65	1.02	0.08	0.21
LOBULAR.TCGA.SUBTYPE.Immune_Cell.2015.PMID.26451490	0.88	0.72	1.07	0.22	0.36
LOBULAR.TCGA.SUBTYPE.Proliferative_Cell.2015.PMID.26451490	0.88	0.71	1.09	0.24	0.38
LOBULAR.TCGA.SUBTYPE.Reactive_Cell.2015.PMID.26451490	0.89	0.71	1.11	0.31	0.46
LTS.score_JCI.2020.PMID.32573490	0.71	0.55	0.89	0.004	0.04
Luminal_Progenitor_Up_Nat.Med.2009.PMID.19648928	1.39	1.04	1.88	0.03	0.14

Luminal.cluster_BMC.Med.Genomics.2011.PMID.21214954	0.78	0.55	1.10	0.17	0.31
Luminal.Progenitor_BCR.2010.PMID.20346151	1.33	1.00	1.81	0.06	0.19
Luminal.Progenitor.Down_Nat.Med.2009.PMID.19648928	1.04	0.80	1.35	0.78	0.86
LumProg.HsEnriched_BCR.2015.PMID.25575446	1.36	1.01	1.85	0.05	0.17
LumProg.HsEnriched.Refined1_BCR.2015.PMID.25575446	1.02	0.79	1.32	0.85	0.91
LumProg.Lim09_BCR.2015.PMID.25575446	1.45	1.09	1.95	0.01	0.09
LumProg.Prat_BCR.2015.PMID.25575446	1.43	1.10	1.88	0.008	0.07
LumProg.Shehata_BCR.2015.PMID.25575446	1.29	1.00	1.67	0.06	0.19
Lums.HER2E.DOWN.metastatic.signature_JCI.2020.PMID.32573490	0.58	0.42	0.78	0.001	0.01
Lums.HER2E.UP.metastatic.signature_JCI.2020.PMID.32573490	1.17	0.87	1.57	0.30	0.45
Lung.WNT_Cancer.Res.2009.PMID.19549913	0.87	0.66	1.13	0.29	0.44
Lymph.vessels_Immunity.2013.PMID.24138885	0.97	0.73	1.28	0.81	0.89
Lymphovascular.Invasion_J.Pathol.2017.PMID.27861902	1.25	0.94	1.66	0.12	0.26
M.D.Metagene_Genome.Biol.2013.PMID.23618380	1.16	0.87	1.55	0.31	0.46
M2.Macrophage_Blood.2006.PMID.16556895	0.99	0.77	1.25	0.91	0.95
Macrophage_CellRep.2017.PMID.28052254	0.79	0.61	1.01	0.06	0.19
Macrophages_CancerImmunolRes.2018.PMID.30266715	1.03	0.80	1.33	0.80	0.88
Macrophages_Immunity.2013.PMID.24138885	1.10	0.84	1.43	0.50	0.64
Macrophages.M0_Nat.Methods.2015.PMID.25822800	1.22	0.95	1.57	0.12	0.25
Macrophages.M1_Nat.Methods.2015.PMID.25822800	1.43	1.08	1.92	0.01	0.09
Macrophages.M2_Nat.Methods.2015.PMID.25822800	0.95	0.74	1.22	0.69	0.80
MacTh1.cluster_CCR.2014.PMID.24916698	1.14	0.87	1.50	0.33	0.48
MammaPrint_Nature.2002.PMID.11823860	0.60	0.44	0.79	<0.001	0.01
MAPK.pathway.activation_NPJ.Precis.Oncol.2018.PMID.29872725	1.15	0.88	1.50	0.31	0.46
MASC.Down_Nat.Med.2009.PMID.19648928	1.28	0.98	1.69	0.08	0.21
MASC.Up_Nat.Med.2009.PMID.19648928	0.79	0.61	1.01	0.06	0.19
Mast.cell_CellRep.2017.PMID.28052254	0.82	0.63	1.08	0.16	0.30
Mast.cells_Immunity.2013.PMID.24138885	0.82	0.63	1.06	0.13	0.26
Mast.cells.activated_Nat.Methods.2015.PMID.25822800	0.99	0.77	1.28	0.95	0.97
Mast.cells.resting_Nat.Methods.2015.PMID.25822800	1.00	0.78	1.28	0.98	0.98
Mature.luminal_BCR.2010.PMID.20346151	1.17	0.86	1.59	0.31	0.46
Mature.Luminal.Down_Nat.Med.2009.PMID.19648928	0.86	0.65	1.11	0.25	0.39
Mature.LuminaUp_Nat.Med.2009.PMID.19648928	1.06	0.78	1.45	0.70	0.81
MatureLum.HsEnriched_BCR.2015.PMID.25575446	1.04	0.77	1.41	0.79	0.87
MatureLum.HsEnriched.Refined1_BCR.2015.PMID.25575446	1.25	0.91	1.71	0.17	0.31
MatureLum.Lim09_BCR.2015.PMID.25575446	1.12	0.84	1.51	0.45	0.60
MatureLum.Prat_BCR.2015.PMID.25575446	1.24	0.96	1.62	0.11	0.24
MatureLum.Shehata_BCR.2015.PMID.25575446	1.30	0.99	1.72	0.06	0.19
MBasal.Cluster_BMC.Med.Genomics.2011.PMID.21214954	0.83	0.66	1.04	0.11	0.25
MCD3.CD8_BMC.Med.Genomics.2011.PMID.21214954	1.22	0.92	1.61	0.17	0.31
MCF7.E2.induced.genes_JCO.2006.PMID.16505416	1.17	0.89	1.55	0.25	0.40
MCF7.E2.repressed.genes_JCO.2006.PMID.16505416	1.09	0.81	1.46	0.57	0.70
MDSC_CellRep.2017.PMID.28052254	1.32	1.01	1.74	0.04	0.16
MDSC.Granulocytic_Leukoc.Biol.2012.PMID.21954284	1.21	0.92	1.61	0.17	0.32
MDSC.Neutrophi_Leukoc.Biol.2012.PMID.21954284	1.11	0.85	1.44	0.45	0.60
MDSC.tumor_J.Immunol.2012.PMID.23152559	0.98	0.74	1.31	0.91	0.94
MDSC.tumor.MO_J.Immunol.2012.PMID.23152559	1.32	1.00	1.77	0.05	0.18

MECM_BMC.Med.Genomics.2011.PMID.21214954	0.80	0.63	1.01	0.06	0.19
Memory.B.cell_CellRep.2017.PMID.28052254	1.04	0.81	1.34	0.77	0.86
MET.DOWN.RNAseq.Significant.Genes_JCI.2018.PMID.29480819	1.15	0.84	1.57	0.39	0.53
MET.DOWN.Significant.Genes.Low.Basal.1_JCI.2018.PMID.29480819	0.96	0.74	1.26	0.78	0.86
MET.DOWN.Significant.Genes.Low.Basal.2_JCI.2018.PMID.29480819	1.00	0.76	1.31	0.99	0.99
MET.UP.RNAseq.Significant.Genes_JCI.2018.PMID.29480819	0.77	0.59	1.00	0.06	0.19
MET.UP.Significant.Genes.HIGH.BASALS.Genes_JCI.2018.PMID.29480819	1.16	0.86	1.58	0.33	0.48
Metaplastic.Up_CanRes.2009.PMID.19435916	0.74	0.57	0.96	0.02	0.12
Metastasis.predictor.TNBC_BCR.2010.PMID.20946665	0.99	0.75	1.31	0.96	0.97
MFGFR2_BMC.Med.Genomics.2011.PMID.21214954	1.02	0.77	1.37	0.87	0.92
MHC.Forero.11_Cancer.Immunol.Res.2016.PMID.26980599	1.36	1.01	1.85	0.05	0.17
MHC.Forero.24_Cancer.Immunol.Res.2016.PMID.26980599	0.94	0.68	1.28	0.68	0.79
MHC.I_BCR.2008.PMID.19272155	1.12	0.83	1.52	0.45	0.60
MHC.II_BCR.2008.PMID.19272155	1.20	0.90	1.59	0.21	0.36
MHCI.coreGenes_Nat.Commun.2017.PMID29170503	1.24	0.95	1.63	0.12	0.26
MIR200c.Induced_ONCO.2015.PMID.25746005	1.37	1.03	1.85	0.04	0.15
MIR200c.Repressed_ONCO.2015.PMID.25746005	0.78	0.58	1.02	0.08	0.21
miRNA.138.signature_Cancer.Res.2014.PMID.25339353	1.41	1.07	1.89	0.02	0.10
MITO1_BMC.Med.Genomics.2011.PMID.21214954	0.96	0.71	1.28	0.77	0.86
MITO2_BMC.Med.Genomics.2011.PMID.21214954	1.04	0.73	1.47	0.84	0.90
Mitotic.Count_J.Pathol.2017.PMID.27861902	1.30	1.00	1.70	0.05	0.17
MK14.K17_BMC.Med.Genomics.2011.PMID.21214954	1.18	0.91	1.54	0.21	0.35
MKRAS.amplicon_BMC.Med.Genomics.2011.PMID.21214954	1.29	0.95	1.77	0.11	0.25
MM.BRCAwnt.1pFDR.UP_Genome.Biology.2007.PMID.17493263	1.18	0.92	1.53	0.19	0.34
MM.C3Tag.1pFDR.UP_Genome.Biology.2007.PMID.17493263	1.40	1.08	1.84	0.01	0.09
MM.C3Tag.2012_Genome.Biol.2013.PMID.24220145	1.37	1.07	1.77	0.01	0.09
MM.Class3_Genome.Biol.2013.PMID.24220145	1.26	0.96	1.67	0.10	0.24
MM.Class8_Genome.Biol.2013.PMID.24220145	0.75	0.56	0.99	0.05	0.17
MM.Claudinlow_Genome.Biol.2013.PMID.24220145	0.80	0.62	1.02	0.08	0.21
MM.DMBAwnt.1pFDR.UP.Genome.Biology.2007.PMID.17493263	0.90	0.69	1.16	0.41	0.56
MM.Erbb2.like_Genome.Biol.2013.PMID.24220145	1.28	0.99	1.67	0.06	0.19
MM.Myc.2012_Genome.Biol.2013.PMID.24220145	1.34	1.01	1.79	0.04	0.16
MM.Myoepithelioma.like_Genome.Biol.2013.PMID.24220145	0.81	0.63	1.04	0.10	0.23
MM.Neu.2012_Genome.Biol.2013.PMID.24220145	1.20	0.94	1.54	0.16	0.30
MM.NeuPyMT.1pFDR.UP_Genome.Biology.2007.PMID.17493263	1.39	1.02	1.91	0.04	0.15
MM.Normal.1pFDR.UP_Genome.Biology.2007.PMID.17493263	0.82	0.65	1.02	0.08	0.21
MM.Normal.like_Genome.Biol.2013.PMID.24220145	0.93	0.76	1.12	0.44	0.59
MM.p53null.1pFDR.UP_Genome.Biology.2007.PMID.17493263	0.77	0.58	1.02	0.07	0.20
MM.p53null.Basal_Genome.Biol.2013.PMID.24220145	0.90	0.69	1.17	0.43	0.58
MM.p53null.Luminal_Genome.Biol.2013.PMID.24220145	0.95	0.69	1.29	0.72	0.82
MM.Potluck_1pFDR_UP_Genome.Biology.2007_PMID.17493263.PMID.24220145	0.85	0.65	1.10	0.21	0.36
MM.PyMT.2012_Genome.Biol.2013.PMID.24220145	1.60	1.18	2.21	0.003	0.04
MM.Squamous.like_Genome.Biol.2013.PMID.24220145	0.94	0.71	1.25	0.69	0.80
MM.Stat1_Genome.Biol.2013.PMID.24220145	0.94	0.73	1.22	0.66	0.78
MM.WapINT3.1pFDR.UP_Genome.Biology.2007.PMID.17493263	1.15	0.88	1.51	0.31	0.46
MM.WapINT3.2012_Genome.Biol.2013.PMID.24220145	0.77	0.57	1.03	0.08	0.21
MM.WAPTag.1pFDR.UP_Genome.Biology.2007.PMID.17493263	1.42	1.12	1.82	0.004	0.04

MM.Wnt1.Early_Genome.Biol.2013.PMID.24220145	1.00	0.77	1.30	>0.99	>0.99
MM.Wnt1.Late_Genome.Biol.2013.PMID.24220145	1.08	0.82	1.42	0.58	0.71
Mmyosin_BMC.Med.Genomics.2011.PMID.21214954	0.67	0.51	0.88	0.004	0.04
MNADH_CYTochrome_BMC.Med.Genomics.2011.PMID.21214954	1.29	0.97	1.74	0.09	0.22
MNB1_BMC.Med.Genomics.2011.PMID.21214954	1.01	0.80	1.26	0.94	0.96
MNB2_BMC.Med.Genomics.2011.PMID.21214954	0.99	0.82	1.20	0.93	0.96
MNB3_BMC.Med.Genomics.2011.PMID.21214954	0.79	0.60	1.01	0.08	0.21
MNOtch4_BMC.Med.Genomics.2011.PMID.21214954	1.06	0.79	1.42	0.69	0.80
Monocyte_CellRep.2017.PMID.28052254	1.19	0.87	1.63	0.28	0.43
Monocyte..DC.25gene_Genome.Biol.2013.PMID.23618380	1.14	0.86	1.52	0.37	0.52
Monocytes_CancerImmunolRes.2018.PMID.30266715	0.89	0.69	1.15	0.38	0.53
Monocytes_Nat.Methods.2015.PMID.25822800	1.02	0.79	1.32	0.88	0.93
Monocytic.lineage.MCP_Nature.2020.PMID.31942075	1.16	0.90	1.51	0.25	0.40
MProliferation_BMC.Med.Genomics.2011.PMID.21214954	1.42	1.12	1.83	0.005	0.05
MProtocadherin_BMC.Med.Genomics.2011.PMID.21214954	0.87	0.67	1.11	0.27	0.42
MPYMT_NEU_Cluster_BMC.Med.Genomics.2011.PMID.21214954	1.23	0.94	1.63	0.13	0.27
MRibosomal_BMC.Med.Genomics.2011.PMID.21214954	0.88	0.66	1.15	0.35	0.50
MS.CD44.DOWN_PNAS.2009.PMID.19666588	1.31	1.02	1.70	0.04	0.15
MS.CD44.UP_PNAS.2009.PMID.19666588	0.88	0.68	1.14	0.35	0.49
MSquamous_BMC.Med.Genomics.2011.PMID.21214954	0.87	0.66	1.12	0.28	0.43
Murat.G07_JCO.2008.PMID.18565887	0.83	0.64	1.07	0.14	0.28
Murat.G18_JCO.2008.PMID.18565887	0.86	0.64	1.14	0.29	0.44
Murat.G24_JCO.2008.PMID.18565887	1.17	0.89	1.53	0.26	0.40
MVEGFC_BMC.Med.Genomics.2011.PMID.21214954	0.85	0.65	1.09	0.21	0.35
Myeloid.cell.chemotaxis.1gene_Nature.2020.PMID.31942077	0.97	0.74	1.28	0.83	0.89
Myeloid.dendritic.cells.MCP_Nature.2020.PMID.31942077	1.01	0.77	1.32	0.96	0.97
Natural.killer.cell_CellRep.2017.PMID.28052254	0.75	0.56	1.00	0.05	0.18
Natural.killer.T.cell_CellRep.2017.PMID.28052254	0.93	0.72	1.20	0.59	0.72
Necrosis_J.Pathol.2017.PMID.27861902	1.73	1.26	2.42	0.001	0.02
Neutrophil_CellRep.2017.PMID.28052254	0.99	0.79	1.23	0.90	0.94
Neutrophils_CancerImmunolRes.2018.PMID.30266715	1.11	0.83	1.49	0.49	0.64
Neutrophils_Immunity.2013.PMID.24138885	0.83	0.64	1.05	0.13	0.26
Neutrophils_Nat.Methods.2015.PMID.25822800	0.93	0.73	1.19	0.59	0.72
Neutrophils.MCP_Nature.2020.PMID.31942077	0.98	0.77	1.24	0.85	0.90
NK_Immunity.2013.PMID.24138885	0.97	0.74	1.27	0.83	0.89
NK.activated_Nat.Methods.2015.PMID.25822800	1.17	0.89	1.55	0.27	0.42
NK.CD56bright_Immunity.2013.PMID.24138885	1.03	0.77	1.38	0.84	0.90
NK.CD56dim_Immunity.2013.PMID.24138885	1.53	1.18	2.01	0.002	0.03
NK.resting_Nat.Methods.2015.PMID.25822800	1.16	0.88	1.54	0.29	0.44
NKcells_CancerImmunolRes.2018.PMID.30266715	1.27	0.95	1.71	0.11	0.24
NKcells.MCP_Nature.2020.PMID.31942077	1.02	0.81	1.28	0.88	0.93
No.Response.Immunotherapy.TLS.Melanoma_Nature.2020.PMID.31942075	0.84	0.65	1.09	0.19	0.33
Normal.mucosa_Immunity.2013.PMID.24138885	0.83	0.65	1.04	0.11	0.24
Nuclear.Pleomorphism_J.Pathol.2017.PMID.27861902	1.20	0.92	1.58	0.18	0.32
Oncotype_NEJM.2004.PMID.15591335	1.91	1.46	2.55	<0.001	0.001
P53.ERPos.MDACC_CCR.2011.PMID.21248301	1.45	1.15	1.87	0.003	0.04
Parity.signature.251genes_BCR.2014.PMID.25005139	1.01	0.77	1.33	0.91	0.95

Parity.signature.40genes_BCR.2014.PMID.25005139	1.06	0.80	1.40	0.69	0.80
PARPi.Resistance_BCRT_2012.PMID.22875744	1.42	1.07	1.92	0.02	0.10
PARPi.Sensitivity_BCRT_2012.PMID.22875744	1.04	0.81	1.33	0.78	0.86
PARPi.Sensitivity.MDACC_NPJ.Syst.Biol.Appl.2017.PMID.28649435	1.28	0.96	1.73	0.09	0.23
PARPi.Sensitivity.Negative_Sci.Adv.2017.PMID.28439535	1.05	0.80	1.39	0.71	0.81
PARPi.Sensitivity.Positive_Sci.Adv.2017.PMID.28439535	1.30	0.98	1.74	0.07	0.20
Pcorr.Breast2Lung.LM2.Correlation_Nature.2005.PMID.16049480	0.94	0.71	1.23	0.65	0.76
Pcorr.Breast2Lung.Parental.Correlation_Nature.2005.PMID.16049480	1.14	0.87	1.50	0.34	0.49
Pcorr.dasatinib.resistant_Cancer.Res.2007.PMID.17332353	0.79	0.59	1.05	0.11	0.25
Pcorr.dasatinib.sensitive_Cancer.Res.2007.PMID.17332353	1.22	0.92	1.64	0.18	0.32
Pcorr.Hypoxia.High.Correlation_PLoS.Med.2006.PMID.16417408	1.07	0.81	1.41	0.62	0.74
Pcorr.Hypoxia.Low.Correlation_PLoS.Med.2006.PMID.16417408	0.85	0.64	1.12	0.24	0.38
Pcorr.IGS_Invasiveness_NJEM.2007.PMID.17229949	1.33	1.05	1.71	0.02	0.11
Pcorr.wound.response.activated_PNAS.2005.PMID.15701700	1.45	1.10	1.91	0.008	0.07
pCR.predictor.ERNeg.55genes_JAMA.2011.PMID.21558518	1.18	0.90	1.55	0.25	0.39
pCR.predictor.ERPos.39genes_JAMA.2011.PMID.21558518	0.96	0.75	1.25	0.78	0.86
PDCD1_Single_Gene.Single	1.34	1.02	1.78	0.04	0.14
Pfefferle2012.LumProg_BCR.2015.PMID.25575446	1.24	0.92	1.68	0.16	0.30
Pfefferle2012.MaSC_BCR.2015.PMID.25575446	0.90	0.70	1.14	0.37	0.52
Pfefferle2012.MatureLum_BCR.2015.PMID.25575446	1.30	0.97	1.75	0.09	0.22
Pfefferle2012.Stroma_BCR.2015.PMID.25575446	0.74	0.58	0.93	0.01	0.08
PGR_Single_Gene.Single	0.44	0.30	0.63	<0.001	0.001
PI3Ki.Down_CancerCell.2017.PMID.28528867	1.44	1.10	1.92	0.01	0.07
PI3Ki.Up_CancerCell.2017.PMID.28528867	1.01	0.77	1.32	0.96	0.97
PIK3CA.Pathway_Ann.Oncol.2017.PMID.28177460	1.07	0.83	1.39	0.58	0.71
PIK3CAmt.signature_Cancer.Res.2012.PMID.22552288	1.22	0.92	1.63	0.17	0.31
Plasma.cells_Nat.Methods.2015.PMID.25822800	1.44	1.07	1.95	0.02	0.10
PlasmaCells_CancerImmunolRes.2018.PMID.30266715	2.09	1.51	2.97	<0.001	0.001
Plasmacytoid.dendritic.cell_CellRep.2017.PMID.28052254	0.78	0.60	1.01	0.06	0.19
PR.Isoform.Ratio.Up.in.PRA.H_JNCI.2017.PMID.28376177	0.87	0.68	1.10	0.24	0.38
PR.Isoform.Ratio.Up.in.PRB.H_JNCI.2017.PMID.28376177	1.43	1.11	1.86	0.006	0.05
Proliferation.Cluster_BMC.Med.Genomics.2011.PMID.21214954	1.42	1.12	1.82	0.004	0.04
Proliferation.Metagene_Genome.Biol.2013.PMID.23618380	1.41	1.11	1.81	0.005	0.05
Proliferation.score.PAM50_JCO.2009.PMID.19204204	1.42	1.10	1.86	0.008	0.07
ProliferationPathway_CancerImmunolRes.2018.PMID.30266715	1.42	1.13	1.83	0.004	0.04
Prosigna.Proliferation.18_BMC.Med.Genomics.2015.PMID.26297356	1.40	1.10	1.81	0.007	0.06
Race.LuminalA.MRE.score_BCRT.2015.PMID.26109344	1.13	0.86	1.49	0.37	0.52
Radiation.induced.genes_Radoat.Res.2014.PMID.24527691	1.31	0.95	1.82	0.10	0.24
RB.LOH_BCR.2008.PMID.18782450	1.38	1.08	1.77	0.01	0.08
RB.LOSS_JCI.2007.PMID.17160137	1.45	1.14	1.88	0.003	0.04
Regulatory.T.cell_CellRep.2017.PMID.28052254	1.10	0.83	1.46	0.51	0.65
Replication.Stress.Down.set_Cell.Rep.2018.PMID.29768207	1.28	0.96	1.73	0.09	0.23
Replication.Stress.Model_Cell.Rep.2018_PMID.29768207.PMID.29768207	0.83	0.63	1.09	0.18	0.32
Replication.Stress.Neg_Cell.Rep.2018_PMID.29768207.PMID.29768207	1.38	1.04	1.87	0.03	0.14
Replication.Stress.Pos_Cell.Rep.2018_PMID.29768207.PMID.29768207	0.76	0.57	1.00	0.06	0.19
Replication.Stress.Up_Set_Cell.Rep.2018_PMID.29768207.PMID.29768207	0.77	0.58	1.02	0.08	0.21
Residual.disease.predictor.ERNeg.54genes_JAMA.2011.PMID.21558518	1.20	0.89	1.63	0.23	0.38

Residual.disease.predictor.ERPos.73genes_JAMA.2011.PMID.21558518	0.93	0.72	1.20	0.59	0.72
Response.Immunotherapy.MCP.TLS.Melanoma_Nature.2020.PMID.31942075	1.62	1.21	2.21	0.002	0.03
Response.Immunotherapy.signature_Science.2018.PMID.30309915	1.30	0.98	1.74	0.07	0.20
Response.Neo.Chemo_common_CCR.2014.PMID.25047707	1.36	1.07	1.75	0.01	0.09
Response.Neo.Chemo_ERNeg_CCR.2014.PMID.25047707	0.93	0.69	1.25	0.63	0.75
Response.Neo.Chemo_ERPos_CCR.2014.PMID.25047707	1.02	0.77	1.36	0.87	0.92
RHOA.pathway_Ann.Oncol.2017.PMID.28177460	1.03	0.78	1.36	0.85	0.91
Ribosomal.Cluster_BMC.Med.Genomics.2011.PMID.21214954	0.88	0.67	1.14	0.35	0.49
ROR.subtype.PAM50_JCO.2009.PMID.19204204	1.85	1.39	2.54	<0.001	0.002
ROR.subtype.proliferation.PAM50_JCO.2009.PMID.19204204	1.54	1.19	2.02	0.002	0.03
RSS.Score_CCR.2018.PMID.29921729	1.36	1.03	1.83	0.04	0.14
S100A9.A8_BMC.Med.Genomics.2011.PMID.21214954	2.73	1.88	4.08	<0.001	<0.001
Scorr.EMAT1.Correlation_BCR.2020.PMID.32641077	0.88	0.67	1.15	0.36	0.50
Scorr.EMAT2.Correlation_BCR.2020.PMID.32641077	0.69	0.52	0.92	0.01	0.09
Scorr.EMAT3.Correlation_BCR.2020.PMID.32641077	1.17	0.90	1.54	0.25	0.39
Scorr.EMAT4.Correlation_BCR.2020.PMID.32641077	1.29	0.95	1.78	0.11	0.24
Scorr.IE.Correlation_JCO.2006.PMID.16505416	0.63	0.48	0.82	0.001	0.01
Scorr.IIE.Correlation_JCO.2006.PMID.16505416	1.65	1.27	2.17	<0.001	0.007
Scorr.PAM50.Basal_JCO.2009.PMID.19204204	1.61	1.10	2.40	0.02	0.10
Scorr.PAM50.Her2_JCO.2009.PMID.19204204	2.03	1.52	2.76	<0.001	<0.001
Scorr.PAM50.LumA_JCO.2009.PMID.19204204	0.50	0.35	0.69	<0.001	0.002
Scorr.PAM50.LumB_JCO.2009.PMID.19204204	1.21	0.95	1.55	0.13	0.26
Scorr.PAM50.Normal_JCO.2009.PMID.19204204	0.72	0.56	0.91	0.007	0.06
Scorr.S329.L_Br.J.Cancer.2008.PMID.18382427	1.24	0.93	1.68	0.15	0.28
Scorr.S329.R_Br.J.Cancer.2008.PMID.18382427	0.73	0.53	1.00	0.05	0.18
Secretoglobin_BMC.Med.Genomics.2011.PMID.21214954	1.32	0.98	1.79	0.07	0.20
Shehata2012.ALDHneg_BCR.2015.PMID.25575446	1.38	1.04	1.84	0.03	0.13
Shehata2012.ALDHpos_BCR.2015.PMID.25575446	1.23	0.93	1.63	0.15	0.29
Shehata2012.Basal_BCR.2015.PMID.25575446	0.76	0.60	0.96	0.02	0.12
Shehata2012.Erbb3neg_BCR.2015.PMID.25575446	1.24	0.94	1.67	0.14	0.27
Shehata2012.LumProg_BCR.2015.PMID.25575446	1.35	1.05	1.75	0.02	0.12
Shehata2012.NCL_BCR.2015.PMID.25575446	1.30	0.99	1.72	0.07	0.20
Shehata2012.Stroma_BCR.2015.PMID.25575446	0.78	0.61	1.00	0.05	0.17
Spike2012.aMaSC_BCR.2015.PMID.25575446	0.71	0.54	0.93	0.01	0.09
Spike2012.fMaSC_BCR.2015.PMID.25575446	0.87	0.67	1.13	0.30	0.45
Spike2012.fStr_BCR.2015.PMID.25575446	0.80	0.63	1.01	0.07	0.20
STAT1_BCR.2008.PMID.19272155	1.38	1.06	1.82	0.02	0.10
STAT3.Basal_PNAS.2014.PMID.25139989	1.13	0.85	1.50	0.42	0.57
STAT3.Basal.short_PNAS.2014.PMID.25139989	1.11	0.83	1.48	0.47	0.62
Stroma.FNA.MDACC.1_JCO.2010.PMID.20805453	1.29	0.97	1.74	0.09	0.22
Stroma.FNA.MDACC.2_JCO.2010.PMID.20805453	0.78	0.60	1.00	0.05	0.18
Stromal.Central.Fibrotic.Focus_J.Pathol.2017.PMID.27861902	0.75	0.54	1.03	0.07	0.21
Stromal.Down_Nat.Med.2009.PMID.19648928	1.30	1.03	1.67	0.03	0.13
Stromal.Inflammation_J.Pathol.2017.PMID.27861902	1.38	1.05	1.82	0.02	0.12
Stromal.Signature_Nat.Med.2008.PMID.18438415	0.88	0.68	1.15	0.36	0.51
Stromal.Up_Nat.Med.2009.PMID.19648928	0.74	0.58	0.93	0.01	0.08
SW480.cancer.cells_Immunity.2013.PMID.24138885	1.03	0.78	1.38	0.82	0.89

T.follicular.helper.cell_CellRep.2017.PMID.28052254	0.92	0.71	1.20	0.55	0.68
Tcell.activation_Nature.2020.PMID.31942077	1.29	0.99	1.69	0.06	0.19
Tcell.CD8.Effector.vs.naive.2_Science.2016.PMID27789795	1.44	1.13	1.85	0.004	0.04
Tcell.CD8.Exhausted.vs.antiPDL1.2_Science.2016.PMID27789795	1.48	1.15	1.92	0.003	0.04
Tcell.CD8.Exhausted.vs.naive.2_Science.2016.PMID27789795	1.42	1.12	1.83	0.005	0.05
Tcell.CD8.Memory.vs.naive.1_Science.2016.PMID27789795	1.21	0.91	1.62	0.20	0.34
Tcell.cluster_CCR.2014.PMID.24916698	1.34	1.01	1.79	0.05	0.17
Tcell.EXH.Anti.PDL1.vs.control.treated.exhausted.CD8.Tcell.Metagene.1.Science.2016.PMID.27789795	1.05	0.80	1.38	0.71	0.81
Tcell.EXH.Effector.CD8.T.cell.at.day.8.p.i.Armstrong.vs.Naive.CD8.Tcell.Metagene.1_Science.2016.PMID.27789795	1.21	0.91	1.62	0.19	0.33
Tcell.EXH.Exhausted.CD8.T.cell.vs.Naive.CD8.T.cell.Metagene.1_Science.2016.PMID.27789795	1.23	0.93	1.64	0.16	0.29
Tcell.EXH.Exhausted.CD8.T.cell.vs.Naive.CD8.T.cell.Metagene.3_Science.2016.PMID.27789795	1.16	0.89	1.53	0.29	0.44
Tcell.EXH.Memory.CD8.T.cell.a.vs.Naive.CD8.T.cell.Metagene.1_Science.2016.PMID.27789795	1.21	0.91	1.62	0.20	0.34
Tcell.EXH.Memory.CD8.T.cell.a.vs.Naive.CD8.T.cell.Metagene.2_Science.2016.PMID.27789795	1.15	0.88	1.50	0.32	0.47
Tcell.EXH.Memory.CD8.T.cell.a.vs.Naive.CD8.T.cell.Metagene.3.Science_2016.PMID.27789795	1.30	0.96	1.79	0.10	0.23
Tcell.NK.51gene_Genome.Biol.2013.PMID.23618380	1.21	0.91	1.62	0.19	0.33
Tcell.NK.Metagene_Genome.Biol.2013.PMID.23618380	1.17	0.89	1.57	0.27	0.42
Tcell.RM_Nat_Med.2018.PMID.29942092	1.12	0.84	1.51	0.44	0.58
Tcell.survival.2gene_Nature.2020.PMID.31942077	1.37	1.04	1.83	0.03	0.13
Tcells_CancerImmunolRes.2018.PMID.30266715	1.18	0.90	1.57	0.24	0.38
Tcells_Immunity.2013.PMID.24138885	1.23	0.93	1.65	0.15	0.29
Tcells_TFH_Nat.Methods.2015.PMID.25822800	1.25	0.94	1.67	0.12	0.26
Tcells.CD4.memory.activated_Nat.Methods.2015.PMID.25822800	1.38	1.04	1.83	0.03	0.12
Tcells.CD4.memory.resting_Nat.Methods.2015.PMID.25822800	1.23	0.93	1.64	0.15	0.29
Tcells.CD4.naive_Nat.Methods.2015.PMID.25822800	1.25	0.95	1.65	0.12	0.25
Tcells.CD8_Immunity.2013.PMID.24138885	0.82	0.60	1.13	0.24	0.38
Tcells.CD8_Nat.Methods.2015.PMID.25822800	1.25	0.95	1.66	0.12	0.25
Tcells.CD8.MCP_Nature.2020.PMID.31942075	1.29	0.97	1.74	0.08	0.21
Tcells.Cytotoxic.MCP_Nature.2020.PMID.31942075	1.28	0.96	1.73	0.09	0.23
Tcells.gammadelta_Nat.Methods.2015.PMID.25822800	1.24	0.95	1.65	0.12	0.26
Tcells.helper_Immunity.2013.PMID.24138885	1.18	0.90	1.56	0.24	0.38
Tcells.MCP_Nature.2020.PMID.31942077	1.27	0.96	1.70	0.10	0.24
Tcells.regulatory.2gene_Nature.2020.PMID.31942077	0.93	0.69	1.25	0.64	0.75
Tcells.Tregs_Nat.Methods.2015.PMID.25822800	1.21	0.92	1.60	0.18	0.33
TCGA.BRCA.1198_BASAL_JCI.2020.PMID.32573490	0.79	0.62	1.01	0.06	0.19
TCGA.BRCA.1198_Chromogranin_JCI.2020.PMID.32573490	1.04	0.79	1.36	0.75	0.85
TCGA.BRCA.1198_COLLAGEN11A_JCI.2020.PMID.32573490	0.95	0.74	1.23	0.69	0.80
TCGA.BRCA.1198_EN1_FDZ9_JCI.2020.PMID.32573490	1.31	0.93	1.85	0.12	0.26
TCGA.BRCA.1198_FGFR4_EGF_JCI.2020.PMID.32573490	2.00	1.48	2.76	<0.001	0.001
TCGA.BRCA.1198_HISTONES_JCI.2020.PMID.32573490	1.06	0.81	1.39	0.68	0.79
TCGA.BRCA.1198_HOXC11_HOTAIR_SIX1_JCI.2020.PMID.32573490	1.48	1.14	1.95	0.004	0.04
TCGA.BRCA.1198_IL8_CCL_JCI.2020.PMID.32573490	1.36	1.04	1.78	0.02	0.12
TCGA.BRCA.1198_immune_CD19_JCI.2020.PMID.32573490	1.30	0.99	1.74	0.06	0.20
TCGA.BRCA.1198_immune_CD34_TIE1_JCI.2020.PMID.32573490	0.82	0.65	1.02	0.08	0.21
TCGA.BRCA.1198_immune_CD4_CD53_CD84_BTK_JCI.2020.PMID.32573490	1.15	0.88	1.51	0.30	0.45
TCGA.BRCA.1198_immune_CD8_GZMK_JCI.2020.PMID.32573490	1.29	0.98	1.72	0.08	0.21
TCGA.BRCA.1198_immune_CTLA4_CXCL_FOXP3_JCI.2020.PMID.32573490	1.48	1.13	1.96	0.005	0.05
TCGA.BRCA.1198_immune_FOS_JUN_IL6_JCI.2020.PMID.32573490	0.80	0.58	1.10	0.18	0.32

TCGA.BRCA.1198_immune_GIMAP_IL16_JCI.2020.PMID.32573490	0.96	0.73	1.27	0.80	0.87
TCGA.BRCA.1198_immune_HLA_A_F_JCI.2020.PMID.32573490	1.18	0.90	1.57	0.24	0.38
TCGA.BRCA.1198_immune_HLA_D_JCI.2020.PMID.32573490	1.16	0.87	1.55	0.30	0.45
TCGA.BRCA.1198_immune_INTERFERON_JCI.2020.PMID.32573490	0.95	0.74	1.24	0.72	0.82
TCGA.BRCA.1198_IMMUNE1_JCI.2020.PMID.32573490	1.85	1.36	2.56	<0.001	0.004
TCGA.BRCA.1198_LUMINAL_JCI.2020.PMID.32573490	0.54	0.36	0.79	0.002	0.03
TCGA.BRCA.1198_MYBL2_APOBEC3B_JCI.2020.PMID.32573490	1.54	1.21	2.00	0.001	0.02
TCGA.BRCA.1198_NORMAL_JCI.2020.PMID.32573490	0.83	0.68	1.02	0.07	0.21
TCGA.BRCA.1198_NORMAL2_JCI.2020.PMID.32573490	1.07	0.85	1.36	0.57	0.70
TCGA.BRCA.1198_PDCHA_MANY_JCI.2020.PMID.32573490	0.92	0.69	1.24	0.60	0.73
TCGA.BRCA.1198_S100A7_8_9_JCI.2020.PMID.32573490	1.51	1.08	2.12	0.02	0.10
TCGA.BRCA.1198_TP63_JCI.2020.PMID.32573490	0.84	0.67	1.05	0.13	0.26
TCGA.BRCA.1198.IMMUNOGLOBULIN_JCI.2020.PMID.32573490	2.24	1.60	3.24	<0.001	0.001
TCGA.CSF1.response_Immunity.2018.PMID.29628290	1.11	0.84	1.46	0.46	0.61
TCGA.IFN.score_Immunity.2018.PMID.29628290	0.96	0.74	1.24	0.76	0.85
TCGA.Liexpression.score_Immunity.2018.PMID.29628290	1.25	0.94	1.66	0.13	0.26
TCGA.Serum.response.up_Immunity.2018.PMID.29628290	1.31	1.00	1.74	0.05	0.18
TCGA.TFH_Immunity.2018.PMID.29628290	1.21	0.89	1.64	0.22	0.37
TCGA.Tgd_Immunity.2018.PMID.29628290	0.83	0.66	1.04	0.11	0.24
TCGA.TGFB.score_Immunity.2018.PMID.29628290	0.99	0.76	1.29	0.91	0.95
Tcm_Immunity.2013.PMID.24138885	1.18	0.90	1.55	0.23	0.38
Tem_Immunity.2013.PMID.24138885	0.67	0.51	0.87	0.003	0.04
TFH_Immunity.2013.PMID.24138885	1.21	0.89	1.64	0.22	0.37
Tgd_Immunity.2013.PMID.24138885	0.83	0.66	1.04	0.11	0.24
Th1_cells_Immunity.2013.PMID.24138885	1.34	1.01	1.80	0.05	0.17
Th17_cells_Immunity.2013.PMID.24138885	1.02	0.77	1.35	0.89	0.93
Th2_cells_Immunity.2013.PMID.24138885	1.16	0.91	1.51	0.24	0.38
TLS.9Gene.Signature_Nature.2020.PMID.31942071	1.00	0.77	1.30	0.97	0.98
TLS.CXCL13.SingleGene_Nature.2020.PMID.31942077	1.31	1.00	1.73	0.05	0.17
TLS.Hallmark.Gene.Signature_Nature.2020.PMID.31942071	1.18	0.89	1.58	0.24	0.39
TLS.Known.Markers_Nature.2020.PMID.31942071	1.12	0.84	1.50	0.44	0.59
TLS.Structure.12chemokine_FrontImmunol.2017.PMID.28713385	1.26	0.95	1.69	0.12	0.25
TLS.tumors.wTLS.and.CD8.vs.CD8alone_Nature.2020.PMID.31942071	1.24	0.94	1.65	0.13	0.27
TNBC.good.prognosis.TNBC.230genes_BCR.2011.PMID.21978456	1.25	0.96	1.64	0.10	0.23
TNBC.good.prognosis.TNBC.26genes_BCR.2011.PMID.21978456	1.27	0.93	1.73	0.13	0.27
TNBC.metastasis.free.survival_PLoS.One.2013.PMID.24349199	1.19	0.93	1.53	0.18	0.32
TNBC.poor.prognosis.TNBC.26genes_BCR.2011.PMID.21978456	1.06	0.83	1.37	0.63	0.75
Translation.Pathway_CancerImmunolRes.2018.PMID.30266715	0.88	0.67	1.14	0.33	0.48
Tumour.hypoxia.causes.DNA.hypermethylation_Nature.2016.PMID.27533040	1.32	0.99	1.78	0.06	0.19
Type.1.T.helper.cell_CellRep.2017.PMID.28052254	0.98	0.74	1.31	0.91	0.95
Type.17.T.helper.cell_CellRep.2017.PMID.28052254	1.63	1.23	2.20	0.001	0.02
Type.2.T.helper.cell_CellRep.2017.PMID.28052254	1.08	0.80	1.45	0.63	0.75
Up.Basal.High_Nat.Cell.Biol.2014.PMID.25173976	0.78	0.58	1.03	0.08	0.22
Up.Proliferation_Nat.Cell.Biol.2014.PMID.25173976	1.50	1.17	1.94	0.002	0.03
Upregulated.by.oncogenic.NRAS.basal_Cell.Rep.2016.PMID.26166574	0.75	0.58	0.96	0.03	0.12
Upregulated.upon.NRAS.repression.basal_Cell.Rep.2017.PMID.26166574	0.65	0.49	0.85	0.002	0.03
Vascular.Content_Clin.Exp.Metastasis.2014.PMID.23975155	0.90	0.68	1.19	0.45	0.60

VEGF.13genes_BMC.Med.2009.PMID.19291283	0.94	0.71	1.24	0.68	0.79
Wirapati.Proliferation_BCR.2008.PMID.18662380	1.35	1.06	1.75	0.02	0.10
Wound.Signature_CCR.2009.PMID.19887484	0.72	0.55	0.93	0.01	0.09
X11q13.Amplicon_BMC.Med.Genomics.2011.PMID.21214954	0.92	0.69	1.21	0.56	0.70
X12qMDM4.BMC.Med.Genomics.2011.PMID.21214954	1.14	0.85	1.55	0.37	0.52
X13q14.Amplicon_BMC.Med.Genomics.2011.PMID.21214954	1.07	0.77	1.49	0.68	0.80
X15q25.Amplicon_BMC.Med.Genomics.2011.PMID.21214954	1.31	0.98	1.76	0.07	0.20
X16.13.Amplicon_BMC.Med.Genomics.2011.PMID.21214954	1.12	0.83	1.51	0.47	0.61
X16q23.Amplicon_BMC.Med.Genomics.2011.PMID.21214954	1.10	0.82	1.49	0.53	0.66
X17PP13.Amplicon_BMC.Med.Genomics.2011.PMID.21214954	1.07	0.82	1.40	0.64	0.76
X17q25x.BMC.Med.Genomics.2011.PMID.21214954	1.49	1.08	2.09	0.02	0.10
X19p13.Amplicon_BMC.Med.Genomics.2011.PMID.21214954	0.94	0.68	1.31	0.73	0.82
X1p36.Amplicon_BMC.Med.Genomics.2011.PMID.21214954	1.18	0.85	1.66	0.33	0.47
X3p21.Amplicon_BMC.Med.Genomics.2011.PMID.21214954	0.85	0.64	1.12	0.26	0.40
X4p16.Amplicon_BMC.Med.Genomics.2011.PMID.21214954	0.91	0.67	1.23	0.52	0.66
X5Q_BCRT.2012.PMID.22048815	0.82	0.60	1.11	0.19	0.33
X8p.Amplicon_BMC.Med.Genomics.2011.PMID.21214954	1.16	0.89	1.51	0.27	0.42
X8p22.Amplicon_BMC.Med.Genomics.2011.PMID.21214954	0.77	0.59	1.02	0.07	0.20
XBP1.Signature_Nature.2014.PMID.24670641	0.92	0.72	1.18	0.52	0.66

NeoALTTO					
Signature	OR	95% CI		*P*	adjusted *P*
Activate.Endothelium_Clin.Exp.Metastasis.2014.PMID.23975155	1.48	1.09	2.03	0.01	0.29
Activated.B.cell_CellRep.2017.PMID.28052254	1.23	0.92	1.65	0.17	0.46
Activated.Blood.Neutrophil.Signature_Nat.Cell.Biol.2019.PMID.31263265	1.03	0.80	1.32	0.84	0.94
Activated.Cancer.Cell.Signature_Nat.Cell.Biol.2019.PMID.31263265	0.94	0.71	1.25	0.68	0.88
Activated.CD4.T.cell_CellRep.2017.PMID.28052254	1.72	1.25	2.42	0.001	0.06
Activated.CD8.T.cell_CellRep.2017.PMID.28052254	1.33	1.00	1.78	0.05	0.37
Activated.dendritic.cell_CellRep.2017.PMID.28052254	1.34	0.99	1.83	0.06	0.37
Activated.Lung.MSC.Signature_Nat.Cell.Biol.2019.PMID.31263265	0.82	0.63	1.07	0.15	0.45
Activated.Lung.Neutrophil.Signature_Nat.Cell.Biol.2019.PMID.31263265	1.25	0.92	1.70	0.16	0.46
aDC_Immunity.2013_PMID.24138885.PMID.24138885	1.34	1.01	1.80	0.04	0.37
ADM.S100A10.A110NDGR1.Cluster_BMC.Med.Genomics.2011.PMID.21214954	1.26	0.94	1.69	0.12	0.40
African.and.European.Ancestry.TCGA.Negative_JAMA.Oncol.2017.PMID.28472234	0.97	0.70	1.32	0.83	0.94
African.and.European.Ancestry.TCGA.Positive_JAMA.Oncol.2017.PMID.28472234	1.02	0.77	1.35	0.89	0.97
Age.associated.signature_Genome.Biol.2015.PMID.26343147	0.92	0.71	1.20	0.55	0.79
aMaSC_BCR.2010.PMID.20346151	0.98	0.71	1.36	0.92	0.98
aMaSC.HsEnriched_BCR.2015.PMID.25575446	1.05	0.78	1.43	0.74	0.91
aMaSC.HsEnriched.Refined1_BCR.2015.PMID.25575446	0.79	0.57	1.09	0.16	0.46
aMaSC.Lim09_BCR.2015.PMID.25575446	1.13	0.85	1.50	0.40	0.69
aMaSC.Prat_BCR.2015.PMID.25575446	1.10	0.82	1.48	0.54	0.79
aMaSC.Shehata_BCR.2015.PMID.25575446	0.93	0.72	1.20	0.56	0.79
aMaSC.Signature_Cell.Stem.Cell.2012.PMID.22305568	1.02	0.75	1.38	0.91	0.98
AMPH.EPIREGULIN.Cluster_BMC.Med.Genomics.2011.PMID.21214954	0.95	0.69	1.30	0.77	0.93
Amplification.50_Genome.Biol.2014.PMID.25164602	1.08	0.83	1.40	0.58	0.79
Amplification.50.better.than._Genome.Biol.2015.PMID.25164602	0.93	0.71	1.22	0.61	0.81
Apocrine.Features_J.Pathol.2017.PMID.27861902	1.30	0.96	1.76	0.09	0.38
aStr.HsEnriched_BCR.2015.PMID.25575446	1.10	0.80	1.53	0.54	0.79
aStr.HsEnriched.Refined1_BCR.2015.PMID.25575446	1.09	0.79	1.50	0.60	0.81
aStr.HsEnriched.Refined2_BCR.2015.PMID.25575446	1.08	0.79	1.49	0.63	0.82
aStr.Lim09_BCR.2015.PMID.25575446	0.99	0.71	1.37	0.94	0.99
aStr.Prat_BCR.2015.PMID.25575446	1.04	0.76	1.43	0.80	0.94
aStr.Shehata_BCR.2015.PMID.25575446	1.02	0.74	1.41	0.90	0.97
BASAL.Cluster_BMC.Med.Genomics.2011.PMID.21214954	0.94	0.68	1.30	0.72	0.90
Bcell.cluster_CCR.2014.PMID.24916698	1.29	0.96	1.77	0.10	0.38
Bcell.IL10.MINUS_Immunol.2014.PMID.25080484	0.96	0.71	1.28	0.77	0.92
Bcell.IL10.PLUS_Immunol.2014.PMID.25080484	1.15	0.85	1.55	0.37	0.66

Bcell.lineage.MCP_Nature.2020.PMID.31942077	1.17	0.88	1.58	0.28	0.56
Bcell.Plasma.52gene_Genome.Biol.2013.PMID.23618380	1.20	0.89	1.63	0.24	0.54
Bcell.Plasma.Metagene_Genome.Biol.2013.PMID.23618380	1.33	0.99	1.83	0.07	0.37
Bcell.Tcell.Cooperation_Cell.2019.PMID.31730857	1.18	0.88	1.59	0.27	0.56
Bcells_CancerImmunolRes.2018.PMID.30266715	1.23	0.92	1.65	0.17	0.46
Bcells_Immunity.2013.PMID.24138885	1.23	0.92	1.65	0.16	0.46
Bcells.Centroblast_JCO.2015.PMID.25800755	1.35	0.99	1.87	0.06	0.37
Bcells.Centrocyte_JCO.2015.PMID.25800755	1.35	1.02	1.80	0.04	0.37
Bcells.Memory_JCO.2015.PMID.25800755	0.96	0.71	1.29	0.76	0.92
Bcells.memory_Nat.Methods.2015.PMID.25822800	1.23	0.93	1.65	0.15	0.45
Bcells.Naive_JCO.2015.PMID.25800755	1.30	0.98	1.75	0.07	0.37
Bcells.naive_Nat.Methods.2015.PMID.25822800	1.18	0.89	1.56	0.26	0.55
Bcells.Plasmablast_JCO.2015.PMID.25800755	1.11	0.82	1.50	0.51	0.78
Blood.vessels_Immunity.2013.PMID.24138885	1.13	0.82	1.56	0.46	0.75
bMYB.Signature_Oncogene.2009.PMID.19043454	1.33	0.98	1.84	0.08	0.37
C3TAG.Responding_CCR.2013.PMID.23780888	0.99	0.70	1.40	0.96	>0.99
C3TAG.Untreated_CCR.2013.PMID.23780888	1.53	1.09	2.17	0.02	0.30
CD103.Negative_Cancer.Cell.2014.PMID.25446897	1.15	0.85	1.56	0.37	0.66
CD103.Positive_Cancer.Cell.2014.PMID.25446897	1.17	0.90	1.52	0.25	0.55
CD103.Ratio_Cancer.Cell.2014.PMID.25446897	1.34	0.99	1.83	0.06	0.37
CD274_Single_Gene.Single	1.21	0.90	1.63	0.22	0.52
CD34.CD36.Cluster_BMC.Med.Genomics.PMID.21214954	1.09	0.77	1.55	0.61	0.82
CD44.downregulated.genes_Cancer.Cell.2007.PMID.17349583	1.06	0.79	1.42	0.69	0.88
CD44.upregulated.genes_Cancer.Cell.2007.PMID.17349583	1.20	0.92	1.58	0.17	0.46
CD56bright.natural.killer.cell_CellRep.2017.PMID.28052254	1.21	0.93	1.58	0.16	0.46
CD56dim.natural.killer.cell_CellRep.2017.PMID.28052254	1.24	0.94	1.64	0.13	0.43
CD68.cluster_CCR.2014.PMID.24916698	1.09	0.82	1.48	0.55	0.79
CD8.cluster_CCR.2014.PMID.24916698	1.32	0.99	1.78	0.06	0.37
CDKN2A_Single_Gene.Single	0.81	0.60	1.06	0.14	0.43
Central.memory.CD4.T.cell_CellRep.2017.PMID.28052254	0.83	0.64	1.07	0.16	0.46
Central.memory.CD8.T.cell_CellRep.2017.PMID.28052254	1.10	0.83	1.49	0.51	0.77
CES.Score_CCR.2017.PMID.27903675	0.46	0.30	0.70	<0.001	0.03
Chromogramin_BMC.Med.Genomics.2011.PMID.21214954	0.91	0.69	1.20	0.50	0.76
CIN70_Nat.Genet.2006.PMID.16921376	1.50	1.07	2.14	0.02	0.35
Claudin.High_Genome.Biol.2007.PMID.17493263	1.29	0.95	1.75	0.10	0.38
Claudin.Low_Genome.Biol.2007.PMID.17493263	0.98	0.70	1.38	0.89	0.97
Claudin.Low.29_Cancer.Res.2009.PMID.19435916	1.26	0.87	1.87	0.24	0.54
cMYB.Signature_PLoS.One.2010.PMID.20949095	0.86	0.67	1.11	0.25	0.55
CORE.Bcell.signature.Garber_Cell.Mol.Gastroenterol.Hepatol.2017.PMID.28508029	1.20	0.90	1.59	0.21	0.52
CTLA4_Single_Gene.Single	1.42	1.04	1.97	0.03	0.37
Cytolytic.activity_Cell.2015.PMID.25594174	1.32	1.00	1.76	0.06	0.37
Cytotoxic.cells_Immunity.2013.PMID.24138885	1.26	0.95	1.68	0.11	0.39
Day7.Downregulated_Nat.Cell.Biol.2014.PMID.25173976	1.00	0.73	1.39	0.99	>0.99
Day7.Upregulated_Nat.Cell.Biol.2014.PMID.25173976	1.22	0.94	1.60	0.15	0.45
DC_Immunity.2013.PMID.24138885	1.26	0.89	1.79	0.19	0.49
DCIS.HGF.down_BCR.2013.PMID.24025166	0.79	0.60	1.03	0.08	0.37
DCIS.HGF.up_BCR.2014.PMID.24025166	1.05	0.81	1.37	0.69	0.88

Delection.50_Genome.Biol.2016.PMID.25164602	0.79	0.57	1.09	0.16	0.46
Delection.50.better.than_Genome.Biol.2017.PMID.25164602	0.90	0.68	1.18	0.44	0.73
Dendritic.cells.activated_Nat.Methods.2015.PMID.25822800	1.31	0.96	1.79	0.09	0.38
Dendritic.cells.resting_Nat.Methods.2015.PMID.25822800	1.39	1.02	1.91	0.04	0.37
Down.Basal.High_Nat.Cell.Biol.2014.PMID.25173976	0.94	0.67	1.32	0.74	0.91
Down.CLOW.High_Nat.Cell.Biol.2014.PMID.25173976	1.00	0.73	1.37	0.99	>0.99
Downregulated.upon.NRAS.repression.basal_Cell.Rep.2015.PMID.26166574	1.07	0.81	1.42	0.63	0.83
Ductal.Carcinoma.In.Situ_J.Pathol.2017.PMID.27861902	1.12	0.84	1.49	0.44	0.72
Duke.Module01.acidosis_PNASUSA.2010.PMID.20335537	0.81	0.57	1.14	0.23	0.53
Duke.Module02.akt_PNASUSA.2010.PMID.20335537	1.21	0.93	1.59	0.16	0.46
Duke.Module03.betacatenin_PNASUSA.2010.PMID.20335537	1.11	0.84	1.46	0.46	0.74
Duke.Module04.E2F1_PNASUSA.2010.PMID.20335537	1.14	0.87	1.50	0.35	0.64
Duke.Module05.EGFR_PNASUSA.2010.PMID.20335537	1.11	0.83	1.49	0.49	0.76
Duke.Module06.ER_PNASUSA.2010.PMID.20335537	1.07	0.76	1.51	0.70	0.89
Duke.Module07.glucosedepletion_PNASUSA.2010.PMID.20335537	1.04	0.80	1.36	0.78	0.93
Duke.Module08.HER2_PNASUSA.2010.PMID.20335537	1.46	1.12	1.94	0.006	0.23
Duke.Module09.hypoxia_PNASUSA.2010.PMID.20335537	0.97	0.75	1.26	0.82	0.94
Duke.Module10.IFNA_PNASUSA.2010.PMID.20335537	0.98	0.75	1.28	0.87	0.96
Duke.Module11.IFNG_PNASUSA.2010.PMID.20335537	0.98	0.75	1.29	0.89	0.97
Duke.Module12.lacticacidosis_PNASUSA.2010.PMID.20335537	1.25	0.95	1.66	0.11	0.39
Duke.Module13.myc_PNASUSA.2010.PMID.20335537	1.21	0.93	1.58	0.16	0.46
Duke.Module14.p53_PNASUSA.2010.PMID.20335537	0.81	0.59	1.11	0.19	0.49
Duke.Module15.p63_PNASUSA.2010.PMID.20335537	1.10	0.84	1.45	0.48	0.76
Duke.Module16.pi3k_PNASUSA.2010.PMID.20335537	1.13	0.86	1.49	0.37	0.66
Duke.Module17.PR_PNASUSA.2010.PMID.20335537	0.75	0.52	1.07	0.11	0.39
Duke.Module18.ras_PNASUSA.2010.PMID.20335537	1.35	1.04	1.77	0.03	0.37
Duke.Module19.src_PNASUSA.2010.PMID.20335537	0.91	0.70	1.18	0.49	0.76
Duke.Module20.STAT3_PNASUSA.2010.PMID.20335537	1.31	0.96	1.81	0.09	0.38
Duke.Module21.TGFB_PNASUSA.2010.PMID.20335537	1.29	0.96	1.75	0.10	0.38
Duke.Module22.TNFA_PNASUSA.2010.PMID.20335537	1.19	0.88	1.62	0.25	0.55
Durvalumab.signature_CCR.2018.PMID.29716923	1.25	0.95	1.65	0.11	0.39
Early.IRS.1_PLoS.One.2016.PMID.26991655	1.25	0.92	1.71	0.16	0.46
Early.IRS.2_PLoS.One.2016.PMID.26991655	0.93	0.71	1.22	0.58	0.79
Early.Relapse.ERPos.33genes_JAMA.2011.PMID.21558518	0.92	0.70	1.20	0.53	0.79
Early.Response.ERNeg.27genes_JAMA.2011.PMID.21558518	1.08	0.83	1.41	0.58	0.79
Effector.memeory.CD4.T.cell_CellRep.2017.PMID.28052254	1.09	0.85	1.41	0.52	0.78
Effector.memeory.CD8.T.cell_CellRep.2017.PMID.28052254	1.48	1.09	2.03	0.01	0.29
EGFR_Single_Gene.Single	0.83	0.62	1.11	0.21	0.52
EMT.down.Taube_PNAS.2010.PMID.20713713	1.18	0.88	1.60	0.27	0.56
EMT.down.Weingberg_PNAS.2010.PMID.20713713	0.92	0.66	1.26	0.59	0.79
EMT.up.Taube_PNAS.2010.PMID.20713713	0.87	0.66	1.16	0.35	0.64
EMT.up.Weinberg_PNAS.2010.PMID.20713713	1.05	0.78	1.42	0.74	0.91
Endothelial.cells.MCP_Nature.2020..PMID.31942077	0.99	0.72	1.37	0.97	>0.99
Endothelial.Normal_Angiogenesis.2014.PMID.24257808	1.31	0.97	1.80	0.08	0.37
Endothelial.Tumor_Angiogenesis.2014.PMID.24257808	1.05	0.79	1.40	0.74	0.91
Eosinophil_CellRep.2017.PMID.28052254	0.93	0.71	1.20	0.56	0.79
Eosinophils_Immunity.2013.PMID.24138885	0.94	0.70	1.26	0.68	0.88

Eosinophils_Nat.Methods.2015.PMID.25822800	1.09	0.81	1.46	0.58	0.79
Epithelial.Tubule.Formation_J.Pathol.2017.PMID.27861902	0.61	0.46	0.79	<0.001	0.03
ERBB2_Single_Gene.Single	3.39	2.13	5.74	<0.001	0.001
ERBB3_Single_Gene.Single	1.14	0.80	1.63	0.47	0.75
ESR1_Single_Gene.Single	0.47	0.31	0.71	<0.001	0.03
ESTIMATE.Immune_Nat.Commun.2013.PMID.24113773	1.32	0.99	1.77	0.06	0.37
ESTIMATE.Stromal_Nat.Commun.2013.PMID.24113773	1.05	0.78	1.42	0.75	0.91
Euclidean.Distance.CLOW_BCR.2010.PMID.20813035	1.01	0.72	1.41	0.96	>0.99
EXTENDED.Bcell.signature.Garber_Cell.Mol.Gastroenterol.Hepatol.2017.PMID.28508029	1.02	0.78	1.34	0.86	0.96
FGFR4_Single_Gene.Single	1.33	1.01	1.77	0.05	0.37
FGFR4.Induced_JCI.2020.PMID.32573490	1.10	0.85	1.43	0.48	0.76
FGFR4.Repressed_JCI.2020.PMID.32573490	0.78	0.59	1.04	0.09	0.38
Fibrinogen.Cluster_BMC.Med.Genomics.2011.PMID.21214954	0.73	0.55	0.97	0.03	0.37
Fibroblast.Cluster_BMC.Med.Genomics.2011.PMID.21214954	0.98	0.73	1.32	0.91	0.98
Fibroblasts.MCP_Nature.2020.PMID.31942077	1.00	0.76	1.31	0.98	>0.99
Fibromatosis_Lab.Invest.2008.PMID.18414401	0.98	0.73	1.31	0.88	0.96
fMaSC.Metab_CellRep.2018.PMID.30089273	0.92	0.70	1.21	0.56	0.79
fMaSC.Metab8_CellRep.2018.PMID.30089273	1.19	0.90	1.59	0.22	0.52
fMaSC.refined1_BCR.2015.PMID.25575446	1.05	0.78	1.43	0.73	0.91
fMasC.Signature_Cell.Stem.Cell.2012.PMID.22305568	0.90	0.67	1.20	0.46	0.74
fMaSC.Signature_CellRep.2018.PMID.30089273	1.19	0.93	1.54	0.17	0.46
FOS.JUN_Cluster_BMC.Med.Genomics.2011.PMID.21214954	1.07	0.84	1.36	0.57	0.79
FOXC1.Hair.Follicles.P30C.LO.vs.WT.Negative_Science.2016.PMID.26912704	0.94	0.72	1.24	0.66	0.86
FOXC1.Hair.Follicles.P30C.LO.vs.WT.Positive_Science.2016.PMID.26912704	1.14	0.86	1.53	0.35	0.64
fSTR.Signature_Cell.Stem.Cell.2012.PMID.22305568	0.74	0.52	1.04	0.08	0.37
Gamma.delta.T.cell_CellRep.2017.PMID.28052254	1.02	0.78	1.33	0.90	0.97
GATA3.induced.genes_JCO.2006.PMID.16505416	1.34	0.98	1.86	0.07	0.37
GATA3.induced.genes_Oncogene.2004.PMID.15361840	1.37	1.02	1.88	0.05	0.37
GDF11.TGFBR3_Nat.Cell.Biol.2014.PMID.24658685	0.97	0.73	1.28	0.82	0.94
Glycolysis_BMC.Med.2009.PMID.19291283	1.29	0.97	1.72	0.08	0.37
GO.DOWN.with.SOX10.OE_Cell.Rep.2015.PMID.26365194	1.15	0.87	1.53	0.33	0.62
GO.UP.with.SOX10.OE_Cell.Rep.2015.PMID.26365194	0.86	0.66	1.13	0.29	0.58
GSEA_BIOCARTA_ALK_PATHWAY.PMID.16199517	0.80	0.61	1.05	0.10	0.38
GSEA_BIOCARTA.AKT.PATHWAY.PMID.16199517	1.10	0.83	1.46	0.50	0.76
GSEA_BIOCARTA.BRCA.ATR.PATHWAY.ATRBRCA.PMID.16199517	1.22	0.94	1.60	0.14	0.43
GSEA_BIOCARTA.CASPASE.PATHWAY.PMID.16199517	1.09	0.84	1.43	0.52	0.78
GSEA_BIOCARTA.CTLA4.PATHWAY.PMID.16199517	1.33	1.00	1.80	0.05	0.37
GSEA_BIOCARTA.IGF1R.PATHWAY.PMID.16199517	0.78	0.58	1.04	0.09	0.38
GSEA_BIOCARTA.MTOR.PATHWAY.PMID.16199517	1.07	0.81	1.41	0.62	0.82
GSEA_BIOCARTA.PTEN.PATHWAY.PMID.16199517	0.74	0.55	0.98	0.04	0.37
GSEA_BIOCARTA.RAS.PATHWAY.PMID.16199517	0.99	0.74	1.32	0.95	>0.99
GSEA_BIOCARTA.RB.PATHWAY.PMID.16199517	1.26	0.97	1.66	0.09	0.38
GSEA_BIOCARTA.VEGF.PATHWAY.PMID.16199517	1.07	0.82	1.40	0.62	0.82
GSEA_HALLMARK.MYC.TARGETS.V1.PMID.16199517	0.96	0.72	1.27	0.77	0.92
GSEA_HELLER.HDAC.TARGETS.DOWN.PMID.16199517	1.32	0.98	1.80	0.07	0.37
GSEA_NELSON.RESPONSE.TO.ANDROGEN.UP.PMID.16199517	1.05	0.81	1.36	0.73	0.91
GSEA_REACTOME.PD1.SIGNALING.PMID.16199517	1.20	0.90	1.61	0.22	0.52

GSEA_REACTOME.PI3K.CASCADE.PMID.16199517	0.89	0.68	1.18	0.43	0.72
GSEA_RETINOL.METABOLISM.KEGG.PMID.16199517	0.74	0.52	1.05	0.10	0.38
GSEA.GP1_Proliferation.DNA.repair..PUJANA.CHEK2.PCC.NETWORK.PMID.25109877	1.24	0.95	1.64	0.12	0.41
GSEA.GP1_Proliferation.DNA.repair.REACTOME.CELL.CYCLE.MITOTIC.PMID.25109877	1.22	0.93	1.61	0.16	0.46
GSEA.GP10_Fatty.acid.oxidation.CARBOXYLIC.ACID.METABOLIC.PROCESS.PMID.25109877	0.86	0.65	1.12	0.26	0.55
GSEA.GP11_Immune.IFN.PerouLab.PMID.25109877	1.01	0.76	1.33	0.96	>0.99
GSEA.GP12_Hypoxia.glycolosis.SEMENZA.HIF1.TARGETS.PMID.25109877	1.13	0.87	1.48	0.36	0.65
GSEA.GP13_Neural.signailng.MODULE100.PMID.25109877	1.06	0.78	1.46	0.70	0.89
GSEA.GP13_Neural.signailng.NERVOUS.SYSTEM.DEVELOPMENT.PMID.25109877	0.95	0.72	1.26	0.73	0.91
GSEA.GP14_Plasma.membrane.cell.cell.signaling.MORF.CNTN1.PMID.25109877	1.07	0.84	1.36	0.59	0.79
GSEA.GP15_EGF.signaling.NAGASHIMA.EGF.SIGNALING.UP.PMID.25109877	1.05	0.82	1.35	0.71	0.90
GSEA.GP16_Protein.kinase.signaling.MAPKs.INTRACELLULAR.SIGNALING.CASCADE.PMID.25109877	1.20	0.89	1.64	0.23	0.53
GSEA.GP16_Protein.kinase.signaling.MAPKs.REGULATION.OF.KINASE.ACTIVITY.PMID.25109877	1.19	0.89	1.61	0.25	0.55
GSEA.GP17_Basal.signaling.SMID.BREAST.CANCER.BASAL.UP.PMID.25109877	1.20	0.85	1.71	0.30	0.58
GSEA.GP18_Vesicle.EPR.MEMBRANE.COAT.PMID.25109877	0.95	0.71	1.26	0.72	0.91
GSEA.GP19_1Q.amplicon.PerouLab.PMID.25109877	1.09	0.82	1.45	0.57	0.79
GSEA.GP2_Immune.Tcell.Bcell.KEGG.HEMATOPOIETIC.CELL.LINEAGE.PMID.25109877	1.32	0.98	1.79	0.07	0.37
GSEA.GP2_Immune.Tcell.Bcell.PerouLab.PMID.25109877	1.32	0.99	1.76	0.06	0.37
GSEA.GP20_TAL1.Leukemia.erythropoiesis.GNF2.TAL1.PMID.25109877	1.00	0.77	1.31	0.98	>0.99
GSEA.GP21_Anti.apoptosis.DNA.stability.MORF.BCL2.PMID.25109877	0.80	0.61	1.04	0.11	0.38
GSEA.GP21_Anti.apoptosis.DNA.stability.MORF.MT4.PMID.25109877	0.97	0.75	1.25	0.79	0.94
GSEA.GP21_Anti.apoptosis.DNA.stability.MORF.STK17A.PMID.25109877	0.79	0.60	1.02	0.08	0.37
GSEA.GP22_16Q22.24.amplicon.PerouLab.PMID.25109877	1.08	0.85	1.38	0.53	0.79
GSEA.GP3_Tumo.suppressing.miRNA.targets.GTTTGTT.MIR.495.PMID.25109877	0.98	0.73	1.31	0.87	0.96
GSEA.GP3_Tumor.suppressing.miRNA.targets.DACOSTA.UV.RESPONSE.VIA.ERCC3.DN.PMID.25109877	0.97	0.74	1.28	0.83	0.94
GSEA.GP3_Tumor.suppressing.miRNA.targets.TGCTTTG.MIR.330.PMID.25109877	1.05	0.79	1.39	0.74	0.91
GSEA.GP4_MES.ECM.PerouLab.PMID.25109877	0.97	0.72	1.29	0.82	0.94
GSEA.GP5_MYC.targets.TERT.PerouLab.PMID.25109877	1.11	0.86	1.44	0.41	0.71
GSEA.GP6_Squamous.differentiation.development.RICKMAN.TUMOR.DIFFERENTIATED.WELL.VS.POORLY.DN.PMID.25109877	1.21	0.90	1.62	0.21	0.51
GSEA.GP7_Estrogen.signaling.SMID.BREAST.CANCER.BASAL.DN.PMID.25109877	0.75	0.52	1.06	0.11	0.38
GSEA.GP8_FOXO.stemness.MORF.PTPRB.PMID.25109877	0.82	0.63	1.07	0.15	0.46
GSEA.GP8_FOXO.stemness.TTGTTT.VSFOXO4.01.PMID.25109877	0.99	0.76	1.28	0.93	0.99
GSEA.GP9_Cell.cell.adhesion.PerouLab.PMID.25109877	1.02	0.72	1.44	0.93	0.99
HCK_BCR.2008.PMID.19272155	1.29	0.98	1.73	0.08	0.37
HER1.Cluster1_BMC.Genomics.2007.PMID.17663798	1.13	0.88	1.46	0.34	0.63
HER1.Cluster2_BMC.Genomics.2007.PMID.17663798	1.14	0.87	1.49	0.35	0.64
HER1.Cluster3_BMC.Genomics.2007.PMID.17663798	1.09	0.85	1.41	0.49	0.76
HER2.Amplicon.PerouLab_BMC.Med.Genomic.2011.PMID.21214954	1.28	0.98	1.68	0.07	0.37
Histological.Grade_J.Pathol.2017.PMID.27861902	1.36	1.00	1.88	0.06	0.37
HouseKeeping_Genome.Biol.2004.PMID.15287981	1.00	0.77	1.31	0.99	>0.99
iDC.Median_Immunity.2013.PMID.24138885	1.20	0.90	1.61	0.23	0.53
IFN.Cluster_BMC.Med.Genomics.2011.PMID.21214954	0.87	0.66	1.14	0.32	0.60
IgG_BCR.2008.PMID.19272155	1.18	0.88	1.61	0.28	0.56
IGG.Cluster_BMC.Med.Genomics.2011.PMID.21214954	1.20	0.89	1.63	0.23	0.53
Immature..B.cell_CellRep.2017.PMID.28052254	1.19	0.88	1.60	0.26	0.55
Immature.dendritic.cell_CellRep.2017.PMID.28052254	0.86	0.64	1.14	0.30	0.58
ImmLandscape_Macro.mono.CSF1.core.response_CCR.2009.PMID.29628290	1.33	0.98	1.81	0.07	0.37

ImmLandscape_Wound.Healing_Immunity.2018.PMID.29628290	1.32	1.00	1.76	0.05	0.37
ImmLandscape.IFN3_Plos.One.2014.PMID.24516633	0.84	0.63	1.10	0.21	0.52
ImmLandscape.IFNG5_Plos.One.2014.PMID.24516633	1.42	1.05	1.94	0.02	0.37
ImmLandscape.lymphocyte.Infil.T.B.PMID.18592372	1.28	0.96	1.72	0.09	0.38
Immune.Hot.CD8.vs.Cold_Nature.2020.PMID.31942071	1.25	0.94	1.68	0.13	0.43
Immune.Perez.14_JCO.2015.PMID.25605861	1.17	0.88	1.58	0.28	0.56
Immune.Perez.87_JCO.2015.PMID.25605861	1.34	1.01	1.81	0.05	0.37
Immune.Suppression_JCI.Insight.2016.PMID.27699256	1.36	1.02	1.85	0.04	0.37
ImmuneActive_Cell.2019.PMID.31730857	1.35	1.00	1.83	0.05	0.37
Immunosuppression.PMID.31942077	1.22	0.93	1.60	0.15	0.45
IMS.Score_CCR.2018.PMID.29921729	0.90	0.67	1.20	0.46	0.75
Induced.in.Bcells_PNAS.2013.PMID.23382184	1.04	0.80	1.37	0.76	0.92
Induced.in.DC_PNAS.2013.PMID.23382184	1.36	1.02	1.83	0.04	0.37
Induced.in.GN_PNAS.2013.PMID.23382184	1.15	0.85	1.57	0.36	0.66
Induced.in.HSC_PNAS.2013.PMID.23382184	1.13	0.87	1.47	0.38	0.67
Induced.in.MOs_PNAS.2013.PMID.23382184	1.27	0.93	1.75	0.13	0.43
Induced.in.NKcells_PNAS.2013.PMID.23382184	1.19	0.88	1.61	0.26	0.55
Induced.in.Tcells_PNAS.2013.PMID.23382184	1.15	0.87	1.51	0.33	0.61
Inflammatory.breast.cancer.491genes_CCR.2013.PMID.23396049	1.35	1.01	1.82	0.04	0.37
Inflammatory.breast.cancer.79genes_CCR.2013.PMID.23396049	1.26	0.95	1.67	0.11	0.39
Inflammatory.breast.cancer.expressed.noIBC_79genes_CCR.2013.PMID.23396049	0.96	0.73	1.28	0.78	0.93
Inflammatory.breast.cancer.expressed.noIBC.491genes_CCR.2013.PMID.23396049	0.85	0.63	1.14	0.28	0.56
Influenza.11genes.Metasignature_Immunity.2015.PMID.26682989	0.88	0.66	1.16	0.37	0.66
Interferon_BCR.2008.PMID.19272155	0.82	0.62	1.09	0.18	0.47
Interferon.Pathway_CancerImmunolRes.2018.PMID.30266715	0.94	0.72	1.23	0.66	0.86
JUND.KRT5_Nat.Cell.Biol.2014.PMID.24658685	1.00	0.78	1.28	0.98	>0.99
Keller2012.CD10.Adam_BCR.2015.PMID.25575446	1.10	0.80	1.52	0.55	0.79
KRAS.amplicon_Genome.Biology.2007.PMID.17493263	1.10	0.85	1.42	0.48	0.76
Late.IRS.1_PLoS.One.2016.PMID.26991655	1.09	0.81	1.46	0.56	0.79
Late.IRS.2_PLoS.One.2016.PMID.26991655	0.92	0.71	1.20	0.54	0.79
LCK_BCR.2008.PMID.19272155	1.32	0.99	1.78	0.06	0.37
Lim2009.LumProg.Adam_BCR.2015.PMID.25575446	0.96	0.70	1.31	0.80	0.94
Lim2009.MaSC.Adam_BCR.2015.PMID.25575446	0.92	0.68	1.25	0.58	0.79
Lim2009.MatureLum.Adam_BCR.2015.PMID.25575446	0.67	0.45	0.98	0.04	0.37
Lim2009.Stroma.Adam_BCR.2015.PMID.25575446	0.96	0.69	1.34	0.81	0.94
Lim2010.LumProg.Adam_BCR.2015.PMID.25575446	1.11	0.84	1.46	0.47	0.75
Lim2010.MaSC.Adam_BCR.2015.PMID.25575446	0.99	0.72	1.36	0.97	>0.99
Lim2010.MatureLum.Adam_BCR.2015.PMID.25575446	0.82	0.63	1.07	0.15	0.45
Lim2010.Stroma.Adam_BCR.2015.PMID.25575446	0.90	0.66	1.23	0.51	0.77
Lobular.Carcinoma.In.Situ_J.Pathol.2017.PMID.27861902	0.99	0.74	1.31	0.94	0.99
LOBULAR.TCGA.SIGNATURE.ImmuneCell.2015.PMID.26451490	1.50	1.09	2.09	0.02	0.29
LOBULAR.TCGA.SIGNATURE.Reactive_Cell.2015.PMID.26451490	0.89	0.63	1.24	0.48	0.76
LOBULAR.TCGA.SUBTYPE.Immune_Cell.2015.PMID.26451490	1.36	0.94	1.98	0.11	0.38
LOBULAR.TCGA.SUBTYPE.Proliferative_Cell.2015.PMID.26451490	1.10	0.79	1.55	0.57	0.79
LOBULAR.TCGA.SUBTYPE.Reactive_Cell.2015.PMID.26451490	0.96	0.70	1.31	0.82	0.94
LTS.score_JCI.2020.PMID.32573490	0.71	0.50	0.98	0.04	0.37
Luminal_Progenitor_Up_Nat.Med.2009.PMID.19648928	1.05	0.75	1.47	0.78	0.93

Luminal.cluster_BMC.Med.Genomics.2011.PMID.21214954	0.83	0.60	1.16	0.28	0.56
Luminal.Progenitor_BCR.2010.PMID.20346151	1.03	0.75	1.43	0.85	0.95
Luminal.Progenitor.Down_Nat.Med.2009.PMID.19648928	0.97	0.72	1.31	0.84	0.94
LumProg.HsEnriched_BCR.2015.PMID.25575446	0.99	0.72	1.36	0.96	>0.99
LumProg.HsEnriched.Refined1_BCR.2015.PMID.25575446	0.76	0.54	1.05	0.10	0.38
LumProg.Lim09_BCR.2015.PMID.25575446	1.08	0.81	1.45	0.58	0.79
LumProg.Prat_BCR.2015.PMID.25575446	0.99	0.72	1.34	0.93	0.99
LumProg.Shehata_BCR.2015.PMID.25575446	0.93	0.72	1.21	0.57	0.79
Lums.HER2E.DOWN.metastatic.signature_JCI.2020.PMID.32573490	0.69	0.51	0.93	0.02	0.29
Lums.HER2E.UP.metastatic.signature_JCI.2020.PMID.32573490	1.23	0.95	1.63	0.13	0.42
Lung.WNT_Cancer.Res.2009.PMID.19549913	1.27	0.97	1.68	0.08	0.37
Lymph.vessels_Immunity.2013.PMID.24138885	1.10	0.82	1.47	0.54	0.79
Lymphovascular.Invasion_J.Pathol.2017.PMID.27861902	1.21	0.92	1.62	0.18	0.47
M.D.Metagene_Genome.Biol.2013.PMID.23618380	1.16	0.88	1.54	0.31	0.59
M2.Macrophage_Blood.2006.PMID.16556895	1.14	0.86	1.52	0.37	0.66
Macrophage_CellRep.2017.PMID.28052254	0.98	0.73	1.33	0.92	0.98
Macrophages_CancerImmunolRes.2018.PMID.30266715	1.25	0.94	1.69	0.14	0.43
Macrophages_Immunity.2013.PMID.24138885	1.08	0.81	1.46	0.58	0.79
Macrophages.M0_Nat.Methods.2015.PMID.25822800	1.37	1.01	1.89	0.04	0.37
Macrophages.M1_Nat.Methods.2015.PMID.25822800	1.33	0.99	1.80	0.06	0.37
Macrophages.M2_Nat.Methods.2015.PMID.25822800	1.44	1.05	2.00	0.03	0.37
MacTh1.cluster_CCR.2014.PMID.24916698	1.33	0.99	1.80	0.06	0.37
MammaPrint_Nature.2002.PMID.11823860	0.68	0.49	0.93	0.02	0.29
MAPK.pathway.activation_NPJ.Precis.Oncol.2018.PMID.29872725	1.45	1.08	1.96	0.01	0.29
MASC.Down_Nat.Med.2009.PMID.19648928	0.83	0.63	1.09	0.17	0.46
MASC.Up_Nat.Med.2009.PMID.19648928	1.01	0.74	1.40	0.93	0.99
Mast.cell_CellRep.2017.PMID.28052254	1.19	0.88	1.63	0.26	0.55
Mast.cells_Immunity.2013.PMID.24138885	0.79	0.58	1.08	0.15	0.45
Mast.cells.activated_Nat.Methods.2015.PMID.25822800	0.91	0.69	1.21	0.53	0.79
Mast.cells.resting_Nat.Methods.2015.PMID.25822800	0.95	0.70	1.29	0.74	0.91
Mature.luminal_BCR.2010.PMID.20346151	0.83	0.59	1.17	0.29	0.57
Mature.Luminal.Down_Nat.Med.2009.PMID.19648928	1.23	0.87	1.75	0.25	0.55
Mature.LuminaUp_Nat.Med.2009.PMID.19648928	0.70	0.49	1.00	0.05	0.37
MatureLum.HsEnriched_BCR.2015.PMID.25575446	0.68	0.47	0.96	0.03	0.37
MatureLum.HsEnriched.Refined1_BCR.2015.PMID.25575446	0.81	0.56	1.16	0.25	0.55
MatureLum.Lim09_BCR.2015.PMID.25575446	0.70	0.49	0.99	0.04	0.37
MatureLum.Prat_BCR.2015.PMID.25575446	0.83	0.60	1.16	0.28	0.56
MatureLum.Shehata_BCR.2015.PMID.25575446	0.87	0.62	1.21	0.41	0.71
MBasal.Cluster_BMC.Med.Genomics.2011.PMID.21214954	0.98	0.70	1.37	0.88	0.97
MCD3.CD8_BMC.Med.Genomics.2011.PMID.21214954	1.25	0.95	1.66	0.12	0.39
MCF7.E2.induced.genes_JCO.2006.PMID.16505416	0.90	0.68	1.19	0.47	0.75
MCF7.E2.repressed.genes_JCO.2006.PMID.16505416	0.85	0.63	1.16	0.31	0.59
MDSC_CellRep.2017.PMID.28052254	1.41	1.03	1.95	0.03	0.37
MDSC.Granulocytic_Leukoc.Biol.2012.PMID.21954284	1.08	0.83	1.43	0.56	0.79
MDSC.Neutrophi_Leukoc.Biol.2012.PMID.21954284	1.35	0.98	1.88	0.07	0.37
MDSC.tumor_J.Immunol.2012.PMID.23152559	1.13	0.84	1.52	0.44	0.72
MDSC.tumor.MO_J.Immunol.2012.PMID.23152559	1.41	1.04	1.92	0.03	0.37

MECM_BMC.Med.Genomics.2011.PMID.21214954	1.03	0.75	1.43	0.85	0.95
Memory.B.cell_CellRep.2017.PMID.28052254	1.09	0.81	1.49	0.56	0.79
MET.DOWN.RNAseq.Significant.Genes_JCI.2018.PMID.29480819	0.92	0.73	1.17	0.49	0.76
MET.DOWN.Significant.Genes.Low.Basal.1_JCI.2018.PMID.29480819	1.04	0.78	1.39	0.80	0.94
MET.DOWN.Significant.Genes.Low.Basal.2_JCI.2018.PMID.29480819	0.86	0.66	1.12	0.25	0.55
MET.UP.RNAseq.Significant.Genes_JCI.2018.PMID.29480819	1.02	0.78	1.32	0.90	0.97
MET.UP.Significant.Genes.HIGH.BASALS.Genes_JCI.2018.PMID.29480819	0.84	0.63	1.10	0.20	0.51
Metaplastic.Up_CanRes.2009.PMID.19435916	1.01	0.72	1.40	0.97	>0.99
Metastasis.predictor.TNBC_BCR.2010.PMID.20946665	1.27	0.98	1.67	0.08	0.37
MFGFR2_BMC.Med.Genomics.2011.PMID.21214954	1.12	0.85	1.48	0.43	0.72
MHC.Forero.11_Cancer.Immunol.Res.2016.PMID.26980599	1.11	0.83	1.48	0.47	0.75
MHC.Forero.24_Cancer.Immunol.Res.2016.PMID.26980599	1.10	0.86	1.42	0.44	0.72
MHC.I_BCR.2008.PMID.19272155	1.11	0.84	1.45	0.47	0.75
MHC.II_BCR.2008.PMID.19272155	1.27	0.96	1.69	0.10	0.38
MHCI.coreGenes_Nat.Commun.2017.PMID29170503	1.18	0.90	1.56	0.24	0.54
MIR200c.Induced_ONCO.2015.PMID.25746005	1.00	0.77	1.31	0.98	>0.99
MIR200c.Repressed_ONCO.2015.PMID.25746005	1.04	0.77	1.41	0.81	0.94
miRNA.138.signature_Cancer.Res.2014.PMID.25339353	1.17	0.90	1.52	0.24	0.54
MITO1_BMC.Med.Genomics.2011.PMID.21214954	1.12	0.90	1.41	0.30	0.59
MITO2_BMC.Med.Genomics.2011.PMID.21214954	0.92	0.74	1.15	0.49	0.76
Mitotic.Count_J.Pathol.2017.PMID.27861902	1.20	0.90	1.62	0.21	0.52
MK14.K17_BMC.Med.Genomics.2011.PMID.21214954	0.91	0.67	1.23	0.53	0.79
MKRAS.amplicon_BMC.Med.Genomics.2011.PMID.21214954	1.10	0.86	1.42	0.43	0.72
MM.BRCAwnt.1pFDR.UP_Genome.Biology.2007.PMID.17493263	1.10	0.84	1.46	0.50	0.76
MM.C3Tag.1pFDR.UP_Genome.Biology.2007.PMID.17493263	1.33	0.99	1.80	0.06	0.37
MM.C3Tag.2012_Genome.Biol.2013.PMID.24220145	1.29	0.95	1.78	0.11	0.39
MM.Class3_Genome.Biol.2013.PMID.24220145	1.38	1.04	1.85	0.03	0.37
MM.Class8_Genome.Biol.2013.PMID.24220145	1.03	0.78	1.37	0.82	0.94
MM.Claudinlow_Genome.Biol.2013.PMID.24220145	1.09	0.80	1.50	0.58	0.79
MM.DMBAwnt.1pFDR.UP.Genome.Biology.2007.PMID.17493263	1.00	0.75	1.33	0.99	>0.99
MM.Erbb2.like_Genome.Biol.2013.PMID.24220145	0.83	0.63	1.09	0.18	0.46
MM.Myc.2012_Genome.Biol.2013.PMID.24220145	1.20	0.92	1.57	0.18	0.47
MM.Myoepithelioma.like_Genome.Biol.2013.PMID.24220145	1.00	0.74	1.35	>0.99	>0.99
MM.Neu.2012_Genome.Biol.2013.PMID.24220145	1.07	0.80	1.44	0.65	0.85
MM.NeuPyMT.1pFDR.UP_Genome.Biology.2007.PMID.17493263	0.91	0.72	1.15	0.42	0.71
MM.Normal.1pFDR.UP_Genome.Biology.2007.PMID.17493263	1.00	0.70	1.44	>0.99	>0.99
MM.Normal.like_Genome.Biol.2013.PMID.24220145	1.18	0.79	1.77	0.42	0.71
MM.p53null.1pFDR.UP_Genome.Biology.2007.PMID.17493263	1.12	0.85	1.49	0.42	0.71
MM.p53null.Basal_Genome.Biol.2013.PMID.24220145	1.04	0.78	1.40	0.77	0.93
MM.p53null.Luminal_Genome.Biol.2013.PMID.24220145	1.09	0.84	1.40	0.53	0.79
MM.Potluck_1pFDR_UP_Genome.Biology.2007_PMID.17493263.PMID.24220145	1.16	0.84	1.60	0.38	0.67
MM.PyMT.2012_Genome.Biol.2013.PMID.24220145	1.04	0.82	1.33	0.75	0.91
MM.Squamous.like_Genome.Biol.2013.PMID.24220145	1.17	0.87	1.58	0.31	0.59
MM.Stat1_Genome.Biol.2013.PMID.24220145	0.94	0.70	1.24	0.65	0.84
MM.WapINT3.1pFDR.UP_Genome.Biology.2007.PMID.17493263	1.21	0.93	1.59	0.16	0.46
MM.WapINT3.2012_Genome.Biol.2013.PMID.24220145	0.98	0.75	1.27	0.87	0.96
MM.WAPTag.1pFDR.UP_Genome.Biology.2007.PMID.17493263	1.27	0.94	1.74	0.13	0.42

MM.Wnt1.Early_Genome.Biol.2013.PMID.24220145	1.08	0.80	1.47	0.61	0.82
MM.Wnt1.Late_Genome.Biol.2013.PMID.24220145	0.97	0.73	1.28	0.82	0.94
Mmyosin_BMC.Med.Genomics.2011.PMID.21214954	1.17	0.89	1.56	0.28	0.56
MNADH_CYTochrome_BMC.Med.Genomics.2011.PMID.21214954	1.09	0.87	1.38	0.45	0.74
MNB1_BMC.Med.Genomics.2011.PMID.21214954	0.93	0.70	1.23	0.61	0.81
MNB2_BMC.Med.Genomics.2011.PMID.21214954	1.34	0.90	2.02	0.15	0.45
MNB3_BMC.Med.Genomics.2011.PMID.21214954	0.84	0.55	1.17	0.37	0.66
MNOtch4_BMC.Med.Genomics.2011.PMID.21214954	1.01	0.77	1.32	0.97	>0.99
Monocyte_CellRep.2017.PMID.28052254	0.97	0.75	1.27	0.85	0.95
Monocyte..DC.25gene_Genome.Biol.2013.PMID.23618380	1.18	0.89	1.57	0.26	0.55
Monocytes_CancerImmunolRes.2018.PMID.30266715	1.27	0.96	1.69	0.10	0.38
Monocytes_Nat.Methods.2015.PMID.25822800	1.24	0.92	1.68	0.17	0.46
Monocytic.lineage.MCP_Nature.2020.PMID.31942075	0.99	0.72	1.34	0.93	0.98
MProliferation_BMC.Med.Genomics.2011.PMID.21214954	1.34	0.99	1.85	0.07	0.37
MProtocadherin_BMC.Med.Genomics.2011.PMID.21214954	0.94	0.70	1.27	0.71	0.90
MPYMT_NEU_Cluster_BMC.Med.Genomics.2011.PMID.21214954	0.89	0.68	1.16	0.39	0.67
MRibosomal_BMC.Med.Genomics.2011.PMID.21214954	1.10	0.88	1.38	0.42	0.71
MS.CD44.DOWN_PNAS.2009.PMID.19666588	0.92	0.67	1.26	0.58	0.79
MS.CD44.UP_PNAS.2009.PMID.19666588	1.14	0.86	1.53	0.36	0.65
MSquamous_BMC.Med.Genomics.2011.PMID.21214954	0.98	0.74	1.31	0.92	0.98
Murat.G07_JCO.2008.PMID.18565887	1.14	0.84	1.56	0.40	0.68
Murat.G18_JCO.2008.PMID.18565887	0.70	0.52	0.92	0.01	0.29
Murat.G24_JCO.2008.PMID.18565887	1.33	0.98	1.84	0.07	0.37
MVEGFC_BMC.Med.Genomics.2011.PMID.21214954	0.77	0.56	1.03	0.08	0.37
Myeloid.cell.chemotaxis.1gene_Nature.2020.PMID.31942077	0.98	0.75	1.28	0.89	0.97
Myeloid.dendritic.cells.MCP_Nature.2020.PMID.31942077	1.16	0.89	1.52	0.27	0.56
Natural.killer.cell_CellRep.2017.PMID.28052254	0.92	0.71	1.19	0.50	0.77
Natural.killer.T.cell_CellRep.2017.PMID.28052254	1.14	0.84	1.54	0.41	0.70
Necrosis_J.Pathol.2017.PMID.27861902	1.24	0.95	1.64	0.11	0.39
Neutrophil_CellRep.2017.PMID.28052254	0.90	0.66	1.23	0.51	0.77
Neutrophils_CancerImmunolRes.2018.PMID.30266715	1.37	1.03	1.86	0.04	0.37
Neutrophils_Immunity.2013.PMID.24138885	1.59	1.13	2.28	0.01	0.29
Neutrophils_Nat.Methods.2015.PMID.25822800	1.22	0.89	1.69	0.22	0.52
Neutrophils.MCP_Nature.2020.PMID.31942077	0.84	0.62	1.14	0.26	0.55
NK_Immunity.2013.PMID.24138885	1.10	0.83	1.47	0.50	0.76
NK.activated_Nat.Methods.2015.PMID.25822800	1.30	0.98	1.74	0.07	0.37
NK.CD56bright_Immunity.2013.PMID.24138885	1.24	0.97	1.59	0.09	0.38
NK.CD56dim_Immunity.2013.PMID.24138885	1.47	1.08	2.02	0.02	0.29
NK.resting_Nat.Methods.2015.PMID.25822800	1.30	0.98	1.74	0.07	0.37
NKcells_CancerImmunolRes.2018.PMID.30266715	1.29	0.97	1.74	0.09	0.38
NKcells.MCP_Nature.2020.PMID.31942077	0.98	0.72	1.32	0.87	0.96
No.Response.Immunotherapy.TLS.Melanoma_Nature.2020.PMID.31942075	0.92	0.68	1.23	0.57	0.79
Normal.mucosa_Immunity.2013.PMID.24138885	1.00	0.73	1.36	>0.99	>0.99
Nuclear.Pleomorphism_J.Pathol.2017.PMID.27861902	0.85	0.64	1.13	0.26	0.55
Oncotype_NEJM.2004.PMID.15591335	1.92	1.38	2.73	<0.001	0.02
P53.ERPos.MDACC_CCR.2011.PMID.21248301	1.34	0.96	1.89	0.09	0.38
Parity.signature.251genes_BCR.2014.PMID.25005139	1.16	0.86	1.58	0.33	0.61

Parity.signature.40genes_BCR.2014.PMID.25005139	1.30	0.96	1.76	0.09	0.38
PARPi.Resistance_BCRT_2012.PMID.22875744	1.20	0.92	1.57	0.19	0.49
PARPi.Sensitivity_BCRT_2012.PMID.22875744	0.89	0.67	1.17	0.39	0.68
PARPi.Sensitivity.MDACC_NPJ.Syst.Biol.Appl.2017.PMID.28649435	1.15	0.88	1.52	0.30	0.58
PARPi.Sensitivity.Negative_Sci.Adv.2017.PMID.28439535	1.25	0.96	1.64	0.10	0.38
PARPi.Sensitivity.Positive_Sci.Adv.2017.PMID.28439535	1.08	0.81	1.44	0.60	0.80
Pcorr.Breast2Lung.LM2.Correlation_Nature.2005.PMID.16049480	1.03	0.78	1.37	0.82	0.94
Pcorr.Breast2Lung.Parental.Correlation_Nature.2005.PMID.16049480	0.93	0.70	1.24	0.63	0.83
Pcorr.dasatinib.resistant_Cancer.Res.2007.PMID.17332353	1.03	0.75	1.43	0.84	0.94
Pcorr.dasatinib.sensitive_Cancer.Res.2007.PMID.17332353	0.94	0.68	1.31	0.72	0.91
Pcorr.Hypoxia.High.Correlation_PLoS.Med.2006.PMID.16417408	1.10	0.84	1.46	0.48	0.76
Pcorr.Hypoxia.Low.Correlation_PLoS.Med.2006.PMID.16417408	0.87	0.66	1.15	0.33	0.61
Pcorr.IGS_Invasiveness_NJEM.2007.PMID.17229949	1.32	0.96	1.84	0.10	0.38
Pcorr.wound.response.activated_PNAS.2005.PMID.15701700	1.28	0.97	1.70	0.09	0.38
pCR.predictor.ERNeg.55genes_JAMA.2011.PMID.21558518	1.06	0.81	1.40	0.66	0.86
pCR.predictor.ERPos.39genes_JAMA.2011.PMID.21558518	1.29	0.96	1.74	0.09	0.38
PDCD1_Single_Gene.Single	1.29	0.99	1.71	0.06	0.37
Pfefferle2012.LumProg_BCR.2015.PMID.25575446	0.99	0.72	1.37	0.95	>0.99
Pfefferle2012.MaSC_BCR.2015.PMID.25575446	1.03	0.76	1.41	0.84	0.94
Pfefferle2012.MatureLum_BCR.2015.PMID.25575446	0.86	0.60	1.23	0.41	0.71
Pfefferle2012.Stroma_BCR.2015.PMID.25575446	1.00	0.72	1.38	0.99	>0.99
PGR_Single_Gene.Single	0.47	0.31	0.68	<0.001	0.02
PI3Ki.Down_CancerCell.2017.PMID.28528867	1.22	0.94	1.58	0.14	0.43
PI3Ki.Up_CancerCell.2017.PMID.28528867	0.95	0.72	1.25	0.73	0.91
PIK3CA.Pathway_Ann.Oncol.2017.PMID.28177460	1.04	0.78	1.40	0.77	0.93
PIK3CAmt.signature_Cancer.Res.2012.PMID.22552288	1.22	0.92	1.63	0.17	0.46
Plasma.cells_Nat.Methods.2015.PMID.25822800	1.17	0.88	1.58	0.28	0.56
PlasmaCells_CancerImmunolRes.2018.PMID.30266715	1.21	0.90	1.64	0.22	0.52
Plasmacytoid.dendritic.cell_CellRep.2017.PMID.28052254	0.92	0.69	1.22	0.57	0.79
PR.Isoform.Ratio.Up.in.PRA.H_JNCI.2017.PMID.28376177	0.82	0.61	1.10	0.20	0.50
PR.Isoform.Ratio.Up.in.PRB.H_JNCI.2017.PMID.28376177	1.17	0.87	1.59	0.31	0.59
Proliferation.Cluster_BMC.Med.Genomics.2011.PMID.21214954	1.36	0.99	1.91	0.06	0.37
Proliferation.Metagene_Genome.Biol.2013.PMID.23618380	1.40	1.01	1.97	0.05	0.37
Proliferation.score.PAM50_JCO.2009.PMID.19204204	1.50	1.07	2.13	0.02	0.36
ProliferationPathway_CancerImmunolRes.2018.PMID.30266715	1.40	1.01	1.97	0.05	0.37
Prosigna.Proliferation.18_BMC.Med.Genomics.2015.PMID.26297356	1.34	0.97	1.87	0.08	0.37
Race.LuminalA.MRE.score_BCRT.2015.PMID.26109344	1.08	0.81	1.44	0.59	0.80
Radiation.induced.genes_Radoat.Res.2014.PMID.24527691	1.13	0.83	1.56	0.43	0.72
RB.LOH_BCR.2008.PMID.18782450	1.27	0.94	1.74	0.12	0.39
RB.LOSS_JCI.2007.PMID.17160137	1.35	0.99	1.85	0.06	0.37
Regulatory.T.cell_CellRep.2017.PMID.28052254	1.10	0.83	1.46	0.51	0.77
Replication.Stress.Down.set_Cell.Rep.2018.PMID.29768207	0.93	0.71	1.21	0.58	0.79
Replication.Stress.Model_Cell.Rep.2018_PMID.29768207.PMID.29768207	1.14	0.85	1.55	0.38	0.66
Replication.Stress.Neg_Cell.Rep.2018_PMID.29768207.PMID.29768207	0.98	0.75	1.27	0.86	0.96
Replication.Stress.Pos_Cell.Rep.2018_PMID.29768207.PMID.29768207	0.97	0.73	1.29	0.84	0.94
Replication.Stress.Up_Set_Cell.Rep.2018_PMID.29768207.PMID.29768207	0.99	0.75	1.31	0.93	0.99
Residual.disease.predictor.ERNeg.54genes_JAMA.2011.PMID.21558518	0.98	0.75	1.28	0.87	0.96

Residual.disease.predictor.ERPos.73genes_JAMA.2011.PMID.21558518	1.09	0.83	1.45	0.54	0.79
Response.Immunotherapy.MCP.TLS.Melanoma_Nature.2020.PMID.31942075	1.33	0.98	1.84	0.07	0.37
Response.Immunotherapy.signature_Science.2018.PMID.30309915	1.35	1.01	1.82	0.05	0.37
Response.Neo.Chemo_common_CCR.2014.PMID.25047707	1.32	0.96	1.83	0.10	0.38
Response.Neo.Chemo_ERNeg_CCR.2014.PMID.25047707	1.16	0.89	1.52	0.26	0.55
Response.Neo.Chemo_ERPos_CCR.2014.PMID.25047707	0.99	0.74	1.33	0.95	>0.99
RHOA.pathway_Ann.Oncol.2017.PMID.28177460	0.86	0.65	1.11	0.25	0.55
Ribosomal.Cluster_BMC.Med.Genomics.2011.PMID.21214954	1.07	0.85	1.35	0.57	0.79
ROR.subtype.PAM50_JCO.2009.PMID.19204204	2.21	1.47	3.43	<0.001	0.02
ROR.subtype.proliferation.PAM50_JCO.2009.PMID.19204204	1.69	1.18	2.47	0.005	0.20
RSS.Score_CCR.2018.PMID.29921729	1.10	0.84	1.45	0.49	0.76
S100A9.A8_BMC.Med.Genomics.2011.PMID.21214954	1.42	1.01	2.02	0.05	0.37
Scorr.EMAT1.Correlation_BCR.2020.PMID.32641077	1.19	0.88	1.62	0.25	0.55
Scorr.EMAT2.Correlation_BCR.2020.PMID.32641077	0.80	0.59	1.07	0.14	0.43
Scorr.EMAT3.Correlation_BCR.2020.PMID.32641077	0.81	0.59	1.10	0.18	0.46
Scorr.EMAT4.Correlation_BCR.2020.PMID.32641077	1.32	0.94	1.89	0.12	0.39
Scorr.IE.Correlation_JCO.2006.PMID.16505416	0.53	0.36	0.75	0.001	0.03
Scorr.IIE.Correlation_JCO.2006.PMID.16505416	1.89	1.34	2.75	0.001	0.03
Scorr.PAM50.Basal_JCO.2009.PMID.19204204	1.69	1.14	2.54	0.01	0.29
Scorr.PAM50.Her2_JCO.2009.PMID.19204204	2.16	1.46	3.30	<0.001	0.02
Scorr.PAM50.LumA_JCO.2009.PMID.19204204	0.45	0.29	0.67	<0.001	0.02
Scorr.PAM50.LumB_JCO.2009.PMID.19204204	1.17	0.87	1.60	0.31	0.59
Scorr.PAM50.Normal_JCO.2009.PMID.19204204	0.69	0.49	0.97	0.04	0.37
Scorr.S329.L_Br.J.Cancer.2008.PMID.18382427	0.89	0.66	1.19	0.43	0.72
Scorr.S329.R_Br.J.Cancer.2008.PMID.18382427	1.04	0.78	1.38	0.80	0.94
Secretoglobin_BMC.Med.Genomics.2011.PMID.21214954	1.08	0.83	1.42	0.58	0.79
Shehata2012.ALDHneg_BCR.2015.PMID.25575446	0.87	0.66	1.14	0.32	0.60
Shehata2012.ALDHpos_BCR.2015.PMID.25575446	1.03	0.74	1.45	0.84	0.94
Shehata2012.Basal_BCR.2015.PMID.25575446	0.97	0.70	1.33	0.83	0.94
Shehata2012.Erbb3neg_BCR.2015.PMID.25575446	0.74	0.57	0.97	0.03	0.37
Shehata2012.LumProg_BCR.2015.PMID.25575446	1.04	0.77	1.41	0.80	0.94
Shehata2012.NCL_BCR.2015.PMID.25575446	0.80	0.57	1.14	0.22	0.52
Shehata2012.Stroma_BCR.2015.PMID.25575446	1.02	0.74	1.41	0.92	0.98
Spike2012.aMaSC_BCR.2015.PMID.25575446	0.98	0.74	1.31	0.92	0.98
Spike2012.fMaSC_BCR.2015.PMID.25575446	0.97	0.73	1.29	0.84	0.94
Spike2012.fStr_BCR.2015.PMID.25575446	0.99	0.73	1.35	0.96	>0.99
STAT1_BCR.2008.PMID.19272155	1.20	0.90	1.62	0.22	0.52
STAT3.Basal_PNAS.2014.PMID.25139989	1.20	0.90	1.60	0.21	0.52
STAT3.Basal.short_PNAS.2014.PMID.25139989	1.24	0.92	1.68	0.15	0.46
Stroma.FNA.MDACC.1_JCO.2010.PMID.20805453	1.35	1.01	1.83	0.05	0.37
Stroma.FNA.MDACC.2_JCO.2010.PMID.20805453	1.06	0.79	1.43	0.69	0.89
Stromal.Central.Fibrotic.Focus_J.Pathol.2017.PMID.27861902	0.86	0.66	1.13	0.29	0.58
Stromal.Down_Nat.Med.2009.PMID.19648928	1.03	0.75	1.43	0.84	0.94
Stromal.Inflammation_J.Pathol.2017.PMID.27861902	1.44	1.05	1.99	0.02	0.37
Stromal.Signature_Nat.Med.2008.PMID.18438415	1.19	0.90	1.60	0.23	0.53
Stromal.Up_Nat.Med.2009.PMID.19648928	1.01	0.73	1.40	0.95	>0.99
SW480.cancer.cells_Immunity.2013.PMID.24138885	0.97	0.72	1.29	0.81	0.94

T.follicular.helper.cell_CellRep.2017.PMID.28052254	1.14	0.84	1.56	0.39	0.68
Tcell.activation_Nature.2020.PMID.31942077	1.12	0.82	1.53	0.49	0.76
Tcell.CD8.Effector.vs.naive.2_Science.2016.PMID27789795	1.38	1.00	1.94	0.06	0.37
Tcell.CD8.Exhausted.vs.antiPDL1.2_Science.2016.PMID27789795	1.52	1.09	2.17	0.02	0.30
Tcell.CD8.Exhausted.vs.naive.2_Science.2016.PMID27789795	1.36	0.99	1.91	0.06	0.37
Tcell.CD8.Memory.vs.naive.1_Science.2016.PMID27789795	1.44	1.08	1.96	0.02	0.29
Tcell.cluster_CCR.2014.PMID.24916698	1.30	0.98	1.75	0.08	0.37
Tcell.EXH.Anti.PDL1.vs.control.treated.exhausted.CD8.Tcell.Metagene.1.Science.2016.PMID.27789795	1.03	0.78	1.36	0.83	0.94
Tcell.EXH.Effector.CD8.T.cell.at.day.8.p.i.Armstrong.vs.Naive.CD8.Tcell.Metagene.1_Science.2016.PMID.27789795	1.19	0.88	1.64	0.27	0.55
Tcell.EXH.Exhausted.CD8.T.cell.vs.Naive.CD8.T.cell.Metagene.1_Science.2016.PMID.27789795	1.01	0.76	1.34	0.96	>0.99
Tcell.EXH.Exhausted.CD8.T.cell.vs.Naive.CD8.T.cell.Metagene.3_Science.2016.PMID.27789795	1.24	0.90	1.71	0.19	0.49
Tcell.EXH.Memory.CD8.T.cell.a.vs.Naive.CD8.T.cell.Metagene.1_Science.2016.PMID.27789795	1.44	1.08	1.96	0.02	0.29
Tcell.EXH.Memory.CD8.T.cell.a.vs.Naive.CD8.T.cell.Metagene.2_Science.2016.PMID.27789795	1.16	0.84	1.62	0.36	0.66
Tcell.EXH.Memory.CD8.T.cell.a.vs.Naive.CD8.T.cell.Metagene.3.Science_2016.PMID.27789795	1.12	0.83	1.52	0.45	0.73
Tcell.NK.51gene_Genome.Biol.2013.PMID.23618380	1.32	1.00	1.77	0.05	0.37
Tcell.NK.Metagene_Genome.Biol.2013.PMID.23618380	1.31	0.98	1.75	0.07	0.37
Tcell.RM_Nat_Med.2018.PMID.29942092	1.23	0.92	1.66	0.17	0.46
Tcell.survival.2gene_Nature.2020.PMID.31942077	1.29	0.98	1.72	0.08	0.37
Tcells_CancerImmunolRes.2018.PMID.30266715	1.36	1.02	1.83	0.04	0.37
Tcells_Immunity.2013.PMID.24138885	1.37	1.03	1.83	0.03	0.37
Tcells_TFH_Nat.Methods.2015.PMID.25822800	1.37	1.03	1.84	0.04	0.37
Tcells.CD4.memory.activated_Nat.Methods.2015.PMID.25822800	1.37	1.02	1.86	0.04	0.37
Tcells.CD4.memory.resting_Nat.Methods.2015.PMID.25822800	1.29	0.97	1.73	0.08	0.37
Tcells.CD4.naive_Nat.Methods.2015.PMID.25822800	1.28	0.96	1.71	0.10	0.38
Tcells.CD8_Immunity.2013.PMID.24138885	1.03	0.80	1.33	0.80	0.94
Tcells.CD8_Nat.Methods.2015.PMID.25822800	1.33	1.00	1.79	0.05	0.37
Tcells.CD8.MCP_Nature.2020.PMID.31942075	1.18	0.90	1.55	0.23	0.53
Tcells.Cytotoxic.MCP_Nature.2020.PMID.31942075	1.27	0.96	1.71	0.10	0.38
Tcells.gammadelta_Nat.Methods.2015.PMID.25822800	1.33	1.00	1.78	0.05	0.37
Tcells.helper_Immunity.2013.PMID.24138885	1.09	0.83	1.43	0.53	0.79
Tcells.MCP_Nature.2020.PMID.31942077	1.33	0.99	1.79	0.06	0.37
Tcells.regulatory.2gene_Nature.2020.PMID.31942077	1.07	0.83	1.39	0.60	0.80
Tcells.Tregs_Nat.Methods.2015.PMID.25822800	1.23	0.93	1.64	0.15	0.45
TCGA.BRCA.1198_BASAL_JCI.2020.PMID.32573490	0.88	0.63	1.22	0.45	0.74
TCGA.BRCA.1198_Chromogranin_JCI.2020.PMID.32573490	1.01	0.76	1.34	0.94	0.99
TCGA.BRCA.1198_COLLAGEN11A_JCI.2020.PMID.32573490	1.03	0.77	1.39	0.83	0.94
TCGA.BRCA.1198_EN1_FDZ9_JCI.2020.PMID.32573490	1.28	0.92	1.79	0.14	0.45
TCGA.BRCA.1198_FGFR4_EGF_JCI.2020.PMID.32573490	1.23	0.92	1.66	0.17	0.46
TCGA.BRCA.1198_HISTONES_JCI.2020.PMID.32573490	0.96	0.72	1.27	0.76	0.92
TCGA.BRCA.1198_HOXC11_HOTAIR_SIX1_JCI.2020.PMID.32573490	1.21	0.88	1.69	0.25	0.55
TCGA.BRCA.1198_IL8_CCL_JCI.2020.PMID.32573490	1.38	1.03	1.88	0.03	0.37
TCGA.BRCA.1198_immune_CD19_JCI.2020.PMID.32573490	1.29	0.95	1.77	0.11	0.39
TCGA.BRCA.1198_immune_CD34_TIE1_JCI.2020.PMID.32573490	1.04	0.75	1.43	0.83	0.94
TCGA.BRCA.1198_immune_CD4_CD53_CD84_BTK_JCI.2020.PMID.32573490	1.39	1.03	1.89	0.03	0.37
TCGA.BRCA.1198_immune_CD8_GZMK_JCI.2020.PMID.32573490	1.29	0.97	1.73	0.08	0.37
TCGA.BRCA.1198_immune_CTLA4_CXCL_FOXP3_JCI.2020.PMID.32573490	1.31	0.97	1.79	0.08	0.37
TCGA.BRCA.1198_immune_FOS_JUN_IL6_JCI.2020.PMID.32573490	1.09	0.85	1.39	0.47	0.75

TCGA.BRCA.1198_immune_GIMAP_IL16_JCI.2020.PMID.32573490	1.20	0.91	1.59	0.20	0.51
TCGA.BRCA.1198_immune_HLA_A_F_JCI.2020.PMID.32573490	1.15	0.88	1.50	0.31	0.59
TCGA.BRCA.1198_immune_HLA_D_JCI.2020.PMID.32573490	1.18	0.90	1.57	0.23	0.53
TCGA.BRCA.1198_immune_INTERFERON_JCI.2020.PMID.32573490	0.86	0.65	1.13	0.28	0.56
TCGA.BRCA.1198_IMMUNE1_JCI.2020.PMID.32573490	1.23	0.90	1.68	0.20	0.51
TCGA.BRCA.1198_LUMINAL_JCI.2020.PMID.32573490	0.49	0.31	0.75	0.001	0.06
TCGA.BRCA.1198_MYBL2_APOBEC3B_JCI.2020.PMID.32573490	1.70	1.20	2.45	0.003	0.14
TCGA.BRCA.1198_NORMAL_JCI.2020.PMID.32573490	1.07	0.76	1.52	0.69	0.88
TCGA.BRCA.1198_NORMAL2_JCI.2020.PMID.32573490	1.09	0.82	1.45	0.54	0.79
TCGA.BRCA.1198_PDCHA_MANY_JCI.2020.PMID.32573490	1.15	0.88	1.50	0.31	0.59
TCGA.BRCA.1198_S100A7_8_9_JCI.2020.PMID.32573490	1.02	0.74	1.41	0.89	0.97
TCGA.BRCA.1198_TP63_JCI.2020.PMID.32573490	1.06	0.78	1.43	0.70	0.89
TCGA.BRCA.1198.IMMUNOGLOBULIN_JCI.2020.PMID.32573490	1.25	0.94	1.69	0.13	0.43
TCGA.CSF1.response_Immunity.2018.PMID.29628290	1.33	0.98	1.81	0.07	0.37
TCGA.IFN.score_Immunity.2018.PMID.29628290	0.84	0.63	1.10	0.21	0.52
TCGA.Liexpression.score_Immunity.2018.PMID.29628290	1.28	0.96	1.72	0.09	0.38
TCGA.Serum.response.up_Immunity.2018.PMID.29628290	1.28	0.98	1.67	0.07	0.37
TCGA.TFH_Immunity.2018.PMID.29628290	1.32	1.02	1.74	0.04	0.37
TCGA.Tgd_Immunity.2018.PMID.29628290	0.77	0.56	1.05	0.11	0.38
TCGA.TGFB.score_Immunity.2018.PMID.29628290	1.24	0.93	1.65	0.14	0.44
Tcm_Immunity.2013.PMID.24138885	0.97	0.73	1.28	0.81	0.94
Tem_Immunity.2013.PMID.24138885	0.96	0.70	1.30	0.78	0.93
TFH_Immunity.2013.PMID.24138885	1.32	1.02	1.74	0.04	0.37
Tgd_Immunity.2013.PMID.24138885	0.77	0.56	1.05	0.11	0.38
Th1_cells_Immunity.2013.PMID.24138885	1.31	0.97	1.78	0.08	0.37
Th17_cells_Immunity.2013.PMID.24138885	0.65	0.48	0.87	0.004	0.16
Th2_cells_Immunity.2013.PMID.24138885	1.08	0.81	1.44	0.61	0.81
TLS.9Gene.Signature_Nature.2020.PMID.31942071	1.16	0.89	1.52	0.28	0.56
TLS.CXCL13.SingleGene_Nature.2020.PMID.31942077	1.35	1.00	1.85	0.06	0.37
TLS.Hallmark.Gene.Signature_Nature.2020.PMID.31942071	1.40	1.07	1.86	0.02	0.29
TLS.Known.Markers_Nature.2020.PMID.31942071	1.43	1.08	1.91	0.01	0.29
TLS.Structure.12chemokine_FrontImmunol.2017.PMID.28713385	1.38	1.04	1.86	0.03	0.37
TLS.tumors.wTLS.and.CD8.vs.CD8alone_Nature.2020.PMID.31942071	1.30	0.98	1.74	0.07	0.37
TNBC.good.prognosis.TNBC.230genes_BCR.2011.PMID.21978456	1.06	0.80	1.40	0.68	0.88
TNBC.good.prognosis.TNBC.26genes_BCR.2011.PMID.21978456	1.08	0.82	1.43	0.57	0.79
TNBC.metastasis.free.survival_PLoS.One.2013.PMID.24349199	1.51	1.11	2.09	0.01	0.29
TNBC.poor.prognosis.TNBC.26genes_BCR.2011.PMID.21978456	1.05	0.80	1.37	0.74	0.91
Translation.Pathway_CancerImmunolRes.2018.PMID.30266715	1.07	0.85	1.35	0.55	0.79
Tumour.hypoxia.causes.DNA.hypermethylation_Nature.2016.PMID.27533040	1.02	0.78	1.33	0.88	0.97
Type.1.T.helper.cell_CellRep.2017.PMID.28052254	1.32	0.99	1.79	0.07	0.37
Type.17.T.helper.cell_CellRep.2017.PMID.28052254	1.00	0.75	1.33	0.98	>0.99
Type.2.T.helper.cell_CellRep.2017.PMID.28052254	1.15	0.90	1.49	0.26	0.55
Up.Basal.High_Nat.Cell.Biol.2014.PMID.25173976	0.83	0.61	1.12	0.22	0.52
Up.Proliferation_Nat.Cell.Biol.2014.PMID.25173976	1.25	0.92	1.70	0.16	0.46
Upregulated.by.oncogenic.NRAS.basal_Cell.Rep.2016.PMID.26166574	0.83	0.61	1.11	0.21	0.52
Upregulated.upon.NRAS.repression.basal_Cell.Rep.2017.PMID.26166574	0.72	0.54	0.97	0.03	0.37
Vascular.Content_Clin.Exp.Metastasis.2014.PMID.23975155	1.25	0.91	1.75	0.17	0.46

VEGF.13genes_BMC.Med.2009.PMID.19291283	1.20	0.90	1.60	0.20	0.51
Wirapati.Proliferation_BCR.2008.PMID.18662380	1.49	1.10	2.06	0.01	0.29
Wound.Signature_CCR.2009.PMID.19887484	1.01	0.74	1.37	0.96	>0.99
X11q13.Amplicon_BMC.Med.Genomics.2011.PMID.21214954	0.84	0.64	1.08	0.20	0.50
X12qMDM4.BMC.Med.Genomics.2011.PMID.21214954	0.92	0.69	1.20	0.56	0.79
X13q14.Amplicon_BMC.Med.Genomics.2011.PMID.21214954	0.87	0.69	1.10	0.26	0.55
X15q25.Amplicon_BMC.Med.Genomics.2011.PMID.21214954	1.04	0.81	1.34	0.73	0.91
X16.13.Amplicon_BMC.Med.Genomics.2011.PMID.21214954	1.05	0.81	1.36	0.71	0.89
X16q23.Amplicon_BMC.Med.Genomics.2011.PMID.21214954	1.18	0.93	1.52	0.18	0.46
X17PP13.Amplicon_BMC.Med.Genomics.2011.PMID.21214954	1.08	0.83	1.39	0.58	0.79
X17q25x.BMC.Med.Genomics.2011.PMID.21214954	1.12	0.88	1.43	0.35	0.65
X19p13.Amplicon_BMC.Med.Genomics.2011.PMID.21214954	1.00	0.77	1.29	>0.99	>0.99
X1p36.Amplicon_BMC.Med.Genomics.2011.PMID.21214954	1.06	0.82	1.38	0.63	0.83
X3p21.Amplicon_BMC.Med.Genomics.2011.PMID.21214954	0.89	0.69	1.15	0.37	0.66
X4p16.Amplicon_BMC.Med.Genomics.2011.PMID.21214954	0.89	0.69	1.15	0.38	0.67
X5Q_BCRT.2012.PMID.22048815	0.88	0.68	1.14	0.33	0.61
X8p.Amplicon_BMC.Med.Genomics.2011.PMID.21214954	1.19	0.89	1.58	0.23	0.53
X8p22.Amplicon_BMC.Med.Genomics.2011.PMID.21214954	0.84	0.64	1.09	0.19	0.49
XBP1.Signature_Nature.2014.PMID.24670641	0.96	0.72	1.28	0.79	0.94

NSABP B-41					
Signature	OR	95% CI		*P*	adjusted *P*
Activate.Endothelium_Clin.Exp.Metastasis.2014.PMID.23975155	1.23	0.92	1.66	0.16	0.31
Activated.B.cell_CellRep.2017.PMID.28052254	1.45	1.10	1.94	0.01	0.04
Activated.Blood.Neutrophil.Signature_Nat.Cell.Biol.2019.PMID.31263265	1.23	0.92	1.66	0.17	0.32
Activated.Cancer.Cell.Signature_Nat.Cell.Biol.2019.PMID.31263265	0.90	0.69	1.19	0.47	0.66
Activated.CD4.T.cell_CellRep.2017.PMID.28052254	1.98	1.47	2.71	<0.001	0.001
Activated.CD8.T.cell_CellRep.2017.PMID.28052254	1.83	1.37	2.48	<0.001	0.002
Activated.dendritic.cell_CellRep.2017.PMID.28052254	1.79	1.34	2.44	<0.001	0.002
Activated.Lung.MSC.Signature_Nat.Cell.Biol.2019.PMID.31263265	0.89	0.68	1.17	0.41	0.61
Activated.Lung.Neutrophil.Signature_Nat.Cell.Biol.2019.PMID.31263265	1.27	0.96	1.69	0.09	0.21
aDC_Immunity.2013_PMID.24138885.PMID.24138885	1.62	1.22	2.16	0.001	0.006
ADM.S100A10.A110NDGR1.Cluster_BMC.Med.Genomics.2011.PMID.21214954	0.93	0.68	1.27	0.63	0.75
African.and.European.Ancestry.TCGA.Negative_JAMA.Oncol.2017.PMID.28472234	0.98	0.70	1.38	0.93	0.97
African.and.European.Ancestry.TCGA.Positive_JAMA.Oncol.2017.PMID.28472234	0.83	0.61	1.12	0.22	0.40
Age.associated.signature_Genome.Biol.2015.PMID.26343147	0.72	0.54	0.96	0.03	0.08
aMaSC_BCR.2010.PMID.20346151	0.91	0.67	1.23	0.53	0.69
aMaSC.HsEnriched_BCR.2015.PMID.25575446	0.89	0.66	1.19	0.44	0.62
aMaSC.HsEnriched.Refined1_BCR.2015.PMID.25575446	0.80	0.58	1.08	0.15	0.29
aMaSC.Lim09_BCR.2015.PMID.25575446	0.82	0.60	1.10	0.18	0.34
aMaSC.Prat_BCR.2015.PMID.25575446	0.90	0.66	1.22	0.50	0.67
aMaSC.Shehata_BCR.2015.PMID.25575446	1.62	1.13	2.36	0.01	0.04
aMaSC.Signature_Cell.Stem.Cell.2012.PMID.22305568	1.06	0.81	1.40	0.66	0.78
AMPH.EPIREGULIN.Cluster_BMC.Med.Genomics.2011.PMID.21214954	0.80	0.56	1.14	0.22	0.40
Amplification.50_Genome.Biol.2014.PMID.25164602	0.90	0.67	1.22	0.50	0.67
Amplification.50.better.than._Genome.Biol.2015.PMID.25164602	0.84	0.62	1.14	0.26	0.44
Apocrine.Features_J.Pathol.2017.PMID.27861902	1.49	1.11	2.01	0.008	0.03
aStr.HsEnriched_BCR.2015.PMID.25575446	1.18	0.87	1.61	0.30	0.49
aStr.HsEnriched.Refined1_BCR.2015.PMID.25575446	0.91	0.66	1.24	0.54	0.69
aStr.HsEnriched.Refined2_BCR.2015.PMID.25575446	0.98	0.71	1.35	0.92	0.97
aStr.Lim09_BCR.2015.PMID.25575446	1.07	0.79	1.46	0.66	0.78
aStr.Prat_BCR.2015.PMID.25575446	1.16	0.85	1.59	0.34	0.53
aStr.Shehata_BCR.2015.PMID.25575446	1.22	0.89	1.68	0.22	0.40
BASAL.Cluster_BMC.Med.Genomics.2011.PMID.21214954	0.93	0.69	1.27	0.67	0.78
Bcell.cluster_CCR.2014.PMID.24916698	1.33	1.02	1.75	0.04	0.11
Bcell.IL10.MINUS_Immunol.2014.PMID.25080484	1.12	0.86	1.45	0.39	0.59
Bcell.IL10.PLUS_Immunol.2014.PMID.25080484	1.65	1.24	2.23	0.001	0.006

Bcell.lineage.MCP_Nature.2020.PMID.31942077	1.36	1.04	1.80	0.03	0.08
Bcell.Plasma.52gene_Genome.Biol.2013.PMID.23618380	1.31	1.02	1.70	0.04	0.11
Bcell.Plasma.Metagene_Genome.Biol.2013.PMID.23618380	1.31	1.02	1.70	0.03	0.10
Bcell.Tcell.Cooperation_Cell.2019.PMID.31730857	1.28	0.98	1.67	0.07	0.17
Bcells_CancerImmunolRes.2018.PMID.30266715	1.47	1.12	1.95	0.006	0.03
Bcells_Immunity.2013.PMID.24138885	1.38	1.03	1.88	0.03	0.09
Bcells.Centroblast_JCO.2015.PMID.25800755	1.30	0.95	1.78	0.11	0.24
Bcells.Centrocyte_JCO.2015.PMID.25800755	1.29	0.99	1.70	0.06	0.15
Bcells.Memory_JCO.2015.PMID.25800755	1.10	0.82	1.47	0.53	0.69
Bcells.memory_Nat.Methods.2015.PMID.25822800	1.48	1.12	1.98	0.007	0.03
Bcells.Naive_JCO.2015.PMID.25800755	1.32	0.98	1.79	0.07	0.16
Bcells.naive_Nat.Methods.2015.PMID.25822800	1.48	1.11	2.00	0.008	0.03
Bcells.Plasmablast_JCO.2015.PMID.25800755	1.79	1.33	2.47	<0.001	0.002
Blood.vessels_Immunity.2013.PMID.24138885	1.11	0.80	1.56	0.53	0.69
bMYB.Signature_Oncogene.2009.PMID.19043454	1.28	0.94	1.75	0.12	0.25
C3TAG.Responding_CCR.2013.PMID.23780888	1.08	0.75	1.58	0.67	0.79
C3TAG.Untreated_CCR.2013.PMID.23780888	1.19	0.87	1.66	0.28	0.46
CD103.Negative_Cancer.Cell.2014.PMID.25446897	1.24	0.93	1.69	0.15	0.30
CD103.Positive_Cancer.Cell.2014.PMID.25446897	1.50	1.12	2.05	0.008	0.03
CD103.Ratio_Cancer.Cell.2014.PMID.25446897	1.83	1.32	2.59	<0.001	0.004
CD274_Single_Gene.Single	1.56	1.17	2.11	0.003	0.01
CD34.CD36.Cluster_BMC.Med.Genomics.PMID.21214954	0.97	0.65	1.43	0.86	0.93
CD44.downregulated.genes_Cancer.Cell.2007.PMID.17349583	1.47	1.11	1.99	0.009	0.03
CD44.upregulated.genes_Cancer.Cell.2007.PMID.17349583	0.92	0.71	1.19	0.53	0.69
CD56bright.natural.killer.cell_CellRep.2017.PMID.28052254	1.39	1.05	1.85	0.02	0.07
CD56dim.natural.killer.cell_CellRep.2017.PMID.28052254	1.61	1.19	2.22	0.003	0.01
CD68.cluster_CCR.2014.PMID.24916698	1.45	1.06	2.00	0.02	0.07
CD8.cluster_CCR.2014.PMID.24916698	1.80	1.35	2.45	<0.001	0.002
CDKN2A_Single_Gene.Single	0.63	0.46	0.85	0.004	0.02
Central.memory.CD4.T.cell_CellRep.2017.PMID.28052254	1.34	0.98	1.84	0.07	0.17
Central.memory.CD8.T.cell_CellRep.2017.PMID.28052254	1.17	0.89	1.55	0.27	0.45
CES.Score_CCR.2017.PMID.27903675	0.64	0.45	0.90	0.01	0.04
Chromogramin_BMC.Med.Genomics.2011.PMID.21214954	0.79	0.60	1.04	0.10	0.22
CIN70_Nat.Genet.2006.PMID.16921376	1.25	0.92	1.71	0.16	0.32
Claudin.High_Genome.Biol.2007.PMID.17493263	1.75	1.30	2.41	<0.001	0.003
Claudin.Low_Genome.Biol.2007.PMID.17493263	0.67	0.47	0.94	0.02	0.07
Claudin.Low.29_Cancer.Res.2009.PMID.19435916	0.87	0.60	1.24	0.45	0.64
cMYB.Signature_PLoS.One.2010.PMID.20949095	1.00	0.75	1.33	0.98	0.99
CORE.Bcell.signature.Garber_Cell.Mol.Gastroenterol.Hepatol.2017.PMID.28508029	1.39	1.04	1.90	0.03	0.09
CTLA4_Single_Gene.Single	1.93	1.44	2.62	<0.001	0.001
Cytolytic.activity_Cell.2015.PMID.25594174	1.81	1.35	2.47	<0.001	0.002
Cytotoxic.cells_Immunity.2013.PMID.24138885	1.77	1.32	2.41	<0.001	0.002
Day7.Downregulated_Nat.Cell.Biol.2014.PMID.25173976	0.99	0.74	1.34	0.97	0.98
Day7.Upregulated_Nat.Cell.Biol.2014.PMID.25173976	1.06	0.77	1.45	0.73	0.83
DC_Immunity.2013.PMID.24138885	1.46	1.06	2.02	0.02	0.07
DCIS.HGF.down_BCR.2013.PMID.24025166	1.09	0.81	1.48	0.56	0.71
DCIS.HGF.up_BCR.2014.PMID.24025166	0.95	0.70	1.27	0.72	0.82

Delection.50_Genome.Biol.2016.PMID.25164602	0.75	0.56	0.99	0.04	0.12
Delection.50.better.than_Genome.Biol.2017.PMID.25164602	0.95	0.73	1.23	0.70	0.81
Dendritic.cells.activated_Nat.Methods.2015.PMID.25822800	1.66	1.23	2.29	0.001	0.007
Dendritic.cells.resting_Nat.Methods.2015.PMID.25822800	1.68	1.22	2.36	0.002	0.01
Down.Basal.High_Nat.Cell.Biol.2014.PMID.25173976	0.91	0.67	1.23	0.54	0.69
Down.CLOW.High_Nat.Cell.Biol.2014.PMID.25173976	0.77	0.55	1.07	0.12	0.25
Downregulated.upon.NRAS.repression.basal_Cell.Rep.2015.PMID.26166574	1.60	1.20	2.17	0.002	0.009
Ductal.Carcinoma.In.Situ_J.Pathol.2017.PMID.27861902	0.91	0.70	1.17	0.47	0.65
Duke.Module01.acidosis_PNASUSA.2010.PMID.20335537	0.87	0.63	1.20	0.40	0.59
Duke.Module02.akt_PNASUSA.2010.PMID.20335537	1.47	1.08	2.02	0.01	0.05
Duke.Module03.betacatenin_PNASUSA.2010.PMID.20335537	1.12	0.85	1.49	0.41	0.61
Duke.Module04.E2F1_PNASUSA.2010.PMID.20335537	1.66	1.25	2.25	0.001	0.004
Duke.Module05.EGFR_PNASUSA.2010.PMID.20335537	1.22	0.88	1.71	0.23	0.41
Duke.Module06.ER_PNASUSA.2010.PMID.20335537	0.91	0.67	1.23	0.54	0.69
Duke.Module07.glucosedepletion_PNASUSA.2010.PMID.20335537	1.55	1.18	2.07	0.002	0.01
Duke.Module08.HER2_PNASUSA.2010.PMID.20335537	1.71	1.23	2.41	0.002	0.01
Duke.Module09.hypoxia_PNASUSA.2010.PMID.20335537	0.95	0.69	1.29	0.72	0.82
Duke.Module10.IFNA_PNASUSA.2010.PMID.20335537	1.10	0.82	1.47	0.52	0.69
Duke.Module11.IFNG_PNASUSA.2010.PMID.20335537	1.29	0.97	1.72	0.09	0.20
Duke.Module12.lacticacidosis_PNASUSA.2010.PMID.20335537	1.08	0.81	1.46	0.59	0.73
Duke.Module13.myc_PNASUSA.2010.PMID.20335537	0.89	0.66	1.19	0.43	0.62
Duke.Module14.p53_PNASUSA.2010.PMID.20335537	0.68	0.49	0.94	0.02	0.07
Duke.Module15.p63_PNASUSA.2010.PMID.20335537	0.95	0.72	1.25	0.70	0.81
Duke.Module16.pi3k_PNASUSA.2010.PMID.20335537	1.46	1.08	1.98	0.01	0.05
Duke.Module17.PR_PNASUSA.2010.PMID.20335537	0.56	0.39	0.78	0.001	0.005
Duke.Module18.ras_PNASUSA.2010.PMID.20335537	1.12	0.85	1.48	0.42	0.62
Duke.Module19.src_PNASUSA.2010.PMID.20335537	1.08	0.82	1.44	0.60	0.74
Duke.Module20.STAT3_PNASUSA.2010.PMID.20335537	1.26	0.92	1.73	0.15	0.30
Duke.Module21.TGFB_PNASUSA.2010.PMID.20335537	0.93	0.71	1.22	0.60	0.74
Duke.Module22.TNFA_PNASUSA.2010.PMID.20335537	1.46	1.10	1.96	0.009	0.04
Durvalumab.signature_CCR.2018.PMID.29716923	1.68	1.22	2.34	0.002	0.009
Early.IRS.1_PLoS.One.2016.PMID.26991655	1.40	1.05	1.89	0.03	0.08
Early.IRS.2_PLoS.One.2016.PMID.26991655	1.15	0.87	1.51	0.33	0.53
Early.Relapse.ERPos.33genes_JAMA.2011.PMID.21558518	1.03	0.78	1.37	0.81	0.89
Early.Response.ERNeg.27genes_JAMA.2011.PMID.21558518	0.93	0.70	1.23	0.62	0.75
Effector.memeory.CD4.T.cell_CellRep.2017.PMID.28052254	1.28	0.97	1.72	0.09	0.20
Effector.memeory.CD8.T.cell_CellRep.2017.PMID.28052254	1.70	1.26	2.32	0.001	0.004
EGFR_Single_Gene.Single	0.92	0.67	1.27	0.63	0.76
EMT.down.Taube_PNAS.2010.PMID.20713713	1.41	0.99	2.03	0.06	0.15
EMT.down.Weingberg_PNAS.2010.PMID.20713713	0.99	0.72	1.35	0.93	0.97
EMT.up.Taube_PNAS.2010.PMID.20713713	1.04	0.77	1.41	0.80	0.88
EMT.up.Weinberg_PNAS.2010.PMID.20713713	0.94	0.71	1.23	0.63	0.76
Endothelial.cells.MCP_Nature.2020..PMID.31942077	1.14	0.83	1.56	0.42	0.62
Endothelial.Normal_Angiogenesis.2014.PMID.24257808	1.28	0.96	1.72	0.10	0.22
Endothelial.Tumor_Angiogenesis.2014.PMID.24257808	1.12	0.85	1.48	0.43	0.62
Eosinophil_CellRep.2017.PMID.28052254	1.56	1.16	2.14	0.004	0.02
Eosinophils_Immunity.2013.PMID.24138885	1.49	1.11	2.02	0.009	0.03

Eosinophils_Nat.Methods.2015.PMID.25822800	1.89	1.39	2.61	<0.001	0.002
Epithelial.Tubule.Formation_J.Pathol.2017.PMID.27861902	0.83	0.60	1.13	0.24	0.42
ERBB2_Single_Gene.Single	1.50	1.13	2.01	0.006	0.03
ERBB3_Single_Gene.Single	1.03	0.74	1.42	0.86	0.92
ESR1_Single_Gene.Single	0.60	0.40	0.90	0.01	0.05
ESTIMATE.Immune_Nat.Commun.2013.PMID.24113773	1.90	1.41	2.62	<0.001	0.001
ESTIMATE.Stromal_Nat.Commun.2013.PMID.24113773	1.26	0.94	1.69	0.13	0.27
Euclidean.Distance.CLOW_BCR.2010.PMID.20813035	1.06	0.79	1.42	0.70	0.81
EXTENDED.Bcell.signature.Garber_Cell.Mol.Gastroenterol.Hepatol.2017.PMID.28508029	1.46	1.11	1.93	0.008	0.03
FGFR4_Single_Gene.Single	1.29	0.96	1.73	0.09	0.21
FGFR4.Induced_JCI.2020.PMID.32573490	1.43	1.07	1.93	0.02	0.06
FGFR4.Repressed_JCI.2020.PMID.32573490	1.03	0.76	1.39	0.85	0.92
Fibrinogen.Cluster_BMC.Med.Genomics.2011.PMID.21214954	0.74	0.55	0.99	0.05	0.13
Fibroblast.Cluster_BMC.Med.Genomics.2011.PMID.21214954	0.85	0.64	1.11	0.23	0.41
Fibroblasts.MCP_Nature.2020.PMID.31942077	0.94	0.70	1.24	0.64	0.77
Fibromatosis_Lab.Invest.2008.PMID.18414401	0.89	0.68	1.17	0.41	0.61
fMaSC.Metab_CellRep.2018.PMID.30089273	0.76	0.53	1.07	0.12	0.25
fMaSC.Metab8_CellRep.2018.PMID.30089273	0.93	0.68	1.27	0.64	0.77
fMaSC.refined1_BCR.2015.PMID.25575446	1.00	0.76	1.34	0.98	0.99
fMasC.Signature_Cell.Stem.Cell.2012.PMID.22305568	1.16	0.85	1.58	0.36	0.55
fMaSC.Signature_CellRep.2018.PMID.30089273	1.14	0.86	1.53	0.37	0.56
FOS.JUN_Cluster_BMC.Med.Genomics.2011.PMID.21214954	1.06	0.79	1.45	0.70	0.81
FOXC1.Hair.Follicles.P30C.LO.vs.WT.Negative_Science.2016.PMID.26912704	1.01	0.76	1.34	0.93	0.97
FOXC1.Hair.Follicles.P30C.LO.vs.WT.Positive_Science.2016.PMID.26912704	1.13	0.86	1.48	0.37	0.56
fSTR.Signature_Cell.Stem.Cell.2012.PMID.22305568	0.87	0.61	1.25	0.47	0.65
Gamma.delta.T.cell_CellRep.2017.PMID.28052254	1.11	0.83	1.48	0.50	0.67
GATA3.induced.genes_JCO.2006.PMID.16505416	1.36	0.98	1.90	0.07	0.16
GATA3.induced.genes_Oncogene.2004.PMID.15361840	1.16	0.84	1.61	0.38	0.57
GDF11.TGFBR3_Nat.Cell.Biol.2014.PMID.24658685	0.99	0.75	1.29	0.92	0.97
Glycolysis_BMC.Med.2009.PMID.19291283	0.97	0.73	1.28	0.83	0.90
GO.DOWN.with.SOX10.OE_Cell.Rep.2015.PMID.26365194	1.33	1.01	1.78	0.05	0.13
GO.UP.with.SOX10.OE_Cell.Rep.2015.PMID.26365194	1.36	0.99	1.89	0.06	0.15
GSEA_BIOCARTA_ALK_PATHWAY.PMID.16199517	0.92	0.67	1.26	0.61	0.74
GSEA_BIOCARTA.AKT.PATHWAY.PMID.16199517	1.21	0.92	1.60	0.17	0.32
GSEA_BIOCARTA.BRCA.ATR.PATHWAY.ATRBRCA.PMID.16199517	1.02	0.75	1.38	0.91	0.96
GSEA_BIOCARTA.CASPASE.PATHWAY.PMID.16199517	1.77	1.31	2.42	<0.001	0.002
GSEA_BIOCARTA.CTLA4.PATHWAY.PMID.16199517	1.89	1.41	2.56	<0.001	0.001
GSEA_BIOCARTA.IGF1R.PATHWAY.PMID.16199517	0.79	0.57	1.07	0.14	0.28
GSEA_BIOCARTA.MTOR.PATHWAY.PMID.16199517	1.20	0.90	1.62	0.22	0.40
GSEA_BIOCARTA.PTEN.PATHWAY.PMID.16199517	0.87	0.65	1.16	0.34	0.53
GSEA_BIOCARTA.RAS.PATHWAY.PMID.16199517	0.94	0.71	1.25	0.68	0.80
GSEA_BIOCARTA.RB.PATHWAY.PMID.16199517	1.58	1.17	2.17	0.003	0.02
GSEA_BIOCARTA.VEGF.PATHWAY.PMID.16199517	1.14	0.88	1.48	0.34	0.53
GSEA_HALLMARK.MYC.TARGETS.V1.PMID.16199517	0.92	0.71	1.19	0.53	0.69
GSEA_HELLER.HDAC.TARGETS.DOWN.PMID.16199517	1.49	1.12	2.00	0.007	0.03
GSEA_NELSON.RESPONSE.TO.ANDROGEN.UP.PMID.16199517	1.72	1.28	2.34	<0.001	0.003
GSEA_REACTOME.PD1.SIGNALING.PMID.16199517	1.76	1.33	2.36	<0.001	0.002

GSEA_REACTOME.PI3K.CASCADE.PMID.16199517	0.99	0.72	1.35	0.94	0.97
GSEA_RETINOL.METABOLISM.KEGG.PMID.16199517	1.24	0.86	1.80	0.26	0.44
GSEA.GP1_Proliferation.DNA.repair..PUJANA.CHEK2.PCC.NETWORK.PMID.25109877	1.15	0.87	1.54	0.33	0.53
GSEA.GP1_Proliferation.DNA.repair.REACTOME.CELL.CYCLE.MITOTIC.PMID.25109877	1.15	0.87	1.53	0.32	0.52
GSEA.GP10_Fatty.acid.oxidation.CARBOXYLIC.ACID.METABOLIC.PROCESS.PMID.25109877	1.12	0.80	1.57	0.52	0.69
GSEA.GP11_Immune.IFN.PerouLab.PMID.25109877	1.19	0.89	1.60	0.25	0.42
GSEA.GP12_Hypoxia.glycolosis.SEMENZA.HIF1.TARGETS.PMID.25109877	0.86	0.64	1.17	0.34	0.53
GSEA.GP13_Neural.signailng.MODULE100.PMID.25109877	1.11	0.82	1.52	0.50	0.67
GSEA.GP13_Neural.signailng.NERVOUS.SYSTEM.DEVELOPMENT.PMID.25109877	0.98	0.74	1.30	0.89	0.95
GSEA.GP14_Plasma.membrane.cell.cell.signaling.MORF.CNTN1.PMID.25109877	0.86	0.63	1.17	0.32	0.52
GSEA.GP15_EGF.signaling.NAGASHIMA.EGF.SIGNALING.UP.PMID.25109877	0.97	0.73	1.27	0.81	0.88
GSEA.GP16_Protein.kinase.signaling.MAPKs.INTRACELLULAR.SIGNALING.CASCADE.PMID.25109877	1.81	1.32	2.53	<0.001	0.003
GSEA.GP16_Protein.kinase.signaling.MAPKs.REGULATION.OF.KINASE.ACTIVITY.PMID.25109877	1.57	1.15	2.17	0.005	0.02
GSEA.GP17_Basal.signaling.SMID.BREAST.CANCER.BASAL.UP.PMID.25109877	1.11	0.84	1.49	0.45	0.64
GSEA.GP18_Vesicle.EPR.MEMBRANE.COAT.PMID.25109877	1.40	1.08	1.84	0.01	0.05
GSEA.GP19_1Q.amplicon.PerouLab.PMID.25109877	0.83	0.61	1.11	0.21	0.38
GSEA.GP2_Immune.Tcell.Bcell.KEGG.HEMATOPOIETIC.CELL.LINEAGE.PMID.25109877	1.61	1.21	2.16	0.001	0.008
GSEA.GP2_Immune.Tcell.Bcell.PerouLab.PMID.25109877	1.78	1.32	2.42	<0.001	0.002
GSEA.GP20_TAL1.Leukemia.erythropoiesis.GNF2.TAL1.PMID.25109877	0.81	0.61	1.08	0.15	0.30
GSEA.GP21_Anti.apoptosis.DNA.stability.MORF.BCL2.PMID.25109877	0.84	0.62	1.12	0.23	0.41
GSEA.GP21_Anti.apoptosis.DNA.stability.MORF.MT4.PMID.25109877	0.80	0.59	1.10	0.17	0.32
GSEA.GP21_Anti.apoptosis.DNA.stability.MORF.STK17A.PMID.25109877	0.88	0.66	1.18	0.40	0.59
GSEA.GP22_16Q22.24.amplicon.PerouLab.PMID.25109877	0.78	0.58	1.05	0.11	0.24
GSEA.GP3_Tumo.suppressing.miRNA.targets.GTTTGTT.MIR.495.PMID.25109877	1.29	0.95	1.77	0.10	0.23
GSEA.GP3_Tumor.suppressing.miRNA.targets.DACOSTA.UV.RESPONSE.VIA.ERCC3.DN.PMID.25109877	1.37	0.93	2.05	0.11	0.25
GSEA.GP3_Tumor.suppressing.miRNA.targets.TGCTTTG.MIR.330.PMID.25109877	1.28	0.92	1.78	0.14	0.28
GSEA.GP4_MES.ECM.PerouLab.PMID.25109877	0.87	0.65	1.14	0.30	0.49
GSEA.GP5_MYC.targets.TERT.PerouLab.PMID.25109877	0.90	0.65	1.25	0.54	0.69
GSEA.GP6_Squamous.differentiation.development.RICKMAN.TUMOR.DIFFERENTIATED.WELL.VS.POORLY.DN.PMID.25109877	1.25	0.93	1.69	0.14	0.29
GSEA.GP7_Estrogen.signaling.SMID.BREAST.CANCER.BASAL.DN.PMID.25109877	0.88	0.65	1.19	0.43	0.62
GSEA.GP8_FOXO.stemness.MORF.PTPRB.PMID.25109877	1.00	0.75	1.34	0.99	0.99
GSEA.GP8_FOXO.stemness.TTGTTT.VSFOXO4.01.PMID.25109877	1.47	1.07	2.06	0.02	0.07
GSEA.GP9_Cell.cell.adhesion.PerouLab.PMID.25109877	0.86	0.60	1.21	0.39	0.59
HCK_BCR.2008.PMID.19272155	2.08	1.48	2.96	<0.001	0.001
HER1.Cluster1_BMC.Genomics.2007.PMID.17663798	0.75	0.56	1.00	0.06	0.15
HER1.Cluster2_BMC.Genomics.2007.PMID.17663798	0.86	0.63	1.15	0.30	0.50
HER1.Cluster3_BMC.Genomics.2007.PMID.17663798	1.15	0.85	1.54	0.37	0.56
HER2.Amplicon.PerouLab_BMC.Med.Genomic.2011.PMID.21214954	1.76	1.31	2.40	<0.001	0.002
Histological.Grade_J.Pathol.2017.PMID.27861902	1.28	0.94	1.77	0.12	0.25
HouseKeeping_Genome.Biol.2004.PMID.15287981	1.02	0.78	1.33	0.90	0.95
iDC.Median_Immunity.2013.PMID.24138885	1.04	0.78	1.39	0.78	0.86
IFN.Cluster_BMC.Med.Genomics.2011.PMID.21214954	0.90	0.67	1.20	0.46	0.64
IgG_BCR.2008.PMID.19272155	1.32	1.03	1.70	0.03	0.09
IGG.Cluster_BMC.Med.Genomics.2011.PMID.21214954	1.34	1.03	1.76	0.03	0.10
Immature..B.cell_CellRep.2017.PMID.28052254	1.56	1.18	2.09	0.002	0.01
Immature.dendritic.cell_CellRep.2017.PMID.28052254	0.81	0.61	1.07	0.14	0.28
ImmLandscape_Macro.mono.CSF1.core.response_CCR.2009.PMID.29628290	1.94	1.44	2.68	<0.001	0.001

ImmLandscape_Wound.Healing_Immunity.2018.PMID.29628290	0.99	0.74	1.33	0.97	0.98
ImmLandscape.IFN3_Plos.One.2014.PMID.24516633	0.87	0.65	1.16	0.34	0.53
ImmLandscape.IFNG5_Plos.One.2014.PMID.24516633	1.71	1.29	2.28	<0.001	0.002
ImmLandscape.lymphocyte.Infil.T.B.PMID.18592372	1.66	1.25	2.23	0.001	0.004
Immune.Hot.CD8.vs.Cold_Nature.2020.PMID.31942071	1.81	1.36	2.46	<0.001	0.002
Immune.Perez.14_JCO.2015.PMID.25605861	1.81	1.34	2.50	<0.001	0.002
Immune.Perez.87_JCO.2015.PMID.25605861	1.75	1.32	2.36	<0.001	0.002
Immune.Suppression_JCI.Insight.2016.PMID.27699256	1.57	1.18	2.11	0.002	0.01
ImmuneActive_Cell.2019.PMID.31730857	1.81	1.35	2.45	<0.001	0.002
Immunosuppression.PMID.31942077	1.00	0.77	1.31	0.99	0.99
IMS.Score_CCR.2018.PMID.29921729	0.84	0.65	1.10	0.21	0.38
Induced.in.Bcells_PNAS.2013.PMID.23382184	1.57	1.17	2.15	0.004	0.02
Induced.in.DC_PNAS.2013.PMID.23382184	1.45	1.07	2.00	0.02	0.06
Induced.in.GN_PNAS.2013.PMID.23382184	1.52	1.10	2.13	0.01	0.05
Induced.in.HSC_PNAS.2013.PMID.23382184	0.96	0.74	1.24	0.76	0.85
Induced.in.MOs_PNAS.2013.PMID.23382184	1.65	1.22	2.27	0.001	0.008
Induced.in.NKcells_PNAS.2013.PMID.23382184	1.57	1.17	2.14	0.003	0.02
Induced.in.Tcells_PNAS.2013.PMID.23382184	1.44	1.09	1.92	0.01	0.04
Inflammatory.breast.cancer.491genes_CCR.2013.PMID.23396049	1.78	1.34	2.41	<0.001	0.002
Inflammatory.breast.cancer.79genes_CCR.2013.PMID.23396049	1.34	0.99	1.84	0.06	0.15
Inflammatory.breast.cancer.expressed.noIBC_79genes_CCR.2013.PMID.23396049	0.81	0.61	1.07	0.13	0.27
Inflammatory.breast.cancer.expressed.noIBC.491genes_CCR.2013.PMID.23396049	0.71	0.51	0.99	0.04	0.12
Influenza.11genes.Metasignature_Immunity.2015.PMID.26682989	0.93	0.69	1.25	0.63	0.76
Interferon_BCR.2008.PMID.19272155	0.83	0.62	1.11	0.22	0.40
Interferon.Pathway_CancerImmunolRes.2018.PMID.30266715	0.99	0.74	1.33	0.95	0.97
JUND.KRT5_Nat.Cell.Biol.2014.PMID.24658685	0.91	0.67	1.24	0.56	0.71
Keller2012.CD10.Adam_BCR.2015.PMID.25575446	1.13	0.85	1.52	0.40	0.60
KRAS.amplicon_Genome.Biology.2007.PMID.17493263	1.18	0.90	1.58	0.24	0.41
Late.IRS.1_PLoS.One.2016.PMID.26991655	1.26	0.94	1.71	0.12	0.25
Late.IRS.2_PLoS.One.2016.PMID.26991655	1.15	0.87	1.52	0.34	0.53
LCK_BCR.2008.PMID.19272155	1.78	1.33	2.42	<0.001	0.002
Lim2009.LumProg.Adam_BCR.2015.PMID.25575446	0.98	0.71	1.34	0.89	0.95
Lim2009.MaSC.Adam_BCR.2015.PMID.25575446	1.10	0.80	1.53	0.55	0.70
Lim2009.MatureLum.Adam_BCR.2015.PMID.25575446	0.92	0.67	1.25	0.60	0.74
Lim2009.Stroma.Adam_BCR.2015.PMID.25575446	1.01	0.73	1.40	0.96	0.98
Lim2010.LumProg.Adam_BCR.2015.PMID.25575446	1.62	1.24	2.16	0.001	0.004
Lim2010.MaSC.Adam_BCR.2015.PMID.25575446	1.11	0.82	1.51	0.50	0.67
Lim2010.MatureLum.Adam_BCR.2015.PMID.25575446	0.70	0.52	0.94	0.02	0.06
Lim2010.Stroma.Adam_BCR.2015.PMID.25575446	1.01	0.74	1.39	0.93	0.97
Lobular.Carcinoma.In.Situ_J.Pathol.2017.PMID.27861902	0.92	0.69	1.23	0.58	0.73
LOBULAR.TCGA.SIGNATURE.ImmuneCell.2015.PMID.26451490	1.93	1.39	2.73	<0.001	0.002
LOBULAR.TCGA.SIGNATURE.Reactive_Cell.2015.PMID.26451490	0.99	0.70	1.40	0.96	0.98
LOBULAR.TCGA.SUBTYPE.Immune_Cell.2015.PMID.26451490	0.94	0.55	1.59	0.81	0.89
LOBULAR.TCGA.SUBTYPE.Proliferative_Cell.2015.PMID.26451490	0.99	0.69	1.42	0.96	0.98
LOBULAR.TCGA.SUBTYPE.Reactive_Cell.2015.PMID.26451490	0.97	0.69	1.38	0.88	0.95
LTS.score_JCI.2020.PMID.32573490	0.70	0.50	0.96	0.03	0.08
Luminal_Progenitor_Up_Nat.Med.2009.PMID.19648928	1.45	1.06	2.01	0.02	0.07

Luminal.cluster_BMC.Med.Genomics.2011.PMID.21214954	0.90	0.64	1.26	0.54	0.69
Luminal.Progenitor_BCR.2010.PMID.20346151	1.33	0.98	1.82	0.07	0.17
Luminal.Progenitor.Down_Nat.Med.2009.PMID.19648928	1.06	0.81	1.37	0.68	0.80
LumProg.HsEnriched_BCR.2015.PMID.25575446	1.26	0.92	1.72	0.15	0.30
LumProg.HsEnriched.Refined1_BCR.2015.PMID.25575446	0.97	0.71	1.31	0.82	0.90
LumProg.Lim09_BCR.2015.PMID.25575446	1.18	0.89	1.60	0.26	0.44
LumProg.Prat_BCR.2015.PMID.25575446	1.11	0.81	1.51	0.53	0.69
LumProg.Shehata_BCR.2015.PMID.25575446	1.05	0.76	1.45	0.76	0.85
Lums.HER2E.DOWN.metastatic.signature_JCI.2020.PMID.32573490	0.75	0.57	0.97	0.03	0.10
Lums.HER2E.UP.metastatic.signature_JCI.2020.PMID.32573490	1.64	1.17	2.33	0.004	0.02
Lung.WNT_Cancer.Res.2009.PMID.19549913	1.11	0.82	1.49	0.49	0.67
Lymph.vessels_Immunity.2013.PMID.24138885	0.99	0.75	1.31	0.95	0.97
Lymphovascular.Invasion_J.Pathol.2017.PMID.27861902	1.08	0.82	1.41	0.60	0.74
M.D.Metagene_Genome.Biol.2013.PMID.23618380	1.83	1.38	2.49	<0.001	0.001
M2.Macrophage_Blood.2006.PMID.16556895	1.60	1.15	2.26	0.006	0.03
Macrophage_CellRep.2017.PMID.28052254	1.16	0.84	1.61	0.36	0.55
Macrophages_CancerImmunolRes.2018.PMID.30266715	1.84	1.32	2.59	<0.001	0.003
Macrophages_Immunity.2013.PMID.24138885	1.59	1.17	2.18	0.004	0.02
Macrophages.M0_Nat.Methods.2015.PMID.25822800	2.28	1.62	3.30	<0.001	0.001
Macrophages.M1_Nat.Methods.2015.PMID.25822800	1.59	1.20	2.14	0.002	0.009
Macrophages.M2_Nat.Methods.2015.PMID.25822800	1.65	1.20	2.32	0.003	0.01
MacTh1.cluster_CCR.2014.PMID.24916698	1.94	1.42	2.69	<0.001	0.001
MammaPrint_Nature.2002.PMID.11823860	0.77	0.58	1.02	0.08	0.18
MAPK.pathway.activation_NPJ.Precis.Oncol.2018.PMID.29872725	0.92	0.68	1.26	0.62	0.75
MASC.Down_Nat.Med.2009.PMID.19648928	0.85	0.61	1.19	0.36	0.55
MASC.Up_Nat.Med.2009.PMID.19648928	0.96	0.71	1.28	0.76	0.85
Mast.cell_CellRep.2017.PMID.28052254	1.32	1.01	1.75	0.05	0.12
Mast.cells_Immunity.2013.PMID.24138885	0.81	0.61	1.07	0.15	0.29
Mast.cells.activated_Nat.Methods.2015.PMID.25822800	1.08	0.80	1.46	0.62	0.75
Mast.cells.resting_Nat.Methods.2015.PMID.25822800	1.08	0.81	1.45	0.61	0.74
Mature.luminal_BCR.2010.PMID.20346151	0.99	0.73	1.33	0.93	0.97
Mature.Luminal.Down_Nat.Med.2009.PMID.19648928	1.11	0.81	1.52	0.53	0.69
Mature.LuminaUp_Nat.Med.2009.PMID.19648928	0.91	0.67	1.22	0.52	0.69
MatureLum.HsEnriched_BCR.2015.PMID.25575446	0.94	0.67	1.31	0.71	0.81
MatureLum.HsEnriched.Refined1_BCR.2015.PMID.25575446	0.99	0.73	1.34	0.97	0.98
MatureLum.Lim09_BCR.2015.PMID.25575446	0.92	0.66	1.26	0.60	0.74
MatureLum.Prat_BCR.2015.PMID.25575446	1.08	0.77	1.51	0.66	0.78
MatureLum.Shehata_BCR.2015.PMID.25575446	0.80	0.57	1.12	0.20	0.37
MBasal.Cluster_BMC.Med.Genomics.2011.PMID.21214954	1.01	0.74	1.38	0.95	0.97
MCD3.CD8_BMC.Med.Genomics.2011.PMID.21214954	1.73	1.28	2.37	<0.001	0.004
MCF7.E2.induced.genes_JCO.2006.PMID.16505416	0.80	0.61	1.04	0.10	0.23
MCF7.E2.repressed.genes_JCO.2006.PMID.16505416	0.86	0.62	1.18	0.34	0.53
MDSC_CellRep.2017.PMID.28052254	1.86	1.35	2.60	<0.001	0.002
MDSC.Granulocytic_Leukoc.Biol.2012.PMID.21954284	1.06	0.81	1.39	0.66	0.78
MDSC.Neutrophi_Leukoc.Biol.2012.PMID.21954284	1.68	1.26	2.29	0.001	0.004
MDSC.tumor_J.Immunol.2012.PMID.23152559	1.34	1.00	1.81	0.05	0.14
MDSC.tumor.MO_J.Immunol.2012.PMID.23152559	1.20	0.90	1.62	0.22	0.40

MECM_BMC.Med.Genomics.2011.PMID.21214954	0.92	0.67	1.26	0.60	0.74
Memory.B.cell_CellRep.2017.PMID.28052254	0.92	0.70	1.22	0.58	0.73
MET.DOWN.RNAseq.Significant.Genes_JCI.2018.PMID.29480819	1.22	0.90	1.66	0.21	0.38
MET.DOWN.Significant.Genes.Low.Basal.1_JCI.2018.PMID.29480819	0.88	0.67	1.14	0.33	0.52
MET.DOWN.Significant.Genes.Low.Basal.2_JCI.2018.PMID.29480819	1.11	0.80	1.56	0.52	0.69
MET.UP.RNAseq.Significant.Genes_JCI.2018.PMID.29480819	0.64	0.46	0.89	0.008	0.03
MET.UP.Significant.Genes.HIGH.BASALS.Genes_JCI.2018.PMID.29480819	0.81	0.62	1.05	0.11	0.24
Metaplastic.Up_CanRes.2009.PMID.19435916	1.27	0.92	1.77	0.16	0.31
Metastasis.predictor.TNBC_BCR.2010.PMID.20946665	1.47	1.09	2.00	0.01	0.05
MFGFR2_BMC.Med.Genomics.2011.PMID.21214954	1.04	0.78	1.39	0.80	0.88
MHC.Forero.11_Cancer.Immunol.Res.2016.PMID.26980599	1.55	1.19	2.07	0.002	0.01
MHC.Forero.24_Cancer.Immunol.Res.2016.PMID.26980599	1.31	1.01	1.72	0.04	0.12
MHC.I_BCR.2008.PMID.19272155	1.23	0.94	1.62	0.13	0.27
MHC.II_BCR.2008.PMID.19272155	1.98	1.46	2.75	<0.001	0.001
MHCI.coreGenes_Nat.Commun.2017.PMID29170503	1.43	1.08	1.92	0.01	0.05
MIR200c.Induced_ONCO.2015.PMID.25746005	1.29	0.97	1.73	0.09	0.21
MIR200c.Repressed_ONCO.2015.PMID.25746005	1.22	0.92	1.62	0.17	0.32
miRNA.138.signature_Cancer.Res.2014.PMID.25339353	1.15	0.85	1.55	0.36	0.55
MITO1_BMC.Med.Genomics.2011.PMID.21214954	1.65	1.14	2.43	0.009	0.03
MITO2_BMC.Med.Genomics.2011.PMID.21214954	1.24	0.93	1.66	0.14	0.28
Mitotic.Count_J.Pathol.2017.PMID.27861902	1.17	0.87	1.58	0.29	0.47
MK14.K17_BMC.Med.Genomics.2011.PMID.21214954	0.97	0.74	1.26	0.80	0.88
MKRAS.amplicon_BMC.Med.Genomics.2011.PMID.21214954	1.25	0.96	1.66	0.11	0.24
MM.BRCAwnt.1pFDR.UP_Genome.Biology.2007.PMID.17493263	0.82	0.58	1.15	0.25	0.43
MM.C3Tag.1pFDR.UP_Genome.Biology.2007.PMID.17493263	0.98	0.73	1.33	0.90	0.95
MM.C3Tag.2012_Genome.Biol.2013.PMID.24220145	1.37	1.02	1.86	0.04	0.11
MM.Class3_Genome.Biol.2013.PMID.24220145	1.02	0.77	1.35	0.89	0.95
MM.Class8_Genome.Biol.2013.PMID.24220145	0.83	0.64	1.07	0.15	0.30
MM.Claudinlow_Genome.Biol.2013.PMID.24220145	1.22	0.89	1.67	0.23	0.40
MM.DMBAwnt.1pFDR.UP.Genome.Biology.2007.PMID.17493263	1.14	0.86	1.52	0.37	0.56
MM.Erbb2.like_Genome.Biol.2013.PMID.24220145	1.03	0.75	1.41	0.86	0.92
MM.Myc.2012_Genome.Biol.2013.PMID.24220145	0.97	0.73	1.29	0.85	0.92
MM.Myoepithelioma.like_Genome.Biol.2013.PMID.24220145	1.20	0.89	1.61	0.23	0.40
MM.Neu.2012_Genome.Biol.2013.PMID.24220145	1.26	0.93	1.71	0.14	0.29
MM.NeuPyMT.1pFDR.UP_Genome.Biology.2007.PMID.17493263	1.12	0.81	1.56	0.50	0.67
MM.Normal.1pFDR.UP_Genome.Biology.2007.PMID.17493263	1.42	0.99	2.07	0.06	0.15
MM.Normal.like_Genome.Biol.2013.PMID.24220145	1.09	0.69	1.72	0.72	0.82
MM.p53null.1pFDR.UP_Genome.Biology.2007.PMID.17493263	1.34	0.99	1.82	0.06	0.15
MM.p53null.Basal_Genome.Biol.2013.PMID.24220145	1.04	0.79	1.37	0.79	0.88
MM.p53null.Luminal_Genome.Biol.2013.PMID.24220145	0.92	0.70	1.22	0.57	0.72
MM.Potluck_1pFDR_UP_Genome.Biology.2007_PMID.17493263.PMID.24220145	1.37	1.00	1.88	0.05	0.14
MM.PyMT.2012_Genome.Biol.2013.PMID.24220145	1.38	1.02	1.90	0.04	0.12
MM.Squamous.like_Genome.Biol.2013.PMID.24220145	1.17	0.89	1.55	0.26	0.44
MM.Stat1_Genome.Biol.2013.PMID.24220145	1.15	0.87	1.54	0.33	0.53
MM.WapINT3.1pFDR.UP_Genome.Biology.2007.PMID.17493263	1.08	0.81	1.44	0.62	0.75
MM.WapINT3.2012_Genome.Biol.2013.PMID.24220145	1.21	0.93	1.58	0.16	0.31
MM.WAPTag.1pFDR.UP_Genome.Biology.2007.PMID.17493263	1.30	0.95	1.81	0.11	0.24

MM.Wnt1.Early_Genome.Biol.2013.PMID.24220145	1.27	0.96	1.69	0.09	0.22
MM.Wnt1.Late_Genome.Biol.2013.PMID.24220145	1.11	0.85	1.46	0.45	0.63
Mmyosin_BMC.Med.Genomics.2011.PMID.21214954	0.56	0.39	0.79	0.001	0.007
MNADH_CYTochrome_BMC.Med.Genomics.2011.PMID.21214954	1.46	1.05	2.06	0.03	0.08
MNB1_BMC.Med.Genomics.2011.PMID.21214954	1.43	0.97	2.14	0.08	0.18
MNB2_BMC.Med.Genomics.2011.PMID.21214954	0.97	0.60	1.55	0.89	0.95
MNB3_BMC.Med.Genomics.2011.PMID.21214954	1.05	0.66	1.70	0.83	0.90
MNOtch4_BMC.Med.Genomics.2011.PMID.21214954	1.11	0.85	1.44	0.45	0.64
Monocyte_CellRep.2017.PMID.28052254	0.85	0.64	1.14	0.28	0.46
Monocyte..DC.25gene_Genome.Biol.2013.PMID.23618380	1.92	1.43	2.64	<0.001	0.001
Monocytes_CancerImmunolRes.2018.PMID.30266715	1.65	1.18	2.33	0.004	0.02
Monocytes_Nat.Methods.2015.PMID.25822800	1.89	1.36	2.68	<0.001	0.002
Monocytic.lineage.MCP_Nature.2020.PMID.31942075	1.59	1.15	2.22	0.006	0.02
MProliferation_BMC.Med.Genomics.2011.PMID.21214954	1.30	0.96	1.79	0.10	0.22
MProtocadherin_BMC.Med.Genomics.2011.PMID.21214954	1.37	1.02	1.87	0.04	0.12
MPYMT_NEU_Cluster_BMC.Med.Genomics.2011.PMID.21214954	1.33	0.99	1.80	0.06	0.15
MRibosomal_BMC.Med.Genomics.2011.PMID.21214954	0.79	0.54	1.13	0.20	0.36
MS.CD44.DOWN_PNAS.2009.PMID.19666588	0.86	0.62	1.19	0.36	0.56
MS.CD44.UP_PNAS.2009.PMID.19666588	1.12	0.82	1.54	0.47	0.65
MSquamous_BMC.Med.Genomics.2011.PMID.21214954	0.89	0.66	1.20	0.46	0.64
Murat.G07_JCO.2008.PMID.18565887	0.93	0.71	1.23	0.62	0.75
Murat.G18_JCO.2008.PMID.18565887	1.10	0.83	1.45	0.52	0.69
Murat.G24_JCO.2008.PMID.18565887	2.15	1.55	3.04	<0.001	0.001
MVEGFC_BMC.Med.Genomics.2011.PMID.21214954	0.89	0.66	1.17	0.40	0.59
Myeloid.cell.chemotaxis.1gene_Nature.2020.PMID.31942077	1.21	0.88	1.66	0.24	0.41
Myeloid.dendritic.cells.MCP_Nature.2020.PMID.31942077	1.34	1.01	1.81	0.05	0.13
Natural.killer.cell_CellRep.2017.PMID.28052254	1.06	0.79	1.42	0.70	0.81
Natural.killer.T.cell_CellRep.2017.PMID.28052254	1.34	0.99	1.81	0.06	0.15
Necrosis_J.Pathol.2017.PMID.27861902	1.19	0.89	1.60	0.24	0.41
Neutrophil_CellRep.2017.PMID.28052254	1.16	0.84	1.60	0.37	0.56
Neutrophils_CancerImmunolRes.2018.PMID.30266715	1.82	1.31	2.56	<0.001	0.004
Neutrophils_Immunity.2013.PMID.24138885	1.36	0.99	1.88	0.06	0.15
Neutrophils_Nat.Methods.2015.PMID.25822800	1.81	1.30	2.58	0.001	0.004
Neutrophils.MCP_Nature.2020.PMID.31942077	0.86	0.64	1.15	0.31	0.50
NK_Immunity.2013.PMID.24138885	0.85	0.64	1.11	0.23	0.41
NK.activated_Nat.Methods.2015.PMID.25822800	1.83	1.35	2.51	<0.001	0.002
NK.CD56bright_Immunity.2013.PMID.24138885	1.32	0.99	1.77	0.06	0.15
NK.CD56dim_Immunity.2013.PMID.24138885	1.68	1.24	2.31	0.001	0.006
NK.resting_Nat.Methods.2015.PMID.25822800	1.85	1.37	2.54	<0.001	0.002
NKcells_CancerImmunolRes.2018.PMID.30266715	1.65	1.25	2.20	<0.001	0.004
NKcells.MCP_Nature.2020.PMID.31942077	1.03	0.72	1.46	0.89	0.95
No.Response.Immunotherapy.TLS.Melanoma_Nature.2020.PMID.31942075	0.67	0.49	0.91	0.01	0.04
Normal.mucosa_Immunity.2013.PMID.24138885	0.83	0.60	1.13	0.24	0.41
Nuclear.Pleomorphism_J.Pathol.2017.PMID.27861902	0.93	0.71	1.22	0.60	0.74
Oncotype_NEJM.2004.PMID.15591335	1.58	1.17	2.17	0.004	0.02
P53.ERPos.MDACC_CCR.2011.PMID.21248301	1.27	0.94	1.73	0.12	0.25
Parity.signature.251genes_BCR.2014.PMID.25005139	1.87	1.37	2.62	<0.001	0.002

Parity.signature.40genes_BCR.2014.PMID.25005139	1.75	1.30	2.39	<0.001	0.003
PARPi.Resistance_BCRT_2012.PMID.22875744	1.05	0.81	1.38	0.70	0.81
PARPi.Sensitivity_BCRT_2012.PMID.22875744	0.78	0.57	1.05	0.11	0.24
PARPi.Sensitivity.MDACC_NPJ.Syst.Biol.Appl.2017.PMID.28649435	1.19	0.91	1.55	0.20	0.37
PARPi.Sensitivity.Negative_Sci.Adv.2017.PMID.28439535	1.08	0.82	1.42	0.58	0.73
PARPi.Sensitivity.Positive_Sci.Adv.2017.PMID.28439535	1.21	0.93	1.58	0.17	0.32
Pcorr.Breast2Lung.LM2.Correlation_Nature.2005.PMID.16049480	1.05	0.81	1.35	0.73	0.83
Pcorr.Breast2Lung.Parental.Correlation_Nature.2005.PMID.16049480	1.04	0.80	1.34	0.78	0.86
Pcorr.dasatinib.resistant_Cancer.Res.2007.PMID.17332353	1.05	0.77	1.42	0.77	0.86
Pcorr.dasatinib.sensitive_Cancer.Res.2007.PMID.17332353	0.95	0.70	1.29	0.74	0.83
Pcorr.Hypoxia.High.Correlation_PLoS.Med.2006.PMID.16417408	0.71	0.53	0.94	0.02	0.07
Pcorr.Hypoxia.Low.Correlation_PLoS.Med.2006.PMID.16417408	1.37	1.03	1.85	0.03	0.10
Pcorr.IGS_Invasiveness_NJEM.2007.PMID.17229949	1.60	1.18	2.19	0.003	0.02
Pcorr.wound.response.activated_PNAS.2005.PMID.15701700	1.07	0.81	1.40	0.64	0.77
pCR.predictor.ERNeg.55genes_JAMA.2011.PMID.21558518	1.38	1.04	1.87	0.03	0.09
pCR.predictor.ERPos.39genes_JAMA.2011.PMID.21558518	1.02	0.77	1.35	0.91	0.96
PDCD1_Single_Gene.Single	1.78	1.29	2.50	0.001	0.004
Pfefferle2012.LumProg_BCR.2015.PMID.25575446	1.36	0.98	1.90	0.07	0.17
Pfefferle2012.MaSC_BCR.2015.PMID.25575446	0.99	0.73	1.33	0.92	0.97
Pfefferle2012.MatureLum_BCR.2015.PMID.25575446	1.05	0.78	1.40	0.75	0.84
Pfefferle2012.Stroma_BCR.2015.PMID.25575446	1.07	0.79	1.45	0.68	0.80
PGR_Single_Gene.Single	0.69	0.50	0.94	0.02	0.07
PI3Ki.Down_CancerCell.2017.PMID.28528867	1.23	0.89	1.70	0.21	0.38
PI3Ki.Up_CancerCell.2017.PMID.28528867	1.16	0.81	1.65	0.42	0.61
PIK3CA.Pathway_Ann.Oncol.2017.PMID.28177460	1.37	0.99	1.91	0.06	0.15
PIK3CAmt.signature_Cancer.Res.2012.PMID.22552288	1.21	0.92	1.61	0.18	0.34
Plasma.cells_Nat.Methods.2015.PMID.25822800	1.37	1.05	1.79	0.02	0.07
PlasmaCells_CancerImmunolRes.2018.PMID.30266715	1.37	1.05	1.79	0.02	0.07
Plasmacytoid.dendritic.cell_CellRep.2017.PMID.28052254	0.95	0.70	1.29	0.75	0.84
PR.Isoform.Ratio.Up.in.PRA.H_JNCI.2017.PMID.28376177	0.81	0.59	1.11	0.19	0.36
PR.Isoform.Ratio.Up.in.PRB.H_JNCI.2017.PMID.28376177	1.28	0.95	1.74	0.11	0.24
Proliferation.Cluster_BMC.Med.Genomics.2011.PMID.21214954	1.35	0.98	1.87	0.07	0.16
Proliferation.Metagene_Genome.Biol.2013.PMID.23618380	1.34	0.98	1.84	0.07	0.18
Proliferation.score.PAM50_JCO.2009.PMID.19204204	1.35	1.01	1.82	0.04	0.12
ProliferationPathway_CancerImmunolRes.2018.PMID.30266715	1.31	0.96	1.80	0.10	0.22
Prosigna.Proliferation.18_BMC.Med.Genomics.2015.PMID.26297356	1.23	0.91	1.67	0.19	0.35
Race.LuminalA.MRE.score_BCRT.2015.PMID.26109344	0.95	0.73	1.23	0.69	0.81
Radiation.induced.genes_Radoat.Res.2014.PMID.24527691	1.23	0.86	1.76	0.26	0.44
RB.LOH_BCR.2008.PMID.18782450	1.11	0.83	1.51	0.49	0.67
RB.LOSS_JCI.2007.PMID.17160137	1.29	0.95	1.78	0.10	0.23
Regulatory.T.cell_CellRep.2017.PMID.28052254	1.75	1.26	2.47	0.001	0.006
Replication.Stress.Down.set_Cell.Rep.2018.PMID.29768207	0.99	0.74	1.33	0.94	0.97
Replication.Stress.Model_Cell.Rep.2018_PMID.29768207.PMID.29768207	1.09	0.83	1.43	0.53	0.69
Replication.Stress.Neg_Cell.Rep.2018_PMID.29768207.PMID.29768207	1.01	0.75	1.36	0.93	0.97
Replication.Stress.Pos_Cell.Rep.2018_PMID.29768207.PMID.29768207	1.07	0.82	1.42	0.60	0.74
Replication.Stress.Up_Set_Cell.Rep.2018_PMID.29768207.PMID.29768207	1.12	0.85	1.47	0.44	0.62
Residual.disease.predictor.ERNeg.54genes_JAMA.2011.PMID.21558518	0.92	0.70	1.20	0.54	0.69

Residual.disease.predictor.ERPos.73genes_JAMA.2011.PMID.21558518	1.11	0.82	1.51	0.49	0.67
Response.Immunotherapy.MCP.TLS.Melanoma_Nature.2020.PMID.31942075	1.74	1.32	2.32	<0.001	0.002
Response.Immunotherapy.signature_Science.2018.PMID.30309915	1.69	1.28	2.27	<0.001	0.003
Response.Neo.Chemo_common_CCR.2014.PMID.25047707	1.30	0.98	1.75	0.08	0.18
Response.Neo.Chemo_ERNeg_CCR.2014.PMID.25047707	0.82	0.63	1.05	0.12	0.25
Response.Neo.Chemo_ERPos_CCR.2014.PMID.25047707	1.00	0.77	1.29	0.99	0.99
RHOA.pathway_Ann.Oncol.2017.PMID.28177460	0.84	0.62	1.13	0.25	0.42
Ribosomal.Cluster_BMC.Med.Genomics.2011.PMID.21214954	0.84	0.58	1.21	0.35	0.55
ROR.subtype.PAM50_JCO.2009.PMID.19204204	1.89	1.33	2.74	0.001	0.004
ROR.subtype.proliferation.PAM50_JCO.2009.PMID.19204204	1.52	1.12	2.09	0.009	0.03
RSS.Score_CCR.2018.PMID.29921729	1.04	0.80	1.35	0.76	0.85
S100A9.A8_BMC.Med.Genomics.2011.PMID.21214954	1.69	1.22	2.39	0.002	0.01
Scorr.EMAT1.Correlation_BCR.2020.PMID.32641077	1.40	1.06	1.88	0.02	0.07
Scorr.EMAT2.Correlation_BCR.2020.PMID.32641077	0.73	0.57	0.93	0.01	0.04
Scorr.EMAT3.Correlation_BCR.2020.PMID.32641077	0.80	0.60	1.06	0.12	0.26
Scorr.EMAT4.Correlation_BCR.2020.PMID.32641077	1.35	1.01	1.83	0.04	0.12
Scorr.IE.Correlation_JCO.2006.PMID.16505416	0.58	0.42	0.78	0.001	0.004
Scorr.IIE.Correlation_JCO.2006.PMID.16505416	1.78	1.31	2.45	<0.001	0.003
Scorr.PAM50.Basal_JCO.2009.PMID.19204204	1.28	0.94	1.78	0.12	0.25
Scorr.PAM50.Her2_JCO.2009.PMID.19204204	2.14	1.52	3.07	<0.001	0.001
Scorr.PAM50.LumA_JCO.2009.PMID.19204204	0.58	0.40	0.82	0.002	0.01
Scorr.PAM50.LumB_JCO.2009.PMID.19204204	1.20	0.89	1.61	0.24	0.41
Scorr.PAM50.Normal_JCO.2009.PMID.19204204	0.69	0.50	0.94	0.02	0.07
Scorr.S329.L_Br.J.Cancer.2008.PMID.18382427	0.85	0.64	1.13	0.27	0.45
Scorr.S329.R_Br.J.Cancer.2008.PMID.18382427	1.27	0.94	1.71	0.12	0.25
Secretoglobin_BMC.Med.Genomics.2011.PMID.21214954	1.10	0.84	1.43	0.50	0.67
Shehata2012.ALDHneg_BCR.2015.PMID.25575446	1.16	0.89	1.51	0.28	0.46
Shehata2012.ALDHpos_BCR.2015.PMID.25575446	1.40	1.00	1.97	0.05	0.13
Shehata2012.Basal_BCR.2015.PMID.25575446	1.06	0.77	1.47	0.72	0.82
Shehata2012.Erbb3neg_BCR.2015.PMID.25575446	0.91	0.69	1.19	0.51	0.67
Shehata2012.LumProg_BCR.2015.PMID.25575446	1.19	0.86	1.64	0.29	0.48
Shehata2012.NCL_BCR.2015.PMID.25575446	0.88	0.64	1.21	0.45	0.63
Shehata2012.Stroma_BCR.2015.PMID.25575446	1.12	0.82	1.54	0.46	0.64
Spike2012.aMaSC_BCR.2015.PMID.25575446	1.00	0.76	1.33	0.98	0.99
Spike2012.fMaSC_BCR.2015.PMID.25575446	0.97	0.73	1.30	0.85	0.92
Spike2012.fStr_BCR.2015.PMID.25575446	0.88	0.61	1.26	0.48	0.66
STAT1_BCR.2008.PMID.19272155	1.65	1.23	2.23	0.001	0.006
STAT3.Basal_PNAS.2014.PMID.25139989	1.51	1.14	2.03	0.005	0.02
STAT3.Basal.short_PNAS.2014.PMID.25139989	1.56	1.17	2.09	0.003	0.01
Stroma.FNA.MDACC.1_JCO.2010.PMID.20805453	1.65	1.25	2.20	<0.001	0.004
Stroma.FNA.MDACC.2_JCO.2010.PMID.20805453	0.93	0.69	1.24	0.61	0.75
Stromal.Central.Fibrotic.Focus_J.Pathol.2017.PMID.27861902	1.03	0.80	1.35	0.80	0.88
Stromal.Down_Nat.Med.2009.PMID.19648928	0.75	0.53	1.07	0.12	0.25
Stromal.Inflammation_J.Pathol.2017.PMID.27861902	1.77	1.33	2.39	<0.001	0.002
Stromal.Signature_Nat.Med.2008.PMID.18438415	1.81	1.33	2.51	<0.001	0.002
Stromal.Up_Nat.Med.2009.PMID.19648928	1.06	0.77	1.45	0.72	0.82
SW480.cancer.cells_Immunity.2013.PMID.24138885	0.87	0.67	1.11	0.26	0.44

T.follicular.helper.cell_CellRep.2017.PMID.28052254	1.91	1.39	2.70	<0.001	0.002
Tcell.activation_Nature.2020.PMID.31942077	1.72	1.28	2.33	<0.001	0.003
Tcell.CD8.Effector.vs.naive.2_Science.2016.PMID27789795	1.31	0.96	1.81	0.09	0.21
Tcell.CD8.Exhausted.vs.antiPDL1.2_Science.2016.PMID27789795	1.47	1.09	2.00	0.01	0.04
Tcell.CD8.Exhausted.vs.naive.2_Science.2016.PMID27789795	1.36	1.00	1.87	0.05	0.14
Tcell.CD8.Memory.vs.naive.1_Science.2016.PMID27789795	1.99	1.48	2.74	<0.001	0.001
Tcell.cluster_CCR.2014.PMID.24916698	1.77	1.33	2.39	<0.001	0.002
Tcell.EXH.Anti.PDL1.vs.control.treated.exhausted.CD8.Tcell.Metagene.1.Science.2016.PMID.27789795	1.23	0.92	1.64	0.17	0.32
Tcell.EXH.Effector.CD8.T.cell.at.day.8.p.i.Armstrong.vs.Naive.CD8.Tcell.Metagene.1_Science.2016.PMID.27789795	1.86	1.38	2.53	<0.001	0.002
Tcell.EXH.Exhausted.CD8.T.cell.vs.Naive.CD8.T.cell.Metagene.1_Science.2016.PMID.27789795	1.57	1.17	2.14	0.003	0.02
Tcell.EXH.Exhausted.CD8.T.cell.vs.Naive.CD8.T.cell.Metagene.3_Science.2016.PMID.27789795	1.91	1.42	2.61	<0.001	0.001
Tcell.EXH.Memory.CD8.T.cell.a.vs.Naive.CD8.T.cell.Metagene.1_Science.2016.PMID.27789795	1.99	1.48	2.74	<0.001	0.001
Tcell.EXH.Memory.CD8.T.cell.a.vs.Naive.CD8.T.cell.Metagene.2_Science.2016.PMID.27789795	1.79	1.34	2.43	<0.001	0.002
Tcell.EXH.Memory.CD8.T.cell.a.vs.Naive.CD8.T.cell.Metagene.3.Science_2016.PMID.27789795	1.68	1.26	2.29	0.001	0.004
Tcell.NK.51gene_Genome.Biol.2013.PMID.23618380	1.70	1.28	2.29	<0.001	0.003
Tcell.NK.Metagene_Genome.Biol.2013.PMID.23618380	1.75	1.32	2.37	<0.001	0.002
Tcell.RM_Nat_Med.2018.PMID.29942092	1.87	1.40	2.56	<0.001	0.001
Tcell.survival.2gene_Nature.2020.PMID.31942077	1.58	1.17	2.16	0.003	0.02
Tcells_CancerImmunolRes.2018.PMID.30266715	1.79	1.34	2.43	<0.001	0.002
Tcells_Immunity.2013.PMID.24138885	1.78	1.33	2.43	<0.001	0.002
Tcells_TFH_Nat.Methods.2015.PMID.25822800	1.81	1.35	2.49	<0.001	0.002
Tcells.CD4.memory.activated_Nat.Methods.2015.PMID.25822800	1.91	1.42	2.60	<0.001	0.001
Tcells.CD4.memory.resting_Nat.Methods.2015.PMID.25822800	1.66	1.24	2.24	0.001	0.005
Tcells.CD4.naive_Nat.Methods.2015.PMID.25822800	1.70	1.26	2.32	0.001	0.004
Tcells.CD8_Immunity.2013.PMID.24138885	1.17	0.90	1.54	0.24	0.42
Tcells.CD8_Nat.Methods.2015.PMID.25822800	1.81	1.35	2.46	<0.001	0.002
Tcells.CD8.MCP_Nature.2020.PMID.31942075	1.42	1.07	1.91	0.02	0.06
Tcells.Cytotoxic.MCP_Nature.2020.PMID.31942075	1.71	1.29	2.30	<0.001	0.002
Tcells.gammadelta_Nat.Methods.2015.PMID.25822800	1.85	1.37	2.53	<0.001	0.002
Tcells.helper_Immunity.2013.PMID.24138885	1.25	0.93	1.69	0.15	0.30
Tcells.MCP_Nature.2020.PMID.31942077	1.80	1.35	2.43	<0.001	0.002
Tcells.regulatory.2gene_Nature.2020.PMID.31942077	1.51	1.12	2.07	0.008	0.03
Tcells.Tregs_Nat.Methods.2015.PMID.25822800	1.69	1.26	2.30	0.001	0.004
TCGA.BRCA.1198_BASAL_JCI.2020.PMID.32573490	0.94	0.70	1.27	0.70	0.81
TCGA.BRCA.1198_Chromogranin_JCI.2020.PMID.32573490	0.93	0.71	1.21	0.60	0.74
TCGA.BRCA.1198_COLLAGEN11A_JCI.2020.PMID.32573490	1.03	0.78	1.36	0.84	0.91
TCGA.BRCA.1198_EN1_FDZ9_JCI.2020.PMID.32573490	1.10	0.83	1.48	0.50	0.67
TCGA.BRCA.1198_FGFR4_EGF_JCI.2020.PMID.32573490	1.45	1.07	1.99	0.02	0.06
TCGA.BRCA.1198_HISTONES_JCI.2020.PMID.32573490	0.83	0.63	1.09	0.18	0.34
TCGA.BRCA.1198_HOXC11_HOTAIR_SIX1_JCI.2020.PMID.32573490	1.12	0.84	1.50	0.43	0.62
TCGA.BRCA.1198_IL8_CCL_JCI.2020.PMID.32573490	1.27	0.94	1.73	0.12	0.25
TCGA.BRCA.1198_immune_CD19_JCI.2020.PMID.32573490	1.32	1.02	1.74	0.04	0.11
TCGA.BRCA.1198_immune_CD34_TIE1_JCI.2020.PMID.32573490	1.12	0.81	1.56	0.49	0.67
TCGA.BRCA.1198_immune_CD4_CD53_CD84_BTK_JCI.2020.PMID.32573490	1.93	1.42	2.67	<0.001	0.001
TCGA.BRCA.1198_immune_CD8_GZMK_JCI.2020.PMID.32573490	1.77	1.33	2.40	<0.001	0.002
TCGA.BRCA.1198_immune_CTLA4_CXCL_FOXP3_JCI.2020.PMID.32573490	1.69	1.27	2.29	<0.001	0.004
TCGA.BRCA.1198_immune_FOS_JUN_IL6_JCI.2020.PMID.32573490	1.00	0.76	1.33	0.99	0.99

TCGA.BRCA.1198_immune_GIMAP_IL16_JCI.2020.PMID.32573490	1.77	1.31	2.44	<0.001	0.003
TCGA.BRCA.1198_immune_HLA_A_F_JCI.2020.PMID.32573490	1.60	1.19	2.18	0.002	0.01
TCGA.BRCA.1198_immune_HLA_D_JCI.2020.PMID.32573490	1.83	1.36	2.51	<0.001	0.002
TCGA.BRCA.1198_immune_INTERFERON_JCI.2020.PMID.32573490	0.90	0.67	1.20	0.46	0.64
TCGA.BRCA.1198_IMMUNE1_JCI.2020.PMID.32573490	1.38	1.06	1.80	0.02	0.06
TCGA.BRCA.1198_LUMINAL_JCI.2020.PMID.32573490	0.70	0.48	1.00	0.05	0.14
TCGA.BRCA.1198_MYBL2_APOBEC3B_JCI.2020.PMID.32573490	1.59	1.18	2.17	0.003	0.01
TCGA.BRCA.1198_NORMAL_JCI.2020.PMID.32573490	1.04	0.68	1.60	0.86	0.92
TCGA.BRCA.1198_NORMAL2_JCI.2020.PMID.32573490	1.14	0.82	1.59	0.44	0.62
TCGA.BRCA.1198_PDCHA_MANY_JCI.2020.PMID.32573490	0.92	0.71	1.18	0.49	0.67
TCGA.BRCA.1198_S100A7_8_9_JCI.2020.PMID.32573490	1.14	0.86	1.52	0.37	0.56
TCGA.BRCA.1198_TP63_JCI.2020.PMID.32573490	1.18	0.85	1.64	0.33	0.53
TCGA.BRCA.1198.IMMUNOGLOBULIN_JCI.2020.PMID.32573490	1.33	1.03	1.73	0.03	0.09
TCGA.CSF1.response_Immunity.2018.PMID.29628290	1.94	1.44	2.68	<0.001	0.001
TCGA.IFN.score_Immunity.2018.PMID.29628290	0.87	0.65	1.16	0.34	0.53
TCGA.Liexpression.score_Immunity.2018.PMID.29628290	1.66	1.25	2.23	0.001	0.004
TCGA.Serum.response.up_Immunity.2018.PMID.29628290	1.12	0.82	1.53	0.49	0.67
TCGA.TFH_Immunity.2018.PMID.29628290	1.51	1.14	2.04	0.005	0.02
TCGA.Tgd_Immunity.2018.PMID.29628290	0.66	0.46	0.93	0.02	0.07
TCGA.TGFB.score_Immunity.2018.PMID.29628290	0.88	0.67	1.14	0.33	0.53
Tcm_Immunity.2013.PMID.24138885	1.37	1.05	1.81	0.02	0.07
Tem_Immunity.2013.PMID.24138885	1.09	0.82	1.45	0.54	0.69
TFH_Immunity.2013.PMID.24138885	1.51	1.14	2.04	0.005	0.02
Tgd_Immunity.2013.PMID.24138885	0.66	0.46	0.93	0.02	0.07
Th1_cells_Immunity.2013.PMID.24138885	1.76	1.31	2.39	<0.001	0.002
Th17_cells_Immunity.2013.PMID.24138885	0.87	0.67	1.13	0.30	0.49
Th2_cells_Immunity.2013.PMID.24138885	1.13	0.86	1.51	0.39	0.58
TLS.9Gene.Signature_Nature.2020.PMID.31942071	1.67	1.21	2.34	0.002	0.01
TLS.CXCL13.SingleGene_Nature.2020.PMID.31942077	1.55	1.18	2.06	0.002	0.01
TLS.Hallmark.Gene.Signature_Nature.2020.PMID.31942071	1.66	1.22	2.30	0.001	0.008
TLS.Known.Markers_Nature.2020.PMID.31942071	1.64	1.22	2.22	0.001	0.007
TLS.Structure.12chemokine_FrontImmunol.2017.PMID.28713385	1.71	1.29	2.32	<0.001	0.003
TLS.tumors.wTLS.and.CD8.vs.CD8alone_Nature.2020.PMID.31942071	1.71	1.27	2.32	<0.001	0.004
TNBC.good.prognosis.TNBC.230genes_BCR.2011.PMID.21978456	1.02	0.76	1.37	0.90	0.95
TNBC.good.prognosis.TNBC.26genes_BCR.2011.PMID.21978456	1.30	1.00	1.69	0.05	0.13
TNBC.metastasis.free.survival_PLoS.One.2013.PMID.24349199	1.29	0.97	1.73	0.08	0.20
TNBC.poor.prognosis.TNBC.26genes_BCR.2011.PMID.21978456	1.05	0.78	1.41	0.77	0.86
Translation.Pathway_CancerImmunolRes.2018.PMID.30266715	0.85	0.58	1.24	0.41	0.60
Tumour.hypoxia.causes.DNA.hypermethylation_Nature.2016.PMID.27533040	0.79	0.59	1.05	0.10	0.23
Type.1.T.helper.cell_CellRep.2017.PMID.28052254	1.88	1.39	2.57	<0.001	0.001
Type.17.T.helper.cell_CellRep.2017.PMID.28052254	1.92	1.43	2.64	<0.001	0.001
Type.2.T.helper.cell_CellRep.2017.PMID.28052254	1.35	1.01	1.81	0.04	0.12
Up.Basal.High_Nat.Cell.Biol.2014.PMID.25173976	0.92	0.71	1.20	0.54	0.69
Up.Proliferation_Nat.Cell.Biol.2014.PMID.25173976	1.29	0.95	1.76	0.11	0.25
Upregulated.by.oncogenic.NRAS.basal_Cell.Rep.2016.PMID.26166574	0.99	0.74	1.32	0.93	0.97
Upregulated.upon.NRAS.repression.basal_Cell.Rep.2017.PMID.26166574	0.83	0.62	1.10	0.20	0.37
Vascular.Content_Clin.Exp.Metastasis.2014.PMID.23975155	0.99	0.76	1.29	0.94	0.97

VEGF.13genes_BMC.Med.2009.PMID.19291283	0.69	0.49	0.96	0.03	0.08
Wirapati.Proliferation_BCR.2008.PMID.18662380	1.26	0.93	1.73	0.14	0.29
Wound.Signature_CCR.2009.PMID.19887484	1.06	0.81	1.39	0.68	0.80
X11q13.Amplicon_BMC.Med.Genomics.2011.PMID.21214954	1.06	0.77	1.48	0.71	0.82
X12qMDM4.BMC.Med.Genomics.2011.PMID.21214954	1.08	0.83	1.41	0.58	0.73
X13q14.Amplicon_BMC.Med.Genomics.2011.PMID.21214954	1.00	0.76	1.31	0.98	0.99
X15q25.Amplicon_BMC.Med.Genomics.2011.PMID.21214954	1.15	0.84	1.58	0.37	0.56
X16.13.Amplicon_BMC.Med.Genomics.2011.PMID.21214954	0.58	0.42	0.80	0.001	0.006
X16q23.Amplicon_BMC.Med.Genomics.2011.PMID.21214954	0.99	0.73	1.33	0.93	0.97
X17PP13.Amplicon_BMC.Med.Genomics.2011.PMID.21214954	1.35	1.01	1.85	0.05	0.13
X17q25x.BMC.Med.Genomics.2011.PMID.21214954	0.77	0.58	1.02	0.08	0.18
X19p13.Amplicon_BMC.Med.Genomics.2011.PMID.21214954	0.84	0.64	1.09	0.19	0.36
X1p36.Amplicon_BMC.Med.Genomics.2011.PMID.21214954	0.89	0.65	1.19	0.43	0.62
X3p21.Amplicon_BMC.Med.Genomics.2011.PMID.21214954	0.73	0.52	1.02	0.07	0.16
X4p16.Amplicon_BMC.Med.Genomics.2011.PMID.21214954	1.08	0.81	1.43	0.61	0.75
X5Q_BCRT.2012.PMID.22048815	0.83	0.60	1.14	0.24	0.42
X8p.Amplicon_BMC.Med.Genomics.2011.PMID.21214954	1.11	0.85	1.46	0.45	0.63
X8p22.Amplicon_BMC.Med.Genomics.2011.PMID.21214954	0.80	0.59	1.07	0.13	0.27
XBP1.Signature_Nature.2014.PMID.24670641	1.11	0.82	1.50	0.51	0.68

eTable 9
Association of gene expression signatures at baseline with EFS in the combined cohort,
CALGB 40601, NeoALTTO, and NSABP B-41.
The 10 gene expression signatures that were significantly associated with EFS in the combined cohort are underlined.
Cox regression multivariable models adjusted by treatment arm, stage, node status, and HR status have been built
for each gene expression biomarker. Models built using the combined cohort have been stratified by study
Adjusted p-values for multiple testing using a Benjamini & Hochberg method to control the False Discovery Rate are provided
HR: hazard ratio; CI: confident interval.
The biomarkers significantly associated with EFS in the combined cohort are higlighted in blue.

	Combined cohort				
**Signature**	**HR**	**95% CI**		***P***	**adjusted *P***
Activate.Endothelium_Clin.Exp.Metastasis.2014.PMID.23975155	0.96	0.81	1.13	0.60	0.84
Activated.B.cell_CellRep.2017.PMID.28052254	0.79	0.66	0.94	0.007	0.10
Activated.Blood.Neutrophil.Signature_Nat.Cell.Biol.2019.PMID.31263265	0.96	0.82	1.13	0.66	0.86
Activated.Cancer.Cell.Signature_Nat.Cell.Biol.2019.PMID.31263265	1.04	0.88	1.24	0.63	0.85
Activated.CD4.T.cell_CellRep.2017.PMID.28052254	0.87	0.74	1.03	0.10	0.35
Activated.CD8.T.cell_CellRep.2017.PMID.28052254	0.77	0.65	0.91	0.002	0.08
Activated.dendritic.cell_CellRep.2017.PMID.28052254	0.73	0.62	0.87	<0.001	0.04
Activated.Lung.MSC.Signature_Nat.Cell.Biol.2019.PMID.31263265	1.25	1.05	1.49	0.01	0.11
Activated.Lung.Neutrophil.Signature_Nat.Cell.Biol.2019.PMID.31263265	1.00	0.84	1.19	0.99	>0.99
aDC_Immunity.2013_PMID.24138885.PMID.24138885	0.79	0.67	0.94	0.006	0.10
ADM.S100A10.A110NDGR1.Cluster_BMC.Med.Genomics.2011.PMID.21214954	0.98	0.82	1.17	0.82	0.94
African.and.European.Ancestry.TCGA.Negative_JAMA.Oncol.2017.PMID.28472234	1.04	0.88	1.23	0.64	0.85
African.and.European.Ancestry.TCGA.Positive_JAMA.Oncol.2017.PMID.28472234	1.01	0.85	1.19	0.95	>0.99
Age.associated.signature_Genome.Biol.2015.PMID.26343147	1.18	1.00	1.39	0.05	0.23
aMaSC_BCR.2010.PMID.20346151	1.14	0.96	1.35	0.14	0.43
aMaSC.HsEnriched_BCR.2015.PMID.25575446	1.16	0.98	1.37	0.08	0.30
aMaSC.HsEnriched.Refined1_BCR.2015.PMID.25575446	1.19	1.01	1.40	0.04	0.17
aMaSC.Lim09_BCR.2015.PMID.25575446	1.04	0.88	1.24	0.63	0.85
aMaSC.Prat_BCR.2015.PMID.25575446	1.04	0.88	1.24	0.63	0.85
aMaSC.Shehata_BCR.2015.PMID.25575446	0.93	0.79	1.09	0.36	0.70
aMaSC.Signature_Cell.Stem.Cell.2012.PMID.22305568	1.09	0.92	1.30	0.32	0.67
AMPH.EPIREGULIN.Cluster_BMC.Med.Genomics.2011.PMID.21214954	1.09	0.92	1.29	0.30	0.66
Amplification.50_Genome.Biol.2014.PMID.25164602	1.04	0.88	1.22	0.63	0.85
Amplification.50.better.than._Genome.Biol.2015.PMID.25164602	1.04	0.88	1.22	0.68	0.87
Apocrine.Features_J.Pathol.2017.PMID.27861902	1.00	0.84	1.19	0.98	>0.99
aStr.HsEnriched_BCR.2015.PMID.25575446	1.01	0.86	1.20	0.87	0.96
aStr.HsEnriched.Refined1_BCR.2015.PMID.25575446	1.16	0.98	1.38	0.08	0.31
aStr.HsEnriched.Refined2_BCR.2015.PMID.25575446	1.04	0.88	1.23	0.62	0.85
aStr.Lim09_BCR.2015.PMID.25575446	1.07	0.90	1.26	0.46	0.76
aStr.Prat_BCR.2015.PMID.25575446	1.02	0.86	1.21	0.85	0.95
aStr.Shehata_BCR.2015.PMID.25575446	1.03	0.87	1.23	0.72	0.90
BASAL.Cluster_BMC.Med.Genomics.2011.PMID.21214954	1.13	0.95	1.35	0.16	0.45
Bcell.cluster_CCR.2014.PMID.24916698	0.76	0.64	0.91	0.003	0.08
Bcell.IL10.MINUS_Immunol.2014.PMID.25080484	0.89	0.75	1.05	0.18	0.50
Bcell.IL10.PLUS_Immunol.2014.PMID.25080484	0.86	0.72	1.02	0.09	0.33

Bcell.lineage.MCP_Nature.2020.PMID.31942077	0.76	0.64	0.91	0.002	0.08
Bcell.Plasma.52gene_Genome.Biol.2013.PMID.23618380	0.71	0.60	0.84	<0.001	0.02
Bcell.Plasma.Metagene_Genome.Biol.2013.PMID.23618380	0.76	0.65	0.90	0.001	0.07
Bcell.Tcell.Cooperation_Cell.2019.PMID.31730857	0.80	0.67	0.96	0.01	0.11
Bcells_CancerImmunolRes.2018.PMID.30266715	0.76	0.64	0.90	0.002	0.08
Bcells_Immunity.2013.PMID.24138885	0.86	0.73	1.03	0.11	0.36
Bcells.Centroblast_JCO.2015.PMID.25800755	0.94	0.80	1.11	0.46	0.76
Bcells.Centrocyte_JCO.2015.PMID.25800755	0.98	0.83	1.16	0.84	0.95
Bcells.Memory_JCO.2015.PMID.25800755	0.88	0.74	1.04	0.14	0.43
Bcells.memory_Nat.Methods.2015.PMID.25822800	0.78	0.66	0.93	0.006	0.10
Bcells.Naive_JCO.2015.PMID.25800755	1.05	0.88	1.25	0.57	0.82
Bcells.naive_Nat.Methods.2015.PMID.25822800	0.81	0.68	0.97	0.02	0.13
Bcells.Plasmablast_JCO.2015.PMID.25800755	0.90	0.75	1.06	0.21	0.54
Blood.vessels_Immunity.2013.PMID.24138885	1.13	0.96	1.33	0.14	0.43
bMYB.Signature_Oncogene.2009.PMID.19043454	0.96	0.81	1.13	0.62	0.85
C3TAG.Responding_CCR.2013.PMID.23780888	0.90	0.76	1.07	0.25	0.59
C3TAG.Untreated_CCR.2013.PMID.23780888	0.96	0.81	1.14	0.63	0.85
CD103.Negative_Cancer.Cell.2014.PMID.25446897	0.93	0.78	1.10	0.38	0.71
CD103.Positive_Cancer.Cell.2014.PMID.25446897	0.86	0.73	1.01	0.07	0.28
CD103.Ratio_Cancer.Cell.2014.PMID.25446897	0.87	0.73	1.02	0.09	0.33
CD274_Single_Gene.Single	0.76	0.64	0.90	0.002	0.08
CD34.CD36.Cluster_BMC.Med.Genomics.PMID.21214954	1.00	0.85	1.19	0.97	>0.99
CD44.downregulated.genes_Cancer.Cell.2007.PMID.17349583	0.89	0.75	1.05	0.17	0.48
CD44.upregulated.genes_Cancer.Cell.2007.PMID.17349583	1.25	1.06	1.47	0.009	0.10
CD56bright.natural.killer.cell_CellRep.2017.PMID.28052254	0.96	0.82	1.14	0.65	0.86
CD56dim.natural.killer.cell_CellRep.2017.PMID.28052254	0.96	0.81	1.12	0.59	0.84
CD68.cluster_CCR.2014.PMID.24916698	0.99	0.84	1.17	0.93	0.99
CD8.cluster_CCR.2014.PMID.24916698	0.79	0.67	0.94	0.008	0.10
CDKN2A_Single_Gene.Single	1.20	1.03	1.40	0.02	0.13
Central.memory.CD4.T.cell_CellRep.2017.PMID.28052254	1.02	0.87	1.20	0.77	0.92
Central.memory.CD8.T.cell_CellRep.2017.PMID.28052254	0.90	0.76	1.06	0.21	0.54
CES.Score_CCR.2017.PMID.27903675	0.98	0.78	1.22	0.84	0.95
Chromogramin_BMC.Med.Genomics.2011.PMID.21214954	1.00	0.85	1.17	0.98	>0.99
CIN70_Nat.Genet.2006.PMID.16921376	0.99	0.84	1.17	0.93	0.99
Claudin.High_Genome.Biol.2007.PMID.17493263	0.81	0.68	0.97	0.02	0.13
Claudin.Low_Genome.Biol.2007.PMID.17493263	1.10	0.92	1.32	0.29	0.65
Claudin.Low.29_Cancer.Res.2009.PMID.19435916	1.06	0.89	1.27	0.53	0.80
cMYB.Signature_PLoS.One.2010.PMID.20949095	0.96	0.81	1.13	0.60	0.84
CORE.Bcell.signature.Garber_Cell.Mol.Gastroenterol.Hepatol.2017.PMID.28508029	0.81	0.67	0.96	0.02	0.12
CTLA4_Single_Gene.Single	0.80	0.68	0.95	0.009	0.10
Cytolytic.activity_Cell.2015.PMID.25594174	0.78	0.67	0.92	0.003	0.08
Cytotoxic.cells_Immunity.2013.PMID.24138885	0.82	0.69	0.97	0.02	0.13
Day7.Downregulated_Nat.Cell.Biol.2014.PMID.25173976	1.02	0.85	1.21	0.86	0.95
Day7.Upregulated_Nat.Cell.Biol.2014.PMID.25173976	1.00	0.85	1.18	0.97	>0.99
DC_Immunity.2013.PMID.24138885	0.83	0.70	0.99	0.04	0.17
DCIS.HGF.down_BCR.2013.PMID.24025166	1.10	0.93	1.31	0.25	0.60
DCIS.HGF.up_BCR.2014.PMID.24025166	1.07	0.91	1.26	0.42	0.74
Delection.50_Genome.Biol.2016.PMID.25164602	1.07	0.90	1.27	0.45	0.76

Delection.50.better.than_Genome.Biol.2017.PMID.25164602	1.15	0.97	1.35	0.10	0.36
Dendritic.cells.activated_Nat.Methods.2015.PMID.25822800	0.86	0.72	1.02	0.09	0.33
Dendritic.cells.resting_Nat.Methods.2015.PMID.25822800	0.84	0.70	1.00	0.05	0.21
Down.Basal.High_Nat.Cell.Biol.2014.PMID.25173976	1.01	0.85	1.20	0.93	0.99
Down.CLOW.High_Nat.Cell.Biol.2014.PMID.25173976	1.14	0.97	1.34	0.10	0.36
Downregulated.upon.NRAS.repression.basal_Cell.Rep.2015.PMID.26166574	0.88	0.74	1.03	0.12	0.39
Ductal.Carcinoma.In.Situ_J.Pathol.2017.PMID.27861902	0.90	0.76	1.06	0.20	0.53
Duke.Module01.acidosis_PNASUSA.2010.PMID.20335537	0.93	0.78	1.11	0.41	0.72
Duke.Module02.akt_PNASUSA.2010.PMID.20335537	0.83	0.71	0.96	0.01	0.11
Duke.Module03.betacatenin_PNASUSA.2010.PMID.20335537	0.92	0.78	1.08	0.31	0.66
Duke.Module04.E2F1_PNASUSA.2010.PMID.20335537	0.86	0.73	1.01	0.07	0.29
Duke.Module05.EGFR_PNASUSA.2010.PMID.20335537	1.01	0.86	1.19	0.90	0.97
Duke.Module06.ER_PNASUSA.2010.PMID.20335537	1.00	0.82	1.21	0.99	>0.99
Duke.Module07.glucosedepletion_PNASUSA.2010.PMID.20335537	0.83	0.71	0.98	0.03	0.15
Duke.Module08.HER2_PNASUSA.2010.PMID.20335537	0.96	0.81	1.13	0.62	0.85
Duke.Module09.hypoxia_PNASUSA.2010.PMID.20335537	0.97	0.83	1.15	0.74	0.90
Duke.Module10.IFNA_PNASUSA.2010.PMID.20335537	1.05	0.89	1.23	0.60	0.84
Duke.Module11.IFNG_PNASUSA.2010.PMID.20335537	0.99	0.84	1.17	0.89	0.97
Duke.Module12.lacticacidosis_PNASUSA.2010.PMID.20335537	1.11	0.94	1.31	0.23	0.58
Duke.Module13.myc_PNASUSA.2010.PMID.20335537	0.94	0.80	1.11	0.45	0.76
Duke.Module14.p53_PNASUSA.2010.PMID.20335537	0.90	0.75	1.09	0.27	0.62
Duke.Module15.p63_PNASUSA.2010.PMID.20335537	0.88	0.74	1.04	0.13	0.42
Duke.Module16.pi3k_PNASUSA.2010.PMID.20335537	0.95	0.81	1.12	0.52	0.80
Duke.Module17.PR_PNASUSA.2010.PMID.20335537	1.13	0.92	1.39	0.24	0.58
Duke.Module18.ras_PNASUSA.2010.PMID.20335537	0.93	0.79	1.08	0.34	0.69
Duke.Module19.src_PNASUSA.2010.PMID.20335537	1.05	0.90	1.23	0.50	0.79
Duke.Module20.STAT3_PNASUSA.2010.PMID.20335537	0.95	0.79	1.14	0.56	0.82
Duke.Module21.TGFB_PNASUSA.2010.PMID.20335537	1.01	0.86	1.20	0.87	0.96
Duke.Module22.TNFA_PNASUSA.2010.PMID.20335537	0.95	0.80	1.13	0.58	0.83
Durvalumab.signature_CCR.2018.PMID.29716923	0.83	0.71	0.98	0.03	0.16
Early.IRS.1_PLoS.One.2016.PMID.26991655	0.98	0.83	1.16	0.84	0.95
Early.IRS.2_PLoS.One.2016.PMID.26991655	1.09	0.92	1.28	0.34	0.69
Early.Relapse.ERPos.33genes_JAMA.2011.PMID.21558518	0.96	0.81	1.13	0.63	0.85
Early.Response.ERNeg.27genes_JAMA.2011.PMID.21558518	1.00	0.85	1.18	0.99	>0.99
Effector.memeory.CD4.T.cell_CellRep.2017.PMID.28052254	0.94	0.80	1.11	0.50	0.78
Effector.memeory.CD8.T.cell_CellRep.2017.PMID.28052254	0.83	0.70	0.98	0.03	0.15
EGFR_Single_Gene.Single	1.14	0.96	1.36	0.13	0.41
EMT.down.Taube_PNAS.2010.PMID.20713713	0.93	0.79	1.09	0.36	0.70
EMT.down.Weingberg_PNAS.2010.PMID.20713713	1.09	0.92	1.30	0.32	0.67
EMT.up.Taube_PNAS.2010.PMID.20713713	1.05	0.89	1.24	0.58	0.83
EMT.up.Weinberg_PNAS.2010.PMID.20713713	1.11	0.93	1.32	0.26	0.61
Endothelial.cells.MCP_Nature.2020..PMID.31942077	1.21	1.03	1.43	0.02	0.13
Endothelial.Normal_Angiogenesis.2014.PMID.24257808	0.97	0.82	1.15	0.74	0.91
Endothelial.Tumor_Angiogenesis.2014.PMID.24257808	0.94	0.80	1.11	0.48	0.77
Eosinophil_CellRep.2017.PMID.28052254	0.83	0.71	0.97	0.02	0.13
Eosinophils_Immunity.2013.PMID.24138885	0.97	0.82	1.14	0.71	0.89
Eosinophils_Nat.Methods.2015.PMID.25822800	0.78	0.66	0.91	0.002	0.08
Epithelial.Tubule.Formation_J.Pathol.2017.PMID.27861902	0.98	0.83	1.16	0.85	0.95

ERBB2_Single_Gene.Single	0.94	0.80	1.11	0.48	0.77
ERBB3_Single_Gene.Single	1.13	0.94	1.35	0.19	0.52
ESR1_Single_Gene.Single	0.99	0.79	1.25	0.96	>0.99
ESTIMATE.Immune_Nat.Commun.2013.PMID.24113773	0.81	0.68	0.97	0.02	0.12
ESTIMATE.Stromal_Nat.Commun.2013.PMID.24113773	0.97	0.82	1.15	0.73	0.90
Euclidean.Distance.CLOW_BCR.2010.PMID.20813035	0.97	0.82	1.16	0.77	0.92
EXTENDED.Bcell.signature.Garber_Cell.Mol.Gastroenterol.Hepatol.2017.PMID.28508029	0.82	0.70	0.97	0.02	0.12
FGFR4_Single_Gene.Single	1.07	0.90	1.26	0.45	0.76
FGFR4.Induced_JCI.2020.PMID.32573490	0.88	0.75	1.04	0.14	0.43
FGFR4.Repressed_JCI.2020.PMID.32573490	0.93	0.78	1.11	0.43	0.74
Fibrinogen.Cluster_BMC.Med.Genomics.2011.PMID.21214954	1.05	0.90	1.23	0.53	0.80
Fibroblast.Cluster_BMC.Med.Genomics.2011.PMID.21214954	1.15	0.97	1.37	0.11	0.38
Fibroblasts.MCP_Nature.2020.PMID.31942077	1.08	0.91	1.29	0.36	0.70
Fibromatosis_Lab.Invest.2008.PMID.18414401	1.16	0.97	1.38	0.11	0.36
fMaSC.Metab_CellRep.2018.PMID.30089273	1.07	0.91	1.27	0.41	0.72
fMaSC.Metab8_CellRep.2018.PMID.30089273	0.85	0.71	1.02	0.09	0.33
fMaSC.refined1_BCR.2015.PMID.25575446	0.95	0.79	1.13	0.57	0.82
fMasC.Signature_Cell.Stem.Cell.2012.PMID.22305568	0.97	0.82	1.14	0.69	0.88
fMaSC.Signature_CellRep.2018.PMID.30089273	0.93	0.79	1.08	0.34	0.69
FOS.JUN_Cluster_BMC.Med.Genomics.2011.PMID.21214954	1.02	0.87	1.20	0.80	0.94
FOXC1.Hair.Follicles.P30C.LO.vs.WT.Negative_Science.2016.PMID.26912704	1.19	1.00	1.41	0.05	0.22
FOXC1.Hair.Follicles.P30C.LO.vs.WT.Positive_Science.2016.PMID.26912704	1.02	0.86	1.21	0.80	0.94
fSTR.Signature_Cell.Stem.Cell.2012.PMID.22305568	1.07	0.91	1.26	0.43	0.75
Gamma.delta.T.cell_CellRep.2017.PMID.28052254	0.97	0.83	1.15	0.76	0.92
GATA3.induced.genes_JCO.2006.PMID.16505416	1.00	0.85	1.18	0.99	>0.99
GATA3.induced.genes_Oncogene.2004.PMID.15361840	0.86	0.74	1.01	0.06	0.25
GDF11.TGFBR3_Nat.Cell.Biol.2014.PMID.24658685	1.07	0.90	1.26	0.45	0.76
Glycolysis_BMC.Med.2009.PMID.19291283	1.00	0.84	1.20	0.97	>0.99
GO.DOWN.with.SOX10.OE_Cell.Rep.2015.PMID.26365194	1.05	0.88	1.25	0.60	0.84
GO.UP.with.SOX10.OE_Cell.Rep.2015.PMID.26365194	1.01	0.86	1.19	0.91	0.98
GSEA_BIOCARTA_ALK_PATHWAY.PMID.16199517	1.05	0.89	1.24	0.56	0.82
GSEA_BIOCARTA.AKT.PATHWAY.PMID.16199517	0.91	0.77	1.07	0.24	0.58
GSEA_BIOCARTA.BRCA.ATR.PATHWAY.ATRBRCA.PMID.16199517	0.91	0.77	1.08	0.28	0.64
GSEA_BIOCARTA.CASPASE.PATHWAY.PMID.16199517	0.89	0.75	1.05	0.15	0.45
GSEA_BIOCARTA.CTLA4.PATHWAY.PMID.16199517	0.80	0.68	0.95	0.01	0.11
GSEA_BIOCARTA.IGF1R.PATHWAY.PMID.16199517	0.89	0.75	1.05	0.18	0.49
GSEA_BIOCARTA.MTOR.PATHWAY.PMID.16199517	0.98	0.82	1.16	0.77	0.92
GSEA_BIOCARTA.PTEN.PATHWAY.PMID.16199517	0.95	0.81	1.13	0.59	0.84
GSEA_BIOCARTA.RAS.PATHWAY.PMID.16199517	1.00	0.84	1.18	0.97	>0.99
GSEA_BIOCARTA.RB.PATHWAY.PMID.16199517	0.93	0.79	1.09	0.36	0.70
GSEA_BIOCARTA.VEGF.PATHWAY.PMID.16199517	0.99	0.84	1.16	0.86	0.95
GSEA_HALLMARK.MYC.TARGETS.V1.PMID.16199517	1.12	0.95	1.32	0.19	0.51
GSEA_HELLER.HDAC.TARGETS.DOWN.PMID.16199517	0.82	0.69	0.98	0.03	0.15
GSEA_NELSON.RESPONSE.TO.ANDROGEN.UP.PMID.16199517	0.87	0.74	1.03	0.10	0.34
GSEA_REACTOME.PD1.SIGNALING.PMID.16199517	0.81	0.68	0.96	0.01	0.11
GSEA_REACTOME.PI3K.CASCADE.PMID.16199517	0.87	0.73	1.03	0.11	0.37
GSEA_RETINOL.METABOLISM.KEGG.PMID.16199517	0.91	0.77	1.08	0.29	0.65
GSEA.GP1_Proliferation.DNA.repair..PUJANA.CHEK2.PCC.NETWORK.PMID.25109877	0.94	0.80	1.11	0.48	0.77

GSEA.GP1_Proliferation.DNA.repair.REACTOME.CELL.CYCLE.MITOTIC.PMID.25109877	1.00	0.85	1.18	0.98	>0.99
GSEA.GP10_Fatty.acid.oxidation.CARBOXYLIC.ACID.METABOLIC.PROCESS.PMID.25109877	0.87	0.74	1.02	0.09	0.33
GSEA.GP11_Immune.IFN.PerouLab.PMID.25109877	1.02	0.86	1.20	0.86	0.95
GSEA.GP12_Hypoxia.glycolosis.SEMENZA.HIF1.TARGETS.PMID.25109877	0.99	0.84	1.17	0.89	0.97
GSEA.GP13_Neural.signailng.MODULE100.PMID.25109877	1.01	0.85	1.19	0.92	0.99
GSEA.GP13_Neural.signailng.NERVOUS.SYSTEM.DEVELOPMENT.PMID.25109877	1.14	0.96	1.36	0.14	0.43
GSEA.GP14_Plasma.membrane.cell.cell.signaling.MORF.CNTN1.PMID.25109877	0.97	0.82	1.13	0.68	0.87
GSEA.GP15_EGF.signaling.NAGASHIMA.EGF.SIGNALING.UP.PMID.25109877	1.00	0.85	1.17	0.97	>0.99
GSEA.GP16_Protein.kinase.signaling.MAPKs.INTRACELLULAR.SIGNALING.CASCADE.PMID.25109877	0.85	0.71	1.00	0.05	0.22
GSEA.GP16_Protein.kinase.signaling.MAPKs.REGULATION.OF.KINASE.ACTIVITY.PMID.25109877	1.02	0.86	1.21	0.85	0.95
GSEA.GP17_Basal.signaling.SMID.BREAST.CANCER.BASAL.UP.PMID.25109877	0.99	0.82	1.21	0.94	>0.99
GSEA.GP18_Vesicle.EPR.MEMBRANE.COAT.PMID.25109877	0.94	0.79	1.12	0.51	0.79
GSEA.GP19_1Q.amplicon.PerouLab.PMID.25109877	0.99	0.84	1.17	0.93	0.99
GSEA.GP2_Immune.Tcell.Bcell.KEGG.HEMATOPOIETIC.CELL.LINEAGE.PMID.25109877	0.79	0.66	0.94	0.007	0.10
GSEA.GP2_Immune.Tcell.Bcell.PerouLab.PMID.25109877	0.83	0.70	0.99	0.04	0.18
GSEA.GP20_TAL1.Leukemia.erythropoiesis.GNF2.TAL1.PMID.25109877	0.92	0.78	1.09	0.33	0.68
GSEA.GP21_Anti.apoptosis.DNA.stability.MORF.BCL2.PMID.25109877	0.96	0.81	1.14	0.65	0.86
GSEA.GP21_Anti.apoptosis.DNA.stability.MORF.MT4.PMID.25109877	0.93	0.79	1.10	0.38	0.71
GSEA.GP21_Anti.apoptosis.DNA.stability.MORF.STK17A.PMID.25109877	0.91	0.77	1.08	0.27	0.62
GSEA.GP22_16Q22.24.amplicon.PerouLab.PMID.25109877	1.04	0.89	1.22	0.62	0.85
GSEA.GP3_Tumo.suppressing.miRNA.targets.GTTTGTT.MIR.495.PMID.25109877	0.97	0.82	1.14	0.68	0.87
GSEA.GP3_Tumor.suppressing.miRNA.targets.DACOSTA.UV.RESPONSE.VIA.ERCC3.DN.PMID.25109877	0.97	0.83	1.14	0.71	0.89
GSEA.GP3_Tumor.suppressing.miRNA.targets.TGCTTTG.MIR.330.PMID.25109877	0.98	0.83	1.15	0.81	0.94
GSEA.GP4_MES.ECM.PerouLab.PMID.25109877	1.11	0.94	1.32	0.22	0.57
GSEA.GP5_MYC.targets.TERT.PerouLab.PMID.25109877	0.93	0.79	1.10	0.41	0.72
GSEA.GP6_Squamous.differentiation.development.RICKMAN.TUMOR.DIFFERENTIATED.WELL.VS.POORLY.DN.PMID.25109877	0.92	0.77	1.09	0.35	0.70
GSEA.GP7_Estrogen.signaling.SMID.BREAST.CANCER.BASAL.DN.PMID.25109877	1.03	0.84	1.25	0.80	0.94
GSEA.GP8_FOXO.stemness.MORF.PTPRB.PMID.25109877	1.02	0.87	1.20	0.81	0.94
GSEA.GP8_FOXO.stemness.TTGTTT.VSFOXO4.01.PMID.25109877	1.02	0.87	1.19	0.84	0.95
GSEA.GP9_Cell.cell.adhesion.PerouLab.PMID.25109877	1.05	0.88	1.25	0.56	0.82
HCK_BCR.2008.PMID.19272155	0.85	0.71	1.00	0.06	0.24
HER1.Cluster1_BMC.Genomics.2007.PMID.17663798	1.04	0.88	1.23	0.61	0.85
HER1.Cluster2_BMC.Genomics.2007.PMID.17663798	0.93	0.78	1.10	0.38	0.71
HER1.Cluster3_BMC.Genomics.2007.PMID.17663798	0.93	0.80	1.09	0.40	0.71
HER2.Amplicon.PerouLab_BMC.Med.Genomic.2011.PMID.21214954	0.93	0.79	1.10	0.39	0.71
Histological.Grade_J.Pathol.2017.PMID.27861902	1.00	0.84	1.18	0.97	>0.99
HouseKeeping_Genome.Biol.2004.PMID.15287981	1.00	0.85	1.19	0.97	>0.99
iDC.Median_Immunity.2013.PMID.24138885	0.95	0.80	1.12	0.55	0.82
IFN.Cluster_BMC.Med.Genomics.2011.PMID.21214954	1.15	0.97	1.35	0.11	0.36
IgG_BCR.2008.PMID.19272155	0.74	0.63	0.87	<0.001	0.03
IGG.Cluster_BMC.Med.Genomics.2011.PMID.21214954	0.73	0.62	0.87	<0.001	0.03
Immature..B.cell_CellRep.2017.PMID.28052254	0.80	0.67	0.96	0.01	0.11
Immature.dendritic.cell_CellRep.2017.PMID.28052254	1.07	0.91	1.27	0.40	0.71
ImmLandscape_Macro.mono.CSF1.core.response_CCR.2009.PMID.29628290	0.80	0.68	0.96	0.01	0.11
ImmLandscape_Wound.Healing_Immunity.2018.PMID.29628290	0.92	0.78	1.08	0.31	0.67
ImmLandscape.IFN3_Plos.One.2014.PMID.24516633	1.14	0.97	1.35	0.11	0.37
ImmLandscape.IFNG5_Plos.One.2014.PMID.24516633	0.77	0.65	0.91	0.003	0.08
ImmLandscape.lymphocyte.Infil.T.B.PMID.18592372	0.80	0.68	0.95	0.009	0.10

Immune.Hot.CD8.vs.Cold_Nature.2020.PMID.31942071	0.80	0.68	0.95	0.01	0.11
Immune.Perez.14_JCO.2015.PMID.25605861	0.78	0.66	0.92	0.004	0.08
Immune.Perez.87_JCO.2015.PMID.25605861	0.78	0.66	0.93	0.004	0.09
Immune.Suppression_JCI.Insight.2016.PMID.27699256	0.83	0.70	0.98	0.03	0.15
ImmuneActive_Cell.2019.PMID.31730857	0.77	0.65	0.92	0.003	0.08
Immunosuppression.PMID.31942077	1.15	0.97	1.36	0.10	0.36
IMS.Score_CCR.2018.PMID.29921729	0.98	0.83	1.16	0.84	0.95
Induced.in.Bcells_PNAS.2013.PMID.23382184	0.84	0.71	1.00	0.05	0.21
Induced.in.DC_PNAS.2013.PMID.23382184	0.80	0.68	0.95	0.01	0.11
Induced.in.GN_PNAS.2013.PMID.23382184	0.91	0.77	1.08	0.27	0.62
Induced.in.HSC_PNAS.2013.PMID.23382184	1.03	0.87	1.21	0.76	0.92
Induced.in.MOs_PNAS.2013.PMID.23382184	0.93	0.79	1.10	0.41	0.72
Induced.in.NKcells_PNAS.2013.PMID.23382184	1.00	0.84	1.19	0.99	>0.99
Induced.in.Tcells_PNAS.2013.PMID.23382184	0.92	0.78	1.08	0.31	0.66
Inflammatory.breast.cancer.491genes_CCR.2013.PMID.23396049	0.84	0.71	1.00	0.05	0.23
Inflammatory.breast.cancer.79genes_CCR.2013.PMID.23396049	1.00	0.84	1.19	1.00	>0.99
Inflammatory.breast.cancer.expressed.noIBC_79genes_CCR.2013.PMID.23396049	0.91	0.77	1.07	0.24	0.58
Inflammatory.breast.cancer.expressed.noIBC.491genes_CCR.2013.PMID.23396049	0.99	0.83	1.19	0.94	>0.99
Influenza.11genes.Metasignature_Immunity.2015.PMID.26682989	1.04	0.88	1.23	0.62	0.85
Interferon_BCR.2008.PMID.19272155	1.20	1.02	1.41	0.03	0.15
Interferon.Pathway_CancerImmunolRes.2018.PMID.30266715	1.11	0.94	1.30	0.23	0.57
JUND.KRT5_Nat.Cell.Biol.2014.PMID.24658685	1.12	0.95	1.32	0.16	0.47
Keller2012.CD10.Adam_BCR.2015.PMID.25575446	1.06	0.90	1.26	0.47	0.77
KRAS.amplicon_Genome.Biology.2007.PMID.17493263	1.00	0.85	1.18	0.98	>0.99
Late.IRS.1_PLoS.One.2016.PMID.26991655	1.05	0.89	1.24	0.58	0.83
Late.IRS.2_PLoS.One.2016.PMID.26991655	1.03	0.88	1.22	0.69	0.88
LCK_BCR.2008.PMID.19272155	0.79	0.67	0.94	0.009	0.10
Lim2009.LumProg.Adam_BCR.2015.PMID.25575446	0.93	0.77	1.13	0.49	0.77
Lim2009.MaSC.Adam_BCR.2015.PMID.25575446	1.03	0.87	1.22	0.74	0.90
Lim2009.MatureLum.Adam_BCR.2015.PMID.25575446	1.08	0.88	1.31	0.47	0.77
Lim2009.Stroma.Adam_BCR.2015.PMID.25575446	1.08	0.91	1.28	0.36	0.70
Lim2010.LumProg.Adam_BCR.2015.PMID.25575446	0.89	0.76	1.05	0.18	0.50
Lim2010.MaSC.Adam_BCR.2015.PMID.25575446	1.09	0.91	1.29	0.36	0.70
Lim2010.MatureLum.Adam_BCR.2015.PMID.25575446	1.03	0.88	1.22	0.68	0.87
Lim2010.Stroma.Adam_BCR.2015.PMID.25575446	1.03	0.87	1.22	0.70	0.89
Lobular.Carcinoma.In.Situ_J.Pathol.2017.PMID.27861902	1.17	0.99	1.37	0.07	0.27
LOBULAR.TCGA.SIGNATURE.ImmuneCell.2015.PMID.26451490	0.81	0.68	0.96	0.01	0.11
LOBULAR.TCGA.SIGNATURE.Reactive_Cell.2015.PMID.26451490	1.07	0.90	1.27	0.43	0.74
LOBULAR.TCGA.SUBTYPE.Immune_Cell.2015.PMID.26451490	0.93	0.78	1.10	0.38	0.71
LOBULAR.TCGA.SUBTYPE.Proliferative_Cell.2015.PMID.26451490	1.02	0.86	1.20	0.84	0.95
LOBULAR.TCGA.SUBTYPE.Reactive_Cell.2015.PMID.26451490	1.04	0.88	1.23	0.66	0.86
LTS.score_JCI.2020.PMID.32573490	1.09	0.92	1.28	0.31	0.66
Luminal_Progenitor_Up_Nat.Med.2009.PMID.19648928	0.90	0.74	1.09	0.26	0.61
Luminal.cluster_BMC.Med.Genomics.2011.PMID.21214954	1.01	0.83	1.24	0.89	0.97
Luminal.Progenitor_BCR.2010.PMID.20346151	0.86	0.71	1.03	0.10	0.34
Luminal.Progenitor.Down_Nat.Med.2009.PMID.19648928	1.17	0.98	1.40	0.08	0.30
LumProg.HsEnriched_BCR.2015.PMID.25575446	0.89	0.74	1.08	0.24	0.58
LumProg.HsEnriched.Refined1_BCR.2015.PMID.25575446	0.89	0.74	1.07	0.21	0.54

LumProg.Lim09_BCR.2015.PMID.25575446	0.89	0.74	1.06	0.19	0.51
LumProg.Prat_BCR.2015.PMID.25575446	0.91	0.77	1.08	0.29	0.65
LumProg.Shehata_BCR.2015.PMID.25575446	0.91	0.77	1.06	0.23	0.57
Lums.HER2E.DOWN.metastatic.signature_JCI.2020.PMID.32573490	1.24	1.04	1.49	0.02	0.11
Lums.HER2E.UP.metastatic.signature_JCI.2020.PMID.32573490	0.92	0.78	1.08	0.30	0.66
Lung.WNT_Cancer.Res.2009.PMID.19549913	1.04	0.88	1.23	0.65	0.86
Lymph.vessels_Immunity.2013.PMID.24138885	1.04	0.88	1.24	0.64	0.85
Lymphovascular.Invasion_J.Pathol.2017.PMID.27861902	1.02	0.86	1.20	0.84	0.95
M.D.Metagene_Genome.Biol.2013.PMID.23618380	0.81	0.68	0.96	0.02	0.12
M2.Macrophage_Blood.2006.PMID.16556895	0.85	0.72	1.01	0.06	0.25
Macrophage_CellRep.2017.PMID.28052254	0.88	0.75	1.05	0.15	0.44
Macrophages_CancerImmunolRes.2018.PMID.30266715	0.82	0.69	0.97	0.02	0.12
Macrophages_Immunity.2013.PMID.24138885	0.87	0.73	1.04	0.13	0.42
Macrophages.M0_Nat.Methods.2015.PMID.25822800	0.82	0.69	0.98	0.03	0.15
Macrophages.M1_Nat.Methods.2015.PMID.25822800	0.83	0.70	0.99	0.03	0.17
Macrophages.M2_Nat.Methods.2015.PMID.25822800	0.85	0.71	1.01	0.06	0.26
MacTh1.cluster_CCR.2014.PMID.24916698	0.81	0.68	0.96	0.02	0.12
MammaPrint_Nature.2002.PMID.11823860	1.04	0.87	1.25	0.66	0.86
MAPK.pathway.activation_NPJ.Precis.Oncol.2018.PMID.29872725	1.19	1.00	1.41	0.04	0.20
MASC.Down_Nat.Med.2009.PMID.19648928	0.97	0.82	1.16	0.77	0.92
MASC.Up_Nat.Med.2009.PMID.19648928	1.14	0.95	1.35	0.15	0.44
Mast.cell_CellRep.2017.PMID.28052254	0.84	0.71	1.00	0.05	0.22
Mast.cells_Immunity.2013.PMID.24138885	1.01	0.85	1.19	0.94	>0.99
Mast.cells.activated_Nat.Methods.2015.PMID.25822800	0.92	0.78	1.09	0.35	0.70
Mast.cells.resting_Nat.Methods.2015.PMID.25822800	0.90	0.76	1.06	0.21	0.54
Mature.luminal_BCR.2010.PMID.20346151	1.00	0.83	1.20	0.99	>0.99
Mature.Luminal.Down_Nat.Med.2009.PMID.19648928	1.05	0.87	1.26	0.61	0.85
Mature.LuminaUp_Nat.Med.2009.PMID.19648928	1.00	0.82	1.21	0.99	>0.99
MatureLum.HsEnriched_BCR.2015.PMID.25575446	0.91	0.75	1.11	0.36	0.70
MatureLum.HsEnriched.Refined1_BCR.2015.PMID.25575446	0.93	0.77	1.12	0.45	0.76
MatureLum.Lim09_BCR.2015.PMID.25575446	0.96	0.79	1.15	0.64	0.86
MatureLum.Prat_BCR.2015.PMID.25575446	0.94	0.79	1.12	0.52	0.80
MatureLum.Shehata_BCR.2015.PMID.25575446	1.01	0.84	1.21	0.94	>0.99
MBasal.Cluster_BMC.Med.Genomics.2011.PMID.21214954	1.15	0.97	1.36	0.12	0.38
MCD3.CD8_BMC.Med.Genomics.2011.PMID.21214954	0.81	0.68	0.96	0.02	0.12
MCF7.E2.induced.genes_JCO.2006.PMID.16505416	0.89	0.75	1.04	0.15	0.44
MCF7.E2.repressed.genes_JCO.2006.PMID.16505416	0.93	0.77	1.11	0.40	0.71
MDSC_CellRep.2017.PMID.28052254	0.78	0.66	0.93	0.005	0.09
MDSC.Granulocytic_Leukoc.Biol.2012.PMID.21954284	0.94	0.80	1.11	0.46	0.76
MDSC.Neutrophi_Leukoc.Biol.2012.PMID.21954284	0.82	0.70	0.97	0.02	0.13
MDSC.tumor_J.Immunol.2012.PMID.23152559	0.88	0.74	1.05	0.16	0.47
MDSC.tumor.MO_J.Immunol.2012.PMID.23152559	0.90	0.76	1.07	0.24	0.58
MECM_BMC.Med.Genomics.2011.PMID.21214954	1.12	0.94	1.32	0.21	0.55
Memory.B.cell_CellRep.2017.PMID.28052254	1.11	0.94	1.32	0.22	0.57
MET.DOWN.RNAseq.Significant.Genes_JCI.2018.PMID.29480819	0.92	0.79	1.07	0.28	0.64
MET.DOWN.Significant.Genes.Low.Basal.1_JCI.2018.PMID.29480819	1.14	0.96	1.36	0.14	0.43
MET.DOWN.Significant.Genes.Low.Basal.2_JCI.2018.PMID.29480819	0.98	0.83	1.16	0.82	0.94
MET.UP.RNAseq.Significant.Genes_JCI.2018.PMID.29480819	1.16	0.98	1.37	0.08	0.31

MET.UP.Significant.Genes.HIGH.BASALS.Genes_JCI.2018.PMID.29480819	0.95	0.80	1.13	0.56	0.82
Metaplastic.Up_CanRes.2009.PMID.19435916	1.07	0.89	1.27	0.48	0.77
Metastasis.predictor.TNBC_BCR.2010.PMID.20946665	0.80	0.68	0.94	0.007	0.10
MFGFR2_BMC.Med.Genomics.2011.PMID.21214954	1.08	0.90	1.29	0.40	0.71
MHC.Forero.11_Cancer.Immunol.Res.2016.PMID.26980599	0.82	0.68	0.97	0.03	0.14
MHC.Forero.24_Cancer.Immunol.Res.2016.PMID.26980599	0.91	0.77	1.07	0.25	0.59
MHC.I_BCR.2008.PMID.19272155	0.98	0.83	1.16	0.83	0.95
MHC.II_BCR.2008.PMID.19272155	0.77	0.65	0.92	0.003	0.08
MHCI.coreGenes_Nat.Commun.2017.PMID29170503	0.94	0.80	1.11	0.50	0.78
MIR200c.Induced_ONCO.2015.PMID.25746005	0.98	0.83	1.15	0.78	0.93
MIR200c.Repressed_ONCO.2015.PMID.25746005	1.10	0.92	1.32	0.29	0.65
miRNA.138.signature_Cancer.Res.2014.PMID.25339353	0.92	0.78	1.09	0.32	0.67
MITO1_BMC.Med.Genomics.2011.PMID.21214954	0.99	0.85	1.16	0.92	0.99
MITO2_BMC.Med.Genomics.2011.PMID.21214954	0.94	0.80	1.10	0.45	0.76
Mitotic.Count_J.Pathol.2017.PMID.27861902	1.06	0.90	1.25	0.49	0.77
MK14.K17_BMC.Med.Genomics.2011.PMID.21214954	1.10	0.93	1.30	0.27	0.62
MKRAS.amplicon_BMC.Med.Genomics.2011.PMID.21214954	0.91	0.77	1.07	0.26	0.61
MM.BRCAwnt.1pFDR.UP_Genome.Biology.2007.PMID.17493263	1.08	0.91	1.28	0.35	0.70
MM.C3Tag.1pFDR.UP_Genome.Biology.2007.PMID.17493263	1.02	0.86	1.21	0.83	0.95
MM.C3Tag.2012_Genome.Biol.2013.PMID.24220145	0.99	0.84	1.18	0.94	>0.99
MM.Class3_Genome.Biol.2013.PMID.24220145	0.98	0.83	1.16	0.82	0.94
MM.Class8_Genome.Biol.2013.PMID.24220145	1.16	0.98	1.38	0.09	0.33
MM.Claudinlow_Genome.Biol.2013.PMID.24220145	1.00	0.84	1.18	0.97	>0.99
MM.DMBAwnt.1pFDR.UP.Genome.Biology.2007.PMID.17493263	1.11	0.93	1.32	0.24	0.59
MM.Erbb2.like_Genome.Biol.2013.PMID.24220145	0.98	0.83	1.15	0.78	0.93
MM.Myc.2012_Genome.Biol.2013.PMID.24220145	0.91	0.77	1.07	0.25	0.60
MM.Myoepithelioma.like_Genome.Biol.2013.PMID.24220145	1.08	0.91	1.28	0.37	0.70
MM.Neu.2012_Genome.Biol.2013.PMID.24220145	1.03	0.87	1.21	0.76	0.92
MM.NeuPyMT.1pFDR.UP_Genome.Biology.2007.PMID.17493263	0.92	0.79	1.08	0.30	0.66
MM.Normal.1pFDR.UP_Genome.Biology.2007.PMID.17493263	0.94	0.79	1.11	0.45	0.76
MM.Normal.like_Genome.Biol.2013.PMID.24220145	0.93	0.78	1.10	0.40	0.71
MM.p53null.1pFDR.UP_Genome.Biology.2007.PMID.17493263	0.96	0.81	1.14	0.65	0.86
MM.p53null.Basal_Genome.Biol.2013.PMID.24220145	1.18	0.99	1.41	0.07	0.27
MM.p53null.Luminal_Genome.Biol.2013.PMID.24220145	0.97	0.82	1.14	0.68	0.87
MM.Potluck_1pFDR_UP_Genome.Biology.2007_PMID.17493263.PMID.24220145	0.94	0.79	1.12	0.49	0.78
MM.PyMT.2012_Genome.Biol.2013.PMID.24220145	0.96	0.82	1.13	0.63	0.85
MM.Squamous.like_Genome.Biol.2013.PMID.24220145	1.05	0.89	1.25	0.56	0.82
MM.Stat1_Genome.Biol.2013.PMID.24220145	0.92	0.79	1.09	0.34	0.70
MM.WapINT3.1pFDR.UP_Genome.Biology.2007.PMID.17493263	1.03	0.87	1.22	0.77	0.92
MM.WapINT3.2012_Genome.Biol.2013.PMID.24220145	1.14	0.96	1.35	0.14	0.43
MM.WAPTag.1pFDR.UP_Genome.Biology.2007.PMID.17493263	1.02	0.86	1.20	0.85	0.95
MM.Wnt1.Early_Genome.Biol.2013.PMID.24220145	1.09	0.91	1.30	0.34	0.69
MM.Wnt1.Late_Genome.Biol.2013.PMID.24220145	1.13	0.95	1.34	0.17	0.48
Mmyosin_BMC.Med.Genomics.2011.PMID.21214954	1.07	0.90	1.27	0.43	0.75
MNADH_CYTochrome_BMC.Med.Genomics.2011.PMID.21214954	0.84	0.72	0.98	0.03	0.15
MNB1_BMC.Med.Genomics.2011.PMID.21214954	0.83	0.70	0.99	0.03	0.17
MNB2_BMC.Med.Genomics.2011.PMID.21214954	0.87	0.73	1.05	0.14	0.43
MNB3_BMC.Med.Genomics.2011.PMID.21214954	0.95	0.80	1.13	0.56	0.82

MNOtch4_BMC.Med.Genomics.2011.PMID.21214954	1.21	1.02	1.44	0.03	0.15
Monocyte_CellRep.2017.PMID.28052254	0.81	0.68	0.96	0.02	0.11
Monocyte..DC.25gene_Genome.Biol.2013.PMID.23618380	0.80	0.67	0.94	0.009	0.10
Monocytes_CancerImmunolRes.2018.PMID.30266715	0.83	0.70	0.98	0.03	0.15
Monocytes_Nat.Methods.2015.PMID.25822800	0.81	0.68	0.96	0.01	0.11
Monocytic.lineage.MCP_Nature.2020.PMID.31942075	0.83	0.70	0.99	0.04	0.18
MProliferation_BMC.Med.Genomics.2011.PMID.21214954	0.95	0.80	1.12	0.51	0.79
MProtocadherin_BMC.Med.Genomics.2011.PMID.21214954	0.88	0.74	1.05	0.15	0.45
MPYMT_NEU_Cluster_BMC.Med.Genomics.2011.PMID.21214954	0.88	0.75	1.03	0.11	0.37
MRibosomal_BMC.Med.Genomics.2011.PMID.21214954	1.11	0.95	1.31	0.19	0.51
MS.CD44.DOWN_PNAS.2009.PMID.19666588	1.05	0.88	1.24	0.61	0.85
MS.CD44.UP_PNAS.2009.PMID.19666588	1.00	0.85	1.19	0.96	>0.99
MSquamous_BMC.Med.Genomics.2011.PMID.21214954	1.07	0.91	1.27	0.40	0.71
Murat.G07_JCO.2008.PMID.18565887	1.21	1.02	1.45	0.03	0.16
Murat.G18_JCO.2008.PMID.18565887	1.00	0.85	1.19	0.97	>0.99
Murat.G24_JCO.2008.PMID.18565887	0.77	0.65	0.92	0.004	0.08
MVEGFC_BMC.Med.Genomics.2011.PMID.21214954	1.08	0.91	1.28	0.39	0.71
Myeloid.cell.chemotaxis.1gene_Nature.2020.PMID.31942077	0.97	0.82	1.15	0.72	0.90
Myeloid.dendritic.cells.MCP_Nature.2020.PMID.31942077	0.78	0.66	0.91	0.002	0.08
Natural.killer.cell_CellRep.2017.PMID.28052254	0.94	0.80	1.11	0.45	0.76
Natural.killer.T.cell_CellRep.2017.PMID.28052254	0.91	0.76	1.07	0.25	0.60
Necrosis_J.Pathol.2017.PMID.27861902	0.99	0.84	1.17	0.90	0.97
Neutrophil_CellRep.2017.PMID.28052254	0.94	0.80	1.11	0.48	0.77
Neutrophils_CancerImmunolRes.2018.PMID.30266715	0.96	0.80	1.14	0.63	0.85
Neutrophils_Immunity.2013.PMID.24138885	0.89	0.75	1.06	0.19	0.51
Neutrophils_Nat.Methods.2015.PMID.25822800	0.80	0.67	0.95	0.01	0.11
Neutrophils.MCP_Nature.2020.PMID.31942077	1.01	0.85	1.19	0.92	0.99
NK_Immunity.2013.PMID.24138885	1.01	0.85	1.19	0.94	>0.99
NK.activated_Nat.Methods.2015.PMID.25822800	0.79	0.67	0.94	0.008	0.10
NK.CD56bright_Immunity.2013.PMID.24138885	0.95	0.80	1.12	0.51	0.79
NK.CD56dim_Immunity.2013.PMID.24138885	0.81	0.69	0.96	0.02	0.11
NK.resting_Nat.Methods.2015.PMID.25822800	0.80	0.68	0.95	0.009	0.10
NKcells_CancerImmunolRes.2018.PMID.30266715	0.75	0.63	0.88	0.001	0.05
NKcells.MCP_Nature.2020.PMID.31942077	0.82	0.69	0.97	0.02	0.13
No.Response.Immunotherapy.TLS.Melanoma_Nature.2020.PMID.31942075	1.20	1.02	1.42	0.03	0.16
Normal.mucosa_Immunity.2013.PMID.24138885	1.09	0.92	1.28	0.32	0.68
Nuclear.Pleomorphism_J.Pathol.2017.PMID.27861902	0.94	0.80	1.11	0.45	0.76
Oncotype_NEJM.2004.PMID.15591335	1.03	0.86	1.22	0.78	0.93
P53.ERPos.MDACC_CCR.2011.PMID.21248301	0.98	0.83	1.16	0.81	0.94
Parity.signature.251genes_BCR.2014.PMID.25005139	0.81	0.68	0.96	0.01	0.11
Parity.signature.40genes_BCR.2014.PMID.25005139	0.82	0.69	0.97	0.02	0.13
PARPi.Resistance_BCRT_2012.PMID.22875744	0.92	0.78	1.08	0.31	0.66
PARPi.Sensitivity_BCRT_2012.PMID.22875744	0.92	0.77	1.09	0.35	0.70
PARPi.Sensitivity.MDACC_NPJ.Syst.Biol.Appl.2017.PMID.28649435	0.88	0.74	1.04	0.14	0.43
PARPi.Sensitivity.Negative_Sci.Adv.2017.PMID.28439535	1.02	0.87	1.21	0.78	0.93
PARPi.Sensitivity.Positive_Sci.Adv.2017.PMID.28439535	0.93	0.78	1.11	0.42	0.74
Pcorr.Breast2Lung.LM2.Correlation_Nature.2005.PMID.16049480	1.06	0.89	1.26	0.49	0.78
Pcorr.Breast2Lung.Parental.Correlation_Nature.2005.PMID.16049480	0.94	0.79	1.12	0.51	0.79

Pcorr.dasatinib.resistant_Cancer.Res.2007.PMID.17332353	1.07	0.89	1.28	0.46	0.76
Pcorr.dasatinib.sensitive_Cancer.Res.2007.PMID.17332353	0.94	0.79	1.13	0.54	0.81
Pcorr.Hypoxia.High.Correlation_PLoS.Med.2006.PMID.16417408	0.95	0.80	1.12	0.55	0.81
Pcorr.Hypoxia.Low.Correlation_PLoS.Med.2006.PMID.16417408	1.07	0.90	1.27	0.42	0.74
Pcorr.IGS_Invasiveness_NJEM.2007.PMID.17229949	0.96	0.82	1.13	0.65	0.86
Pcorr.wound.response.activated_PNAS.2005.PMID.15701700	1.01	0.86	1.19	0.90	0.97
pCR.predictor.ERNeg.55genes_JAMA.2011.PMID.21558518	1.03	0.88	1.21	0.71	0.89
pCR.predictor.ERPos.39genes_JAMA.2011.PMID.21558518	0.98	0.83	1.15	0.77	0.92
PDCD1_Single_Gene.Single	0.82	0.70	0.96	0.01	0.11
Pfefferle2012.LumProg_BCR.2015.PMID.25575446	0.84	0.70	1.01	0.07	0.27
Pfefferle2012.MaSC_BCR.2015.PMID.25575446	1.08	0.91	1.28	0.37	0.70
Pfefferle2012.MatureLum_BCR.2015.PMID.25575446	1.03	0.85	1.24	0.79	0.93
Pfefferle2012.Stroma_BCR.2015.PMID.25575446	1.08	0.91	1.28	0.38	0.71
PGR_Single_Gene.Single	1.00	0.82	1.22	>0.99	>0.99
PI3Ki.Down_CancerCell.2017.PMID.28528867	0.89	0.76	1.04	0.16	0.46
PI3Ki.Up_CancerCell.2017.PMID.28528867	0.96	0.81	1.14	0.67	0.87
PIK3CA.Pathway_Ann.Oncol.2017.PMID.28177460	0.92	0.78	1.08	0.31	0.66
PIK3CAmt.signature_Cancer.Res.2012.PMID.22552288	1.00	0.84	1.18	0.99	>0.99
Plasma.cells_Nat.Methods.2015.PMID.25822800	0.77	0.65	0.92	0.004	0.08
PlasmaCells_CancerImmunolRes.2018.PMID.30266715	0.76	0.65	0.89	0.001	0.05
Plasmacytoid.dendritic.cell_CellRep.2017.PMID.28052254	0.90	0.77	1.06	0.22	0.57
PR.Isoform.Ratio.Up.in.PRA.H_JNCI.2017.PMID.28376177	0.95	0.80	1.13	0.57	0.82
PR.Isoform.Ratio.Up.in.PRB.H_JNCI.2017.PMID.28376177	1.02	0.86	1.21	0.81	0.94
Proliferation.Cluster_BMC.Med.Genomics.2011.PMID.21214954	0.98	0.83	1.16	0.82	0.94
Proliferation.Metagene_Genome.Biol.2013.PMID.23618380	0.96	0.81	1.14	0.65	0.86
Proliferation.score.PAM50_JCO.2009.PMID.19204204	0.98	0.83	1.17	0.85	0.95
ProliferationPathway_CancerImmunolRes.2018.PMID.30266715	0.96	0.81	1.13	0.60	0.84
Prosigna.Proliferation.18_BMC.Med.Genomics.2015.PMID.26297356	0.98	0.83	1.16	0.84	0.95
Race.LuminalA.MRE.score_BCRT.2015.PMID.26109344	1.07	0.90	1.26	0.46	0.76
Radiation.induced.genes_Radoat.Res.2014.PMID.24527691	1.09	0.90	1.32	0.36	0.70
RB.LOH_BCR.2008.PMID.18782450	0.97	0.82	1.14	0.69	0.88
RB.LOSS_JCI.2007.PMID.17160137	0.96	0.81	1.13	0.62	0.85
Regulatory.T.cell_CellRep.2017.PMID.28052254	0.85	0.71	1.01	0.06	0.25
Replication.Stress.Down.set_Cell.Rep.2018.PMID.29768207	0.93	0.79	1.09	0.37	0.71
Replication.Stress.Model_Cell.Rep.2018_PMID.29768207.PMID.29768207	1.09	0.92	1.29	0.34	0.69
Replication.Stress.Neg_Cell.Rep.2018_PMID.29768207.PMID.29768207	0.92	0.78	1.08	0.29	0.65
Replication.Stress.Pos_Cell.Rep.2018_PMID.29768207.PMID.29768207	1.12	0.94	1.32	0.19	0.52
Replication.Stress.Up_Set_Cell.Rep.2018_PMID.29768207.PMID.29768207	1.11	0.93	1.31	0.24	0.58
Residual.disease.predictor.ERNeg.54genes_JAMA.2011.PMID.21558518	1.03	0.87	1.21	0.74	0.90
Residual.disease.predictor.ERPos.73genes_JAMA.2011.PMID.21558518	0.91	0.77	1.07	0.26	0.61
Response.Immunotherapy.MCP.TLS.Melanoma_Nature.2020.PMID.31942075	0.72	0.60	0.86	<0.001	0.03
Response.Immunotherapy.signature_Science.2018.PMID.30309915	0.80	0.68	0.95	0.009	0.10
Response.Neo.Chemo_common_CCR.2014.PMID.25047707	0.95	0.80	1.12	0.51	0.79
Response.Neo.Chemo_ERNeg_CCR.2014.PMID.25047707	1.00	0.85	1.18	0.97	>0.99
Response.Neo.Chemo_ERPos_CCR.2014.PMID.25047707	1.04	0.88	1.23	0.66	0.86
RHOA.pathway_Ann.Oncol.2017.PMID.28177460	1.16	0.98	1.38	0.08	0.30
Ribosomal.Cluster_BMC.Med.Genomics.2011.PMID.21214954	1.07	0.91	1.26	0.40	0.71
ROR.subtype.PAM50_JCO.2009.PMID.19204204	0.95	0.78	1.15	0.60	0.84

ROR.subtype.proliferation.PAM50_JCO.2009.PMID.19204204	0.97	0.81	1.16	0.73	0.90
RSS.Score_CCR.2018.PMID.29921729	0.98	0.83	1.15	0.81	0.94
S100A9.A8_BMC.Med.Genomics.2011.PMID.21214954	0.92	0.76	1.11	0.38	0.71
Scorr.EMAT1.Correlation_BCR.2020.PMID.32641077	0.92	0.77	1.09	0.32	0.68
Scorr.EMAT2.Correlation_BCR.2020.PMID.32641077	1.25	1.06	1.48	0.010	0.10
Scorr.EMAT3.Correlation_BCR.2020.PMID.32641077	0.99	0.83	1.18	0.93	0.99
Scorr.EMAT4.Correlation_BCR.2020.PMID.32641077	0.88	0.72	1.07	0.19	0.51
Scorr.IE.Correlation_JCO.2006.PMID.16505416	1.05	0.88	1.24	0.60	0.84
Scorr.IIE.Correlation_JCO.2006.PMID.16505416	0.94	0.79	1.11	0.45	0.76
Scorr.PAM50.Basal_JCO.2009.PMID.19204204	1.07	0.87	1.33	0.52	0.79
Scorr.PAM50.Her2_JCO.2009.PMID.19204204	0.93	0.77	1.12	0.43	0.74
Scorr.PAM50.LumA_JCO.2009.PMID.19204204	1.02	0.83	1.26	0.84	0.95
Scorr.PAM50.LumB_JCO.2009.PMID.19204204	0.92	0.78	1.09	0.34	0.69
Scorr.PAM50.Normal_JCO.2009.PMID.19204204	1.07	0.91	1.27	0.40	0.71
Scorr.S329.L_Br.J.Cancer.2008.PMID.18382427	0.99	0.83	1.18	0.90	0.97
Scorr.S329.R_Br.J.Cancer.2008.PMID.18382427	0.96	0.80	1.15	0.66	0.86
Secretoglobin_BMC.Med.Genomics.2011.PMID.21214954	0.95	0.80	1.12	0.55	0.81
Shehata2012.ALDHneg_BCR.2015.PMID.25575446	0.97	0.82	1.15	0.72	0.90
Shehata2012.ALDHpos_BCR.2015.PMID.25575446	0.80	0.66	0.97	0.02	0.13
Shehata2012.Basal_BCR.2015.PMID.25575446	1.06	0.89	1.25	0.53	0.80
Shehata2012.Erbb3neg_BCR.2015.PMID.25575446	0.92	0.78	1.08	0.30	0.66
Shehata2012.LumProg_BCR.2015.PMID.25575446	0.92	0.77	1.09	0.34	0.69
Shehata2012.NCL_BCR.2015.PMID.25575446	1.00	0.83	1.20	>0.99	>0.99
Shehata2012.Stroma_BCR.2015.PMID.25575446	1.06	0.89	1.25	0.53	0.80
Spike2012.aMaSC_BCR.2015.PMID.25575446	1.12	0.95	1.33	0.17	0.48
Spike2012.fMaSC_BCR.2015.PMID.25575446	0.97	0.81	1.15	0.70	0.89
Spike2012.fStr_BCR.2015.PMID.25575446	0.97	0.82	1.15	0.73	0.90
STAT1_BCR.2008.PMID.19272155	0.88	0.74	1.04	0.13	0.40
STAT3.Basal_PNAS.2014.PMID.25139989	0.82	0.69	0.97	0.02	0.13
STAT3.Basal.short_PNAS.2014.PMID.25139989	0.83	0.70	0.98	0.03	0.15
Stroma.FNA.MDACC.1_JCO.2010.PMID.20805453	0.82	0.69	0.97	0.02	0.13
Stroma.FNA.MDACC.2_JCO.2010.PMID.20805453	1.12	0.94	1.33	0.21	0.54
Stromal.Central.Fibrotic.Focus_J.Pathol.2017.PMID.27861902	0.89	0.75	1.06	0.20	0.52
Stromal.Down_Nat.Med.2009.PMID.19648928	1.01	0.85	1.19	0.90	0.98
Stromal.Inflammation_J.Pathol.2017.PMID.27861902	0.77	0.65	0.92	0.004	0.08
Stromal.Signature_Nat.Med.2008.PMID.18438415	0.82	0.69	0.97	0.02	0.13
Stromal.Up_Nat.Med.2009.PMID.19648928	1.08	0.92	1.28	0.35	0.70
SW480.cancer.cells_Immunity.2013.PMID.24138885	1.31	1.10	1.56	0.003	0.08
T.follicular.helper.cell_CellRep.2017.PMID.28052254	0.83	0.70	0.99	0.04	0.18
Tcell.activation_Nature.2020.PMID.31942077	0.86	0.73	1.00	0.05	0.22
Tcell.CD8.Effector.vs.naive.2_Science.2016.PMID27789795	0.96	0.81	1.13	0.59	0.84
Tcell.CD8.Exhausted.vs.antiPDL1.2_Science.2016.PMID27789795	0.98	0.83	1.16	0.83	0.95
Tcell.CD8.Exhausted.vs.naive.2_Science.2016.PMID27789795	0.94	0.80	1.11	0.48	0.77
Tcell.CD8.Memory.vs.naive.1_Science.2016.PMID27789795	0.86	0.73	1.01	0.07	0.28
Tcell.cluster_CCR.2014.PMID.24916698	0.77	0.65	0.92	0.003	0.08
Tcell.EXH.Anti.PDL1.vs.control.treated.exhausted.CD8.Tcell.Metagene.1.Science.2016.PMID.27789795	0.99	0.84	1.16	0.87	0.96
Tcell.EXH.Effector.CD8.T.cell.at.day.8.p.i.Armstrong.vs.Naive.CD8.Tcell.Metagene.1_Science.2016.PMID.27789795	0.85	0.72	1.01	0.07	0.28
Tcell.EXH.Exhausted.CD8.T.cell.vs.Naive.CD8.T.cell.Metagene.1_Science.2016.PMID.27789795	0.92	0.78	1.10	0.37	0.70

Tcell.EXH.Exhausted.CD8.T.cell.vs.Naive.CD8.T.cell.Metagene.3_Science.2016.PMID.27789795	0.86	0.73	1.02	0.08	0.32
Tcell.EXH.Memory.CD8.T.cell.a.vs.Naive.CD8.T.cell.Metagene.1_Science.2016.PMID.27789795	0.86	0.73	1.01	0.07	0.28
Tcell.EXH.Memory.CD8.T.cell.a.vs.Naive.CD8.T.cell.Metagene.2_Science.2016.PMID.27789795	0.89	0.75	1.06	0.18	0.50
Tcell.EXH.Memory.CD8.T.cell.a.vs.Naive.CD8.T.cell.Metagene.3.Science_2016.PMID.27789795	0.85	0.72	1.02	0.08	0.30
Tcell.NK.51gene_Genome.Biol.2013.PMID.23618380	0.79	0.67	0.94	0.009	0.10
Tcell.NK.Metagene_Genome.Biol.2013.PMID.23618380	0.79	0.66	0.93	0.006	0.10
Tcell.RM_Nat_Med.2018.PMID.29942092	0.82	0.69	0.97	0.02	0.13
Tcell.survival.2gene_Nature.2020.PMID.31942077	0.82	0.70	0.96	0.01	0.11
Tcells_CancerImmunolRes.2018.PMID.30266715	0.80	0.67	0.95	0.01	0.11
Tcells_Immunity.2013.PMID.24138885	0.81	0.68	0.95	0.01	0.11
Tcells_TFH_Nat.Methods.2015.PMID.25822800	0.81	0.68	0.96	0.01	0.11
Tcells.CD4.memory.activated_Nat.Methods.2015.PMID.25822800	0.77	0.65	0.91	0.003	0.08
Tcells.CD4.memory.resting_Nat.Methods.2015.PMID.25822800	0.82	0.69	0.97	0.02	0.13
Tcells.CD4.naive_Nat.Methods.2015.PMID.25822800	0.83	0.70	0.99	0.03	0.17
Tcells.CD8_Immunity.2013.PMID.24138885	0.92	0.79	1.08	0.33	0.68
Tcells.CD8_Nat.Methods.2015.PMID.25822800	0.80	0.68	0.95	0.01	0.11
Tcells.CD8.MCP_Nature.2020.PMID.31942075	0.91	0.77	1.07	0.24	0.58
Tcells.Cytotoxic.MCP_Nature.2020.PMID.31942075	0.84	0.71	1.00	0.04	0.20
Tcells.gammadelta_Nat.Methods.2015.PMID.25822800	0.79	0.67	0.94	0.007	0.10
Tcells.helper_Immunity.2013.PMID.24138885	0.93	0.79	1.10	0.39	0.71
Tcells.MCP_Nature.2020.PMID.31942077	0.79	0.67	0.94	0.007	0.10
Tcells.regulatory.2gene_Nature.2020.PMID.31942077	0.93	0.78	1.09	0.36	0.70
Tcells.Tregs_Nat.Methods.2015.PMID.25822800	0.82	0.69	0.98	0.03	0.15
TCGA.BRCA.1198_BASAL_JCI.2020.PMID.32573490	1.06	0.89	1.26	0.54	0.81
TCGA.BRCA.1198_Chromogranin_JCI.2020.PMID.32573490	0.98	0.83	1.17	0.86	0.95
TCGA.BRCA.1198_COLLAGEN11A_JCI.2020.PMID.32573490	1.05	0.89	1.25	0.55	0.81
TCGA.BRCA.1198_EN1_FDZ9_JCI.2020.PMID.32573490	0.92	0.76	1.11	0.39	0.71
TCGA.BRCA.1198_FGFR4_EGF_JCI.2020.PMID.32573490	0.93	0.78	1.10	0.38	0.71
TCGA.BRCA.1198_HISTONES_JCI.2020.PMID.32573490	1.24	1.05	1.47	0.01	0.11
TCGA.BRCA.1198_HOXC11_HOTAIR_SIX1_JCI.2020.PMID.32573490	1.08	0.91	1.28	0.38	0.71
TCGA.BRCA.1198_IL8_CCL_JCI.2020.PMID.32573490	0.86	0.72	1.02	0.09	0.33
TCGA.BRCA.1198_immune_CD19_JCI.2020.PMID.32573490	0.77	0.65	0.92	0.004	0.08
TCGA.BRCA.1198_immune_CD34_TIE1_JCI.2020.PMID.32573490	1.02	0.87	1.21	0.80	0.94
TCGA.BRCA.1198_immune_CD4_CD53_CD84_BTK_JCI.2020.PMID.32573490	0.81	0.68	0.96	0.01	0.11
TCGA.BRCA.1198_immune_CD8_GZMK_JCI.2020.PMID.32573490	0.78	0.66	0.93	0.005	0.09
TCGA.BRCA.1198_immune_CTLA4_CXCL_FOXP3_JCI.2020.PMID.32573490	0.84	0.71	0.99	0.04	0.18
TCGA.BRCA.1198_immune_FOS_JUN_IL6_JCI.2020.PMID.32573490	1.08	0.93	1.26	0.29	0.65
TCGA.BRCA.1198_immune_GIMAP_IL16_JCI.2020.PMID.32573490	0.88	0.74	1.05	0.15	0.44
TCGA.BRCA.1198_immune_HLA_A_F_JCI.2020.PMID.32573490	0.94	0.80	1.11	0.48	0.77
TCGA.BRCA.1198_immune_HLA_D_JCI.2020.PMID.32573490	0.80	0.68	0.95	0.009	0.10
TCGA.BRCA.1198_immune_INTERFERON_JCI.2020.PMID.32573490	1.14	0.97	1.34	0.12	0.39
TCGA.BRCA.1198_IMMUNE1_JCI.2020.PMID.32573490	0.71	0.60	0.84	<0.001	0.02
TCGA.BRCA.1198_LUMINAL_JCI.2020.PMID.32573490	1.08	0.86	1.36	0.50	0.78
TCGA.BRCA.1198_MYBL2_APOBEC3B_JCI.2020.PMID.32573490	0.92	0.78	1.09	0.34	0.70
TCGA.BRCA.1198_NORMAL_JCI.2020.PMID.32573490	0.96	0.81	1.14	0.65	0.86
TCGA.BRCA.1198_NORMAL2_JCI.2020.PMID.32573490	0.95	0.81	1.12	0.55	0.81
TCGA.BRCA.1198_PDCHA_MANY_JCI.2020.PMID.32573490	1.01	0.86	1.19	0.93	0.99
TCGA.BRCA.1198_S100A7_8_9_JCI.2020.PMID.32573490	0.94	0.77	1.15	0.55	0.81

TCGA.BRCA.1198_TP63_JCI.2020.PMID.32573490	1.08	0.92	1.28	0.35	0.70
TCGA.BRCA.1198.IMMUNOGLOBULIN_JCI.2020.PMID.32573490	0.71	0.60	0.83	<0.001	0.02
TCGA.CSF1.response_Immunity.2018.PMID.29628290	0.80	0.68	0.96	0.01	0.11
TCGA.IFN.score_Immunity.2018.PMID.29628290	1.14	0.97	1.35	0.11	0.37
TCGA.Liexpression.score_Immunity.2018.PMID.29628290	0.80	0.68	0.95	0.009	0.10
TCGA.Serum.response.up_Immunity.2018.PMID.29628290	0.92	0.78	1.08	0.31	0.66
TCGA.TFH_Immunity.2018.PMID.29628290	0.97	0.83	1.15	0.76	0.92
TCGA.Tgd_Immunity.2018.PMID.29628290	1.06	0.90	1.24	0.48	0.77
TCGA.TGFB.score_Immunity.2018.PMID.29628290	1.25	1.05	1.49	0.01	0.11
Tcm_Immunity.2013.PMID.24138885	0.92	0.78	1.09	0.35	0.70
Tem_Immunity.2013.PMID.24138885	0.98	0.83	1.16	0.80	0.94
TFH_Immunity.2013.PMID.24138885	0.97	0.83	1.15	0.76	0.92
Tgd_Immunity.2013.PMID.24138885	1.06	0.90	1.24	0.48	0.77
Th1_cells_Immunity.2013.PMID.24138885	0.81	0.68	0.97	0.02	0.13
Th17_cells_Immunity.2013.PMID.24138885	0.96	0.81	1.13	0.62	0.85
Th2_cells_Immunity.2013.PMID.24138885	0.97	0.82	1.15	0.75	0.92
TLS.9Gene.Signature_Nature.2020.PMID.31942071	0.90	0.76	1.06	0.20	0.52
TLS.CXCL13.SingleGene_Nature.2020.PMID.31942077	0.80	0.68	0.93	0.005	0.09
TLS.Hallmark.Gene.Signature_Nature.2020.PMID.31942071	0.80	0.68	0.95	0.010	0.10
TLS.Known.Markers_Nature.2020.PMID.31942071	0.78	0.65	0.92	0.003	0.08
TLS.Structure.12chemokine_FrontImmunol.2017.PMID.28713385	0.81	0.69	0.96	0.02	0.11
TLS.tumors.wTLS.and.CD8.vs.CD8alone_Nature.2020.PMID.31942071	0.78	0.66	0.93	0.006	0.10
TNBC.good.prognosis.TNBC.230genes_BCR.2011.PMID.21978456	0.98	0.83	1.15	0.80	0.94
TNBC.good.prognosis.TNBC.26genes_BCR.2011.PMID.21978456	0.87	0.73	1.02	0.09	0.33
TNBC.metastasis.free.survival_PLoS.One.2013.PMID.24349199	0.80	0.68	0.95	0.009	0.10
TNBC.poor.prognosis.TNBC.26genes_BCR.2011.PMID.21978456	0.98	0.84	1.15	0.82	0.94
Translation.Pathway_CancerImmunolRes.2018.PMID.30266715	1.05	0.89	1.24	0.54	0.81
Tumour.hypoxia.causes.DNA.hypermethylation_Nature.2016.PMID.27533040	1.08	0.91	1.28	0.38	0.71
Type.1.T.helper.cell_CellRep.2017.PMID.28052254	0.82	0.69	0.97	0.02	0.12
Type.17.T.helper.cell_CellRep.2017.PMID.28052254	0.91	0.77	1.07	0.27	0.62
Type.2.T.helper.cell_CellRep.2017.PMID.28052254	0.93	0.79	1.09	0.37	0.70
Up.Basal.High_Nat.Cell.Biol.2014.PMID.25173976	1.06	0.89	1.26	0.50	0.78
Up.Proliferation_Nat.Cell.Biol.2014.PMID.25173976	0.94	0.80	1.12	0.49	0.78
Upregulated.by.oncogenic.NRAS.basal_Cell.Rep.2016.PMID.26166574	1.00	0.85	1.18	>0.99	>0.99
Upregulated.upon.NRAS.repression.basal_Cell.Rep.2017.PMID.26166574	1.04	0.88	1.22	0.66	0.86
Vascular.Content_Clin.Exp.Metastasis.2014.PMID.23975155	1.28	1.07	1.54	0.008	0.10
VEGF.13genes_BMC.Med.2009.PMID.19291283	1.04	0.88	1.24	0.64	0.86
Wirapati.Proliferation_BCR.2008.PMID.18662380	1.03	0.87	1.21	0.77	0.92
Wound.Signature_CCR.2009.PMID.19887484	1.12	0.95	1.32	0.19	0.51
X11q13.Amplicon_BMC.Med.Genomics.2011.PMID.21214954	1.12	0.97	1.30	0.12	0.40
X12qMDM4.BMC.Med.Genomics.2011.PMID.21214954	1.03	0.86	1.22	0.78	0.93
X13q14.Amplicon_BMC.Med.Genomics.2011.PMID.21214954	0.93	0.80	1.08	0.36	0.70
X15q25.Amplicon_BMC.Med.Genomics.2011.PMID.21214954	0.89	0.75	1.05	0.16	0.47
X16.13.Amplicon_BMC.Med.Genomics.2011.PMID.21214954	1.05	0.89	1.25	0.54	0.81
X16q23.Amplicon_BMC.Med.Genomics.2011.PMID.21214954	0.88	0.76	1.03	0.12	0.39
X17PP13.Amplicon_BMC.Med.Genomics.2011.PMID.21214954	0.85	0.71	1.01	0.07	0.27
X17q25x.BMC.Med.Genomics.2011.PMID.21214954	0.96	0.82	1.14	0.66	0.86
X19p13.Amplicon_BMC.Med.Genomics.2011.PMID.21214954	0.96	0.81	1.13	0.59	0.84

X1p36.Amplicon_BMC.Med.Genomics.2011.PMID.21214954	0.90	0.76	1.06	0.20	0.53
X3p21.Amplicon_BMC.Med.Genomics.2011.PMID.21214954	0.89	0.75	1.05	0.17	0.48
X4p16.Amplicon_BMC.Med.Genomics.2011.PMID.21214954	1.03	0.88	1.21	0.71	0.89
X5Q_BCRT.2012.PMID.22048815	0.94	0.79	1.11	0.45	0.76
X8p.Amplicon_BMC.Med.Genomics.2011.PMID.21214954	1.01	0.85	1.18	0.94	>0.99
X8p22.Amplicon_BMC.Med.Genomics.2011.PMID.21214954	0.96	0.82	1.14	0.66	0.86
XBP1.Signature_Nature.2014.PMID.24670641	1.00	0.84	1.18	0.96	>0.99

CALGB 40601					
Signature	HR	95% CI		*P*	adjusted *P*
Activate.Endothelium_Clin.Exp.Metastasis.2014.PMID.23975155	1.19	0.90	1.59	0.23	0.59
Activated.B.cell_CellRep.2017.PMID.28052254	0.70	0.51	0.96	0.02	0.29
Activated.Blood.Neutrophil.Signature_Nat.Cell.Biol.2019.PMID.31263265	1.02	0.72	1.43	0.93	0.96
Activated.Cancer.Cell.Signature_Nat.Cell.Biol.2019.PMID.31263265	1.13	0.84	1.52	0.43	0.75
Activated.CD4.T.cell_CellRep.2017.PMID.28052254	0.97	0.74	1.29	0.85	0.94
Activated.CD8.T.cell_CellRep.2017.PMID.28052254	0.70	0.53	0.93	0.01	0.29
Activated.dendritic.cell_CellRep.2017.PMID.28052254	0.78	0.57	1.06	0.11	0.48
Activated.Lung.MSC.Signature_Nat.Cell.Biol.2019.PMID.31263265	1.42	0.98	2.06	0.06	0.41
Activated.Lung.Neutrophil.Signature_Nat.Cell.Biol.2019.PMID.31263265	1.32	0.96	1.80	0.08	0.47
aDC_Immunity.2013_PMID.24138885.PMID.24138885	0.79	0.57	1.09	0.15	0.53
ADM.S100A10.A110NDGR1.Cluster_BMC.Med.Genomics.2011.PMID.21214954	1.01	0.74	1.39	0.93	0.96
African.and.European.Ancestry.TCGA.Negative_JAMA.Oncol.2017.PMID.28472234	1.01	0.78	1.30	0.94	0.97
African.and.European.Ancestry.TCGA.Positive_JAMA.Oncol.2017.PMID.28472234	1.04	0.78	1.38	0.81	0.93
Age.associated.signature_Genome.Biol.2015.PMID.26343147	0.94	0.68	1.29	0.68	0.87
aMaSC_BCR.2010.PMID.20346151	1.04	0.80	1.36	0.75	0.92
aMaSC.HsEnriched_BCR.2015.PMID.25575446	1.21	0.92	1.58	0.17	0.56
aMaSC.HsEnriched.Refined1_BCR.2015.PMID.25575446	1.04	0.80	1.35	0.76	0.92
aMaSC.Lim09_BCR.2015.PMID.25575446	1.05	0.77	1.44	0.76	0.92
aMaSC.Prat_BCR.2015.PMID.25575446	1.14	0.85	1.54	0.38	0.72
aMaSC.Shehata_BCR.2015.PMID.25575446	0.91	0.66	1.24	0.54	0.79
aMaSC.Signature_Cell.Stem.Cell.2012.PMID.22305568	0.93	0.69	1.26	0.65	0.85
AMPH.EPIREGULIN.Cluster_BMC.Med.Genomics.2011.PMID.21214954	0.89	0.69	1.15	0.39	0.72
Amplification.50_Genome.Biol.2014.PMID.25164602	1.22	0.91	1.64	0.19	0.57
Amplification.50.better.than._Genome.Biol.2015.PMID.25164602	1.25	0.94	1.66	0.13	0.51
Apocrine.Features_J.Pathol.2017.PMID.27861902	1.04	0.74	1.46	0.82	0.93
aStr.HsEnriched_BCR.2015.PMID.25575446	0.96	0.74	1.26	0.79	0.93
aStr.HsEnriched.Refined1_BCR.2015.PMID.25575446	1.06	0.81	1.38	0.68	0.87
aStr.HsEnriched.Refined2_BCR.2015.PMID.25575446	0.97	0.75	1.25	0.82	0.93
aStr.Lim09_BCR.2015.PMID.25575446	1.02	0.78	1.33	0.88	0.95
aStr.Prat_BCR.2015.PMID.25575446	1.03	0.79	1.36	0.81	0.93
aStr.Shehata_BCR.2015.PMID.25575446	1.08	0.82	1.43	0.56	0.80
BASAL.Cluster_BMC.Med.Genomics.2011.PMID.21214954	0.86	0.65	1.14	0.30	0.67
Bcell.cluster_CCR.2014.PMID.24916698	0.67	0.49	0.92	0.01	0.29
Bcell.IL10.MINUS_Immunol.2014.PMID.25080484	1.01	0.75	1.36	0.96	0.98
Bcell.IL10.PLUS_Immunol.2014.PMID.25080484	0.96	0.69	1.34	0.83	0.93

Bcell.lineage.MCP_Nature.2020.PMID.31942077	0.69	0.50	0.95	0.02	0.29
Bcell.Plasma.52gene_Genome.Biol.2013.PMID.23618380	0.60	0.44	0.81	0.001	0.15
Bcell.Plasma.Metagene_Genome.Biol.2013.PMID.23618380	0.61	0.45	0.82	0.001	0.15
Bcell.Tcell.Cooperation_Cell.2019.PMID.31730857	0.65	0.47	0.89	0.01	0.29
Bcells_CancerImmunolRes.2018.PMID.30266715	0.68	0.49	0.94	0.02	0.29
Bcells_Immunity.2013.PMID.24138885	0.87	0.63	1.19	0.38	0.72
Bcells.Centroblast_JCO.2015.PMID.25800755	1.08	0.82	1.42	0.59	0.81
Bcells.Centrocyte_JCO.2015.PMID.25800755	0.93	0.67	1.29	0.67	0.87
Bcells.Memory_JCO.2015.PMID.25800755	0.81	0.58	1.13	0.21	0.59
Bcells.memory_Nat.Methods.2015.PMID.25822800	0.69	0.50	0.95	0.02	0.29
Bcells.Naive_JCO.2015.PMID.25800755	1.21	0.86	1.69	0.27	0.65
Bcells.naive_Nat.Methods.2015.PMID.25822800	0.78	0.56	1.07	0.12	0.48
Bcells.Plasmablast_JCO.2015.PMID.25800755	0.96	0.69	1.33	0.81	0.93
Blood.vessels_Immunity.2013.PMID.24138885	1.02	0.79	1.31	0.90	0.95
bMYB.Signature_Oncogene.2009.PMID.19043454	1.10	0.84	1.44	0.50	0.77
C3TAG.Responding_CCR.2013.PMID.23780888	0.74	0.56	0.97	0.03	0.29
C3TAG.Untreated_CCR.2013.PMID.23780888	1.25	0.96	1.64	0.10	0.48
CD103.Negative_Cancer.Cell.2014.PMID.25446897	0.82	0.62	1.10	0.18	0.56
CD103.Positive_Cancer.Cell.2014.PMID.25446897	0.79	0.57	1.11	0.18	0.56
CD103.Ratio_Cancer.Cell.2014.PMID.25446897	0.82	0.62	1.08	0.16	0.53
CD274_Single_Gene.Single	0.85	0.62	1.16	0.30	0.67
CD34.CD36.Cluster_BMC.Med.Genomics.PMID.21214954	0.87	0.68	1.11	0.25	0.64
CD44.downregulated.genes_Cancer.Cell.2007.PMID.17349583	0.68	0.49	0.94	0.02	0.29
CD44.upregulated.genes_Cancer.Cell.2007.PMID.17349583	1.58	1.11	2.24	0.01	0.29
CD56bright.natural.killer.cell_CellRep.2017.PMID.28052254	1.23	0.89	1.71	0.21	0.59
CD56dim.natural.killer.cell_CellRep.2017.PMID.28052254	1.28	0.97	1.70	0.08	0.46
CD68.cluster_CCR.2014.PMID.24916698	1.02	0.76	1.35	0.91	0.96
CD8.cluster_CCR.2014.PMID.24916698	0.71	0.53	0.95	0.02	0.29
CDKN2A_Single_Gene.Single	1.09	0.81	1.47	0.56	0.80
Central.memory.CD4.T.cell_CellRep.2017.PMID.28052254	0.94	0.69	1.28	0.70	0.88
Central.memory.CD8.T.cell_CellRep.2017.PMID.28052254	1.03	0.76	1.38	0.86	0.94
CES.Score_CCR.2017.PMID.27903675	0.68	0.46	1.00	0.05	0.38
Chromogramin_BMC.Med.Genomics.2011.PMID.21214954	1.04	0.78	1.39	0.77	0.92
CIN70_Nat.Genet.2006.PMID.16921376	1.17	0.89	1.54	0.25	0.63
Claudin.High_Genome.Biol.2007.PMID.17493263	0.80	0.58	1.09	0.15	0.53
Claudin.Low_Genome.Biol.2007.PMID.17493263	1.08	0.82	1.42	0.56	0.80
Claudin.Low.29_Cancer.Res.2009.PMID.19435916	1.07	0.83	1.38	0.60	0.82
cMYB.Signature_PLoS.One.2010.PMID.20949095	1.12	0.79	1.59	0.53	0.79
CORE.Bcell.signature.Garber_Cell.Mol.Gastroenterol.Hepatol.2017.PMID.28508029	0.81	0.59	1.11	0.19	0.57
CTLA4_Single_Gene.Single	0.83	0.62	1.11	0.21	0.59
Cytolytic.activity_Cell.2015.PMID.25594174	0.66	0.48	0.91	0.01	0.29
Cytotoxic.cells_Immunity.2013.PMID.24138885	0.75	0.56	1.01	0.06	0.40
Day7.Downregulated_Nat.Cell.Biol.2014.PMID.25173976	0.93	0.69	1.25	0.63	0.84
Day7.Upregulated_Nat.Cell.Biol.2014.PMID.25173976	1.06	0.79	1.43	0.70	0.88
DC_Immunity.2013.PMID.24138885	0.80	0.62	1.04	0.09	0.47
DCIS.HGF.down_BCR.2013.PMID.24025166	1.15	0.84	1.58	0.39	0.72
DCIS.HGF.up_BCR.2014.PMID.24025166	0.89	0.66	1.20	0.44	0.75
Delection.50_Genome.Biol.2016.PMID.25164602	1.02	0.77	1.35	0.88	0.95

Delection.50.better.than_Genome.Biol.2017.PMID.25164602	1.28	0.95	1.72	0.10	0.48
Dendritic.cells.activated_Nat.Methods.2015.PMID.25822800	0.88	0.64	1.20	0.41	0.73
Dendritic.cells.resting_Nat.Methods.2015.PMID.25822800	0.82	0.60	1.13	0.22	0.59
Down.Basal.High_Nat.Cell.Biol.2014.PMID.25173976	0.83	0.63	1.11	0.21	0.59
Down.CLOW.High_Nat.Cell.Biol.2014.PMID.25173976	1.01	0.78	1.29	0.97	0.98
Downregulated.upon.NRAS.repression.basal_Cell.Rep.2015.PMID.26166574	0.89	0.64	1.25	0.51	0.78
Ductal.Carcinoma.In.Situ_J.Pathol.2017.PMID.27861902	0.90	0.67	1.20	0.46	0.76
Duke.Module01.acidosis_PNASUSA.2010.PMID.20335537	0.83	0.64	1.08	0.18	0.56
Duke.Module02.akt_PNASUSA.2010.PMID.20335537	0.74	0.53	1.03	0.07	0.44
Duke.Module03.betacatenin_PNASUSA.2010.PMID.20335537	1.13	0.83	1.52	0.44	0.75
Duke.Module04.E2F1_PNASUSA.2010.PMID.20335537	1.14	0.83	1.57	0.41	0.73
Duke.Module05.EGFR_PNASUSA.2010.PMID.20335537	1.24	0.94	1.64	0.13	0.51
Duke.Module06.ER_PNASUSA.2010.PMID.20335537	0.95	0.68	1.35	0.79	0.93
Duke.Module07.glucosedepletion_PNASUSA.2010.PMID.20335537	1.50	1.09	2.06	0.01	0.29
Duke.Module08.HER2_PNASUSA.2010.PMID.20335537	1.01	0.74	1.36	0.97	0.98
Duke.Module09.hypoxia_PNASUSA.2010.PMID.20335537	1.13	0.84	1.51	0.41	0.73
Duke.Module10.IFNA_PNASUSA.2010.PMID.20335537	0.98	0.71	1.35	0.89	0.95
Duke.Module11.IFNG_PNASUSA.2010.PMID.20335537	1.05	0.76	1.45	0.77	0.92
Duke.Module12.lacticacidosis_PNASUSA.2010.PMID.20335537	1.16	0.85	1.59	0.36	0.72
Duke.Module13.myc_PNASUSA.2010.PMID.20335537	0.98	0.72	1.32	0.88	0.95
Duke.Module14.p53_PNASUSA.2010.PMID.20335537	0.88	0.62	1.24	0.46	0.76
Duke.Module15.p63_PNASUSA.2010.PMID.20335537	0.91	0.66	1.24	0.54	0.79
Duke.Module16.pi3k_PNASUSA.2010.PMID.20335537	1.32	0.98	1.78	0.07	0.43
Duke.Module17.PR_PNASUSA.2010.PMID.20335537	1.08	0.76	1.54	0.67	0.87
Duke.Module18.ras_PNASUSA.2010.PMID.20335537	1.23	0.89	1.72	0.21	0.59
Duke.Module19.src_PNASUSA.2010.PMID.20335537	0.76	0.51	1.13	0.17	0.56
Duke.Module20.STAT3_PNASUSA.2010.PMID.20335537	1.17	0.83	1.66	0.36	0.72
Duke.Module21.TGFB_PNASUSA.2010.PMID.20335537	1.37	1.01	1.85	0.04	0.37
Duke.Module22.TNFA_PNASUSA.2010.PMID.20335537	1.03	0.76	1.38	0.87	0.94
Durvalumab.signature_CCR.2018.PMID.29716923	0.89	0.65	1.21	0.46	0.76
Early.IRS.1_PLoS.One.2016.PMID.26991655	1.27	0.94	1.70	0.12	0.48
Early.IRS.2_PLoS.One.2016.PMID.26991655	1.22	0.86	1.74	0.27	0.65
Early.Relapse.ERPos.33genes_JAMA.2011.PMID.21558518	1.10	0.81	1.49	0.56	0.80
Early.Response.ERNeg.27genes_JAMA.2011.PMID.21558518	0.96	0.69	1.33	0.79	0.93
Effector.memeory.CD4.T.cell_CellRep.2017.PMID.28052254	0.95	0.67	1.34	0.75	0.92
Effector.memeory.CD8.T.cell_CellRep.2017.PMID.28052254	0.81	0.59	1.12	0.21	0.59
EGFR_Single_Gene.Single	0.99	0.71	1.37	0.93	0.96
EMT.down.Taube_PNAS.2010.PMID.20713713	0.97	0.75	1.24	0.80	0.93
EMT.down.Weingberg_PNAS.2010.PMID.20713713	0.97	0.76	1.25	0.84	0.93
EMT.up.Taube_PNAS.2010.PMID.20713713	1.28	0.96	1.71	0.09	0.48
EMT.up.Weinberg_PNAS.2010.PMID.20713713	1.23	0.90	1.70	0.19	0.57
Endothelial.cells.MCP_Nature.2020..PMID.31942077	1.10	0.85	1.41	0.48	0.76
Endothelial.Normal_Angiogenesis.2014.PMID.24257808	1.12	0.84	1.49	0.43	0.75
Endothelial.Tumor_Angiogenesis.2014.PMID.24257808	0.91	0.64	1.28	0.58	0.80
Eosinophil_CellRep.2017.PMID.28052254	0.87	0.65	1.18	0.38	0.72
Eosinophils_Immunity.2013.PMID.24138885	1.09	0.81	1.47	0.55	0.80
Eosinophils_Nat.Methods.2015.PMID.25822800	0.78	0.60	1.03	0.08	0.47
Epithelial.Tubule.Formation_J.Pathol.2017.PMID.27861902	1.02	0.72	1.45	0.90	0.95

ERBB2_Single_Gene.Single	0.97	0.75	1.26	0.83	0.93
ERBB3_Single_Gene.Single	1.06	0.80	1.40	0.69	0.88
ESR1_Single_Gene.Single	0.87	0.60	1.27	0.46	0.76
ESTIMATE.Immune_Nat.Commun.2013.PMID.24113773	0.80	0.58	1.10	0.17	0.55
ESTIMATE.Stromal_Nat.Commun.2013.PMID.24113773	0.93	0.69	1.23	0.60	0.82
Euclidean.Distance.CLOW_BCR.2010.PMID.20813035	0.92	0.70	1.21	0.56	0.80
EXTENDED.Bcell.signature.Garber_Cell.Mol.Gastroenterol.Hepatol.2017.PMID.28508029	0.88	0.64	1.20	0.41	0.73
FGFR4_Single_Gene.Single	1.16	0.86	1.55	0.33	0.70
FGFR4.Induced_JCI.2020.PMID.32573490	1.06	0.72	1.56	0.77	0.92
FGFR4.Repressed_JCI.2020.PMID.32573490	0.88	0.61	1.25	0.47	0.76
Fibrinogen.Cluster_BMC.Med.Genomics.2011.PMID.21214954	0.99	0.75	1.30	0.94	0.97
Fibroblast.Cluster_BMC.Med.Genomics.2011.PMID.21214954	1.25	0.91	1.71	0.16	0.55
Fibroblasts.MCP_Nature.2020.PMID.31942077	1.02	0.75	1.39	0.89	0.95
Fibromatosis_Lab.Invest.2008.PMID.18414401	1.25	0.90	1.74	0.18	0.56
fMaSC.Metab_CellRep.2018.PMID.30089273	0.88	0.66	1.18	0.41	0.73
fMaSC.Metab8_CellRep.2018.PMID.30089273	0.90	0.66	1.21	0.48	0.76
fMaSC.refined1_BCR.2015.PMID.25575446	0.77	0.56	1.04	0.09	0.48
fMasC.Signature_Cell.Stem.Cell.2012.PMID.22305568	0.77	0.60	1.00	0.05	0.39
fMaSC.Signature_CellRep.2018.PMID.30089273	1.17	0.83	1.65	0.38	0.72
FOS.JUN_Cluster_BMC.Med.Genomics.2011.PMID.21214954	0.92	0.65	1.31	0.64	0.85
FOXC1.Hair.Follicles.P30C.LO.vs.WT.Negative_Science.2016.PMID.26912704	1.30	0.96	1.74	0.09	0.47
FOXC1.Hair.Follicles.P30C.LO.vs.WT.Positive_Science.2016.PMID.26912704	0.88	0.64	1.21	0.45	0.75
fSTR.Signature_Cell.Stem.Cell.2012.PMID.22305568	0.97	0.77	1.23	0.79	0.93
Gamma.delta.T.cell_CellRep.2017.PMID.28052254	1.03	0.76	1.39	0.84	0.93
GATA3.induced.genes_JCO.2006.PMID.16505416	1.02	0.78	1.33	0.88	0.95
GATA3.induced.genes_Oncogene.2004.PMID.15361840	1.00	0.77	1.31	0.98	0.99
GDF11.TGFBR3_Nat.Cell.Biol.2014.PMID.24658685	1.05	0.77	1.42	0.76	0.92
Glycolysis_BMC.Med.2009.PMID.19291283	1.04	0.74	1.45	0.84	0.93
GO.DOWN.with.SOX10.OE_Cell.Rep.2015.PMID.26365194	0.99	0.71	1.37	0.94	0.97
GO.UP.with.SOX10.OE_Cell.Rep.2015.PMID.26365194	1.11	0.83	1.49	0.48	0.76
GSEA_BIOCARTA_ALK_PATHWAY.PMID.16199517	1.15	0.87	1.54	0.33	0.70
GSEA_BIOCARTA.AKT.PATHWAY.PMID.16199517	0.87	0.64	1.18	0.36	0.72
GSEA_BIOCARTA.BRCA.ATR.PATHWAY.ATRBRCA.PMID.16199517	0.99	0.73	1.34	0.96	0.98
GSEA_BIOCARTA.CASPASE.PATHWAY.PMID.16199517	0.89	0.64	1.24	0.51	0.78
GSEA_BIOCARTA.CTLA4.PATHWAY.PMID.16199517	0.69	0.51	0.94	0.02	0.29
GSEA_BIOCARTA.IGF1R.PATHWAY.PMID.16199517	0.82	0.61	1.10	0.18	0.56
GSEA_BIOCARTA.MTOR.PATHWAY.PMID.16199517	0.95	0.69	1.30	0.74	0.92
GSEA_BIOCARTA.PTEN.PATHWAY.PMID.16199517	0.83	0.62	1.11	0.22	0.59
GSEA_BIOCARTA.RAS.PATHWAY.PMID.16199517	0.86	0.65	1.15	0.31	0.68
GSEA_BIOCARTA.RB.PATHWAY.PMID.16199517	1.17	0.86	1.58	0.33	0.70
GSEA_BIOCARTA.VEGF.PATHWAY.PMID.16199517	1.07	0.78	1.47	0.69	0.87
GSEA_HALLMARK.MYC.TARGETS.V1.PMID.16199517	1.25	0.94	1.65	0.12	0.48
GSEA_HELLER.HDAC.TARGETS.DOWN.PMID.16199517	0.91	0.66	1.24	0.53	0.79
GSEA_NELSON.RESPONSE.TO.ANDROGEN.UP.PMID.16199517	1.06	0.76	1.48	0.74	0.92
GSEA_REACTOME.PD1.SIGNALING.PMID.16199517	0.68	0.49	0.94	0.02	0.29
GSEA_REACTOME.PI3K.CASCADE.PMID.16199517	1.07	0.79	1.46	0.65	0.85
GSEA_RETINOL.METABOLISM.KEGG.PMID.16199517	0.83	0.66	1.06	0.14	0.52
GSEA.GP1_Proliferation.DNA.repair..PUJANA.CHEK2.PCC.NETWORK.PMID.25109877	1.14	0.85	1.53	0.37	0.72

GSEA.GP1_Proliferation.DNA.repair.REACTOME.CELL.CYCLE.MITOTIC.PMID.25109877	1.15	0.86	1.54	0.36	0.72
GSEA.GP10_Fatty.acid.oxidation.CARBOXYLIC.ACID.METABOLIC.PROCESS.PMID.25109877	0.82	0.62	1.10	0.19	0.57
GSEA.GP11_Immune.IFN.PerouLab.PMID.25109877	1.02	0.75	1.39	0.90	0.95
GSEA.GP12_Hypoxia.glycolosis.SEMENZA.HIF1.TARGETS.PMID.25109877	1.24	0.94	1.64	0.13	0.51
GSEA.GP13_Neural.signailng.MODULE100.PMID.25109877	0.89	0.68	1.15	0.37	0.72
GSEA.GP13_Neural.signailng.NERVOUS.SYSTEM.DEVELOPMENT.PMID.25109877	1.08	0.80	1.46	0.61	0.83
GSEA.GP14_Plasma.membrane.cell.cell.signaling.MORF.CNTN1.PMID.25109877	0.81	0.58	1.14	0.22	0.59
GSEA.GP15_EGF.signaling.NAGASHIMA.EGF.SIGNALING.UP.PMID.25109877	1.00	0.69	1.43	0.98	0.99
GSEA.GP16_Protein.kinase.signaling.MAPKs.INTRACELLULAR.SIGNALING.CASCADE.PMID.25109877	1.08	0.78	1.50	0.63	0.84
GSEA.GP16_Protein.kinase.signaling.MAPKs.REGULATION.OF.KINASE.ACTIVITY.PMID.25109877	1.32	0.95	1.84	0.10	0.48
GSEA.GP17_Basal.signaling.SMID.BREAST.CANCER.BASAL.UP.PMID.25109877	0.96	0.66	1.40	0.84	0.93
GSEA.GP18_Vesicle.EPR.MEMBRANE.COAT.PMID.25109877	0.93	0.67	1.30	0.68	0.87
GSEA.GP19_1Q.amplicon.PerouLab.PMID.25109877	1.06	0.81	1.38	0.66	0.86
GSEA.GP2_Immune.Tcell.Bcell.KEGG.HEMATOPOIETIC.CELL.LINEAGE.PMID.25109877	0.73	0.53	1.00	0.05	0.38
GSEA.GP2_Immune.Tcell.Bcell.PerouLab.PMID.25109877	0.82	0.59	1.13	0.23	0.59
GSEA.GP20_TAL1.Leukemia.erythropoiesis.GNF2.TAL1.PMID.25109877	1.03	0.74	1.42	0.86	0.94
GSEA.GP21_Anti.apoptosis.DNA.stability.MORF.BCL2.PMID.25109877	0.71	0.51	0.99	0.04	0.36
GSEA.GP21_Anti.apoptosis.DNA.stability.MORF.MT4.PMID.25109877	0.87	0.62	1.20	0.39	0.72
GSEA.GP21_Anti.apoptosis.DNA.stability.MORF.STK17A.PMID.25109877	0.70	0.50	0.96	0.03	0.29
GSEA.GP22_16Q22.24.amplicon.PerouLab.PMID.25109877	1.10	0.82	1.48	0.53	0.79
GSEA.GP3_Tumo.suppressing.miRNA.targets.GTTTGTT.MIR.495.PMID.25109877	1.41	1.04	1.91	0.03	0.29
GSEA.GP3_Tumor.suppressing.miRNA.targets.DACOSTA.UV.RESPONSE.VIA.ERCC3.DN.PMID.25109877	1.25	0.95	1.65	0.11	0.48
GSEA.GP3_Tumor.suppressing.miRNA.targets.TGCTTTG.MIR.330.PMID.25109877	1.40	1.04	1.89	0.03	0.29
GSEA.GP4_MES.ECM.PerouLab.PMID.25109877	1.31	0.94	1.81	0.11	0.48
GSEA.GP5_MYC.targets.TERT.PerouLab.PMID.25109877	1.02	0.76	1.38	0.89	0.95
GSEA.GP6_Squamous.differentiation.development.RICKMAN.TUMOR.DIFFERENTIATED.WELL.VS.POORLY.DN.PMID.25109[illegible]	1.12	0.83	1.53	0.46	0.76
GSEA.GP7_Estrogen.signaling.SMID.BREAST.CANCER.BASAL.DN.PMID.25109877	0.88	0.62	1.26	0.50	0.77
GSEA.GP8_FOXO.stemness.MORF.PTPRB.PMID.25109877	1.05	0.75	1.47	0.79	0.93
GSEA.GP8_FOXO.stemness.TTGTTT.VSFOXO4.01.PMID.25109877	1.15	0.85	1.55	0.38	0.72
GSEA.GP9_Cell.cell.adhesion.PerouLab.PMID.25109877	1.01	0.79	1.30	0.94	0.97
HCK_BCR.2008.PMID.19272155	0.86	0.64	1.15	0.31	0.68
HER1.Cluster1_BMC.Genomics.2007.PMID.17663798	1.09	0.77	1.55	0.61	0.83
HER1.Cluster2_BMC.Genomics.2007.PMID.17663798	1.04	0.76	1.42	0.80	0.93
HER1.Cluster3_BMC.Genomics.2007.PMID.17663798	1.26	0.92	1.71	0.15	0.53
HER2.Amplicon.PerouLab_BMC.Med.Genomic.2011.PMID.21214954	0.90	0.64	1.25	0.53	0.79
Histological.Grade_J.Pathol.2017.PMID.27861902	1.17	0.88	1.56	0.28	0.67
HouseKeeping_Genome.Biol.2004.PMID.15287981	0.98	0.70	1.36	0.88	0.95
iDC.Median_Immunity.2013.PMID.24138885	0.77	0.58	1.03	0.08	0.46
IFN.Cluster_BMC.Med.Genomics.2011.PMID.21214954	1.12	0.82	1.53	0.48	0.76
IgG_BCR.2008.PMID.19272155	0.58	0.44	0.78	<0.001	0.10
IGG.Cluster_BMC.Med.Genomics.2011.PMID.21214954	0.64	0.48	0.87	0.00	0.29
Immature..B.cell_CellRep.2017.PMID.28052254	0.75	0.54	1.04	0.09	0.47
Immature.dendritic.cell_CellRep.2017.PMID.28052254	0.94	0.70	1.26	0.69	0.87
ImmLandscape_Macro.mono.CSF1.core.response_CCR.2009.PMID.29628290	0.83	0.60	1.13	0.23	0.59
ImmLandscape_Wound.Healing_Immunity.2018.PMID.29628290	1.09	0.81	1.45	0.58	0.80
ImmLandscape.IFN3_Plos.One.2014.PMID.24516633	1.15	0.85	1.55	0.36	0.72
ImmLandscape.IFNG5_Plos.One.2014.PMID.24516633	0.72	0.52	1.00	0.05	0.38
ImmLandscape.lymphocyte.Infil.T.B.PMID.18592372	0.70	0.52	0.94	0.02	0.29

Immune.Hot.CD8.vs.Cold_Nature.2020.PMID.31942071	0.75	0.55	1.04	0.08	0.47
Immune.Perez.14_JCO.2015.PMID.25605861	0.69	0.52	0.90	0.01	0.29
Immune.Perez.87_JCO.2015.PMID.25605861	0.67	0.49	0.92	0.01	0.29
Immune.Suppression_JCI.Insight.2016.PMID.27699256	0.84	0.61	1.15	0.27	0.65
ImmuneActive_Cell.2019.PMID.31730857	0.73	0.54	1.00	0.05	0.38
Immunosuppression.PMID.31942077	1.13	0.82	1.57	0.46	0.76
IMS.Score_CCR.2018.PMID.29921729	0.98	0.72	1.32	0.88	0.95
Induced.in.Bcells_PNAS.2013.PMID.23382184	0.99	0.72	1.34	0.93	0.97
Induced.in.DC_PNAS.2013.PMID.23382184	0.92	0.67	1.27	0.63	0.84
Induced.in.GN_PNAS.2013.PMID.23382184	0.83	0.61	1.12	0.22	0.59
Induced.in.HSC_PNAS.2013.PMID.23382184	1.06	0.74	1.52	0.75	0.92
Induced.in.MOs_PNAS.2013.PMID.23382184	0.97	0.74	1.28	0.84	0.93
Induced.in.NKcells_PNAS.2013.PMID.23382184	0.91	0.68	1.21	0.50	0.77
Induced.in.Tcells_PNAS.2013.PMID.23382184	0.88	0.63	1.22	0.44	0.75
Inflammatory.breast.cancer.491genes_CCR.2013.PMID.23396049	0.83	0.59	1.17	0.29	0.67
Inflammatory.breast.cancer.79genes_CCR.2013.PMID.23396049	1.14	0.85	1.54	0.38	0.72
Inflammatory.breast.cancer.expressed.noIBC_79genes_CCR.2013.PMID.23396049	1.10	0.82	1.47	0.54	0.79
Inflammatory.breast.cancer.expressed.noIBC.491genes_CCR.2013.PMID.23396049	1.18	0.86	1.62	0.30	0.67
Influenza.11genes.Metasignature_Immunity.2015.PMID.26682989	1.08	0.80	1.45	0.63	0.84
Interferon_BCR.2008.PMID.19272155	1.17	0.87	1.58	0.30	0.67
Interferon.Pathway_CancerImmunolRes.2018.PMID.30266715	1.08	0.80	1.48	0.61	0.83
JUND.KRT5_Nat.Cell.Biol.2014.PMID.24658685	1.03	0.74	1.44	0.84	0.93
Keller2012.CD10.Adam_BCR.2015.PMID.25575446	0.97	0.73	1.28	0.81	0.93
KRAS.amplicon_Genome.Biology.2007.PMID.17493263	1.47	1.06	2.04	0.02	0.29
Late.IRS.1_PLoS.One.2016.PMID.26991655	1.20	0.90	1.60	0.21	0.59
Late.IRS.2_PLoS.One.2016.PMID.26991655	0.88	0.63	1.22	0.44	0.75
LCK_BCR.2008.PMID.19272155	0.73	0.53	0.99	0.05	0.38
Lim2009.LumProg.Adam_BCR.2015.PMID.25575446	0.82	0.59	1.15	0.26	0.64
Lim2009.MaSC.Adam_BCR.2015.PMID.25575446	0.88	0.67	1.15	0.34	0.71
Lim2009.MatureLum.Adam_BCR.2015.PMID.25575446	1.05	0.74	1.50	0.78	0.93
Lim2009.Stroma.Adam_BCR.2015.PMID.25575446	1.00	0.78	1.28	0.98	0.99
Lim2010.LumProg.Adam_BCR.2015.PMID.25575446	0.89	0.65	1.22	0.47	0.76
Lim2010.MaSC.Adam_BCR.2015.PMID.25575446	0.89	0.67	1.17	0.39	0.72
Lim2010.MatureLum.Adam_BCR.2015.PMID.25575446	0.81	0.60	1.11	0.19	0.57
Lim2010.Stroma.Adam_BCR.2015.PMID.25575446	0.97	0.74	1.26	0.79	0.93
Lobular.Carcinoma.In.Situ_J.Pathol.2017.PMID.27861902	0.97	0.74	1.28	0.84	0.93
LOBULAR.TCGA.SIGNATURE.ImmuneCell.2015.PMID.26451490	0.80	0.61	1.06	0.12	0.48
LOBULAR.TCGA.SIGNATURE.Reactive_Cell.2015.PMID.26451490	0.87	0.67	1.13	0.29	0.67
LOBULAR.TCGA.SUBTYPE.Immune_Cell.2015.PMID.26451490	0.83	0.65	1.05	0.12	0.48
LOBULAR.TCGA.SUBTYPE.Proliferative_Cell.2015.PMID.26451490	0.86	0.69	1.08	0.19	0.57
LOBULAR.TCGA.SUBTYPE.Reactive_Cell.2015.PMID.26451490	0.81	0.62	1.05	0.12	0.48
LTS.score_JCI.2020.PMID.32573490	0.84	0.64	1.09	0.18	0.56
Luminal_Progenitor_Up_Nat.Med.2009.PMID.19648928	0.75	0.53	1.06	0.10	0.48
Luminal.cluster_BMC.Med.Genomics.2011.PMID.21214954	1.09	0.75	1.58	0.64	0.85
Luminal.Progenitor_BCR.2010.PMID.20346151	0.77	0.57	1.04	0.08	0.47
Luminal.Progenitor.Down_Nat.Med.2009.PMID.19648928	1.27	0.92	1.77	0.15	0.53
LumProg.HsEnriched_BCR.2015.PMID.25575446	0.76	0.54	1.07	0.12	0.48
LumProg.HsEnriched.Refined1_BCR.2015.PMID.25575446	0.74	0.55	1.00	0.05	0.38

LumProg.Lim09_BCR.2015.PMID.25575446	0.79	0.56	1.12	0.18	0.56
LumProg.Prat_BCR.2015.PMID.25575446	0.85	0.63	1.14	0.28	0.66
LumProg.Shehata_BCR.2015.PMID.25575446	0.93	0.68	1.26	0.64	0.85
Lums.HER2E.DOWN.metastatic.signature_JCI.2020.PMID.32573490	1.17	0.84	1.63	0.35	0.72
Lums.HER2E.UP.metastatic.signature_JCI.2020.PMID.32573490	0.94	0.69	1.28	0.71	0.88
Lung.WNT_Cancer.Res.2009.PMID.19549913	1.15	0.83	1.58	0.40	0.72
Lymph.vessels_Immunity.2013.PMID.24138885	1.15	0.82	1.60	0.43	0.75
Lymphovascular.Invasion_J.Pathol.2017.PMID.27861902	0.99	0.73	1.33	0.93	0.96
M.D.Metagene_Genome.Biol.2013.PMID.23618380	0.71	0.51	1.01	0.06	0.40
M2.Macrophage_Blood.2006.PMID.16556895	0.78	0.59	1.03	0.08	0.47
Macrophage_CellRep.2017.PMID.28052254	0.68	0.49	0.94	0.02	0.29
Macrophages_CancerImmunolRes.2018.PMID.30266715	0.84	0.63	1.13	0.26	0.64
Macrophages_Immunity.2013.PMID.24138885	0.96	0.72	1.29	0.80	0.93
Macrophages.M0_Nat.Methods.2015.PMID.25822800	0.91	0.69	1.19	0.48	0.76
Macrophages.M1_Nat.Methods.2015.PMID.25822800	0.90	0.65	1.25	0.53	0.79
Macrophages.M2_Nat.Methods.2015.PMID.25822800	0.85	0.63	1.15	0.29	0.67
MacTh1.cluster_CCR.2014.PMID.24916698	0.80	0.59	1.09	0.16	0.53
MammaPrint_Nature.2002.PMID.11823860	0.83	0.61	1.12	0.22	0.59
MAPK.pathway.activation_NPJ.Precis.Oncol.2018.PMID.29872725	1.18	0.90	1.55	0.23	0.59
MASC.Down_Nat.Med.2009.PMID.19648928	0.96	0.70	1.31	0.81	0.93
MASC.Up_Nat.Med.2009.PMID.19648928	1.04	0.79	1.37	0.76	0.92
Mast.cell_CellRep.2017.PMID.28052254	0.78	0.57	1.07	0.12	0.48
Mast.cells_Immunity.2013.PMID.24138885	0.89	0.67	1.18	0.41	0.73
Mast.cells.activated_Nat.Methods.2015.PMID.25822800	0.74	0.57	0.98	0.04	0.35
Mast.cells.resting_Nat.Methods.2015.PMID.25822800	0.73	0.57	0.95	0.02	0.29
Mature.luminal_BCR.2010.PMID.20346151	0.96	0.69	1.34	0.80	0.93
Mature.Luminal.Down_Nat.Med.2009.PMID.19648928	0.96	0.71	1.28	0.76	0.92
Mature.LuminaUp_Nat.Med.2009.PMID.19648928	1.05	0.74	1.48	0.79	0.93
MatureLum.HsEnriched_BCR.2015.PMID.25575446	0.97	0.69	1.36	0.86	0.94
MatureLum.HsEnriched.Refined1_BCR.2015.PMID.25575446	0.90	0.64	1.26	0.54	0.79
MatureLum.Lim09_BCR.2015.PMID.25575446	1.04	0.75	1.43	0.82	0.93
MatureLum.Prat_BCR.2015.PMID.25575446	1.11	0.83	1.49	0.47	0.76
MatureLum.Shehata_BCR.2015.PMID.25575446	1.13	0.83	1.52	0.44	0.75
MBasal.Cluster_BMC.Med.Genomics.2011.PMID.21214954	0.90	0.70	1.17	0.43	0.75
MCD3.CD8_BMC.Med.Genomics.2011.PMID.21214954	0.74	0.55	1.01	0.06	0.40
MCF7.E2.induced.genes_JCO.2006.PMID.16505416	0.98	0.73	1.32	0.90	0.95
MCF7.E2.repressed.genes_JCO.2006.PMID.16505416	1.15	0.84	1.58	0.37	0.72
MDSC_CellRep.2017.PMID.28052254	0.75	0.56	1.01	0.06	0.40
MDSC.Granulocytic_Leukoc.Biol.2012.PMID.21954284	0.89	0.64	1.24	0.50	0.77
MDSC.Neutrophi_Leukoc.Biol.2012.PMID.21954284	0.83	0.62	1.11	0.21	0.59
MDSC.tumor_J.Immunol.2012.PMID.23152559	1.04	0.76	1.42	0.82	0.93
MDSC.tumor.MO_J.Immunol.2012.PMID.23152559	0.89	0.65	1.23	0.48	0.76
MECM_BMC.Med.Genomics.2011.PMID.21214954	0.96	0.74	1.24	0.74	0.92
Memory.B.cell_CellRep.2017.PMID.28052254	1.15	0.84	1.56	0.39	0.72
MET.DOWN.RNAseq.Significant.Genes_JCI.2018.PMID.29480819	1.10	0.77	1.57	0.61	0.83
MET.DOWN.Significant.Genes.Low.Basal.1_JCI.2018.PMID.29480819	1.25	0.92	1.71	0.16	0.53
MET.DOWN.Significant.Genes.Low.Basal.2_JCI.2018.PMID.29480819	1.19	0.88	1.60	0.26	0.64
MET.UP.RNAseq.Significant.Genes_JCI.2018.PMID.29480819	1.09	0.81	1.47	0.56	0.80

MET.UP.Significant.Genes.HIGH.BASALS.Genes_JCI.2018.PMID.29480819	0.92	0.65	1.30	0.63	0.84
Metaplastic.Up_CanRes.2009.PMID.19435916	1.15	0.87	1.51	0.33	0.70
Metastasis.predictor.TNBC_BCR.2010.PMID.20946665	0.98	0.71	1.35	0.89	0.95
MFGFR2_BMC.Med.Genomics.2011.PMID.21214954	1.19	0.86	1.65	0.29	0.67
MHC.Forero.11_Cancer.Immunol.Res.2016.PMID.26980599	0.78	0.54	1.14	0.20	0.57
MHC.Forero.24_Cancer.Immunol.Res.2016.PMID.26980599	0.63	0.44	0.89	0.01	0.29
MHC.I_BCR.2008.PMID.19272155	0.88	0.61	1.26	0.48	0.76
MHC.II_BCR.2008.PMID.19272155	0.76	0.55	1.05	0.10	0.48
MHCI.coreGenes_Nat.Commun.2017.PMID29170503	0.97	0.70	1.33	0.84	0.93
MIR200c.Induced_ONCO.2015.PMID.25746005	0.87	0.65	1.17	0.36	0.72
MIR200c.Repressed_ONCO.2015.PMID.25746005	1.17	0.84	1.63	0.36	0.72
miRNA.138.signature_Cancer.Res.2014.PMID.25339353	1.23	0.90	1.68	0.20	0.57
MITO1_BMC.Med.Genomics.2011.PMID.21214954	1.05	0.76	1.46	0.75	0.92
MITO2_BMC.Med.Genomics.2011.PMID.21214954	1.23	0.85	1.80	0.27	0.66
Mitotic.Count_J.Pathol.2017.PMID.27861902	1.17	0.87	1.56	0.29	0.67
MK14.K17_BMC.Med.Genomics.2011.PMID.21214954	1.06	0.80	1.41	0.67	0.87
MKRAS.amplicon_BMC.Med.Genomics.2011.PMID.21214954	1.32	0.94	1.86	0.11	0.48
MM.BRCAwnt.1pFDR.UP_Genome.Biology.2007.PMID.17493263	1.48	1.09	2.01	0.01	0.29
MM.C3Tag.1pFDR.UP_Genome.Biology.2007.PMID.17493263	1.15	0.85	1.56	0.36	0.72
MM.C3Tag.2012_Genome.Biol.2013.PMID.24220145	1.16	0.88	1.53	0.30	0.67
MM.Class3_Genome.Biol.2013.PMID.24220145	0.85	0.62	1.16	0.30	0.67
MM.Class8_Genome.Biol.2013.PMID.24220145	1.19	0.83	1.70	0.34	0.70
MM.Claudinlow_Genome.Biol.2013.PMID.24220145	0.95	0.72	1.26	0.74	0.92
MM.DMBAwnt.1pFDR.UP.Genome.Biology.2007.PMID.17493263	0.98	0.73	1.32	0.88	0.95
MM.Erbb2.like_Genome.Biol.2013.PMID.24220145	0.80	0.61	1.04	0.09	0.48
MM.Myc.2012_Genome.Biol.2013.PMID.24220145	0.90	0.64	1.25	0.52	0.79
MM.Myoepithelioma.like_Genome.Biol.2013.PMID.24220145	0.92	0.70	1.22	0.58	0.80
MM.Neu.2012_Genome.Biol.2013.PMID.24220145	0.76	0.57	1.02	0.07	0.43
MM.NeuPyMT.1pFDR.UP_Genome.Biology.2007.PMID.17493263	1.01	0.69	1.49	0.95	0.97
MM.Normal.1pFDR.UP_Genome.Biology.2007.PMID.17493263	0.87	0.67	1.11	0.26	0.64
MM.Normal.like_Genome.Biol.2013.PMID.24220145	0.82	0.64	1.05	0.11	0.48
MM.p53null.1pFDR.UP_Genome.Biology.2007.PMID.17493263	1.01	0.74	1.37	0.97	0.98
MM.p53null.Basal_Genome.Biol.2013.PMID.24220145	1.05	0.77	1.44	0.75	0.92
MM.p53null.Luminal_Genome.Biol.2013.PMID.24220145	1.14	0.79	1.65	0.48	0.76
MM.Potluck_1pFDR_UP_Genome.Biology.2007_PMID.17493263.PMID.24220145	0.99	0.74	1.34	0.97	0.98
MM.PyMT.2012_Genome.Biol.2013.PMID.24220145	0.78	0.56	1.07	0.13	0.50
MM.Squamous.like_Genome.Biol.2013.PMID.24220145	0.96	0.71	1.30	0.79	0.93
MM.Stat1_Genome.Biol.2013.PMID.24220145	0.86	0.64	1.17	0.33	0.70
MM.WapINT3.1pFDR.UP_Genome.Biology.2007.PMID.17493263	1.18	0.84	1.64	0.34	0.70
MM.WapINT3.2012_Genome.Biol.2013.PMID.24220145	1.34	0.93	1.93	0.12	0.48
MM.WAPTag.1pFDR.UP_Genome.Biology.2007.PMID.17493263	1.12	0.86	1.45	0.41	0.73
MM.Wnt1.Early_Genome.Biol.2013.PMID.24220145	0.97	0.73	1.30	0.86	0.94
MM.Wnt1.Late_Genome.Biol.2013.PMID.24220145	1.11	0.81	1.50	0.52	0.79
Mmyosin_BMC.Med.Genomics.2011.PMID.21214954	1.12	0.83	1.52	0.45	0.76
MNADH_CYTochrome_BMC.Med.Genomics.2011.PMID.21214954	0.89	0.63	1.24	0.48	0.76
MNB1_BMC.Med.Genomics.2011.PMID.21214954	0.81	0.62	1.07	0.14	0.51
MNB2_BMC.Med.Genomics.2011.PMID.21214954	0.77	0.60	0.98	0.04	0.35
MNB3_BMC.Med.Genomics.2011.PMID.21214954	0.80	0.59	1.09	0.15	0.53

MNOtch4_BMC.Med.Genomics.2011.PMID.21214954	1.07	0.76	1.53	0.69	0.88
Monocyte_CellRep.2017.PMID.28052254	0.63	0.43	0.93	0.02	0.29
Monocyte..DC.25gene_Genome.Biol.2013.PMID.23618380	0.74	0.53	1.02	0.07	0.44
Monocytes_CancerImmunolRes.2018.PMID.30266715	0.77	0.57	1.05	0.09	0.48
Monocytes_Nat.Methods.2015.PMID.25822800	0.85	0.63	1.14	0.28	0.67
Monocytic.lineage.MCP_Nature.2020.PMID.31942075	0.88	0.66	1.16	0.36	0.72
MProliferation_BMC.Med.Genomics.2011.PMID.21214954	1.13	0.86	1.48	0.38	0.72
MProtocadherin_BMC.Med.Genomics.2011.PMID.21214954	0.92	0.69	1.22	0.56	0.80
MPYMT_NEU_Cluster_BMC.Med.Genomics.2011.PMID.21214954	0.86	0.64	1.16	0.32	0.69
MRibosomal_BMC.Med.Genomics.2011.PMID.21214954	0.90	0.64	1.26	0.53	0.79
MS.CD44.DOWN_PNAS.2009.PMID.19666588	1.09	0.82	1.44	0.57	0.80
MS.CD44.UP_PNAS.2009.PMID.19666588	1.02	0.77	1.37	0.88	0.95
MSquamous_BMC.Med.Genomics.2011.PMID.21214954	1.02	0.79	1.33	0.86	0.94
Murat.G07_JCO.2008.PMID.18565887	1.31	0.96	1.79	0.09	0.48
Murat.G18_JCO.2008.PMID.18565887	0.98	0.73	1.33	0.92	0.96
Murat.G24_JCO.2008.PMID.18565887	0.85	0.63	1.15	0.30	0.67
MVEGFC_BMC.Med.Genomics.2011.PMID.21214954	1.03	0.77	1.38	0.84	0.93
Myeloid.cell.chemotaxis.1gene_Nature.2020.PMID.31942077	1.01	0.73	1.41	0.93	0.96
Myeloid.dendritic.cells.MCP_Nature.2020.PMID.31942077	0.70	0.53	0.94	0.02	0.29
Natural.killer.cell_CellRep.2017.PMID.28052254	0.84	0.61	1.15	0.29	0.67
Natural.killer.T.cell_CellRep.2017.PMID.28052254	0.97	0.72	1.30	0.82	0.93
Necrosis_J.Pathol.2017.PMID.27861902	1.32	0.94	1.85	0.11	0.48
Neutrophil_CellRep.2017.PMID.28052254	0.80	0.61	1.05	0.11	0.48
Neutrophils_CancerImmunolRes.2018.PMID.30266715	0.94	0.67	1.31	0.71	0.89
Neutrophils_Immunity.2013.PMID.24138885	0.82	0.62	1.09	0.17	0.56
Neutrophils_Nat.Methods.2015.PMID.25822800	0.94	0.70	1.25	0.66	0.86
Neutrophils.MCP_Nature.2020.PMID.31942077	0.88	0.68	1.12	0.30	0.67
NK_Immunity.2013.PMID.24138885	0.78	0.57	1.09	0.14	0.52
NK.activated_Nat.Methods.2015.PMID.25822800	0.71	0.53	0.96	0.03	0.29
NK.CD56bright_Immunity.2013.PMID.24138885	0.82	0.60	1.13	0.22	0.59
NK.CD56dim_Immunity.2013.PMID.24138885	0.82	0.62	1.08	0.16	0.53
NK.resting_Nat.Methods.2015.PMID.25822800	0.71	0.52	0.96	0.03	0.29
NKcells_CancerImmunolRes.2018.PMID.30266715	0.65	0.47	0.89	0.01	0.29
NKcells.MCP_Nature.2020.PMID.31942077	0.75	0.59	0.97	0.03	0.29
No.Response.Immunotherapy.TLS.Melanoma_Nature.2020.PMID.31942075	1.00	0.75	1.34	>0.99	>0.99
Normal.mucosa_Immunity.2013.PMID.24138885	0.97	0.75	1.26	0.83	0.93
Nuclear.Pleomorphism_J.Pathol.2017.PMID.27861902	0.85	0.62	1.17	0.32	0.70
Oncotype_NEJM.2004.PMID.15591335	1.22	0.92	1.62	0.18	0.56
P53.ERPos.MDACC_CCR.2011.PMID.21248301	1.18	0.89	1.57	0.25	0.62
Parity.signature.251genes_BCR.2014.PMID.25005139	0.83	0.61	1.12	0.22	0.59
Parity.signature.40genes_BCR.2014.PMID.25005139	0.80	0.59	1.10	0.18	0.56
PARPi.Resistance_BCRT_2012.PMID.22875744	1.24	0.94	1.63	0.14	0.51
PARPi.Sensitivity_BCRT_2012.PMID.22875744	1.03	0.77	1.36	0.86	0.94
PARPi.Sensitivity.MDACC_NPJ.Syst.Biol.Appl.2017.PMID.28649435	0.91	0.65	1.29	0.62	0.83
PARPi.Sensitivity.Negative_Sci.Adv.2017.PMID.28439535	0.95	0.68	1.31	0.73	0.91
PARPi.Sensitivity.Positive_Sci.Adv.2017.PMID.28439535	1.07	0.78	1.47	0.68	0.87
Pcorr.Breast2Lung.LM2.Correlation_Nature.2005.PMID.16049480	1.15	0.84	1.57	0.39	0.72
Pcorr.Breast2Lung.Parental.Correlation_Nature.2005.PMID.16049480	0.84	0.61	1.16	0.30	0.67

Pcorr.dasatinib.resistant_Cancer.Res.2007.PMID.17332353	1.03	0.76	1.38	0.87	0.94
Pcorr.dasatinib.sensitive_Cancer.Res.2007.PMID.17332353	0.99	0.73	1.34	0.93	0.96
Pcorr.Hypoxia.High.Correlation_PLoS.Med.2006.PMID.16417408	1.11	0.81	1.53	0.52	0.79
Pcorr.Hypoxia.Low.Correlation_PLoS.Med.2006.PMID.16417408	0.91	0.65	1.26	0.57	0.80
Pcorr.IGS_Invasiveness_NJEM.2007.PMID.17229949	1.27	0.97	1.67	0.09	0.47
Pcorr.wound.response.activated_PNAS.2005.PMID.15701700	1.16	0.86	1.55	0.33	0.70
pCR.predictor.ERNeg.55genes_JAMA.2011.PMID.21558518	1.31	0.96	1.78	0.09	0.47
pCR.predictor.ERPos.39genes_JAMA.2011.PMID.21558518	1.16	0.85	1.58	0.34	0.71
PDCD1_Single_Gene.Single	0.80	0.60	1.08	0.15	0.53
Pfefferle2012.LumProg_BCR.2015.PMID.25575446	0.66	0.47	0.93	0.02	0.29
Pfefferle2012.MaSC_BCR.2015.PMID.25575446	1.06	0.81	1.39	0.67	0.87
Pfefferle2012.MatureLum_BCR.2015.PMID.25575446	1.16	0.82	1.64	0.40	0.73
Pfefferle2012.Stroma_BCR.2015.PMID.25575446	1.00	0.77	1.31	0.97	0.98
PGR_Single_Gene.Single	0.85	0.58	1.23	0.39	0.72
PI3Ki.Down_CancerCell.2017.PMID.28528867	1.18	0.87	1.60	0.29	0.67
PI3Ki.Up_CancerCell.2017.PMID.28528867	1.20	0.87	1.65	0.26	0.64
PIK3CA.Pathway_Ann.Oncol.2017.PMID.28177460	1.06	0.79	1.42	0.69	0.87
PIK3CAmt.signature_Cancer.Res.2012.PMID.22552288	1.30	0.95	1.80	0.10	0.48
Plasma.cells_Nat.Methods.2015.PMID.25822800	0.69	0.51	0.95	0.02	0.29
PlasmaCells_CancerImmunolRes.2018.PMID.30266715	0.73	0.55	0.98	0.04	0.35
Plasmacytoid.dendritic.cell_CellRep.2017.PMID.28052254	0.84	0.64	1.11	0.22	0.59
PR.Isoform.Ratio.Up.in.PRA.H_JNCI.2017.PMID.28376177	0.69	0.51	0.93	0.02	0.29
PR.Isoform.Ratio.Up.in.PRB.H_JNCI.2017.PMID.28376177	1.12	0.83	1.51	0.44	0.75
Proliferation.Cluster_BMC.Med.Genomics.2011.PMID.21214954	1.14	0.87	1.50	0.35	0.72
Proliferation.Metagene_Genome.Biol.2013.PMID.23618380	1.13	0.86	1.49	0.38	0.72
Proliferation.score.PAM50_JCO.2009.PMID.19204204	1.37	1.00	1.88	0.05	0.39
ProliferationPathway_CancerImmunolRes.2018.PMID.30266715	1.12	0.85	1.47	0.41	0.73
Prosigna.Proliferation.18_BMC.Med.Genomics.2015.PMID.26297356	1.12	0.85	1.47	0.43	0.75
Race.LuminalA.MRE.score_BCRT.2015.PMID.26109344	1.28	0.94	1.75	0.12	0.49
Radiation.induced.genes_Radoat.Res.2014.PMID.24527691	1.03	0.73	1.45	0.87	0.95
RB.LOH_BCR.2008.PMID.18782450	1.18	0.89	1.56	0.24	0.62
RB.LOSS_JCI.2007.PMID.17160137	1.11	0.85	1.46	0.45	0.76
Regulatory.T.cell_CellRep.2017.PMID.28052254	0.87	0.63	1.20	0.39	0.72
Replication.Stress.Down.set_Cell.Rep.2018.PMID.29768207	0.85	0.61	1.18	0.32	0.70
Replication.Stress.Model_Cell.Rep.2018_PMID.29768207.PMID.29768207	1.37	0.98	1.90	0.06	0.41
Replication.Stress.Neg_Cell.Rep.2018_PMID.29768207.PMID.29768207	0.80	0.59	1.09	0.15	0.53
Replication.Stress.Pos_Cell.Rep.2018_PMID.29768207.PMID.29768207	1.14	0.84	1.55	0.41	0.73
Replication.Stress.Up_Set_Cell.Rep.2018_PMID.29768207.PMID.29768207	1.16	0.85	1.59	0.35	0.72
Residual.disease.predictor.ERNeg.54genes_JAMA.2011.PMID.21558518	0.78	0.55	1.11	0.17	0.55
Residual.disease.predictor.ERPos.73genes_JAMA.2011.PMID.21558518	1.28	0.95	1.71	0.11	0.48
Response.Immunotherapy.MCP.TLS.Melanoma_Nature.2020.PMID.31942075	0.63	0.47	0.85	0.003	0.29
Response.Immunotherapy.signature_Science.2018.PMID.30309915	0.74	0.55	1.01	0.06	0.40
Response.Neo.Chemo_common_CCR.2014.PMID.25047707	1.18	0.89	1.55	0.25	0.62
Response.Neo.Chemo_ERNeg_CCR.2014.PMID.25047707	0.96	0.69	1.34	0.82	0.93
Response.Neo.Chemo_ERPos_CCR.2014.PMID.25047707	1.09	0.79	1.52	0.59	0.82
RHOA.pathway_Ann.Oncol.2017.PMID.28177460	1.12	0.82	1.52	0.49	0.76
Ribosomal.Cluster_BMC.Med.Genomics.2011.PMID.21214954	0.90	0.66	1.24	0.53	0.79
ROR.subtype.PAM50_JCO.2009.PMID.19204204	1.43	1.02	2.00	0.04	0.35

ROR.subtype.proliferation.PAM50_JCO.2009.PMID.19204204	1.38	1.00	1.91	0.05	0.38
RSS.Score_CCR.2018.PMID.29921729	1.21	0.88	1.69	0.24	0.62
S100A9.A8_BMC.Med.Genomics.2011.PMID.21214954	0.90	0.63	1.29	0.56	0.80
Scorr.EMAT1.Correlation_BCR.2020.PMID.32641077	0.88	0.64	1.20	0.41	0.73
Scorr.EMAT2.Correlation_BCR.2020.PMID.32641077	1.14	0.83	1.57	0.41	0.73
Scorr.EMAT3.Correlation_BCR.2020.PMID.32641077	0.99	0.73	1.36	0.97	0.98
Scorr.EMAT4.Correlation_BCR.2020.PMID.32641077	0.79	0.56	1.11	0.17	0.55
Scorr.IE.Correlation_JCO.2006.PMID.16505416	0.81	0.61	1.08	0.15	0.53
Scorr.IIE.Correlation_JCO.2006.PMID.16505416	1.19	0.90	1.59	0.23	0.59
Scorr.PAM50.Basal_JCO.2009.PMID.19204204	1.23	0.84	1.80	0.29	0.67
Scorr.PAM50.Her2_JCO.2009.PMID.19204204	1.31	0.97	1.78	0.08	0.46
Scorr.PAM50.LumA_JCO.2009.PMID.19204204	0.67	0.47	0.97	0.03	0.33
Scorr.PAM50.LumB_JCO.2009.PMID.19204204	1.22	0.92	1.63	0.17	0.56
Scorr.PAM50.Normal_JCO.2009.PMID.19204204	0.79	0.60	1.05	0.11	0.48
Scorr.S329.L_Br.J.Cancer.2008.PMID.18382427	1.08	0.78	1.49	0.65	0.85
Scorr.S329.R_Br.J.Cancer.2008.PMID.18382427	0.91	0.65	1.27	0.58	0.80
Secretoglobin_BMC.Med.Genomics.2011.PMID.21214954	0.98	0.70	1.39	0.93	0.96
Shehata2012.ALDHneg_BCR.2015.PMID.25575446	0.82	0.59	1.13	0.22	0.59
Shehata2012.ALDHpos_BCR.2015.PMID.25575446	0.70	0.52	0.95	0.02	0.29
Shehata2012.Basal_BCR.2015.PMID.25575446	0.96	0.74	1.25	0.76	0.92
Shehata2012.Erbb3neg_BCR.2015.PMID.25575446	0.80	0.59	1.09	0.16	0.54
Shehata2012.LumProg_BCR.2015.PMID.25575446	0.79	0.58	1.06	0.11	0.48
Shehata2012.NCL_BCR.2015.PMID.25575446	1.05	0.77	1.41	0.77	0.92
Shehata2012.Stroma_BCR.2015.PMID.25575446	1.03	0.79	1.35	0.82	0.93
Spike2012.aMaSC_BCR.2015.PMID.25575446	1.08	0.83	1.42	0.55	0.80
Spike2012.fMaSC_BCR.2015.PMID.25575446	0.80	0.60	1.06	0.12	0.48
Spike2012.fStr_BCR.2015.PMID.25575446	0.92	0.71	1.20	0.54	0.79
STAT1_BCR.2008.PMID.19272155	0.93	0.69	1.25	0.64	0.85
STAT3.Basal_PNAS.2014.PMID.25139989	0.82	0.59	1.16	0.26	0.65
STAT3.Basal.short_PNAS.2014.PMID.25139989	0.78	0.56	1.08	0.13	0.51
Stroma.FNA.MDACC.1_JCO.2010.PMID.20805453	0.71	0.50	1.01	0.05	0.39
Stroma.FNA.MDACC.2_JCO.2010.PMID.20805453	1.09	0.81	1.45	0.57	0.80
Stromal.Central.Fibrotic.Focus_J.Pathol.2017.PMID.27861902	0.85	0.58	1.25	0.41	0.73
Stromal.Down_Nat.Med.2009.PMID.19648928	0.97	0.76	1.23	0.78	0.93
Stromal.Inflammation_J.Pathol.2017.PMID.27861902	0.76	0.56	1.04	0.09	0.47
Stromal.Signature_Nat.Med.2008.PMID.18438415	0.70	0.52	0.94	0.02	0.29
Stromal.Up_Nat.Med.2009.PMID.19648928	1.00	0.77	1.29	0.99	0.99
SW480.cancer.cells_Immunity.2013.PMID.24138885	1.20	0.84	1.72	0.32	0.70
T.follicular.helper.cell_CellRep.2017.PMID.28052254	0.79	0.59	1.04	0.09	0.48
Tcell.activation_Nature.2020.PMID.31942077	0.91	0.69	1.20	0.50	0.78
Tcell.CD8.Effector.vs.naive.2_Science.2016.PMID27789795	1.13	0.86	1.48	0.39	0.72
Tcell.CD8.Exhausted.vs.antiPDL1.2_Science.2016.PMID27789795	1.15	0.87	1.53	0.31	0.68
Tcell.CD8.Exhausted.vs.naive.2_Science.2016.PMID27789795	1.11	0.84	1.45	0.47	0.76
Tcell.CD8.Memory.vs.naive.1_Science.2016.PMID27789795	0.90	0.66	1.22	0.50	0.77
Tcell.cluster_CCR.2014.PMID.24916698	0.69	0.51	0.94	0.02	0.29
Tcell.EXH.Anti.PDL1.vs.control.treated.exhausted.CD8.Tcell.Metagene.1.Science.2016.PMID.27789795	1.01	0.74	1.39	0.95	0.97
Tcell.EXH.Effector.CD8.T.cell.at.day.8.p.i.Armstrong.vs.Naive.CD8.Tcell.Metagene.1_Science.2016.PMID.27789795	0.87	0.63	1.19	0.39	0.72
Tcell.EXH.Exhausted.CD8.T.cell.vs.Naive.CD8.T.cell.Metagene.1_Science.2016.PMID.27789795	0.94	0.68	1.29	0.69	0.87

Tcell.EXH.Exhausted.CD8.T.cell.vs.Naive.CD8.T.cell.Metagene.3_Science.2016.PMID.27789795	0.95	0.70	1.29	0.72	0.90
Tcell.EXH.Memory.CD8.T.cell.a.vs.Naive.CD8.T.cell.Metagene.1_Science.2016.PMID.27789795	0.90	0.66	1.22	0.50	0.77
Tcell.EXH.Memory.CD8.T.cell.a.vs.Naive.CD8.T.cell.Metagene.2_Science.2016.PMID.27789795	0.92	0.68	1.25	0.61	0.83
Tcell.EXH.Memory.CD8.T.cell.a.vs.Naive.CD8.T.cell.Metagene.3.Science_2016.PMID.27789795	0.88	0.63	1.24	0.47	0.76
Tcell.NK.51gene_Genome.Biol.2013.PMID.23618380	0.70	0.51	0.97	0.03	0.32
Tcell.NK.Metagene_Genome.Biol.2013.PMID.23618380	0.71	0.52	0.97	0.03	0.33
Tcell.RM_Nat_Med.2018.PMID.29942092	0.76	0.54	1.07	0.11	0.48
Tcell.survival.2gene_Nature.2020.PMID.31942077	0.66	0.49	0.89	0.01	0.29
Tcells_CancerImmunolRes.2018.PMID.30266715	0.75	0.54	1.02	0.07	0.44
Tcells_Immunity.2013.PMID.24138885	0.72	0.53	0.98	0.04	0.35
Tcells_TFH_Nat.Methods.2015.PMID.25822800	0.70	0.52	0.95	0.02	0.29
Tcells.CD4.memory.activated_Nat.Methods.2015.PMID.25822800	0.78	0.57	1.05	0.10	0.48
Tcells.CD4.memory.resting_Nat.Methods.2015.PMID.25822800	0.68	0.50	0.92	0.01	0.29
Tcells.CD4.naive_Nat.Methods.2015.PMID.25822800	0.71	0.52	0.95	0.02	0.29
Tcells.CD8_Immunity.2013.PMID.24138885	1.04	0.74	1.46	0.83	0.93
Tcells.CD8_Nat.Methods.2015.PMID.25822800	0.69	0.51	0.94	0.02	0.29
Tcells.CD8.MCP_Nature.2020.PMID.31942075	0.70	0.51	0.95	0.02	0.29
Tcells.Cytotoxic.MCP_Nature.2020.PMID.31942075	0.78	0.57	1.07	0.13	0.50
Tcells.gammadelta_Nat.Methods.2015.PMID.25822800	0.69	0.51	0.93	0.01	0.29
Tcells.helper_Immunity.2013.PMID.24138885	1.11	0.81	1.51	0.52	0.79
Tcells.MCP_Nature.2020.PMID.31942077	0.74	0.54	1.00	0.05	0.39
Tcells.regulatory.2gene_Nature.2020.PMID.31942077	1.19	0.82	1.72	0.36	0.72
Tcells.Tregs_Nat.Methods.2015.PMID.25822800	0.71	0.52	0.96	0.03	0.29
TCGA.BRCA.1198_BASAL_JCI.2020.PMID.32573490	0.81	0.62	1.07	0.14	0.51
TCGA.BRCA.1198_Chromogranin_JCI.2020.PMID.32573490	0.95	0.68	1.33	0.77	0.92
TCGA.BRCA.1198_COLLAGEN11A_JCI.2020.PMID.32573490	1.31	0.97	1.78	0.08	0.47
TCGA.BRCA.1198_EN1_FDZ9_JCI.2020.PMID.32573490	0.96	0.66	1.39	0.83	0.93
TCGA.BRCA.1198_FGFR4_EGF_JCI.2020.PMID.32573490	0.91	0.67	1.23	0.53	0.79
TCGA.BRCA.1198_HISTONES_JCI.2020.PMID.32573490	1.17	0.87	1.57	0.30	0.67
TCGA.BRCA.1198_HOXC11_HOTAIR_SIX1_JCI.2020.PMID.32573490	1.12	0.83	1.50	0.46	0.76
TCGA.BRCA.1198_IL8_CCL_JCI.2020.PMID.32573490	1.10	0.81	1.49	0.53	0.79
TCGA.BRCA.1198_immune_CD19_JCI.2020.PMID.32573490	0.68	0.50	0.94	0.02	0.29
TCGA.BRCA.1198_immune_CD34_TIE1_JCI.2020.PMID.32573490	0.90	0.71	1.15	0.39	0.72
TCGA.BRCA.1198_immune_CD4_CD53_CD84_BTK_JCI.2020.PMID.32573490	0.80	0.59	1.08	0.14	0.52
TCGA.BRCA.1198_immune_CD8_GZMK_JCI.2020.PMID.32573490	0.70	0.52	0.95	0.02	0.29
TCGA.BRCA.1198_immune_CTLA4_CXCL_FOXP3_JCI.2020.PMID.32573490	0.90	0.68	1.19	0.44	0.75
TCGA.BRCA.1198_immune_FOS_JUN_IL6_JCI.2020.PMID.32573490	1.02	0.71	1.48	0.91	0.96
TCGA.BRCA.1198_immune_GIMAP_IL16_JCI.2020.PMID.32573490	0.81	0.60	1.11	0.19	0.57
TCGA.BRCA.1198_immune_HLA_A_F_JCI.2020.PMID.32573490	0.90	0.65	1.26	0.55	0.80
TCGA.BRCA.1198_immune_HLA_D_JCI.2020.PMID.32573490	0.72	0.51	1.00	0.05	0.38
TCGA.BRCA.1198_immune_INTERFERON_JCI.2020.PMID.32573490	1.10	0.81	1.49	0.55	0.80
TCGA.BRCA.1198_IMMUNE1_JCI.2020.PMID.32573490	0.63	0.47	0.85	0.003	0.29
TCGA.BRCA.1198_LUMINAL_JCI.2020.PMID.32573490	1.16	0.78	1.73	0.47	0.76
TCGA.BRCA.1198_MYBL2_APOBEC3B_JCI.2020.PMID.32573490	1.09	0.83	1.42	0.54	0.79
TCGA.BRCA.1198_NORMAL_JCI.2020.PMID.32573490	0.82	0.65	1.04	0.10	0.48
TCGA.BRCA.1198_NORMAL2_JCI.2020.PMID.32573490	0.83	0.65	1.07	0.15	0.53
TCGA.BRCA.1198_PDCHA_MANY_JCI.2020.PMID.32573490	0.89	0.63	1.26	0.52	0.79
TCGA.BRCA.1198_S100A7_8_9_JCI.2020.PMID.32573490	0.90	0.62	1.30	0.57	0.80

TCGA.BRCA.1198_TP63_JCI.2020.PMID.32573490	0.89	0.69	1.14	0.35	0.72
TCGA.BRCA.1198.IMMUNOGLOBULIN_JCI.2020.PMID.32573490	0.57	0.42	0.77	<0.001	0.10
TCGA.CSF1.response_Immunity.2018.PMID.29628290	0.83	0.60	1.13	0.23	0.59
TCGA.IFN.score_Immunity.2018.PMID.29628290	1.15	0.85	1.55	0.36	0.72
TCGA.Liexpression.score_Immunity.2018.PMID.29628290	0.70	0.52	0.94	0.02	0.29
TCGA.Serum.response.up_Immunity.2018.PMID.29628290	1.06	0.79	1.42	0.70	0.88
TCGA.TFH_Immunity.2018.PMID.29628290	0.76	0.53	1.09	0.14	0.51
TCGA.Tgd_Immunity.2018.PMID.29628290	0.86	0.68	1.08	0.19	0.57
TCGA.TGFB.score_Immunity.2018.PMID.29628290	1.36	0.99	1.88	0.06	0.40
Tcm_Immunity.2013.PMID.24138885	0.97	0.71	1.32	0.83	0.93
Tem_Immunity.2013.PMID.24138885	1.08	0.82	1.43	0.58	0.80
TFH_Immunity.2013.PMID.24138885	0.76	0.53	1.09	0.14	0.51
Tgd_Immunity.2013.PMID.24138885	0.86	0.68	1.08	0.19	0.57
Th1_cells_Immunity.2013.PMID.24138885	0.89	0.65	1.24	0.50	0.77
Th17_cells_Immunity.2013.PMID.24138885	0.65	0.48	0.87	0.00	0.29
Th2_cells_Immunity.2013.PMID.24138885	1.20	0.90	1.61	0.22	0.59
TLS.9Gene.Signature_Nature.2020.PMID.31942071	0.79	0.60	1.05	0.11	0.48
TLS.CXCL13.SingleGene_Nature.2020.PMID.31942077	0.95	0.71	1.26	0.71	0.88
TLS.Hallmark.Gene.Signature_Nature.2020.PMID.31942071	0.74	0.54	1.01	0.06	0.40
TLS.Known.Markers_Nature.2020.PMID.31942071	0.73	0.53	0.99	0.05	0.38
TLS.Structure.12chemokine_FrontImmunol.2017.PMID.28713385	0.86	0.63	1.19	0.37	0.72
TLS.tumors.wTLS.and.CD8.vs.CD8alone_Nature.2020.PMID.31942071	0.67	0.49	0.92	0.01	0.29
TNBC.good.prognosis.TNBC.230genes_BCR.2011.PMID.21978456	1.21	0.91	1.62	0.19	0.57
TNBC.good.prognosis.TNBC.26genes_BCR.2011.PMID.21978456	0.80	0.58	1.10	0.18	0.56
TNBC.metastasis.free.survival_PLoS.One.2013.PMID.24349199	1.17	0.88	1.56	0.27	0.65
TNBC.poor.prognosis.TNBC.26genes_BCR.2011.PMID.21978456	1.39	1.04	1.87	0.03	0.29
Translation.Pathway_CancerImmunolRes.2018.PMID.30266715	0.88	0.64	1.21	0.43	0.75
Tumour.hypoxia.causes.DNA.hypermethylation_Nature.2016.PMID.27533040	1.35	0.98	1.85	0.06	0.42
Type.1.T.helper.cell_CellRep.2017.PMID.28052254	0.75	0.53	1.06	0.11	0.48
Type.17.T.helper.cell_CellRep.2017.PMID.28052254	1.00	0.75	1.33	>0.99	>0.99
Type.2.T.helper.cell_CellRep.2017.PMID.28052254	1.30	0.92	1.84	0.14	0.51
Up.Basal.High_Nat.Cell.Biol.2014.PMID.25173976	0.85	0.63	1.16	0.31	0.68
Up.Proliferation_Nat.Cell.Biol.2014.PMID.25173976	1.12	0.84	1.47	0.44	0.75
Upregulated.by.oncogenic.NRAS.basal_Cell.Rep.2016.PMID.26166574	0.89	0.67	1.17	0.40	0.72
Upregulated.upon.NRAS.repression.basal_Cell.Rep.2017.PMID.26166574	1.02	0.76	1.36	0.91	0.96
Vascular.Content_Clin.Exp.Metastasis.2014.PMID.23975155	1.40	0.99	2.00	0.06	0.40
VEGF.13genes_BMC.Med.2009.PMID.19291283	1.09	0.80	1.49	0.59	0.81
Wirapati.Proliferation_BCR.2008.PMID.18662380	1.18	0.89	1.57	0.25	0.62
Wound.Signature_CCR.2009.PMID.19887484	0.93	0.69	1.25	0.62	0.84
X11q13.Amplicon_BMC.Med.Genomics.2011.PMID.21214954	1.21	0.88	1.66	0.23	0.60
X12qMDM4.BMC.Med.Genomics.2011.PMID.21214954	1.27	1.01	1.61	0.04	0.37
X13q14.Amplicon_BMC.Med.Genomics.2011.PMID.21214954	0.89	0.62	1.27	0.52	0.79
X15q25.Amplicon_BMC.Med.Genomics.2011.PMID.21214954	0.74	0.53	1.03	0.07	0.44
X16.13.Amplicon_BMC.Med.Genomics.2011.PMID.21214954	0.92	0.63	1.35	0.68	0.87
X16q23.Amplicon_BMC.Med.Genomics.2011.PMID.21214954	1.11	0.81	1.52	0.53	0.79
X17PP13.Amplicon_BMC.Med.Genomics.2011.PMID.21214954	0.89	0.65	1.21	0.46	0.76
X17q25x.BMC.Med.Genomics.2011.PMID.21214954	1.10	0.78	1.56	0.58	0.80
X19p13.Amplicon_BMC.Med.Genomics.2011.PMID.21214954	0.98	0.65	1.46	0.90	0.95

X1p36.Amplicon_BMC.Med.Genomics.2011.PMID.21214954	0.95	0.66	1.36	0.78	0.93
X3p21.Amplicon_BMC.Med.Genomics.2011.PMID.21214954	0.75	0.55	1.03	0.08	0.46
X4p16.Amplicon_BMC.Med.Genomics.2011.PMID.21214954	1.15	0.83	1.59	0.39	0.72
X5Q_BCRT.2012.PMID.22048815	1.08	0.76	1.53	0.67	0.87
X8p.Amplicon_BMC.Med.Genomics.2011.PMID.21214954	1.19	0.91	1.56	0.20	0.59
X8p22.Amplicon_BMC.Med.Genomics.2011.PMID.21214954	0.87	0.64	1.19	0.39	0.72
XBP1.Signature_Nature.2014.PMID.24670641	1.35	1.02	1.78	0.04	0.35

NeoALTTO					
Signature	HR	95% CI		*P*	adjusted *P*
Activate.Endothelium_Clin.Exp.Metastasis.2014.PMID.23975155	0.79	0.60	1.03	0.08	0.32
Activated.B.cell_CellRep.2017.PMID.28052254	0.87	0.68	1.13	0.31	0.55
Activated.Blood.Neutrophil.Signature_Nat.Cell.Biol.2019.PMID.31263265	0.89	0.72	1.10	0.30	0.53
Activated.Cancer.Cell.Signature_Nat.Cell.Biol.2019.PMID.31263265	1.06	0.81	1.38	0.68	0.82
Activated.CD4.T.cell_CellRep.2017.PMID.28052254	0.75	0.57	0.99	0.04	0.28
Activated.CD8.T.cell_CellRep.2017.PMID.28052254	0.86	0.67	1.10	0.23	0.48
Activated.dendritic.cell_CellRep.2017.PMID.28052254	0.75	0.57	0.98	0.03	0.28
Activated.Lung.MSC.Signature_Nat.Cell.Biol.2019.PMID.31263265	1.29	1.01	1.65	0.04	0.28
Activated.Lung.Neutrophil.Signature_Nat.Cell.Biol.2019.PMID.31263265	0.69	0.54	0.90	0.006	0.18
aDC_Immunity.2013_PMID.24138885.PMID.24138885	0.76	0.59	0.97	0.03	0.28
ADM.S100A10.A110NDGR1.Cluster_BMC.Med.Genomics.2011.PMID.21214954	0.84	0.63	1.11	0.21	0.46
African.and.European.Ancestry.TCGA.Negative_JAMA.Oncol.2017.PMID.28472234	1.10	0.83	1.45	0.50	0.71
African.and.European.Ancestry.TCGA.Positive_JAMA.Oncol.2017.PMID.28472234	0.97	0.76	1.23	0.79	0.90
Age.associated.signature_Genome.Biol.2015.PMID.26343147	1.46	1.15	1.85	0.002	0.13
aMaSC_BCR.2010.PMID.20346151	1.38	1.02	1.86	0.04	0.28
aMaSC.HsEnriched_BCR.2015.PMID.25575446	1.33	1.01	1.77	0.05	0.28
aMaSC.HsEnriched.Refined1_BCR.2015.PMID.25575446	1.41	1.05	1.89	0.02	0.28
aMaSC.Lim09_BCR.2015.PMID.25575446	1.14	0.88	1.47	0.32	0.56
aMaSC.Prat_BCR.2015.PMID.25575446	1.16	0.89	1.53	0.28	0.51
aMaSC.Shehata_BCR.2015.PMID.25575446	1.00	0.79	1.25	0.99	>0.99
aMaSC.Signature_Cell.Stem.Cell.2012.PMID.22305568	1.29	0.98	1.71	0.07	0.31
AMPH.EPIREGULIN.Cluster_BMC.Med.Genomics.2011.PMID.21214954	1.32	1.00	1.74	0.05	0.28
Amplification.50_Genome.Biol.2014.PMID.25164602	0.93	0.74	1.16	0.50	0.72
Amplification.50.better.than._Genome.Biol.2015.PMID.25164602	0.85	0.67	1.09	0.21	0.46
Apocrine.Features_J.Pathol.2017.PMID.27861902	0.84	0.65	1.08	0.18	0.44
aStr.HsEnriched_BCR.2015.PMID.25575446	1.10	0.83	1.45	0.52	0.73
aStr.HsEnriched.Refined1_BCR.2015.PMID.25575446	1.29	0.97	1.72	0.08	0.31
aStr.HsEnriched.Refined2_BCR.2015.PMID.25575446	1.22	0.92	1.62	0.17	0.44
aStr.Lim09_BCR.2015.PMID.25575446	1.12	0.84	1.50	0.44	0.65
aStr.Prat_BCR.2015.PMID.25575446	1.04	0.79	1.38	0.77	0.88
aStr.Shehata_BCR.2015.PMID.25575446	1.01	0.77	1.34	0.92	0.95
BASAL.Cluster_BMC.Med.Genomics.2011.PMID.21214954	1.63	1.23	2.16	0.001	0.11
Bcell.cluster_CCR.2014.PMID.24916698	0.84	0.64	1.11	0.22	0.46
Bcell.IL10.MINUS_Immunol.2014.PMID.25080484	0.86	0.67	1.12	0.26	0.50
Bcell.IL10.PLUS_Immunol.2014.PMID.25080484	0.78	0.60	1.00	0.05	0.28

Bcell.lineage.MCP_Nature.2020.PMID.31942077	0.83	0.64	1.09	0.19	0.44
Bcell.Plasma.52gene_Genome.Biol.2013.PMID.23618380	0.75	0.58	0.97	0.03	0.28
Bcell.Plasma.Metagene_Genome.Biol.2013.PMID.23618380	0.86	0.66	1.12	0.26	0.49
Bcell.Tcell.Cooperation_Cell.2019.PMID.31730857	0.93	0.71	1.21	0.59	0.77
Bcells_CancerImmunolRes.2018.PMID.30266715	0.83	0.63	1.08	0.17	0.43
Bcells_Immunity.2013.PMID.24138885	0.89	0.68	1.15	0.37	0.60
Bcells.Centroblast_JCO.2015.PMID.25800755	0.76	0.58	0.99	0.04	0.28
Bcells.Centrocyte_JCO.2015.PMID.25800755	0.94	0.74	1.20	0.62	0.79
Bcells.Memory_JCO.2015.PMID.25800755	0.86	0.67	1.11	0.24	0.49
Bcells.memory_Nat.Methods.2015.PMID.25822800	0.84	0.65	1.09	0.20	0.45
Bcells.Naive_JCO.2015.PMID.25800755	0.99	0.77	1.27	0.92	0.95
Bcells.naive_Nat.Methods.2015.PMID.25822800	0.84	0.65	1.10	0.20	0.45
Bcells.Plasmablast_JCO.2015.PMID.25800755	0.79	0.61	1.03	0.08	0.31
Blood.vessels_Immunity.2013.PMID.24138885	1.29	0.98	1.71	0.07	0.31
bMYB.Signature_Oncogene.2009.PMID.19043454	0.80	0.61	1.05	0.11	0.36
C3TAG.Responding_CCR.2013.PMID.23780888	1.32	0.97	1.79	0.08	0.31
C3TAG.Untreated_CCR.2013.PMID.23780888	0.71	0.53	0.96	0.03	0.28
CD103.Negative_Cancer.Cell.2014.PMID.25446897	1.06	0.82	1.38	0.65	0.81
CD103.Positive_Cancer.Cell.2014.PMID.25446897	0.94	0.74	1.18	0.59	0.77
CD103.Ratio_Cancer.Cell.2014.PMID.25446897	1.02	0.79	1.32	0.86	0.94
CD274_Single_Gene.Single	0.68	0.52	0.89	0.005	0.18
CD34.CD36.Cluster_BMC.Med.Genomics.PMID.21214954	1.21	0.88	1.66	0.24	0.48
CD44.downregulated.genes_Cancer.Cell.2007.PMID.17349583	0.91	0.70	1.19	0.51	0.72
CD44.upregulated.genes_Cancer.Cell.2007.PMID.17349583	1.06	0.84	1.35	0.63	0.80
CD56bright.natural.killer.cell_CellRep.2017.PMID.28052254	0.85	0.67	1.08	0.18	0.44
CD56dim.natural.killer.cell_CellRep.2017.PMID.28052254	0.78	0.62	0.99	0.04	0.28
CD68.cluster_CCR.2014.PMID.24916698	0.97	0.75	1.24	0.79	0.90
CD8.cluster_CCR.2014.PMID.24916698	0.90	0.70	1.16	0.40	0.63
CDKN2A_Single_Gene.Single	1.24	0.99	1.55	0.06	0.29
Central.memory.CD4.T.cell_CellRep.2017.PMID.28052254	1.20	0.95	1.51	0.13	0.39
Central.memory.CD8.T.cell_CellRep.2017.PMID.28052254	0.81	0.63	1.04	0.10	0.35
CES.Score_CCR.2017.PMID.27903675	1.24	0.86	1.80	0.25	0.49
Chromogramin_BMC.Med.Genomics.2011.PMID.21214954	1.15	0.89	1.48	0.29	0.53
CIN70_Nat.Genet.2006.PMID.16921376	0.80	0.61	1.06	0.12	0.37
Claudin.High_Genome.Biol.2007.PMID.17493263	0.85	0.66	1.11	0.24	0.48
Claudin.Low_Genome.Biol.2007.PMID.17493263	1.16	0.84	1.61	0.36	0.59
Claudin.Low.29_Cancer.Res.2009.PMID.19435916	1.19	0.84	1.68	0.32	0.56
cMYB.Signature_PLoS.One.2010.PMID.20949095	0.90	0.73	1.12	0.36	0.59
CORE.Bcell.signature.Garber_Cell.Mol.Gastroenterol.Hepatol.2017.PMID.28508029	0.86	0.66	1.12	0.27	0.50
CTLA4_Single_Gene.Single	0.79	0.61	1.02	0.08	0.31
Cytolytic.activity_Cell.2015.PMID.25594174	0.89	0.71	1.12	0.32	0.55
Cytotoxic.cells_Immunity.2013.PMID.24138885	0.90	0.71	1.16	0.42	0.64
Day7.Downregulated_Nat.Cell.Biol.2014.PMID.25173976	1.09	0.82	1.45	0.55	0.74
Day7.Upregulated_Nat.Cell.Biol.2014.PMID.25173976	0.98	0.78	1.23	0.85	0.93
DC_Immunity.2013.PMID.24138885	0.90	0.66	1.23	0.51	0.73
DCIS.HGF.down_BCR.2013.PMID.24025166	1.29	1.00	1.66	0.05	0.28
DCIS.HGF.up_BCR.2014.PMID.24025166	1.40	1.09	1.78	0.008	0.18
Delection.50_Genome.Biol.2016.PMID.25164602	1.13	0.84	1.51	0.41	0.64

Delection.50.better.than_Genome.Biol.2017.PMID.25164602	1.18	0.93	1.51	0.17	0.44
Dendritic.cells.activated_Nat.Methods.2015.PMID.25822800	0.84	0.65	1.09	0.19	0.44
Dendritic.cells.resting_Nat.Methods.2015.PMID.25822800	0.83	0.63	1.09	0.18	0.44
Down.Basal.High_Nat.Cell.Biol.2014.PMID.25173976	1.27	0.96	1.69	0.10	0.35
Down.CLOW.High_Nat.Cell.Biol.2014.PMID.25173976	1.32	1.01	1.74	0.05	0.28
Downregulated.upon.NRAS.repression.basal_Cell.Rep.2015.PMID.26166574	0.87	0.69	1.10	0.25	0.49
Ductal.Carcinoma.In.Situ_J.Pathol.2017.PMID.27861902	0.85	0.65	1.11	0.24	0.48
Duke.Module01.acidosis_PNASUSA.2010.PMID.20335537	1.05	0.78	1.41	0.75	0.87
Duke.Module02.akt_PNASUSA.2010.PMID.20335537	0.89	0.72	1.08	0.24	0.48
Duke.Module03.betacatenin_PNASUSA.2010.PMID.20335537	0.83	0.65	1.05	0.12	0.38
Duke.Module04.E2F1_PNASUSA.2010.PMID.20335537	0.75	0.60	0.94	0.01	0.21
Duke.Module05.EGFR_PNASUSA.2010.PMID.20335537	0.90	0.70	1.15	0.39	0.62
Duke.Module06.ER_PNASUSA.2010.PMID.20335537	1.02	0.76	1.37	0.88	0.95
Duke.Module07.glucosedepletion_PNASUSA.2010.PMID.20335537	0.63	0.51	0.78	<0.001	0.008
Duke.Module08.HER2_PNASUSA.2010.PMID.20335537	0.92	0.72	1.18	0.52	0.73
Duke.Module09.hypoxia_PNASUSA.2010.PMID.20335537	0.82	0.64	1.04	0.10	0.35
Duke.Module10.IFNA_PNASUSA.2010.PMID.20335537	1.02	0.80	1.28	0.90	0.95
Duke.Module11.IFNG_PNASUSA.2010.PMID.20335537	0.93	0.73	1.17	0.53	0.73
Duke.Module12.lacticacidosis_PNASUSA.2010.PMID.20335537	1.15	0.91	1.47	0.25	0.49
Duke.Module13.myc_PNASUSA.2010.PMID.20335537	0.94	0.75	1.19	0.61	0.79
Duke.Module14.p53_PNASUSA.2010.PMID.20335537	0.98	0.74	1.29	0.89	0.95
Duke.Module15.p63_PNASUSA.2010.PMID.20335537	0.83	0.64	1.06	0.13	0.39
Duke.Module16.pi3k_PNASUSA.2010.PMID.20335537	0.78	0.63	0.98	0.03	0.28
Duke.Module17.PR_PNASUSA.2010.PMID.20335537	1.23	0.89	1.68	0.21	0.46
Duke.Module18.ras_PNASUSA.2010.PMID.20335537	0.82	0.67	1.00	0.05	0.28
Duke.Module19.src_PNASUSA.2010.PMID.20335537	1.11	0.89	1.39	0.34	0.57
Duke.Module20.STAT3_PNASUSA.2010.PMID.20335537	0.83	0.63	1.10	0.19	0.44
Duke.Module21.TGFB_PNASUSA.2010.PMID.20335537	0.88	0.66	1.16	0.35	0.58
Duke.Module22.TNFA_PNASUSA.2010.PMID.20335537	0.84	0.64	1.09	0.18	0.44
Durvalumab.signature_CCR.2018.PMID.29716923	0.79	0.62	1.01	0.06	0.28
Early.IRS.1_PLoS.One.2016.PMID.26991655	0.80	0.61	1.04	0.09	0.33
Early.IRS.2_PLoS.One.2016.PMID.26991655	1.06	0.83	1.35	0.65	0.81
Early.Relapse.ERPos.33genes_JAMA.2011.PMID.21558518	0.81	0.63	1.04	0.10	0.35
Early.Response.ERNeg.27genes_JAMA.2011.PMID.21558518	1.02	0.81	1.29	0.86	0.93
Effector.memeory.CD4.T.cell_CellRep.2017.PMID.28052254	0.95	0.76	1.18	0.62	0.79
Effector.memeory.CD8.T.cell_CellRep.2017.PMID.28052254	0.86	0.67	1.11	0.25	0.49
EGFR_Single_Gene.Single	1.15	0.90	1.47	0.26	0.49
EMT.down.Taube_PNAS.2010.PMID.20713713	0.99	0.76	1.28	0.93	0.96
EMT.down.Weingberg_PNAS.2010.PMID.20713713	1.40	1.03	1.90	0.03	0.28
EMT.up.Taube_PNAS.2010.PMID.20713713	0.94	0.73	1.20	0.61	0.79
EMT.up.Weinberg_PNAS.2010.PMID.20713713	1.03	0.79	1.36	0.82	0.91
Endothelial.cells.MCP_Nature.2020..PMID.31942077	1.45	1.10	1.92	0.009	0.20
Endothelial.Normal_Angiogenesis.2014.PMID.24257808	0.86	0.65	1.13	0.27	0.50
Endothelial.Tumor_Angiogenesis.2014.PMID.24257808	1.02	0.79	1.30	0.89	0.95
Eosinophil_CellRep.2017.PMID.28052254	0.85	0.68	1.06	0.16	0.41
Eosinophils_Immunity.2013.PMID.24138885	0.85	0.66	1.09	0.19	0.44
Eosinophils_Nat.Methods.2015.PMID.25822800	0.81	0.62	1.05	0.11	0.36
Epithelial.Tubule.Formation_J.Pathol.2017.PMID.27861902	0.92	0.74	1.14	0.43	0.65

ERBB2_Single_Gene.Single	0.89	0.66	1.18	0.41	0.64
ERBB3_Single_Gene.Single	1.22	0.91	1.62	0.18	0.44
ESR1_Single_Gene.Single	1.07	0.75	1.53	0.69	0.83
ESTIMATE.Immune_Nat.Commun.2013.PMID.24113773	0.85	0.66	1.09	0.20	0.45
ESTIMATE.Stromal_Nat.Commun.2013.PMID.24113773	1.02	0.78	1.32	0.91	0.95
Euclidean.Distance.CLOW_BCR.2010.PMID.20813035	1.09	0.81	1.47	0.55	0.75
EXTENDED.Bcell.signature.Garber_Cell.Mol.Gastroenterol.Hepatol.2017.PMID.28508029	0.84	0.67	1.07	0.15	0.41
FGFR4_Single_Gene.Single	1.07	0.83	1.37	0.60	0.78
FGFR4.Induced_JCI.2020.PMID.32573490	0.84	0.68	1.04	0.10	0.35
FGFR4.Repressed_JCI.2020.PMID.32573490	0.98	0.76	1.26	0.88	0.95
Fibrinogen.Cluster_BMC.Med.Genomics.2011.PMID.21214954	1.25	0.98	1.60	0.07	0.31
Fibroblast.Cluster_BMC.Med.Genomics.2011.PMID.21214954	1.17	0.89	1.54	0.25	0.49
Fibroblasts.MCP_Nature.2020.PMID.31942077	1.29	0.99	1.67	0.06	0.29
Fibromatosis_Lab.Invest.2008.PMID.18414401	1.24	0.95	1.62	0.12	0.37
fMaSC.Metab_CellRep.2018.PMID.30089273	1.11	0.86	1.44	0.42	0.65
fMaSC.Metab8_CellRep.2018.PMID.30089273	0.71	0.54	0.92	0.01	0.21
fMaSC.refined1_BCR.2015.PMID.25575446	1.17	0.90	1.54	0.24	0.49
fMasC.Signature_Cell.Stem.Cell.2012.PMID.22305568	1.38	1.05	1.81	0.02	0.26
fMaSC.Signature_CellRep.2018.PMID.30089273	0.84	0.70	1.01	0.06	0.29
FOS.JUN_Cluster_BMC.Med.Genomics.2011.PMID.21214954	1.02	0.83	1.26	0.83	0.92
FOXC1.Hair.Follicles.P30C.LO.vs.WT.Negative_Science.2016.PMID.26912704	1.20	0.93	1.55	0.17	0.43
FOXC1.Hair.Follicles.P30C.LO.vs.WT.Positive_Science.2016.PMID.26912704	1.14	0.89	1.47	0.30	0.53
fSTR.Signature_Cell.Stem.Cell.2012.PMID.22305568	1.23	0.92	1.65	0.16	0.41
Gamma.delta.T.cell_CellRep.2017.PMID.28052254	0.93	0.73	1.18	0.53	0.73
GATA3.induced.genes_JCO.2006.PMID.16505416	1.09	0.83	1.43	0.52	0.73
GATA3.induced.genes_Oncogene.2004.PMID.15361840	0.86	0.69	1.07	0.19	0.44
GDF11.TGFBR3_Nat.Cell.Biol.2014.PMID.24658685	0.99	0.78	1.27	0.94	0.96
Glycolysis_BMC.Med.2009.PMID.19291283	1.01	0.78	1.31	0.93	0.96
GO.DOWN.with.SOX10.OE_Cell.Rep.2015.PMID.26365194	1.17	0.90	1.50	0.24	0.48
GO.UP.with.SOX10.OE_Cell.Rep.2015.PMID.26365194	1.03	0.82	1.31	0.78	0.89
GSEA_BIOCARTA_ALK_PATHWAY.PMID.16199517	1.01	0.80	1.28	0.90	0.95
GSEA_BIOCARTA.AKT.PATHWAY.PMID.16199517	0.86	0.68	1.10	0.23	0.48
GSEA_BIOCARTA.BRCA.ATR.PATHWAY.ATRBRCA.PMID.16199517	0.83	0.65	1.06	0.13	0.38
GSEA_BIOCARTA.CASPASE.PATHWAY.PMID.16199517	0.85	0.67	1.07	0.16	0.42
GSEA_BIOCARTA.CTLA4.PATHWAY.PMID.16199517	0.90	0.70	1.16	0.43	0.65
GSEA_BIOCARTA.IGF1R.PATHWAY.PMID.16199517	0.83	0.65	1.06	0.14	0.39
GSEA_BIOCARTA.MTOR.PATHWAY.PMID.16199517	1.01	0.79	1.30	0.92	0.95
GSEA_BIOCARTA.PTEN.PATHWAY.PMID.16199517	1.06	0.82	1.37	0.64	0.80
GSEA_BIOCARTA.RAS.PATHWAY.PMID.16199517	1.00	0.77	1.30	>0.99	>0.99
GSEA_BIOCARTA.RB.PATHWAY.PMID.16199517	0.79	0.63	0.99	0.04	0.28
GSEA_BIOCARTA.VEGF.PATHWAY.PMID.16199517	0.88	0.69	1.11	0.28	0.52
GSEA_HALLMARK.MYC.TARGETS.V1.PMID.16199517	1.16	0.90	1.50	0.26	0.49
GSEA_HELLER.HDAC.TARGETS.DOWN.PMID.16199517	0.78	0.60	1.01	0.06	0.29
GSEA_NELSON.RESPONSE.TO.ANDROGEN.UP.PMID.16199517	0.79	0.64	0.99	0.04	0.28
GSEA_REACTOME.PD1.SIGNALING.PMID.16199517	0.95	0.74	1.24	0.72	0.85
GSEA_REACTOME.PI3K.CASCADE.PMID.16199517	0.71	0.56	0.90	0.005	0.18
GSEA_RETINOL.METABOLISM.KEGG.PMID.16199517	1.05	0.77	1.43	0.74	0.87
GSEA.GP1_Proliferation.DNA.repair..PUJANA.CHEK2.PCC.NETWORK.PMID.25109877	0.83	0.66	1.05	0.12	0.37

GSEA.GP1_Proliferation.DNA.repair.REACTOME.CELL.CYCLE.MITOTIC.PMID.25109877	0.89	0.69	1.13	0.33	0.56
GSEA.GP10_Fatty.acid.oxidation.CARBOXYLIC.ACID.METABOLIC.PROCESS.PMID.25109877	0.85	0.68	1.07	0.18	0.44
GSEA.GP11_Immune.IFN.PerouLab.PMID.25109877	0.94	0.74	1.20	0.62	0.79
GSEA.GP12_Hypoxia.glycolosis.SEMENZA.HIF1.TARGETS.PMID.25109877	0.88	0.68	1.12	0.30	0.54
GSEA.GP13_Neural.signailng.MODULE100.PMID.25109877	1.35	1.00	1.82	0.05	0.28
GSEA.GP13_Neural.signailng.NERVOUS.SYSTEM.DEVELOPMENT.PMID.25109877	1.32	1.00	1.76	0.05	0.28
GSEA.GP14_Plasma.membrane.cell.cell.signaling.MORF.CNTN1.PMID.25109877	1.04	0.85	1.28	0.69	0.83
GSEA.GP15_EGF.signaling.NAGASHIMA.EGF.SIGNALING.UP.PMID.25109877	1.00	0.81	1.24	0.98	0.98
GSEA.GP16_Protein.kinase.signaling.MAPKs.INTRACELLULAR.SIGNALING.CASCADE.PMID.25109877	0.68	0.54	0.87	0.002	0.13
GSEA.GP16_Protein.kinase.signaling.MAPKs.REGULATION.OF.KINASE.ACTIVITY.PMID.25109877	0.88	0.69	1.13	0.33	0.56
GSEA.GP17_Basal.signaling.SMID.BREAST.CANCER.BASAL.UP.PMID.25109877	1.03	0.76	1.41	0.83	0.92
GSEA.GP18_Vesicle.EPR.MEMBRANE.COAT.PMID.25109877	0.90	0.69	1.19	0.46	0.68
GSEA.GP19_1Q.amplicon.PerouLab.PMID.25109877	0.93	0.72	1.20	0.57	0.76
GSEA.GP2_Immune.Tcell.Bcell.KEGG.HEMATOPOIETIC.CELL.LINEAGE.PMID.25109877	0.84	0.64	1.09	0.19	0.44
GSEA.GP2_Immune.Tcell.Bcell.PerouLab.PMID.25109877	0.86	0.67	1.10	0.22	0.47
GSEA.GP20_TAL1.Leukemia.erythropoiesis.GNF2.TAL1.PMID.25109877	0.99	0.78	1.25	0.94	0.96
GSEA.GP21_Anti.apoptosis.DNA.stability.MORF.BCL2.PMID.25109877	1.18	0.92	1.51	0.19	0.44
GSEA.GP21_Anti.apoptosis.DNA.stability.MORF.MT4.PMID.25109877	1.06	0.85	1.33	0.61	0.78
GSEA.GP21_Anti.apoptosis.DNA.stability.MORF.STK17A.PMID.25109877	1.13	0.88	1.44	0.33	0.57
GSEA.GP22_16Q22.24.amplicon.PerouLab.PMID.25109877	0.95	0.76	1.19	0.66	0.81
GSEA.GP3_Tumo.suppressing.miRNA.targets.GTTTGTT.MIR.495.PMID.25109877	0.79	0.62	0.99	0.04	0.28
GSEA.GP3_Tumor.suppressing.miRNA.targets.DACOSTA.UV.RESPONSE.VIA.ERCC3.DN.PMID.25109877	0.77	0.61	0.97	0.03	0.28
GSEA.GP3_Tumor.suppressing.miRNA.targets.TGCTTTG.MIR.330.PMID.25109877	0.80	0.64	1.01	0.06	0.28
GSEA.GP4_MES.ECM.PerouLab.PMID.25109877	1.07	0.82	1.38	0.62	0.79
GSEA.GP5_MYC.targets.TERT.PerouLab.PMID.25109877	0.90	0.72	1.12	0.35	0.58
GSEA.GP6_Squamous.differentiation.development.RICKMAN.TUMOR.DIFFERENTIATED.WELL.VS.POORLY.DN.PMID.25109	0.83	0.65	1.07	0.15	0.41
GSEA.GP7_Estrogen.signaling.SMID.BREAST.CANCER.BASAL.DN.PMID.25109877	1.21	0.87	1.68	0.26	0.49
GSEA.GP8_FOXO.stemness.MORF.PTPRB.PMID.25109877	1.13	0.89	1.43	0.31	0.55
GSEA.GP8_FOXO.stemness.TTGTTT.VSFOXO4.01.PMID.25109877	0.94	0.75	1.17	0.56	0.75
GSEA.GP9_Cell.cell.adhesion.PerouLab.PMID.25109877	1.20	0.87	1.66	0.27	0.51
HCK_BCR.2008.PMID.19272155	0.86	0.67	1.10	0.24	0.49
HER1.Cluster1_BMC.Genomics.2007.PMID.17663798	1.05	0.83	1.33	0.66	0.81
HER1.Cluster2_BMC.Genomics.2007.PMID.17663798	0.85	0.67	1.09	0.20	0.45
HER1.Cluster3_BMC.Genomics.2007.PMID.17663798	0.82	0.67	1.01	0.06	0.30
HER2.Amplicon.PerouLab_BMC.Med.Genomic.2011.PMID.21214954	1.05	0.83	1.33	0.66	0.81
Histological.Grade_J.Pathol.2017.PMID.27861902	0.82	0.63	1.06	0.12	0.38
HouseKeeping_Genome.Biol.2004.PMID.15287981	1.09	0.85	1.39	0.52	0.73
iDC.Median_Immunity.2013.PMID.24138885	1.03	0.80	1.33	0.83	0.92
IFN.Cluster_BMC.Med.Genomics.2011.PMID.21214954	1.10	0.87	1.40	0.43	0.65
IgG_BCR.2008.PMID.19272155	0.82	0.63	1.06	0.13	0.39
IGG.Cluster_BMC.Med.Genomics.2011.PMID.21214954	0.77	0.59	1.00	0.05	0.28
Immature..B.cell_CellRep.2017.PMID.28052254	0.83	0.63	1.07	0.16	0.41
Immature.dendritic.cell_CellRep.2017.PMID.28052254	1.09	0.85	1.41	0.49	0.71
ImmLandscape_Macro.mono.CSF1.core.response_CCR.2009.PMID.29628290	0.81	0.63	1.05	0.12	0.37
ImmLandscape_Wound.Healing_Immunity.2018.PMID.29628290	0.86	0.68	1.10	0.23	0.48
ImmLandscape.IFN3_Plos.One.2014.PMID.24516633	1.05	0.82	1.34	0.69	0.83
ImmLandscape.IFNG5_Plos.One.2014.PMID.24516633	0.79	0.61	1.01	0.06	0.29
ImmLandscape.lymphocyte.Infil.T.B.PMID.18592372	0.91	0.70	1.16	0.44	0.65

Immune.Hot.CD8.vs.Cold_Nature.2020.PMID.31942071	0.86	0.67	1.11	0.25	0.49
Immune.Perez.14_JCO.2015.PMID.25605861	0.99	0.76	1.28	0.93	0.96
Immune.Perez.87_JCO.2015.PMID.25605861	0.89	0.69	1.15	0.37	0.60
Immune.Suppression_JCI.Insight.2016.PMID.27699256	0.77	0.60	1.00	0.05	0.28
ImmuneActive_Cell.2019.PMID.31730857	0.82	0.63	1.06	0.13	0.39
Immunosuppression.PMID.31942077	1.22	0.96	1.56	0.10	0.35
IMS.Score_CCR.2018.PMID.29921729	0.97	0.75	1.24	0.79	0.90
Induced.in.Bcells_PNAS.2013.PMID.23382184	0.72	0.55	0.93	0.01	0.21
Induced.in.DC_PNAS.2013.PMID.23382184	0.79	0.62	1.01	0.06	0.28
Induced.in.GN_PNAS.2013.PMID.23382184	0.91	0.71	1.16	0.43	0.65
Induced.in.HSC_PNAS.2013.PMID.23382184	0.96	0.77	1.21	0.76	0.88
Induced.in.MOs_PNAS.2013.PMID.23382184	0.95	0.73	1.23	0.70	0.83
Induced.in.NKcells_PNAS.2013.PMID.23382184	1.17	0.90	1.51	0.25	0.49
Induced.in.Tcells_PNAS.2013.PMID.23382184	0.96	0.75	1.22	0.72	0.85
Inflammatory.breast.cancer.491genes_CCR.2013.PMID.23396049	0.83	0.64	1.08	0.17	0.43
Inflammatory.breast.cancer.79genes_CCR.2013.PMID.23396049	0.81	0.63	1.05	0.11	0.36
Inflammatory.breast.cancer.expressed.noIBC_79genes_CCR.2013.PMID.23396049	0.79	0.63	1.00	0.05	0.28
Inflammatory.breast.cancer.expressed.noIBC.491genes_CCR.2013.PMID.23396049	0.89	0.70	1.13	0.32	0.56
Influenza.11genes.Metasignature_Immunity.2015.PMID.26682989	0.99	0.77	1.26	0.91	0.95
Interferon_BCR.2008.PMID.19272155	1.12	0.88	1.43	0.36	0.59
Interferon.Pathway_CancerImmunolRes.2018.PMID.30266715	1.05	0.83	1.33	0.67	0.82
JUND.KRT5_Nat.Cell.Biol.2014.PMID.24658685	1.21	0.98	1.51	0.08	0.31
Keller2012.CD10.Adam_BCR.2015.PMID.25575446	1.14	0.85	1.51	0.38	0.61
KRAS.amplicon_Genome.Biology.2007.PMID.17493263	0.86	0.68	1.09	0.21	0.46
Late.IRS.1_PLoS.One.2016.PMID.26991655	0.87	0.67	1.13	0.28	0.52
Late.IRS.2_PLoS.One.2016.PMID.26991655	1.15	0.91	1.45	0.25	0.49
LCK_BCR.2008.PMID.19272155	0.88	0.68	1.14	0.35	0.58
Lim2009.LumProg.Adam_BCR.2015.PMID.25575446	1.03	0.77	1.37	0.84	0.92
Lim2009.MaSC.Adam_BCR.2015.PMID.25575446	1.30	1.00	1.69	0.05	0.28
Lim2009.MatureLum.Adam_BCR.2015.PMID.25575446	1.13	0.82	1.57	0.46	0.68
Lim2009.Stroma.Adam_BCR.2015.PMID.25575446	1.24	0.93	1.66	0.14	0.40
Lim2010.LumProg.Adam_BCR.2015.PMID.25575446	1.00	0.78	1.28	>0.99	>0.99
Lim2010.MaSC.Adam_BCR.2015.PMID.25575446	1.44	1.09	1.92	0.01	0.21
Lim2010.MatureLum.Adam_BCR.2015.PMID.25575446	1.19	0.94	1.50	0.15	0.41
Lim2010.Stroma.Adam_BCR.2015.PMID.25575446	1.16	0.87	1.54	0.30	0.54
Lobular.Carcinoma.In.Situ_J.Pathol.2017.PMID.27861902	1.44	1.13	1.85	0.004	0.17
LOBULAR.TCGA.SIGNATURE.ImmuneCell.2015.PMID.26451490	0.77	0.59	1.01	0.06	0.28
LOBULAR.TCGA.SIGNATURE.Reactive_Cell.2015.PMID.26451490	1.48	1.12	1.96	0.005	0.18
LOBULAR.TCGA.SUBTYPE.Immune_Cell.2015.PMID.26451490	1.08	0.78	1.50	0.64	0.80
LOBULAR.TCGA.SUBTYPE.Proliferative_Cell.2015.PMID.26451490	1.29	0.95	1.74	0.10	0.35
LOBULAR.TCGA.SUBTYPE.Reactive_Cell.2015.PMID.26451490	1.34	1.03	1.73	0.03	0.28
LTS.score_JCI.2020.PMID.32573490	1.55	1.18	2.03	0.002	0.13
Luminal_Progenitor_Up_Nat.Med.2009.PMID.19648928	1.09	0.81	1.47	0.55	0.74
Luminal.cluster_BMC.Med.Genomics.2011.PMID.21214954	0.94	0.70	1.26	0.70	0.83
Luminal.Progenitor_BCR.2010.PMID.20346151	1.13	0.83	1.52	0.44	0.65
Luminal.Progenitor.Down_Nat.Med.2009.PMID.19648928	1.19	0.91	1.57	0.21	0.46
LumProg.HsEnriched_BCR.2015.PMID.25575446	0.95	0.71	1.27	0.71	0.84
LumProg.HsEnriched.Refined1_BCR.2015.PMID.25575446	1.13	0.84	1.51	0.43	0.65

LumProg.Lim09_BCR.2015.PMID.25575446	0.92	0.71	1.20	0.55	0.74
LumProg.Prat_BCR.2015.PMID.25575446	1.02	0.77	1.34	0.91	0.95
LumProg.Shehata_BCR.2015.PMID.25575446	0.89	0.71	1.10	0.28	0.52
Lums.HER2E.DOWN.metastatic.signature_JCI.2020.PMID.32573490	1.42	1.10	1.84	0.008	0.18
Lums.HER2E.UP.metastatic.signature_JCI.2020.PMID.32573490	0.99	0.78	1.25	0.91	0.95
Lung.WNT_Cancer.Res.2009.PMID.19549913	0.93	0.73	1.17	0.52	0.73
Lymph.vessels_Immunity.2013.PMID.24138885	0.96	0.75	1.24	0.76	0.88
Lymphovascular.Invasion_J.Pathol.2017.PMID.27861902	0.95	0.73	1.23	0.70	0.83
M.D.Metagene_Genome.Biol.2013.PMID.23618380	0.94	0.73	1.20	0.61	0.78
M2.Macrophage_Blood.2006.PMID.16556895	0.97	0.75	1.27	0.83	0.92
Macrophage_CellRep.2017.PMID.28052254	1.06	0.82	1.38	0.65	0.81
Macrophages_CancerImmunolRes.2018.PMID.30266715	0.82	0.64	1.04	0.10	0.36
Macrophages_Immunity.2013.PMID.24138885	0.80	0.62	1.04	0.09	0.34
Macrophages.M0_Nat.Methods.2015.PMID.25822800	0.79	0.60	1.03	0.09	0.32
Macrophages.M1_Nat.Methods.2015.PMID.25822800	0.78	0.60	0.99	0.05	0.28
Macrophages.M2_Nat.Methods.2015.PMID.25822800	0.82	0.62	1.08	0.16	0.42
MacTh1.cluster_CCR.2014.PMID.24916698	0.84	0.64	1.09	0.18	0.44
MammaPrint_Nature.2002.PMID.11823860	1.30	0.97	1.74	0.08	0.32
MAPK.pathway.activation_NPJ.Precis.Oncol.2018.PMID.29872725	1.21	0.93	1.57	0.16	0.41
MASC.Down_Nat.Med.2009.PMID.19648928	0.98	0.77	1.24	0.86	0.93
MASC.Up_Nat.Med.2009.PMID.19648928	1.42	1.05	1.92	0.02	0.28
Mast.cell_CellRep.2017.PMID.28052254	0.97	0.74	1.27	0.80	0.91
Mast.cells_Immunity.2013.PMID.24138885	1.21	0.92	1.58	0.18	0.44
Mast.cells.activated_Nat.Methods.2015.PMID.25822800	1.09	0.84	1.41	0.53	0.73
Mast.cells.resting_Nat.Methods.2015.PMID.25822800	1.09	0.83	1.44	0.54	0.74
Mature.luminal_BCR.2010.PMID.20346151	1.12	0.83	1.51	0.47	0.68
Mature.Luminal.Down_Nat.Med.2009.PMID.19648928	1.16	0.86	1.56	0.33	0.56
Mature.LuminaUp_Nat.Med.2009.PMID.19648928	0.99	0.73	1.34	0.94	0.96
MatureLum.HsEnriched_BCR.2015.PMID.25575446	0.92	0.68	1.24	0.57	0.76
MatureLum.HsEnriched.Refined1_BCR.2015.PMID.25575446	0.97	0.71	1.31	0.84	0.92
MatureLum.Lim09_BCR.2015.PMID.25575446	0.88	0.66	1.17	0.37	0.59
MatureLum.Prat_BCR.2015.PMID.25575446	0.83	0.64	1.09	0.18	0.44
MatureLum.Shehata_BCR.2015.PMID.25575446	0.94	0.70	1.25	0.67	0.81
MBasal.Cluster_BMC.Med.Genomics.2011.PMID.21214954	1.77	1.28	2.44	0.001	0.11
MCD3.CD8_BMC.Med.Genomics.2011.PMID.21214954	0.89	0.70	1.15	0.38	0.61
MCF7.E2.induced.genes_JCO.2006.PMID.16505416	0.83	0.65	1.05	0.12	0.37
MCF7.E2.repressed.genes_JCO.2006.PMID.16505416	0.75	0.57	0.99	0.04	0.28
MDSC_CellRep.2017.PMID.28052254	0.84	0.65	1.09	0.20	0.44
MDSC.Granulocytic_Leukoc.Biol.2012.PMID.21954284	0.94	0.75	1.18	0.60	0.78
MDSC.Neutrophi_Leukoc.Biol.2012.PMID.21954284	0.74	0.57	0.97	0.03	0.28
MDSC.tumor_J.Immunol.2012.PMID.23152559	0.78	0.61	1.01	0.06	0.28
MDSC.tumor.MO_J.Immunol.2012.PMID.23152559	0.89	0.69	1.14	0.36	0.59
MECM_BMC.Med.Genomics.2011.PMID.21214954	1.37	1.02	1.86	0.04	0.28
Memory.B.cell_CellRep.2017.PMID.28052254	1.24	0.95	1.62	0.12	0.37
MET.DOWN.RNAseq.Significant.Genes_JCI.2018.PMID.29480819	0.88	0.72	1.06	0.18	0.44
MET.DOWN.Significant.Genes.Low.Basal.1_JCI.2018.PMID.29480819	1.09	0.84	1.43	0.52	0.73
MET.DOWN.Significant.Genes.Low.Basal.2_JCI.2018.PMID.29480819	0.90	0.72	1.13	0.38	0.61
MET.UP.RNAseq.Significant.Genes_JCI.2018.PMID.29480819	1.15	0.91	1.46	0.23	0.48

MET.UP.Significant.Genes.HIGH.BASALS.Genes_JCI.2018.PMID.29480819	0.90	0.70	1.16	0.41	0.64
Metaplastic.Up_CanRes.2009.PMID.19435916	1.03	0.78	1.36	0.85	0.93
Metastasis.predictor.TNBC_BCR.2010.PMID.20946665	0.72	0.57	0.90	0.004	0.17
MFGFR2_BMC.Med.Genomics.2011.PMID.21214954	0.98	0.76	1.27	0.90	0.95
MHC.Forero.11_Cancer.Immunol.Res.2016.PMID.26980599	0.86	0.65	1.12	0.26	0.50
MHC.Forero.24_Cancer.Immunol.Res.2016.PMID.26980599	0.99	0.80	1.24	0.96	0.97
MHC.I_BCR.2008.PMID.19272155	1.03	0.81	1.30	0.83	0.92
MHC.II_BCR.2008.PMID.19272155	0.79	0.62	1.01	0.06	0.29
MHCI.coreGenes_Nat.Commun.2017.PMID29170503	0.91	0.71	1.16	0.43	0.65
MIR200c.Induced_ONCO.2015.PMID.25746005	1.16	0.91	1.48	0.24	0.49
MIR200c.Repressed_ONCO.2015.PMID.25746005	1.12	0.85	1.46	0.43	0.65
miRNA.138.signature_Cancer.Res.2014.PMID.25339353	0.82	0.65	1.03	0.09	0.34
MITO1_BMC.Med.Genomics.2011.PMID.21214954	1.00	0.82	1.22	0.98	0.98
MITO2_BMC.Med.Genomics.2011.PMID.21214954	0.86	0.71	1.03	0.10	0.36
Mitotic.Count_J.Pathol.2017.PMID.27861902	1.03	0.81	1.31	0.82	0.91
MK14.K17_BMC.Med.Genomics.2011.PMID.21214954	1.31	1.00	1.73	0.05	0.28
MKRAS.amplicon_BMC.Med.Genomics.2011.PMID.21214954	0.78	0.62	0.97	0.03	0.28
MM.BRCAwnt.1pFDR.UP_Genome.Biology.2007.PMID.17493263	0.92	0.73	1.16	0.49	0.71
MM.C3Tag.1pFDR.UP_Genome.Biology.2007.PMID.17493263	0.97	0.75	1.24	0.79	0.90
MM.C3Tag.2012_Genome.Biol.2013.PMID.24220145	0.81	0.62	1.06	0.13	0.39
MM.Class3_Genome.Biol.2013.PMID.24220145	0.97	0.76	1.25	0.81	0.91
MM.Class8_Genome.Biol.2013.PMID.24220145	1.23	0.95	1.59	0.11	0.37
MM.Claudinlow_Genome.Biol.2013.PMID.24220145	1.08	0.82	1.42	0.58	0.77
MM.DMBAwnt.1pFDR.UP.Genome.Biology.2007.PMID.17493263	1.46	1.12	1.92	0.006	0.18
MM.Erbb2.like_Genome.Biol.2013.PMID.24220145	1.19	0.92	1.54	0.18	0.44
MM.Myc.2012_Genome.Biol.2013.PMID.24220145	0.91	0.73	1.14	0.42	0.65
MM.Myoepithelioma.like_Genome.Biol.2013.PMID.24220145	1.42	1.08	1.87	0.01	0.21
MM.Neu.2012_Genome.Biol.2013.PMID.24220145	1.38	1.05	1.81	0.02	0.28
MM.NeuPyMT.1pFDR.UP_Genome.Biology.2007.PMID.17493263	0.89	0.73	1.08	0.23	0.48
MM.Normal.1pFDR.UP_Genome.Biology.2007.PMID.17493263	1.12	0.81	1.54	0.50	0.71
MM.Normal.like_Genome.Biol.2013.PMID.24220145	1.13	0.80	1.59	0.49	0.71
MM.p53null.1pFDR.UP_Genome.Biology.2007.PMID.17493263	0.94	0.74	1.20	0.64	0.81
MM.p53null.Basal_Genome.Biol.2013.PMID.24220145	1.32	1.01	1.73	0.04	0.28
MM.p53null.Luminal_Genome.Biol.2013.PMID.24220145	0.83	0.67	1.03	0.09	0.33
MM.Potluck_1pFDR_UP_Genome.Biology.2007_PMID.17493263.PMID.24220145	0.94	0.72	1.23	0.65	0.81
MM.PyMT.2012_Genome.Biol.2013.PMID.24220145	1.07	0.86	1.34	0.53	0.73
MM.Squamous.like_Genome.Biol.2013.PMID.24220145	1.18	0.92	1.52	0.20	0.44
MM.Stat1_Genome.Biol.2013.PMID.24220145	1.02	0.80	1.30	0.88	0.95
MM.WapINT3.1pFDR.UP_Genome.Biology.2007.PMID.17493263	1.03	0.81	1.31	0.81	0.91
MM.WapINT3.2012_Genome.Biol.2013.PMID.24220145	1.12	0.88	1.43	0.34	0.58
MM.WAPTag.1pFDR.UP_Genome.Biology.2007.PMID.17493263	0.88	0.68	1.15	0.36	0.59
MM.Wnt1.Early_Genome.Biol.2013.PMID.24220145	1.37	1.03	1.83	0.03	0.28
MM.Wnt1.Late_Genome.Biol.2013.PMID.24220145	1.41	1.08	1.85	0.01	0.21
Mmyosin_BMC.Med.Genomics.2011.PMID.21214954	1.03	0.80	1.33	0.80	0.91
MNADH_CYTochrome_BMC.Med.Genomics.2011.PMID.21214954	0.85	0.70	1.03	0.09	0.33
MNB1_BMC.Med.Genomics.2011.PMID.21214954	0.83	0.66	1.05	0.12	0.37
MNB2_BMC.Med.Genomics.2011.PMID.21214954	1.11	0.77	1.60	0.58	0.76
MNB3_BMC.Med.Genomics.2011.PMID.21214954	1.05	0.87	1.26	0.64	0.80

MNOtch4_BMC.Med.Genomics.2011.PMID.21214954	1.54	1.19	1.99	0.001	0.11
Monocyte_CellRep.2017.PMID.28052254	0.84	0.66	1.06	0.14	0.40
Monocyte..DC.25gene_Genome.Biol.2013.PMID.23618380	0.85	0.66	1.09	0.20	0.45
Monocytes_CancerImmunolRes.2018.PMID.30266715	0.89	0.70	1.13	0.34	0.57
Monocytes_Nat.Methods.2015.PMID.25822800	0.77	0.60	0.99	0.05	0.28
Monocytic.lineage.MCP_Nature.2020.PMID.31942075	0.74	0.56	0.98	0.04	0.28
MProliferation_BMC.Med.Genomics.2011.PMID.21214954	0.77	0.59	1.01	0.06	0.28
MProtocadherin_BMC.Med.Genomics.2011.PMID.21214954	0.94	0.72	1.21	0.61	0.79
MPYMT_NEU_Cluster_BMC.Med.Genomics.2011.PMID.21214954	0.90	0.71	1.14	0.38	0.61
MRibosomal_BMC.Med.Genomics.2011.PMID.21214954	1.24	1.00	1.53	0.05	0.28
MS.CD44.DOWN_PNAS.2009.PMID.19666588	1.02	0.78	1.33	0.89	0.95
MS.CD44.UP_PNAS.2009.PMID.19666588	1.00	0.78	1.28	0.99	>0.99
MSquamous_BMC.Med.Genomics.2011.PMID.21214954	1.19	0.91	1.56	0.19	0.44
Murat.G07_JCO.2008.PMID.18565887	1.24	0.93	1.64	0.14	0.39
Murat.G18_JCO.2008.PMID.18565887	1.13	0.87	1.47	0.34	0.58
Murat.G24_JCO.2008.PMID.18565887	0.75	0.58	0.97	0.03	0.28
MVEGFC_BMC.Med.Genomics.2011.PMID.21214954	1.20	0.92	1.57	0.19	0.44
Myeloid.cell.chemotaxis.1gene_Nature.2020.PMID.31942077	0.90	0.72	1.13	0.38	0.61
Myeloid.dendritic.cells.MCP_Nature.2020.PMID.31942077	0.87	0.69	1.09	0.22	0.47
Natural.killer.cell_CellRep.2017.PMID.28052254	1.03	0.82	1.30	0.78	0.89
Natural.killer.T.cell_CellRep.2017.PMID.28052254	0.95	0.73	1.23	0.68	0.82
Necrosis_J.Pathol.2017.PMID.27861902	0.79	0.63	0.98	0.03	0.28
Neutrophil_CellRep.2017.PMID.28052254	1.12	0.85	1.47	0.42	0.65
Neutrophils_CancerImmunolRes.2018.PMID.30266715	1.00	0.78	1.29	0.98	0.98
Neutrophils_Immunity.2013.PMID.24138885	0.91	0.69	1.21	0.53	0.73
Neutrophils_Nat.Methods.2015.PMID.25822800	0.75	0.58	0.99	0.04	0.28
Neutrophils.MCP_Nature.2020.PMID.31942077	1.04	0.78	1.38	0.79	0.90
NK_Immunity.2013.PMID.24138885	1.09	0.85	1.41	0.49	0.71
NK.activated_Nat.Methods.2015.PMID.25822800	0.88	0.68	1.13	0.32	0.56
NK.CD56bright_Immunity.2013.PMID.24138885	1.15	0.92	1.43	0.22	0.47
NK.CD56dim_Immunity.2013.PMID.24138885	0.78	0.60	1.03	0.08	0.31
NK.resting_Nat.Methods.2015.PMID.25822800	0.90	0.71	1.15	0.42	0.64
NKcells_CancerImmunolRes.2018.PMID.30266715	0.83	0.64	1.06	0.13	0.39
NKcells.MCP_Nature.2020.PMID.31942077	0.92	0.71	1.20	0.54	0.74
No.Response.Immunotherapy.TLS.Melanoma_Nature.2020.PMID.31942075	1.28	0.98	1.67	0.07	0.31
Normal.mucosa_Immunity.2013.PMID.24138885	1.26	0.97	1.65	0.08	0.32
Nuclear.Pleomorphism_J.Pathol.2017.PMID.27861902	0.98	0.77	1.26	0.89	0.95
Oncotype_NEJM.2004.PMID.15591335	0.94	0.69	1.27	0.67	0.81
P53.ERPos.MDACC_CCR.2011.PMID.21248301	0.79	0.60	1.05	0.11	0.36
Parity.signature.251genes_BCR.2014.PMID.25005139	0.80	0.63	1.03	0.08	0.32
Parity.signature.40genes_BCR.2014.PMID.25005139	0.79	0.62	1.01	0.06	0.29
PARPi.Resistance_BCRT_2012.PMID.22875744	0.66	0.51	0.87	0.003	0.15
PARPi.Sensitivity_BCRT_2012.PMID.22875744	0.88	0.67	1.15	0.34	0.57
PARPi.Sensitivity.MDACC_NPJ.Syst.Biol.Appl.2017.PMID.28649435	0.84	0.65	1.09	0.19	0.44
PARPi.Sensitivity.Negative_Sci.Adv.2017.PMID.28439535	1.25	0.97	1.60	0.08	0.32
PARPi.Sensitivity.Positive_Sci.Adv.2017.PMID.28439535	0.87	0.67	1.12	0.28	0.51
Pcorr.Breast2Lung.LM2.Correlation_Nature.2005.PMID.16049480	1.10	0.83	1.46	0.49	0.71
Pcorr.Breast2Lung.Parental.Correlation_Nature.2005.PMID.16049480	0.93	0.70	1.23	0.60	0.78

Pcorr.dasatinib.resistant_Cancer.Res.2007.PMID.17332353	1.07	0.80	1.41	0.65	0.81
Pcorr.dasatinib.sensitive_Cancer.Res.2007.PMID.17332353	0.96	0.72	1.27	0.77	0.88
Pcorr.Hypoxia.High.Correlation_PLoS.Med.2006.PMID.16417408	0.79	0.61	1.01	0.06	0.29
Pcorr.Hypoxia.Low.Correlation_PLoS.Med.2006.PMID.16417408	1.30	1.01	1.68	0.05	0.28
Pcorr.IGS_Invasiveness_NJEM.2007.PMID.17229949	0.76	0.58	1.00	0.05	0.28
Pcorr.wound.response.activated_PNAS.2005.PMID.15701700	0.92	0.71	1.18	0.50	0.71
pCR.predictor.ERNeg.55genes_JAMA.2011.PMID.21558518	0.95	0.75	1.19	0.66	0.81
pCR.predictor.ERPos.39genes_JAMA.2011.PMID.21558518	0.77	0.59	1.00	0.05	0.28
PDCD1_Single_Gene.Single	0.87	0.70	1.08	0.21	0.46
Pfefferle2012.LumProg_BCR.2015.PMID.25575446	1.03	0.77	1.38	0.83	0.92
Pfefferle2012.MaSC_BCR.2015.PMID.25575446	1.33	0.99	1.78	0.06	0.29
Pfefferle2012.MatureLum_BCR.2015.PMID.25575446	1.01	0.75	1.36	0.94	0.96
Pfefferle2012.Stroma_BCR.2015.PMID.25575446	1.24	0.93	1.67	0.15	0.41
PGR_Single_Gene.Single	1.20	0.89	1.62	0.23	0.48
PI3Ki.Down_CancerCell.2017.PMID.28528867	0.80	0.65	0.98	0.03	0.28
PI3Ki.Up_CancerCell.2017.PMID.28528867	0.85	0.67	1.08	0.18	0.44
PIK3CA.Pathway_Ann.Oncol.2017.PMID.28177460	0.83	0.66	1.06	0.14	0.39
PIK3CAmt.signature_Cancer.Res.2012.PMID.22552288	0.81	0.63	1.03	0.09	0.33
Plasma.cells_Nat.Methods.2015.PMID.25822800	0.83	0.63	1.08	0.16	0.43
PlasmaCells_CancerImmunolRes.2018.PMID.30266715	0.80	0.62	1.03	0.08	0.32
Plasmacytoid.dendritic.cell_CellRep.2017.PMID.28052254	1.06	0.82	1.36	0.66	0.81
PR.Isoform.Ratio.Up.in.PRA.H_JNCI.2017.PMID.28376177	1.27	0.97	1.67	0.08	0.32
PR.Isoform.Ratio.Up.in.PRB.H_JNCI.2017.PMID.28376177	0.98	0.74	1.30	0.90	0.95
Proliferation.Cluster_BMC.Med.Genomics.2011.PMID.21214954	0.79	0.60	1.04	0.10	0.35
Proliferation.Metagene_Genome.Biol.2013.PMID.23618380	0.78	0.59	1.02	0.07	0.31
Proliferation.score.PAM50_JCO.2009.PMID.19204204	0.73	0.55	0.97	0.03	0.28
ProliferationPathway_CancerImmunolRes.2018.PMID.30266715	0.76	0.58	0.99	0.05	0.28
Prosigna.Proliferation.18_BMC.Med.Genomics.2015.PMID.26297356	0.80	0.61	1.05	0.11	0.37
Race.LuminalA.MRE.score_BCRT.2015.PMID.26109344	0.99	0.77	1.28	0.96	0.97
Radiation.induced.genes_Radoat.Res.2014.PMID.24527691	1.12	0.85	1.47	0.43	0.65
RB.LOH_BCR.2008.PMID.18782450	0.81	0.62	1.05	0.11	0.37
RB.LOSS_JCI.2007.PMID.17160137	0.81	0.62	1.06	0.12	0.37
Regulatory.T.cell_CellRep.2017.PMID.28052254	0.84	0.66	1.07	0.15	0.41
Replication.Stress.Down.set_Cell.Rep.2018.PMID.29768207	0.95	0.76	1.19	0.64	0.81
Replication.Stress.Model_Cell.Rep.2018_PMID.29768207.PMID.29768207	1.03	0.80	1.34	0.82	0.91
Replication.Stress.Neg_Cell.Rep.2018_PMID.29768207.PMID.29768207	0.99	0.79	1.25	0.95	0.97
Replication.Stress.Pos_Cell.Rep.2018_PMID.29768207.PMID.29768207	1.30	1.01	1.68	0.04	0.28
Replication.Stress.Up_Set_Cell.Rep.2018_PMID.29768207.PMID.29768207	1.26	0.98	1.63	0.07	0.31
Residual.disease.predictor.ERNeg.54genes_JAMA.2011.PMID.21558518	1.23	0.97	1.55	0.08	0.32
Residual.disease.predictor.ERPos.73genes_JAMA.2011.PMID.21558518	0.75	0.60	0.94	0.01	0.21
Response.Immunotherapy.MCP.TLS.Melanoma_Nature.2020.PMID.31942075	0.78	0.60	1.03	0.08	0.32
Response.Immunotherapy.signature_Science.2018.PMID.30309915	0.85	0.66	1.09	0.19	0.44
Response.Neo.Chemo_common_CCR.2014.PMID.25047707	0.73	0.56	0.95	0.02	0.24
Response.Neo.Chemo_ERNeg_CCR.2014.PMID.25047707	0.88	0.70	1.12	0.31	0.55
Response.Neo.Chemo_ERPos_CCR.2014.PMID.25047707	0.93	0.73	1.19	0.57	0.76
RHOA.pathway_Ann.Oncol.2017.PMID.28177460	1.13	0.89	1.43	0.33	0.57
Ribosomal.Cluster_BMC.Med.Genomics.2011.PMID.21214954	1.24	1.00	1.54	0.05	0.28
ROR.subtype.PAM50_JCO.2009.PMID.19204204	0.67	0.49	0.92	0.01	0.21

ROR.subtype.proliferation.PAM50_JCO.2009.PMID.19204204	0.70	0.53	0.94	0.02	0.24
RSS.Score_CCR.2018.PMID.29921729	0.87	0.68	1.11	0.26	0.49
S100A9.A8_BMC.Med.Genomics.2011.PMID.21214954	0.80	0.60	1.08	0.14	0.40
Scorr.EMAT1.Correlation_BCR.2020.PMID.32641077	0.92	0.71	1.21	0.56	0.75
Scorr.EMAT2.Correlation_BCR.2020.PMID.32641077	1.50	1.15	1.97	0.003	0.17
Scorr.EMAT3.Correlation_BCR.2020.PMID.32641077	0.97	0.74	1.27	0.84	0.92
Scorr.EMAT4.Correlation_BCR.2020.PMID.32641077	0.93	0.68	1.28	0.66	0.81
Scorr.IE.Correlation_JCO.2006.PMID.16505416	1.48	1.11	1.97	0.007	0.18
Scorr.IIE.Correlation_JCO.2006.PMID.16505416	0.71	0.53	0.94	0.02	0.24
Scorr.PAM50.Basal_JCO.2009.PMID.19204204	1.02	0.72	1.45	0.91	0.95
Scorr.PAM50.Her2_JCO.2009.PMID.19204204	0.71	0.53	0.96	0.03	0.28
Scorr.PAM50.LumA_JCO.2009.PMID.19204204	1.41	1.00	1.99	0.05	0.28
Scorr.PAM50.LumB_JCO.2009.PMID.19204204	0.73	0.56	0.94	0.01	0.21
Scorr.PAM50.Normal_JCO.2009.PMID.19204204	1.43	1.10	1.86	0.007	0.18
Scorr.S329.L_Br.J.Cancer.2008.PMID.18382427	0.94	0.73	1.21	0.62	0.79
Scorr.S329.R_Br.J.Cancer.2008.PMID.18382427	1.04	0.80	1.34	0.78	0.89
Secretoglobin_BMC.Med.Genomics.2011.PMID.21214954	1.09	0.85	1.40	0.50	0.71
Shehata2012.ALDHneg_BCR.2015.PMID.25575446	1.15	0.89	1.47	0.28	0.52
Shehata2012.ALDHpos_BCR.2015.PMID.25575446	0.96	0.70	1.30	0.78	0.89
Shehata2012.Basal_BCR.2015.PMID.25575446	1.33	1.00	1.76	0.05	0.28
Shehata2012.Erbb3neg_BCR.2015.PMID.25575446	1.06	0.84	1.34	0.64	0.80
Shehata2012.LumProg_BCR.2015.PMID.25575446	1.05	0.79	1.38	0.75	0.87
Shehata2012.NCL_BCR.2015.PMID.25575446	1.08	0.79	1.47	0.65	0.81
Shehata2012.Stroma_BCR.2015.PMID.25575446	1.13	0.85	1.50	0.39	0.62
Spike2012.aMaSC_BCR.2015.PMID.25575446	1.20	0.92	1.56	0.18	0.44
Spike2012.fMaSC_BCR.2015.PMID.25575446	1.30	1.00	1.70	0.05	0.28
Spike2012.fStr_BCR.2015.PMID.25575446	1.05	0.80	1.37	0.73	0.86
STAT1_BCR.2008.PMID.19272155	0.84	0.65	1.08	0.17	0.44
STAT3.Basal_PNAS.2014.PMID.25139989	0.85	0.66	1.09	0.19	0.44
STAT3.Basal.short_PNAS.2014.PMID.25139989	0.87	0.67	1.12	0.27	0.50
Stroma.FNA.MDACC.1_JCO.2010.PMID.20805453	0.89	0.69	1.14	0.35	0.58
Stroma.FNA.MDACC.2_JCO.2010.PMID.20805453	1.19	0.91	1.56	0.21	0.46
Stromal.Central.Fibrotic.Focus_J.Pathol.2017.PMID.27861902	0.93	0.72	1.19	0.55	0.74
Stromal.Down_Nat.Med.2009.PMID.19648928	1.23	0.90	1.68	0.19	0.44
Stromal.Inflammation_J.Pathol.2017.PMID.27861902	0.79	0.60	1.03	0.08	0.31
Stromal.Signature_Nat.Med.2008.PMID.18438415	0.94	0.73	1.21	0.65	0.81
Stromal.Up_Nat.Med.2009.PMID.19648928	1.25	0.93	1.68	0.14	0.40
SW480.cancer.cells_Immunity.2013.PMID.24138885	1.69	1.28	2.23	<0.001	0.07
T.follicular.helper.cell_CellRep.2017.PMID.28052254	0.95	0.72	1.26	0.74	0.87
Tcell.activation_Nature.2020.PMID.31942077	0.79	0.62	1.00	0.05	0.28
Tcell.CD8.Effector.vs.naive.2_Science.2016.PMID27789795	0.76	0.58	1.00	0.05	0.28
Tcell.CD8.Exhausted.vs.antiPDL1.2_Science.2016.PMID27789795	0.82	0.62	1.08	0.15	0.41
Tcell.CD8.Exhausted.vs.naive.2_Science.2016.PMID27789795	0.75	0.57	0.99	0.04	0.28
Tcell.CD8.Memory.vs.naive.1_Science.2016.PMID27789795	0.87	0.68	1.11	0.26	0.50
Tcell.cluster_CCR.2014.PMID.24916698	0.86	0.66	1.10	0.23	0.48
Tcell.EXH.Anti.PDL1.vs.control.treated.exhausted.CD8.Tcell.Metagene.1.Science.2016.PMID.27789795	0.94	0.74	1.19	0.60	0.78
Tcell.EXH.Effector.CD8.T.cell.at.day.8.p.i.Armstrong.vs.Naive.CD8.Tcell.Metagene.1_Science.2016.PMID.27789795	0.82	0.64	1.06	0.13	0.38
Tcell.EXH.Exhausted.CD8.T.cell.vs.Naive.CD8.T.cell.Metagene.1_Science.2016.PMID.27789795	0.86	0.68	1.09	0.21	0.46

Tcell.EXH.Exhausted.CD8.T.cell.vs.Naive.CD8.T.cell.Metagene.3_Science.2016.PMID.27789795	0.82	0.64	1.06	0.14	0.39
Tcell.EXH.Memory.CD8.T.cell.a.vs.Naive.CD8.T.cell.Metagene.1_Science.2016.PMID.27789795	0.87	0.68	1.11	0.26	0.50
Tcell.EXH.Memory.CD8.T.cell.a.vs.Naive.CD8.T.cell.Metagene.2_Science.2016.PMID.27789795	0.84	0.64	1.09	0.20	0.44
Tcell.EXH.Memory.CD8.T.cell.a.vs.Naive.CD8.T.cell.Metagene.3.Science_2016.PMID.27789795	0.75	0.58	0.97	0.03	0.28
Tcell.NK.51gene_Genome.Biol.2013.PMID.23618380	0.89	0.69	1.14	0.35	0.58
Tcell.NK.Metagene_Genome.Biol.2013.PMID.23618380	0.87	0.67	1.13	0.29	0.53
Tcell.RM_Nat_Med.2018.PMID.29942092	0.81	0.63	1.05	0.11	0.36
Tcell.survival.2gene_Nature.2020.PMID.31942077	0.93	0.74	1.18	0.57	0.75
Tcells_CancerImmunolRes.2018.PMID.30266715	0.87	0.67	1.13	0.29	0.53
Tcells_Immunity.2013.PMID.24138885	0.90	0.70	1.15	0.39	0.62
Tcells_TFH_Nat.Methods.2015.PMID.25822800	0.92	0.72	1.18	0.53	0.73
Tcells.CD4.memory.activated_Nat.Methods.2015.PMID.25822800	0.79	0.61	1.02	0.07	0.31
Tcells.CD4.memory.resting_Nat.Methods.2015.PMID.25822800	0.92	0.71	1.18	0.52	0.73
Tcells.CD4.naive_Nat.Methods.2015.PMID.25822800	0.95	0.74	1.22	0.70	0.83
Tcells.CD8_Immunity.2013.PMID.24138885	0.84	0.67	1.04	0.11	0.37
Tcells.CD8_Nat.Methods.2015.PMID.25822800	0.91	0.71	1.17	0.47	0.69
Tcells.CD8.MCP_Nature.2020.PMID.31942075	1.07	0.84	1.37	0.56	0.75
Tcells.Cytotoxic.MCP_Nature.2020.PMID.31942075	0.89	0.70	1.15	0.38	0.61
Tcells.gammadelta_Nat.Methods.2015.PMID.25822800	0.91	0.71	1.17	0.46	0.68
Tcells.helper_Immunity.2013.PMID.24138885	0.82	0.64	1.04	0.11	0.36
Tcells.MCP_Nature.2020.PMID.31942077	0.84	0.65	1.08	0.18	0.44
Tcells.regulatory.2gene_Nature.2020.PMID.31942077	0.97	0.77	1.23	0.82	0.91
Tcells.Tregs_Nat.Methods.2015.PMID.25822800	0.96	0.74	1.23	0.74	0.87
TCGA.BRCA.1198_BASAL_JCI.2020.PMID.32573490	1.49	1.11	1.99	0.008	0.18
TCGA.BRCA.1198_Chromogranin_JCI.2020.PMID.32573490	1.13	0.87	1.48	0.35	0.58
TCGA.BRCA.1198_COLLAGEN11A_JCI.2020.PMID.32573490	0.99	0.76	1.29	0.91	0.95
TCGA.BRCA.1198_EN1_FDZ9_JCI.2020.PMID.32573490	0.89	0.66	1.20	0.45	0.66
TCGA.BRCA.1198_FGFR4_EGF_JCI.2020.PMID.32573490	0.91	0.71	1.18	0.48	0.71
TCGA.BRCA.1198_HISTONES_JCI.2020.PMID.32573490	1.38	1.06	1.81	0.02	0.24
TCGA.BRCA.1198_HOXC11_HOTAIR_SIX1_JCI.2020.PMID.32573490	1.12	0.84	1.49	0.42	0.65
TCGA.BRCA.1198_IL8_CCL_JCI.2020.PMID.32573490	0.64	0.49	0.84	0.001	0.13
TCGA.BRCA.1198_immune_CD19_JCI.2020.PMID.32573490	0.85	0.65	1.12	0.25	0.49
TCGA.BRCA.1198_immune_CD34_TIE1_JCI.2020.PMID.32573490	1.27	0.95	1.69	0.11	0.36
TCGA.BRCA.1198_immune_CD4_CD53_CD84_BTK_JCI.2020.PMID.32573490	0.84	0.65	1.10	0.21	0.46
TCGA.BRCA.1198_immune_CD8_GZMK_JCI.2020.PMID.32573490	0.88	0.68	1.13	0.30	0.54
TCGA.BRCA.1198_immune_CTLA4_CXCL_FOXP3_JCI.2020.PMID.32573490	0.78	0.60	1.01	0.06	0.28
TCGA.BRCA.1198_immune_FOS_JUN_IL6_JCI.2020.PMID.32573490	1.00	0.81	1.24	0.97	0.98
TCGA.BRCA.1198_immune_GIMAP_IL16_JCI.2020.PMID.32573490	1.02	0.79	1.30	0.90	0.95
TCGA.BRCA.1198_immune_HLA_A_F_JCI.2020.PMID.32573490	0.94	0.75	1.19	0.63	0.80
TCGA.BRCA.1198_immune_HLA_D_JCI.2020.PMID.32573490	0.90	0.71	1.15	0.41	0.64
TCGA.BRCA.1198_immune_INTERFERON_JCI.2020.PMID.32573490	1.09	0.86	1.38	0.49	0.71
TCGA.BRCA.1198_IMMUNE1_JCI.2020.PMID.32573490	0.72	0.55	0.95	0.02	0.24
TCGA.BRCA.1198_LUMINAL_JCI.2020.PMID.32573490	1.06	0.74	1.53	0.74	0.87
TCGA.BRCA.1198_MYBL2_APOBEC3B_JCI.2020.PMID.32573490	0.70	0.52	0.94	0.02	0.24
TCGA.BRCA.1198_NORMAL_JCI.2020.PMID.32573490	1.27	0.94	1.72	0.12	0.37
TCGA.BRCA.1198_NORMAL2_JCI.2020.PMID.32573490	1.08	0.83	1.41	0.55	0.74
TCGA.BRCA.1198_PDCHA_MANY_JCI.2020.PMID.32573490	0.97	0.77	1.23	0.82	0.91
TCGA.BRCA.1198_S100A7_8_9_JCI.2020.PMID.32573490	0.98	0.72	1.32	0.88	0.95

TCGA.BRCA.1198_TP63_JCI.2020.PMID.32573490	1.47	1.13	1.92	0.004	0.17
TCGA.BRCA.1198.IMMUNOGLOBULIN_JCI.2020.PMID.32573490	0.75	0.58	0.97	0.03	0.28
TCGA.CSF1.response_Immunity.2018.PMID.29628290	0.81	0.63	1.05	0.12	0.37
TCGA.IFN.score_Immunity.2018.PMID.29628290	1.05	0.82	1.34	0.69	0.83
TCGA.Liexpression.score_Immunity.2018.PMID.29628290	0.91	0.70	1.16	0.44	0.65
TCGA.Serum.response.up_Immunity.2018.PMID.29628290	0.88	0.71	1.10	0.28	0.51
TCGA.TFH_Immunity.2018.PMID.29628290	0.99	0.79	1.23	0.92	0.95
TCGA.Tgd_Immunity.2018.PMID.29628290	1.34	1.02	1.77	0.04	0.28
TCGA.TGFB.score_Immunity.2018.PMID.29628290	1.20	0.93	1.56	0.16	0.43
Tcm_Immunity.2013.PMID.24138885	0.78	0.60	1.02	0.07	0.31
Tem_Immunity.2013.PMID.24138885	0.99	0.76	1.29	0.95	0.97
TFH_Immunity.2013.PMID.24138885	0.99	0.79	1.23	0.92	0.95
Tgd_Immunity.2013.PMID.24138885	1.34	1.02	1.77	0.04	0.28
Th1_cells_Immunity.2013.PMID.24138885	0.80	0.61	1.03	0.09	0.33
Th17_cells_Immunity.2013.PMID.24138885	1.18	0.90	1.53	0.23	0.48
Th2_cells_Immunity.2013.PMID.24138885	0.76	0.58	0.98	0.04	0.28
TLS.9Gene.Signature_Nature.2020.PMID.31942071	1.04	0.81	1.32	0.76	0.88
TLS.CXCL13.SingleGene_Nature.2020.PMID.31942077	0.74	0.59	0.94	0.01	0.21
TLS.Hallmark.Gene.Signature_Nature.2020.PMID.31942071	0.88	0.70	1.12	0.30	0.54
TLS.Known.Markers_Nature.2020.PMID.31942071	0.83	0.65	1.06	0.14	0.39
TLS.Structure.12chemokine_FrontImmunol.2017.PMID.28713385	0.79	0.62	1.01	0.06	0.28
TLS.tumors.wTLS.and.CD8.vs.CD8alone_Nature.2020.PMID.31942071	0.89	0.69	1.14	0.35	0.58
TNBC.good.prognosis.TNBC.230genes_BCR.2011.PMID.21978456	0.87	0.69	1.10	0.24	0.49
TNBC.good.prognosis.TNBC.26genes_BCR.2011.PMID.21978456	0.94	0.73	1.22	0.65	0.81
TNBC.metastasis.free.survival_PLoS.One.2013.PMID.24349199	0.66	0.51	0.85	0.001	0.13
TNBC.poor.prognosis.TNBC.26genes_BCR.2011.PMID.21978456	0.81	0.65	1.01	0.06	0.29
Translation.Pathway_CancerImmunolRes.2018.PMID.30266715	1.21	0.98	1.50	0.08	0.31
Tumour.hypoxia.causes.DNA.hypermethylation_Nature.2016.PMID.27533040	0.93	0.73	1.19	0.57	0.76
Type.1.T.helper.cell_CellRep.2017.PMID.28052254	0.80	0.63	1.02	0.07	0.31
Type.17.T.helper.cell_CellRep.2017.PMID.28052254	0.97	0.76	1.24	0.84	0.92
Type.2.T.helper.cell_CellRep.2017.PMID.28052254	0.80	0.64	0.98	0.04	0.28
Up.Basal.High_Nat.Cell.Biol.2014.PMID.25173976	1.42	1.08	1.86	0.01	0.21
Up.Proliferation_Nat.Cell.Biol.2014.PMID.25173976	0.77	0.59	1.01	0.06	0.28
Upregulated.by.oncogenic.NRAS.basal_Cell.Rep.2016.PMID.26166574	1.29	1.00	1.67	0.05	0.28
Upregulated.upon.NRAS.repression.basal_Cell.Rep.2017.PMID.26166574	1.08	0.85	1.38	0.52	0.73
Vascular.Content_Clin.Exp.Metastasis.2014.PMID.23975155	1.35	1.00	1.81	0.05	0.28
VEGF.13genes_BMC.Med.2009.PMID.19291283	0.90	0.69	1.16	0.41	0.64
Wirapati.Proliferation_BCR.2008.PMID.18662380	0.90	0.69	1.18	0.46	0.68
Wound.Signature_CCR.2009.PMID.19887484	1.46	1.12	1.91	0.006	0.18
X11q13.Amplicon_BMC.Med.Genomics.2011.PMID.21214954	1.11	0.92	1.34	0.29	0.53
X12qMDM4.BMC.Med.Genomics.2011.PMID.21214954	0.98	0.75	1.29	0.90	0.95
X13q14.Amplicon_BMC.Med.Genomics.2011.PMID.21214954	0.86	0.70	1.05	0.13	0.39
X15q25.Amplicon_BMC.Med.Genomics.2011.PMID.21214954	0.97	0.78	1.21	0.80	0.90
X16.13.Amplicon_BMC.Med.Genomics.2011.PMID.21214954	1.18	0.94	1.47	0.15	0.41
X16q23.Amplicon_BMC.Med.Genomics.2011.PMID.21214954	0.80	0.65	0.99	0.04	0.28
X17PP13.Amplicon_BMC.Med.Genomics.2011.PMID.21214954	0.83	0.66	1.06	0.13	0.39
X17q25x.BMC.Med.Genomics.2011.PMID.21214954	0.79	0.63	1.00	0.05	0.28
X19p13.Amplicon_BMC.Med.Genomics.2011.PMID.21214954	1.01	0.81	1.27	0.92	0.95

X1p36.Amplicon_BMC.Med.Genomics.2011.PMID.21214954	1.01	0.81	1.27	0.93	0.96
X3p21.Amplicon_BMC.Med.Genomics.2011.PMID.21214954	1.00	0.80	1.25	0.97	0.98
X4p16.Amplicon_BMC.Med.Genomics.2011.PMID.21214954	1.07	0.86	1.34	0.53	0.73
X5Q_BCRT.2012.PMID.22048815	0.85	0.68	1.05	0.13	0.39
X8p.Amplicon_BMC.Med.Genomics.2011.PMID.21214954	0.98	0.76	1.26	0.87	0.94
X8p22.Amplicon_BMC.Med.Genomics.2011.PMID.21214954	0.96	0.76	1.23	0.76	0.88
XBP1.Signature_Nature.2014.PMID.24670641	0.71	0.55	0.91	0.008	0.18

NSABP B-41					
Signature	HR	95% CI		*P*	adjusted *P*
Activate.Endothelium_Clin.Exp.Metastasis.2014.PMID.23975155	0.80	0.55	1.18	0.27	0.73
Activated.B.cell_CellRep.2017.PMID.28052254	0.71	0.50	1.02	0.07	0.38
Activated.Blood.Neutrophil.Signature_Nat.Cell.Biol.2019.PMID.31263265	1.08	0.74	1.58	0.70	0.96
Activated.Cancer.Cell.Signature_Nat.Cell.Biol.2019.PMID.31263265	0.94	0.66	1.34	0.74	0.96
Activated.CD4.T.cell_CellRep.2017.PMID.28052254	0.80	0.57	1.14	0.22	0.69
Activated.CD8.T.cell_CellRep.2017.PMID.28052254	0.67	0.47	0.96	0.03	0.38
Activated.dendritic.cell_CellRep.2017.PMID.28052254	0.58	0.40	0.85	0.01	0.38
Activated.Lung.MSC.Signature_Nat.Cell.Biol.2019.PMID.31263265	1.09	0.77	1.55	0.62	0.96
Activated.Lung.Neutrophil.Signature_Nat.Cell.Biol.2019.PMID.31263265	1.13	0.80	1.59	0.48	0.91
aDC_Immunity.2013_PMID.24138885.PMID.24138885	0.83	0.59	1.17	0.28	0.75
ADM.S100A10.A110NDGR1.Cluster_BMC.Med.Genomics.2011.PMID.21214954	1.34	0.89	2.03	0.16	0.61
African.and.European.Ancestry.TCGA.Negative_JAMA.Oncol.2017.PMID.28472234	0.96	0.63	1.48	0.87	0.97
African.and.European.Ancestry.TCGA.Positive_JAMA.Oncol.2017.PMID.28472234	1.02	0.70	1.48	0.91	0.99
Age.associated.signature_Genome.Biol.2015.PMID.26343147	1.01	0.70	1.46	0.95	0.99
aMaSC_BCR.2010.PMID.20346151	1.12	0.75	1.65	0.59	0.95
aMaSC.HsEnriched_BCR.2015.PMID.25575446	1.06	0.72	1.55	0.77	0.96
aMaSC.HsEnriched.Refined1_BCR.2015.PMID.25575446	1.39	0.96	2.03	0.08	0.44
aMaSC.Lim09_BCR.2015.PMID.25575446	0.99	0.67	1.48	0.98	>0.99
aMaSC.Prat_BCR.2015.PMID.25575446	0.81	0.55	1.20	0.29	0.77
aMaSC.Shehata_BCR.2015.PMID.25575446	0.74	0.48	1.14	0.17	0.62
aMaSC.Signature_Cell.Stem.Cell.2012.PMID.22305568	1.06	0.74	1.53	0.73	0.96
AMPH.EPIREGULIN.Cluster_BMC.Med.Genomics.2011.PMID.21214954	1.37	0.89	2.09	0.15	0.59
Amplification.50_Genome.Biol.2014.PMID.25164602	1.13	0.78	1.64	0.53	0.93
Amplification.50.better.than._Genome.Biol.2015.PMID.25164602	1.23	0.86	1.76	0.26	0.73
Apocrine.Features_J.Pathol.2017.PMID.27861902	1.20	0.82	1.75	0.35	0.83
aStr.HsEnriched_BCR.2015.PMID.25575446	1.00	0.67	1.49	>0.99	>0.99
aStr.HsEnriched.Refined1_BCR.2015.PMID.25575446	1.23	0.82	1.84	0.32	0.80
aStr.HsEnriched.Refined2_BCR.2015.PMID.25575446	0.95	0.63	1.41	0.78	0.96
aStr.Lim09_BCR.2015.PMID.25575446	1.11	0.74	1.66	0.61	0.95
aStr.Prat_BCR.2015.PMID.25575446	0.97	0.65	1.44	0.87	0.97
aStr.Shehata_BCR.2015.PMID.25575446	1.01	0.67	1.51	0.97	>0.99
BASAL.Cluster_BMC.Med.Genomics.2011.PMID.21214954	1.13	0.76	1.66	0.55	0.94
Bcell.cluster_CCR.2014.PMID.24916698	0.72	0.52	1.01	0.05	0.38
Bcell.IL10.MINUS_Immunol.2014.PMID.25080484	0.83	0.60	1.16	0.28	0.75
Bcell.IL10.PLUS_Immunol.2014.PMID.25080484	0.86	0.61	1.21	0.37	0.85

Bcell.lineage.MCP_Nature.2020.PMID.31942077	0.69	0.49	0.98	0.04	0.38
Bcell.Plasma.52gene_Genome.Biol.2013.PMID.23618380	0.72	0.52	1.00	0.05	0.38
Bcell.Plasma.Metagene_Genome.Biol.2013.PMID.23618380	0.76	0.56	1.04	0.09	0.45
Bcell.Tcell.Cooperation_Cell.2019.PMID.31730857	0.73	0.52	1.03	0.07	0.39
Bcells_CancerImmunolRes.2018.PMID.30266715	0.70	0.51	0.97	0.03	0.38
Bcells_Immunity.2013.PMID.24138885	0.76	0.53	1.10	0.15	0.59
Bcells.Centroblast_JCO.2015.PMID.25800755	0.98	0.66	1.47	0.94	0.99
Bcells.Centrocyte_JCO.2015.PMID.25800755	1.09	0.77	1.53	0.64	0.96
Bcells.Memory_JCO.2015.PMID.25800755	1.03	0.71	1.51	0.86	0.97
Bcells.memory_Nat.Methods.2015.PMID.25822800	0.74	0.52	1.05	0.09	0.46
Bcells.Naive_JCO.2015.PMID.25800755	0.97	0.67	1.40	0.87	0.97
Bcells.naive_Nat.Methods.2015.PMID.25822800	0.74	0.51	1.06	0.10	0.49
Bcells.Plasmablast_JCO.2015.PMID.25800755	0.95	0.69	1.32	0.78	0.96
Blood.vessels_Immunity.2013.PMID.24138885	1.17	0.76	1.80	0.48	0.91
bMYB.Signature_Oncogene.2009.PMID.19043454	0.94	0.63	1.41	0.78	0.96
C3TAG.Responding_CCR.2013.PMID.23780888	0.71	0.45	1.12	0.14	0.58
C3TAG.Untreated_CCR.2013.PMID.23780888	0.75	0.48	1.17	0.20	0.67
CD103.Negative_Cancer.Cell.2014.PMID.25446897	0.79	0.54	1.16	0.24	0.71
CD103.Positive_Cancer.Cell.2014.PMID.25446897	0.71	0.49	1.02	0.07	0.38
CD103.Ratio_Cancer.Cell.2014.PMID.25446897	0.60	0.40	0.90	0.01	0.38
CD274_Single_Gene.Single	0.75	0.53	1.05	0.09	0.46
CD34.CD36.Cluster_BMC.Med.Genomics.PMID.21214954	1.14	0.71	1.85	0.59	0.95
CD44.downregulated.genes_Cancer.Cell.2007.PMID.17349583	1.07	0.78	1.46	0.69	0.96
CD44.upregulated.genes_Cancer.Cell.2007.PMID.17349583	1.29	0.94	1.79	0.12	0.53
CD56bright.natural.killer.cell_CellRep.2017.PMID.28052254	0.88	0.63	1.23	0.45	0.90
CD56dim.natural.killer.cell_CellRep.2017.PMID.28052254	0.96	0.67	1.36	0.80	0.96
CD68.cluster_CCR.2014.PMID.24916698	0.93	0.62	1.38	0.70	0.96
CD8.cluster_CCR.2014.PMID.24916698	0.68	0.48	0.98	0.04	0.38
CDKN2A_Single_Gene.Single	1.33	0.94	1.88	0.10	0.49
Central.memory.CD4.T.cell_CellRep.2017.PMID.28052254	0.69	0.47	1.01	0.06	0.38
Central.memory.CD8.T.cell_CellRep.2017.PMID.28052254	0.89	0.63	1.26	0.53	0.93
CES.Score_CCR.2017.PMID.27903675	1.18	0.75	1.85	0.48	0.91
Chromogramin_BMC.Med.Genomics.2011.PMID.21214954	0.83	0.59	1.16	0.27	0.73
CIN70_Nat.Genet.2006.PMID.16921376	0.93	0.62	1.39	0.72	0.96
Claudin.High_Genome.Biol.2007.PMID.17493263	0.69	0.48	1.01	0.06	0.38
Claudin.Low_Genome.Biol.2007.PMID.17493263	1.08	0.72	1.60	0.71	0.96
Claudin.Low.29_Cancer.Res.2009.PMID.19435916	0.91	0.62	1.36	0.66	0.96
cMYB.Signature_PLoS.One.2010.PMID.20949095	0.94	0.64	1.38	0.76	0.96
CORE.Bcell.signature.Garber_Cell.Mol.Gastroenterol.Hepatol.2017.PMID.28508029	0.66	0.45	0.97	0.03	0.38
CTLA4_Single_Gene.Single	0.72	0.52	0.99	0.05	0.38
Cytolytic.activity_Cell.2015.PMID.25594174	0.64	0.44	0.92	0.02	0.38
Cytotoxic.cells_Immunity.2013.PMID.24138885	0.69	0.47	1.00	0.05	0.38
Day7.Downregulated_Nat.Cell.Biol.2014.PMID.25173976	1.13	0.77	1.66	0.53	0.93
Day7.Upregulated_Nat.Cell.Biol.2014.PMID.25173976	0.88	0.60	1.30	0.52	0.93
DC_Immunity.2013.PMID.24138885	0.71	0.49	1.02	0.07	0.38
DCIS.HGF.down_BCR.2013.PMID.24025166	0.71	0.48	1.04	0.08	0.41
DCIS.HGF.up_BCR.2014.PMID.24025166	0.77	0.54	1.10	0.14	0.58
Delection.50_Genome.Biol.2016.PMID.25164602	1.15	0.80	1.63	0.45	0.90

Delection.50.better.than_Genome.Biol.2017.PMID.25164602	1.02	0.73	1.42	0.91		0.99
Dendritic.cells.activated_Nat.Methods.2015.PMID.25822800	0.79	0.55	1.14	0.20		0.67
Dendritic.cells.resting_Nat.Methods.2015.PMID.25822800	0.74	0.50	1.09	0.12		0.53
Down.Basal.High_Nat.Cell.Biol.2014.PMID.25173976	1.05	0.71	1.53	0.82		0.96
Down.CLOW.High_Nat.Cell.Biol.2014.PMID.25173976	1.31	0.87	1.96	0.20		0.67
Downregulated.upon.NRAS.repression.basal_Cell.Rep.2015.PMID.26166574	0.87	0.61	1.24	0.44		0.90
Ductal.Carcinoma.In.Situ_J.Pathol.2017.PMID.27861902	1.02	0.75	1.37	0.91		0.99
Duke.Module01.acidosis_PNASUSA.2010.PMID.20335537	1.05	0.70	1.59	0.80		0.96
Duke.Module02.akt_PNASUSA.2010.PMID.20335537	0.73	0.49	1.07	0.11		0.49
Duke.Module03.betacatenin_PNASUSA.2010.PMID.20335537	0.87	0.59	1.29	0.50		0.92
Duke.Module04.E2F1_PNASUSA.2010.PMID.20335537	0.82	0.57	1.18	0.29		0.76
Duke.Module05.EGFR_PNASUSA.2010.PMID.20335537	0.90	0.59	1.38	0.63		0.96
Duke.Module06.ER_PNASUSA.2010.PMID.20335537	0.99	0.67	1.47	0.96	>0.99	
Duke.Module07.glucosedepletion_PNASUSA.2010.PMID.20335537	0.99	0.70	1.42	0.97	>0.99	
Duke.Module08.HER2_PNASUSA.2010.PMID.20335537	0.86	0.58	1.27	0.45		0.90
Duke.Module09.hypoxia_PNASUSA.2010.PMID.20335537	1.26	0.84	1.91	0.27		0.73
Duke.Module10.IFNA_PNASUSA.2010.PMID.20335537	1.26	0.86	1.84	0.24		0.71
Duke.Module11.IFNG_PNASUSA.2010.PMID.20335537	1.07	0.74	1.55	0.70		0.96
Duke.Module12.lacticacidosis_PNASUSA.2010.PMID.20335537	0.96	0.69	1.36	0.83		0.97
Duke.Module13.myc_PNASUSA.2010.PMID.20335537	0.95	0.64	1.40	0.78		0.96
Duke.Module14.p53_PNASUSA.2010.PMID.20335537	0.76	0.49	1.17	0.21		0.69
Duke.Module15.p63_PNASUSA.2010.PMID.20335537	1.00	0.68	1.45	0.99	>0.99	
Duke.Module16.pi3k_PNASUSA.2010.PMID.20335537	0.89	0.60	1.32	0.57		0.95
Duke.Module17.PR_PNASUSA.2010.PMID.20335537	1.09	0.71	1.67	0.69		0.96
Duke.Module18.ras_PNASUSA.2010.PMID.20335537	0.97	0.67	1.39	0.86		0.97
Duke.Module19.src_PNASUSA.2010.PMID.20335537	1.27	0.93	1.73	0.13		0.55
Duke.Module20.STAT3_PNASUSA.2010.PMID.20335537	0.96	0.65	1.43	0.85		0.97
Duke.Module21.TGFB_PNASUSA.2010.PMID.20335537	0.78	0.55	1.11	0.17		0.62
Duke.Module22.TNFA_PNASUSA.2010.PMID.20335537	1.05	0.73	1.50	0.79		0.96
Durvalumab.signature_CCR.2018.PMID.29716923	0.76	0.52	1.12	0.16		0.62
Early.IRS.1_PLoS.One.2016.PMID.26991655	0.99	0.67	1.45	0.95		0.99
Early.IRS.2_PLoS.One.2016.PMID.26991655	1.09	0.77	1.54	0.64		0.96
Early.Relapse.ERPos.33genes_JAMA.2011.PMID.21558518	1.13	0.80	1.60	0.49		0.91
Early.Response.ERNeg.27genes_JAMA.2011.PMID.21558518	1.14	0.79	1.65	0.47		0.91
Effector.memeory.CD4.T.cell_CellRep.2017.PMID.28052254	0.99	0.68	1.43	0.94		0.99
Effector.memeory.CD8.T.cell_CellRep.2017.PMID.28052254	0.70	0.48	1.01	0.05		0.38
EGFR_Single_Gene.Single	1.29	0.92	1.81	0.15		0.59
EMT.down.Taube_PNAS.2010.PMID.20713713	0.66	0.44	0.99	0.05		0.38
EMT.down.Weingberg_PNAS.2010.PMID.20713713	0.91	0.63	1.31	0.61		0.95
EMT.up.Taube_PNAS.2010.PMID.20713713	1.01	0.69	1.49	0.94		0.99
EMT.up.Weinberg_PNAS.2010.PMID.20713713	1.12	0.78	1.61	0.53		0.94
Endothelial.cells.MCP_Nature.2020..PMID.31942077	1.20	0.81	1.79	0.36		0.83
Endothelial.Normal_Angiogenesis.2014.PMID.24257808	0.90	0.62	1.31	0.59		0.95
Endothelial.Tumor_Angiogenesis.2014.PMID.24257808	0.79	0.55	1.15	0.22		0.69
Eosinophil_CellRep.2017.PMID.28052254	0.71	0.51	1.00	0.05		0.38
Eosinophils_Immunity.2013.PMID.24138885	1.07	0.73	1.56	0.74		0.96
Eosinophils_Nat.Methods.2015.PMID.25822800	0.63	0.44	0.91	0.01		0.38
Epithelial.Tubule.Formation_J.Pathol.2017.PMID.27861902	1.27	0.86	1.88	0.23		0.70

ERBB2_Single_Gene.Single	0.92	0.66	1.29	0.63	0.96
ERBB3_Single_Gene.Single	1.08	0.74	1.56	0.69	0.96
ESR1_Single_Gene.Single	1.12	0.66	1.89	0.67	0.96
ESTIMATE.Immune_Nat.Commun.2013.PMID.24113773	0.67	0.46	0.97	0.03	0.38
ESTIMATE.Stromal_Nat.Commun.2013.PMID.24113773	0.93	0.64	1.35	0.70	0.96
Euclidean.Distance.CLOW_BCR.2010.PMID.20813035	0.88	0.62	1.24	0.46	0.90
EXTENDED.Bcell.signature.Garber_Cell.Mol.Gastroenterol.Hepatol.2017.PMID.28508029	0.65	0.46	0.93	0.02	0.38
FGFR4_Single_Gene.Single	0.96	0.67	1.39	0.85	0.97
FGFR4.Induced_JCI.2020.PMID.32573490	0.86	0.60	1.23	0.41	0.87
FGFR4.Repressed_JCI.2020.PMID.32573490	0.98	0.67	1.44	0.94	0.99
Fibrinogen.Cluster_BMC.Med.Genomics.2011.PMID.21214954	0.87	0.60	1.26	0.46	0.90
Fibroblast.Cluster_BMC.Med.Genomics.2011.PMID.21214954	1.04	0.73	1.48	0.81	0.96
Fibroblasts.MCP_Nature.2020.PMID.31942077	0.86	0.59	1.25	0.43	0.90
Fibromatosis_Lab.Invest.2008.PMID.18414401	0.99	0.69	1.40	0.94	0.99
fMaSC.Metab_CellRep.2018.PMID.30089273	1.55	1.00	2.40	0.05	0.38
fMaSC.Metab8_CellRep.2018.PMID.30089273	1.15	0.78	1.69	0.47	0.91
fMaSC.refined1_BCR.2015.PMID.25575446	0.99	0.68	1.45	0.98	>0.99
fMasC.Signature_Cell.Stem.Cell.2012.PMID.22305568	0.80	0.55	1.18	0.26	0.73
fMaSC.Signature_CellRep.2018.PMID.30089273	0.99	0.67	1.46	0.95	0.99
FOS.JUN_Cluster_BMC.Med.Genomics.2011.PMID.21214954	1.15	0.80	1.65	0.46	0.90
FOXC1.Hair.Follicles.P30C.LO.vs.WT.Negative_Science.2016.PMID.26912704	1.08	0.75	1.55	0.67	0.96
FOXC1.Hair.Follicles.P30C.LO.vs.WT.Positive_Science.2016.PMID.26912704	1.03	0.73	1.47	0.86	0.97
fSTR.Signature_Cell.Stem.Cell.2012.PMID.22305568	1.21	0.75	1.96	0.44	0.90
Gamma.delta.T.cell_CellRep.2017.PMID.28052254	0.98	0.68	1.40	0.90	0.99
GATA3.induced.genes_JCO.2006.PMID.16505416	0.74	0.51	1.07	0.11	0.51
GATA3.induced.genes_Oncogene.2004.PMID.15361840	0.62	0.43	0.90	0.01	0.38
GDF11.TGFBR3_Nat.Cell.Biol.2014.PMID.24658685	1.31	0.93	1.85	0.12	0.54
Glycolysis_BMC.Med.2009.PMID.19291283	0.90	0.64	1.27	0.54	0.94
GO.DOWN.with.SOX10.OE_Cell.Rep.2015.PMID.26365194	0.88	0.60	1.27	0.48	0.91
GO.UP.with.SOX10.OE_Cell.Rep.2015.PMID.26365194	0.86	0.59	1.27	0.46	0.90
GSEA_BIOCARTA_ALK_PATHWAY.PMID.16199517	1.05	0.71	1.56	0.81	0.96
GSEA_BIOCARTA.AKT.PATHWAY.PMID.16199517	0.94	0.67	1.32	0.72	0.96
GSEA_BIOCARTA.BRCA.ATR.PATHWAY.ATRBRCA.PMID.16199517	0.93	0.63	1.37	0.71	0.96
GSEA_BIOCARTA.CASPASE.PATHWAY.PMID.16199517	0.93	0.65	1.33	0.69	0.96
GSEA_BIOCARTA.CTLA4.PATHWAY.PMID.16199517	0.71	0.50	1.00	0.05	0.38
GSEA_BIOCARTA.IGF1R.PATHWAY.PMID.16199517	1.12	0.75	1.69	0.57	0.95
GSEA_BIOCARTA.MTOR.PATHWAY.PMID.16199517	0.90	0.62	1.31	0.59	0.95
GSEA_BIOCARTA.PTEN.PATHWAY.PMID.16199517	0.95	0.66	1.37	0.79	0.96
GSEA_BIOCARTA.RAS.PATHWAY.PMID.16199517	1.19	0.83	1.71	0.34	0.82
GSEA_BIOCARTA.RB.PATHWAY.PMID.16199517	0.94	0.63	1.38	0.74	0.96
GSEA_BIOCARTA.VEGF.PATHWAY.PMID.16199517	1.20	0.87	1.67	0.26	0.73
GSEA_HALLMARK.MYC.TARGETS.V1.PMID.16199517	0.99	0.71	1.38	0.94	0.99
GSEA_HELLER.HDAC.TARGETS.DOWN.PMID.16199517	0.77	0.55	1.08	0.14	0.57
GSEA_NELSON.RESPONSE.TO.ANDROGEN.UP.PMID.16199517	0.85	0.59	1.21	0.37	0.84
GSEA_REACTOME.PD1.SIGNALING.PMID.16199517	0.67	0.47	0.94	0.02	0.38
GSEA_REACTOME.PI3K.CASCADE.PMID.16199517	1.06	0.70	1.59	0.79	0.96
GSEA_RETINOL.METABOLISM.KEGG.PMID.16199517	0.81	0.50	1.31	0.39	0.85
GSEA.GP1_Proliferation.DNA.repair..PUJANA.CHEK2.PCC.NETWORK.PMID.25109877	0.94	0.64	1.38	0.75	0.96

GSEA.GP1_Proliferation.DNA.repair.REACTOME.CELL.CYCLE.MITOTIC.PMID.25109877	0.99	0.69	1.41	0.94		0.99
GSEA.GP10_Fatty.acid.oxidation.CARBOXYLIC.ACID.METABOLIC.PROCESS.PMID.25109877	1.04	0.71	1.53	0.84		0.97
GSEA.GP11_Immune.IFN.PerouLab.PMID.25109877	1.21	0.83	1.78	0.32		0.79
GSEA.GP12_Hypoxia.glycolosis.SEMENZA.HIF1.TARGETS.PMID.25109877	1.00	0.68	1.46	0.99	>0.99	
GSEA.GP13_Neural.signailng.MODULE100.PMID.25109877	0.82	0.54	1.23	0.34		0.82
GSEA.GP13_Neural.signailng.NERVOUS.SYSTEM.DEVELOPMENT.PMID.25109877	1.08	0.74	1.56	0.69		0.96
GSEA.GP14_Plasma.membrane.cell.cell.signaling.MORF.CNTN1.PMID.25109877	0.76	0.50	1.14	0.18		0.64
GSEA.GP15_EGF.signaling.NAGASHIMA.EGF.SIGNALING.UP.PMID.25109877	1.03	0.71	1.51	0.87		0.97
GSEA.GP16_Protein.kinase.signaling.MAPKs.INTRACELLULAR.SIGNALING.CASCADE.PMID.25109877	0.88	0.61	1.27	0.49		0.91
GSEA.GP16_Protein.kinase.signaling.MAPKs.REGULATION.OF.KINASE.ACTIVITY.PMID.25109877	0.89	0.60	1.30	0.54		0.94
GSEA.GP17_Basal.signaling.SMID.BREAST.CANCER.BASAL.UP.PMID.25109877	0.99	0.68	1.42	0.94		0.99
GSEA.GP18_Vesicle.EPR.MEMBRANE.COAT.PMID.25109877	0.84	0.60	1.18	0.32		0.80
GSEA.GP19_1Q.amplicon.PerouLab.PMID.25109877	1.00	0.69	1.47	0.99	>0.99	
GSEA.GP2_Immune.Tcell.Bcell.KEGG.HEMATOPOIETIC.CELL.LINEAGE.PMID.25109877	0.74	0.52	1.05	0.09		0.45
GSEA.GP2_Immune.Tcell.Bcell.PerouLab.PMID.25109877	0.73	0.51	1.05	0.09		0.45
GSEA.GP20_TAL1.Leukemia.erythropoiesis.GNF2.TAL1.PMID.25109877	0.51	0.34	0.77	0.00		0.38
GSEA.GP21_Anti.apoptosis.DNA.stability.MORF.BCL2.PMID.25109877	0.93	0.63	1.37	0.71		0.96
GSEA.GP21_Anti.apoptosis.DNA.stability.MORF.MT4.PMID.25109877	0.69	0.46	1.03	0.07		0.39
GSEA.GP21_Anti.apoptosis.DNA.stability.MORF.STK17A.PMID.25109877	0.78	0.55	1.11	0.16		0.62
GSEA.GP22_16Q22.24.amplicon.PerouLab.PMID.25109877	1.08	0.76	1.53	0.69		0.96
GSEA.GP3_Tumo.suppressing.miRNA.targets.GTTTGTT.MIR.495.PMID.25109877	0.84	0.57	1.24	0.38		0.85
GSEA.GP3_Tumor.suppressing.miRNA.targets.DACOSTA.UV.RESPONSE.VIA.ERCC3.DN.PMID.25109877	1.10	0.67	1.81	0.71		0.96
GSEA.GP3_Tumor.suppressing.miRNA.targets.TGCTTTG.MIR.330.PMID.25109877	0.89	0.60	1.33	0.57		0.95
GSEA.GP4_MES.ECM.PerouLab.PMID.25109877	1.03	0.72	1.47	0.87		0.97
GSEA.GP5_MYC.targets.TERT.PerouLab.PMID.25109877	0.91	0.59	1.40	0.67		0.96
GSEA.GP6_Squamous.differentiation.development.RICKMAN.TUMOR.DIFFERENTIATED.WELL.VS.POORLY.DN.PMID.251098	0.83	0.57	1.22	0.35		0.83
GSEA.GP7_Estrogen.signaling.SMID.BREAST.CANCER.BASAL.DN.PMID.25109877	0.96	0.66	1.41	0.85		0.97
GSEA.GP8_FOXO.stemness.MORF.PTPRB.PMID.25109877	0.87	0.62	1.24	0.45		0.90
GSEA.GP8_FOXO.stemness.TTGTTT.VSFOXO4.01.PMID.25109877	1.11	0.75	1.65	0.60		0.95
GSEA.GP9_Cell.cell.adhesion.PerouLab.PMID.25109877	0.96	0.64	1.43	0.84		0.97
HCK_BCR.2008.PMID.19272155	0.69	0.46	1.04	0.07		0.40
HER1.Cluster1_BMC.Genomics.2007.PMID.17663798	1.03	0.73	1.46	0.85		0.97
HER1.Cluster2_BMC.Genomics.2007.PMID.17663798	0.95	0.64	1.39	0.78		0.96
HER1.Cluster3_BMC.Genomics.2007.PMID.17663798	0.98	0.66	1.45	0.92		0.99
HER2.Amplicon.PerouLab_BMC.Med.Genomic.2011.PMID.21214954	0.75	0.52	1.08	0.13		0.54
Histological.Grade_J.Pathol.2017.PMID.27861902	0.99	0.66	1.46	0.95		0.99
HouseKeeping_Genome.Biol.2004.PMID.15287981	0.85	0.60	1.22	0.39		0.85
iDC.Median_Immunity.2013.PMID.24138885	1.06	0.75	1.51	0.73		0.96
IFN.Cluster_BMC.Med.Genomics.2011.PMID.21214954	1.40	0.97	2.01	0.07		0.39
IgG_BCR.2008.PMID.19272155	0.77	0.57	1.05	0.10		0.49
IGG.Cluster_BMC.Med.Genomics.2011.PMID.21214954	0.72	0.51	1.02	0.07		0.39
Immature..B.cell_CellRep.2017.PMID.28052254	0.74	0.52	1.06	0.10		0.49
Immature.dendritic.cell_CellRep.2017.PMID.28052254	1.12	0.80	1.57	0.52		0.93
ImmLandscape_Macro.mono.CSF1.core.response_CCR.2009.PMID.29628290	0.68	0.47	0.99	0.04		0.38
ImmLandscape_Wound.Healing_Immunity.2018.PMID.29628290	0.76	0.52	1.12	0.16		0.62
ImmLandscape.IFN3_Plos.One.2014.PMID.24516633	1.50	1.05	2.14	0.03		0.38
ImmLandscape.IFNG5_Plos.One.2014.PMID.24516633	0.73	0.52	1.02	0.06		0.38
ImmLandscape.lymphocyte.Infil.T.B.PMID.18592372	0.71	0.51	1.00	0.05		0.38

Immune.Hot.CD8.vs.Cold_Nature.2020.PMID.31942071	0.68	0.47	0.97	0.04	0.38
Immune.Perez.14_JCO.2015.PMID.25605861	0.56	0.38	0.83	0.00	0.38
Immune.Perez.87_JCO.2015.PMID.25605861	0.68	0.48	0.96	0.03	0.38
Immune.Suppression_JCI.Insight.2016.PMID.27699256	0.82	0.58	1.16	0.27	0.73
ImmuneActive_Cell.2019.PMID.31730857	0.67	0.46	0.97	0.03	0.38
Immunosuppression.PMID.31942077	1.12	0.77	1.61	0.56	0.95
IMS.Score_CCR.2018.PMID.29921729	0.96	0.68	1.34	0.80	0.96
Induced.in.Bcells_PNAS.2013.PMID.23382184	0.84	0.60	1.18	0.32	0.79
Induced.in.DC_PNAS.2013.PMID.23382184	0.68	0.46	1.00	0.05	0.38
Induced.in.GN_PNAS.2013.PMID.23382184	0.95	0.62	1.44	0.81	0.96
Induced.in.HSC_PNAS.2013.PMID.23382184	1.16	0.83	1.63	0.39	0.85
Induced.in.MOs_PNAS.2013.PMID.23382184	0.80	0.55	1.15	0.22	0.70
Induced.in.NKcells_PNAS.2013.PMID.23382184	0.90	0.63	1.30	0.58	0.95
Induced.in.Tcells_PNAS.2013.PMID.23382184	0.81	0.58	1.12	0.20	0.67
Inflammatory.breast.cancer.491genes_CCR.2013.PMID.23396049	0.77	0.54	1.10	0.15	0.60
Inflammatory.breast.cancer.79genes_CCR.2013.PMID.23396049	1.05	0.72	1.55	0.79	0.96
Inflammatory.breast.cancer.expressed.noIBC_79genes_CCR.2013.PMID.23396049	1.04	0.73	1.49	0.81	0.96
Inflammatory.breast.cancer.expressed.noIBC.491genes_CCR.2013.PMID.23396049	1.12	0.73	1.74	0.60	0.95
Influenza.11genes.Metasignature_Immunity.2015.PMID.26682989	1.22	0.83	1.80	0.31	0.78
Interferon_BCR.2008.PMID.19272155	1.55	1.09	2.19	0.01	0.38
Interferon.Pathway_CancerImmunolRes.2018.PMID.30266715	1.37	0.93	2.00	0.11	0.51
JUND.KRT5_Nat.Cell.Biol.2014.PMID.24658685	0.99	0.66	1.50	0.96	>0.99
Keller2012.CD10.Adam_BCR.2015.PMID.25575446	1.08	0.76	1.53	0.67	0.96
KRAS.amplicon_Genome.Biology.2007.PMID.17493263	0.93	0.65	1.34	0.69	0.96
Late.IRS.1_PLoS.One.2016.PMID.26991655	1.19	0.83	1.71	0.33	0.81
Late.IRS.2_PLoS.One.2016.PMID.26991655	0.99	0.68	1.43	0.94	0.99
LCK_BCR.2008.PMID.19272155	0.65	0.45	0.94	0.02	0.38
Lim2009.LumProg.Adam_BCR.2015.PMID.25575446	0.95	0.64	1.42	0.81	0.96
Lim2009.MaSC.Adam_BCR.2015.PMID.25575446	1.00	0.67	1.50	>0.99	>0.99
Lim2009.MatureLum.Adam_BCR.2015.PMID.25575446	1.03	0.69	1.55	0.87	0.97
Lim2009.Stroma.Adam_BCR.2015.PMID.25575446	1.07	0.70	1.63	0.75	0.96
Lim2010.LumProg.Adam_BCR.2015.PMID.25575446	0.82	0.60	1.11	0.20	0.67
Lim2010.MaSC.Adam_BCR.2015.PMID.25575446	1.02	0.67	1.54	0.94	0.99
Lim2010.MatureLum.Adam_BCR.2015.PMID.25575446	1.12	0.78	1.59	0.54	0.94
Lim2010.Stroma.Adam_BCR.2015.PMID.25575446	0.98	0.65	1.47	0.91	0.99
Lobular.Carcinoma.In.Situ_J.Pathol.2017.PMID.27861902	1.13	0.78	1.64	0.50	0.93
LOBULAR.TCGA.SIGNATURE.ImmuneCell.2015.PMID.26451490	0.76	0.51	1.14	0.19	0.65
LOBULAR.TCGA.SIGNATURE.Reactive_Cell.2015.PMID.26451490	1.07	0.69	1.68	0.76	0.96
LOBULAR.TCGA.SUBTYPE.Immune_Cell.2015.PMID.26451490	1.04	0.55	1.97	0.89	0.99
LOBULAR.TCGA.SUBTYPE.Proliferative_Cell.2015.PMID.26451490	1.16	0.74	1.83	0.51	0.93
LOBULAR.TCGA.SUBTYPE.Reactive_Cell.2015.PMID.26451490	1.19	0.78	1.81	0.43	0.89
LTS.score_JCI.2020.PMID.32573490	1.12	0.74	1.70	0.59	0.95
Luminal_Progenitor_Up_Nat.Med.2009.PMID.19648928	0.79	0.52	1.21	0.27	0.74
Luminal.cluster_BMC.Med.Genomics.2011.PMID.21214954	1.07	0.69	1.66	0.76	0.96
Luminal.Progenitor_BCR.2010.PMID.20346151	0.68	0.46	0.99	0.04	0.38
Luminal.Progenitor.Down_Nat.Med.2009.PMID.19648928	1.06	0.74	1.51	0.75	0.96
LumProg.HsEnriched_BCR.2015.PMID.25575446	1.02	0.70	1.50	0.91	0.99
LumProg.HsEnriched.Refined1_BCR.2015.PMID.25575446	0.89	0.61	1.32	0.57	0.95

LumProg.Lim09_BCR.2015.PMID.25575446	0.90	0.60	1.34	0.59	0.95
LumProg.Prat_BCR.2015.PMID.25575446	0.78	0.53	1.16	0.23	0.70
LumProg.Shehata_BCR.2015.PMID.25575446	0.90	0.60	1.36	0.61	0.95
Lums.HER2E.DOWN.metastatic.signature_JCI.2020.PMID.32573490	1.17	0.82	1.67	0.39	0.85
Lums.HER2E.UP.metastatic.signature_JCI.2020.PMID.32573490	0.63	0.41	0.97	0.04	0.38
Lung.WNT_Cancer.Res.2009.PMID.19549913	1.12	0.76	1.65	0.56	0.95
Lymph.vessels_Immunity.2013.PMID.24138885	1.03	0.71	1.49	0.87	0.97
Lymphovascular.Invasion_J.Pathol.2017.PMID.27861902	0.97	0.70	1.34	0.87	0.97
M.D.Metagene_Genome.Biol.2013.PMID.23618380	0.64	0.45	0.92	0.02	0.38
M2.Macrophage_Blood.2006.PMID.16556895	0.65	0.44	0.97	0.03	0.38
Macrophage_CellRep.2017.PMID.28052254	0.86	0.58	1.25	0.42	0.89
Macrophages_CancerImmunolRes.2018.PMID.30266715	0.67	0.44	1.02	0.06	0.38
Macrophages_Immunity.2013.PMID.24138885	0.83	0.57	1.23	0.36	0.83
Macrophages.M0_Nat.Methods.2015.PMID.25822800	0.64	0.43	0.94	0.02	0.38
Macrophages.M1_Nat.Methods.2015.PMID.25822800	0.78	0.55	1.11	0.17	0.62
Macrophages.M2_Nat.Methods.2015.PMID.25822800	0.77	0.53	1.12	0.17	0.62
MacTh1.cluster_CCR.2014.PMID.24916698	0.66	0.45	0.98	0.04	0.38
MammaPrint_Nature.2002.PMID.11823860	1.09	0.75	1.59	0.65	0.96
MAPK.pathway.activation_NPJ.Precis.Oncol.2018.PMID.29872725	1.18	0.80	1.74	0.40	0.85
MASC.Down_Nat.Med.2009.PMID.19648928	0.95	0.63	1.44	0.83	0.96
MASC.Up_Nat.Med.2009.PMID.19648928	1.06	0.72	1.57	0.77	0.96
Mast.cell_CellRep.2017.PMID.28052254	0.73	0.52	1.02	0.07	0.39
Mast.cells_Immunity.2013.PMID.24138885	0.88	0.62	1.24	0.46	0.90
Mast.cells.activated_Nat.Methods.2015.PMID.25822800	0.89	0.61	1.30	0.55	0.94
Mast.cells.resting_Nat.Methods.2015.PMID.25822800	0.88	0.62	1.25	0.48	0.91
Mature.luminal_BCR.2010.PMID.20346151	0.87	0.60	1.25	0.45	0.90
Mature.Luminal.Down_Nat.Med.2009.PMID.19648928	1.10	0.71	1.69	0.67	0.96
Mature.LuminaUp_Nat.Med.2009.PMID.19648928	0.97	0.66	1.42	0.86	0.97
MatureLum.HsEnriched_BCR.2015.PMID.25575446	0.82	0.53	1.26	0.36	0.83
MatureLum.HsEnriched.Refined1_BCR.2015.PMID.25575446	0.90	0.63	1.29	0.57	0.95
MatureLum.Lim09_BCR.2015.PMID.25575446	1.01	0.66	1.53	0.97	>0.99
MatureLum.Prat_BCR.2015.PMID.25575446	0.90	0.58	1.40	0.64	0.96
MatureLum.Shehata_BCR.2015.PMID.25575446	0.91	0.60	1.39	0.67	0.96
MBasal.Cluster_BMC.Med.Genomics.2011.PMID.21214954	1.20	0.80	1.79	0.37	0.84
MCD3.CD8_BMC.Med.Genomics.2011.PMID.21214954	0.68	0.47	0.99	0.05	0.38
MCF7.E2.induced.genes_JCO.2006.PMID.16505416	0.95	0.66	1.37	0.80	0.96
MCF7.E2.repressed.genes_JCO.2006.PMID.16505416	0.99	0.66	1.47	0.95	0.99
MDSC_CellRep.2017.PMID.28052254	0.58	0.40	0.86	0.01	0.38
MDSC.Granulocytic_Leukoc.Biol.2012.PMID.21954284	0.90	0.63	1.27	0.54	0.94
MDSC.Neutrophi_Leukoc.Biol.2012.PMID.21954284	0.89	0.63	1.26	0.51	0.93
MDSC.tumor_J.Immunol.2012.PMID.23152559	0.90	0.62	1.30	0.56	0.95
MDSC.tumor.MO_J.Immunol.2012.PMID.23152559	0.85	0.57	1.27	0.43	0.90
MECM_BMC.Med.Genomics.2011.PMID.21214954	1.13	0.74	1.73	0.57	0.95
Memory.B.cell_CellRep.2017.PMID.28052254	0.87	0.62	1.22	0.42	0.89
MET.DOWN.RNAseq.Significant.Genes_JCI.2018.PMID.29480819	0.93	0.63	1.37	0.70	0.96
MET.DOWN.Significant.Genes.Low.Basal.1_JCI.2018.PMID.29480819	1.11	0.79	1.57	0.55	0.94
MET.DOWN.Significant.Genes.Low.Basal.2_JCI.2018.PMID.29480819	0.88	0.57	1.35	0.56	0.95
MET.UP.RNAseq.Significant.Genes_JCI.2018.PMID.29480819	1.33	0.85	2.09	0.22	0.69

MET.UP.Significant.Genes.HIGH.BASALS.Genes_JCI.2018.PMID.29480819	0.99	0.71	1.38	0.94	0.99
Metaplastic.Up_CanRes.2009.PMID.19435916	1.06	0.71	1.59	0.78	0.96
Metastasis.predictor.TNBC_BCR.2010.PMID.20946665	0.78	0.55	1.12	0.18	0.62
MFGFR2_BMC.Med.Genomics.2011.PMID.21214954	1.28	0.87	1.89	0.22	0.69
MHC.Forero.11_Cancer.Immunol.Res.2016.PMID.26980599	0.71	0.50	1.00	0.05	0.38
MHC.Forero.24_Cancer.Immunol.Res.2016.PMID.26980599	1.01	0.73	1.40	0.94	0.99
MHC.I_BCR.2008.PMID.19272155	0.98	0.70	1.39	0.93	0.99
MHC.II_BCR.2008.PMID.19272155	0.65	0.44	0.94	0.02	0.38
MHCI.coreGenes_Nat.Commun.2017.PMID29170503	0.98	0.68	1.41	0.91	0.99
MIR200c.Induced_ONCO.2015.PMID.25746005	0.79	0.57	1.11	0.17	0.62
MIR200c.Repressed_ONCO.2015.PMID.25746005	1.13	0.78	1.64	0.51	0.93
miRNA.138.signature_Cancer.Res.2014.PMID.25339353	0.84	0.58	1.22	0.36	0.83
MITO1_BMC.Med.Genomics.2011.PMID.21214954	0.74	0.45	1.20	0.22	0.70
MITO2_BMC.Med.Genomics.2011.PMID.21214954	1.05	0.72	1.53	0.79	0.96
Mitotic.Count_J.Pathol.2017.PMID.27861902	0.95	0.67	1.36	0.79	0.96
MK14.K17_BMC.Med.Genomics.2011.PMID.21214954	0.88	0.62	1.24	0.47	0.91
MKRAS.amplicon_BMC.Med.Genomics.2011.PMID.21214954	0.93	0.66	1.32	0.69	0.96
MM.BRCAwnt.1pFDR.UP_Genome.Biology.2007.PMID.17493263	1.17	0.77	1.79	0.46	0.90
MM.C3Tag.1pFDR.UP_Genome.Biology.2007.PMID.17493263	0.92	0.62	1.36	0.67	0.96
MM.C3Tag.2012_Genome.Biol.2013.PMID.24220145	1.01	0.69	1.47	0.96	>0.99
MM.Class3_Genome.Biol.2013.PMID.24220145	1.17	0.84	1.63	0.36	0.83
MM.Class8_Genome.Biol.2013.PMID.24220145	1.12	0.81	1.54	0.51	0.93
MM.Claudinlow_Genome.Biol.2013.PMID.24220145	0.91	0.61	1.37	0.65	0.96
MM.DMBAwnt.1pFDR.UP.Genome.Biology.2007.PMID.17493263	0.84	0.58	1.21	0.35	0.83
MM.Erbb2.like_Genome.Biol.2013.PMID.24220145	0.95	0.66	1.36	0.77	0.96
MM.Myc.2012_Genome.Biol.2013.PMID.24220145	0.96	0.66	1.39	0.81	0.96
MM.Myoepithelioma.like_Genome.Biol.2013.PMID.24220145	0.87	0.60	1.28	0.49	0.92
MM.Neu.2012_Genome.Biol.2013.PMID.24220145	0.95	0.68	1.33	0.76	0.96
MM.NeuPyMT.1pFDR.UP_Genome.Biology.2007.PMID.17493263	0.95	0.62	1.46	0.82	0.96
MM.Normal.1pFDR.UP_Genome.Biology.2007.PMID.17493263	0.80	0.52	1.23	0.31	0.78
MM.Normal.like_Genome.Biol.2013.PMID.24220145	1.01	0.58	1.76	0.96	>0.99
MM.p53null.1pFDR.UP_Genome.Biology.2007.PMID.17493263	0.92	0.64	1.33	0.66	0.96
MM.p53null.Basal_Genome.Biol.2013.PMID.24220145	1.22	0.85	1.77	0.28	0.75
MM.p53null.Luminal_Genome.Biol.2013.PMID.24220145	1.23	0.86	1.75	0.26	0.73
MM.Potluck_1pFDR_UP_Genome.Biology.2007_PMID.17493263.PMID.24220145	0.83	0.56	1.22	0.34	0.82
MM.PyMT.2012_Genome.Biol.2013.PMID.24220145	0.79	0.55	1.13	0.20	0.67
MM.Squamous.like_Genome.Biol.2013.PMID.24220145	0.94	0.66	1.35	0.75	0.96
MM.Stat1_Genome.Biol.2013.PMID.24220145	0.82	0.57	1.16	0.26	0.73
MM.WapINT3.1pFDR.UP_Genome.Biology.2007.PMID.17493263	0.86	0.58	1.28	0.46	0.90
MM.WapINT3.2012_Genome.Biol.2013.PMID.24220145	1.10	0.79	1.53	0.58	0.95
MM.WAPTag.1pFDR.UP_Genome.Biology.2007.PMID.17493263	1.05	0.68	1.61	0.83	0.96
MM.Wnt1.Early_Genome.Biol.2013.PMID.24220145	0.92	0.64	1.32	0.65	0.96
MM.Wnt1.Late_Genome.Biol.2013.PMID.24220145	0.83	0.59	1.18	0.30	0.77
Mmyosin_BMC.Med.Genomics.2011.PMID.21214954	1.10	0.74	1.64	0.63	0.96
MNADH_CYTochrome_BMC.Med.Genomics.2011.PMID.21214954	0.77	0.51	1.15	0.20	0.67
MNB1_BMC.Med.Genomics.2011.PMID.21214954	0.89	0.55	1.44	0.63	0.96
MNB2_BMC.Med.Genomics.2011.PMID.21214954	0.86	0.48	1.52	0.60	0.95
MNB3_BMC.Med.Genomics.2011.PMID.21214954	1.06	0.59	1.89	0.85	0.97

MNOtch4_BMC.Med.Genomics.2011.PMID.21214954	0.94	0.67	1.30	0.70	0.96
Monocyte_CellRep.2017.PMID.28052254	0.81	0.57	1.15	0.24	0.71
Monocyte..DC.25gene_Genome.Biol.2013.PMID.23618380	0.68	0.48	0.98	0.04	0.38
Monocytes_CancerImmunolRes.2018.PMID.30266715	0.71	0.47	1.08	0.11	0.51
Monocytes_Nat.Methods.2015.PMID.25822800	0.72	0.48	1.09	0.12	0.53
Monocytic.lineage.MCP_Nature.2020.PMID.31942075	0.78	0.52	1.17	0.23	0.70
MProliferation_BMC.Med.Genomics.2011.PMID.21214954	0.90	0.60	1.35	0.60	0.95
MProtocadherin_BMC.Med.Genomics.2011.PMID.21214954	0.73	0.51	1.07	0.11	0.50
MPYMT_NEU_Cluster_BMC.Med.Genomics.2011.PMID.21214954	0.86	0.63	1.19	0.36	0.83
MRibosomal_BMC.Med.Genomics.2011.PMID.21214954	1.05	0.66	1.67	0.84	0.97
MS.CD44.DOWN_PNAS.2009.PMID.19666588	1.07	0.72	1.58	0.75	0.96
MS.CD44.UP_PNAS.2009.PMID.19666588	0.96	0.64	1.43	0.83	0.97
MSquamous_BMC.Med.Genomics.2011.PMID.21214954	1.03	0.70	1.51	0.87	0.97
Murat.G07_JCO.2008.PMID.18565887	1.13	0.78	1.64	0.51	0.93
Murat.G18_JCO.2008.PMID.18565887	0.86	0.61	1.21	0.39	0.85
Murat.G24_JCO.2008.PMID.18565887	0.61	0.41	0.91	0.02	0.38
MVEGFC_BMC.Med.Genomics.2011.PMID.21214954	0.98	0.68	1.41	0.92	0.99
Myeloid.cell.chemotaxis.1gene_Nature.2020.PMID.31942077	1.11	0.77	1.62	0.57	0.95
Myeloid.dendritic.cells.MCP_Nature.2020.PMID.31942077	0.65	0.45	0.92	0.01	0.38
Natural.killer.cell_CellRep.2017.PMID.28052254	0.82	0.57	1.17	0.28	0.75
Natural.killer.T.cell_CellRep.2017.PMID.28052254	0.67	0.46	0.97	0.04	0.38
Necrosis_J.Pathol.2017.PMID.27861902	1.26	0.86	1.84	0.24	0.71
Neutrophil_CellRep.2017.PMID.28052254	1.07	0.72	1.59	0.74	0.96
Neutrophils_CancerImmunolRes.2018.PMID.30266715	0.79	0.52	1.20	0.27	0.73
Neutrophils_Immunity.2013.PMID.24138885	0.91	0.61	1.36	0.65	0.96
Neutrophils_Nat.Methods.2015.PMID.25822800	0.61	0.41	0.93	0.02	0.38
Neutrophils.MCP_Nature.2020.PMID.31942077	1.32	0.84	2.07	0.23	0.70
NK_Immunity.2013.PMID.24138885	1.01	0.73	1.39	0.96	0.99
NK.activated_Nat.Methods.2015.PMID.25822800	0.67	0.46	0.98	0.04	0.38
NK.CD56bright_Immunity.2013.PMID.24138885	0.70	0.49	1.00	0.05	0.38
NK.CD56dim_Immunity.2013.PMID.24138885	0.76	0.52	1.10	0.15	0.59
NK.resting_Nat.Methods.2015.PMID.25822800	0.69	0.47	1.00	0.05	0.38
NKcells_CancerImmunolRes.2018.PMID.30266715	0.67	0.47	0.94	0.02	0.38
NKcells.MCP_Nature.2020.PMID.31942077	0.72	0.45	1.15	0.17	0.62
No.Response.Immunotherapy.TLS.Melanoma_Nature.2020.PMID.31942075	1.55	1.08	2.23	0.02	0.38
Normal.mucosa_Immunity.2013.PMID.24138885	1.09	0.74	1.61	0.67	0.96
Nuclear.Pleomorphism_J.Pathol.2017.PMID.27861902	0.93	0.68	1.29	0.67	0.96
Oncotype_NEJM.2004.PMID.15591335	0.83	0.57	1.21	0.34	0.82
P53.ERPos.MDACC_CCR.2011.PMID.21248301	0.93	0.63	1.35	0.69	0.96
Parity.signature.251genes_BCR.2014.PMID.25005139	0.69	0.47	1.01	0.05	0.38
Parity.signature.40genes_BCR.2014.PMID.25005139	0.79	0.55	1.14	0.20	0.67
PARPi.Resistance_BCRT_2012.PMID.22875744	1.04	0.74	1.47	0.82	0.96
PARPi.Sensitivity_BCRT_2012.PMID.22875744	0.90	0.61	1.34	0.61	0.95
PARPi.Sensitivity.MDACC_NPJ.Syst.Biol.Appl.2017.PMID.28649435	0.96	0.69	1.35	0.83	0.96
PARPi.Sensitivity.Negative_Sci.Adv.2017.PMID.28439535	0.82	0.59	1.15	0.25	0.72
PARPi.Sensitivity.Positive_Sci.Adv.2017.PMID.28439535	0.92	0.64	1.33	0.67	0.96
Pcorr.Breast2Lung.LM2.Correlation_Nature.2005.PMID.16049480	0.95	0.69	1.31	0.75	0.96
Pcorr.Breast2Lung.Parental.Correlation_Nature.2005.PMID.16049480	1.05	0.76	1.45	0.76	0.96

Pcorr.dasatinib.resistant_Cancer.Res.2007.PMID.17332353	1.14	0.78	1.67	0.49	0.91
Pcorr.dasatinib.sensitive_Cancer.Res.2007.PMID.17332353	0.87	0.60	1.28	0.49	0.91
Pcorr.Hypoxia.High.Correlation_PLoS.Med.2006.PMID.16417408	1.21	0.86	1.71	0.27	0.73
Pcorr.Hypoxia.Low.Correlation_PLoS.Med.2006.PMID.16417408	0.85	0.60	1.20	0.35	0.83
Pcorr.IGS_Invasiveness_NJEM.2007.PMID.17229949	0.81	0.57	1.16	0.25	0.72
Pcorr.wound.response.activated_PNAS.2005.PMID.15701700	0.98	0.70	1.38	0.92	0.99
pCR.predictor.ERNeg.55genes_JAMA.2011.PMID.21558518	0.91	0.63	1.31	0.60	0.95
pCR.predictor.ERPos.39genes_JAMA.2011.PMID.21558518	1.26	0.90	1.76	0.17	0.62
PDCD1_Single_Gene.Single	0.67	0.46	0.98	0.04	0.38
Pfefferle2012.LumProg_BCR.2015.PMID.25575446	0.78	0.51	1.19	0.25	0.72
Pfefferle2012.MaSC_BCR.2015.PMID.25575446	0.88	0.60	1.30	0.53	0.93
Pfefferle2012.MatureLum_BCR.2015.PMID.25575446	0.91	0.63	1.31	0.60	0.95
Pfefferle2012.Stroma_BCR.2015.PMID.25575446	1.01	0.68	1.49	0.97	>0.99
PGR_Single_Gene.Single	1.02	0.68	1.54	0.91	0.99
PI3Ki.Down_CancerCell.2017.PMID.28528867	0.82	0.54	1.25	0.36	0.83
PI3Ki.Up_CancerCell.2017.PMID.28528867	0.90	0.58	1.39	0.62	0.96
PIK3CA.Pathway_Ann.Oncol.2017.PMID.28177460	0.87	0.58	1.30	0.49	0.91
PIK3CAmt.signature_Cancer.Res.2012.PMID.22552288	1.07	0.76	1.52	0.70	0.96
Plasma.cells_Nat.Methods.2015.PMID.25822800	0.72	0.51	1.02	0.06	0.38
PlasmaCells_CancerImmunolRes.2018.PMID.30266715	0.66	0.48	0.90	0.01	0.38
Plasmacytoid.dendritic.cell_CellRep.2017.PMID.28052254	0.68	0.46	1.01	0.06	0.38
PR.Isoform.Ratio.Up.in.PRA.H_JNCI.2017.PMID.28376177	1.07	0.72	1.58	0.75	0.96
PR.Isoform.Ratio.Up.in.PRB.H_JNCI.2017.PMID.28376177	0.88	0.61	1.27	0.51	0.93
Proliferation.Cluster_BMC.Med.Genomics.2011.PMID.21214954	0.97	0.65	1.47	0.90	0.99
Proliferation.Metagene_Genome.Biol.2013.PMID.23618380	0.93	0.62	1.39	0.72	0.96
Proliferation.score.PAM50_JCO.2009.PMID.19204204	0.83	0.57	1.20	0.32	0.79
ProliferationPathway_CancerImmunolRes.2018.PMID.30266715	0.95	0.64	1.43	0.82	0.96
Prosigna.Proliferation.18_BMC.Med.Genomics.2015.PMID.26297356	1.02	0.68	1.51	0.94	0.99
Race.LuminalA.MRE.score_BCRT.2015.PMID.26109344	0.98	0.71	1.36	0.92	0.99
Radiation.induced.genes_Radoat.Res.2014.PMID.24527691	1.11	0.69	1.79	0.67	0.96
RB.LOH_BCR.2008.PMID.18782450	0.95	0.64	1.41	0.79	0.96
RB.LOSS_JCI.2007.PMID.17160137	0.91	0.61	1.36	0.64	0.96
Regulatory.T.cell_CellRep.2017.PMID.28052254	0.72	0.48	1.08	0.11	0.51
Replication.Stress.Down.set_Cell.Rep.2018.PMID.29768207	0.94	0.66	1.33	0.71	0.96
Replication.Stress.Model_Cell.Rep.2018_PMID.29768207.PMID.29768207	1.06	0.77	1.45	0.72	0.96
Replication.Stress.Neg_Cell.Rep.2018_PMID.29768207.PMID.29768207	0.86	0.61	1.23	0.41	0.87
Replication.Stress.Pos_Cell.Rep.2018_PMID.29768207.PMID.29768207	0.90	0.63	1.27	0.54	0.94
Replication.Stress.Up_Set_Cell.Rep.2018_PMID.29768207.PMID.29768207	0.89	0.63	1.25	0.50	0.92
Residual.disease.predictor.ERNeg.54genes_JAMA.2011.PMID.21558518	0.95	0.68	1.31	0.74	0.96
Residual.disease.predictor.ERPos.73genes_JAMA.2011.PMID.21558518	0.82	0.56	1.20	0.31	0.78
Response.Immunotherapy.MCP.TLS.Melanoma_Nature.2020.PMID.31942075	0.68	0.48	0.97	0.03	0.38
Response.Immunotherapy.signature_Science.2018.PMID.30309915	0.71	0.50	1.02	0.06	0.38
Response.Neo.Chemo_common_CCR.2014.PMID.25047707	0.93	0.63	1.35	0.69	0.96
Response.Neo.Chemo_ERNeg_CCR.2014.PMID.25047707	1.23	0.92	1.65	0.17	0.62
Response.Neo.Chemo_ERPos_CCR.2014.PMID.25047707	1.24	0.89	1.73	0.21	0.67
RHOA.pathway_Ann.Oncol.2017.PMID.28177460	1.47	1.00	2.16	0.05	0.38
Ribosomal.Cluster_BMC.Med.Genomics.2011.PMID.21214954	0.82	0.51	1.32	0.41	0.87
ROR.subtype.PAM50_JCO.2009.PMID.19204204	0.70	0.45	1.08	0.11	0.49

ROR.subtype.proliferation.PAM50_JCO.2009.PMID.19204204	0.77	0.52	1.15	0.20	0.67
RSS.Score_CCR.2018.PMID.29921729	1.00	0.72	1.38	0.98	>0.99
S100A9.A8_BMC.Med.Genomics.2011.PMID.21214954	0.98	0.65	1.50	0.94	0.99
Scorr.EMAT1.Correlation_BCR.2020.PMID.32641077	0.92	0.65	1.31	0.66	0.96
Scorr.EMAT2.Correlation_BCR.2020.PMID.32641077	1.19	0.87	1.64	0.28	0.75
Scorr.EMAT3.Correlation_BCR.2020.PMID.32641077	1.02	0.71	1.46	0.93	0.99
Scorr.EMAT4.Correlation_BCR.2020.PMID.32641077	0.89	0.61	1.31	0.56	0.95
Scorr.IE.Correlation_JCO.2006.PMID.16505416	1.07	0.74	1.55	0.70	0.96
Scorr.IIE.Correlation_JCO.2006.PMID.16505416	0.85	0.59	1.23	0.39	0.85
Scorr.PAM50.Basal_JCO.2009.PMID.19204204	1.01	0.67	1.51	0.98	>0.99
Scorr.PAM50.Her2_JCO.2009.PMID.19204204	0.67	0.45	1.01	0.05	0.38
Scorr.PAM50.LumA_JCO.2009.PMID.19204204	1.35	0.86	2.13	0.19	0.67
Scorr.PAM50.LumB_JCO.2009.PMID.19204204	0.82	0.56	1.20	0.30	0.77
Scorr.PAM50.Normal_JCO.2009.PMID.19204204	1.28	0.86	1.89	0.23	0.70
Scorr.S329.L_Br.J.Cancer.2008.PMID.18382427	0.93	0.64	1.36	0.72	0.96
Scorr.S329.R_Br.J.Cancer.2008.PMID.18382427	0.93	0.64	1.36	0.72	0.96
Secretoglobin_BMC.Med.Genomics.2011.PMID.21214954	0.70	0.51	0.96	0.03	0.38
Shehata2012.ALDHneg_BCR.2015.PMID.25575446	0.88	0.64	1.22	0.44	0.90
Shehata2012.ALDHpos_BCR.2015.PMID.25575446	0.75	0.49	1.14	0.18	0.62
Shehata2012.Basal_BCR.2015.PMID.25575446	0.92	0.61	1.41	0.71	0.96
Shehata2012.Erbb3neg_BCR.2015.PMID.25575446	0.83	0.60	1.14	0.25	0.72
Shehata2012.LumProg_BCR.2015.PMID.25575446	0.89	0.60	1.31	0.56	0.95
Shehata2012.NCL_BCR.2015.PMID.25575446	0.86	0.58	1.27	0.45	0.90
Shehata2012.Stroma_BCR.2015.PMID.25575446	1.00	0.67	1.51	0.98	>0.99
Spike2012.aMaSC_BCR.2015.PMID.25575446	1.07	0.74	1.56	0.71	0.96
Spike2012.fMaSC_BCR.2015.PMID.25575446	0.84	0.58	1.21	0.34	0.82
Spike2012.fStr_BCR.2015.PMID.25575446	0.99	0.61	1.59	0.96	0.99
STAT1_BCR.2008.PMID.19272155	0.85	0.60	1.22	0.38	0.85
STAT3.Basal_PNAS.2014.PMID.25139989	0.70	0.49	1.01	0.06	0.38
STAT3.Basal.short_PNAS.2014.PMID.25139989	0.75	0.53	1.07	0.11	0.51
Stroma.FNA.MDACC.1_JCO.2010.PMID.20805453	0.73	0.52	1.02	0.07	0.39
Stroma.FNA.MDACC.2_JCO.2010.PMID.20805453	1.07	0.73	1.57	0.72	0.96
Stromal.Central.Fibrotic.Focus_J.Pathol.2017.PMID.27861902	0.78	0.55	1.10	0.15	0.60
Stromal.Down_Nat.Med.2009.PMID.19648928	0.86	0.60	1.24	0.43	0.90
Stromal.Inflammation_J.Pathol.2017.PMID.27861902	0.69	0.49	0.98	0.04	0.38
Stromal.Signature_Nat.Med.2008.PMID.18438415	0.73	0.51	1.06	0.10	0.49
Stromal.Up_Nat.Med.2009.PMID.19648928	1.08	0.72	1.63	0.70	0.96
SW480.cancer.cells_Immunity.2013.PMID.24138885	1.06	0.77	1.45	0.73	0.96
T.follicular.helper.cell_CellRep.2017.PMID.28052254	0.63	0.43	0.91	0.01	0.38
Tcell.activation_Nature.2020.PMID.31942077	0.87	0.62	1.21	0.41	0.87
Tcell.CD8.Effector.vs.naive.2_Science.2016.PMID27789795	0.94	0.63	1.41	0.76	0.96
Tcell.CD8.Exhausted.vs.antiPDL1.2_Science.2016.PMID27789795	0.88	0.60	1.30	0.53	0.93
Tcell.CD8.Exhausted.vs.naive.2_Science.2016.PMID27789795	0.93	0.62	1.40	0.74	0.96
Tcell.CD8.Memory.vs.naive.1_Science.2016.PMID27789795	0.72	0.51	1.01	0.06	0.38
Tcell.cluster_CCR.2014.PMID.24916698	0.69	0.48	0.98	0.04	0.38
Tcell.EXH.Anti.PDL1.vs.control.treated.exhausted.CD8.Tcell.Metagene.1.Science.2016.PMID.27789795	1.06	0.74	1.53	0.74	0.96
Tcell.EXH.Effector.CD8.T.cell.at.day.8.p.i.Armstrong.vs.Naive.CD8.Tcell.Metagene.1_Science.2016.PMID.27789795	0.84	0.59	1.19	0.32	0.80
Tcell.EXH.Exhausted.CD8.T.cell.vs.Naive.CD8.T.cell.Metagene.1_Science.2016.PMID.27789795	1.07	0.74	1.55	0.73	0.96

Tcell.EXH.Exhausted.CD8.T.cell.vs.Naive.CD8.T.cell.Metagene.3_Science.2016.PMID.27789795	0.77	0.54	1.08	0.13	0.55
Tcell.EXH.Memory.CD8.T.cell.a.vs.Naive.CD8.T.cell.Metagene.1_Science.2016.PMID.27789795	0.72	0.51	1.01	0.06	0.38
Tcell.EXH.Memory.CD8.T.cell.a.vs.Naive.CD8.T.cell.Metagene.2_Science.2016.PMID.27789795	0.88	0.63	1.22	0.44	0.90
Tcell.EXH.Memory.CD8.T.cell.a.vs.Naive.CD8.T.cell.Metagene.3.Science_2016.PMID.27789795	1.00	0.70	1.42	0.98	>0.99
Tcell.NK.51gene_Genome.Biol.2013.PMID.23618380	0.69	0.49	0.98	0.04	0.38
Tcell.NK.Metagene_Genome.Biol.2013.PMID.23618380	0.69	0.50	0.96	0.03	0.38
Tcell.RM_Nat_Med.2018.PMID.29942092	0.81	0.57	1.14	0.23	0.70
Tcell.survival.2gene_Nature.2020.PMID.31942077	0.69	0.47	1.02	0.06	0.38
Tcells_CancerImmunolRes.2018.PMID.30266715	0.66	0.46	0.95	0.02	0.38
Tcells_Immunity.2013.PMID.24138885	0.69	0.48	0.97	0.04	0.38
Tcells_TFH_Nat.Methods.2015.PMID.25822800	0.69	0.49	0.99	0.05	0.38
Tcells.CD4.memory.activated_Nat.Methods.2015.PMID.25822800	0.66	0.47	0.94	0.02	0.38
Tcells.CD4.memory.resting_Nat.Methods.2015.PMID.25822800	0.73	0.51	1.05	0.09	0.46
Tcells.CD4.naive_Nat.Methods.2015.PMID.25822800	0.71	0.49	1.03	0.07	0.39
Tcells.CD8_Immunity.2013.PMID.24138885	1.07	0.76	1.51	0.70	0.96
Tcells.CD8_Nat.Methods.2015.PMID.25822800	0.69	0.48	0.99	0.04	0.38
Tcells.CD8.MCP_Nature.2020.PMID.31942075	0.82	0.57	1.20	0.31	0.78
Tcells.Cytotoxic.MCP_Nature.2020.PMID.31942075	0.75	0.52	1.08	0.12	0.53
Tcells.gammadelta_Nat.Methods.2015.PMID.25822800	0.67	0.47	0.98	0.04	0.38
Tcells.helper_Immunity.2013.PMID.24138885	1.03	0.73	1.47	0.86	0.97
Tcells.MCP_Nature.2020.PMID.31942077	0.72	0.52	1.00	0.05	0.38
Tcells.regulatory.2gene_Nature.2020.PMID.31942077	0.66	0.49	0.89	0.01	0.38
Tcells.Tregs_Nat.Methods.2015.PMID.25822800	0.67	0.47	0.97	0.04	0.38
TCGA.BRCA.1198_BASAL_JCI.2020.PMID.32573490	1.17	0.80	1.72	0.41	0.87
TCGA.BRCA.1198_Chromogranin_JCI.2020.PMID.32573490	0.90	0.64	1.28	0.57	0.95
TCGA.BRCA.1198_COLLAGEN11A_JCI.2020.PMID.32573490	0.93	0.66	1.31	0.67	0.96
TCGA.BRCA.1198_EN1_FDZ9_JCI.2020.PMID.32573490	0.99	0.68	1.44	0.96	0.99
TCGA.BRCA.1198_FGFR4_EGF_JCI.2020.PMID.32573490	0.87	0.61	1.24	0.45	0.90
TCGA.BRCA.1198_HISTONES_JCI.2020.PMID.32573490	1.02	0.73	1.42	0.92	0.99
TCGA.BRCA.1198_HOXC11_HOTAIR_SIX1_JCI.2020.PMID.32573490	0.96	0.68	1.37	0.83	0.96
TCGA.BRCA.1198_IL8_CCL_JCI.2020.PMID.32573490	0.88	0.60	1.31	0.53	0.94
TCGA.BRCA.1198_immune_CD19_JCI.2020.PMID.32573490	0.71	0.51	0.99	0.05	0.38
TCGA.BRCA.1198_immune_CD34_TIE1_JCI.2020.PMID.32573490	0.96	0.64	1.44	0.85	0.97
TCGA.BRCA.1198_immune_CD4_CD53_CD84_BTK_JCI.2020.PMID.32573490	0.66	0.45	0.97	0.03	0.38
TCGA.BRCA.1198_immune_CD8_GZMK_JCI.2020.PMID.32573490	0.67	0.47	0.96	0.03	0.38
TCGA.BRCA.1198_immune_CTLA4_CXCL_FOXP3_JCI.2020.PMID.32573490	0.81	0.57	1.16	0.25	0.72
TCGA.BRCA.1198_immune_FOS_JUN_IL6_JCI.2020.PMID.32573490	1.36	1.06	1.75	0.02	0.38
TCGA.BRCA.1198_immune_GIMAP_IL16_JCI.2020.PMID.32573490	0.67	0.46	0.98	0.04	0.38
TCGA.BRCA.1198_immune_HLA_A_F_JCI.2020.PMID.32573490	0.94	0.65	1.34	0.72	0.96
TCGA.BRCA.1198_immune_HLA_D_JCI.2020.PMID.32573490	0.62	0.42	0.91	0.01	0.38
TCGA.BRCA.1198_immune_INTERFERON_JCI.2020.PMID.32573490	1.48	1.03	2.12	0.03	0.38
TCGA.BRCA.1198_IMMUNE1_JCI.2020.PMID.32573490	0.75	0.54	1.03	0.08	0.42
TCGA.BRCA.1198_LUMINAL_JCI.2020.PMID.32573490	1.10	0.69	1.77	0.68	0.96
TCGA.BRCA.1198_MYBL2_APOBEC3B_JCI.2020.PMID.32573490	0.88	0.60	1.28	0.51	0.93
TCGA.BRCA.1198_NORMAL_JCI.2020.PMID.32573490	0.91	0.54	1.53	0.71	0.96
TCGA.BRCA.1198_NORMAL2_JCI.2020.PMID.32573490	0.94	0.62	1.44	0.79	0.96
TCGA.BRCA.1198_PDCHA_MANY_JCI.2020.PMID.32573490	1.17	0.84	1.63	0.35	0.83
TCGA.BRCA.1198_S100A7_8_9_JCI.2020.PMID.32573490	0.82	0.57	1.19	0.30	0.77

TCGA.BRCA.1198_TP63_JCI.2020.PMID.32573490	1.06	0.70	1.59	0.79		0.96
TCGA.BRCA.1198.IMMUNOGLOBULIN_JCI.2020.PMID.32573490	0.77	0.56	1.07	0.12		0.52
TCGA.CSF1.response_Immunity.2018.PMID.29628290	0.68	0.47	0.99	0.04		0.38
TCGA.IFN.score_Immunity.2018.PMID.29628290	1.50	1.05	2.14	0.03		0.38
TCGA.Liexpression.score_Immunity.2018.PMID.29628290	0.71	0.51	1.00	0.05		0.38
TCGA.Serum.response.up_Immunity.2018.PMID.29628290	0.77	0.51	1.17	0.22		0.70
TCGA.TFH_Immunity.2018.PMID.29628290	1.10	0.77	1.55	0.61		0.95
TCGA.Tgd_Immunity.2018.PMID.29628290	1.28	0.84	1.96	0.25		0.72
TCGA.TGFB.score_Immunity.2018.PMID.29628290	1.28	0.89	1.86	0.18		0.64
Tcm_Immunity.2013.PMID.24138885	1.01	0.75	1.37	0.94		0.99
Tem_Immunity.2013.PMID.24138885	0.83	0.59	1.16	0.27		0.74
TFH_Immunity.2013.PMID.24138885	1.10	0.77	1.55	0.61		0.95
Tgd_Immunity.2013.PMID.24138885	1.28	0.84	1.96	0.25		0.72
Th1_cells_Immunity.2013.PMID.24138885	0.70	0.49	1.01	0.05		0.38
Th17_cells_Immunity.2013.PMID.24138885	1.00	0.74	1.36	>0.99	>0.99	
Th2_cells_Immunity.2013.PMID.24138885	1.11	0.76	1.62	0.59		0.95
TLS.9Gene.Signature_Nature.2020.PMID.31942071	0.74	0.51	1.09	0.13		0.55
TLS.CXCL13.SingleGene_Nature.2020.PMID.31942077	0.69	0.50	0.95	0.02		0.38
TLS.Hallmark.Gene.Signature_Nature.2020.PMID.31942071	0.64	0.44	0.94	0.02		0.38
TLS.Known.Markers_Nature.2020.PMID.31942071	0.71	0.50	1.02	0.06		0.38
TLS.Structure.12chemokine_FrontImmunol.2017.PMID.28713385	0.74	0.52	1.04	0.09		0.45
TLS.tumors.wTLS.and.CD8.vs.CD8alone_Nature.2020.PMID.31942071	0.67	0.46	0.97	0.03		0.38
TNBC.good.prognosis.TNBC.230genes_BCR.2011.PMID.21978456	0.90	0.61	1.32	0.59		0.95
TNBC.good.prognosis.TNBC.26genes_BCR.2011.PMID.21978456	0.81	0.60	1.10	0.17		0.62
TNBC.metastasis.free.survival_PLoS.One.2013.PMID.24349199	0.64	0.45	0.91	0.01		0.38
TNBC.poor.prognosis.TNBC.26genes_BCR.2011.PMID.21978456	0.97	0.66	1.41	0.86		0.97
Translation.Pathway_CancerImmunolRes.2018.PMID.30266715	0.81	0.49	1.31	0.38		0.85
Tumour.hypoxia.causes.DNA.hypermethylation_Nature.2016.PMID.27533040	1.21	0.85	1.73	0.29		0.76
Type.1.T.helper.cell_CellRep.2017.PMID.28052254	0.86	0.61	1.22	0.39		0.85
Type.17.T.helper.cell_CellRep.2017.PMID.28052254	0.73	0.51	1.04	0.08		0.44
Type.2.T.helper.cell_CellRep.2017.PMID.28052254	1.00	0.69	1.46	0.98	>0.99	
Up.Basal.High_Nat.Cell.Biol.2014.PMID.25173976	0.95	0.68	1.33	0.77		0.96
Up.Proliferation_Nat.Cell.Biol.2014.PMID.25173976	0.92	0.62	1.36	0.68		0.96
Upregulated.by.oncogenic.NRAS.basal_Cell.Rep.2016.PMID.26166574	0.73	0.50	1.07	0.10		0.49
Upregulated.upon.NRAS.repression.basal_Cell.Rep.2017.PMID.26166574	1.04	0.73	1.50	0.81		0.96
Vascular.Content_Clin.Exp.Metastasis.2014.PMID.23975155	1.17	0.82	1.67	0.40		0.85
VEGF.13genes_BMC.Med.2009.PMID.19291283	1.34	0.91	1.99	0.14		0.57
Wirapati.Proliferation_BCR.2008.PMID.18662380	0.94	0.63	1.39	0.74		0.96
Wound.Signature_CCR.2009.PMID.19887484	1.01	0.71	1.44	0.93		0.99
X11q13.Amplicon_BMC.Med.Genomics.2011.PMID.21214954	1.08	0.75	1.56	0.66		0.96
X12qMDM4.BMC.Med.Genomics.2011.PMID.21214954	0.74	0.47	1.17	0.20		0.67
X13q14.Amplicon_BMC.Med.Genomics.2011.PMID.21214954	1.15	0.83	1.58	0.41		0.87
X15q25.Amplicon_BMC.Med.Genomics.2011.PMID.21214954	0.90	0.61	1.34	0.61		0.95
X16.13.Amplicon_BMC.Med.Genomics.2011.PMID.21214954	0.89	0.61	1.28	0.53		0.93
X16q23.Amplicon_BMC.Med.Genomics.2011.PMID.21214954	0.82	0.57	1.18	0.29		0.76
X17PP13.Amplicon_BMC.Med.Genomics.2011.PMID.21214954	0.82	0.54	1.24	0.35		0.83
X17q25x.BMC.Med.Genomics.2011.PMID.21214954	1.25	0.91	1.72	0.16		0.62
X19p13.Amplicon_BMC.Med.Genomics.2011.PMID.21214954	0.94	0.67	1.31	0.70		0.96

X1p36.Amplicon_BMC.Med.Genomics.2011.PMID.21214954	0.66	0.46	0.94	0.02	0.38
X3p21.Amplicon_BMC.Med.Genomics.2011.PMID.21214954	0.79	0.51	1.23	0.30	0.77
X4p16.Amplicon_BMC.Med.Genomics.2011.PMID.21214954	0.83	0.59	1.18	0.30	0.77
X5Q_BCRT.2012.PMID.22048815	1.05	0.70	1.58	0.80	0.96
X8p.Amplicon_BMC.Med.Genomics.2011.PMID.21214954	0.87	0.61	1.24	0.44	0.90
X8p22.Amplicon_BMC.Med.Genomics.2011.PMID.21214954	1.15	0.80	1.65	0.46	0.90
XBP1.Signature_Nature.2014.PMID.24670641	1.32	0.91	1.92	0.15	0.59

eTable 10
Association of gene expression signatures at baseline with EFS in patients with residual disease in the combined cohort, CALGB 40601, NeoALTTO, and NSABP B-41.
Cox regression multivariable models adjusted by treatment arm, stage, node status, and HR status have been built for each gene expression biomarker. Models built using the combined cohort have been stratified by study
Adjusted p-values for multiple testing using a Benjamini & Hochberg method to control the False Discovery Rate are provided
HR: hazard ratio; CI: confident interval.

Combined cohort					
**Signature**	**HR**	**95% CI**		***P***	**adjusted *P***
Activate.Endothelium_Clin.Exp.Metastasis.2014.PMID.23975155	1.07	0.88	1.31	0.48	0.83
Activated.B.cell_CellRep.2017.PMID.28052254	0.79	0.65	0.96	0.02	0.19
Activated.Blood.Neutrophil.Signature_Nat.Cell.Biol.2019.PMID.31263265	0.88	0.73	1.06	0.17	0.54
Activated.Cancer.Cell.Signature_Nat.Cell.Biol.2019.PMID.31263265	1.03	0.85	1.27	0.74	0.93
Activated.CD4.T.cell_CellRep.2017.PMID.28052254	0.97	0.80	1.17	0.74	0.93
Activated.CD8.T.cell_CellRep.2017.PMID.28052254	0.77	0.64	0.93	0.01	0.19
Activated.dendritic.cell_CellRep.2017.PMID.28052254	0.74	0.60	0.92	0.01	0.19
Activated.Lung.MSC.Signature_Nat.Cell.Biol.2019.PMID.31263265	1.23	1.00	1.52	0.06	0.30
Activated.Lung.Neutrophil.Signature_Nat.Cell.Biol.2019.PMID.31263265	1.01	0.83	1.23	0.91	0.97
aDC_Immunity.2013_PMID.24138885.PMID.24138885	0.80	0.66	0.97	0.03	0.21
ADM.S100A10.A110NDGR1.Cluster_BMC.Med.Genomics.2011.PMID.21214954	1.04	0.85	1.28	0.68	0.91
African.and.European.Ancestry.TCGA.Negative_JAMA.Oncol.2017.PMID.28472234	0.98	0.81	1.20	0.87	0.97
African.and.European.Ancestry.TCGA.Positive_JAMA.Oncol.2017.PMID.28472234	1.12	0.94	1.34	0.22	0.60
Age.associated.signature_Genome.Biol.2015.PMID.26343147	1.12	0.92	1.35	0.27	0.68
aMaSC_BCR.2010.PMID.20346151	1.06	0.87	1.30	0.56	0.87
aMaSC.HsEnriched_BCR.2015.PMID.25575446	1.23	1.01	1.50	0.04	0.26
aMaSC.HsEnriched.Refined1_BCR.2015.PMID.25575446	1.14	0.94	1.39	0.17	0.54
aMaSC.Lim09_BCR.2015.PMID.25575446	1.09	0.89	1.33	0.39	0.77
aMaSC.Prat_BCR.2015.PMID.25575446	1.10	0.91	1.35	0.33	0.72
aMaSC.Shehata_BCR.2015.PMID.25575446	0.96	0.79	1.16	0.64	0.89
aMaSC.Signature_Cell.Stem.Cell.2012.PMID.22305568	1.09	0.89	1.34	0.40	0.77
AMPH.EPIREGULIN.Cluster_BMC.Med.Genomics.2011.PMID.21214954	1.04	0.85	1.27	0.68	0.91
Amplification.50_Genome.Biol.2014.PMID.25164602	1.14	0.96	1.36	0.14	0.50
Amplification.50.better.than._Genome.Biol.2015.PMID.25164602	1.06	0.88	1.27	0.53	0.86
Apocrine.Features_J.Pathol.2017.PMID.27861902	1.06	0.87	1.30	0.56	0.87
aStr.HsEnriched_BCR.2015.PMID.25575446	0.93	0.76	1.12	0.43	0.78
aStr.HsEnriched.Refined1_BCR.2015.PMID.25575446	1.06	0.87	1.30	0.54	0.86
aStr.HsEnriched.Refined2_BCR.2015.PMID.25575446	0.95	0.79	1.14	0.57	0.87
aStr.Lim09_BCR.2015.PMID.25575446	0.99	0.82	1.20	0.91	0.97
aStr.Prat_BCR.2015.PMID.25575446	0.94	0.78	1.15	0.56	0.87
aStr.Shehata_BCR.2015.PMID.25575446	0.97	0.79	1.18	0.73	0.93
BASAL.Cluster_BMC.Med.Genomics.2011.PMID.21214954	1.09	0.89	1.34	0.40	0.77
Bcell.cluster_CCR.2014.PMID.24916698	0.79	0.65	0.96	0.02	0.19
Bcell.IL10.MINUS_Immunol.2014.PMID.25080484	0.93	0.76	1.15	0.52	0.85
Bcell.IL10.PLUS_Immunol.2014.PMID.25080484	0.84	0.68	1.03	0.10	0.42
Bcell.lineage.MCP_Nature.2020.PMID.31942077	0.75	0.61	0.92	0.00	0.19
Bcell.Plasma.52gene_Genome.Biol.2013.PMID.23618380	0.75	0.62	0.92	0.00	0.19
Bcell.Plasma.Metagene_Genome.Biol.2013.PMID.23618380	0.80	0.66	0.97	0.02	0.20
Bcell.Tcell.Cooperation_Cell.2019.PMID.31730857	0.84	0.69	1.02	0.08	0.37

Bcells_CancerImmunolRes.2018.PMID.30266715	0.77	0.63	0.93	0.01	0.19
Bcells_Immunity.2013.PMID.24138885	0.86	0.70	1.05	0.13	0.48
Bcells.Centroblast_JCO.2015.PMID.25800755	1.09	0.90	1.31	0.38	0.77
Bcells.Centrocyte_JCO.2015.PMID.25800755	1.04	0.86	1.27	0.67	0.90
Bcells.Memory_JCO.2015.PMID.25800755	0.85	0.70	1.04	0.12	0.47
Bcells.memory_Nat.Methods.2015.PMID.25822800	0.79	0.65	0.96	0.02	0.19
Bcells.Naive_JCO.2015.PMID.25800755	0.98	0.80	1.20	0.82	0.97
Bcells.naive_Nat.Methods.2015.PMID.25822800	0.82	0.67	1.00	0.05	0.27
Bcells.Plasmablast_JCO.2015.PMID.25800755	0.91	0.74	1.12	0.36	0.76
Blood.vessels_Immunity.2013.PMID.24138885	1.05	0.87	1.27	0.59	0.88
bMYB.Signature_Oncogene.2009.PMID.19043454	1.06	0.88	1.27	0.56	0.87
C3TAG.Responding_CCR.2013.PMID.23780888	0.92	0.76	1.11	0.40	0.77
C3TAG.Untreated_CCR.2013.PMID.23780888	1.04	0.86	1.26	0.67	0.90
CD103.Negative_Cancer.Cell.2014.PMID.25446897	0.89	0.74	1.07	0.20	0.58
CD103.Positive_Cancer.Cell.2014.PMID.25446897	0.86	0.72	1.03	0.11	0.45
CD103.Ratio_Cancer.Cell.2014.PMID.25446897	0.84	0.70	1.02	0.08	0.37
CD274_Single_Gene.Single	0.79	0.64	0.97	0.03	0.21
CD34.CD36.Cluster_BMC.Med.Genomics.PMID.21214954	0.93	0.76	1.13	0.46	0.81
CD44.downregulated.genes_Cancer.Cell.2007.PMID.17349583	0.90	0.73	1.10	0.29	0.69
CD44.upregulated.genes_Cancer.Cell.2007.PMID.17349583	1.26	1.04	1.53	0.02	0.19
CD56bright.natural.killer.cell_CellRep.2017.PMID.28052254	0.98	0.81	1.19	0.86	0.97
CD56dim.natural.killer.cell_CellRep.2017.PMID.28052254	0.97	0.80	1.17	0.74	0.93
CD68.cluster_CCR.2014.PMID.24916698	0.95	0.79	1.15	0.62	0.89
CD8.cluster_CCR.2014.PMID.24916698	0.79	0.65	0.96	0.02	0.19
CDKN2A_Single_Gene.Single	1.18	0.99	1.40	0.07	0.36
Central.memory.CD4.T.cell_CellRep.2017.PMID.28052254	0.94	0.78	1.13	0.51	0.85
Central.memory.CD8.T.cell_CellRep.2017.PMID.28052254	0.88	0.73	1.07	0.19	0.56
CES.Score_CCR.2017.PMID.27903675	0.86	0.67	1.11	0.25	0.65
Chromogramin_BMC.Med.Genomics.2011.PMID.21214954	0.92	0.75	1.12	0.39	0.77
CIN70_Nat.Genet.2006.PMID.16921376	1.11	0.92	1.34	0.27	0.68
Claudin.High_Genome.Biol.2007.PMID.17493263	0.79	0.65	0.96	0.02	0.19
Claudin.Low_Genome.Biol.2007.PMID.17493263	1.20	0.97	1.48	0.09	0.40
Claudin.Low.29_Cancer.Res.2009.PMID.19435916	1.17	0.95	1.44	0.13	0.48
cMYB.Signature_PLoS.One.2010.PMID.20949095	1.03	0.84	1.25	0.80	0.96
CORE.Bcell.signature.Garber_Cell.Mol.Gastroenterol.Hepatol.2017.PMID.28508029	0.78	0.64	0.96	0.02	0.19
CTLA4_Single_Gene.Single	0.85	0.71	1.03	0.09	0.40
Cytolytic.activity_Cell.2015.PMID.25594174	0.78	0.65	0.93	0.01	0.19
Cytotoxic.cells_Immunity.2013.PMID.24138885	0.84	0.68	1.02	0.08	0.37
Day7.Downregulated_Nat.Cell.Biol.2014.PMID.25173976	1.01	0.82	1.24	0.91	0.97
Day7.Upregulated_Nat.Cell.Biol.2014.PMID.25173976	1.10	0.91	1.31	0.32	0.72
DC_Immunity.2013.PMID.24138885	0.78	0.64	0.94	0.01	0.19
DCIS.HGF.down_BCR.2013.PMID.24025166	1.08	0.88	1.33	0.45	0.80
DCIS.HGF.up_BCR.2014.PMID.24025166	1.04	0.85	1.27	0.71	0.92
Delection.50_Genome.Biol.2016.PMID.25164602	1.14	0.94	1.39	0.19	0.56
Delection.50.better.than_Genome.Biol.2017.PMID.25164602	1.26	1.04	1.53	0.02	0.19
Dendritic.cells.activated_Nat.Methods.2015.PMID.25822800	0.88	0.73	1.08	0.23	0.62
Dendritic.cells.resting_Nat.Methods.2015.PMID.25822800	0.79	0.64	0.97	0.02	0.20
Down.Basal.High_Nat.Cell.Biol.2014.PMID.25173976	1.01	0.82	1.24	0.95	0.98
Down.CLOW.High_Nat.Cell.Biol.2014.PMID.25173976	1.14	0.95	1.37	0.15	0.52

Downregulated.upon.NRAS.repression.basal_Cell.Rep.2015.PMID.26166574	0.87	0.72	1.05	0.16	0.52
Ductal.Carcinoma.In.Situ_J.Pathol.2017.PMID.27861902	0.91	0.75	1.10	0.33	0.72
Duke.Module01.acidosis_PNASUSA.2010.PMID.20335537	0.85	0.69	1.04	0.11	0.45
Duke.Module02.akt_PNASUSA.2010.PMID.20335537	0.81	0.69	0.95	0.01	0.19
Duke.Module03.betacatenin_PNASUSA.2010.PMID.20335537	1.03	0.86	1.23	0.78	0.96
Duke.Module04.E2F1_PNASUSA.2010.PMID.20335537	0.98	0.81	1.18	0.82	0.97
Duke.Module05.EGFR_PNASUSA.2010.PMID.20335537	1.05	0.87	1.26	0.60	0.88
Duke.Module06.ER_PNASUSA.2010.PMID.20335537	0.99	0.79	1.24	0.93	0.98
Duke.Module07.glucosedepletion_PNASUSA.2010.PMID.20335537	0.83	0.68	1.01	0.06	0.32
Duke.Module08.HER2_PNASUSA.2010.PMID.20335537	1.04	0.86	1.26	0.70	0.92
Duke.Module09.hypoxia_PNASUSA.2010.PMID.20335537	1.06	0.87	1.28	0.58	0.87
Duke.Module10.IFNA_PNASUSA.2010.PMID.20335537	1.07	0.88	1.30	0.48	0.82
Duke.Module11.IFNG_PNASUSA.2010.PMID.20335537	1.02	0.84	1.24	0.86	0.97
Duke.Module12.lacticacidosis_PNASUSA.2010.PMID.20335537	1.22	1.00	1.48	0.04	0.27
Duke.Module13.myc_PNASUSA.2010.PMID.20335537	1.05	0.88	1.25	0.60	0.88
Duke.Module14.p53_PNASUSA.2010.PMID.20335537	0.95	0.77	1.18	0.66	0.90
Duke.Module15.p63_PNASUSA.2010.PMID.20335537	0.92	0.76	1.11	0.39	0.77
Duke.Module16.pi3k_PNASUSA.2010.PMID.20335537	1.08	0.89	1.31	0.41	0.78
Duke.Module17.PR_PNASUSA.2010.PMID.20335537	1.05	0.82	1.35	0.70	0.92
Duke.Module18.ras_PNASUSA.2010.PMID.20335537	0.98	0.82	1.18	0.86	0.97
Duke.Module19.src_PNASUSA.2010.PMID.20335537	0.98	0.81	1.19	0.84	0.97
Duke.Module20.STAT3_PNASUSA.2010.PMID.20335537	0.95	0.76	1.18	0.62	0.89
Duke.Module21.TGFB_PNASUSA.2010.PMID.20335537	1.10	0.91	1.34	0.32	0.72
Duke.Module22.TNFA_PNASUSA.2010.PMID.20335537	0.95	0.78	1.16	0.59	0.88
Durvalumab.signature_CCR.2018.PMID.29716923	0.87	0.72	1.06	0.17	0.54
Early.IRS.1_PLoS.One.2016.PMID.26991655	1.06	0.86	1.30	0.61	0.89
Early.IRS.2_PLoS.One.2016.PMID.26991655	1.13	0.94	1.37	0.20	0.58
Early.Relapse.ERPos.33genes_JAMA.2011.PMID.21558518	0.99	0.82	1.20	0.95	0.98
Early.Response.ERNeg.27genes_JAMA.2011.PMID.21558518	1.01	0.83	1.22	0.95	0.98
Effector.memeory.CD4.T.cell_CellRep.2017.PMID.28052254	0.89	0.74	1.07	0.21	0.59
Effector.memeory.CD8.T.cell_CellRep.2017.PMID.28052254	0.81	0.67	0.99	0.04	0.25
EGFR_Single_Gene.Single	1.18	0.96	1.45	0.11	0.45
EMT.down.Taube_PNAS.2010.PMID.20713713	1.06	0.88	1.28	0.54	0.86
EMT.down.Weingberg_PNAS.2010.PMID.20713713	1.17	0.96	1.42	0.12	0.47
EMT.up.Taube_PNAS.2010.PMID.20713713	0.97	0.80	1.17	0.73	0.93
EMT.up.Weinberg_PNAS.2010.PMID.20713713	1.05	0.86	1.28	0.62	0.89
Endothelial.cells.MCP_Nature.2020..PMID.31942077	1.14	0.94	1.38	0.19	0.56
Endothelial.Normal_Angiogenesis.2014.PMID.24257808	1.12	0.92	1.36	0.27	0.68
Endothelial.Tumor_Angiogenesis.2014.PMID.24257808	0.99	0.81	1.20	0.90	0.97
Eosinophil_CellRep.2017.PMID.28052254	0.85	0.71	1.02	0.07	0.36
Eosinophils_Immunity.2013.PMID.24138885	1.01	0.83	1.23	0.94	0.98
Eosinophils_Nat.Methods.2015.PMID.25822800	0.75	0.63	0.91	0.00	0.19
Epithelial.Tubule.Formation_J.Pathol.2017.PMID.27861902	1.00	0.82	1.21	>0.99	>0.99
ERBB2_Single_Gene.Single	1.02	0.85	1.24	0.81	0.97
ERBB3_Single_Gene.Single	1.27	1.03	1.55	0.02	0.20
ESR1_Single_Gene.Single	0.92	0.69	1.24	0.60	0.88
ESTIMATE.Immune_Nat.Commun.2013.PMID.24113773	0.79	0.64	0.96	0.02	0.19
ESTIMATE.Stromal_Nat.Commun.2013.PMID.24113773	0.89	0.74	1.08	0.25	0.65
Euclidean.Distance.CLOW_BCR.2010.PMID.20813035	1.09	0.89	1.33	0.42	0.78

EXTENDED.Bcell.signature.Garber_Cell.Mol.Gastroenterol.Hepatol.2017.PMID.28508029	0.78	0.66	0.94	0.01	0.19
FGFR4_Single_Gene.Single	1.17	0.96	1.42	0.13	0.47
FGFR4.Induced_JCI.2020.PMID.32573490	0.98	0.80	1.20	0.87	0.97
FGFR4.Repressed_JCI.2020.PMID.32573490	0.89	0.73	1.09	0.26	0.66
Fibrinogen.Cluster_BMC.Med.Genomics.2011.PMID.21214954	1.01	0.83	1.22	0.96	0.98
Fibroblast.Cluster_BMC.Med.Genomics.2011.PMID.21214954	1.08	0.89	1.32	0.44	0.79
Fibroblasts.MCP_Nature.2020.PMID.31942077	1.00	0.83	1.22	0.96	0.98
Fibromatosis_Lab.Invest.2008.PMID.18414401	1.07	0.87	1.30	0.54	0.86
fMaSC.Metab_CellRep.2018.PMID.30089273	1.06	0.88	1.29	0.52	0.85
fMaSC.Metab8_CellRep.2018.PMID.30089273	0.98	0.79	1.21	0.84	0.97
fMaSC.refined1_BCR.2015.PMID.25575446	0.94	0.77	1.14	0.52	0.85
fMasC.Signature_Cell.Stem.Cell.2012.PMID.22305568	0.85	0.70	1.05	0.13	0.47
fMaSC.Signature_CellRep.2018.PMID.30089273	0.99	0.83	1.18	0.91	0.97
FOS.JUN_Cluster_BMC.Med.Genomics.2011.PMID.21214954	1.03	0.85	1.24	0.77	0.95
FOXC1.Hair.Follicles.P30C.LO.vs.WT.Negative_Science.2016.PMID.26912704	1.17	0.97	1.42	0.11	0.44
FOXC1.Hair.Follicles.P30C.LO.vs.WT.Positive_Science.2016.PMID.26912704	0.88	0.73	1.06	0.19	0.57
fSTR.Signature_Cell.Stem.Cell.2012.PMID.22305568	0.96	0.79	1.16	0.66	0.90
Gamma.delta.T.cell_CellRep.2017.PMID.28052254	0.98	0.81	1.18	0.84	0.97
GATA3.induced.genes_JCO.2006.PMID.16505416	1.10	0.91	1.34	0.31	0.71
GATA3.induced.genes_Oncogene.2004.PMID.15361840	1.00	0.84	1.20	>0.99	>0.99
GDF11.TGFBR3_Nat.Cell.Biol.2014.PMID.24658685	1.03	0.85	1.26	0.74	0.93
Glycolysis_BMC.Med.2009.PMID.19291283	1.10	0.89	1.37	0.37	0.76
GO.DOWN.with.SOX10.OE_Cell.Rep.2015.PMID.26365194	1.18	0.96	1.45	0.11	0.45
GO.UP.with.SOX10.OE_Cell.Rep.2015.PMID.26365194	1.03	0.85	1.25	0.73	0.93
GSEA_BIOCARTA_ALK_PATHWAY.PMID.16199517	1.06	0.88	1.28	0.55	0.86
GSEA_BIOCARTA.AKT.PATHWAY.PMID.16199517	0.92	0.77	1.11	0.38	0.77
GSEA_BIOCARTA.BRCA.ATR.PATHWAY.ATRBRCA.PMID.16199517	1.01	0.83	1.22	0.93	0.97
GSEA_BIOCARTA.CASPASE.PATHWAY.PMID.16199517	0.90	0.73	1.10	0.30	0.69
GSEA_BIOCARTA.CTLA4.PATHWAY.PMID.16199517	0.80	0.66	0.97	0.02	0.20
GSEA_BIOCARTA.IGF1R.PATHWAY.PMID.16199517	0.85	0.70	1.04	0.12	0.46
GSEA_BIOCARTA.MTOR.PATHWAY.PMID.16199517	0.93	0.76	1.13	0.45	0.80
GSEA_BIOCARTA.PTEN.PATHWAY.PMID.16199517	0.88	0.72	1.07	0.21	0.59
GSEA_BIOCARTA.RAS.PATHWAY.PMID.16199517	0.96	0.80	1.15	0.65	0.90
GSEA_BIOCARTA.RB.PATHWAY.PMID.16199517	1.02	0.84	1.23	0.86	0.97
GSEA_BIOCARTA.VEGF.PATHWAY.PMID.16199517	1.06	0.86	1.29	0.60	0.88
GSEA_HALLMARK.MYC.TARGETS.V1.PMID.16199517	1.24	1.02	1.50	0.03	0.21
GSEA_HELLER.HDAC.TARGETS.DOWN.PMID.16199517	0.82	0.67	1.02	0.07	0.36
GSEA_NELSON.RESPONSE.TO.ANDROGEN.UP.PMID.16199517	0.92	0.76	1.11	0.37	0.76
GSEA_REACTOME.PD1.SIGNALING.PMID.16199517	0.79	0.64	0.97	0.02	0.20
GSEA_REACTOME.PI3K.CASCADE.PMID.16199517	0.93	0.76	1.13	0.46	0.81
GSEA_RETINOL.METABOLISM.KEGG.PMID.16199517	0.90	0.74	1.09	0.27	0.68
GSEA.GP1_Proliferation.DNA.repair..PUJANA.CHEK2.PCC.NETWORK.PMID.25109877	1.02	0.84	1.22	0.87	0.97
GSEA.GP1_Proliferation.DNA.repair.REACTOME.CELL.CYCLE.MITOTIC.PMID.25109877	1.12	0.93	1.34	0.24	0.63
GSEA.GP10_Fatty.acid.oxidation.CARBOXYLIC.ACID.METABOLIC.PROCESS.PMID.25109877	0.88	0.73	1.06	0.17	0.54
GSEA.GP11_Immune.IFN.PerouLab.PMID.25109877	1.05	0.86	1.27	0.64	0.89
GSEA.GP12_Hypoxia.glycolosis.SEMENZA.HIF1.TARGETS.PMID.25109877	1.02	0.83	1.26	0.82	0.97
GSEA.GP13_Neural.signailng.MODULE100.PMID.25109877	0.97	0.80	1.18	0.78	0.96
GSEA.GP13_Neural.signailng.NERVOUS.SYSTEM.DEVELOPMENT.PMID.25109877	1.06	0.86	1.32	0.57	0.87
GSEA.GP14_Plasma.membrane.cell.cell.signaling.MORF.CNTN1.PMID.25109877	0.96	0.80	1.16	0.66	0.90

GSEA.GP15_EGF.signaling.NAGASHIMA.EGF.SIGNALING.UP.PMID.25109877	1.02	0.84	1.25	0.82	0.97
GSEA.GP16_Protein.kinase.signaling.MAPKs.INTRACELLULAR.SIGNALING.CASCADE.PMID.25109877	0.92	0.75	1.12	0.40	0.77
GSEA.GP16_Protein.kinase.signaling.MAPKs.REGULATION.OF.KINASE.ACTIVITY.PMID.25109877	1.13	0.92	1.38	0.23	0.62
GSEA.GP17_Basal.signaling.SMID.BREAST.CANCER.BASAL.UP.PMID.25109877	1.11	0.91	1.36	0.32	0.72
GSEA.GP18_Vesicle.EPR.MEMBRANE.COAT.PMID.25109877	1.00	0.81	1.24	0.99	>0.99
GSEA.GP19_1Q.amplicon.PerouLab.PMID.25109877	1.10	0.93	1.31	0.27	0.68
GSEA.GP2_Immune.Tcell.Bcell.KEGG.HEMATOPOIETIC.CELL.LINEAGE.PMID.25109877	0.76	0.63	0.93	0.01	0.19
GSEA.GP2_Immune.Tcell.Bcell.PerouLab.PMID.25109877	0.82	0.67	1.00	0.05	0.28
GSEA.GP20_TAL1.Leukemia.erythropoiesis.GNF2.TAL1.PMID.25109877	0.92	0.76	1.12	0.43	0.78
GSEA.GP21_Anti.apoptosis.DNA.stability.MORF.BCL2.PMID.25109877	1.05	0.85	1.30	0.66	0.90
GSEA.GP21_Anti.apoptosis.DNA.stability.MORF.MT4.PMID.25109877	0.99	0.81	1.22	0.95	0.98
GSEA.GP21_Anti.apoptosis.DNA.stability.MORF.STK17A.PMID.25109877	1.02	0.83	1.25	0.88	0.97
GSEA.GP22_16Q22.24.amplicon.PerouLab.PMID.25109877	1.01	0.84	1.21	0.91	0.97
GSEA.GP3_Tumo.suppressing.miRNA.targets.GTTTGTT.MIR.495.PMID.25109877	0.99	0.82	1.20	0.93	0.97
GSEA.GP3_Tumor.suppressing.miRNA.targets.DACOSTA.UV.RESPONSE.VIA.ERCC3.DN.PMID.25109877	1.01	0.84	1.22	0.89	0.97
GSEA.GP3_Tumor.suppressing.miRNA.targets.TGCTTTG.MIR.330.PMID.25109877	1.01	0.84	1.21	0.92	0.97
GSEA.GP4_MES.ECM.PerouLab.PMID.25109877	1.06	0.87	1.30	0.55	0.86
GSEA.GP5_MYC.targets.TERT.PerouLab.PMID.25109877	1.04	0.87	1.26	0.65	0.89
GSEA.GP6_Squamous.differentiation.development.RICKMAN.TUMOR.DIFFERENTIATED.WELL.VS.POORLY.DN.PMID.2510987	0.97	0.80	1.17	0.73	0.93
GSEA.GP7_Estrogen.signaling.SMID.BREAST.CANCER.BASAL.DN.PMID.25109877	0.96	0.78	1.19	0.72	0.93
GSEA.GP8_FOXO.stemness.MORF.PTPRB.PMID.25109877	0.97	0.80	1.17	0.74	0.93
GSEA.GP8_FOXO.stemness.TTGTTT.VSFOXO4.01.PMID.25109877	1.02	0.85	1.22	0.86	0.97
GSEA.GP9_Cell.cell.adhesion.PerouLab.PMID.25109877	1.16	0.95	1.41	0.16	0.52
HCK_BCR.2008.PMID.19272155	0.81	0.67	0.98	0.03	0.21
HER1.Cluster1_BMC.Genomics.2007.PMID.17663798	1.13	0.94	1.35	0.20	0.58
HER1.Cluster2_BMC.Genomics.2007.PMID.17663798	1.04	0.86	1.24	0.71	0.92
HER1.Cluster3_BMC.Genomics.2007.PMID.17663798	1.00	0.84	1.19	0.98	>0.99
HER2.Amplicon.PerouLab_BMC.Med.Genomic.2011.PMID.21214954	0.83	0.67	1.04	0.10	0.43
Histological.Grade_J.Pathol.2017.PMID.27861902	1.11	0.92	1.33	0.28	0.68
HouseKeeping_Genome.Biol.2004.PMID.15287981	1.00	0.83	1.21	0.98	>0.99
iDC.Median_Immunity.2013.PMID.24138885	0.92	0.76	1.11	0.40	0.77
IFN.Cluster_BMC.Med.Genomics.2011.PMID.21214954	1.16	0.96	1.41	0.12	0.47
IgG_BCR.2008.PMID.19272155	0.78	0.65	0.95	0.01	0.19
IGG.Cluster_BMC.Med.Genomics.2011.PMID.21214954	0.77	0.63	0.93	0.01	0.19
Immature..B.cell_CellRep.2017.PMID.28052254	0.79	0.64	0.97	0.02	0.20
Immature.dendritic.cell_CellRep.2017.PMID.28052254	1.05	0.86	1.29	0.60	0.89
ImmLandscape_Macro.mono.CSF1.core.response_CCR.2009.PMID.29628290	0.79	0.65	0.96	0.02	0.19
ImmLandscape_Wound.Healing_Immunity.2018.PMID.29628290	1.02	0.84	1.23	0.86	0.97
ImmLandscape.IFN3_Plos.One.2014.PMID.24516633	1.17	0.97	1.42	0.10	0.42
ImmLandscape.IFNG5_Plos.One.2014.PMID.24516633	0.79	0.65	0.96	0.02	0.19
ImmLandscape.lymphocyte.Infil.T.B.PMID.18592372	0.80	0.66	0.97	0.02	0.19
Immune.Hot.CD8.vs.Cold_Nature.2020.PMID.31942071	0.79	0.65	0.97	0.02	0.20
Immune.Perez.14_JCO.2015.PMID.25605861	0.79	0.66	0.96	0.02	0.19
Immune.Perez.87_JCO.2015.PMID.25605861	0.78	0.64	0.95	0.02	0.19
Immune.Suppression_JCI.Insight.2016.PMID.27699256	0.90	0.74	1.10	0.29	0.69
ImmuneActive_Cell.2019.PMID.31730857	0.79	0.65	0.96	0.02	0.19
Immunosuppression.PMID.31942077	1.00	0.83	1.21	0.98	>0.99
IMS.Score_CCR.2018.PMID.29921729	0.95	0.78	1.16	0.62	0.89
Induced.in.Bcells_PNAS.2013.PMID.23382184	0.84	0.69	1.02	0.08	0.37

Induced.in.DC_PNAS.2013.PMID.23382184	0.77	0.63	0.95	0.01	0.19
Induced.in.GN_PNAS.2013.PMID.23382184	0.91	0.75	1.10	0.33	0.72
Induced.in.HSC_PNAS.2013.PMID.23382184	1.14	0.94	1.37	0.19	0.56
Induced.in.MOs_PNAS.2013.PMID.23382184	0.88	0.72	1.08	0.23	0.62
Induced.in.NKcells_PNAS.2013.PMID.23382184	0.99	0.81	1.20	0.89	0.97
Induced.in.Tcells_PNAS.2013.PMID.23382184	0.90	0.74	1.09	0.28	0.68
Inflammatory.breast.cancer.491genes_CCR.2013.PMID.23396049	0.84	0.69	1.03	0.09	0.40
Inflammatory.breast.cancer.79genes_CCR.2013.PMID.23396049	1.08	0.88	1.32	0.47	0.81
Inflammatory.breast.cancer.expressed.noIBC_79genes_CCR.2013.PMID.23396049	1.02	0.84	1.23	0.84	0.97
Inflammatory.breast.cancer.expressed.noIBC.491genes_CCR.2013.PMID.23396049	1.02	0.83	1.24	0.86	0.97
Influenza.11genes.Metasignature_Immunity.2015.PMID.26682989	1.07	0.88	1.29	0.51	0.85
Interferon_BCR.2008.PMID.19272155	1.22	1.01	1.47	0.04	0.26
Interferon.Pathway_CancerImmunolRes.2018.PMID.30266715	1.15	0.95	1.39	0.16	0.53
JUND.KRT5_Nat.Cell.Biol.2014.PMID.24658685	1.02	0.83	1.24	0.87	0.97
Keller2012.CD10.Adam_BCR.2015.PMID.25575446	1.15	0.95	1.40	0.15	0.50
KRAS.amplicon_Genome.Biology.2007.PMID.17493263	1.00	0.83	1.21	0.99	>0.99
Late.IRS.1_PLoS.One.2016.PMID.26991655	1.16	0.96	1.39	0.12	0.46
Late.IRS.2_PLoS.One.2016.PMID.26991655	1.06	0.88	1.28	0.55	0.87
LCK_BCR.2008.PMID.19272155	0.78	0.64	0.95	0.01	0.19
Lim2009.LumProg.Adam_BCR.2015.PMID.25575446	1.11	0.91	1.36	0.31	0.71
Lim2009.MaSC.Adam_BCR.2015.PMID.25575446	0.95	0.78	1.17	0.64	0.89
Lim2009.MatureLum.Adam_BCR.2015.PMID.25575446	1.07	0.86	1.33	0.54	0.86
Lim2009.Stroma.Adam_BCR.2015.PMID.25575446	0.98	0.81	1.19	0.86	0.97
Lim2010.LumProg.Adam_BCR.2015.PMID.25575446	1.00	0.82	1.22	0.98	>0.99
Lim2010.MaSC.Adam_BCR.2015.PMID.25575446	1.09	0.89	1.33	0.41	0.78
Lim2010.MatureLum.Adam_BCR.2015.PMID.25575446	1.01	0.83	1.23	0.92	0.97
Lim2010.Stroma.Adam_BCR.2015.PMID.25575446	0.96	0.79	1.16	0.65	0.90
Lobular.Carcinoma.In.Situ_J.Pathol.2017.PMID.27861902	1.11	0.92	1.36	0.28	0.68
LOBULAR.TCGA.SIGNATURE.ImmuneCell.2015.PMID.26451490	0.81	0.66	0.98	0.03	0.22
LOBULAR.TCGA.SIGNATURE.Reactive_Cell.2015.PMID.26451490	1.06	0.87	1.30	0.57	0.87
LOBULAR.TCGA.SUBTYPE.Immune_Cell.2015.PMID.26451490	0.87	0.71	1.07	0.19	0.56
LOBULAR.TCGA.SUBTYPE.Proliferative_Cell.2015.PMID.26451490	0.95	0.78	1.15	0.58	0.88
LOBULAR.TCGA.SUBTYPE.Reactive_Cell.2015.PMID.26451490	0.99	0.81	1.21	0.92	0.97
LTS.score_JCI.2020.PMID.32573490	0.97	0.80	1.18	0.78	0.96
Luminal_Progenitor_Up_Nat.Med.2009.PMID.19648928	1.03	0.83	1.26	0.81	0.97
Luminal.cluster_BMC.Med.Genomics.2011.PMID.21214954	0.96	0.78	1.19	0.72	0.93
Luminal.Progenitor_BCR.2010.PMID.20346151	0.93	0.75	1.15	0.51	0.85
Luminal.Progenitor.Down_Nat.Med.2009.PMID.19648928	1.10	0.90	1.34	0.36	0.76
LumProg.HsEnriched_BCR.2015.PMID.25575446	1.00	0.81	1.23	>0.99	>0.99
LumProg.HsEnriched.Refined1_BCR.2015.PMID.25575446	0.98	0.80	1.21	0.89	0.97
LumProg.Lim09_BCR.2015.PMID.25575446	1.08	0.88	1.31	0.46	0.81
LumProg.Prat_BCR.2015.PMID.25575446	1.05	0.87	1.27	0.62	0.89
LumProg.Shehata_BCR.2015.PMID.25575446	1.04	0.86	1.24	0.71	0.92
Lums.HER2E.DOWN.metastatic.signature_JCI.2020.PMID.32573490	1.17	0.94	1.46	0.15	0.52
Lums.HER2E.UP.metastatic.signature_JCI.2020.PMID.32573490	0.93	0.76	1.13	0.46	0.81
Lung.WNT_Cancer.Res.2009.PMID.19549913	0.99	0.81	1.21	0.93	0.97
Lymph.vessels_Immunity.2013.PMID.24138885	0.97	0.79	1.18	0.73	0.93
Lymphovascular.Invasion_J.Pathol.2017.PMID.27861902	0.99	0.82	1.20	0.95	0.98
M.D.Metagene_Genome.Biol.2013.PMID.23618380	0.77	0.64	0.93	0.01	0.19

M2.Macrophage_Blood.2006.PMID.16556895	0.83	0.68	1.00	0.06	0.30
Macrophage_CellRep.2017.PMID.28052254	0.82	0.67	0.99	0.04	0.26
Macrophages_CancerImmunolRes.2018.PMID.30266715	0.81	0.66	0.98	0.03	0.21
Macrophages_Immunity.2013.PMID.24138885	0.92	0.75	1.12	0.38	0.77
Macrophages.M0_Nat.Methods.2015.PMID.25822800	0.83	0.68	1.03	0.09	0.39
Macrophages.M1_Nat.Methods.2015.PMID.25822800	0.88	0.72	1.06	0.18	0.56
Macrophages.M2_Nat.Methods.2015.PMID.25822800	0.79	0.65	0.96	0.02	0.19
MacTh1.cluster_CCR.2014.PMID.24916698	0.79	0.65	0.96	0.02	0.19
MammaPrint_Nature.2002.PMID.11823860	0.90	0.73	1.10	0.31	0.71
MAPK.pathway.activation_NPJ.Precis.Oncol.2018.PMID.29872725	1.30	1.06	1.59	0.01	0.19
MASC.Down_Nat.Med.2009.PMID.19648928	1.05	0.87	1.27	0.62	0.89
MASC.Up_Nat.Med.2009.PMID.19648928	1.09	0.89	1.34	0.40	0.77
Mast.cell_CellRep.2017.PMID.28052254	0.82	0.68	0.99	0.04	0.26
Mast.cells_Immunity.2013.PMID.24138885	0.93	0.77	1.12	0.43	0.78
Mast.cells.activated_Nat.Methods.2015.PMID.25822800	0.89	0.73	1.07	0.22	0.60
Mast.cells.resting_Nat.Methods.2015.PMID.25822800	0.84	0.70	1.01	0.07	0.35
Mature.luminal_BCR.2010.PMID.20346151	1.03	0.84	1.27	0.75	0.94
Mature.Luminal.Down_Nat.Med.2009.PMID.19648928	1.02	0.83	1.27	0.83	0.97
Mature.LuminaUp_Nat.Med.2009.PMID.19648928	0.99	0.80	1.23	0.94	0.98
MatureLum.HsEnriched_BCR.2015.PMID.25575446	0.94	0.76	1.16	0.56	0.87
MatureLum.HsEnriched.Refined1_BCR.2015.PMID.25575446	0.95	0.78	1.17	0.65	0.89
MatureLum.Lim09_BCR.2015.PMID.25575446	0.98	0.79	1.21	0.86	0.97
MatureLum.Prat_BCR.2015.PMID.25575446	1.00	0.82	1.22	0.99	>0.99
MatureLum.Shehata_BCR.2015.PMID.25575446	1.10	0.90	1.34	0.37	0.76
MBasal.Cluster_BMC.Med.Genomics.2011.PMID.21214954	1.08	0.89	1.33	0.43	0.78
MCD3.CD8_BMC.Med.Genomics.2011.PMID.21214954	0.80	0.66	0.97	0.02	0.20
MCF7.E2.induced.genes_JCO.2006.PMID.16505416	0.96	0.80	1.15	0.66	0.90
MCF7.E2.repressed.genes_JCO.2006.PMID.16505416	0.94	0.77	1.15	0.54	0.86
MDSC_CellRep.2017.PMID.28052254	0.80	0.65	0.97	0.02	0.20
MDSC.Granulocytic_Leukoc.Biol.2012.PMID.21954284	1.01	0.83	1.23	0.91	0.97
MDSC.Neutrophi_Leukoc.Biol.2012.PMID.21954284	0.87	0.71	1.06	0.15	0.52
MDSC.tumor_J.Immunol.2012.PMID.23152559	0.91	0.74	1.12	0.38	0.77
MDSC.tumor.MO_J.Immunol.2012.PMID.23152559	0.94	0.77	1.16	0.59	0.88
MECM_BMC.Med.Genomics.2011.PMID.21214954	1.01	0.84	1.23	0.90	0.97
Memory.B.cell_CellRep.2017.PMID.28052254	1.23	1.00	1.50	0.05	0.27
MET.DOWN.RNAseq.Significant.Genes_JCI.2018.PMID.29480819	0.96	0.80	1.14	0.60	0.89
MET.DOWN.Significant.Genes.Low.Basal.1_JCI.2018.PMID.29480819	1.11	0.91	1.35	0.32	0.72
MET.DOWN.Significant.Genes.Low.Basal.2_JCI.2018.PMID.29480819	0.96	0.80	1.15	0.64	0.89
MET.UP.RNAseq.Significant.Genes_JCI.2018.PMID.29480819	1.09	0.90	1.32	0.37	0.77
MET.UP.Significant.Genes.HIGH.BASALS.Genes_JCI.2018.PMID.29480819	0.94	0.77	1.15	0.54	0.86
Metaplastic.Up_CanRes.2009.PMID.19435916	1.01	0.83	1.23	0.92	0.97
Metastasis.predictor.TNBC_BCR.2010.PMID.20946665	0.80	0.66	0.96	0.02	0.19
MFGFR2_BMC.Med.Genomics.2011.PMID.21214954	1.15	0.93	1.42	0.19	0.57
MHC.Forero.11_Cancer.Immunol.Res.2016.PMID.26980599	0.79	0.64	0.97	0.03	0.20
MHC.Forero.24_Cancer.Immunol.Res.2016.PMID.26980599	0.84	0.70	1.00	0.05	0.29
MHC.I_BCR.2008.PMID.19272155	0.99	0.82	1.21	0.96	0.98
MHC.II_BCR.2008.PMID.19272155	0.74	0.61	0.90	0.00	0.19
MHCI.coreGenes_Nat.Commun.2017.PMID29170503	1.01	0.83	1.23	0.91	0.97
MIR200c.Induced_ONCO.2015.PMID.25746005	1.03	0.85	1.25	0.78	0.96

MIR200c.Repressed_ONCO.2015.PMID.25746005	1.09	0.88	1.35	0.42	0.78
miRNA.138.signature_Cancer.Res.2014.PMID.25339353	0.98	0.81	1.19	0.87	0.97
MITO1_BMC.Med.Genomics.2011.PMID.21214954	0.95	0.78	1.14	0.56	0.87
MITO2_BMC.Med.Genomics.2011.PMID.21214954	0.91	0.76	1.08	0.26	0.66
Mitotic.Count_J.Pathol.2017.PMID.27861902	1.24	1.01	1.51	0.04	0.25
MK14.K17_BMC.Med.Genomics.2011.PMID.21214954	1.12	0.93	1.35	0.23	0.62
MKRAS.amplicon_BMC.Med.Genomics.2011.PMID.21214954	0.95	0.78	1.15	0.59	0.88
MM.BRCAwnt.1pFDR.UP_Genome.Biology.2007.PMID.17493263	1.17	0.96	1.43	0.12	0.46
MM.C3Tag.1pFDR.UP_Genome.Biology.2007.PMID.17493263	1.20	0.99	1.45	0.07	0.35
MM.C3Tag.2012_Genome.Biol.2013.PMID.24220145	1.12	0.93	1.35	0.22	0.60
MM.Class3_Genome.Biol.2013.PMID.24220145	1.04	0.86	1.26	0.71	0.92
MM.Class8_Genome.Biol.2013.PMID.24220145	1.06	0.87	1.29	0.58	0.87
MM.Claudinlow_Genome.Biol.2013.PMID.24220145	0.92	0.76	1.12	0.41	0.78
MM.DMBAwnt.1pFDR.UP.Genome.Biology.2007.PMID.17493263	1.14	0.93	1.39	0.21	0.59
MM.Erbb2.like_Genome.Biol.2013.PMID.24220145	1.09	0.89	1.32	0.40	0.77
MM.Myc.2012_Genome.Biol.2013.PMID.24220145	1.05	0.88	1.26	0.60	0.88
MM.Myoepithelioma.like_Genome.Biol.2013.PMID.24220145	1.06	0.86	1.29	0.59	0.88
MM.Neu.2012_Genome.Biol.2013.PMID.24220145	1.06	0.88	1.29	0.52	0.85
MM.NeuPyMT.1pFDR.UP_Genome.Biology.2007.PMID.17493263	0.99	0.82	1.19	0.88	0.97
MM.Normal.1pFDR.UP_Genome.Biology.2007.PMID.17493263	0.90	0.74	1.09	0.30	0.69
MM.Normal.like_Genome.Biol.2013.PMID.24220145	0.94	0.78	1.14	0.52	0.85
MM.p53null.1pFDR.UP_Genome.Biology.2007.PMID.17493263	0.95	0.78	1.16	0.63	0.89
MM.p53null.Basal_Genome.Biol.2013.PMID.24220145	1.19	0.97	1.46	0.10	0.42
MM.p53null.Luminal_Genome.Biol.2013.PMID.24220145	1.01	0.84	1.23	0.88	0.97
MM.Potluck_1pFDR_UP_Genome.Biology.2007_PMID.17493263.PMID.24220145	0.89	0.73	1.08	0.24	0.65
MM.PyMT.2012_Genome.Biol.2013.PMID.24220145	0.97	0.80	1.17	0.76	0.94
MM.Squamous.like_Genome.Biol.2013.PMID.24220145	1.08	0.89	1.32	0.43	0.78
MM.Stat1_Genome.Biol.2013.PMID.24220145	0.99	0.81	1.20	0.90	0.97
MM.WapINT3.1pFDR.UP_Genome.Biology.2007.PMID.17493263	1.13	0.93	1.37	0.20	0.59
MM.WapINT3.2012_Genome.Biol.2013.PMID.24220145	1.06	0.88	1.29	0.52	0.85
MM.WAPTag.1pFDR.UP_Genome.Biology.2007.PMID.17493263	1.17	0.97	1.41	0.10	0.43
MM.Wnt1.Early_Genome.Biol.2013.PMID.24220145	1.12	0.91	1.37	0.28	0.68
MM.Wnt1.Late_Genome.Biol.2013.PMID.24220145	1.17	0.95	1.44	0.14	0.48
Mmyosin_BMC.Med.Genomics.2011.PMID.21214954	0.94	0.76	1.16	0.55	0.86
MNADH_CYTochrome_BMC.Med.Genomics.2011.PMID.21214954	0.87	0.73	1.04	0.14	0.49
MNB1_BMC.Med.Genomics.2011.PMID.21214954	0.83	0.69	1.01	0.06	0.30
MNB2_BMC.Med.Genomics.2011.PMID.21214954	0.88	0.72	1.08	0.23	0.62
MNB3_BMC.Med.Genomics.2011.PMID.21214954	0.85	0.68	1.07	0.18	0.56
MNOtch4_BMC.Med.Genomics.2011.PMID.21214954	1.21	0.99	1.48	0.07	0.35
Monocyte_CellRep.2017.PMID.28052254	0.80	0.65	0.98	0.03	0.21
Monocyte..DC.25gene_Genome.Biol.2013.PMID.23618380	0.78	0.64	0.95	0.01	0.19
Monocytes_CancerImmunolRes.2018.PMID.30266715	0.80	0.66	0.97	0.02	0.19
Monocytes_Nat.Methods.2015.PMID.25822800	0.80	0.66	0.98	0.03	0.21
Monocytic.lineage.MCP_Nature.2020.PMID.31942075	0.85	0.69	1.04	0.12	0.47
MProliferation_BMC.Med.Genomics.2011.PMID.21214954	1.06	0.88	1.28	0.51	0.85
MProtocadherin_BMC.Med.Genomics.2011.PMID.21214954	0.82	0.68	1.00	0.05	0.28
MPYMT_NEU_Cluster_BMC.Med.Genomics.2011.PMID.21214954	0.87	0.72	1.04	0.12	0.46
MRibosomal_BMC.Med.Genomics.2011.PMID.21214954	0.99	0.81	1.20	0.89	0.97
MS.CD44.DOWN_PNAS.2009.PMID.19666588	1.14	0.94	1.39	0.17	0.54

MS.CD44.UP_PNAS.2009.PMID.19666588	0.94	0.78	1.14	0.54	0.86
MSquamous_BMC.Med.Genomics.2011.PMID.21214954	1.03	0.85	1.24	0.79	0.96
Murat.G07_JCO.2008.PMID.18565887	1.13	0.92	1.38	0.25	0.65
Murat.G18_JCO.2008.PMID.18565887	1.12	0.92	1.37	0.26	0.66
Murat.G24_JCO.2008.PMID.18565887	0.78	0.63	0.95	0.01	0.19
MVEGFC_BMC.Med.Genomics.2011.PMID.21214954	0.97	0.80	1.18	0.79	0.96
Myeloid.cell.chemotaxis.1gene_Nature.2020.PMID.31942077	0.93	0.77	1.13	0.48	0.82
Myeloid.dendritic.cells.MCP_Nature.2020.PMID.31942077	0.72	0.60	0.87	0.00	0.19
Natural.killer.cell_CellRep.2017.PMID.28052254	0.91	0.75	1.10	0.33	0.72
Natural.killer.T.cell_CellRep.2017.PMID.28052254	0.87	0.72	1.05	0.14	0.49
Necrosis_J.Pathol.2017.PMID.27861902	1.09	0.90	1.33	0.36	0.76
Neutrophil_CellRep.2017.PMID.28052254	0.87	0.71	1.06	0.17	0.54
Neutrophils_CancerImmunolRes.2018.PMID.30266715	0.92	0.76	1.12	0.40	0.77
Neutrophils_Immunity.2013.PMID.24138885	0.87	0.71	1.06	0.16	0.52
Neutrophils_Nat.Methods.2015.PMID.25822800	0.81	0.67	0.98	0.03	0.21
Neutrophils.MCP_Nature.2020.PMID.31942077	0.98	0.81	1.19	0.86	0.97
NK_Immunity.2013.PMID.24138885	1.03	0.84	1.26	0.79	0.96
NK.activated_Nat.Methods.2015.PMID.25822800	0.79	0.65	0.96	0.02	0.19
NK.CD56bright_Immunity.2013.PMID.24138885	0.96	0.80	1.17	0.71	0.93
NK.CD56dim_Immunity.2013.PMID.24138885	0.83	0.68	1.00	0.05	0.29
NK.resting_Nat.Methods.2015.PMID.25822800	0.79	0.65	0.96	0.02	0.19
NKcells_CancerImmunolRes.2018.PMID.30266715	0.76	0.62	0.92	0.00	0.19
NKcells.MCP_Nature.2020.PMID.31942077	0.81	0.66	0.98	0.03	0.21
No.Response.Immunotherapy.TLS.Melanoma_Nature.2020.PMID.31942075	1.12	0.91	1.37	0.28	0.68
Normal.mucosa_Immunity.2013.PMID.24138885	1.05	0.88	1.27	0.57	0.87
Nuclear.Pleomorphism_J.Pathol.2017.PMID.27861902	1.05	0.87	1.26	0.64	0.89
Oncotype_NEJM.2004.PMID.15591335	1.08	0.88	1.33	0.47	0.81
P53.ERPos.MDACC_CCR.2011.PMID.21248301	1.10	0.91	1.32	0.33	0.72
Parity.signature.251genes_BCR.2014.PMID.25005139	0.79	0.65	0.96	0.02	0.19
Parity.signature.40genes_BCR.2014.PMID.25005139	0.79	0.65	0.96	0.02	0.19
PARPi.Resistance_BCRT_2012.PMID.22875744	0.97	0.80	1.18	0.79	0.96
PARPi.Sensitivity_BCRT_2012.PMID.22875744	0.99	0.81	1.21	0.94	0.98
PARPi.Sensitivity.MDACC_NPJ.Syst.Biol.Appl.2017.PMID.28649435	1.00	0.82	1.22	0.98	>0.99
PARPi.Sensitivity.Negative_Sci.Adv.2017.PMID.28439535	1.12	0.92	1.36	0.27	0.68
PARPi.Sensitivity.Positive_Sci.Adv.2017.PMID.28439535	1.02	0.84	1.25	0.81	0.97
Pcorr.Breast2Lung.LM2.Correlation_Nature.2005.PMID.16049480	0.99	0.80	1.22	0.91	0.97
Pcorr.Breast2Lung.Parental.Correlation_Nature.2005.PMID.16049480	1.01	0.82	1.25	0.92	0.97
Pcorr.dasatinib.resistant_Cancer.Res.2007.PMID.17332353	0.98	0.79	1.21	0.85	0.97
Pcorr.dasatinib.sensitive_Cancer.Res.2007.PMID.17332353	1.03	0.83	1.28	0.78	0.96
Pcorr.Hypoxia.High.Correlation_PLoS.Med.2006.PMID.16417408	1.05	0.85	1.30	0.66	0.90
Pcorr.Hypoxia.Low.Correlation_PLoS.Med.2006.PMID.16417408	0.97	0.78	1.21	0.80	0.96
Pcorr.IGS_Invasiveness_NJEM.2007.PMID.17229949	0.99	0.81	1.20	0.90	0.97
Pcorr.wound.response.activated_PNAS.2005.PMID.15701700	1.07	0.89	1.28	0.47	0.81
pCR.predictor.ERNeg.55genes_JAMA.2011.PMID.21558518	1.12	0.93	1.36	0.23	0.62
pCR.predictor.ERPos.39genes_JAMA.2011.PMID.21558518	1.05	0.86	1.27	0.64	0.89
PDCD1_Single_Gene.Single	0.84	0.70	1.01	0.06	0.32
Pfefferle2012.LumProg_BCR.2015.PMID.25575446	0.89	0.71	1.11	0.30	0.69
Pfefferle2012.MaSC_BCR.2015.PMID.25575446	1.09	0.90	1.33	0.38	0.77
Pfefferle2012.MatureLum_BCR.2015.PMID.25575446	1.09	0.89	1.35	0.40	0.77

Pfefferle2012.Stroma_BCR.2015.PMID.25575446	0.98	0.80	1.19	0.81	0.97
PGR_Single_Gene.Single	0.98	0.77	1.25	0.89	0.97
PI3Ki.Down_CancerCell.2017.PMID.28528867	0.97	0.81	1.17	0.76	0.95
PI3Ki.Up_CancerCell.2017.PMID.28528867	1.01	0.83	1.23	0.92	0.97
PIK3CA.Pathway_Ann.Oncol.2017.PMID.28177460	0.95	0.78	1.16	0.64	0.89
PIK3CAmt.signature_Cancer.Res.2012.PMID.22552288	1.08	0.89	1.30	0.44	0.80
Plasma.cells_Nat.Methods.2015.PMID.25822800	0.81	0.67	0.99	0.04	0.26
PlasmaCells_CancerImmunolRes.2018.PMID.30266715	0.86	0.71	1.05	0.14	0.50
Plasmacytoid.dendritic.cell_CellRep.2017.PMID.28052254	0.85	0.70	1.02	0.07	0.36
PR.Isoform.Ratio.Up.in.PRA.H_JNCI.2017.PMID.28376177	0.93	0.76	1.13	0.45	0.80
PR.Isoform.Ratio.Up.in.PRB.H_JNCI.2017.PMID.28376177	1.18	0.97	1.44	0.10	0.43
Proliferation.Cluster_BMC.Med.Genomics.2011.PMID.21214954	1.11	0.92	1.34	0.29	0.69
Proliferation.Metagene_Genome.Biol.2013.PMID.23618380	1.07	0.89	1.29	0.44	0.80
Proliferation.score.PAM50_JCO.2009.PMID.19204204	1.05	0.86	1.28	0.63	0.89
ProliferationPathway_CancerImmunolRes.2018.PMID.30266715	1.07	0.89	1.29	0.46	0.80
Prosigna.Proliferation.18_BMC.Med.Genomics.2015.PMID.26297356	1.10	0.92	1.32	0.31	0.71
Race.LuminalA.MRE.score_BCRT.2015.PMID.26109344	1.05	0.86	1.28	0.61	0.89
Radiation.induced.genes_Radoat.Res.2014.PMID.24527691	1.10	0.89	1.36	0.38	0.77
RB.LOH_BCR.2008.PMID.18782450	1.10	0.92	1.32	0.28	0.68
RB.LOSS_JCI.2007.PMID.17160137	1.07	0.89	1.29	0.48	0.82
Regulatory.T.cell_CellRep.2017.PMID.28052254	0.87	0.72	1.06	0.17	0.54
Replication.Stress.Down.set_Cell.Rep.2018.PMID.29768207	1.00	0.82	1.20	0.96	0.98
Replication.Stress.Model_Cell.Rep.2018_PMID.29768207.PMID.29768207	1.00	0.82	1.23	>0.99	>0.99
Replication.Stress.Neg_Cell.Rep.2018_PMID.29768207.PMID.29768207	0.98	0.81	1.19	0.87	0.97
Replication.Stress.Pos_Cell.Rep.2018_PMID.29768207.PMID.29768207	1.08	0.89	1.32	0.42	0.78
Replication.Stress.Up_Set_Cell.Rep.2018_PMID.29768207.PMID.29768207	1.09	0.89	1.33	0.42	0.78
Residual.disease.predictor.ERNeg.54genes_JAMA.2011.PMID.21558518	1.06	0.87	1.29	0.56	0.87
Residual.disease.predictor.ERPos.73genes_JAMA.2011.PMID.21558518	0.97	0.80	1.17	0.73	0.93
Response.Immunotherapy.MCP.TLS.Melanoma_Nature.2020.PMID.31942075	0.75	0.61	0.93	0.01	0.19
Response.Immunotherapy.signature_Science.2018.PMID.30309915	0.80	0.66	0.98	0.03	0.21
Response.Neo.Chemo_common_CCR.2014.PMID.25047707	1.10	0.90	1.33	0.35	0.74
Response.Neo.Chemo_ERNeg_CCR.2014.PMID.25047707	1.11	0.92	1.33	0.29	0.69
Response.Neo.Chemo_ERPos_CCR.2014.PMID.25047707	1.11	0.91	1.36	0.28	0.68
RHOA.pathway_Ann.Oncol.2017.PMID.28177460	1.14	0.94	1.39	0.19	0.56
Ribosomal.Cluster_BMC.Med.Genomics.2011.PMID.21214954	0.96	0.79	1.16	0.68	0.91
ROR.subtype.PAM50_JCO.2009.PMID.19204204	1.10	0.88	1.38	0.41	0.78
ROR.subtype.proliferation.PAM50_JCO.2009.PMID.19204204	1.06	0.87	1.30	0.57	0.87
RSS.Score_CCR.2018.PMID.29921729	1.02	0.84	1.22	0.86	0.97
S100A9.A8_BMC.Med.Genomics.2011.PMID.21214954	1.05	0.83	1.32	0.67	0.90
Scorr.EMAT1.Correlation_BCR.2020.PMID.32641077	0.88	0.72	1.08	0.21	0.60
Scorr.EMAT2.Correlation_BCR.2020.PMID.32641077	1.17	0.95	1.43	0.14	0.50
Scorr.EMAT3.Correlation_BCR.2020.PMID.32641077	1.08	0.88	1.31	0.46	0.81
Scorr.EMAT4.Correlation_BCR.2020.PMID.32641077	0.97	0.77	1.22	0.79	0.96
Scorr.IE.Correlation_JCO.2006.PMID.16505416	0.97	0.79	1.19	0.77	0.95
Scorr.IIE.Correlation_JCO.2006.PMID.16505416	1.02	0.83	1.25	0.86	0.97
Scorr.PAM50.Basal_JCO.2009.PMID.19204204	1.13	0.88	1.45	0.32	0.72
Scorr.PAM50.Her2_JCO.2009.PMID.19204204	1.07	0.86	1.34	0.55	0.86
Scorr.PAM50.LumA_JCO.2009.PMID.19204204	0.88	0.69	1.12	0.29	0.69
Scorr.PAM50.LumB_JCO.2009.PMID.19204204	0.98	0.81	1.19	0.87	0.97

Scorr.PAM50.Normal_JCO.2009.PMID.19204204	0.97	0.80	1.17	0.74	0.93
Scorr.S329.L_Br.J.Cancer.2008.PMID.18382427	1.01	0.82	1.25	0.92	0.97
Scorr.S329.R_Br.J.Cancer.2008.PMID.18382427	0.94	0.75	1.17	0.58	0.87
Secretoglobin_BMC.Med.Genomics.2011.PMID.21214954	0.96	0.79	1.17	0.67	0.90
Shehata2012.ALDHneg_BCR.2015.PMID.25575446	1.08	0.89	1.32	0.44	0.79
Shehata2012.ALDHpos_BCR.2015.PMID.25575446	0.91	0.73	1.13	0.38	0.77
Shehata2012.Basal_BCR.2015.PMID.25575446	1.02	0.84	1.24	0.85	0.97
Shehata2012.Erbb3neg_BCR.2015.PMID.25575446	0.95	0.79	1.15	0.61	0.89
Shehata2012.LumProg_BCR.2015.PMID.25575446	1.05	0.86	1.28	0.62	0.89
Shehata2012.NCL_BCR.2015.PMID.25575446	1.05	0.86	1.29	0.62	0.89
Shehata2012.Stroma_BCR.2015.PMID.25575446	0.98	0.81	1.19	0.85	0.97
Spike2012.aMaSC_BCR.2015.PMID.25575446	1.09	0.89	1.33	0.39	0.77
Spike2012.fMaSC_BCR.2015.PMID.25575446	0.91	0.74	1.10	0.32	0.72
Spike2012.fStr_BCR.2015.PMID.25575446	0.92	0.76	1.11	0.38	0.77
STAT1_BCR.2008.PMID.19272155	0.91	0.74	1.11	0.34	0.74
STAT3.Basal_PNAS.2014.PMID.25139989	0.82	0.67	1.00	0.05	0.29
STAT3.Basal.short_PNAS.2014.PMID.25139989	0.82	0.68	1.00	0.05	0.28
Stroma.FNA.MDACC.1_JCO.2010.PMID.20805453	0.82	0.67	1.00	0.05	0.27
Stroma.FNA.MDACC.2_JCO.2010.PMID.20805453	1.03	0.84	1.25	0.80	0.96
Stromal.Central.Fibrotic.Focus_J.Pathol.2017.PMID.27861902	0.97	0.79	1.19	0.75	0.94
Stromal.Down_Nat.Med.2009.PMID.19648928	1.11	0.93	1.34	0.25	0.66
Stromal.Inflammation_J.Pathol.2017.PMID.27861902	0.78	0.63	0.95	0.01	0.19
Stromal.Signature_Nat.Med.2008.PMID.18438415	0.81	0.67	0.98	0.03	0.21
Stromal.Up_Nat.Med.2009.PMID.19648928	1.00	0.82	1.21	0.96	0.98
SW480.cancer.cells_Immunity.2013.PMID.24138885	1.33	1.08	1.65	0.01	0.19
T.follicular.helper.cell_CellRep.2017.PMID.28052254	0.87	0.72	1.07	0.18	0.56
Tcell.activation_Nature.2020.PMID.31942077	0.92	0.76	1.10	0.35	0.75
Tcell.CD8.Effector.vs.naive.2_Science.2016.PMID27789795	1.07	0.89	1.28	0.49	0.83
Tcell.CD8.Exhausted.vs.antiPDL1.2_Science.2016.PMID27789795	1.10	0.91	1.33	0.34	0.73
Tcell.CD8.Exhausted.vs.naive.2_Science.2016.PMID27789795	1.05	0.87	1.26	0.61	0.89
Tcell.CD8.Memory.vs.naive.1_Science.2016.PMID27789795	0.86	0.71	1.04	0.12	0.47
Tcell.cluster_CCR.2014.PMID.24916698	0.78	0.64	0.94	0.01	0.19
Tcell.EXH.Anti.PDL1.vs.control.treated.exhausted.CD8.Tcell.Metagene.1.Science.2016.PMID.27789795	1.02	0.84	1.24	0.82	0.97
Tcell.EXH.Effector.CD8.T.cell.at.day.8.p.i.Armstrong.vs.Naive.CD8.Tcell.Metagene.1_Science.2016.PMID.27789795	0.87	0.71	1.06	0.16	0.53
Tcell.EXH.Exhausted.CD8.T.cell.vs.Naive.CD8.T.cell.Metagene.1_Science.2016.PMID.27789795	0.96	0.79	1.18	0.73	0.93
Tcell.EXH.Exhausted.CD8.T.cell.vs.Naive.CD8.T.cell.Metagene.3_Science.2016.PMID.27789795	0.86	0.71	1.04	0.13	0.47
Tcell.EXH.Memory.CD8.T.cell.a.vs.Naive.CD8.T.cell.Metagene.1_Science.2016.PMID.27789795	0.86	0.71	1.04	0.12	0.47
Tcell.EXH.Memory.CD8.T.cell.a.vs.Naive.CD8.T.cell.Metagene.2_Science.2016.PMID.27789795	0.89	0.73	1.09	0.27	0.68
Tcell.EXH.Memory.CD8.T.cell.a.vs.Naive.CD8.T.cell.Metagene.3.Science_2016.PMID.27789795	0.86	0.70	1.06	0.17	0.54
Tcell.NK.51gene_Genome.Biol.2013.PMID.23618380	0.77	0.64	0.94	0.01	0.19
Tcell.NK.Metagene_Genome.Biol.2013.PMID.23618380	0.77	0.64	0.94	0.01	0.19
Tcell.RM_Nat_Med.2018.PMID.29942092	0.84	0.69	1.03	0.10	0.42
Tcell.survival.2gene_Nature.2020.PMID.31942077	0.85	0.71	1.01	0.07	0.35
Tcells_CancerImmunolRes.2018.PMID.30266715	0.78	0.64	0.96	0.02	0.19
Tcells_Immunity.2013.PMID.24138885	0.80	0.66	0.97	0.02	0.20
Tcells_TFH_Nat.Methods.2015.PMID.25822800	0.84	0.69	1.02	0.08	0.36
Tcells.CD4.memory.activated_Nat.Methods.2015.PMID.25822800	0.81	0.67	0.99	0.03	0.23
Tcells.CD4.memory.resting_Nat.Methods.2015.PMID.25822800	0.81	0.67	0.98	0.03	0.23
Tcells.CD4.naive_Nat.Methods.2015.PMID.25822800	0.82	0.68	0.99	0.04	0.26

Tcells.CD8_Immunity.2013.PMID.24138885	0.89	0.74	1.07	0.23	0.62
Tcells.CD8_Nat.Methods.2015.PMID.25822800	0.81	0.67	0.98	0.03	0.22
Tcells.CD8.MCP_Nature.2020.PMID.31942075	0.88	0.72	1.07	0.19	0.56
Tcells.Cytotoxic.MCP_Nature.2020.PMID.31942075	0.85	0.69	1.04	0.11	0.45
Tcells.gammadelta_Nat.Methods.2015.PMID.25822800	0.79	0.65	0.96	0.02	0.19
Tcells.helper_Immunity.2013.PMID.24138885	0.97	0.79	1.18	0.73	0.93
Tcells.MCP_Nature.2020.PMID.31942077	0.79	0.66	0.96	0.02	0.19
Tcells.regulatory.2gene_Nature.2020.PMID.31942077	0.98	0.81	1.18	0.81	0.97
Tcells.Tregs_Nat.Methods.2015.PMID.25822800	0.80	0.66	0.98	0.03	0.21
TCGA.BRCA.1198_BASAL_JCI.2020.PMID.32573490	1.01	0.83	1.24	0.91	0.97
TCGA.BRCA.1198_Chromogranin_JCI.2020.PMID.32573490	1.01	0.83	1.22	0.95	0.98
TCGA.BRCA.1198_COLLAGEN11A_JCI.2020.PMID.32573490	1.05	0.86	1.28	0.63	0.89
TCGA.BRCA.1198_EN1_FDZ9_JCI.2020.PMID.32573490	1.11	0.89	1.38	0.35	0.75
TCGA.BRCA.1198_FGFR4_EGF_JCI.2020.PMID.32573490	1.02	0.84	1.24	0.85	0.97
TCGA.BRCA.1198_HISTONES_JCI.2020.PMID.32573490	1.27	1.03	1.56	0.02	0.20
TCGA.BRCA.1198_HOXC11_HOTAIR_SIX1_JCI.2020.PMID.32573490	1.10	0.90	1.33	0.36	0.76
TCGA.BRCA.1198_IL8_CCL_JCI.2020.PMID.32573490	0.96	0.78	1.17	0.67	0.90
TCGA.BRCA.1198_immune_CD19_JCI.2020.PMID.32573490	0.80	0.66	0.97	0.03	0.21
TCGA.BRCA.1198_immune_CD34_TIE1_JCI.2020.PMID.32573490	0.94	0.77	1.14	0.52	0.86
TCGA.BRCA.1198_immune_CD4_CD53_CD84_BTK_JCI.2020.PMID.32573490	0.79	0.65	0.96	0.02	0.19
TCGA.BRCA.1198_immune_CD8_GZMK_JCI.2020.PMID.32573490	0.78	0.64	0.95	0.01	0.19
TCGA.BRCA.1198_immune_CTLA4_CXCL_FOXP3_JCI.2020.PMID.32573490	0.89	0.73	1.08	0.24	0.63
TCGA.BRCA.1198_immune_FOS_JUN_IL6_JCI.2020.PMID.32573490	1.11	0.94	1.30	0.23	0.62
TCGA.BRCA.1198_immune_GIMAP_IL16_JCI.2020.PMID.32573490	0.85	0.70	1.03	0.10	0.42
TCGA.BRCA.1198_immune_HLA_A_F_JCI.2020.PMID.32573490	0.96	0.80	1.17	0.71	0.92
TCGA.BRCA.1198_immune_HLA_D_JCI.2020.PMID.32573490	0.76	0.63	0.91	0.00	0.19
TCGA.BRCA.1198_immune_INTERFERON_JCI.2020.PMID.32573490	1.15	0.95	1.39	0.15	0.50
TCGA.BRCA.1198_IMMUNE1_JCI.2020.PMID.32573490	0.76	0.63	0.93	0.01	0.19
TCGA.BRCA.1198_LUMINAL_JCI.2020.PMID.32573490	0.96	0.74	1.26	0.78	0.96
TCGA.BRCA.1198_MYBL2_APOBEC3B_JCI.2020.PMID.32573490	1.06	0.87	1.29	0.55	0.86
TCGA.BRCA.1198_NORMAL_JCI.2020.PMID.32573490	0.91	0.74	1.10	0.32	0.72
TCGA.BRCA.1198_NORMAL2_JCI.2020.PMID.32573490	0.86	0.71	1.05	0.14	0.49
TCGA.BRCA.1198_PDCHA_MANY_JCI.2020.PMID.32573490	1.05	0.86	1.29	0.62	0.89
TCGA.BRCA.1198_S100A7_8_9_JCI.2020.PMID.32573490	1.07	0.86	1.33	0.55	0.86
TCGA.BRCA.1198_TP63_JCI.2020.PMID.32573490	1.02	0.84	1.24	0.85	0.97
TCGA.BRCA.1198.IMMUNOGLOBULIN_JCI.2020.PMID.32573490	0.75	0.62	0.91	0.00	0.19
TCGA.CSF1.response_Immunity.2018.PMID.29628290	0.79	0.65	0.96	0.02	0.19
TCGA.IFN.score_Immunity.2018.PMID.29628290	1.17	0.97	1.42	0.10	0.42
TCGA.Liexpression.score_Immunity.2018.PMID.29628290	0.80	0.66	0.97	0.02	0.19
TCGA.Serum.response.up_Immunity.2018.PMID.29628290	0.99	0.82	1.18	0.88	0.97
TCGA.TFH_Immunity.2018.PMID.29628290	0.98	0.81	1.18	0.83	0.97
TCGA.Tgd_Immunity.2018.PMID.29628290	0.96	0.81	1.15	0.69	0.91
TCGA.TGFB.score_Immunity.2018.PMID.29628290	1.17	0.96	1.44	0.13	0.47
Tcm_Immunity.2013.PMID.24138885	0.90	0.74	1.10	0.29	0.69
Tem_Immunity.2013.PMID.24138885	0.89	0.73	1.07	0.21	0.59
TFH_Immunity.2013.PMID.24138885	0.98	0.81	1.18	0.83	0.97
Tgd_Immunity.2013.PMID.24138885	0.96	0.81	1.15	0.69	0.91
Th1_cells_Immunity.2013.PMID.24138885	0.89	0.72	1.10	0.27	0.68
Th17_cells_Immunity.2013.PMID.24138885	1.08	0.88	1.33	0.45	0.80

Th2_cells_Immunity.2013.PMID.24138885	1.04	0.86	1.26	0.69	0.91
TLS.9Gene.Signature_Nature.2020.PMID.31942071	0.85	0.71	1.03	0.10	0.43
TLS.CXCL13.SingleGene_Nature.2020.PMID.31942077	0.85	0.71	1.01	0.07	0.36
TLS.Hallmark.Gene.Signature_Nature.2020.PMID.31942071	0.78	0.65	0.94	0.01	0.19
TLS.Known.Markers_Nature.2020.PMID.31942071	0.74	0.61	0.90	0.00	0.19
TLS.Structure.12chemokine_FrontImmunol.2017.PMID.28713385	0.83	0.68	1.00	0.05	0.29
TLS.tumors.wTLS.and.CD8.vs.CD8alone_Nature.2020.PMID.31942071	0.79	0.64	0.96	0.02	0.19
TNBC.good.prognosis.TNBC.230genes_BCR.2011.PMID.21978456	1.08	0.89	1.30	0.43	0.79
TNBC.good.prognosis.TNBC.26genes_BCR.2011.PMID.21978456	0.90	0.74	1.09	0.28	0.68
TNBC.metastasis.free.survival_PLoS.One.2013.PMID.24349199	0.91	0.74	1.10	0.33	0.72
TNBC.poor.prognosis.TNBC.26genes_BCR.2011.PMID.21978456	1.06	0.88	1.29	0.52	0.85
Translation.Pathway_CancerImmunolRes.2018.PMID.30266715	0.94	0.77	1.13	0.50	0.84
Tumour.hypoxia.causes.DNA.hypermethylation_Nature.2016.PMID.27533040	1.30	1.06	1.59	0.01	0.19
Type.1.T.helper.cell_CellRep.2017.PMID.28052254	0.81	0.67	0.99	0.04	0.26
Type.17.T.helper.cell_CellRep.2017.PMID.28052254	0.93	0.76	1.14	0.49	0.83
Type.2.T.helper.cell_CellRep.2017.PMID.28052254	0.96	0.79	1.17	0.71	0.93
Up.Basal.High_Nat.Cell.Biol.2014.PMID.25173976	0.98	0.80	1.21	0.87	0.97
Up.Proliferation_Nat.Cell.Biol.2014.PMID.25173976	1.08	0.90	1.31	0.40	0.77
Upregulated.by.oncogenic.NRAS.basal_Cell.Rep.2016.PMID.26166574	0.92	0.76	1.12	0.41	0.77
Upregulated.upon.NRAS.repression.basal_Cell.Rep.2017.PMID.26166574	0.95	0.78	1.16	0.64	0.89
Vascular.Content_Clin.Exp.Metastasis.2014.PMID.23975155	1.21	0.97	1.51	0.09	0.39
VEGF.13genes_BMC.Med.2009.PMID.19291283	1.03	0.84	1.26	0.80	0.96
Wirapati.Proliferation_BCR.2008.PMID.18662380	1.14	0.94	1.37	0.18	0.56
Wound.Signature_CCR.2009.PMID.19887484	1.09	0.89	1.33	0.40	0.77
X11q13.Amplicon_BMC.Med.Genomics.2011.PMID.21214954	1.02	0.85	1.23	0.80	0.96
X12qMDM4.BMC.Med.Genomics.2011.PMID.21214954	1.07	0.89	1.29	0.45	0.80
X13q14.Amplicon_BMC.Med.Genomics.2011.PMID.21214954	0.92	0.77	1.10	0.36	0.76
X15q25.Amplicon_BMC.Med.Genomics.2011.PMID.21214954	0.86	0.71	1.05	0.13	0.48
X16.13.Amplicon_BMC.Med.Genomics.2011.PMID.21214954	0.98	0.80	1.20	0.84	0.97
X16q23.Amplicon_BMC.Med.Genomics.2011.PMID.21214954	0.85	0.71	1.02	0.09	0.40
X17PP13.Amplicon_BMC.Med.Genomics.2011.PMID.21214954	0.91	0.74	1.12	0.36	0.76
X17q25x.BMC.Med.Genomics.2011.PMID.21214954	0.96	0.79	1.17	0.68	0.91
X19p13.Amplicon_BMC.Med.Genomics.2011.PMID.21214954	0.99	0.82	1.20	0.95	0.98
X1p36.Amplicon_BMC.Med.Genomics.2011.PMID.21214954	0.90	0.74	1.10	0.32	0.72
X3p21.Amplicon_BMC.Med.Genomics.2011.PMID.21214954	0.92	0.76	1.12	0.43	0.78
X4p16.Amplicon_BMC.Med.Genomics.2011.PMID.21214954	0.91	0.74	1.11	0.35	0.74
X5Q_BCRT.2012.PMID.22048815	0.87	0.72	1.05	0.16	0.52
X8p.Amplicon_BMC.Med.Genomics.2011.PMID.21214954	1.10	0.91	1.32	0.33	0.72
X8p22.Amplicon_BMC.Med.Genomics.2011.PMID.21214954	0.94	0.77	1.14	0.51	0.85
XBP1.Signature_Nature.2014.PMID.24670641	0.92	0.76	1.12	0.41	0.78

CALGB 40601					
Signature	HR	95% CI		*P*	adjusted *P*
Activate.Endothelium_Clin.Exp.Metastasis.2014.PMID.23975155	1.47	1.05	2.05	0.02	0.21
Activated.B.cell_CellRep.2017.PMID.28052254	0.64	0.45	0.92	0.01	0.19
Activated.Blood.Neutrophil.Signature_Nat.Cell.Biol.2019.PMID.31263265	0.97	0.65	1.45	0.90	0.94
Activated.Cancer.Cell.Signature_Nat.Cell.Biol.2019.PMID.31263265	1.01	0.69	1.46	0.98	0.99
Activated.CD4.T.cell_CellRep.2017.PMID.28052254	1.02	0.74	1.39	0.92	0.95
Activated.CD8.T.cell_CellRep.2017.PMID.28052254	0.65	0.48	0.89	0.008	0.19
Activated.dendritic.cell_CellRep.2017.PMID.28052254	0.77	0.53	1.11	0.16	0.42
Activated.Lung.MSC.Signature_Nat.Cell.Biol.2019.PMID.31263265	1.44	0.89	2.34	0.14	0.39
Activated.Lung.Neutrophil.Signature_Nat.Cell.Biol.2019.PMID.31263265	1.25	0.88	1.76	0.21	0.47
aDC_Immunity.2013_PMID.24138885.PMID.24138885	0.75	0.52	1.08	0.12	0.37
ADM.S100A10.A110NDGR1.Cluster_BMC.Med.Genomics.2011.PMID.21214954	1.13	0.79	1.61	0.51	0.72
African.and.European.Ancestry.TCGA.Negative_JAMA.Oncol.2017.PMID.28472234	0.91	0.68	1.21	0.51	0.72
African.and.European.Ancestry.TCGA.Positive_JAMA.Oncol.2017.PMID.28472234	1.17	0.87	1.56	0.31	0.56
Age.associated.signature_Genome.Biol.2015.PMID.26343147	0.90	0.63	1.30	0.58	0.76
aMaSC_BCR.2010.PMID.20346151	0.94	0.70	1.26	0.66	0.82
aMaSC.HsEnriched_BCR.2015.PMID.25575446	1.19	0.88	1.60	0.25	0.51
aMaSC.HsEnriched.Refined1_BCR.2015.PMID.25575446	1.03	0.76	1.39	0.85	0.92
aMaSC.Lim09_BCR.2015.PMID.25575446	1.04	0.73	1.46	0.84	0.92
aMaSC.Prat_BCR.2015.PMID.25575446	1.18	0.87	1.60	0.30	0.55
aMaSC.Shehata_BCR.2015.PMID.25575446	0.78	0.55	1.12	0.18	0.44
aMaSC.Signature_Cell.Stem.Cell.2012.PMID.22305568	0.92	0.67	1.28	0.64	0.80
AMPH.EPIREGULIN.Cluster_BMC.Med.Genomics.2011.PMID.21214954	0.76	0.55	1.06	0.10	0.33
Amplification.50_Genome.Biol.2014.PMID.25164602	1.31	0.95	1.80	0.10	0.32
Amplification.50.better.than._Genome.Biol.2015.PMID.25164602	1.18	0.87	1.61	0.28	0.53
Apocrine.Features_J.Pathol.2017.PMID.27861902	1.13	0.76	1.68	0.56	0.76
aStr.HsEnriched_BCR.2015.PMID.25575446	0.85	0.64	1.14	0.28	0.53
aStr.HsEnriched.Refined1_BCR.2015.PMID.25575446	0.93	0.68	1.26	0.63	0.79
aStr.HsEnriched.Refined2_BCR.2015.PMID.25575446	0.88	0.67	1.16	0.35	0.60
aStr.Lim09_BCR.2015.PMID.25575446	0.88	0.66	1.17	0.38	0.62
aStr.Prat_BCR.2015.PMID.25575446	0.91	0.67	1.22	0.51	0.72
aStr.Shehata_BCR.2015.PMID.25575446	0.94	0.70	1.27	0.69	0.84
BASAL.Cluster_BMC.Med.Genomics.2011.PMID.21214954	0.87	0.63	1.19	0.37	0.61
Bcell.cluster_CCR.2014.PMID.24916698	0.65	0.45	0.94	0.02	0.21
Bcell.IL10.MINUS_Immunol.2014.PMID.25080484	1.14	0.80	1.63	0.48	0.69
Bcell.IL10.PLUS_Immunol.2014.PMID.25080484	0.87	0.60	1.26	0.46	0.69
Bcell.lineage.MCP_Nature.2020.PMID.31942077	0.65	0.45	0.94	0.02	0.21
Bcell.Plasma.52gene_Genome.Biol.2013.PMID.23618380	0.66	0.46	0.96	0.03	0.22
Bcell.Plasma.Metagene_Genome.Biol.2013.PMID.23618380	0.65	0.45	0.93	0.02	0.21
Bcell.Tcell.Cooperation_Cell.2019.PMID.31730857	0.67	0.46	0.96	0.03	0.22

Bcells_CancerImmunolRes.2018.PMID.30266715	0.64	0.44	0.93	0.02	0.20
Bcells_Immunity.2013.PMID.24138885	0.78	0.53	1.14	0.20	0.45
Bcells.Centroblast_JCO.2015.PMID.25800755	1.25	0.90	1.72	0.18	0.43
Bcells.Centrocyte_JCO.2015.PMID.25800755	1.33	0.88	1.99	0.17	0.42
Bcells.Memory_JCO.2015.PMID.25800755	0.75	0.51	1.08	0.12	0.37
Bcells.memory_Nat.Methods.2015.PMID.25822800	0.65	0.45	0.94	0.02	0.21
Bcells.Naive_JCO.2015.PMID.25800755	1.07	0.73	1.56	0.74	0.87
Bcells.naive_Nat.Methods.2015.PMID.25822800	0.72	0.50	1.04	0.08	0.31
Bcells.Plasmablast_JCO.2015.PMID.25800755	0.92	0.61	1.37	0.67	0.82
Blood.vessels_Immunity.2013.PMID.24138885	0.92	0.69	1.21	0.55	0.75
bMYB.Signature_Oncogene.2009.PMID.19043454	1.26	0.93	1.71	0.14	0.39
C3TAG.Responding_CCR.2013.PMID.23780888	0.74	0.54	1.00	0.05	0.25
C3TAG.Untreated_CCR.2013.PMID.23780888	1.34	0.97	1.86	0.08	0.31
CD103.Negative_Cancer.Cell.2014.PMID.25446897	0.81	0.60	1.10	0.17	0.42
CD103.Positive_Cancer.Cell.2014.PMID.25446897	0.68	0.47	0.99	0.05	0.25
CD103.Ratio_Cancer.Cell.2014.PMID.25446897	0.83	0.61	1.12	0.22	0.48
CD274_Single_Gene.Single	0.84	0.59	1.21	0.34	0.60
CD34.CD36.Cluster_BMC.Med.Genomics.PMID.21214954	0.80	0.61	1.06	0.12	0.37
CD44.downregulated.genes_Cancer.Cell.2007.PMID.17349583	0.57	0.38	0.88	0.01	0.19
CD44.upregulated.genes_Cancer.Cell.2007.PMID.17349583	1.97	1.28	3.03	0.002	0.19
CD56bright.natural.killer.cell_CellRep.2017.PMID.28052254	1.16	0.81	1.67	0.42	0.65
CD56dim.natural.killer.cell_CellRep.2017.PMID.28052254	1.30	0.94	1.78	0.11	0.34
CD68.cluster_CCR.2014.PMID.24916698	0.96	0.71	1.31	0.82	0.91
CD8.cluster_CCR.2014.PMID.24916698	0.65	0.46	0.92	0.02	0.19
CDKN2A_Single_Gene.Single	1.10	0.79	1.52	0.59	0.76
Central.memory.CD4.T.cell_CellRep.2017.PMID.28052254	0.85	0.59	1.22	0.37	0.61
Central.memory.CD8.T.cell_CellRep.2017.PMID.28052254	0.91	0.67	1.24	0.55	0.75
CES.Score_CCR.2017.PMID.27903675	0.49	0.30	0.79	0.003	0.19
Chromogramin_BMC.Med.Genomics.2011.PMID.21214954	0.86	0.62	1.20	0.38	0.62
CIN70_Nat.Genet.2006.PMID.16921376	1.38	1.01	1.88	0.04	0.25
Claudin.High_Genome.Biol.2007.PMID.17493263	0.72	0.51	1.02	0.07	0.29
Claudin.Low_Genome.Biol.2007.PMID.17493263	1.25	0.89	1.73	0.19	0.45
Claudin.Low.29_Cancer.Res.2009.PMID.19435916	1.20	0.89	1.61	0.23	0.50
cMYB.Signature_PLoS.One.2010.PMID.20949095	1.26	0.83	1.93	0.28	0.53
CORE.Bcell.signature.Garber_Cell.Mol.Gastroenterol.Hepatol.2017.PMID.28508029	0.72	0.49	1.05	0.08	0.32
CTLA4_Single_Gene.Single	0.86	0.62	1.19	0.36	0.61
Cytolytic.activity_Cell.2015.PMID.25594174	0.60	0.41	0.86	0.006	0.19
Cytotoxic.cells_Immunity.2013.PMID.24138885	0.72	0.52	1.01	0.06	0.27
Day7.Downregulated_Nat.Cell.Biol.2014.PMID.25173976	0.92	0.66	1.27	0.60	0.77
Day7.Upregulated_Nat.Cell.Biol.2014.PMID.25173976	1.33	0.95	1.84	0.09	0.32
DC_Immunity.2013.PMID.24138885	0.71	0.53	0.95	0.02	0.21
DCIS.HGF.down_BCR.2013.PMID.24025166	0.87	0.59	1.28	0.48	0.69
DCIS.HGF.up_BCR.2014.PMID.24025166	0.95	0.66	1.36	0.77	0.88
Delection.50_Genome.Biol.2016.PMID.25164602	1.08	0.80	1.48	0.61	0.77
Delection.50.better.than_Genome.Biol.2017.PMID.25164602	1.44	1.00	2.07	0.05	0.25
Dendritic.cells.activated_Nat.Methods.2015.PMID.25822800	0.85	0.59	1.21	0.36	0.61
Dendritic.cells.resting_Nat.Methods.2015.PMID.25822800	0.72	0.49	1.06	0.10	0.32
Down.Basal.High_Nat.Cell.Biol.2014.PMID.25173976	0.86	0.62	1.19	0.36	0.61
Down.CLOW.High_Nat.Cell.Biol.2014.PMID.25173976	0.96	0.73	1.28	0.79	0.90

Downregulated.upon.NRAS.repression.basal_Cell.Rep.2015.PMID.26166574	0.81	0.56	1.18	0.28	0.53
Ductal.Carcinoma.In.Situ_J.Pathol.2017.PMID.27861902	0.93	0.66	1.30	0.66	0.82
Duke.Module01.acidosis_PNASUSA.2010.PMID.20335537	0.75	0.55	1.02	0.06	0.29
Duke.Module02.akt_PNASUSA.2010.PMID.20335537	0.69	0.48	0.98	0.04	0.22
Duke.Module03.betacatenin_PNASUSA.2010.PMID.20335537	1.30	0.93	1.81	0.12	0.37
Duke.Module04.E2F1_PNASUSA.2010.PMID.20335537	1.32	0.89	1.95	0.16	0.42
Duke.Module05.EGFR_PNASUSA.2010.PMID.20335537	1.24	0.92	1.68	0.16	0.41
Duke.Module06.ER_PNASUSA.2010.PMID.20335537	0.90	0.60	1.36	0.62	0.79
Duke.Module07.glucosedepletion_PNASUSA.2010.PMID.20335537	1.50	0.99	2.29	0.06	0.27
Duke.Module08.HER2_PNASUSA.2010.PMID.20335537	1.32	0.89	1.95	0.16	0.42
Duke.Module09.hypoxia_PNASUSA.2010.PMID.20335537	1.58	1.13	2.22	0.008	0.19
Duke.Module10.IFNA_PNASUSA.2010.PMID.20335537	1.02	0.72	1.44	0.91	0.95
Duke.Module11.IFNG_PNASUSA.2010.PMID.20335537	1.05	0.74	1.48	0.80	0.90
Duke.Module12.lacticacidosis_PNASUSA.2010.PMID.20335537	1.35	0.93	1.97	0.11	0.35
Duke.Module13.myc_PNASUSA.2010.PMID.20335537	1.21	0.90	1.63	0.20	0.45
Duke.Module14.p53_PNASUSA.2010.PMID.20335537	0.84	0.56	1.25	0.39	0.63
Duke.Module15.p63_PNASUSA.2010.PMID.20335537	1.06	0.73	1.54	0.74	0.87
Duke.Module16.pi3k_PNASUSA.2010.PMID.20335537	1.58	1.11	2.24	0.01	0.19
Duke.Module17.PR_PNASUSA.2010.PMID.20335537	0.88	0.56	1.37	0.57	0.76
Duke.Module18.ras_PNASUSA.2010.PMID.20335537	1.84	1.19	2.84	0.006	0.19
Duke.Module19.src_PNASUSA.2010.PMID.20335537	0.84	0.56	1.27	0.42	0.65
Duke.Module20.STAT3_PNASUSA.2010.PMID.20335537	1.21	0.82	1.79	0.33	0.58
Duke.Module21.TGFB_PNASUSA.2010.PMID.20335537	1.46	1.05	2.02	0.02	0.21
Duke.Module22.TNFA_PNASUSA.2010.PMID.20335537	1.02	0.73	1.41	0.92	0.95
Durvalumab.signature_CCR.2018.PMID.29716923	0.91	0.64	1.29	0.59	0.76
Early.IRS.1_PLoS.One.2016.PMID.26991655	1.36	0.95	1.97	0.10	0.32
Early.IRS.2_PLoS.One.2016.PMID.26991655	1.17	0.82	1.68	0.38	0.63
Early.Relapse.ERPos.33genes_JAMA.2011.PMID.21558518	1.15	0.80	1.65	0.45	0.67
Early.Response.ERNeg.27genes_JAMA.2011.PMID.21558518	0.86	0.60	1.22	0.40	0.64
Effector.memeory.CD4.T.cell_CellRep.2017.PMID.28052254	0.96	0.64	1.42	0.82	0.91
Effector.memeory.CD8.T.cell_CellRep.2017.PMID.28052254	0.72	0.50	1.04	0.08	0.32
EGFR_Single_Gene.Single	0.95	0.65	1.40	0.80	0.90
EMT.down.Taube_PNAS.2010.PMID.20713713	1.22	0.89	1.68	0.21	0.47
EMT.down.Weingberg_PNAS.2010.PMID.20713713	1.21	0.88	1.66	0.24	0.50
EMT.up.Taube_PNAS.2010.PMID.20713713	1.04	0.74	1.46	0.83	0.91
EMT.up.Weinberg_PNAS.2010.PMID.20713713	1.07	0.76	1.51	0.69	0.83
Endothelial.cells.MCP_Nature.2020..PMID.31942077	0.99	0.75	1.31	0.94	0.96
Endothelial.Normal_Angiogenesis.2014.PMID.24257808	1.33	0.96	1.86	0.09	0.32
Endothelial.Tumor_Angiogenesis.2014.PMID.24257808	0.89	0.61	1.31	0.57	0.76
Eosinophil_CellRep.2017.PMID.28052254	0.80	0.58	1.10	0.17	0.42
Eosinophils_Immunity.2013.PMID.24138885	1.00	0.70	1.42	>0.99	>0.99
Eosinophils_Nat.Methods.2015.PMID.25822800	0.73	0.55	0.97	0.03	0.22
Epithelial.Tubule.Formation_J.Pathol.2017.PMID.27861902	0.91	0.59	1.39	0.66	0.82
ERBB2_Single_Gene.Single	1.17	0.86	1.59	0.32	0.58
ERBB3_Single_Gene.Single	1.19	0.86	1.65	0.30	0.55
ESR1_Single_Gene.Single	0.62	0.36	1.09	0.09	0.32
ESTIMATE.Immune_Nat.Commun.2013.PMID.24113773	0.72	0.50	1.04	0.08	0.32
ESTIMATE.Stromal_Nat.Commun.2013.PMID.24113773	0.79	0.59	1.07	0.13	0.38
Euclidean.Distance.CLOW_BCR.2010.PMID.20813035	1.10	0.79	1.54	0.57	0.76

EXTENDED.Bcell.signature.Garber_Cell.Mol.Gastroenterol.Hepatol.2017.PMID.28508029	0.84	0.59	1.19	0.32	0.58
FGFR4_Single_Gene.Single	1.49	1.05	2.12	0.02	0.21
FGFR4.Induced_JCI.2020.PMID.32573490	1.32	0.85	2.05	0.22	0.47
FGFR4.Repressed_JCI.2020.PMID.32573490	0.64	0.42	0.98	0.04	0.24
Fibrinogen.Cluster_BMC.Med.Genomics.2011.PMID.21214954	0.93	0.67	1.29	0.68	0.83
Fibroblast.Cluster_BMC.Med.Genomics.2011.PMID.21214954	1.06	0.74	1.54	0.74	0.87
Fibroblasts.MCP_Nature.2020.PMID.31942077	0.91	0.65	1.28	0.59	0.76
Fibromatosis_Lab.Invest.2008.PMID.18414401	1.06	0.73	1.53	0.76	0.88
fMaSC.Metab_CellRep.2018.PMID.30089273	0.96	0.71	1.32	0.82	0.91
fMaSC.Metab8_CellRep.2018.PMID.30089273	1.20	0.88	1.64	0.26	0.51
fMaSC.refined1_BCR.2015.PMID.25575446	0.80	0.57	1.12	0.19	0.44
fMasC.Signature_Cell.Stem.Cell.2012.PMID.22305568	0.56	0.39	0.79	0.001	0.19
fMaSC.Signature_CellRep.2018.PMID.30089273	1.44	0.98	2.13	0.06	0.29
FOS.JUN_Cluster_BMC.Med.Genomics.2011.PMID.21214954	0.97	0.65	1.47	0.90	0.94
FOXC1.Hair.Follicles.P30C.LO.vs.WT.Negative_Science.2016.PMID.26912704	1.14	0.83	1.57	0.43	0.66
FOXC1.Hair.Follicles.P30C.LO.vs.WT.Positive_Science.2016.PMID.26912704	0.72	0.53	1.00	0.05	0.25
fSTR.Signature_Cell.Stem.Cell.2012.PMID.22305568	0.81	0.61	1.07	0.13	0.37
Gamma.delta.T.cell_CellRep.2017.PMID.28052254	1.07	0.76	1.50	0.70	0.84
GATA3.induced.genes_JCO.2006.PMID.16505416	1.23	0.88	1.72	0.23	0.50
GATA3.induced.genes_Oncogene.2004.PMID.15361840	1.30	0.92	1.84	0.14	0.39
GDF11.TGFBR3_Nat.Cell.Biol.2014.PMID.24658685	0.95	0.65	1.39	0.78	0.89
Glycolysis_BMC.Med.2009.PMID.19291283	1.73	1.12	2.66	0.01	0.19
GO.DOWN.with.SOX10.OE_Cell.Rep.2015.PMID.26365194	1.14	0.79	1.66	0.48	0.70
GO.UP.with.SOX10.OE_Cell.Rep.2015.PMID.26365194	1.01	0.74	1.38	0.95	0.97
GSEA_BIOCARTA_ALK_PATHWAY.PMID.16199517	1.17	0.85	1.61	0.35	0.60
GSEA_BIOCARTA.AKT.PATHWAY.PMID.16199517	0.78	0.56	1.07	0.12	0.35
GSEA_BIOCARTA.BRCA.ATR.PATHWAY.ATRBRCA.PMID.16199517	1.12	0.80	1.58	0.50	0.71
GSEA_BIOCARTA.CASPASE.PATHWAY.PMID.16199517	0.71	0.48	1.06	0.09	0.32
GSEA_BIOCARTA.CTLA4.PATHWAY.PMID.16199517	0.62	0.43	0.88	0.007	0.19
GSEA_BIOCARTA.IGF1R.PATHWAY.PMID.16199517	0.76	0.56	1.05	0.10	0.32
GSEA_BIOCARTA.MTOR.PATHWAY.PMID.16199517	0.95	0.66	1.36	0.78	0.89
GSEA_BIOCARTA.PTEN.PATHWAY.PMID.16199517	0.75	0.54	1.03	0.08	0.32
GSEA_BIOCARTA.RAS.PATHWAY.PMID.16199517	0.89	0.66	1.21	0.46	0.68
GSEA_BIOCARTA.RB.PATHWAY.PMID.16199517	1.31	0.91	1.88	0.15	0.39
GSEA_BIOCARTA.VEGF.PATHWAY.PMID.16199517	1.31	0.88	1.96	0.19	0.44
GSEA_HALLMARK.MYC.TARGETS.V1.PMID.16199517	1.40	1.01	1.94	0.04	0.24
GSEA_HELLER.HDAC.TARGETS.DOWN.PMID.16199517	0.86	0.58	1.28	0.46	0.69
GSEA_NELSON.RESPONSE.TO.ANDROGEN.UP.PMID.16199517	1.12	0.75	1.67	0.59	0.76
GSEA_REACTOME.PD1.SIGNALING.PMID.16199517	0.61	0.41	0.92	0.02	0.20
GSEA_REACTOME.PI3K.CASCADE.PMID.16199517	1.18	0.82	1.69	0.38	0.62
GSEA_RETINOL.METABOLISM.KEGG.PMID.16199517	0.82	0.63	1.07	0.14	0.39
GSEA.GP1_Proliferation.DNA.repair..PUJANA.CHEK2.PCC.NETWORK.PMID.25109877	1.31	0.95	1.81	0.11	0.33
GSEA.GP1_Proliferation.DNA.repair.REACTOME.CELL.CYCLE.MITOTIC.PMID.25109877	1.35	0.99	1.85	0.06	0.27
GSEA.GP10_Fatty.acid.oxidation.CARBOXYLIC.ACID.METABOLIC.PROCESS.PMID.25109877	0.82	0.60	1.12	0.22	0.47
GSEA.GP11_Immune.IFN.PerouLab.PMID.25109877	1.07	0.76	1.50	0.70	0.84
GSEA.GP12_Hypoxia.glycolosis.SEMENZA.HIF1.TARGETS.PMID.25109877	1.50	1.04	2.17	0.03	0.22
GSEA.GP13_Neural.signailng.MODULE100.PMID.25109877	0.86	0.64	1.15	0.30	0.55
GSEA.GP13_Neural.signailng.NERVOUS.SYSTEM.DEVELOPMENT.PMID.25109877	0.90	0.63	1.29	0.58	0.76
GSEA.GP14_Plasma.membrane.cell.cell.signaling.MORF.CNTN1.PMID.25109877	0.83	0.58	1.18	0.30	0.55

GSEA.GP15_EGF.signaling.NAGASHIMA.EGF.SIGNALING.UP.PMID.25109877	1.22	0.76	1.98	0.41	0.65
GSEA.GP16_Protein.kinase.signaling.MAPKs.INTRACELLULAR.SIGNALING.CASCADE.PMID.25109877	1.00	0.69	1.43	>0.99	>0.99
GSEA.GP16_Protein.kinase.signaling.MAPKs.REGULATION.OF.KINASE.ACTIVITY.PMID.25109877	1.39	0.94	2.04	0.09	0.32
GSEA.GP17_Basal.signaling.SMID.BREAST.CANCER.BASAL.UP.PMID.25109877	1.17	0.81	1.69	0.41	0.65
GSEA.GP18_Vesicle.EPR.MEMBRANE.COAT.PMID.25109877	0.96	0.64	1.43	0.83	0.91
GSEA.GP19_1Q.amplicon.PerouLab.PMID.25109877	1.12	0.86	1.48	0.40	0.64
GSEA.GP2_Immune.Tcell.Bcell.KEGG.HEMATOPOIETIC.CELL.LINEAGE.PMID.25109877	0.60	0.41	0.88	0.008	0.19
GSEA.GP2_Immune.Tcell.Bcell.PerouLab.PMID.25109877	0.76	0.52	1.12	0.17	0.42
GSEA.GP20_TAL1.Leukemia.erythropoiesis.GNF2.TAL1.PMID.25109877	1.09	0.74	1.61	0.65	0.81
GSEA.GP21_Anti.apoptosis.DNA.stability.MORF.BCL2.PMID.25109877	0.81	0.55	1.21	0.30	0.55
GSEA.GP21_Anti.apoptosis.DNA.stability.MORF.MT4.PMID.25109877	0.92	0.61	1.38	0.69	0.83
GSEA.GP21_Anti.apoptosis.DNA.stability.MORF.STK17A.PMID.25109877	0.75	0.53	1.07	0.11	0.35
GSEA.GP22_16Q22.24.amplicon.PerouLab.PMID.25109877	1.09	0.76	1.57	0.65	0.81
GSEA.GP3_Tumo.suppressing.miRNA.targets.GTTTGTT.MIR.495.PMID.25109877	1.48	1.05	2.08	0.03	0.21
GSEA.GP3_Tumor.suppressing.miRNA.targets.DACOSTA.UV.RESPONSE.VIA.ERCC3.DN.PMID.25109877	1.25	0.91	1.71	0.17	0.42
GSEA.GP3_Tumor.suppressing.miRNA.targets.TGCTTTG.MIR.330.PMID.25109877	1.29	0.94	1.77	0.11	0.35
GSEA.GP4_MES.ECM.PerouLab.PMID.25109877	1.18	0.79	1.76	0.42	0.65
GSEA.GP5_MYC.targets.TERT.PerouLab.PMID.25109877	1.24	0.91	1.69	0.17	0.42
GSEA.GP6_Squamous.differentiation.development.RICKMAN.TUMOR.DIFFERENTIATED.WELL.VS.POORLY.DN.PMID.251098	1.23	0.86	1.76	0.25	0.51
GSEA.GP7_Estrogen.signaling.SMID.BREAST.CANCER.BASAL.DN.PMID.25109877	0.76	0.52	1.12	0.17	0.42
GSEA.GP8_FOXO.stemness.MORF.PTPRB.PMID.25109877	0.87	0.59	1.29	0.49	0.71
GSEA.GP8_FOXO.stemness.TTGTTT.VSFOXO4.01.PMID.25109877	1.05	0.77	1.44	0.75	0.87
GSEA.GP9_Cell.cell.adhesion.PerouLab.PMID.25109877	1.16	0.85	1.58	0.36	0.60
HCK_BCR.2008.PMID.19272155	0.81	0.58	1.12	0.21	0.46
HER1.Cluster1_BMC.Genomics.2007.PMID.17663798	1.23	0.85	1.78	0.27	0.52
HER1.Cluster2_BMC.Genomics.2007.PMID.17663798	1.19	0.87	1.63	0.27	0.52
HER1.Cluster3_BMC.Genomics.2007.PMID.17663798	1.47	1.04	2.08	0.03	0.22
HER2.Amplicon.PerouLab_BMC.Med.Genomic.2011.PMID.21214954	1.00	0.66	1.52	0.98	0.99
Histological.Grade_J.Pathol.2017.PMID.27861902	1.36	0.99	1.89	0.06	0.27
HouseKeeping_Genome.Biol.2004.PMID.15287981	1.04	0.70	1.54	0.85	0.92
iDC.Median_Immunity.2013.PMID.24138885	0.81	0.60	1.11	0.19	0.44
IFN.Cluster_BMC.Med.Genomics.2011.PMID.21214954	1.19	0.86	1.67	0.30	0.55
IgG_BCR.2008.PMID.19272155	0.65	0.45	0.93	0.02	0.20
IGG.Cluster_BMC.Med.Genomics.2011.PMID.21214954	0.63	0.44	0.92	0.02	0.19
Immature..B.cell_CellRep.2017.PMID.28052254	0.66	0.44	1.00	0.05	0.26
Immature.dendritic.cell_CellRep.2017.PMID.28052254	0.94	0.66	1.35	0.75	0.87
ImmLandscape_Macro.mono.CSF1.core.response_CCR.2009.PMID.29628290	0.76	0.53	1.09	0.14	0.39
ImmLandscape_Wound.Healing_Immunity.2018.PMID.29628290	1.25	0.94	1.67	0.13	0.37
ImmLandscape.IFN3_Plos.One.2014.PMID.24516633	1.20	0.87	1.67	0.26	0.51
ImmLandscape.IFNG5_Plos.One.2014.PMID.24516633	0.68	0.46	1.00	0.05	0.25
ImmLandscape.lymphocyte.Infil.T.B.PMID.18592372	0.62	0.43	0.89	0.009	0.19
Immune.Hot.CD8.vs.Cold_Nature.2020.PMID.31942071	0.69	0.47	1.01	0.06	0.27
Immune.Perez.14_JCO.2015.PMID.25605861	0.64	0.47	0.88	0.005	0.19
Immune.Perez.87_JCO.2015.PMID.25605861	0.61	0.43	0.88	0.008	0.19
Immune.Suppression_JCI.Insight.2016.PMID.27699256	0.88	0.60	1.27	0.48	0.70
ImmuneActive_Cell.2019.PMID.31730857	0.69	0.48	0.99	0.05	0.25
Immunosuppression.PMID.31942077	0.98	0.67	1.42	0.90	0.94
IMS.Score_CCR.2018.PMID.29921729	0.87	0.61	1.24	0.44	0.66
Induced.in.Bcells_PNAS.2013.PMID.23382184	0.91	0.65	1.26	0.56	0.76

Induced.in.DC_PNAS.2013.PMID.23382184	0.85	0.59	1.22	0.37	0.61
Induced.in.GN_PNAS.2013.PMID.23382184	0.83	0.60	1.14	0.25	0.51
Induced.in.HSC_PNAS.2013.PMID.23382184	1.67	1.08	2.58	0.02	0.21
Induced.in.MOs_PNAS.2013.PMID.23382184	0.95	0.70	1.27	0.71	0.85
Induced.in.NKcells_PNAS.2013.PMID.23382184	0.93	0.68	1.28	0.67	0.82
Induced.in.Tcells_PNAS.2013.PMID.23382184	0.81	0.54	1.20	0.29	0.54
Inflammatory.breast.cancer.491genes_CCR.2013.PMID.23396049	0.81	0.55	1.19	0.28	0.53
Inflammatory.breast.cancer.79genes_CCR.2013.PMID.23396049	1.36	0.92	2.03	0.12	0.37
Inflammatory.breast.cancer.expressed.noIBC_79genes_CCR.2013.PMID.23396049	1.26	0.87	1.82	0.21	0.47
Inflammatory.breast.cancer.expressed.noIBC.491genes_CCR.2013.PMID.23396049	1.20	0.83	1.74	0.33	0.58
Influenza.11genes.Metasignature_Immunity.2015.PMID.26682989	1.14	0.83	1.57	0.41	0.64
Interferon_BCR.2008.PMID.19272155	1.25	0.90	1.73	0.18	0.44
Interferon.Pathway_CancerImmunolRes.2018.PMID.30266715	1.14	0.82	1.60	0.43	0.66
JUND.KRT5_Nat.Cell.Biol.2014.PMID.24658685	0.93	0.66	1.33	0.71	0.84
Keller2012.CD10.Adam_BCR.2015.PMID.25575446	1.09	0.81	1.45	0.58	0.76
KRAS.amplicon_Genome.Biology.2007.PMID.17493263	1.51	1.06	2.13	0.02	0.21
Late.IRS.1_PLoS.One.2016.PMID.26991655	1.38	1.02	1.87	0.03	0.22
Late.IRS.2_PLoS.One.2016.PMID.26991655	0.87	0.60	1.26	0.46	0.69
LCK_BCR.2008.PMID.19272155	0.66	0.46	0.95	0.02	0.21
Lim2009.LumProg.Adam_BCR.2015.PMID.25575446	1.09	0.76	1.56	0.66	0.82
Lim2009.MaSC.Adam_BCR.2015.PMID.25575446	0.78	0.57	1.06	0.11	0.34
Lim2009.MatureLum.Adam_BCR.2015.PMID.25575446	1.05	0.71	1.56	0.80	0.90
Lim2009.Stroma.Adam_BCR.2015.PMID.25575446	0.85	0.65	1.13	0.27	0.52
Lim2010.LumProg.Adam_BCR.2015.PMID.25575446	0.93	0.64	1.35	0.71	0.85
Lim2010.MaSC.Adam_BCR.2015.PMID.25575446	0.90	0.67	1.21	0.48	0.69
Lim2010.MatureLum.Adam_BCR.2015.PMID.25575446	0.84	0.57	1.22	0.35	0.60
Lim2010.Stroma.Adam_BCR.2015.PMID.25575446	0.85	0.63	1.13	0.26	0.52
Lobular.Carcinoma.In.Situ_J.Pathol.2017.PMID.27861902	0.82	0.60	1.12	0.22	0.47
LOBULAR.TCGA.SIGNATURE.ImmuneCell.2015.PMID.26451490	0.76	0.55	1.04	0.09	0.32
LOBULAR.TCGA.SIGNATURE.Reactive_Cell.2015.PMID.26451490	0.83	0.62	1.13	0.24	0.50
LOBULAR.TCGA.SUBTYPE.Immune_Cell.2015.PMID.26451490	0.76	0.57	1.01	0.06	0.27
LOBULAR.TCGA.SUBTYPE.Proliferative_Cell.2015.PMID.26451490	0.79	0.61	1.02	0.07	0.31
LOBULAR.TCGA.SUBTYPE.Reactive_Cell.2015.PMID.26451490	0.79	0.59	1.07	0.13	0.38
LTS.score_JCI.2020.PMID.32573490	0.75	0.56	1.00	0.05	0.26
Luminal_Progenitor_Up_Nat.Med.2009.PMID.19648928	0.96	0.66	1.40	0.83	0.91
Luminal.cluster_BMC.Med.Genomics.2011.PMID.21214954	1.03	0.67	1.57	0.89	0.94
Luminal.Progenitor_BCR.2010.PMID.20346151	0.85	0.60	1.21	0.36	0.60
Luminal.Progenitor.Down_Nat.Med.2009.PMID.19648928	1.15	0.81	1.62	0.43	0.66
LumProg.HsEnriched_BCR.2015.PMID.25575446	0.94	0.66	1.36	0.75	0.87
LumProg.HsEnriched.Refined1_BCR.2015.PMID.25575446	0.86	0.62	1.21	0.40	0.64
LumProg.Lim09_BCR.2015.PMID.25575446	1.10	0.77	1.57	0.60	0.77
LumProg.Prat_BCR.2015.PMID.25575446	1.09	0.80	1.49	0.58	0.76
LumProg.Shehata_BCR.2015.PMID.25575446	1.15	0.83	1.58	0.40	0.64
Lums.HER2E.DOWN.metastatic.signature_JCI.2020.PMID.32573490	1.06	0.70	1.61	0.78	0.89
Lums.HER2E.UP.metastatic.signature_JCI.2020.PMID.32573490	1.03	0.73	1.45	0.88	0.94
Lung.WNT_Cancer.Res.2009.PMID.19549913	0.93	0.66	1.31	0.69	0.84
Lymph.vessels_Immunity.2013.PMID.24138885	1.06	0.72	1.57	0.75	0.87
Lymphovascular.Invasion_J.Pathol.2017.PMID.27861902	0.95	0.68	1.33	0.76	0.88
M.D.Metagene_Genome.Biol.2013.PMID.23618380	0.64	0.43	0.95	0.03	0.22

M2.Macrophage_Blood.2006.PMID.16556895	0.80	0.59	1.09	0.15	0.40
Macrophage_CellRep.2017.PMID.28052254	0.54	0.36	0.81	0.003	0.19
Macrophages_CancerImmunolRes.2018.PMID.30266715	0.79	0.56	1.11	0.17	0.42
Macrophages_Immunity.2013.PMID.24138885	0.96	0.70	1.31	0.79	0.89
Macrophages.M0_Nat.Methods.2015.PMID.25822800	0.90	0.64	1.25	0.53	0.73
Macrophages.M1_Nat.Methods.2015.PMID.25822800	0.91	0.63	1.32	0.63	0.80
Macrophages.M2_Nat.Methods.2015.PMID.25822800	0.71	0.50	1.00	0.05	0.25
MacTh1.cluster_CCR.2014.PMID.24916698	0.75	0.52	1.06	0.10	0.33
MammaPrint_Nature.2002.PMID.11823860	0.67	0.48	0.95	0.03	0.21
MAPK.pathway.activation_NPJ.Precis.Oncol.2018.PMID.29872725	1.29	0.95	1.75	0.11	0.33
MASC.Down_Nat.Med.2009.PMID.19648928	1.12	0.79	1.58	0.52	0.72
MASC.Up_Nat.Med.2009.PMID.19648928	0.94	0.70	1.28	0.71	0.85
Mast.cell_CellRep.2017.PMID.28052254	0.69	0.50	0.97	0.03	0.22
Mast.cells_Immunity.2013.PMID.24138885	0.84	0.63	1.14	0.27	0.52
Mast.cells.activated_Nat.Methods.2015.PMID.25822800	0.72	0.53	0.97	0.03	0.22
Mast.cells.resting_Nat.Methods.2015.PMID.25822800	0.74	0.56	0.98	0.03	0.22
Mature.luminal_BCR.2010.PMID.20346151	0.95	0.66	1.38	0.79	0.90
Mature.Luminal.Down_Nat.Med.2009.PMID.19648928	0.93	0.67	1.29	0.66	0.82
Mature.LuminaUp_Nat.Med.2009.PMID.19648928	1.02	0.69	1.49	0.93	0.96
MatureLum.HsEnriched_BCR.2015.PMID.25575446	0.95	0.66	1.37	0.80	0.90
MatureLum.HsEnriched.Refined1_BCR.2015.PMID.25575446	0.92	0.64	1.34	0.67	0.82
MatureLum.Lim09_BCR.2015.PMID.25575446	1.04	0.72	1.49	0.85	0.92
MatureLum.Prat_BCR.2015.PMID.25575446	1.14	0.82	1.59	0.44	0.66
MatureLum.Shehata_BCR.2015.PMID.25575446	1.19	0.86	1.66	0.29	0.54
MBasal.Cluster_BMC.Med.Genomics.2011.PMID.21214954	0.90	0.67	1.20	0.47	0.69
MCD3.CD8_BMC.Med.Genomics.2011.PMID.21214954	0.68	0.47	0.97	0.03	0.22
MCF7.E2.induced.genes_JCO.2006.PMID.16505416	1.20	0.88	1.65	0.26	0.51
MCF7.E2.repressed.genes_JCO.2006.PMID.16505416	1.13	0.78	1.62	0.52	0.72
MDSC_CellRep.2017.PMID.28052254	0.72	0.50	1.03	0.07	0.29
MDSC.Granulocytic_Leukoc.Biol.2012.PMID.21954284	1.23	0.80	1.90	0.34	0.59
MDSC.Neutrophi_Leukoc.Biol.2012.PMID.21954284	0.83	0.59	1.15	0.26	0.51
MDSC.tumor_J.Immunol.2012.PMID.23152559	1.03	0.70	1.50	0.90	0.94
MDSC.tumor.MO_J.Immunol.2012.PMID.23152559	1.03	0.70	1.52	0.89	0.94
MECM_BMC.Med.Genomics.2011.PMID.21214954	0.83	0.62	1.11	0.20	0.46
Memory.B.cell_CellRep.2017.PMID.28052254	1.27	0.88	1.84	0.20	0.46
MET.DOWN.RNAseq.Significant.Genes_JCI.2018.PMID.29480819	1.12	0.75	1.67	0.57	0.76
MET.DOWN.Significant.Genes.Low.Basal.1_JCI.2018.PMID.29480819	1.21	0.83	1.75	0.33	0.58
MET.DOWN.Significant.Genes.Low.Basal.2_JCI.2018.PMID.29480819	1.10	0.78	1.56	0.57	0.76
MET.UP.RNAseq.Significant.Genes_JCI.2018.PMID.29480819	1.03	0.74	1.44	0.84	0.92
MET.UP.Significant.Genes.HIGH.BASALS.Genes_JCI.2018.PMID.29480819	1.18	0.78	1.77	0.44	0.67
Metaplastic.Up_CanRes.2009.PMID.19435916	1.00	0.74	1.35	0.98	0.99
Metastasis.predictor.TNBC_BCR.2010.PMID.20946665	0.90	0.61	1.33	0.61	0.77
MFGFR2_BMC.Med.Genomics.2011.PMID.21214954	1.12	0.76	1.65	0.58	0.76
MHC.Forero.11_Cancer.Immunol.Res.2016.PMID.26980599	0.66	0.42	1.06	0.08	0.32
MHC.Forero.24_Cancer.Immunol.Res.2016.PMID.26980599	0.58	0.40	0.85	0.005	0.19
MHC.I_BCR.2008.PMID.19272155	0.87	0.58	1.31	0.51	0.72
MHC.II_BCR.2008.PMID.19272155	0.67	0.45	0.99	0.04	0.24
MHCI.coreGenes_Nat.Commun.2017.PMID29170503	0.99	0.67	1.45	0.95	0.97
MIR200c.Induced_ONCO.2015.PMID.25746005	0.94	0.68	1.30	0.70	0.84

MIR200c.Repressed_ONCO.2015.PMID.25746005	1.03	0.71	1.48	0.89	0.94
miRNA.138.signature_Cancer.Res.2014.PMID.25339353	1.23	0.87	1.74	0.24	0.50
MITO1_BMC.Med.Genomics.2011.PMID.21214954	0.99	0.71	1.38	0.94	0.96
MITO2_BMC.Med.Genomics.2011.PMID.21214954	1.12	0.74	1.71	0.59	0.76
Mitotic.Count_J.Pathol.2017.PMID.27861902	1.30	0.93	1.82	0.13	0.37
MK14.K17_BMC.Med.Genomics.2011.PMID.21214954	1.13	0.84	1.54	0.42	0.65
MKRAS.amplicon_BMC.Med.Genomics.2011.PMID.21214954	1.34	0.96	1.88	0.09	0.32
MM.BRCAwnt.1pFDR.UP_Genome.Biology.2007.PMID.17493263	1.60	1.13	2.26	0.009	0.19
MM.C3Tag.1pFDR.UP_Genome.Biology.2007.PMID.17493263	1.40	1.01	1.95	0.04	0.24
MM.C3Tag.2012_Genome.Biol.2013.PMID.24220145	1.35	0.99	1.83	0.06	0.27
MM.Class3_Genome.Biol.2013.PMID.24220145	1.03	0.72	1.47	0.89	0.94
MM.Class8_Genome.Biol.2013.PMID.24220145	1.03	0.66	1.60	0.91	0.94
MM.Claudinlow_Genome.Biol.2013.PMID.24220145	0.83	0.61	1.13	0.24	0.51
MM.DMBAwnt.1pFDR.UP.Genome.Biology.2007.PMID.17493263	1.00	0.73	1.36	0.98	0.99
MM.Erbb2.like_Genome.Biol.2013.PMID.24220145	0.87	0.64	1.17	0.36	0.61
MM.Myc.2012_Genome.Biol.2013.PMID.24220145	1.10	0.79	1.53	0.57	0.76
MM.Myoepithelioma.like_Genome.Biol.2013.PMID.24220145	0.88	0.65	1.20	0.43	0.66
MM.Neu.2012_Genome.Biol.2013.PMID.24220145	0.80	0.58	1.10	0.18	0.42
MM.NeuPyMT.1pFDR.UP_Genome.Biology.2007.PMID.17493263	1.14	0.72	1.79	0.58	0.76
MM.Normal.1pFDR.UP_Genome.Biology.2007.PMID.17493263	0.80	0.60	1.06	0.11	0.35
MM.Normal.like_Genome.Biol.2013.PMID.24220145	0.82	0.63	1.07	0.14	0.39
MM.p53null.1pFDR.UP_Genome.Biology.2007.PMID.17493263	1.00	0.71	1.40	0.98	0.99
MM.p53null.Basal_Genome.Biol.2013.PMID.24220145	1.11	0.79	1.57	0.54	0.74
MM.p53null.Luminal_Genome.Biol.2013.PMID.24220145	1.26	0.85	1.89	0.25	0.51
MM.Potluck_1pFDR_UP_Genome.Biology.2007_PMID.17493263.PMID.24220145	0.86	0.62	1.19	0.37	0.62
MM.PyMT.2012_Genome.Biol.2013.PMID.24220145	0.71	0.50	1.01	0.05	0.27
MM.Squamous.like_Genome.Biol.2013.PMID.24220145	0.98	0.71	1.37	0.92	0.95
MM.Stat1_Genome.Biol.2013.PMID.24220145	0.85	0.61	1.19	0.35	0.60
MM.WapINT3.1pFDR.UP_Genome.Biology.2007.PMID.17493263	1.38	0.95	1.99	0.09	0.32
MM.WapINT3.2012_Genome.Biol.2013.PMID.24220145	1.34	0.86	2.07	0.20	0.45
MM.WAPTag.1pFDR.UP_Genome.Biology.2007.PMID.17493263	1.32	0.97	1.78	0.07	0.31
MM.Wnt1.Early_Genome.Biol.2013.PMID.24220145	1.04	0.76	1.43	0.81	0.91
MM.Wnt1.Late_Genome.Biol.2013.PMID.24220145	1.05	0.74	1.47	0.79	0.90
Mmyosin_BMC.Med.Genomics.2011.PMID.21214954	1.03	0.72	1.47	0.89	0.94
MNADH_CYTochrome_BMC.Med.Genomics.2011.PMID.21214954	1.02	0.71	1.46	0.90	0.94
MNB1_BMC.Med.Genomics.2011.PMID.21214954	0.79	0.58	1.08	0.15	0.39
MNB2_BMC.Med.Genomics.2011.PMID.21214954	0.76	0.57	1.01	0.06	0.28
MNB3_BMC.Med.Genomics.2011.PMID.21214954	0.66	0.45	0.97	0.03	0.22
MNOtch4_BMC.Med.Genomics.2011.PMID.21214954	1.38	0.88	2.16	0.16	0.41
Monocyte_CellRep.2017.PMID.28052254	0.68	0.43	1.06	0.09	0.32
Monocyte..DC.25gene_Genome.Biol.2013.PMID.23618380	0.69	0.47	1.00	0.05	0.26
Monocytes_CancerImmunolRes.2018.PMID.30266715	0.75	0.53	1.05	0.09	0.32
Monocytes_Nat.Methods.2015.PMID.25822800	0.78	0.55	1.11	0.16	0.42
Monocytic.lineage.MCP_Nature.2020.PMID.31942075	0.87	0.63	1.21	0.41	0.65
MProliferation_BMC.Med.Genomics.2011.PMID.21214954	1.31	0.96	1.78	0.08	0.32
MProtocadherin_BMC.Med.Genomics.2011.PMID.21214954	0.86	0.63	1.17	0.33	0.59
MPYMT_NEU_Cluster_BMC.Med.Genomics.2011.PMID.21214954	0.78	0.56	1.09	0.15	0.39
MRibosomal_BMC.Med.Genomics.2011.PMID.21214954	0.88	0.59	1.30	0.52	0.72
MS.CD44.DOWN_PNAS.2009.PMID.19666588	1.21	0.88	1.67	0.25	0.51

MS.CD44.UP_PNAS.2009.PMID.19666588	0.94	0.69	1.27	0.66	0.82
MSquamous_BMC.Med.Genomics.2011.PMID.21214954	1.10	0.84	1.44	0.47	0.69
Murat.G07_JCO.2008.PMID.18565887	1.15	0.83	1.61	0.40	0.64
Murat.G18_JCO.2008.PMID.18565887	0.99	0.70	1.41	0.97	0.98
Murat.G24_JCO.2008.PMID.18565887	0.81	0.57	1.15	0.24	0.50
MVEGFC_BMC.Med.Genomics.2011.PMID.21214954	0.91	0.66	1.25	0.55	0.75
Myeloid.cell.chemotaxis.1gene_Nature.2020.PMID.31942077	0.88	0.62	1.27	0.50	0.71
Myeloid.dendritic.cells.MCP_Nature.2020.PMID.31942077	0.57	0.41	0.79	0.001	0.19
Natural.killer.cell_CellRep.2017.PMID.28052254	0.74	0.53	1.04	0.08	0.32
Natural.killer.T.cell_CellRep.2017.PMID.28052254	0.91	0.65	1.27	0.58	0.76
Necrosis_J.Pathol.2017.PMID.27861902	1.63	1.08	2.46	0.02	0.21
Neutrophil_CellRep.2017.PMID.28052254	0.75	0.54	1.04	0.09	0.32
Neutrophils_CancerImmunolRes.2018.PMID.30266715	0.84	0.59	1.20	0.34	0.59
Neutrophils_Immunity.2013.PMID.24138885	0.69	0.49	0.97	0.03	0.22
Neutrophils_Nat.Methods.2015.PMID.25822800	0.86	0.63	1.18	0.34	0.59
Neutrophils.MCP_Nature.2020.PMID.31942077	0.85	0.64	1.12	0.24	0.50
NK_Immunity.2013.PMID.24138885	0.83	0.58	1.20	0.32	0.58
NK.activated_Nat.Methods.2015.PMID.25822800	0.65	0.46	0.91	0.01	0.19
NK.CD56bright_Immunity.2013.PMID.24138885	0.86	0.58	1.27	0.44	0.67
NK.CD56dim_Immunity.2013.PMID.24138885	0.81	0.59	1.12	0.20	0.46
NK.resting_Nat.Methods.2015.PMID.25822800	0.62	0.44	0.89	0.01	0.19
NKcells_CancerImmunolRes.2018.PMID.30266715	0.57	0.40	0.83	0.003	0.19
NKcells.MCP_Nature.2020.PMID.31942077	0.69	0.51	0.93	0.02	0.19
No.Response.Immunotherapy.TLS.Melanoma_Nature.2020.PMID.31942075	0.91	0.65	1.26	0.56	0.76
Normal.mucosa_Immunity.2013.PMID.24138885	0.92	0.69	1.21	0.55	0.75
Nuclear.Pleomorphism_J.Pathol.2017.PMID.27861902	1.14	0.77	1.67	0.51	0.72
Oncotype_NEJM.2004.PMID.15591335	1.42	1.03	1.95	0.03	0.22
P53.ERPos.MDACC_CCR.2011.PMID.21248301	1.30	0.95	1.77	0.10	0.32
Parity.signature.251genes_BCR.2014.PMID.25005139	0.77	0.55	1.09	0.14	0.39
Parity.signature.40genes_BCR.2014.PMID.25005139	0.72	0.51	1.03	0.07	0.30
PARPi.Resistance_BCRT_2012.PMID.22875744	1.61	1.08	2.41	0.02	0.21
PARPi.Sensitivity_BCRT_2012.PMID.22875744	1.18	0.88	1.59	0.28	0.53
PARPi.Sensitivity.MDACC_NPJ.Syst.Biol.Appl.2017.PMID.28649435	1.05	0.71	1.56	0.81	0.91
PARPi.Sensitivity.Negative_Sci.Adv.2017.PMID.28439535	1.17	0.80	1.71	0.41	0.65
PARPi.Sensitivity.Positive_Sci.Adv.2017.PMID.28439535	1.14	0.81	1.61	0.45	0.67
Pcorr.Breast2Lung.LM2.Correlation_Nature.2005.PMID.16049480	0.97	0.67	1.42	0.89	0.94
Pcorr.Breast2Lung.Parental.Correlation_Nature.2005.PMID.16049480	0.98	0.68	1.43	0.93	0.96
Pcorr.dasatinib.resistant_Cancer.Res.2007.PMID.17332353	0.89	0.61	1.29	0.54	0.74
Pcorr.dasatinib.sensitive_Cancer.Res.2007.PMID.17332353	1.14	0.78	1.68	0.50	0.72
Pcorr.Hypoxia.High.Correlation_PLoS.Med.2006.PMID.16417408	1.33	0.87	2.02	0.19	0.44
Pcorr.Hypoxia.Low.Correlation_PLoS.Med.2006.PMID.16417408	0.76	0.49	1.17	0.21	0.47
Pcorr.IGS_Invasiveness_NJEM.2007.PMID.17229949	1.34	0.96	1.86	0.09	0.32
Pcorr.wound.response.activated_PNAS.2005.PMID.15701700	1.32	0.96	1.81	0.08	0.32
pCR.predictor.ERNeg.55genes_JAMA.2011.PMID.21558518	1.41	0.98	2.03	0.07	0.29
pCR.predictor.ERPos.39genes_JAMA.2011.PMID.21558518	1.03	0.73	1.45	0.88	0.94
PDCD1_Single_Gene.Single	0.75	0.53	1.06	0.10	0.33
Pfefferle2012.LumProg_BCR.2015.PMID.25575446	0.78	0.53	1.15	0.22	0.47
Pfefferle2012.MaSC_BCR.2015.PMID.25575446	1.05	0.78	1.40	0.75	0.87
Pfefferle2012.MatureLum_BCR.2015.PMID.25575446	1.20	0.82	1.75	0.34	0.59

Pfefferle2012.Stroma_BCR.2015.PMID.25575446	0.85	0.64	1.14	0.29	0.54
PGR_Single_Gene.Single	0.88	0.56	1.39	0.58	0.76
PI3Ki.Down_CancerCell.2017.PMID.28528867	1.46	1.03	2.06	0.04	0.22
PI3Ki.Up_CancerCell.2017.PMID.28528867	1.19	0.83	1.72	0.34	0.59
PIK3CA.Pathway_Ann.Oncol.2017.PMID.28177460	1.01	0.75	1.36	0.96	0.97
PIK3CAmt.signature_Cancer.Res.2012.PMID.22552288	1.45	1.01	2.08	0.05	0.25
Plasma.cells_Nat.Methods.2015.PMID.25822800	0.66	0.45	0.97	0.03	0.22
PlasmaCells_CancerImmunolRes.2018.PMID.30266715	0.86	0.60	1.22	0.40	0.64
Plasmacytoid.dendritic.cell_CellRep.2017.PMID.28052254	0.71	0.52	0.97	0.03	0.22
PR.Isoform.Ratio.Up.in.PRA.H_JNCI.2017.PMID.28376177	0.67	0.48	0.94	0.02	0.21
PR.Isoform.Ratio.Up.in.PRB.H_JNCI.2017.PMID.28376177	1.28	0.91	1.80	0.15	0.40
Proliferation.Cluster_BMC.Med.Genomics.2011.PMID.21214954	1.32	0.97	1.80	0.08	0.32
Proliferation.Metagene_Genome.Biol.2013.PMID.23618380	1.29	0.95	1.75	0.11	0.34
Proliferation.score.PAM50_JCO.2009.PMID.19204204	1.43	1.02	2.02	0.04	0.23
ProliferationPathway_CancerImmunolRes.2018.PMID.30266715	1.27	0.94	1.73	0.12	0.35
Prosigna.Proliferation.18_BMC.Med.Genomics.2015.PMID.26297356	1.26	0.93	1.70	0.14	0.38
Race.LuminalA.MRE.score_BCRT.2015.PMID.26109344	1.26	0.89	1.77	0.19	0.44
Radiation.induced.genes_Radoat.Res.2014.PMID.24527691	1.06	0.73	1.53	0.77	0.88
RB.LOH_BCR.2008.PMID.18782450	1.36	1.01	1.81	0.04	0.24
RB.LOSS_JCI.2007.PMID.17160137	1.28	0.94	1.74	0.12	0.35
Regulatory.T.cell_CellRep.2017.PMID.28052254	0.84	0.59	1.19	0.32	0.57
Replication.Stress.Down.set_Cell.Rep.2018.PMID.29768207	0.97	0.68	1.40	0.89	0.94
Replication.Stress.Model_Cell.Rep.2018_PMID.29768207.PMID.29768207	1.20	0.84	1.73	0.31	0.57
Replication.Stress.Neg_Cell.Rep.2018_PMID.29768207.PMID.29768207	0.90	0.63	1.29	0.57	0.76
Replication.Stress.Pos_Cell.Rep.2018_PMID.29768207.PMID.29768207	1.06	0.76	1.49	0.72	0.85
Replication.Stress.Up_Set_Cell.Rep.2018_PMID.29768207.PMID.29768207	1.10	0.78	1.56	0.57	0.76
Residual.disease.predictor.ERNeg.54genes_JAMA.2011.PMID.21558518	0.87	0.59	1.30	0.50	0.71
Residual.disease.predictor.ERPos.73genes_JAMA.2011.PMID.21558518	1.24	0.87	1.77	0.24	0.51
Response.Immunotherapy.MCP.TLS.Melanoma_Nature.2020.PMID.31942075	0.62	0.43	0.89	0.009	0.19
Response.Immunotherapy.signature_Science.2018.PMID.30309915	0.68	0.48	0.98	0.04	0.22
Response.Neo.Chemo_common_CCR.2014.PMID.25047707	1.38	1.01	1.88	0.05	0.25
Response.Neo.Chemo_ERNeg_CCR.2014.PMID.25047707	1.04	0.72	1.50	0.82	0.91
Response.Neo.Chemo_ERPos_CCR.2014.PMID.25047707	1.26	0.85	1.87	0.24	0.50
RHOA.pathway_Ann.Oncol.2017.PMID.28177460	1.15	0.80	1.66	0.45	0.68
Ribosomal.Cluster_BMC.Med.Genomics.2011.PMID.21214954	0.91	0.64	1.31	0.62	0.78
ROR.subtype.PAM50_JCO.2009.PMID.19204204	1.76	1.18	2.62	0.006	0.19
ROR.subtype.proliferation.PAM50_JCO.2009.PMID.19204204	1.51	1.06	2.15	0.02	0.21
RSS.Score_CCR.2018.PMID.29921729	1.26	0.88	1.81	0.21	0.46
S100A9.A8_BMC.Med.Genomics.2011.PMID.21214954	1.13	0.71	1.81	0.60	0.76
Scorr.EMAT1.Correlation_BCR.2020.PMID.32641077	0.76	0.53	1.11	0.15	0.40
Scorr.EMAT2.Correlation_BCR.2020.PMID.32641077	1.02	0.69	1.51	0.90	0.94
Scorr.EMAT3.Correlation_BCR.2020.PMID.32641077	1.13	0.79	1.60	0.51	0.72
Scorr.EMAT4.Correlation_BCR.2020.PMID.32641077	0.93	0.62	1.38	0.71	0.84
Scorr.IE.Correlation_JCO.2006.PMID.16505416	0.73	0.52	1.02	0.07	0.29
Scorr.IIE.Correlation_JCO.2006.PMID.16505416	1.34	0.95	1.89	0.10	0.32
Scorr.PAM50.Basal_JCO.2009.PMID.19204204	1.57	0.97	2.54	0.06	0.29
Scorr.PAM50.Her2_JCO.2009.PMID.19204204	1.73	1.17	2.56	0.006	0.19
Scorr.PAM50.LumA_JCO.2009.PMID.19204204	0.52	0.34	0.81	0.003	0.19
Scorr.PAM50.LumB_JCO.2009.PMID.19204204	1.25	0.91	1.73	0.17	0.42

Scorr.PAM50.Normal_JCO.2009.PMID.19204204	0.73	0.53	1.01	0.06	0.27
Scorr.S329.L_Br.J.Cancer.2008.PMID.18382427	1.04	0.70	1.53	0.85	0.92
Scorr.S329.R_Br.J.Cancer.2008.PMID.18382427	0.97	0.65	1.43	0.87	0.93
Secretoglobin_BMC.Med.Genomics.2011.PMID.21214954	1.05	0.70	1.56	0.83	0.91
Shehata2012.ALDHneg_BCR.2015.PMID.25575446	0.92	0.63	1.36	0.68	0.83
Shehata2012.ALDHpos_BCR.2015.PMID.25575446	0.80	0.57	1.11	0.18	0.44
Shehata2012.Basal_BCR.2015.PMID.25575446	0.88	0.65	1.19	0.40	0.64
Shehata2012.Erbb3neg_BCR.2015.PMID.25575446	0.84	0.58	1.21	0.35	0.60
Shehata2012.LumProg_BCR.2015.PMID.25575446	1.03	0.74	1.44	0.85	0.92
Shehata2012.NCL_BCR.2015.PMID.25575446	1.11	0.79	1.55	0.56	0.76
Shehata2012.Stroma_BCR.2015.PMID.25575446	0.92	0.69	1.23	0.58	0.76
Spike2012.aMaSC_BCR.2015.PMID.25575446	0.98	0.71	1.35	0.88	0.94
Spike2012.fMaSC_BCR.2015.PMID.25575446	0.67	0.47	0.94	0.02	0.21
Spike2012.fStr_BCR.2015.PMID.25575446	0.77	0.57	1.05	0.10	0.32
STAT1_BCR.2008.PMID.19272155	0.94	0.67	1.33	0.73	0.86
STAT3.Basal_PNAS.2014.PMID.25139989	0.76	0.51	1.12	0.17	0.42
STAT3.Basal.short_PNAS.2014.PMID.25139989	0.72	0.50	1.05	0.09	0.32
Stroma.FNA.MDACC.1_JCO.2010.PMID.20805453	0.70	0.47	1.04	0.08	0.31
Stroma.FNA.MDACC.2_JCO.2010.PMID.20805453	0.96	0.70	1.31	0.79	0.90
Stromal.Central.Fibrotic.Focus_J.Pathol.2017.PMID.27861902	0.90	0.54	1.48	0.67	0.82
Stromal.Down_Nat.Med.2009.PMID.19648928	1.15	0.86	1.53	0.35	0.60
Stromal.Inflammation_J.Pathol.2017.PMID.27861902	0.72	0.49	1.04	0.08	0.32
Stromal.Signature_Nat.Med.2008.PMID.18438415	0.65	0.47	0.89	0.008	0.19
Stromal.Up_Nat.Med.2009.PMID.19648928	0.86	0.65	1.14	0.30	0.55
SW480.cancer.cells_Immunity.2013.PMID.24138885	1.28	0.82	2.01	0.27	0.52
T.follicular.helper.cell_CellRep.2017.PMID.28052254	0.74	0.54	1.01	0.06	0.27
Tcell.activation_Nature.2020.PMID.31942077	0.95	0.70	1.28	0.73	0.86
Tcell.CD8.Effector.vs.naive.2_Science.2016.PMID27789795	1.30	0.95	1.76	0.10	0.32
Tcell.CD8.Exhausted.vs.antiPDL1.2_Science.2016.PMID27789795	1.42	1.01	1.98	0.04	0.24
Tcell.CD8.Exhausted.vs.naive.2_Science.2016.PMID27789795	1.27	0.93	1.71	0.13	0.37
Tcell.CD8.Memory.vs.naive.1_Science.2016.PMID27789795	0.87	0.62	1.23	0.43	0.66
Tcell.cluster_CCR.2014.PMID.24916698	0.64	0.45	0.91	0.01	0.19
Tcell.EXH.Anti.PDL1.vs.control.treated.exhausted.CD8.Tcell.Metagene.1.Science.2016.PMID.27789795	1.05	0.73	1.49	0.80	0.90
Tcell.EXH.Effector.CD8.T.cell.at.day.8.p.i.Armstrong.vs.Naive.CD8.Tcell.Metagene.1_Science.2016.PMID.27789795	0.85	0.59	1.21	0.37	0.61
Tcell.EXH.Exhausted.CD8.T.cell.vs.Naive.CD8.T.cell.Metagene.1_Science.2016.PMID.27789795	1.00	0.69	1.43	0.99	0.99
Tcell.EXH.Exhausted.CD8.T.cell.vs.Naive.CD8.T.cell.Metagene.3_Science.2016.PMID.27789795	0.89	0.64	1.26	0.52	0.72
Tcell.EXH.Memory.CD8.T.cell.a.vs.Naive.CD8.T.cell.Metagene.1_Science.2016.PMID.27789795	0.87	0.62	1.23	0.43	0.66
Tcell.EXH.Memory.CD8.T.cell.a.vs.Naive.CD8.T.cell.Metagene.2_Science.2016.PMID.27789795	0.90	0.65	1.25	0.52	0.72
Tcell.EXH.Memory.CD8.T.cell.a.vs.Naive.CD8.T.cell.Metagene.3.Science_2016.PMID.27789795	0.96	0.64	1.43	0.84	0.92
Tcell.NK.51gene_Genome.Biol.2013.PMID.23618380	0.61	0.42	0.89	0.01	0.19
Tcell.NK.Metagene_Genome.Biol.2013.PMID.23618380	0.62	0.42	0.90	0.01	0.19
Tcell.RM_Nat_Med.2018.PMID.29942092	0.71	0.47	1.05	0.09	0.32
Tcell.survival.2gene_Nature.2020.PMID.31942077	0.63	0.44	0.90	0.01	0.19
Tcells_CancerImmunolRes.2018.PMID.30266715	0.65	0.45	0.95	0.03	0.21
Tcells_Immunity.2013.PMID.24138885	0.63	0.44	0.90	0.01	0.19
Tcells_TFH_Nat.Methods.2015.PMID.25822800	0.66	0.46	0.95	0.03	0.21
Tcells.CD4.memory.activated_Nat.Methods.2015.PMID.25822800	0.77	0.55	1.09	0.15	0.39
Tcells.CD4.memory.resting_Nat.Methods.2015.PMID.25822800	0.62	0.43	0.88	0.008	0.19
Tcells.CD4.naive_Nat.Methods.2015.PMID.25822800	0.65	0.46	0.92	0.01	0.19

Tcells.CD8_Immunity.2013.PMID.24138885	1.03	0.69	1.52	0.90	0.94
Tcells.CD8_Nat.Methods.2015.PMID.25822800	0.64	0.45	0.91	0.01	0.19
Tcells.CD8.MCP_Nature.2020.PMID.31942075	0.61	0.43	0.87	0.006	0.19
Tcells.Cytotoxic.MCP_Nature.2020.PMID.31942075	0.69	0.47	1.01	0.06	0.27
Tcells.gammadelta_Nat.Methods.2015.PMID.25822800	0.62	0.44	0.88	0.007	0.19
Tcells.helper_Immunity.2013.PMID.24138885	1.17	0.81	1.71	0.40	0.64
Tcells.MCP_Nature.2020.PMID.31942077	0.69	0.49	0.98	0.04	0.22
Tcells.regulatory.2gene_Nature.2020.PMID.31942077	1.25	0.84	1.86	0.27	0.52
Tcells.Tregs_Nat.Methods.2015.PMID.25822800	0.63	0.44	0.90	0.01	0.19
TCGA.BRCA.1198_BASAL_JCI.2020.PMID.32573490	0.80	0.59	1.08	0.14	0.39
TCGA.BRCA.1198_Chromogranin_JCI.2020.PMID.32573490	0.93	0.64	1.35	0.72	0.85
TCGA.BRCA.1198_COLLAGEN11A_JCI.2020.PMID.32573490	1.27	0.86	1.86	0.23	0.49
TCGA.BRCA.1198_EN1_FDZ9_JCI.2020.PMID.32573490	1.17	0.78	1.76	0.45	0.68
TCGA.BRCA.1198_FGFR4_EGF_JCI.2020.PMID.32573490	1.21	0.82	1.79	0.34	0.59
TCGA.BRCA.1198_HISTONES_JCI.2020.PMID.32573490	1.17	0.83	1.64	0.37	0.61
TCGA.BRCA.1198_HOXC11_HOTAIR_SIX1_JCI.2020.PMID.32573490	1.22	0.86	1.74	0.27	0.52
TCGA.BRCA.1198_IL8_CCL_JCI.2020.PMID.32573490	1.23	0.86	1.76	0.25	0.51
TCGA.BRCA.1198_immune_CD19_JCI.2020.PMID.32573490	0.64	0.44	0.94	0.02	0.21
TCGA.BRCA.1198_immune_CD34_TIE1_JCI.2020.PMID.32573490	0.80	0.60	1.08	0.14	0.39
TCGA.BRCA.1198_immune_CD4_CD53_CD84_BTK_JCI.2020.PMID.32573490	0.74	0.52	1.05	0.09	0.32
TCGA.BRCA.1198_immune_CD8_GZMK_JCI.2020.PMID.32573490	0.64	0.45	0.92	0.01	0.19
TCGA.BRCA.1198_immune_CTLA4_CXCL_FOXP3_JCI.2020.PMID.32573490	0.96	0.70	1.32	0.82	0.91
TCGA.BRCA.1198_immune_FOS_JUN_IL6_JCI.2020.PMID.32573490	1.14	0.77	1.69	0.52	0.72
TCGA.BRCA.1198_immune_GIMAP_IL16_JCI.2020.PMID.32573490	0.74	0.53	1.04	0.08	0.32
TCGA.BRCA.1198_immune_HLA_A_F_JCI.2020.PMID.32573490	0.87	0.60	1.27	0.47	0.69
TCGA.BRCA.1198_immune_HLA_D_JCI.2020.PMID.32573490	0.66	0.45	0.97	0.04	0.22
TCGA.BRCA.1198_immune_INTERFERON_JCI.2020.PMID.32573490	1.17	0.84	1.63	0.35	0.60
TCGA.BRCA.1198_IMMUNE1_JCI.2020.PMID.32573490	0.65	0.45	0.93	0.02	0.21
TCGA.BRCA.1198_LUMINAL_JCI.2020.PMID.32573490	0.98	0.60	1.61	0.93	0.96
TCGA.BRCA.1198_MYBL2_APOBEC3B_JCI.2020.PMID.32573490	1.26	0.93	1.72	0.13	0.38
TCGA.BRCA.1198_NORMAL_JCI.2020.PMID.32573490	0.74	0.56	0.98	0.03	0.22
TCGA.BRCA.1198_NORMAL2_JCI.2020.PMID.32573490	0.84	0.64	1.11	0.22	0.47
TCGA.BRCA.1198_PDCHA_MANY_JCI.2020.PMID.32573490	0.96	0.62	1.51	0.87	0.94
TCGA.BRCA.1198_S100A7_8_9_JCI.2020.PMID.32573490	1.11	0.71	1.72	0.64	0.80
TCGA.BRCA.1198_TP63_JCI.2020.PMID.32573490	0.78	0.58	1.06	0.11	0.35
TCGA.BRCA.1198.IMMUNOGLOBULIN_JCI.2020.PMID.32573490	0.62	0.43	0.90	0.01	0.19
TCGA.CSF1.response_Immunity.2018.PMID.29628290	0.76	0.53	1.09	0.14	0.39
TCGA.IFN.score_Immunity.2018.PMID.29628290	1.20	0.87	1.67	0.26	0.51
TCGA.Liexpression.score_Immunity.2018.PMID.29628290	0.62	0.43	0.89	0.009	0.19
TCGA.Serum.response.up_Immunity.2018.PMID.29628290	1.20	0.89	1.61	0.23	0.48
TCGA.TFH_Immunity.2018.PMID.29628290	0.64	0.42	0.97	0.04	0.22
TCGA.Tgd_Immunity.2018.PMID.29628290	0.81	0.63	1.03	0.08	0.32
TCGA.TGFB.score_Immunity.2018.PMID.29628290	1.28	0.89	1.84	0.19	0.44
Tcm_Immunity.2013.PMID.24138885	0.97	0.68	1.37	0.85	0.92
Tem_Immunity.2013.PMID.24138885	0.91	0.66	1.24	0.54	0.74
TFH_Immunity.2013.PMID.24138885	0.64	0.42	0.97	0.04	0.22
Tgd_Immunity.2013.PMID.24138885	0.81	0.63	1.03	0.08	0.32
Th1_cells_Immunity.2013.PMID.24138885	0.92	0.62	1.37	0.68	0.83
Th17_cells_Immunity.2013.PMID.24138885	0.76	0.52	1.12	0.17	0.42

Th2_cells_Immunity.2013.PMID.24138885	1.27	0.91	1.79	0.16	0.42
TLS.9Gene.Signature_Nature.2020.PMID.31942071	0.70	0.51	0.97	0.03	0.22
TLS.CXCL13.SingleGene_Nature.2020.PMID.31942077	0.96	0.70	1.30	0.78	0.89
TLS.Hallmark.Gene.Signature_Nature.2020.PMID.31942071	0.64	0.45	0.92	0.02	0.19
TLS.Known.Markers_Nature.2020.PMID.31942071	0.61	0.42	0.88	0.009	0.19
TLS.Structure.12chemokine_FrontImmunol.2017.PMID.28713385	0.81	0.57	1.16	0.26	0.51
TLS.tumors.wTLS.and.CD8.vs.CD8alone_Nature.2020.PMID.31942071	0.59	0.41	0.87	0.007	0.19
TNBC.good.prognosis.TNBC.230genes_BCR.2011.PMID.21978456	1.45	1.03	2.03	0.03	0.22
TNBC.good.prognosis.TNBC.26genes_BCR.2011.PMID.21978456	0.83	0.58	1.19	0.31	0.57
TNBC.metastasis.free.survival_PLoS.One.2013.PMID.24349199	1.27	0.88	1.84	0.20	0.46
TNBC.poor.prognosis.TNBC.26genes_BCR.2011.PMID.21978456	1.55	1.08	2.22	0.02	0.20
Translation.Pathway_CancerImmunolRes.2018.PMID.30266715	0.88	0.61	1.27	0.50	0.71
Tumour.hypoxia.causes.DNA.hypermethylation_Nature.2016.PMID.27533040	2.14	1.44	3.19	<0.001	0.11
Type.1.T.helper.cell_CellRep.2017.PMID.28052254	0.68	0.44	1.03	0.07	0.29
Type.17.T.helper.cell_CellRep.2017.PMID.28052254	1.15	0.81	1.63	0.42	0.65
Type.2.T.helper.cell_CellRep.2017.PMID.28052254	1.50	0.96	2.33	0.07	0.31
Up.Basal.High_Nat.Cell.Biol.2014.PMID.25173976	0.88	0.62	1.25	0.48	0.70
Up.Proliferation_Nat.Cell.Biol.2014.PMID.25173976	1.36	0.99	1.86	0.06	0.27
Upregulated.by.oncogenic.NRAS.basal_Cell.Rep.2016.PMID.26166574	0.75	0.55	1.04	0.08	0.32
Upregulated.upon.NRAS.repression.basal_Cell.Rep.2017.PMID.26166574	0.91	0.64	1.29	0.58	0.76
Vascular.Content_Clin.Exp.Metastasis.2014.PMID.23975155	1.16	0.77	1.76	0.48	0.69
VEGF.13genes_BMC.Med.2009.PMID.19291283	1.20	0.83	1.73	0.34	0.59
Wirapati.Proliferation_BCR.2008.PMID.18662380	1.34	0.98	1.83	0.06	0.28
Wound.Signature_CCR.2009.PMID.19887484	0.83	0.59	1.15	0.26	0.52
X11q13.Amplicon_BMC.Med.Genomics.2011.PMID.21214954	1.05	0.71	1.56	0.80	0.90
X12qMDM4.BMC.Med.Genomics.2011.PMID.21214954	1.40	1.06	1.84	0.02	0.20
X13q14.Amplicon_BMC.Med.Genomics.2011.PMID.21214954	0.69	0.42	1.13	0.14	0.39
X15q25.Amplicon_BMC.Med.Genomics.2011.PMID.21214954	0.94	0.65	1.37	0.76	0.88
X16.13.Amplicon_BMC.Med.Genomics.2011.PMID.21214954	1.12	0.76	1.64	0.56	0.76
X16q23.Amplicon_BMC.Med.Genomics.2011.PMID.21214954	1.14	0.76	1.71	0.52	0.72
X17PP13.Amplicon_BMC.Med.Genomics.2011.PMID.21214954	0.94	0.69	1.29	0.72	0.85
X17q25x.BMC.Med.Genomics.2011.PMID.21214954	1.34	0.88	2.03	0.17	0.42
X19p13.Amplicon_BMC.Med.Genomics.2011.PMID.21214954	1.02	0.66	1.58	0.94	0.96
X1p36.Amplicon_BMC.Med.Genomics.2011.PMID.21214954	1.02	0.66	1.59	0.91	0.95
X3p21.Amplicon_BMC.Med.Genomics.2011.PMID.21214954	0.75	0.53	1.06	0.10	0.32
X4p16.Amplicon_BMC.Med.Genomics.2011.PMID.21214954	0.97	0.64	1.45	0.87	0.93
X5Q_BCRT.2012.PMID.22048815	1.01	0.65	1.55	0.97	0.98
X8p.Amplicon_BMC.Med.Genomics.2011.PMID.21214954	1.37	1.02	1.84	0.04	0.22
X8p22.Amplicon_BMC.Med.Genomics.2011.PMID.21214954	0.79	0.53	1.18	0.25	0.51
XBP1.Signature_Nature.2014.PMID.24670641	1.20	0.87	1.65	0.26	0.52

NeoALTTO					
Signature	HR	95% CI		*P*	adjusted *P*
Activate.Endothelium_Clin.Exp.Metastasis.2014.PMID.23975155	0.87	0.63	1.20	0.39	0.77
Activated.B.cell_CellRep.2017.PMID.28052254	0.95	0.71	1.26	0.70	0.88
Activated.Blood.Neutrophil.Signature_Nat.Cell.Biol.2019.PMID.31263265	0.81	0.64	1.04	0.10	0.55
Activated.Cancer.Cell.Signature_Nat.Cell.Biol.2019.PMID.31263265	1.11	0.82	1.50	0.50	0.82
Activated.CD4.T.cell_CellRep.2017.PMID.28052254	0.82	0.59	1.15	0.25	0.71
Activated.CD8.T.cell_CellRep.2017.PMID.28052254	0.90	0.67	1.19	0.44	0.80
Activated.dendritic.cell_CellRep.2017.PMID.28052254	0.85	0.62	1.17	0.32	0.74
Activated.Lung.MSC.Signature_Nat.Cell.Biol.2019.PMID.31263265	1.37	1.02	1.84	0.04	0.37
Activated.Lung.Neutrophil.Signature_Nat.Cell.Biol.2019.PMID.31263265	0.71	0.52	0.96	0.02	0.37
aDC_Immunity.2013_PMID.24138885.PMID.24138885	0.80	0.60	1.06	0.12	0.58
ADM.S100A10.A110NDGR1.Cluster_BMC.Med.Genomics.2011.PMID.21214954	0.91	0.65	1.26	0.57	0.85
African.and.European.Ancestry.TCGA.Negative_JAMA.Oncol.2017.PMID.28472234	1.35	0.97	1.90	0.08	0.50
African.and.European.Ancestry.TCGA.Positive_JAMA.Oncol.2017.PMID.28472234	1.02	0.77	1.35	0.89	0.96
Age.associated.signature_Genome.Biol.2015.PMID.26343147	1.29	0.98	1.68	0.07	0.47
aMaSC_BCR.2010.PMID.20346151	1.64	1.14	2.36	0.007	0.22
aMaSC.HsEnriched_BCR.2015.PMID.25575446	1.78	1.24	2.55	0.002	0.15
aMaSC.HsEnriched.Refined1_BCR.2015.PMID.25575446	1.54	1.09	2.17	0.01	0.34
aMaSC.Lim09_BCR.2015.PMID.25575446	1.43	1.04	1.97	0.03	0.37
aMaSC.Prat_BCR.2015.PMID.25575446	1.47	1.05	2.07	0.03	0.37
aMaSC.Shehata_BCR.2015.PMID.25575446	1.27	0.95	1.69	0.11	0.56
aMaSC.Signature_Cell.Stem.Cell.2012.PMID.22305568	1.43	1.02	2.00	0.04	0.37
AMPH.EPIREGULIN.Cluster_BMC.Med.Genomics.2011.PMID.21214954	1.43	1.06	1.93	0.02	0.37
Amplification.50_Genome.Biol.2014.PMID.25164602	0.99	0.77	1.28	0.95	0.97
Amplification.50.better.than._Genome.Biol.2015.PMID.25164602	0.86	0.65	1.14	0.30	0.74
Apocrine.Features_J.Pathol.2017.PMID.27861902	0.87	0.65	1.18	0.37	0.77
aStr.HsEnriched_BCR.2015.PMID.25575446	1.19	0.85	1.66	0.31	0.74
aStr.HsEnriched.Refined1_BCR.2015.PMID.25575446	1.33	0.95	1.85	0.10	0.55
aStr.HsEnriched.Refined2_BCR.2015.PMID.25575446	1.28	0.92	1.77	0.15	0.64
aStr.Lim09_BCR.2015.PMID.25575446	1.30	0.92	1.84	0.14	0.63
aStr.Prat_BCR.2015.PMID.25575446	1.19	0.85	1.66	0.31	0.74
aStr.Shehata_BCR.2015.PMID.25575446	1.15	0.82	1.62	0.43	0.79
BASAL.Cluster_BMC.Med.Genomics.2011.PMID.21214954	1.75	1.26	2.43	0.001	0.13
Bcell.cluster_CCR.2014.PMID.24916698	0.93	0.68	1.27	0.66	0.88
Bcell.IL10.MINUS_Immunol.2014.PMID.25080484	0.90	0.64	1.25	0.52	0.83
Bcell.IL10.PLUS_Immunol.2014.PMID.25080484	0.79	0.59	1.07	0.13	0.62
Bcell.lineage.MCP_Nature.2020.PMID.31942077	0.88	0.64	1.20	0.42	0.78
Bcell.Plasma.52gene_Genome.Biol.2013.PMID.23618380	0.79	0.59	1.07	0.13	0.61
Bcell.Plasma.Metagene_Genome.Biol.2013.PMID.23618380	0.94	0.69	1.26	0.67	0.88
Bcell.Tcell.Cooperation_Cell.2019.PMID.31730857	1.01	0.75	1.37	0.94	0.97

Bcells_CancerImmunolRes.2018.PMID.30266715	0.89	0.66	1.21	0.46	0.81
Bcells_Immunity.2013.PMID.24138885	0.98	0.73	1.30	0.87	0.95
Bcells.Centroblast_JCO.2015.PMID.25800755	0.86	0.63	1.16	0.31	0.74
Bcells.Centrocyte_JCO.2015.PMID.25800755	1.04	0.78	1.38	0.80	0.93
Bcells.Memory_JCO.2015.PMID.25800755	0.95	0.71	1.27	0.72	0.88
Bcells.memory_Nat.Methods.2015.PMID.25822800	0.92	0.69	1.23	0.58	0.85
Bcells.Naive_JCO.2015.PMID.25800755	1.00	0.74	1.35	>0.99	>0.99
Bcells.naive_Nat.Methods.2015.PMID.25822800	0.93	0.70	1.24	0.62	0.87
Bcells.Plasmablast_JCO.2015.PMID.25800755	0.86	0.63	1.16	0.32	0.74
Blood.vessels_Immunity.2013.PMID.24138885	1.34	0.96	1.86	0.08	0.50
bMYB.Signature_Oncogene.2009.PMID.19043454	0.82	0.61	1.11	0.20	0.66
C3TAG.Responding_CCR.2013.PMID.23780888	1.36	0.97	1.92	0.08	0.50
C3TAG.Untreated_CCR.2013.PMID.23780888	0.73	0.51	1.03	0.08	0.50
CD103.Negative_Cancer.Cell.2014.PMID.25446897	1.07	0.79	1.46	0.65	0.88
CD103.Positive_Cancer.Cell.2014.PMID.25446897	0.97	0.75	1.27	0.83	0.94
CD103.Ratio_Cancer.Cell.2014.PMID.25446897	1.03	0.76	1.40	0.84	0.94
CD274_Single_Gene.Single	0.75	0.53	1.04	0.08	0.50
CD34.CD36.Cluster_BMC.Med.Genomics.PMID.21214954	1.35	0.93	1.94	0.11	0.57
CD44.downregulated.genes_Cancer.Cell.2007.PMID.17349583	0.99	0.73	1.35	0.97	0.98
CD44.upregulated.genes_Cancer.Cell.2007.PMID.17349583	0.98	0.74	1.28	0.87	0.95
CD56bright.natural.killer.cell_CellRep.2017.PMID.28052254	0.86	0.65	1.13	0.29	0.74
CD56dim.natural.killer.cell_CellRep.2017.PMID.28052254	0.78	0.60	1.02	0.07	0.47
CD68.cluster_CCR.2014.PMID.24916698	0.96	0.72	1.26	0.75	0.90
CD8.cluster_CCR.2014.PMID.24916698	0.94	0.70	1.26	0.68	0.88
CDKN2A_Single_Gene.Single	1.18	0.90	1.54	0.23	0.69
Central.memory.CD4.T.cell_CellRep.2017.PMID.28052254	1.18	0.91	1.53	0.22	0.69
Central.memory.CD8.T.cell_CellRep.2017.PMID.28052254	0.81	0.60	1.11	0.19	0.65
CES.Score_CCR.2017.PMID.27903675	1.17	0.77	1.78	0.47	0.82
Chromogramin_BMC.Med.Genomics.2011.PMID.21214954	1.04	0.77	1.41	0.80	0.93
CIN70_Nat.Genet.2006.PMID.16921376	0.83	0.61	1.13	0.24	0.70
Claudin.High_Genome.Biol.2007.PMID.17493263	0.91	0.68	1.23	0.56	0.84
Claudin.Low_Genome.Biol.2007.PMID.17493263	1.09	0.79	1.52	0.60	0.85
Claudin.Low.29_Cancer.Res.2009.PMID.19435916	1.14	0.79	1.63	0.49	0.82
cMYB.Signature_PLoS.One.2010.PMID.20949095	0.99	0.76	1.28	0.92	0.97
CORE.Bcell.signature.Garber_Cell.Mol.Gastroenterol.Hepatol.2017.PMID.28508029	0.91	0.68	1.22	0.53	0.83
CTLA4_Single_Gene.Single	0.89	0.66	1.21	0.47	0.82
Cytolytic.activity_Cell.2015.PMID.25594174	0.91	0.70	1.18	0.48	0.82
Cytotoxic.cells_Immunity.2013.PMID.24138885	0.97	0.73	1.31	0.87	0.95
Day7.Downregulated_Nat.Cell.Biol.2014.PMID.25173976	1.41	0.98	2.02	0.07	0.47
Day7.Upregulated_Nat.Cell.Biol.2014.PMID.25173976	0.97	0.75	1.25	0.79	0.92
DC_Immunity.2013.PMID.24138885	0.94	0.65	1.35	0.74	0.90
DCIS.HGF.down_BCR.2013.PMID.24025166	1.61	1.15	2.24	0.005	0.18
DCIS.HGF.up_BCR.2014.PMID.24025166	1.34	0.99	1.82	0.06	0.46
Delection.50_Genome.Biol.2016.PMID.25164602	1.17	0.83	1.66	0.36	0.77
Delection.50.better.than_Genome.Biol.2017.PMID.25164602	1.25	0.93	1.66	0.13	0.62
Dendritic.cells.activated_Nat.Methods.2015.PMID.25822800	0.94	0.70	1.27	0.70	0.88
Dendritic.cells.resting_Nat.Methods.2015.PMID.25822800	0.90	0.65	1.24	0.51	0.83
Down.Basal.High_Nat.Cell.Biol.2014.PMID.25173976	1.46	1.05	2.03	0.02	0.37
Down.CLOW.High_Nat.Cell.Biol.2014.PMID.25173976	1.59	1.15	2.21	0.005	0.18

Downregulated.upon.NRAS.repression.basal_Cell.Rep.2015.PMID.26166574	0.97	0.74	1.27	0.82	0.93
Ductal.Carcinoma.In.Situ_J.Pathol.2017.PMID.27861902	0.99	0.72	1.36	0.94	0.97
Duke.Module01.acidosis_PNASUSA.2010.PMID.20335537	1.18	0.84	1.66	0.33	0.74
Duke.Module02.akt_PNASUSA.2010.PMID.20335537	0.87	0.69	1.10	0.24	0.70
Duke.Module03.betacatenin_PNASUSA.2010.PMID.20335537	0.85	0.65	1.12	0.25	0.71
Duke.Module04.E2F1_PNASUSA.2010.PMID.20335537	0.84	0.65	1.09	0.18	0.65
Duke.Module05.EGFR_PNASUSA.2010.PMID.20335537	1.01	0.76	1.35	0.92	0.97
Duke.Module06.ER_PNASUSA.2010.PMID.20335537	0.96	0.67	1.38	0.84	0.94
Duke.Module07.glucosedepletion_PNASUSA.2010.PMID.20335537	0.63	0.48	0.83	0.001	0.13
Duke.Module08.HER2_PNASUSA.2010.PMID.20335537	0.90	0.69	1.19	0.46	0.81
Duke.Module09.hypoxia_PNASUSA.2010.PMID.20335537	0.85	0.64	1.14	0.28	0.74
Duke.Module10.IFNA_PNASUSA.2010.PMID.20335537	1.06	0.80	1.41	0.67	0.88
Duke.Module11.IFNG_PNASUSA.2010.PMID.20335537	0.99	0.75	1.33	0.97	0.98
Duke.Module12.lacticacidosis_PNASUSA.2010.PMID.20335537	1.17	0.89	1.55	0.26	0.71
Duke.Module13.myc_PNASUSA.2010.PMID.20335537	0.98	0.75	1.29	0.91	0.97
Duke.Module14.p53_PNASUSA.2010.PMID.20335537	1.07	0.77	1.48	0.68	0.88
Duke.Module15.p63_PNASUSA.2010.PMID.20335537	0.86	0.65	1.14	0.30	0.74
Duke.Module16.pi3k_PNASUSA.2010.PMID.20335537	0.87	0.67	1.14	0.32	0.74
Duke.Module17.PR_PNASUSA.2010.PMID.20335537	1.12	0.77	1.62	0.56	0.85
Duke.Module18.ras_PNASUSA.2010.PMID.20335537	0.84	0.66	1.07	0.15	0.64
Duke.Module19.src_PNASUSA.2010.PMID.20335537	0.95	0.74	1.23	0.71	0.88
Duke.Module20.STAT3_PNASUSA.2010.PMID.20335537	0.88	0.61	1.26	0.48	0.82
Duke.Module21.TGFB_PNASUSA.2010.PMID.20335537	1.01	0.72	1.43	0.94	0.97
Duke.Module22.TNFA_PNASUSA.2010.PMID.20335537	0.90	0.65	1.24	0.52	0.83
Durvalumab.signature_CCR.2018.PMID.29716923	0.82	0.62	1.09	0.16	0.64
Early.IRS.1_PLoS.One.2016.PMID.26991655	0.92	0.67	1.26	0.59	0.85
Early.IRS.2_PLoS.One.2016.PMID.26991655	1.24	0.92	1.66	0.16	0.64
Early.Relapse.ERPos.33genes_JAMA.2011.PMID.21558518	0.81	0.60	1.08	0.15	0.64
Early.Response.ERNeg.27genes_JAMA.2011.PMID.21558518	1.08	0.82	1.43	0.59	0.85
Effector.memeory.CD4.T.cell_CellRep.2017.PMID.28052254	0.88	0.69	1.11	0.27	0.73
Effector.memeory.CD8.T.cell_CellRep.2017.PMID.28052254	0.92	0.69	1.24	0.59	0.85
EGFR_Single_Gene.Single	1.21	0.92	1.58	0.17	0.64
EMT.down.Taube_PNAS.2010.PMID.20713713	1.05	0.77	1.42	0.76	0.90
EMT.down.Weingberg_PNAS.2010.PMID.20713713	1.34	0.95	1.89	0.10	0.55
EMT.up.Taube_PNAS.2010.PMID.20713713	0.94	0.71	1.25	0.67	0.88
EMT.up.Weinberg_PNAS.2010.PMID.20713713	1.12	0.82	1.54	0.48	0.82
Endothelial.cells.MCP_Nature.2020..PMID.31942077	1.47	1.07	2.03	0.02	0.37
Endothelial.Normal_Angiogenesis.2014.PMID.24257808	0.85	0.61	1.19	0.35	0.76
Endothelial.Tumor_Angiogenesis.2014.PMID.24257808	1.19	0.89	1.58	0.23	0.70
Eosinophil_CellRep.2017.PMID.28052254	0.94	0.72	1.22	0.64	0.88
Eosinophils_Immunity.2013.PMID.24138885	0.98	0.73	1.32	0.90	0.97
Eosinophils_Nat.Methods.2015.PMID.25822800	0.85	0.63	1.16	0.31	0.74
Epithelial.Tubule.Formation_J.Pathol.2017.PMID.27861902	0.99	0.78	1.27	0.96	0.97
ERBB2_Single_Gene.Single	0.84	0.62	1.15	0.28	0.74
ERBB3_Single_Gene.Single	1.18	0.86	1.62	0.30	0.74
ESR1_Single_Gene.Single	1.09	0.70	1.69	0.71	0.88
ESTIMATE.Immune_Nat.Commun.2013.PMID.24113773	0.88	0.66	1.17	0.39	0.77
ESTIMATE.Stromal_Nat.Commun.2013.PMID.24113773	1.10	0.81	1.51	0.54	0.84
Euclidean.Distance.CLOW_BCR.2010.PMID.20813035	1.00	0.72	1.38	0.98	0.99

EXTENDED.Bcell.signature.Garber_Cell.Mol.Gastroenterol.Hepatol.2017.PMID.28508029	0.79	0.61	1.03	0.08	0.50
FGFR4_Single_Gene.Single	1.04	0.78	1.39	0.77	0.90
FGFR4.Induced_JCI.2020.PMID.32573490	0.89	0.67	1.17	0.40	0.77
FGFR4.Repressed_JCI.2020.PMID.32573490	1.02	0.77	1.37	0.87	0.96
Fibrinogen.Cluster_BMC.Med.Genomics.2011.PMID.21214954	1.30	0.97	1.74	0.08	0.50
Fibroblast.Cluster_BMC.Med.Genomics.2011.PMID.21214954	1.22	0.89	1.66	0.22	0.69
Fibroblasts.MCP_Nature.2020.PMID.31942077	1.34	0.99	1.82	0.06	0.46
Fibromatosis_Lab.Invest.2008.PMID.18414401	1.30	0.95	1.79	0.10	0.55
fMaSC.Metab_CellRep.2018.PMID.30089273	1.12	0.81	1.54	0.49	0.82
fMaSC.Metab8_CellRep.2018.PMID.30089273	0.73	0.52	1.01	0.06	0.46
fMaSC.refined1_BCR.2015.PMID.25575446	1.23	0.92	1.66	0.16	0.64
fMasC.Signature_Cell.Stem.Cell.2012.PMID.22305568	1.40	1.02	1.92	0.03	0.37
fMaSC.Signature_CellRep.2018.PMID.30089273	0.85	0.69	1.05	0.14	0.63
FOS.JUN_Cluster_BMC.Med.Genomics.2011.PMID.21214954	1.09	0.86	1.39	0.47	0.82
FOXC1.Hair.Follicles.P30C.LO.vs.WT.Negative_Science.2016.PMID.26912704	1.30	0.97	1.76	0.08	0.50
FOXC1.Hair.Follicles.P30C.LO.vs.WT.Positive_Science.2016.PMID.26912704	1.13	0.85	1.50	0.39	0.77
fSTR.Signature_Cell.Stem.Cell.2012.PMID.22305568	1.38	0.98	1.94	0.07	0.47
Gamma.delta.T.cell_CellRep.2017.PMID.28052254	0.99	0.73	1.33	0.94	0.97
GATA3.induced.genes_JCO.2006.PMID.16505416	1.06	0.79	1.41	0.71	0.88
GATA3.induced.genes_Oncogene.2004.PMID.15361840	0.90	0.71	1.14	0.37	0.77
GDF11.TGFBR3_Nat.Cell.Biol.2014.PMID.24658685	1.05	0.78	1.42	0.73	0.89
Glycolysis_BMC.Med.2009.PMID.19291283	0.96	0.70	1.32	0.81	0.93
GO.DOWN.with.SOX10.OE_Cell.Rep.2015.PMID.26365194	1.44	1.03	2.00	0.03	0.37
GO.UP.with.SOX10.OE_Cell.Rep.2015.PMID.26365194	1.24	0.92	1.67	0.16	0.64
GSEA_BIOCARTA_ALK_PATHWAY.PMID.16199517	1.06	0.80	1.41	0.67	0.88
GSEA_BIOCARTA.AKT.PATHWAY.PMID.16199517	1.03	0.78	1.37	0.82	0.93
GSEA_BIOCARTA.BRCA.ATR.PATHWAY.ATRBRCA.PMID.16199517	0.89	0.68	1.17	0.41	0.77
GSEA_BIOCARTA.CASPASE.PATHWAY.PMID.16199517	0.99	0.72	1.36	0.93	0.97
GSEA_BIOCARTA.CTLA4.PATHWAY.PMID.16199517	0.96	0.72	1.28	0.79	0.93
GSEA_BIOCARTA.IGF1R.PATHWAY.PMID.16199517	0.84	0.61	1.15	0.29	0.74
GSEA_BIOCARTA.MTOR.PATHWAY.PMID.16199517	0.89	0.67	1.17	0.40	0.77
GSEA_BIOCARTA.PTEN.PATHWAY.PMID.16199517	1.05	0.76	1.47	0.76	0.90
GSEA_BIOCARTA.RAS.PATHWAY.PMID.16199517	0.97	0.71	1.31	0.83	0.93
GSEA_BIOCARTA.RB.PATHWAY.PMID.16199517	0.87	0.67	1.13	0.29	0.74
GSEA_BIOCARTA.VEGF.PATHWAY.PMID.16199517	0.86	0.65	1.15	0.31	0.74
GSEA_HALLMARK.MYC.TARGETS.V1.PMID.16199517	1.23	0.90	1.68	0.18	0.65
GSEA_HELLER.HDAC.TARGETS.DOWN.PMID.16199517	0.81	0.59	1.12	0.20	0.67
GSEA_NELSON.RESPONSE.TO.ANDROGEN.UP.PMID.16199517	0.82	0.64	1.06	0.13	0.61
GSEA_REACTOME.PD1.SIGNALING.PMID.16199517	1.01	0.74	1.37	0.95	0.97
GSEA_REACTOME.PI3K.CASCADE.PMID.16199517	0.76	0.57	1.00	0.05	0.45
GSEA_RETINOL.METABOLISM.KEGG.PMID.16199517	1.14	0.79	1.65	0.49	0.82
GSEA.GP1_Proliferation.DNA.repair..PUJANA.CHEK2.PCC.NETWORK.PMID.25109877	0.85	0.65	1.10	0.22	0.69
GSEA.GP1_Proliferation.DNA.repair.REACTOME.CELL.CYCLE.MITOTIC.PMID.25109877	0.92	0.70	1.21	0.55	0.84
GSEA.GP10_Fatty.acid.oxidation.CARBOXYLIC.ACID.METABOLIC.PROCESS.PMID.25109877	0.86	0.65	1.15	0.31	0.74
GSEA.GP11_Immune.IFN.PerouLab.PMID.25109877	0.99	0.75	1.31	0.94	0.97
GSEA.GP12_Hypoxia.glycolosis.SEMENZA.HIF1.TARGETS.PMID.25109877	0.86	0.63	1.17	0.33	0.74
GSEA.GP13_Neural.signailng.MODULE100.PMID.25109877	1.45	1.02	2.06	0.04	0.37
GSEA.GP13_Neural.signailng.NERVOUS.SYSTEM.DEVELOPMENT.PMID.25109877	1.39	0.99	1.95	0.06	0.45
GSEA.GP14_Plasma.membrane.cell.cell.signaling.MORF.CNTN1.PMID.25109877	0.96	0.74	1.24	0.75	0.90

GSEA.GP15_EGF.signaling.NAGASHIMA.EGF.SIGNALING.UP.PMID.25109877	1.10	0.84	1.43	0.50	0.83
GSEA.GP16_Protein.kinase.signaling.MAPKs.INTRACELLULAR.SIGNALING.CASCADE.PMID.25109877	0.82	0.59	1.12	0.21	0.68
GSEA.GP16_Protein.kinase.signaling.MAPKs.REGULATION.OF.KINASE.ACTIVITY.PMID.25109877	1.06	0.79	1.44	0.68	0.88
GSEA.GP17_Basal.signaling.SMID.BREAST.CANCER.BASAL.UP.PMID.25109877	1.28	0.90	1.83	0.17	0.64
GSEA.GP18_Vesicle.EPR.MEMBRANE.COAT.PMID.25109877	0.79	0.57	1.09	0.15	0.64
GSEA.GP19_1Q.amplicon.PerouLab.PMID.25109877	1.00	0.76	1.33	0.98	0.99
GSEA.GP2_Immune.Tcell.Bcell.KEGG.HEMATOPOIETIC.CELL.LINEAGE.PMID.25109877	0.94	0.69	1.27	0.68	0.88
GSEA.GP2_Immune.Tcell.Bcell.PerouLab.PMID.25109877	0.90	0.68	1.19	0.45	0.80
GSEA.GP20_TAL1.Leukemia.erythropoiesis.GNF2.TAL1.PMID.25109877	0.95	0.72	1.24	0.70	0.88
GSEA.GP21_Anti.apoptosis.DNA.stability.MORF.BCL2.PMID.25109877	1.35	0.99	1.85	0.06	0.46
GSEA.GP21_Anti.apoptosis.DNA.stability.MORF.MT4.PMID.25109877	1.28	0.97	1.70	0.08	0.50
GSEA.GP21_Anti.apoptosis.DNA.stability.MORF.STK17A.PMID.25109877	1.48	1.06	2.05	0.02	0.37
GSEA.GP22_16Q22.24.amplicon.PerouLab.PMID.25109877	0.97	0.76	1.24	0.79	0.93
GSEA.GP3_Tumo.suppressing.miRNA.targets.GTTTGTT.MIR.495.PMID.25109877	0.80	0.60	1.06	0.12	0.61
GSEA.GP3_Tumor.suppressing.miRNA.targets.DACOSTA.UV.RESPONSE.VIA.ERCC3.DN.PMID.25109877	0.86	0.66	1.12	0.26	0.71
GSEA.GP3_Tumor.suppressing.miRNA.targets.TGCTTTG.MIR.330.PMID.25109877	0.91	0.69	1.19	0.49	0.82
GSEA.GP4_MES.ECM.PerouLab.PMID.25109877	1.14	0.85	1.54	0.38	0.77
GSEA.GP5_MYC.targets.TERT.PerouLab.PMID.25109877	0.89	0.68	1.15	0.36	0.77
GSEA.GP6_Squamous.differentiation.development.RICKMAN.TUMOR.DIFFERENTIATED.WELL.VS.POORLY.DN.PMID.251098	0.89	0.66	1.20	0.44	0.80
GSEA.GP7_Estrogen.signaling.SMID.BREAST.CANCER.BASAL.DN.PMID.25109877	1.18	0.80	1.74	0.40	0.77
GSEA.GP8_FOXO.stemness.MORF.PTPRB.PMID.25109877	1.21	0.91	1.61	0.19	0.65
GSEA.GP8_FOXO.stemness.TTGTTT.VSFOXO4.01.PMID.25109877	1.04	0.79	1.36	0.79	0.93
GSEA.GP9_Cell.cell.adhesion.PerouLab.PMID.25109877	1.14	0.82	1.59	0.45	0.80
HCK_BCR.2008.PMID.19272155	0.88	0.66	1.15	0.34	0.75
HER1.Cluster1_BMC.Genomics.2007.PMID.17663798	1.08	0.83	1.41	0.58	0.85
HER1.Cluster2_BMC.Genomics.2007.PMID.17663798	0.98	0.74	1.29	0.88	0.96
HER1.Cluster3_BMC.Genomics.2007.PMID.17663798	0.86	0.68	1.08	0.19	0.65
HER2.Amplicon.PerouLab_BMC.Med.Genomic.2011.PMID.21214954	0.86	0.64	1.16	0.33	0.74
Histological.Grade_J.Pathol.2017.PMID.27861902	0.85	0.64	1.13	0.25	0.71
HouseKeeping_Genome.Biol.2004.PMID.15287981	0.95	0.71	1.27	0.71	0.88
iDC.Median_Immunity.2013.PMID.24138885	1.17	0.86	1.61	0.32	0.74
IFN.Cluster_BMC.Med.Genomics.2011.PMID.21214954	1.12	0.84	1.49	0.44	0.80
IgG_BCR.2008.PMID.19272155	0.87	0.65	1.18	0.37	0.77
IGG.Cluster_BMC.Med.Genomics.2011.PMID.21214954	0.88	0.65	1.20	0.43	0.79
Immature..B.cell_CellRep.2017.PMID.28052254	0.89	0.66	1.20	0.45	0.80
Immature.dendritic.cell_CellRep.2017.PMID.28052254	1.14	0.84	1.54	0.40	0.77
ImmLandscape_Macro.mono.CSF1.core.response_CCR.2009.PMID.29628290	0.86	0.64	1.15	0.30	0.74
ImmLandscape_Wound.Healing_Immunity.2018.PMID.29628290	0.90	0.66	1.21	0.48	0.82
ImmLandscape.IFN3_Plos.One.2014.PMID.24516633	1.10	0.81	1.48	0.55	0.84
ImmLandscape.IFNG5_Plos.One.2014.PMID.24516633	0.86	0.64	1.16	0.33	0.74
ImmLandscape.lymphocyte.Infil.T.B.PMID.18592372	0.97	0.73	1.29	0.82	0.93
Immune.Hot.CD8.vs.Cold_Nature.2020.PMID.31942071	0.91	0.68	1.21	0.51	0.83
Immune.Perez.14_JCO.2015.PMID.25605861	1.13	0.84	1.52	0.43	0.79
Immune.Perez.87_JCO.2015.PMID.25605861	0.96	0.72	1.28	0.77	0.90
Immune.Suppression_JCI.Insight.2016.PMID.27699256	0.86	0.63	1.16	0.32	0.74
ImmuneActive_Cell.2019.PMID.31730857	0.90	0.67	1.22	0.49	0.82
Immunosuppression.PMID.31942077	1.11	0.85	1.45	0.45	0.80
IMS.Score_CCR.2018.PMID.29921729	1.08	0.79	1.48	0.61	0.86
Induced.in.Bcells_PNAS.2013.PMID.23382184	0.82	0.62	1.10	0.18	0.65

Induced.in.DC_PNAS.2013.PMID.23382184	0.76	0.56	1.03	0.08	0.50
Induced.in.GN_PNAS.2013.PMID.23382184	0.97	0.73	1.30	0.85	0.94
Induced.in.HSC_PNAS.2013.PMID.23382184	0.95	0.74	1.23	0.71	0.88
Induced.in.MOs_PNAS.2013.PMID.23382184	0.90	0.65	1.24	0.52	0.83
Induced.in.NKcells_PNAS.2013.PMID.23382184	1.08	0.80	1.47	0.61	0.86
Induced.in.Tcells_PNAS.2013.PMID.23382184	0.98	0.75	1.29	0.91	0.97
Inflammatory.breast.cancer.491genes_CCR.2013.PMID.23396049	0.87	0.65	1.17	0.35	0.76
Inflammatory.breast.cancer.79genes_CCR.2013.PMID.23396049	0.82	0.61	1.11	0.20	0.67
Inflammatory.breast.cancer.expressed.noIBC_79genes_CCR.2013.PMID.23396049	0.86	0.66	1.13	0.29	0.74
Inflammatory.breast.cancer.expressed.noIBC.491genes_CCR.2013.PMID.23396049	0.90	0.69	1.17	0.43	0.79
Influenza.11genes.Metasignature_Immunity.2015.PMID.26682989	1.04	0.77	1.39	0.81	0.93
Interferon_BCR.2008.PMID.19272155	1.13	0.84	1.52	0.41	0.77
Interferon.Pathway_CancerImmunolRes.2018.PMID.30266715	1.11	0.84	1.47	0.46	0.81
JUND.KRT5_Nat.Cell.Biol.2014.PMID.24658685	1.07	0.80	1.42	0.64	0.88
Keller2012.CD10.Adam_BCR.2015.PMID.25575446	1.46	1.04	2.04	0.03	0.37
KRAS.amplicon_Genome.Biology.2007.PMID.17493263	0.84	0.64	1.10	0.21	0.68
Late.IRS.1_PLoS.One.2016.PMID.26991655	0.90	0.67	1.22	0.50	0.82
Late.IRS.2_PLoS.One.2016.PMID.26991655	1.25	0.95	1.64	0.11	0.56
LCK_BCR.2008.PMID.19272155	0.92	0.69	1.23	0.58	0.85
Lim2009.LumProg.Adam_BCR.2015.PMID.25575446	1.18	0.86	1.62	0.32	0.74
Lim2009.MaSC.Adam_BCR.2015.PMID.25575446	1.46	1.05	2.03	0.02	0.37
Lim2009.MatureLum.Adam_BCR.2015.PMID.25575446	1.10	0.77	1.57	0.60	0.85
Lim2009.Stroma.Adam_BCR.2015.PMID.25575446	1.38	0.98	1.95	0.06	0.46
Lim2010.LumProg.Adam_BCR.2015.PMID.25575446	1.29	0.95	1.76	0.10	0.55
Lim2010.MaSC.Adam_BCR.2015.PMID.25575446	1.67	1.21	2.31	0.002	0.15
Lim2010.MatureLum.Adam_BCR.2015.PMID.25575446	1.17	0.89	1.54	0.25	0.71
Lim2010.Stroma.Adam_BCR.2015.PMID.25575446	1.28	0.91	1.79	0.16	0.64
Lobular.Carcinoma.In.Situ_J.Pathol.2017.PMID.27861902	1.54	1.15	2.07	0.004	0.18
LOBULAR.TCGA.SIGNATURE.ImmuneCell.2015.PMID.26451490	0.87	0.64	1.19	0.39	0.77
LOBULAR.TCGA.SIGNATURE.Reactive_Cell.2015.PMID.26451490	1.74	1.26	2.41	0.001	0.13
LOBULAR.TCGA.SUBTYPE.Immune_Cell.2015.PMID.26451490	1.25	0.86	1.81	0.25	0.71
LOBULAR.TCGA.SUBTYPE.Proliferative_Cell.2015.PMID.26451490	1.37	0.96	1.96	0.08	0.50
LOBULAR.TCGA.SUBTYPE.Reactive_Cell.2015.PMID.26451490	1.38	1.01	1.89	0.04	0.38
LTS.score_JCI.2020.PMID.32573490	1.52	1.11	2.08	0.009	0.26
Luminal_Progenitor_Up_Nat.Med.2009.PMID.19648928	1.23	0.88	1.71	0.22	0.69
Luminal.cluster_BMC.Med.Genomics.2011.PMID.21214954	0.85	0.62	1.17	0.32	0.74
Luminal.Progenitor_BCR.2010.PMID.20346151	1.23	0.87	1.74	0.24	0.70
Luminal.Progenitor.Down_Nat.Med.2009.PMID.19648928	1.26	0.89	1.78	0.19	0.65
LumProg.HsEnriched_BCR.2015.PMID.25575446	1.03	0.74	1.44	0.85	0.94
LumProg.HsEnriched.Refined1_BCR.2015.PMID.25575446	1.26	0.91	1.74	0.16	0.64
LumProg.Lim09_BCR.2015.PMID.25575446	1.05	0.78	1.42	0.74	0.90
LumProg.Prat_BCR.2015.PMID.25575446	1.09	0.79	1.49	0.61	0.86
LumProg.Shehata_BCR.2015.PMID.25575446	0.94	0.73	1.20	0.61	0.86
Lums.HER2E.DOWN.metastatic.signature_JCI.2020.PMID.32573490	1.48	1.08	2.03	0.01	0.34
Lums.HER2E.UP.metastatic.signature_JCI.2020.PMID.32573490	1.00	0.75	1.34	0.98	0.99
Lung.WNT_Cancer.Res.2009.PMID.19549913	0.99	0.74	1.33	0.95	0.97
Lymph.vessels_Immunity.2013.PMID.24138885	1.03	0.77	1.37	0.87	0.95
Lymphovascular.Invasion_J.Pathol.2017.PMID.27861902	1.06	0.79	1.42	0.71	0.88
M.D.Metagene_Genome.Biol.2013.PMID.23618380	0.93	0.70	1.22	0.59	0.85

M2.Macrophage_Blood.2006.PMID.16556895	0.95	0.70	1.30	0.77	0.90
Macrophage_CellRep.2017.PMID.28052254	1.22	0.88	1.69	0.23	0.70
Macrophages_CancerImmunolRes.2018.PMID.30266715	0.84	0.64	1.12	0.24	0.70
Macrophages_Immunity.2013.PMID.24138885	0.90	0.67	1.20	0.46	0.81
Macrophages.M0_Nat.Methods.2015.PMID.25822800	0.84	0.61	1.15	0.28	0.74
Macrophages.M1_Nat.Methods.2015.PMID.25822800	0.85	0.64	1.13	0.25	0.71
Macrophages.M2_Nat.Methods.2015.PMID.25822800	0.91	0.66	1.25	0.54	0.84
MacTh1.cluster_CCR.2014.PMID.24916698	0.86	0.64	1.16	0.32	0.74
MammaPrint_Nature.2002.PMID.11823860	1.20	0.83	1.73	0.32	0.74
MAPK.pathway.activation_NPJ.Precis.Oncol.2018.PMID.29872725	1.48	1.06	2.08	0.02	0.37
MASC.Down_Nat.Med.2009.PMID.19648928	0.95	0.73	1.22	0.68	0.88
MASC.Up_Nat.Med.2009.PMID.19648928	1.77	1.23	2.54	0.002	0.15
Mast.cell_CellRep.2017.PMID.28052254	1.11	0.81	1.51	0.52	0.83
Mast.cells_Immunity.2013.PMID.24138885	1.21	0.89	1.65	0.22	0.69
Mast.cells.activated_Nat.Methods.2015.PMID.25822800	1.22	0.91	1.63	0.19	0.65
Mast.cells.resting_Nat.Methods.2015.PMID.25822800	1.10	0.80	1.50	0.55	0.84
Mature.luminal_BCR.2010.PMID.20346151	1.10	0.78	1.55	0.58	0.85
Mature.Luminal.Down_Nat.Med.2009.PMID.19648928	1.31	0.92	1.86	0.13	0.62
Mature.LuminaUp_Nat.Med.2009.PMID.19648928	0.96	0.69	1.34	0.82	0.93
MatureLum.HsEnriched_BCR.2015.PMID.25575446	0.91	0.65	1.27	0.58	0.85
MatureLum.HsEnriched.Refined1_BCR.2015.PMID.25575446	0.93	0.67	1.28	0.64	0.88
MatureLum.Lim09_BCR.2015.PMID.25575446	0.87	0.64	1.19	0.38	0.77
MatureLum.Prat_BCR.2015.PMID.25575446	0.86	0.65	1.15	0.31	0.74
MatureLum.Shehata_BCR.2015.PMID.25575446	0.94	0.68	1.29	0.69	0.88
MBasal.Cluster_BMC.Med.Genomics.2011.PMID.21214954	1.97	1.33	2.92	0.001	0.13
MCD3.CD8_BMC.Med.Genomics.2011.PMID.21214954	0.93	0.70	1.24	0.63	0.87
MCF7.E2.induced.genes_JCO.2006.PMID.16505416	0.85	0.64	1.13	0.25	0.71
MCF7.E2.repressed.genes_JCO.2006.PMID.16505416	0.82	0.61	1.12	0.22	0.69
MDSC_CellRep.2017.PMID.28052254	0.91	0.67	1.22	0.53	0.83
MDSC.Granulocytic_Leukoc.Biol.2012.PMID.21954284	0.90	0.67	1.20	0.46	0.81
MDSC.Neutrophi_Leukoc.Biol.2012.PMID.21954284	0.86	0.62	1.18	0.35	0.75
MDSC.tumor_J.Immunol.2012.PMID.23152559	0.87	0.65	1.18	0.37	0.77
MDSC.tumor.MO_J.Immunol.2012.PMID.23152559	0.99	0.73	1.36	0.97	0.98
MECM_BMC.Med.Genomics.2011.PMID.21214954	1.45	1.02	2.07	0.04	0.37
Memory.B.cell_CellRep.2017.PMID.28052254	1.54	1.08	2.19	0.02	0.37
MET.DOWN.RNAseq.Significant.Genes_JCI.2018.PMID.29480819	0.93	0.75	1.16	0.54	0.84
MET.DOWN.Significant.Genes.Low.Basal.1_JCI.2018.PMID.29480819	1.10	0.81	1.50	0.52	0.83
MET.DOWN.Significant.Genes.Low.Basal.2_JCI.2018.PMID.29480819	0.90	0.70	1.15	0.41	0.77
MET.UP.RNAseq.Significant.Genes_JCI.2018.PMID.29480819	1.09	0.84	1.41	0.51	0.83
MET.UP.Significant.Genes.HIGH.BASALS.Genes_JCI.2018.PMID.29480819	0.84	0.63	1.12	0.23	0.70
Metaplastic.Up_CanRes.2009.PMID.19435916	1.23	0.89	1.71	0.20	0.67
Metastasis.predictor.TNBC_BCR.2010.PMID.20946665	0.75	0.58	0.98	0.03	0.37
MFGFR2_BMC.Med.Genomics.2011.PMID.21214954	1.16	0.86	1.57	0.34	0.75
MHC.Forero.11_Cancer.Immunol.Res.2016.PMID.26980599	0.80	0.58	1.09	0.15	0.64
MHC.Forero.24_Cancer.Immunol.Res.2016.PMID.26980599	0.95	0.74	1.23	0.70	0.88
MHC.I_BCR.2008.PMID.19272155	1.06	0.82	1.38	0.64	0.88
MHC.II_BCR.2008.PMID.19272155	0.80	0.61	1.05	0.11	0.56
MHCI.coreGenes_Nat.Commun.2017.PMID29170503	1.01	0.76	1.34	0.96	0.98
MIR200c.Induced_ONCO.2015.PMID.25746005	1.23	0.91	1.67	0.17	0.64

MIR200c.Repressed_ONCO.2015.PMID.25746005	1.28	0.91	1.80	0.15	0.64
miRNA.138.signature_Cancer.Res.2014.PMID.25339353	0.85	0.65	1.10	0.22	0.69
MITO1_BMC.Med.Genomics.2011.PMID.21214954	0.90	0.70	1.14	0.38	0.77
MITO2_BMC.Med.Genomics.2011.PMID.21214954	0.83	0.68	1.01	0.07	0.47
Mitotic.Count_J.Pathol.2017.PMID.27861902	1.08	0.81	1.45	0.58	0.85
MK14.K17_BMC.Med.Genomics.2011.PMID.21214954	1.36	0.99	1.87	0.05	0.45
MKRAS.amplicon_BMC.Med.Genomics.2011.PMID.21214954	0.84	0.64	1.09	0.18	0.65
MM.BRCAwnt.1pFDR.UP_Genome.Biology.2007.PMID.17493263	1.03	0.78	1.36	0.81	0.93
MM.C3Tag.1pFDR.UP_Genome.Biology.2007.PMID.17493263	1.11	0.82	1.51	0.50	0.82
MM.C3Tag.2012_Genome.Biol.2013.PMID.24220145	0.88	0.64	1.19	0.40	0.77
MM.Class3_Genome.Biol.2013.PMID.24220145	0.95	0.71	1.27	0.71	0.88
MM.Class8_Genome.Biol.2013.PMID.24220145	1.24	0.92	1.67	0.16	0.64
MM.Claudinlow_Genome.Biol.2013.PMID.24220145	1.16	0.85	1.59	0.36	0.77
MM.DMBAwnt.1pFDR.UP.Genome.Biology.2007.PMID.17493263	1.67	1.22	2.31	0.002	0.15
MM.Erbb2.like_Genome.Biol.2013.PMID.24220145	1.43	1.04	1.95	0.03	0.37
MM.Myc.2012_Genome.Biol.2013.PMID.24220145	1.01	0.79	1.31	0.91	0.97
MM.Myoepithelioma.like_Genome.Biol.2013.PMID.24220145	1.69	1.20	2.37	0.003	0.16
MM.Neu.2012_Genome.Biol.2013.PMID.24220145	1.48	1.08	2.02	0.01	0.34
MM.NeuPyMT.1pFDR.UP_Genome.Biology.2007.PMID.17493263	0.90	0.72	1.13	0.38	0.77
MM.Normal.1pFDR.UP_Genome.Biology.2007.PMID.17493263	1.31	0.89	1.95	0.17	0.64
MM.Normal.like_Genome.Biol.2013.PMID.24220145	1.49	1.04	2.13	0.03	0.37
MM.p53null.1pFDR.UP_Genome.Biology.2007.PMID.17493263	0.99	0.74	1.32	0.92	0.97
MM.p53null.Basal_Genome.Biol.2013.PMID.24220145	1.59	1.14	2.22	0.006	0.19
MM.p53null.Luminal_Genome.Biol.2013.PMID.24220145	0.95	0.73	1.22	0.66	0.88
MM.Potluck_1pFDR_UP_Genome.Biology.2007_PMID.17493263.PMID.24220145	1.03	0.76	1.41	0.83	0.94
MM.PyMT.2012_Genome.Biol.2013.PMID.24220145	1.10	0.85	1.42	0.48	0.82
MM.Squamous.like_Genome.Biol.2013.PMID.24220145	1.41	1.05	1.90	0.02	0.37
MM.Stat1_Genome.Biol.2013.PMID.24220145	1.31	0.94	1.82	0.11	0.56
MM.WapINT3.1pFDR.UP_Genome.Biology.2007.PMID.17493263	1.15	0.87	1.51	0.32	0.74
MM.WapINT3.2012_Genome.Biol.2013.PMID.24220145	1.03	0.79	1.33	0.84	0.94
MM.WAPTag.1pFDR.UP_Genome.Biology.2007.PMID.17493263	0.94	0.70	1.26	0.67	0.88
MM.Wnt1.Early_Genome.Biol.2013.PMID.24220145	1.56	1.11	2.19	0.01	0.28
MM.Wnt1.Late_Genome.Biol.2013.PMID.24220145	1.59	1.15	2.20	0.005	0.18
Mmyosin_BMC.Med.Genomics.2011.PMID.21214954	0.93	0.68	1.26	0.63	0.88
MNADH_CYTochrome_BMC.Med.Genomics.2011.PMID.21214954	0.83	0.67	1.04	0.10	0.55
MNB1_BMC.Med.Genomics.2011.PMID.21214954	0.85	0.65	1.12	0.25	0.71
MNB2_BMC.Med.Genomics.2011.PMID.21214954	1.41	0.92	2.16	0.11	0.56
MNB3_BMC.Med.Genomics.2011.PMID.21214954	1.04	0.84	1.28	0.75	0.90
MNOtch4_BMC.Med.Genomics.2011.PMID.21214954	1.34	1.01	1.78	0.04	0.37
Monocyte_CellRep.2017.PMID.28052254	0.80	0.60	1.05	0.11	0.56
Monocyte..DC.25gene_Genome.Biol.2013.PMID.23618380	0.87	0.66	1.15	0.32	0.74
Monocytes_CancerImmunolRes.2018.PMID.30266715	0.90	0.69	1.18	0.45	0.80
Monocytes_Nat.Methods.2015.PMID.25822800	0.88	0.66	1.17	0.37	0.77
Monocytic.lineage.MCP_Nature.2020.PMID.31942075	0.80	0.58	1.11	0.19	0.65
MProliferation_BMC.Med.Genomics.2011.PMID.21214954	0.81	0.60	1.09	0.16	0.64
MProtocadherin_BMC.Med.Genomics.2011.PMID.21214954	0.94	0.70	1.27	0.71	0.88
MPYMT_NEU_Cluster_BMC.Med.Genomics.2011.PMID.21214954	0.94	0.72	1.22	0.63	0.87
MRibosomal_BMC.Med.Genomics.2011.PMID.21214954	1.05	0.82	1.36	0.68	0.88
MS.CD44.DOWN_PNAS.2009.PMID.19666588	1.03	0.78	1.36	0.85	0.94

MS.CD44.UP_PNAS.2009.PMID.19666588	1.03	0.78	1.36	0.81	0.93
MSquamous_BMC.Med.Genomics.2011.PMID.21214954	1.11	0.81	1.51	0.52	0.83
Murat.G07_JCO.2008.PMID.18565887	1.31	0.94	1.82	0.11	0.57
Murat.G18_JCO.2008.PMID.18565887	1.38	1.02	1.87	0.04	0.37
Murat.G24_JCO.2008.PMID.18565887	0.82	0.61	1.10	0.19	0.65
MVEGFC_BMC.Med.Genomics.2011.PMID.21214954	1.17	0.85	1.62	0.34	0.75
Myeloid.cell.chemotaxis.1gene_Nature.2020.PMID.31942077	0.94	0.73	1.22	0.64	0.88
Myeloid.dendritic.cells.MCP_Nature.2020.PMID.31942077	0.88	0.67	1.15	0.33	0.74
Natural.killer.cell_CellRep.2017.PMID.28052254	1.13	0.85	1.50	0.40	0.77
Natural.killer.T.cell_CellRep.2017.PMID.28052254	0.97	0.73	1.30	0.85	0.94
Necrosis_J.Pathol.2017.PMID.27861902	0.89	0.69	1.15	0.36	0.77
Neutrophil_CellRep.2017.PMID.28052254	1.05	0.75	1.48	0.76	0.90
Neutrophils_CancerImmunolRes.2018.PMID.30266715	1.07	0.81	1.42	0.61	0.86
Neutrophils_Immunity.2013.PMID.24138885	1.11	0.80	1.52	0.54	0.84
Neutrophils_Nat.Methods.2015.PMID.25822800	0.84	0.61	1.14	0.26	0.71
Neutrophils.MCP_Nature.2020.PMID.31942077	1.07	0.76	1.53	0.69	0.88
NK_Immunity.2013.PMID.24138885	1.11	0.78	1.57	0.56	0.84
NK.activated_Nat.Methods.2015.PMID.25822800	0.95	0.71	1.27	0.71	0.88
NK.CD56bright_Immunity.2013.PMID.24138885	1.16	0.88	1.54	0.29	0.74
NK.CD56dim_Immunity.2013.PMID.24138885	0.81	0.60	1.10	0.17	0.64
NK.resting_Nat.Methods.2015.PMID.25822800	0.96	0.72	1.27	0.77	0.90
NKcells_CancerImmunolRes.2018.PMID.30266715	0.85	0.64	1.14	0.28	0.74
NKcells.MCP_Nature.2020.PMID.31942077	0.93	0.67	1.28	0.66	0.88
No.Response.Immunotherapy.TLS.Melanoma_Nature.2020.PMID.31942075	1.31	0.95	1.81	0.10	0.55
Normal.mucosa_Immunity.2013.PMID.24138885	1.35	0.99	1.85	0.06	0.45
Nuclear.Pleomorphism_J.Pathol.2017.PMID.27861902	0.97	0.74	1.28	0.85	0.94
Oncotype_NEJM.2004.PMID.15591335	0.78	0.53	1.14	0.20	0.67
P53.ERPos.MDACC_CCR.2011.PMID.21248301	0.85	0.62	1.16	0.30	0.74
Parity.signature.251genes_BCR.2014.PMID.25005139	0.84	0.63	1.12	0.24	0.70
Parity.signature.40genes_BCR.2014.PMID.25005139	0.86	0.64	1.14	0.29	0.74
PARPi.Resistance_BCRT_2012.PMID.22875744	0.68	0.50	0.93	0.02	0.37
PARPi.Sensitivity_BCRT_2012.PMID.22875744	0.82	0.59	1.13	0.22	0.69
PARPi.Sensitivity.MDACC_NPJ.Syst.Biol.Appl.2017.PMID.28649435	0.94	0.70	1.26	0.67	0.88
PARPi.Sensitivity.Negative_Sci.Adv.2017.PMID.28439535	1.32	0.99	1.77	0.06	0.46
PARPi.Sensitivity.Positive_Sci.Adv.2017.PMID.28439535	0.94	0.70	1.26	0.67	0.88
Pcorr.Breast2Lung.LM2.Correlation_Nature.2005.PMID.16049480	1.10	0.80	1.50	0.57	0.85
Pcorr.Breast2Lung.Parental.Correlation_Nature.2005.PMID.16049480	0.94	0.69	1.29	0.71	0.88
Pcorr.dasatinib.resistant_Cancer.Res.2007.PMID.17332353	1.13	0.81	1.57	0.47	0.81
Pcorr.dasatinib.sensitive_Cancer.Res.2007.PMID.17332353	0.90	0.65	1.26	0.54	0.84
Pcorr.Hypoxia.High.Correlation_PLoS.Med.2006.PMID.16417408	0.91	0.67	1.25	0.57	0.85
Pcorr.Hypoxia.Low.Correlation_PLoS.Med.2006.PMID.16417408	1.11	0.81	1.53	0.51	0.83
Pcorr.IGS_Invasiveness_NJEM.2007.PMID.17229949	0.71	0.51	0.97	0.03	0.37
Pcorr.wound.response.activated_PNAS.2005.PMID.15701700	0.90	0.67	1.21	0.49	0.82
pCR.predictor.ERNeg.55genes_JAMA.2011.PMID.21558518	1.02	0.79	1.33	0.85	0.94
pCR.predictor.ERPos.39genes_JAMA.2011.PMID.21558518	0.85	0.63	1.15	0.28	0.74
PDCD1_Single_Gene.Single	0.88	0.68	1.15	0.35	0.76
Pfefferle2012.LumProg_BCR.2015.PMID.25575446	1.07	0.75	1.53	0.72	0.88
Pfefferle2012.MaSC_BCR.2015.PMID.25575446	1.62	1.14	2.30	0.007	0.22
Pfefferle2012.MatureLum_BCR.2015.PMID.25575446	1.02	0.73	1.41	0.92	0.97

Pfefferle2012.Stroma_BCR.2015.PMID.25575446	1.38	0.96	1.98	0.08	0.50
PGR_Single_Gene.Single	1.20	0.84	1.70	0.32	0.74
PI3Ki.Down_CancerCell.2017.PMID.28528867	0.80	0.63	1.01	0.06	0.46
PI3Ki.Up_CancerCell.2017.PMID.28528867	0.93	0.71	1.22	0.59	0.85
PIK3CA.Pathway_Ann.Oncol.2017.PMID.28177460	0.99	0.73	1.34	0.94	0.97
PIK3CAmt.signature_Cancer.Res.2012.PMID.22552288	0.87	0.65	1.15	0.32	0.74
Plasma.cells_Nat.Methods.2015.PMID.25822800	0.95	0.71	1.28	0.75	0.90
PlasmaCells_CancerImmunolRes.2018.PMID.30266715	0.93	0.69	1.26	0.64	0.88
Plasmacytoid.dendritic.cell_CellRep.2017.PMID.28052254	1.09	0.81	1.47	0.58	0.85
PR.Isoform.Ratio.Up.in.PRA.H_JNCI.2017.PMID.28376177	1.41	1.03	1.94	0.03	0.37
PR.Isoform.Ratio.Up.in.PRB.H_JNCI.2017.PMID.28376177	1.13	0.82	1.55	0.46	0.81
Proliferation.Cluster_BMC.Med.Genomics.2011.PMID.21214954	0.83	0.61	1.13	0.23	0.70
Proliferation.Metagene_Genome.Biol.2013.PMID.23618380	0.80	0.59	1.09	0.16	0.64
Proliferation.score.PAM50_JCO.2009.PMID.19204204	0.72	0.52	0.99	0.04	0.39
ProliferationPathway_CancerImmunolRes.2018.PMID.30266715	0.80	0.59	1.09	0.15	0.64
Prosigna.Proliferation.18_BMC.Med.Genomics.2015.PMID.26297356	0.84	0.61	1.15	0.28	0.74
Race.LuminalA.MRE.score_BCRT.2015.PMID.26109344	0.88	0.64	1.21	0.44	0.80
Radiation.induced.genes_Radoat.Res.2014.PMID.24527691	1.17	0.86	1.60	0.31	0.74
RB.LOH_BCR.2008.PMID.18782450	0.88	0.66	1.19	0.40	0.77
RB.LOSS_JCI.2007.PMID.17160137	0.83	0.62	1.12	0.23	0.70
Regulatory.T.cell_CellRep.2017.PMID.28052254	0.94	0.71	1.24	0.65	0.88
Replication.Stress.Down.set_Cell.Rep.2018.PMID.29768207	1.02	0.78	1.32	0.90	0.97
Replication.Stress.Model_Cell.Rep.2018_PMID.29768207.PMID.29768207	0.98	0.71	1.35	0.90	0.97
Replication.Stress.Neg_Cell.Rep.2018_PMID.29768207.PMID.29768207	1.06	0.81	1.38	0.66	0.88
Replication.Stress.Pos_Cell.Rep.2018_PMID.29768207.PMID.29768207	1.41	1.04	1.91	0.03	0.37
Replication.Stress.Up_Set_Cell.Rep.2018_PMID.29768207.PMID.29768207	1.36	1.00	1.87	0.05	0.45
Residual.disease.predictor.ERNeg.54genes_JAMA.2011.PMID.21558518	1.22	0.92	1.62	0.17	0.64
Residual.disease.predictor.ERPos.73genes_JAMA.2011.PMID.21558518	0.84	0.65	1.09	0.19	0.65
Response.Immunotherapy.MCP.TLS.Melanoma_Nature.2020.PMID.31942075	0.87	0.63	1.19	0.38	0.77
Response.Immunotherapy.signature_Science.2018.PMID.30309915	0.93	0.70	1.24	0.63	0.87
Response.Neo.Chemo_common_CCR.2014.PMID.25047707	0.81	0.59	1.10	0.17	0.64
Response.Neo.Chemo_ERNeg_CCR.2014.PMID.25047707	0.98	0.75	1.30	0.91	0.97
Response.Neo.Chemo_ERPos_CCR.2014.PMID.25047707	0.95	0.71	1.27	0.73	0.89
RHOA.pathway_Ann.Oncol.2017.PMID.28177460	1.01	0.77	1.32	0.95	0.97
Ribosomal.Cluster_BMC.Med.Genomics.2011.PMID.21214954	1.06	0.82	1.37	0.64	0.88
ROR.subtype.PAM50_JCO.2009.PMID.19204204	0.67	0.46	0.98	0.04	0.37
ROR.subtype.proliferation.PAM50_JCO.2009.PMID.19204204	0.69	0.50	0.97	0.03	0.37
RSS.Score_CCR.2018.PMID.29921729	0.88	0.66	1.17	0.38	0.77
S100A9.A8_BMC.Med.Genomics.2011.PMID.21214954	0.90	0.64	1.26	0.54	0.84
Scorr.EMAT1.Correlation_BCR.2020.PMID.32641077	1.01	0.75	1.36	0.95	0.97
Scorr.EMAT2.Correlation_BCR.2020.PMID.32641077	1.43	1.04	1.95	0.03	0.37
Scorr.EMAT3.Correlation_BCR.2020.PMID.32641077	0.94	0.70	1.26	0.68	0.88
Scorr.EMAT4.Correlation_BCR.2020.PMID.32641077	1.18	0.80	1.74	0.39	0.77
Scorr.IE.Correlation_JCO.2006.PMID.16505416	1.43	1.02	2.01	0.04	0.37
Scorr.IIE.Correlation_JCO.2006.PMID.16505416	0.75	0.54	1.05	0.09	0.55
Scorr.PAM50.Basal_JCO.2009.PMID.19204204	1.08	0.73	1.62	0.69	0.88
Scorr.PAM50.Her2_JCO.2009.PMID.19204204	0.70	0.49	0.99	0.05	0.41
Scorr.PAM50.LumA_JCO.2009.PMID.19204204	1.36	0.92	2.03	0.13	0.61
Scorr.PAM50.LumB_JCO.2009.PMID.19204204	0.71	0.53	0.96	0.03	0.37

Scorr.PAM50.Normal_JCO.2009.PMID.19204204	1.45	1.06	1.98	0.02	0.37
Scorr.S329.L_Br.J.Cancer.2008.PMID.18382427	1.07	0.79	1.44	0.68	0.88
Scorr.S329.R_Br.J.Cancer.2008.PMID.18382427	0.90	0.66	1.23	0.52	0.83
Secretoglobin_BMC.Med.Genomics.2011.PMID.21214954	0.98	0.73	1.31	0.88	0.96
Shehata2012.ALDHneg_BCR.2015.PMID.25575446	1.24	0.92	1.65	0.15	0.64
Shehata2012.ALDHpos_BCR.2015.PMID.25575446	1.18	0.82	1.71	0.38	0.77
Shehata2012.Basal_BCR.2015.PMID.25575446	1.61	1.16	2.24	0.005	0.18
Shehata2012.Erbb3neg_BCR.2015.PMID.25575446	1.13	0.85	1.51	0.40	0.77
Shehata2012.LumProg_BCR.2015.PMID.25575446	1.09	0.79	1.49	0.60	0.85
Shehata2012.NCL_BCR.2015.PMID.25575446	1.04	0.74	1.45	0.83	0.94
Shehata2012.Stroma_BCR.2015.PMID.25575446	1.26	0.89	1.78	0.19	0.65
Spike2012.aMaSC_BCR.2015.PMID.25575446	1.27	0.93	1.72	0.13	0.61
Spike2012.fMaSC_BCR.2015.PMID.25575446	1.37	1.01	1.84	0.04	0.37
Spike2012.fStr_BCR.2015.PMID.25575446	1.17	0.86	1.58	0.33	0.74
STAT1_BCR.2008.PMID.19272155	0.89	0.66	1.21	0.47	0.81
STAT3.Basal_PNAS.2014.PMID.25139989	0.92	0.69	1.23	0.58	0.85
STAT3.Basal.short_PNAS.2014.PMID.25139989	0.94	0.71	1.25	0.67	0.88
Stroma.FNA.MDACC.1_JCO.2010.PMID.20805453	0.89	0.66	1.18	0.41	0.77
Stroma.FNA.MDACC.2_JCO.2010.PMID.20805453	1.26	0.91	1.75	0.16	0.64
Stromal.Central.Fibrotic.Focus_J.Pathol.2017.PMID.27861902	1.04	0.78	1.39	0.80	0.93
Stromal.Down_Nat.Med.2009.PMID.19648928	1.20	0.86	1.68	0.27	0.74
Stromal.Inflammation_J.Pathol.2017.PMID.27861902	0.84	0.62	1.14	0.25	0.71
Stromal.Signature_Nat.Med.2008.PMID.18438415	0.97	0.74	1.28	0.84	0.94
Stromal.Up_Nat.Med.2009.PMID.19648928	1.39	0.98	1.98	0.06	0.47
SW480.cancer.cells_Immunity.2013.PMID.24138885	1.63	1.18	2.24	0.003	0.16
T.follicular.helper.cell_CellRep.2017.PMID.28052254	1.14	0.83	1.57	0.43	0.79
Tcell.activation_Nature.2020.PMID.31942077	0.86	0.63	1.18	0.36	0.77
Tcell.CD8.Effector.vs.naive.2_Science.2016.PMID27789795	0.79	0.58	1.08	0.14	0.63
Tcell.CD8.Exhausted.vs.antiPDL1.2_Science.2016.PMID27789795	0.85	0.63	1.15	0.29	0.74
Tcell.CD8.Exhausted.vs.naive.2_Science.2016.PMID27789795	0.79	0.58	1.07	0.13	0.61
Tcell.CD8.Memory.vs.naive.1_Science.2016.PMID27789795	0.92	0.70	1.22	0.56	0.85
Tcell.cluster_CCR.2014.PMID.24916698	0.91	0.68	1.22	0.52	0.83
Tcell.EXH.Anti.PDL1.vs.control.treated.exhausted.CD8.Tcell.Metagene.1.Science.2016.PMID.27789795	1.02	0.77	1.37	0.88	0.96
Tcell.EXH.Effector.CD8.T.cell.at.day.8.p.i.Armstrong.vs.Naive.CD8.Tcell.Metagene.1_Science.2016.PMID.27789795	0.90	0.67	1.22	0.51	0.83
Tcell.EXH.Exhausted.CD8.T.cell.vs.Naive.CD8.T.cell.Metagene.1_Science.2016.PMID.27789795	0.92	0.68	1.24	0.59	0.85
Tcell.EXH.Exhausted.CD8.T.cell.vs.Naive.CD8.T.cell.Metagene.3_Science.2016.PMID.27789795	0.88	0.65	1.19	0.40	0.77
Tcell.EXH.Memory.CD8.T.cell.a.vs.Naive.CD8.T.cell.Metagene.1_Science.2016.PMID.27789795	0.92	0.70	1.22	0.56	0.85
Tcell.EXH.Memory.CD8.T.cell.a.vs.Naive.CD8.T.cell.Metagene.2_Science.2016.PMID.27789795	0.87	0.63	1.21	0.42	0.78
Tcell.EXH.Memory.CD8.T.cell.a.vs.Naive.CD8.T.cell.Metagene.3.Science_2016.PMID.27789795	0.80	0.58	1.10	0.17	0.64
Tcell.NK.51gene_Genome.Biol.2013.PMID.23618380	0.91	0.68	1.22	0.54	0.84
Tcell.NK.Metagene_Genome.Biol.2013.PMID.23618380	0.90	0.67	1.21	0.47	0.82
Tcell.RM_Nat_Med.2018.PMID.29942092	0.90	0.67	1.21	0.49	0.82
Tcell.survival.2gene_Nature.2020.PMID.31942077	1.01	0.78	1.30	0.95	0.97
Tcells_CancerImmunolRes.2018.PMID.30266715	0.92	0.69	1.23	0.57	0.85
Tcells_Immunity.2013.PMID.24138885	0.98	0.73	1.31	0.90	0.96
Tcells_TFH_Nat.Methods.2015.PMID.25822800	1.00	0.75	1.33	0.99	>0.99
Tcells.CD4.memory.activated_Nat.Methods.2015.PMID.25822800	0.85	0.63	1.14	0.27	0.74
Tcells.CD4.memory.resting_Nat.Methods.2015.PMID.25822800	0.98	0.74	1.30	0.88	0.96
Tcells.CD4.naive_Nat.Methods.2015.PMID.25822800	1.00	0.75	1.32	0.99	0.99

Tcells.CD8_Immunity.2013.PMID.24138885	0.86	0.67	1.11	0.24	0.70
Tcells.CD8_Nat.Methods.2015.PMID.25822800	0.96	0.72	1.29	0.81	0.93
Tcells.CD8.MCP_Nature.2020.PMID.31942075	1.11	0.83	1.48	0.48	0.82
Tcells.Cytotoxic.MCP_Nature.2020.PMID.31942075	0.96	0.72	1.27	0.76	0.90
Tcells.gammadelta_Nat.Methods.2015.PMID.25822800	0.96	0.72	1.28	0.78	0.91
Tcells.helper_Immunity.2013.PMID.24138885	0.89	0.67	1.18	0.42	0.78
Tcells.MCP_Nature.2020.PMID.31942077	0.88	0.66	1.18	0.40	0.77
Tcells.regulatory.2gene_Nature.2020.PMID.31942077	1.01	0.76	1.33	0.97	0.98
Tcells.Tregs_Nat.Methods.2015.PMID.25822800	0.99	0.74	1.32	0.94	0.97
TCGA.BRCA.1198_BASAL_JCI.2020.PMID.32573490	1.66	1.19	2.32	0.003	0.16
TCGA.BRCA.1198_Chromogranin_JCI.2020.PMID.32573490	1.14	0.86	1.50	0.36	0.77
TCGA.BRCA.1198_COLLAGEN11A_JCI.2020.PMID.32573490	1.01	0.75	1.37	0.93	0.97
TCGA.BRCA.1198_EN1_FDZ9_JCI.2020.PMID.32573490	1.08	0.76	1.52	0.67	0.88
TCGA.BRCA.1198_FGFR4_EGF_JCI.2020.PMID.32573490	0.93	0.70	1.24	0.63	0.87
TCGA.BRCA.1198_HISTONES_JCI.2020.PMID.32573490	1.46	1.04	2.05	0.03	0.37
TCGA.BRCA.1198_HOXC11_HOTAIR_SIX1_JCI.2020.PMID.32573490	1.01	0.76	1.35	0.93	0.97
TCGA.BRCA.1198_IL8_CCL_JCI.2020.PMID.32573490	0.77	0.56	1.05	0.10	0.55
TCGA.BRCA.1198_immune_CD19_JCI.2020.PMID.32573490	0.95	0.69	1.29	0.73	0.89
TCGA.BRCA.1198_immune_CD34_TIE1_JCI.2020.PMID.32573490	1.25	0.89	1.76	0.19	0.65
TCGA.BRCA.1198_immune_CD4_CD53_CD84_BTK_JCI.2020.PMID.32573490	0.88	0.65	1.18	0.38	0.77
TCGA.BRCA.1198_immune_CD8_GZMK_JCI.2020.PMID.32573490	0.93	0.69	1.24	0.60	0.85
TCGA.BRCA.1198_immune_CTLA4_CXCL_FOXP3_JCI.2020.PMID.32573490	0.83	0.60	1.13	0.24	0.70
TCGA.BRCA.1198_immune_FOS_JUN_IL6_JCI.2020.PMID.32573490	1.09	0.86	1.37	0.48	0.82
TCGA.BRCA.1198_immune_GIMAP_IL16_JCI.2020.PMID.32573490	1.05	0.80	1.38	0.73	0.89
TCGA.BRCA.1198_immune_HLA_A_F_JCI.2020.PMID.32573490	1.00	0.77	1.30	>0.99	>0.99
TCGA.BRCA.1198_immune_HLA_D_JCI.2020.PMID.32573490	0.90	0.69	1.17	0.43	0.79
TCGA.BRCA.1198_immune_INTERFERON_JCI.2020.PMID.32573490	1.10	0.83	1.48	0.50	0.82
TCGA.BRCA.1198_IMMUNE1_JCI.2020.PMID.32573490	0.82	0.60	1.13	0.22	0.69
TCGA.BRCA.1198_LUMINAL_JCI.2020.PMID.32573490	0.98	0.64	1.49	0.91	0.97
TCGA.BRCA.1198_MYBL2_APOBEC3B_JCI.2020.PMID.32573490	0.75	0.53	1.07	0.11	0.56
TCGA.BRCA.1198_NORMAL_JCI.2020.PMID.32573490	1.46	1.04	2.07	0.03	0.37
TCGA.BRCA.1198_NORMAL2_JCI.2020.PMID.32573490	0.95	0.70	1.30	0.76	0.90
TCGA.BRCA.1198_PDCHA_MANY_JCI.2020.PMID.32573490	1.05	0.79	1.40	0.75	0.90
TCGA.BRCA.1198_S100A7_8_9_JCI.2020.PMID.32573490	1.07	0.76	1.49	0.71	0.88
TCGA.BRCA.1198_TP63_JCI.2020.PMID.32573490	1.57	1.15	2.15	0.004	0.18
TCGA.BRCA.1198.IMMUNOGLOBULIN_JCI.2020.PMID.32573490	0.81	0.60	1.10	0.17	0.64
TCGA.CSF1.response_Immunity.2018.PMID.29628290	0.86	0.64	1.15	0.30	0.74
TCGA.IFN.score_Immunity.2018.PMID.29628290	1.10	0.81	1.48	0.55	0.84
TCGA.Liexpression.score_Immunity.2018.PMID.29628290	0.97	0.73	1.29	0.82	0.93
TCGA.Serum.response.up_Immunity.2018.PMID.29628290	0.89	0.68	1.15	0.37	0.77
TCGA.TFH_Immunity.2018.PMID.29628290	1.08	0.83	1.40	0.55	0.84
TCGA.Tgd_Immunity.2018.PMID.29628290	1.36	0.98	1.88	0.07	0.47
TCGA.TGFB.score_Immunity.2018.PMID.29628290	1.21	0.88	1.65	0.24	0.70
Tcm_Immunity.2013.PMID.24138885	0.82	0.60	1.13	0.22	0.69
Tem_Immunity.2013.PMID.24138885	1.04	0.77	1.40	0.82	0.93
TFH_Immunity.2013.PMID.24138885	1.08	0.83	1.40	0.55	0.84
Tgd_Immunity.2013.PMID.24138885	1.36	0.98	1.88	0.07	0.47
Th1_cells_Immunity.2013.PMID.24138885	0.98	0.72	1.33	0.88	0.96
Th17_cells_Immunity.2013.PMID.24138885	1.28	0.91	1.80	0.16	0.64

Th2_cells_Immunity.2013.PMID.24138885	0.81	0.60	1.08	0.15	0.64
TLS.9Gene.Signature_Nature.2020.PMID.31942071	1.06	0.81	1.39	0.68	0.88
TLS.CXCL13.SingleGene_Nature.2020.PMID.31942077	0.79	0.59	1.06	0.12	0.57
TLS.Hallmark.Gene.Signature_Nature.2020.PMID.31942071	0.92	0.71	1.20	0.54	0.84
TLS.Known.Markers_Nature.2020.PMID.31942071	0.87	0.65	1.15	0.32	0.74
TLS.Structure.12chemokine_FrontImmunol.2017.PMID.28713385	0.85	0.64	1.14	0.29	0.74
TLS.tumors.wTLS.and.CD8.vs.CD8alone_Nature.2020.PMID.31942071	0.95	0.71	1.27	0.73	0.89
TNBC.good.prognosis.TNBC.230genes_BCR.2011.PMID.21978456	0.95	0.73	1.24	0.70	0.88
TNBC.good.prognosis.TNBC.26genes_BCR.2011.PMID.21978456	1.05	0.78	1.42	0.73	0.89
TNBC.metastasis.free.survival_PLoS.One.2013.PMID.24349199	0.77	0.57	1.05	0.10	0.55
TNBC.poor.prognosis.TNBC.26genes_BCR.2011.PMID.21978456	0.88	0.67	1.15	0.34	0.75
Translation.Pathway_CancerImmunolRes.2018.PMID.30266715	1.05	0.82	1.36	0.69	0.88
Tumour.hypoxia.causes.DNA.hypermethylation_Nature.2016.PMID.27533040	1.02	0.75	1.38	0.91	0.97
Type.1.T.helper.cell_CellRep.2017.PMID.28052254	0.87	0.65	1.16	0.34	0.74
Type.17.T.helper.cell_CellRep.2017.PMID.28052254	0.92	0.69	1.22	0.56	0.84
Type.2.T.helper.cell_CellRep.2017.PMID.28052254	0.83	0.65	1.06	0.14	0.64
Up.Basal.High_Nat.Cell.Biol.2014.PMID.25173976	1.40	1.02	1.92	0.04	0.37
Up.Proliferation_Nat.Cell.Biol.2014.PMID.25173976	0.82	0.60	1.11	0.19	0.65
Upregulated.by.oncogenic.NRAS.basal_Cell.Rep.2016.PMID.26166574	1.36	1.00	1.84	0.05	0.45
Upregulated.upon.NRAS.repression.basal_Cell.Rep.2017.PMID.26166574	1.15	0.85	1.54	0.36	0.77
Vascular.Content_Clin.Exp.Metastasis.2014.PMID.23975155	1.47	1.04	2.09	0.03	0.37
VEGF.13genes_BMC.Med.2009.PMID.19291283	0.93	0.68	1.27	0.65	0.88
Wirapati.Proliferation_BCR.2008.PMID.18662380	0.92	0.68	1.25	0.59	0.85
Wound.Signature_CCR.2009.PMID.19887484	1.75	1.25	2.45	0.001	0.13
X11q13.Amplicon_BMC.Med.Genomics.2011.PMID.21214954	1.02	0.80	1.30	0.90	0.96
X12qMDM4.BMC.Med.Genomics.2011.PMID.21214954	1.06	0.80	1.40	0.70	0.88
X13q14.Amplicon_BMC.Med.Genomics.2011.PMID.21214954	0.90	0.71	1.14	0.37	0.77
X15q25.Amplicon_BMC.Med.Genomics.2011.PMID.21214954	0.77	0.59	1.00	0.05	0.45
X16.13.Amplicon_BMC.Med.Genomics.2011.PMID.21214954	0.97	0.72	1.29	0.81	0.93
X16q23.Amplicon_BMC.Med.Genomics.2011.PMID.21214954	0.82	0.64	1.04	0.10	0.55
X17PP13.Amplicon_BMC.Med.Genomics.2011.PMID.21214954	0.90	0.67	1.22	0.51	0.83
X17q25x.BMC.Med.Genomics.2011.PMID.21214954	0.73	0.55	0.97	0.03	0.37
X19p13.Amplicon_BMC.Med.Genomics.2011.PMID.21214954	0.96	0.73	1.25	0.74	0.90
X1p36.Amplicon_BMC.Med.Genomics.2011.PMID.21214954	1.02	0.78	1.33	0.91	0.97
X3p21.Amplicon_BMC.Med.Genomics.2011.PMID.21214954	0.98	0.75	1.28	0.89	0.96
X4p16.Amplicon_BMC.Med.Genomics.2011.PMID.21214954	0.87	0.65	1.15	0.32	0.74
X5Q_BCRT.2012.PMID.22048815	0.76	0.59	0.97	0.02	0.37
X8p.Amplicon_BMC.Med.Genomics.2011.PMID.21214954	0.98	0.73	1.32	0.89	0.96
X8p22.Amplicon_BMC.Med.Genomics.2011.PMID.21214954	0.99	0.74	1.32	0.93	0.97
XBP1.Signature_Nature.2014.PMID.24670641	0.74	0.56	0.98	0.04	0.37

NSABP B-41					
Signature	HR	95% CI		*P*	adjusted *P*
Activate.Endothelium_Clin.Exp.Metastasis.2014.PMID.23975155	0.68	0.43	1.09	0.11	0.79
Activated.B.cell_CellRep.2017.PMID.28052254	0.78	0.52	1.16	0.22	0.79
Activated.Blood.Neutrophil.Signature_Nat.Cell.Biol.2019.PMID.31263265	1.00	0.62	1.61	>0.99	>0.99
Activated.Cancer.Cell.Signature_Nat.Cell.Biol.2019.PMID.31263265	0.74	0.48	1.15	0.18	0.79
Activated.CD4.T.cell_CellRep.2017.PMID.28052254	1.03	0.69	1.53	0.90	>0.99
Activated.CD8.T.cell_CellRep.2017.PMID.28052254	0.82	0.54	1.24	0.34	0.82
Activated.dendritic.cell_CellRep.2017.PMID.28052254	0.47	0.28	0.80	0.01	0.79
Activated.Lung.MSC.Signature_Nat.Cell.Biol.2019.PMID.31263265	0.94	0.61	1.43	0.76	0.98
Activated.Lung.Neutrophil.Signature_Nat.Cell.Biol.2019.PMID.31263265	1.23	0.81	1.86	0.33	0.80
aDC_Immunity.2013_PMID.24138885.PMID.24138885	0.95	0.64	1.40	0.80	0.98
ADM.S100A10.A110NDGR1.Cluster_BMC.Med.Genomics.2011.PMID.21214954	1.28	0.83	1.99	0.27	0.80
African.and.European.Ancestry.TCGA.Negative_JAMA.Oncol.2017.PMID.28472234	0.65	0.39	1.09	0.10	0.79
African.and.European.Ancestry.TCGA.Positive_JAMA.Oncol.2017.PMID.28472234	1.22	0.78	1.90	0.38	0.83
Age.associated.signature_Genome.Biol.2015.PMID.26343147	1.01	0.62	1.63	0.98	>0.99
aMaSC_BCR.2010.PMID.20346151	0.74	0.46	1.22	0.24	0.79
aMaSC.HsEnriched_BCR.2015.PMID.25575446	0.80	0.47	1.36	0.40	0.83
aMaSC.HsEnriched.Refined1_BCR.2015.PMID.25575446	0.97	0.62	1.53	0.90	>0.99
aMaSC.Lim09_BCR.2015.PMID.25575446	0.69	0.41	1.16	0.17	0.79
aMaSC.Prat_BCR.2015.PMID.25575446	0.49	0.29	0.81	0.01	0.79
aMaSC.Shehata_BCR.2015.PMID.25575446	0.61	0.36	1.03	0.07	0.79
aMaSC.Signature_Cell.Stem.Cell.2012.PMID.22305568	0.89	0.57	1.39	0.61	0.91
AMPH.EPIREGULIN.Cluster_BMC.Med.Genomics.2011.PMID.21214954	1.10	0.59	2.05	0.77	0.98
Amplification.50_Genome.Biol.2014.PMID.25164602	1.18	0.79	1.75	0.42	0.83
Amplification.50.better.than._Genome.Biol.2015.PMID.25164602	1.20	0.83	1.74	0.33	0.80
Apocrine.Features_J.Pathol.2017.PMID.27861902	1.54	0.97	2.45	0.07	0.79
aStr.HsEnriched_BCR.2015.PMID.25575446	0.75	0.47	1.20	0.23	0.79
aStr.HsEnriched.Refined1_BCR.2015.PMID.25575446	0.96	0.58	1.61	0.89	>0.99
aStr.HsEnriched.Refined2_BCR.2015.PMID.25575446	0.75	0.48	1.18	0.22	0.79
aStr.Lim09_BCR.2015.PMID.25575446	0.82	0.50	1.34	0.44	0.83
aStr.Prat_BCR.2015.PMID.25575446	0.72	0.45	1.15	0.17	0.79
aStr.Shehata_BCR.2015.PMID.25575446	0.79	0.49	1.27	0.33	0.80
BASAL.Cluster_BMC.Med.Genomics.2011.PMID.21214954	0.74	0.44	1.27	0.28	0.80
Bcell.cluster_CCR.2014.PMID.24916698	0.78	0.53	1.12	0.18	0.79
Bcell.IL10.MINUS_Immunol.2014.PMID.25080484	0.85	0.56	1.29	0.44	0.83
Bcell.IL10.PLUS_Immunol.2014.PMID.25080484	0.93	0.60	1.43	0.72	0.96
Bcell.lineage.MCP_Nature.2020.PMID.31942077	0.69	0.46	1.04	0.07	0.79
Bcell.Plasma.52gene_Genome.Biol.2013.PMID.23618380	0.81	0.54	1.21	0.30	0.80
Bcell.Plasma.Metagene_Genome.Biol.2013.PMID.23618380	0.80	0.54	1.17	0.25	0.79
Bcell.Tcell.Cooperation_Cell.2019.PMID.31730857	0.82	0.55	1.22	0.33	0.80

Bcells_CancerImmunolRes.2018.PMID.30266715	0.78	0.55	1.11	0.17	0.79
Bcells_Immunity.2013.PMID.24138885	0.79	0.51	1.21	0.28	0.80
Bcells.Centroblast_JCO.2015.PMID.25800755	1.04	0.67	1.63	0.86	0.99
Bcells.Centrocyte_JCO.2015.PMID.25800755	0.83	0.54	1.27	0.39	0.83
Bcells.Memory_JCO.2015.PMID.25800755	0.96	0.59	1.57	0.88	>0.99
Bcells.memory_Nat.Methods.2015.PMID.25822800	0.81	0.55	1.20	0.30	0.80
Bcells.Naive_JCO.2015.PMID.25800755	0.85	0.53	1.34	0.48	0.86
Bcells.naive_Nat.Methods.2015.PMID.25822800	0.79	0.52	1.18	0.24	0.79
Bcells.Plasmablast_JCO.2015.PMID.25800755	1.02	0.68	1.53	0.92	>0.99
Blood.vessels_Immunity.2013.PMID.24138885	0.96	0.54	1.71	0.89	>0.99
bMYB.Signature_Oncogene.2009.PMID.19043454	1.00	0.64	1.57	>0.99	>0.99
C3TAG.Responding_CCR.2013.PMID.23780888	0.83	0.49	1.40	0.48	0.86
C3TAG.Untreated_CCR.2013.PMID.23780888	0.89	0.54	1.47	0.65	0.91
CD103.Negative_Cancer.Cell.2014.PMID.25446897	0.77	0.52	1.14	0.19	0.79
CD103.Positive_Cancer.Cell.2014.PMID.25446897	0.82	0.56	1.21	0.31	0.80
CD103.Ratio_Cancer.Cell.2014.PMID.25446897	0.60	0.38	0.94	0.03	0.79
CD274_Single_Gene.Single	0.75	0.50	1.13	0.17	0.79
CD34.CD36.Cluster_BMC.Med.Genomics.PMID.21214954	0.86	0.46	1.59	0.62	0.91
CD44.downregulated.genes_Cancer.Cell.2007.PMID.17349583	1.25	0.86	1.82	0.25	0.79
CD44.upregulated.genes_Cancer.Cell.2007.PMID.17349583	1.28	0.91	1.82	0.16	0.79
CD56bright.natural.killer.cell_CellRep.2017.PMID.28052254	1.01	0.68	1.51	0.95	>0.99
CD56dim.natural.killer.cell_CellRep.2017.PMID.28052254	1.00	0.62	1.59	0.99	>0.99
CD68.cluster_CCR.2014.PMID.24916698	0.98	0.60	1.61	0.94	>0.99
CD8.cluster_CCR.2014.PMID.24916698	0.82	0.54	1.23	0.33	0.80
CDKN2A_Single_Gene.Single	1.34	0.89	2.01	0.16	0.79
Central.memory.CD4.T.cell_CellRep.2017.PMID.28052254	0.59	0.37	0.92	0.02	0.79
Central.memory.CD8.T.cell_CellRep.2017.PMID.28052254	0.97	0.62	1.52	0.90	>0.99
CES.Score_CCR.2017.PMID.27903675	1.11	0.65	1.88	0.71	0.95
Chromogramin_BMC.Med.Genomics.2011.PMID.21214954	0.85	0.54	1.34	0.48	0.86
CIN70_Nat.Genet.2006.PMID.16921376	1.04	0.66	1.65	0.86	0.99
Claudin.High_Genome.Biol.2007.PMID.17493263	0.69	0.44	1.09	0.11	0.79
Claudin.Low_Genome.Biol.2007.PMID.17493263	1.31	0.74	2.32	0.36	0.83
Claudin.Low.29_Cancer.Res.2009.PMID.19435916	1.02	0.58	1.79	0.96	>0.99
cMYB.Signature_PLoS.One.2010.PMID.20949095	0.77	0.49	1.21	0.26	0.79
CORE.Bcell.signature.Garber_Cell.Mol.Gastroenterol.Hepatol.2017.PMID.28508029	0.64	0.40	1.01	0.06	0.79
CTLA4_Single_Gene.Single	0.82	0.56	1.19	0.30	0.80
Cytolytic.activity_Cell.2015.PMID.25594174	0.78	0.49	1.22	0.27	0.80
Cytotoxic.cells_Immunity.2013.PMID.24138885	0.82	0.51	1.32	0.42	0.83
Day7.Downregulated_Nat.Cell.Biol.2014.PMID.25173976	0.83	0.51	1.34	0.44	0.83
Day7.Upregulated_Nat.Cell.Biol.2014.PMID.25173976	0.86	0.56	1.32	0.49	0.86
DC_Immunity.2013.PMID.24138885	0.66	0.45	0.98	0.04	0.79
DCIS.HGF.down_BCR.2013.PMID.24025166	0.65	0.43	0.99	0.04	0.79
DCIS.HGF.up_BCR.2014.PMID.24025166	0.67	0.43	1.04	0.07	0.79
Delection.50_Genome.Biol.2016.PMID.25164602	1.08	0.69	1.71	0.73	0.96
Delection.50.better.than_Genome.Biol.2017.PMID.25164602	1.13	0.73	1.74	0.59	0.90
Dendritic.cells.activated_Nat.Methods.2015.PMID.25822800	0.80	0.52	1.24	0.32	0.80
Dendritic.cells.resting_Nat.Methods.2015.PMID.25822800	0.61	0.38	0.99	0.05	0.79
Down.Basal.High_Nat.Cell.Biol.2014.PMID.25173976	0.67	0.40	1.15	0.15	0.79
Down.CLOW.High_Nat.Cell.Biol.2014.PMID.25173976	1.06	0.66	1.72	0.81	0.98

Downregulated.upon.NRAS.repression.basal_Cell.Rep.2015.PMID.26166574	0.78	0.51	1.19	0.25	0.79
Ductal.Carcinoma.In.Situ_J.Pathol.2017.PMID.27861902	0.85	0.59	1.23	0.39	0.83
Duke.Module01.acidosis_PNASUSA.2010.PMID.20335537	0.70	0.42	1.15	0.16	0.79
Duke.Module02.akt_PNASUSA.2010.PMID.20335537	0.69	0.43	1.11	0.12	0.79
Duke.Module03.betacatenin_PNASUSA.2010.PMID.20335537	1.00	0.65	1.52	0.99	>0.99
Duke.Module04.E2F1_PNASUSA.2010.PMID.20335537	0.88	0.55	1.41	0.61	0.91
Duke.Module05.EGFR_PNASUSA.2010.PMID.20335537	0.67	0.39	1.15	0.14	0.79
Duke.Module06.ER_PNASUSA.2010.PMID.20335537	1.07	0.68	1.69	0.78	0.98
Duke.Module07.glucosedepletion_PNASUSA.2010.PMID.20335537	0.96	0.63	1.45	0.83	0.98
Duke.Module08.HER2_PNASUSA.2010.PMID.20335537	0.88	0.55	1.41	0.60	0.91
Duke.Module09.hypoxia_PNASUSA.2010.PMID.20335537	1.22	0.76	1.94	0.41	0.83
Duke.Module10.IFNA_PNASUSA.2010.PMID.20335537	1.34	0.87	2.07	0.19	0.79
Duke.Module11.IFNG_PNASUSA.2010.PMID.20335537	1.19	0.78	1.83	0.42	0.83
Duke.Module12.lacticacidosis_PNASUSA.2010.PMID.20335537	1.14	0.73	1.79	0.55	0.88
Duke.Module13.myc_PNASUSA.2010.PMID.20335537	0.99	0.63	1.53	0.95	>0.99
Duke.Module14.p53_PNASUSA.2010.PMID.20335537	0.97	0.59	1.58	0.90	>0.99
Duke.Module15.p63_PNASUSA.2010.PMID.20335537	0.75	0.47	1.22	0.24	0.79
Duke.Module16.pi3k_PNASUSA.2010.PMID.20335537	0.88	0.55	1.39	0.57	0.89
Duke.Module17.PR_PNASUSA.2010.PMID.20335537	1.04	0.57	1.89	0.90	>0.99
Duke.Module18.ras_PNASUSA.2010.PMID.20335537	0.82	0.56	1.21	0.32	0.80
Duke.Module19.src_PNASUSA.2010.PMID.20335537	1.39	0.89	2.17	0.14	0.79
Duke.Module20.STAT3_PNASUSA.2010.PMID.20335537	0.71	0.45	1.11	0.14	0.79
Duke.Module21.TGFB_PNASUSA.2010.PMID.20335537	0.69	0.43	1.12	0.13	0.79
Duke.Module22.TNFA_PNASUSA.2010.PMID.20335537	1.01	0.67	1.54	0.95	>0.99
Durvalumab.signature_CCR.2018.PMID.29716923	0.92	0.56	1.50	0.73	0.96
Early.IRS.1_PLoS.One.2016.PMID.26991655	0.94	0.57	1.56	0.81	0.98
Early.IRS.2_PLoS.One.2016.PMID.26991655	0.95	0.63	1.43	0.81	0.98
Early.Relapse.ERPos.33genes_JAMA.2011.PMID.21558518	1.28	0.85	1.93	0.24	0.79
Early.Response.ERNeg.27genes_JAMA.2011.PMID.21558518	1.40	0.90	2.18	0.14	0.79
Effector.memeory.CD4.T.cell_CellRep.2017.PMID.28052254	0.91	0.59	1.38	0.65	0.91
Effector.memeory.CD8.T.cell_CellRep.2017.PMID.28052254	0.70	0.45	1.08	0.11	0.79
EGFR_Single_Gene.Single	1.87	1.09	3.21	0.02	0.79
EMT.down.Taube_PNAS.2010.PMID.20713713	0.66	0.41	1.06	0.08	0.79
EMT.down.Weingberg_PNAS.2010.PMID.20713713	0.74	0.46	1.18	0.21	0.79
EMT.up.Taube_PNAS.2010.PMID.20713713	0.89	0.57	1.38	0.59	0.90
EMT.up.Weinberg_PNAS.2010.PMID.20713713	0.93	0.61	1.42	0.74	0.97
Endothelial.cells.MCP_Nature.2020..PMID.31942077	1.02	0.58	1.81	0.93	>0.99
Endothelial.Normal_Angiogenesis.2014.PMID.24257808	1.06	0.65	1.74	0.82	0.98
Endothelial.Tumor_Angiogenesis.2014.PMID.24257808	0.66	0.43	1.00	0.05	0.79
Eosinophil_CellRep.2017.PMID.28052254	0.75	0.51	1.12	0.16	0.79
Eosinophils_Immunity.2013.PMID.24138885	1.11	0.73	1.71	0.62	0.91
Eosinophils_Nat.Methods.2015.PMID.25822800	0.64	0.41	1.00	0.05	0.79
Epithelial.Tubule.Formation_J.Pathol.2017.PMID.27861902	1.19	0.73	1.94	0.48	0.86
ERBB2_Single_Gene.Single	0.93	0.61	1.42	0.73	0.96
ERBB3_Single_Gene.Single	2.00	1.15	3.49	0.01	0.79
ESR1_Single_Gene.Single	1.05	0.51	2.17	0.88	>0.99
ESTIMATE.Immune_Nat.Commun.2013.PMID.24113773	0.69	0.44	1.10	0.12	0.79
ESTIMATE.Stromal_Nat.Commun.2013.PMID.24113773	0.79	0.50	1.24	0.31	0.80
Euclidean.Distance.CLOW_BCR.2010.PMID.20813035	1.18	0.73	1.91	0.49	0.86

EXTENDED.Bcell.signature.Garber_Cell.Mol.Gastroenterol.Hepatol.2017.PMID.28508029	0.67	0.44	1.01	0.06	0.79
FGFR4_Single_Gene.Single	1.14	0.70	1.86	0.61	0.91
FGFR4.Induced_JCI.2020.PMID.32573490	0.84	0.55	1.28	0.42	0.83
FGFR4.Repressed_JCI.2020.PMID.32573490	1.04	0.70	1.56	0.83	0.98
Fibrinogen.Cluster_BMC.Med.Genomics.2011.PMID.21214954	0.67	0.44	1.03	0.07	0.79
Fibroblast.Cluster_BMC.Med.Genomics.2011.PMID.21214954	0.93	0.61	1.43	0.75	0.97
Fibroblasts.MCP_Nature.2020.PMID.31942077	0.72	0.47	1.10	0.13	0.79
Fibromatosis_Lab.Invest.2008.PMID.18414401	0.82	0.55	1.22	0.32	0.80
fMaSC.Metab_CellRep.2018.PMID.30089273	1.44	0.81	2.55	0.22	0.79
fMaSC.Metab8_CellRep.2018.PMID.30089273	1.22	0.72	2.05	0.46	0.85
fMaSC.refined1_BCR.2015.PMID.25575446	0.65	0.39	1.08	0.09	0.79
fMasC.Signature_Cell.Stem.Cell.2012.PMID.22305568	0.71	0.45	1.13	0.15	0.79
fMaSC.Signature_CellRep.2018.PMID.30089273	1.01	0.65	1.57	0.97	>0.99
FOS.JUN_Cluster_BMC.Med.Genomics.2011.PMID.21214954	1.13	0.72	1.79	0.59	0.90
FOXC1.Hair.Follicles.P30C.LO.vs.WT.Negative_Science.2016.PMID.26912704	1.02	0.67	1.57	0.92	>0.99
FOXC1.Hair.Follicles.P30C.LO.vs.WT.Positive_Science.2016.PMID.26912704	0.77	0.51	1.16	0.21	0.79
fSTR.Signature_Cell.Stem.Cell.2012.PMID.22305568	0.86	0.45	1.63	0.65	0.91
Gamma.delta.T.cell_CellRep.2017.PMID.28052254	0.76	0.50	1.15	0.20	0.79
GATA3.induced.genes_JCO.2006.PMID.16505416	0.73	0.43	1.26	0.26	0.79
GATA3.induced.genes_Oncogene.2004.PMID.15361840	0.62	0.36	1.07	0.09	0.79
GDF11.TGFBR3_Nat.Cell.Biol.2014.PMID.24658685	1.10	0.75	1.60	0.63	0.91
Glycolysis_BMC.Med.2009.PMID.19291283	0.83	0.54	1.28	0.41	0.83
GO.DOWN.with.SOX10.OE_Cell.Rep.2015.PMID.26365194	0.78	0.53	1.16	0.22	0.79
GO.UP.with.SOX10.OE_Cell.Rep.2015.PMID.26365194	0.72	0.43	1.19	0.20	0.79
GSEA_BIOCARTA_ALK_PATHWAY.PMID.16199517	0.86	0.54	1.38	0.54	0.88
GSEA_BIOCARTA.AKT.PATHWAY.PMID.16199517	0.79	0.54	1.14	0.21	0.79
GSEA_BIOCARTA.BRCA.ATR.PATHWAY.ATRBRCA.PMID.16199517	0.99	0.61	1.61	0.97	>0.99
GSEA_BIOCARTA.CASPASE.PATHWAY.PMID.16199517	1.03	0.67	1.57	0.91	>0.99
GSEA_BIOCARTA.CTLA4.PATHWAY.PMID.16199517	0.83	0.56	1.24	0.37	0.83
GSEA_BIOCARTA.IGF1R.PATHWAY.PMID.16199517	0.79	0.49	1.27	0.33	0.80
GSEA_BIOCARTA.MTOR.PATHWAY.PMID.16199517	0.95	0.62	1.46	0.82	0.98
GSEA_BIOCARTA.PTEN.PATHWAY.PMID.16199517	0.76	0.48	1.20	0.24	0.79
GSEA_BIOCARTA.RAS.PATHWAY.PMID.16199517	0.88	0.58	1.35	0.56	0.88
GSEA_BIOCARTA.RB.PATHWAY.PMID.16199517	1.10	0.67	1.79	0.72	0.96
GSEA_BIOCARTA.VEGF.PATHWAY.PMID.16199517	1.42	0.91	2.24	0.13	0.79
GSEA_HALLMARK.MYC.TARGETS.V1.PMID.16199517	1.03	0.68	1.58	0.88	>0.99
GSEA_HELLER.HDAC.TARGETS.DOWN.PMID.16199517	0.82	0.54	1.25	0.35	0.82
GSEA_NELSON.RESPONSE.TO.ANDROGEN.UP.PMID.16199517	0.88	0.58	1.34	0.56	0.88
GSEA_REACTOME.PD1.SIGNALING.PMID.16199517	0.65	0.42	1.00	0.05	0.79
GSEA_REACTOME.PI3K.CASCADE.PMID.16199517	1.27	0.78	2.08	0.33	0.80
GSEA_RETINOL.METABOLISM.KEGG.PMID.16199517	0.80	0.46	1.40	0.44	0.83
GSEA.GP1_Proliferation.DNA.repair..PUJANA.CHEK2.PCC.NETWORK.PMID.25109877	0.98	0.64	1.50	0.94	>0.99
GSEA.GP1_Proliferation.DNA.repair.REACTOME.CELL.CYCLE.MITOTIC.PMID.25109877	1.05	0.69	1.59	0.84	0.98
GSEA.GP10_Fatty.acid.oxidation.CARBOXYLIC.ACID.METABOLIC.PROCESS.PMID.25109877	1.27	0.77	2.08	0.35	0.82
GSEA.GP11_Immune.IFN.PerouLab.PMID.25109877	1.30	0.85	1.99	0.23	0.79
GSEA.GP12_Hypoxia.glycolosis.SEMENZA.HIF1.TARGETS.PMID.25109877	0.90	0.57	1.44	0.67	0.93
GSEA.GP13_Neural.signailng.MODULE100.PMID.25109877	0.68	0.42	1.11	0.13	0.79
GSEA.GP13_Neural.signailng.NERVOUS.SYSTEM.DEVELOPMENT.PMID.25109877	0.81	0.49	1.34	0.41	0.83
GSEA.GP14_Plasma.membrane.cell.cell.signaling.MORF.CNTN1.PMID.25109877	1.09	0.62	1.91	0.76	0.98

GSEA.GP15_EGF.signaling.NAGASHIMA.EGF.SIGNALING.UP.PMID.25109877	0.70	0.42	1.16	0.16	0.79
GSEA.GP16_Protein.kinase.signaling.MAPKs.INTRACELLULAR.SIGNALING.CASCADE.PMID.25109877	0.89	0.59	1.34	0.56	0.88
GSEA.GP16_Protein.kinase.signaling.MAPKs.REGULATION.OF.KINASE.ACTIVITY.PMID.25109877	0.85	0.55	1.31	0.47	0.86
GSEA.GP17_Basal.signaling.SMID.BREAST.CANCER.BASAL.UP.PMID.25109877	0.86	0.57	1.29	0.46	0.85
GSEA.GP18_Vesicle.EPR.MEMBRANE.COAT.PMID.25109877	1.39	0.87	2.19	0.16	0.79
GSEA.GP19_1Q.amplicon.PerouLab.PMID.25109877	1.21	0.79	1.83	0.38	0.83
GSEA.GP2_Immune.Tcell.Bcell.KEGG.HEMATOPOIETIC.CELL.LINEAGE.PMID.25109877	0.74	0.50	1.10	0.14	0.79
GSEA.GP2_Immune.Tcell.Bcell.PerouLab.PMID.25109877	0.78	0.50	1.20	0.25	0.79
GSEA.GP20_TAL1.Leukemia.erythropoiesis.GNF2.TAL1.PMID.25109877	0.67	0.41	1.07	0.10	0.79
GSEA.GP21_Anti.apoptosis.DNA.stability.MORF.BCL2.PMID.25109877	0.88	0.56	1.37	0.56	0.88
GSEA.GP21_Anti.apoptosis.DNA.stability.MORF.MT4.PMID.25109877	0.59	0.35	0.98	0.04	0.79
GSEA.GP21_Anti.apoptosis.DNA.stability.MORF.STK17A.PMID.25109877	0.83	0.56	1.23	0.36	0.83
GSEA.GP22_16Q22.24.amplicon.PerouLab.PMID.25109877	0.94	0.61	1.46	0.78	0.98
GSEA.GP3_Tumo.suppressing.miRNA.targets.GTTTGTT.MIR.495.PMID.25109877	0.65	0.38	1.11	0.12	0.79
GSEA.GP3_Tumor.suppressing.miRNA.targets.DACOSTA.UV.RESPONSE.VIA.ERCC3.DN.PMID.25109877	0.89	0.50	1.58	0.69	0.93
GSEA.GP3_Tumor.suppressing.miRNA.targets.TGCTTTG.MIR.330.PMID.25109877	0.76	0.48	1.22	0.25	0.79
GSEA.GP4_MES.ECM.PerouLab.PMID.25109877	0.85	0.57	1.27	0.42	0.83
GSEA.GP5_MYC.targets.TERT.PerouLab.PMID.25109877	1.16	0.70	1.92	0.56	0.88
GSEA.GP6_Squamous.differentiation.development.RICKMAN.TUMOR.DIFFERENTIATED.WELL.VS.POORLY.DN.PMID.251098	0.77	0.53	1.14	0.19	0.79
GSEA.GP7_Estrogen.signaling.SMID.BREAST.CANCER.BASAL.DN.PMID.25109877	0.96	0.63	1.47	0.86	0.99
GSEA.GP8_FOXO.stemness.MORF.PTPRB.PMID.25109877	0.69	0.45	1.05	0.09	0.79
GSEA.GP8_FOXO.stemness.TTGTTT.VSFOXO4.01.PMID.25109877	0.95	0.61	1.49	0.83	0.98
GSEA.GP9_Cell.cell.adhesion.PerouLab.PMID.25109877	1.07	0.62	1.84	0.80	0.98
HCK_BCR.2008.PMID.19272155	0.65	0.37	1.12	0.12	0.79
HER1.Cluster1_BMC.Genomics.2007.PMID.17663798	1.08	0.74	1.58	0.68	0.93
HER1.Cluster2_BMC.Genomics.2007.PMID.17663798	0.85	0.55	1.30	0.45	0.83
HER1.Cluster3_BMC.Genomics.2007.PMID.17663798	0.95	0.62	1.45	0.81	0.98
HER2.Amplicon.PerouLab_BMC.Med.Genomic.2011.PMID.21214954	0.52	0.28	0.95	0.03	0.79
Histological.Grade_J.Pathol.2017.PMID.27861902	1.00	0.63	1.60	0.99	>0.99
HouseKeeping_Genome.Biol.2004.PMID.15287981	1.01	0.70	1.46	0.96	>0.99
iDC.Median_Immunity.2013.PMID.24138885	0.76	0.55	1.05	0.10	0.79
IFN.Cluster_BMC.Med.Genomics.2011.PMID.21214954	1.43	0.94	2.20	0.10	0.79
IgG_BCR.2008.PMID.19272155	0.83	0.57	1.21	0.32	0.80
IGG.Cluster_BMC.Med.Genomics.2011.PMID.21214954	0.75	0.48	1.17	0.20	0.79
Immature..B.cell_CellRep.2017.PMID.28052254	0.73	0.46	1.16	0.19	0.79
Immature.dendritic.cell_CellRep.2017.PMID.28052254	0.94	0.59	1.52	0.81	0.98
ImmLandscape_Macro.mono.CSF1.core.response_CCR.2009.PMID.29628290	0.73	0.47	1.15	0.17	0.79
ImmLandscape_Wound.Healing_Immunity.2018.PMID.29628290	0.77	0.50	1.19	0.24	0.79
ImmLandscape.IFN3_Plos.One.2014.PMID.24516633	1.49	1.00	2.20	0.05	0.79
ImmLandscape.IFNG5_Plos.One.2014.PMID.24516633	0.84	0.56	1.26	0.40	0.83
ImmLandscape.lymphocyte.Infil.T.B.PMID.18592372	0.83	0.57	1.21	0.33	0.80
Immune.Hot.CD8.vs.Cold_Nature.2020.PMID.31942071	0.75	0.48	1.16	0.19	0.79
Immune.Perez.14_JCO.2015.PMID.25605861	0.60	0.39	0.94	0.02	0.79
Immune.Perez.87_JCO.2015.PMID.25605861	0.79	0.53	1.19	0.26	0.79
Immune.Suppression_JCI.Insight.2016.PMID.27699256	1.04	0.69	1.57	0.85	0.98
ImmuneActive_Cell.2019.PMID.31730857	0.76	0.49	1.19	0.23	0.79
Immunosuppression.PMID.31942077	0.91	0.61	1.36	0.66	0.92
IMS.Score_CCR.2018.PMID.29921729	0.83	0.56	1.24	0.36	0.83
Induced.in.Bcells_PNAS.2013.PMID.23382184	0.84	0.57	1.23	0.37	0.83

Induced.in.DC_PNAS.2013.PMID.23382184	0.92	0.58	1.47	0.73	0.97
Induced.in.GN_PNAS.2013.PMID.23382184	0.86	0.53	1.41	0.56	0.88
Induced.in.HSC_PNAS.2013.PMID.23382184	1.16	0.79	1.71	0.46	0.84
Induced.in.MOs_PNAS.2013.PMID.23382184	0.70	0.43	1.13	0.14	0.79
Induced.in.NKcells_PNAS.2013.PMID.23382184	0.86	0.53	1.40	0.54	0.88
Induced.in.Tcells_PNAS.2013.PMID.23382184	0.81	0.55	1.20	0.30	0.80
Inflammatory.breast.cancer.491genes_CCR.2013.PMID.23396049	0.82	0.55	1.24	0.35	0.82
Inflammatory.breast.cancer.79genes_CCR.2013.PMID.23396049	1.31	0.85	2.03	0.22	0.79
Inflammatory.breast.cancer.expressed.noIBC_79genes_CCR.2013.PMID.23396049	1.16	0.74	1.80	0.53	0.88
Inflammatory.breast.cancer.expressed.noIBC.491genes_CCR.2013.PMID.23396049	1.18	0.69	2.03	0.55	0.88
Influenza.11genes.Metasignature_Immunity.2015.PMID.26682989	1.12	0.71	1.78	0.62	0.91
Interferon_BCR.2008.PMID.19272155	1.53	1.04	2.24	0.03	0.79
Interferon.Pathway_CancerImmunolRes.2018.PMID.30266715	1.41	0.91	2.18	0.12	0.79
JUND.KRT5_Nat.Cell.Biol.2014.PMID.24658685	1.05	0.62	1.79	0.85	0.98
Keller2012.CD10.Adam_BCR.2015.PMID.25575446	0.84	0.54	1.30	0.44	0.83
KRAS.amplicon_Genome.Biology.2007.PMID.17493263	0.89	0.59	1.33	0.57	0.89
Late.IRS.1_PLoS.One.2016.PMID.26991655	1.28	0.88	1.84	0.19	0.79
Late.IRS.2_PLoS.One.2016.PMID.26991655	0.84	0.52	1.38	0.49	0.86
LCK_BCR.2008.PMID.19272155	0.73	0.48	1.11	0.14	0.79
Lim2009.LumProg.Adam_BCR.2015.PMID.25575446	1.05	0.66	1.65	0.85	0.98
Lim2009.MaSC.Adam_BCR.2015.PMID.25575446	0.73	0.45	1.19	0.21	0.79
Lim2009.MatureLum.Adam_BCR.2015.PMID.25575446	0.99	0.62	1.58	0.96	>0.99
Lim2009.Stroma.Adam_BCR.2015.PMID.25575446	0.81	0.49	1.32	0.39	0.83
Lim2010.LumProg.Adam_BCR.2015.PMID.25575446	0.90	0.62	1.29	0.55	0.88
Lim2010.MaSC.Adam_BCR.2015.PMID.25575446	0.64	0.38	1.07	0.09	0.79
Lim2010.MatureLum.Adam_BCR.2015.PMID.25575446	0.91	0.57	1.45	0.68	0.93
Lim2010.Stroma.Adam_BCR.2015.PMID.25575446	0.82	0.51	1.32	0.42	0.83
Lobular.Carcinoma.In.Situ_J.Pathol.2017.PMID.27861902	1.07	0.68	1.69	0.77	0.98
LOBULAR.TCGA.SIGNATURE.ImmuneCell.2015.PMID.26451490	0.82	0.50	1.35	0.43	0.83
LOBULAR.TCGA.SIGNATURE.Reactive_Cell.2015.PMID.26451490	0.71	0.39	1.32	0.28	0.80
LOBULAR.TCGA.SUBTYPE.Immune_Cell.2015.PMID.26451490	0.79	0.37	1.67	0.54	0.88
LOBULAR.TCGA.SUBTYPE.Proliferative_Cell.2015.PMID.26451490	1.02	0.60	1.75	0.93	>0.99
LOBULAR.TCGA.SUBTYPE.Reactive_Cell.2015.PMID.26451490	0.86	0.48	1.56	0.63	0.91
LTS.score_JCI.2020.PMID.32573490	0.88	0.54	1.44	0.61	0.91
Luminal_Progenitor_Up_Nat.Med.2009.PMID.19648928	0.79	0.49	1.28	0.34	0.81
Luminal.cluster_BMC.Med.Genomics.2011.PMID.21214954	1.12	0.70	1.77	0.64	0.91
Luminal.Progenitor_BCR.2010.PMID.20346151	0.68	0.42	1.10	0.12	0.79
Luminal.Progenitor.Down_Nat.Med.2009.PMID.19648928	0.87	0.62	1.21	0.40	0.83
LumProg.HsEnriched_BCR.2015.PMID.25575446	1.01	0.64	1.62	0.95	>0.99
LumProg.HsEnriched.Refined1_BCR.2015.PMID.25575446	0.78	0.48	1.27	0.32	0.80
LumProg.Lim09_BCR.2015.PMID.25575446	1.00	0.65	1.52	0.99	>0.99
LumProg.Prat_BCR.2015.PMID.25575446	0.81	0.51	1.29	0.37	0.83
LumProg.Shehata_BCR.2015.PMID.25575446	1.07	0.67	1.71	0.76	0.98
Lums.HER2E.DOWN.metastatic.signature_JCI.2020.PMID.32573490	0.84	0.53	1.32	0.44	0.83
Lums.HER2E.UP.metastatic.signature_JCI.2020.PMID.32573490	0.53	0.29	0.98	0.04	0.79
Lung.WNT_Cancer.Res.2009.PMID.19549913	1.09	0.71	1.69	0.68	0.93
Lymph.vessels_Immunity.2013.PMID.24138885	0.73	0.49	1.08	0.11	0.79
Lymphovascular.Invasion_J.Pathol.2017.PMID.27861902	0.81	0.57	1.17	0.26	0.79
M.D.Metagene_Genome.Biol.2013.PMID.23618380	0.62	0.40	0.98	0.04	0.79

M2.Macrophage_Blood.2006.PMID.16556895	0.66	0.41	1.05	0.08	0.79
Macrophage_CellRep.2017.PMID.28052254	0.68	0.45	1.04	0.08	0.79
Macrophages_CancerImmunolRes.2018.PMID.30266715	0.74	0.43	1.28	0.28	0.80
Macrophages_Immunity.2013.PMID.24138885	0.94	0.56	1.56	0.80	0.98
Macrophages.M0_Nat.Methods.2015.PMID.25822800	0.68	0.42	1.11	0.12	0.79
Macrophages.M1_Nat.Methods.2015.PMID.25822800	0.87	0.57	1.33	0.52	0.88
Macrophages.M2_Nat.Methods.2015.PMID.25822800	0.67	0.44	1.02	0.06	0.79
MacTh1.cluster_CCR.2014.PMID.24916698	0.71	0.45	1.13	0.15	0.79
MammaPrint_Nature.2002.PMID.11823860	1.01	0.64	1.59	0.96	>0.99
MAPK.pathway.activation_NPJ.Precis.Oncol.2018.PMID.29872725	0.99	0.60	1.64	0.98	>0.99
MASC.Down_Nat.Med.2009.PMID.19648928	1.29	0.72	2.34	0.39	0.83
MASC.Up_Nat.Med.2009.PMID.19648928	0.70	0.43	1.14	0.15	0.79
Mast.cell_CellRep.2017.PMID.28052254	0.72	0.48	1.09	0.12	0.79
Mast.cells_Immunity.2013.PMID.24138885	0.69	0.46	1.02	0.06	0.79
Mast.cells.activated_Nat.Methods.2015.PMID.25822800	0.68	0.42	1.09	0.11	0.79
Mast.cells.resting_Nat.Methods.2015.PMID.25822800	0.67	0.43	1.07	0.09	0.79
Mature.luminal_BCR.2010.PMID.20346151	0.97	0.61	1.53	0.90	>0.99
Mature.Luminal.Down_Nat.Med.2009.PMID.19648928	0.82	0.48	1.37	0.44	0.83
Mature.LuminaUp_Nat.Med.2009.PMID.19648928	0.94	0.60	1.48	0.79	0.98
MatureLum.HsEnriched_BCR.2015.PMID.25575446	0.92	0.54	1.56	0.75	0.97
MatureLum.HsEnriched.Refined1_BCR.2015.PMID.25575446	1.01	0.67	1.53	0.96	>0.99
MatureLum.Lim09_BCR.2015.PMID.25575446	1.04	0.61	1.75	0.89	>0.99
MatureLum.Prat_BCR.2015.PMID.25575446	1.03	0.61	1.75	0.90	>0.99
MatureLum.Shehata_BCR.2015.PMID.25575446	1.15	0.69	1.93	0.60	0.91
MBasal.Cluster_BMC.Med.Genomics.2011.PMID.21214954	0.71	0.44	1.15	0.17	0.79
MCD3.CD8_BMC.Med.Genomics.2011.PMID.21214954	0.77	0.51	1.17	0.22	0.79
MCF7.E2.induced.genes_JCO.2006.PMID.16505416	0.84	0.57	1.25	0.40	0.83
MCF7.E2.repressed.genes_JCO.2006.PMID.16505416	0.94	0.62	1.44	0.78	0.98
MDSC_CellRep.2017.PMID.28052254	0.67	0.41	1.08	0.10	0.79
MDSC.Granulocytic_Leukoc.Biol.2012.PMID.21954284	1.01	0.69	1.47	0.97	>0.99
MDSC.Neutrophi_Leukoc.Biol.2012.PMID.21954284	0.89	0.58	1.38	0.61	0.91
MDSC.tumor_J.Immunol.2012.PMID.23152559	0.80	0.51	1.26	0.33	0.80
MDSC.tumor.MO_J.Immunol.2012.PMID.23152559	0.73	0.45	1.16	0.18	0.79
MECM_BMC.Med.Genomics.2011.PMID.21214954	0.89	0.55	1.42	0.62	0.91
Memory.B.cell_CellRep.2017.PMID.28052254	0.76	0.52	1.13	0.18	0.79
MET.DOWN.RNAseq.Significant.Genes_JCI.2018.PMID.29480819	0.91	0.58	1.42	0.66	0.92
MET.DOWN.Significant.Genes.Low.Basal.1_JCI.2018.PMID.29480819	0.97	0.64	1.46	0.88	>0.99
MET.DOWN.Significant.Genes.Low.Basal.2_JCI.2018.PMID.29480819	0.84	0.54	1.31	0.43	0.83
MET.UP.RNAseq.Significant.Genes_JCI.2018.PMID.29480819	1.31	0.77	2.25	0.32	0.80
MET.UP.Significant.Genes.HIGH.BASALS.Genes_JCI.2018.PMID.29480819	0.95	0.63	1.44	0.82	0.98
Metaplastic.Up_CanRes.2009.PMID.19435916	0.71	0.43	1.18	0.18	0.79
Metastasis.predictor.TNBC_BCR.2010.PMID.20946665	0.77	0.49	1.21	0.26	0.79
MFGFR2_BMC.Med.Genomics.2011.PMID.21214954	1.14	0.74	1.77	0.56	0.88
MHC.Forero.11_Cancer.Immunol.Res.2016.PMID.26980599	0.90	0.59	1.37	0.63	0.91
MHC.Forero.24_Cancer.Immunol.Res.2016.PMID.26980599	0.90	0.62	1.30	0.56	0.88
MHC.I_BCR.2008.PMID.19272155	1.04	0.68	1.60	0.84	0.98
MHC.II_BCR.2008.PMID.19272155	0.67	0.41	1.10	0.11	0.79
MHCI.coreGenes_Nat.Commun.2017.PMID29170503	1.08	0.69	1.68	0.75	0.97
MIR200c.Induced_ONCO.2015.PMID.25746005	0.76	0.49	1.19	0.23	0.79

MIR200c.Repressed_ONCO.2015.PMID.25746005	0.99	0.63	1.56	0.96	>0.99
miRNA.138.signature_Cancer.Res.2014.PMID.25339353	0.98	0.64	1.51	0.94	>0.99
MITO1_BMC.Med.Genomics.2011.PMID.21214954	1.01	0.54	1.87	0.98	>0.99
MITO2_BMC.Med.Genomics.2011.PMID.21214954	1.07	0.71	1.61	0.76	0.98
Mitotic.Count_J.Pathol.2017.PMID.27861902	1.32	0.79	2.21	0.30	0.80
MK14.K17_BMC.Med.Genomics.2011.PMID.21214954	0.70	0.46	1.07	0.10	0.79
MKRAS.amplicon_BMC.Med.Genomics.2011.PMID.21214954	0.86	0.58	1.28	0.46	0.85
MM.BRCAwnt.1pFDR.UP_Genome.Biology.2007.PMID.17493263	0.86	0.48	1.54	0.62	0.91
MM.C3Tag.1pFDR.UP_Genome.Biology.2007.PMID.17493263	0.96	0.62	1.49	0.85	0.98
MM.C3Tag.2012_Genome.Biol.2013.PMID.24220145	1.03	0.68	1.55	0.90	>0.99
MM.Class3_Genome.Biol.2013.PMID.24220145	1.26	0.84	1.87	0.26	0.79
MM.Class8_Genome.Biol.2013.PMID.24220145	0.86	0.60	1.24	0.42	0.83
MM.Claudinlow_Genome.Biol.2013.PMID.24220145	0.75	0.48	1.19	0.22	0.79
MM.DMBAwnt.1pFDR.UP.Genome.Biology.2007.PMID.17493263	0.60	0.36	0.99	0.04	0.79
MM.Erbb2.like_Genome.Biol.2013.PMID.24220145	0.94	0.59	1.52	0.81	0.98
MM.Myc.2012_Genome.Biol.2013.PMID.24220145	1.03	0.64	1.66	0.90	>0.99
MM.Myoepithelioma.like_Genome.Biol.2013.PMID.24220145	0.67	0.43	1.03	0.07	0.79
MM.Neu.2012_Genome.Biol.2013.PMID.24220145	0.95	0.60	1.51	0.83	0.98
MM.NeuPyMT.1pFDR.UP_Genome.Biology.2007.PMID.17493263	1.17	0.70	1.94	0.55	0.88
MM.Normal.1pFDR.UP_Genome.Biology.2007.PMID.17493263	0.81	0.51	1.28	0.36	0.83
MM.Normal.like_Genome.Biol.2013.PMID.24220145	0.85	0.46	1.58	0.60	0.91
MM.p53null.1pFDR.UP_Genome.Biology.2007.PMID.17493263	0.87	0.55	1.38	0.55	0.88
MM.p53null.Basal_Genome.Biol.2013.PMID.24220145	0.78	0.51	1.19	0.24	0.79
MM.p53null.Luminal_Genome.Biol.2013.PMID.24220145	0.87	0.57	1.32	0.52	0.87
MM.Potluck_1pFDR_UP_Genome.Biology.2007_PMID.17493263.PMID.24220145	0.69	0.44	1.08	0.11	0.79
MM.PyMT.2012_Genome.Biol.2013.PMID.24220145	0.89	0.55	1.42	0.61	0.91
MM.Squamous.like_Genome.Biol.2013.PMID.24220145	0.76	0.51	1.12	0.16	0.79
MM.Stat1_Genome.Biol.2013.PMID.24220145	0.70	0.46	1.07	0.10	0.79
MM.WapINT3.1pFDR.UP_Genome.Biology.2007.PMID.17493263	0.78	0.48	1.27	0.31	0.80
MM.WapINT3.2012_Genome.Biol.2013.PMID.24220145	0.84	0.55	1.28	0.42	0.83
MM.WAPTag.1pFDR.UP_Genome.Biology.2007.PMID.17493263	1.20	0.76	1.90	0.42	0.83
MM.Wnt1.Early_Genome.Biol.2013.PMID.24220145	0.75	0.50	1.12	0.16	0.79
MM.Wnt1.Late_Genome.Biol.2013.PMID.24220145	0.75	0.44	1.27	0.28	0.80
Mmyosin_BMC.Med.Genomics.2011.PMID.21214954	0.87	0.49	1.55	0.64	0.91
MNADH_CYTochrome_BMC.Med.Genomics.2011.PMID.21214954	0.90	0.57	1.41	0.64	0.91
MNB1_BMC.Med.Genomics.2011.PMID.21214954	0.87	0.49	1.57	0.65	0.91
MNB2_BMC.Med.Genomics.2011.PMID.21214954	0.76	0.40	1.44	0.40	0.83
MNB3_BMC.Med.Genomics.2011.PMID.21214954	0.85	0.41	1.74	0.65	0.91
MNOtch4_BMC.Med.Genomics.2011.PMID.21214954	0.91	0.60	1.37	0.64	0.91
Monocyte_CellRep.2017.PMID.28052254	0.74	0.45	1.22	0.24	0.79
Monocyte..DC.25gene_Genome.Biol.2013.PMID.23618380	0.72	0.45	1.15	0.17	0.79
Monocytes_CancerImmunolRes.2018.PMID.30266715	0.65	0.38	1.11	0.11	0.79
Monocytes_Nat.Methods.2015.PMID.25822800	0.66	0.39	1.09	0.11	0.79
Monocytic.lineage.MCP_Nature.2020.PMID.31942075	0.76	0.44	1.31	0.32	0.80
MProliferation_BMC.Med.Genomics.2011.PMID.21214954	0.98	0.62	1.53	0.92	>0.99
MProtocadherin_BMC.Med.Genomics.2011.PMID.21214954	0.64	0.41	1.00	0.05	0.79
MPYMT_NEU_Cluster_BMC.Med.Genomics.2011.PMID.21214954	0.97	0.68	1.40	0.87	>0.99
MRibosomal_BMC.Med.Genomics.2011.PMID.21214954	0.99	0.53	1.82	0.97	>0.99
MS.CD44.DOWN_PNAS.2009.PMID.19666588	1.27	0.72	2.23	0.40	0.83

MS.CD44.UP_PNAS.2009.PMID.19666588	0.77	0.48	1.23	0.27	0.80
MSquamous_BMC.Med.Genomics.2011.PMID.21214954	0.82	0.52	1.29	0.39	0.83
Murat.G07_JCO.2008.PMID.18565887	0.88	0.57	1.35	0.54	0.88
Murat.G18_JCO.2008.PMID.18565887	0.99	0.67	1.48	0.97	>0.99
Murat.G24_JCO.2008.PMID.18565887	0.61	0.36	1.03	0.06	0.79
MVEGFC_BMC.Med.Genomics.2011.PMID.21214954	0.85	0.56	1.32	0.48	0.86
Myeloid.cell.chemotaxis.1gene_Nature.2020.PMID.31942077	1.20	0.70	2.05	0.51	0.87
Myeloid.dendritic.cells.MCP_Nature.2020.PMID.31942077	0.69	0.46	1.03	0.07	0.79
Natural.killer.cell_CellRep.2017.PMID.28052254	0.74	0.50	1.08	0.11	0.79
Natural.killer.T.cell_CellRep.2017.PMID.28052254	0.56	0.36	0.87	0.01	0.79
Necrosis_J.Pathol.2017.PMID.27861902	1.26	0.80	2.01	0.32	0.80
Neutrophil_CellRep.2017.PMID.28052254	0.96	0.54	1.70	0.88	>0.99
Neutrophils_CancerImmunolRes.2018.PMID.30266715	0.65	0.40	1.04	0.07	0.79
Neutrophils_Immunity.2013.PMID.24138885	0.74	0.44	1.23	0.25	0.79
Neutrophils_Nat.Methods.2015.PMID.25822800	0.59	0.35	0.99	0.05	0.79
Neutrophils.MCP_Nature.2020.PMID.31942077	1.28	0.73	2.24	0.38	0.83
NK_Immunity.2013.PMID.24138885	1.05	0.72	1.54	0.79	0.98
NK.activated_Nat.Methods.2015.PMID.25822800	0.79	0.51	1.23	0.30	0.80
NK.CD56bright_Immunity.2013.PMID.24138885	0.72	0.48	1.09	0.12	0.79
NK.CD56dim_Immunity.2013.PMID.24138885	0.82	0.51	1.30	0.39	0.83
NK.resting_Nat.Methods.2015.PMID.25822800	0.81	0.51	1.27	0.35	0.82
NKcells_CancerImmunolRes.2018.PMID.30266715	0.86	0.57	1.30	0.48	0.86
NKcells.MCP_Nature.2020.PMID.31942077	0.80	0.47	1.35	0.40	0.83
No.Response.Immunotherapy.TLS.Melanoma_Nature.2020.PMID.31942075	1.21	0.75	1.93	0.43	0.83
Normal.mucosa_Immunity.2013.PMID.24138885	0.99	0.62	1.57	0.97	>0.99
Nuclear.Pleomorphism_J.Pathol.2017.PMID.27861902	1.04	0.71	1.50	0.85	0.98
Oncotype_NEJM.2004.PMID.15591335	0.83	0.52	1.33	0.44	0.83
P53.ERPos.MDACC_CCR.2011.PMID.21248301	0.99	0.65	1.50	0.97	>0.99
Parity.signature.251genes_BCR.2014.PMID.25005139	0.68	0.42	1.10	0.12	0.79
Parity.signature.40genes_BCR.2014.PMID.25005139	0.74	0.46	1.19	0.21	0.79
PARPi.Resistance_BCRT_2012.PMID.22875744	1.09	0.74	1.62	0.67	0.92
PARPi.Sensitivity_BCRT_2012.PMID.22875744	1.08	0.66	1.77	0.75	0.97
PARPi.Sensitivity.MDACC_NPJ.Syst.Biol.Appl.2017.PMID.28649435	1.11	0.75	1.64	0.59	0.90
PARPi.Sensitivity.Negative_Sci.Adv.2017.PMID.28439535	0.82	0.57	1.17	0.27	0.80
PARPi.Sensitivity.Positive_Sci.Adv.2017.PMID.28439535	0.98	0.63	1.52	0.94	>0.99
Pcorr.Breast2Lung.LM2.Correlation_Nature.2005.PMID.16049480	0.87	0.56	1.33	0.51	0.87
Pcorr.Breast2Lung.Parental.Correlation_Nature.2005.PMID.16049480	1.15	0.75	1.76	0.52	0.88
Pcorr.dasatinib.resistant_Cancer.Res.2007.PMID.17332353	0.87	0.53	1.44	0.59	0.91
Pcorr.dasatinib.sensitive_Cancer.Res.2007.PMID.17332353	1.14	0.69	1.89	0.60	0.91
Pcorr.Hypoxia.High.Correlation_PLoS.Med.2006.PMID.16417408	1.16	0.76	1.78	0.48	0.86
Pcorr.Hypoxia.Low.Correlation_PLoS.Med.2006.PMID.16417408	0.89	0.58	1.37	0.61	0.91
Pcorr.IGS_Invasiveness_NJEM.2007.PMID.17229949	0.95	0.60	1.52	0.84	0.98
Pcorr.wound.response.activated_PNAS.2005.PMID.15701700	0.96	0.66	1.39	0.82	0.98
pCR.predictor.ERNeg.55genes_JAMA.2011.PMID.21558518	1.02	0.65	1.61	0.92	>0.99
pCR.predictor.ERPos.39genes_JAMA.2011.PMID.21558518	1.94	1.27	2.95	0.00	0.79
PDCD1_Single_Gene.Single	0.92	0.60	1.40	0.69	0.93
Pfefferle2012.LumProg_BCR.2015.PMID.25575446	0.72	0.43	1.20	0.20	0.79
Pfefferle2012.MaSC_BCR.2015.PMID.25575446	0.58	0.35	0.96	0.03	0.79
Pfefferle2012.MatureLum_BCR.2015.PMID.25575446	1.06	0.69	1.63	0.78	0.98

Pfefferle2012.Stroma_BCR.2015.PMID.25575446	0.79	0.49	1.26	0.31	0.80
PGR_Single_Gene.Single	0.89	0.53	1.48	0.65	0.91
PI3Ki.Down_CancerCell.2017.PMID.28528867	0.84	0.52	1.36	0.49	0.86
PI3Ki.Up_CancerCell.2017.PMID.28528867	0.94	0.58	1.51	0.79	0.98
PIK3CA.Pathway_Ann.Oncol.2017.PMID.28177460	0.73	0.45	1.20	0.21	0.79
PIK3CAmt.signature_Cancer.Res.2012.PMID.22552288	1.03	0.70	1.51	0.89	>0.99
Plasma.cells_Nat.Methods.2015.PMID.25822800	0.76	0.49	1.18	0.23	0.79
PlasmaCells_CancerImmunolRes.2018.PMID.30266715	0.74	0.49	1.11	0.15	0.79
Plasmacytoid.dendritic.cell_CellRep.2017.PMID.28052254	0.68	0.44	1.08	0.10	0.79
PR.Isoform.Ratio.Up.in.PRA.H_JNCI.2017.PMID.28376177	0.77	0.46	1.28	0.31	0.80
PR.Isoform.Ratio.Up.in.PRB.H_JNCI.2017.PMID.28376177	0.86	0.55	1.35	0.52	0.87
Proliferation.Cluster_BMC.Med.Genomics.2011.PMID.21214954	1.08	0.68	1.72	0.74	0.97
Proliferation.Metagene_Genome.Biol.2013.PMID.23618380	1.03	0.66	1.61	0.91	>0.99
Proliferation.score.PAM50_JCO.2009.PMID.19204204	0.95	0.61	1.50	0.84	0.98
ProliferationPathway_CancerImmunolRes.2018.PMID.30266715	1.04	0.67	1.62	0.86	0.99
Prosigna.Proliferation.18_BMC.Med.Genomics.2015.PMID.26297356	1.11	0.73	1.69	0.64	0.91
Race.LuminalA.MRE.score_BCRT.2015.PMID.26109344	1.05	0.69	1.61	0.81	0.98
Radiation.induced.genes_Radoat.Res.2014.PMID.24527691	0.83	0.48	1.43	0.50	0.86
RB.LOH_BCR.2008.PMID.18782450	1.01	0.67	1.54	0.95	>0.99
RB.LOSS_JCI.2007.PMID.17160137	1.00	0.65	1.55	>0.99	>0.99
Regulatory.T.cell_CellRep.2017.PMID.28052254	0.72	0.43	1.20	0.21	0.79
Replication.Stress.Down.set_Cell.Rep.2018.PMID.29768207	0.90	0.56	1.43	0.65	0.91
Replication.Stress.Model_Cell.Rep.2018_PMID.29768207.PMID.29768207	0.85	0.56	1.28	0.44	0.83
Replication.Stress.Neg_Cell.Rep.2018_PMID.29768207.PMID.29768207	0.86	0.54	1.38	0.54	0.88
Replication.Stress.Pos_Cell.Rep.2018_PMID.29768207.PMID.29768207	0.74	0.49	1.12	0.16	0.79
Replication.Stress.Up_Set_Cell.Rep.2018_PMID.29768207.PMID.29768207	0.76	0.51	1.13	0.18	0.79
Residual.disease.predictor.ERNeg.54genes_JAMA.2011.PMID.21558518	1.02	0.71	1.45	0.93	>0.99
Residual.disease.predictor.ERPos.73genes_JAMA.2011.PMID.21558518	0.81	0.50	1.31	0.39	0.83
Response.Immunotherapy.MCP.TLS.Melanoma_Nature.2020.PMID.31942075	0.79	0.51	1.22	0.28	0.80
Response.Immunotherapy.signature_Science.2018.PMID.30309915	0.78	0.49	1.24	0.29	0.80
Response.Neo.Chemo_common_CCR.2014.PMID.25047707	1.03	0.67	1.59	0.90	>0.99
Response.Neo.Chemo_ERNeg_CCR.2014.PMID.25047707	1.23	0.88	1.71	0.23	0.79
Response.Neo.Chemo_ERPos_CCR.2014.PMID.25047707	1.30	0.84	2.01	0.23	0.79
RHOA.pathway_Ann.Oncol.2017.PMID.28177460	1.67	0.98	2.86	0.06	0.79
Ribosomal.Cluster_BMC.Med.Genomics.2011.PMID.21214954	0.75	0.41	1.39	0.37	0.83
ROR.subtype.PAM50_JCO.2009.PMID.19204204	0.90	0.51	1.57	0.70	0.95
ROR.subtype.proliferation.PAM50_JCO.2009.PMID.19204204	0.93	0.57	1.52	0.78	0.98
RSS.Score_CCR.2018.PMID.29921729	0.90	0.63	1.30	0.59	0.90
S100A9.A8_BMC.Med.Genomics.2011.PMID.21214954	1.08	0.64	1.83	0.78	0.98
Scorr.EMAT1.Correlation_BCR.2020.PMID.32641077	0.91	0.58	1.41	0.66	0.92
Scorr.EMAT2.Correlation_BCR.2020.PMID.32641077	1.00	0.67	1.49	0.98	>0.99
Scorr.EMAT3.Correlation_BCR.2020.PMID.32641077	1.23	0.78	1.94	0.38	0.83
Scorr.EMAT4.Correlation_BCR.2020.PMID.32641077	0.79	0.50	1.26	0.32	0.80
Scorr.IE.Correlation_JCO.2006.PMID.16505416	0.95	0.59	1.51	0.82	0.98
Scorr.IIE.Correlation_JCO.2006.PMID.16505416	0.99	0.62	1.58	0.96	>0.99
Scorr.PAM50.Basal_JCO.2009.PMID.19204204	0.93	0.58	1.51	0.78	0.98
Scorr.PAM50.Her2_JCO.2009.PMID.19204204	0.84	0.51	1.38	0.49	0.86
Scorr.PAM50.LumA_JCO.2009.PMID.19204204	1.12	0.64	1.97	0.68	0.93
Scorr.PAM50.LumB_JCO.2009.PMID.19204204	1.09	0.67	1.79	0.72	0.96

Scorr.PAM50.Normal_JCO.2009.PMID.19204204	0.94	0.56	1.57	0.81	0.98
Scorr.S329.L_Br.J.Cancer.2008.PMID.18382427	0.83	0.51	1.35	0.45	0.83
Scorr.S329.R_Br.J.Cancer.2008.PMID.18382427	1.14	0.65	2.00	0.64	0.91
Secretoglobin_BMC.Med.Genomics.2011.PMID.21214954	0.75	0.50	1.13	0.17	0.79
Shehata2012.ALDHneg_BCR.2015.PMID.25575446	1.04	0.69	1.56	0.86	0.99
Shehata2012.ALDHpos_BCR.2015.PMID.25575446	0.70	0.42	1.18	0.18	0.79
Shehata2012.Basal_BCR.2015.PMID.25575446	0.64	0.38	1.08	0.10	0.79
Shehata2012.Erbb3neg_BCR.2015.PMID.25575446	0.81	0.57	1.17	0.26	0.79
Shehata2012.LumProg_BCR.2015.PMID.25575446	0.93	0.58	1.49	0.76	0.98
Shehata2012.NCL_BCR.2015.PMID.25575446	0.94	0.57	1.54	0.80	0.98
Shehata2012.Stroma_BCR.2015.PMID.25575446	0.77	0.49	1.23	0.27	0.80
Spike2012.aMaSC_BCR.2015.PMID.25575446	0.92	0.57	1.49	0.74	0.97
Spike2012.fMaSC_BCR.2015.PMID.25575446	0.73	0.46	1.17	0.19	0.79
Spike2012.fStr_BCR.2015.PMID.25575446	0.82	0.47	1.44	0.49	0.86
STAT1_BCR.2008.PMID.19272155	0.98	0.62	1.54	0.93	>0.99
STAT3.Basal_PNAS.2014.PMID.25139989	0.70	0.46	1.07	0.10	0.79
STAT3.Basal.short_PNAS.2014.PMID.25139989	0.76	0.50	1.14	0.18	0.79
Stroma.FNA.MDACC.1_JCO.2010.PMID.20805453	0.82	0.52	1.31	0.41	0.83
Stroma.FNA.MDACC.2_JCO.2010.PMID.20805453	0.87	0.56	1.35	0.52	0.88
Stromal.Central.Fibrotic.Focus_J.Pathol.2017.PMID.27861902	0.84	0.57	1.24	0.38	0.83
Stromal.Down_Nat.Med.2009.PMID.19648928	0.79	0.50	1.24	0.30	0.80
Stromal.Inflammation_J.Pathol.2017.PMID.27861902	0.76	0.49	1.18	0.22	0.79
Stromal.Signature_Nat.Med.2008.PMID.18438415	0.87	0.58	1.32	0.53	0.88
Stromal.Up_Nat.Med.2009.PMID.19648928	0.82	0.50	1.34	0.42	0.83
SW480.cancer.cells_Immunity.2013.PMID.24138885	0.89	0.58	1.35	0.57	0.89
T.follicular.helper.cell_CellRep.2017.PMID.28052254	0.70	0.45	1.08	0.10	0.79
Tcell.activation_Nature.2020.PMID.31942077	1.09	0.71	1.67	0.69	0.94
Tcell.CD8.Effector.vs.naive.2_Science.2016.PMID27789795	0.99	0.63	1.54	0.95	>0.99
Tcell.CD8.Exhausted.vs.antiPDL1.2_Science.2016.PMID27789795	0.92	0.60	1.41	0.71	0.95
Tcell.CD8.Exhausted.vs.naive.2_Science.2016.PMID27789795	0.97	0.63	1.51	0.90	>0.99
Tcell.CD8.Memory.vs.naive.1_Science.2016.PMID27789795	0.75	0.48	1.18	0.21	0.79
Tcell.cluster_CCR.2014.PMID.24916698	0.82	0.54	1.23	0.33	0.80
Tcell.EXH.Anti.PDL1.vs.control.treated.exhausted.CD8.Tcell.Metagene.1.Science.2016.PMID.27789795	1.16	0.76	1.75	0.49	0.86
Tcell.EXH.Effector.CD8.T.cell.at.day.8.p.i.Armstrong.vs.Naive.CD8.Tcell.Metagene.1_Science.2016.PMID.27789795	0.82	0.52	1.28	0.37	0.83
Tcell.EXH.Exhausted.CD8.T.cell.vs.Naive.CD8.T.cell.Metagene.1_Science.2016.PMID.27789795	1.12	0.71	1.76	0.63	0.91
Tcell.EXH.Exhausted.CD8.T.cell.vs.Naive.CD8.T.cell.Metagene.3_Science.2016.PMID.27789795	0.73	0.46	1.15	0.17	0.79
Tcell.EXH.Memory.CD8.T.cell.a.vs.Naive.CD8.T.cell.Metagene.1_Science.2016.PMID.27789795	0.75	0.48	1.18	0.21	0.79
Tcell.EXH.Memory.CD8.T.cell.a.vs.Naive.CD8.T.cell.Metagene.2_Science.2016.PMID.27789795	0.88	0.58	1.34	0.57	0.88
Tcell.EXH.Memory.CD8.T.cell.a.vs.Naive.CD8.T.cell.Metagene.3.Science_2016.PMID.27789795	0.91	0.60	1.37	0.64	0.91
Tcell.NK.51gene_Genome.Biol.2013.PMID.23618380	0.80	0.54	1.17	0.25	0.79
Tcell.NK.Metagene_Genome.Biol.2013.PMID.23618380	0.83	0.57	1.20	0.32	0.80
Tcell.RM_Nat_Med.2018.PMID.29942092	0.88	0.58	1.33	0.55	0.88
Tcell.survival.2gene_Nature.2020.PMID.31942077	0.82	0.51	1.33	0.43	0.83
Tcells_CancerImmunolRes.2018.PMID.30266715	0.77	0.51	1.15	0.20	0.79
Tcells_Immunity.2013.PMID.24138885	0.79	0.53	1.16	0.23	0.79
Tcells_TFH_Nat.Methods.2015.PMID.25822800	0.86	0.57	1.30	0.47	0.86
Tcells.CD4.memory.activated_Nat.Methods.2015.PMID.25822800	0.80	0.54	1.19	0.27	0.80
Tcells.CD4.memory.resting_Nat.Methods.2015.PMID.25822800	0.82	0.54	1.25	0.35	0.82
Tcells.CD4.naive_Nat.Methods.2015.PMID.25822800	0.78	0.51	1.19	0.25	0.79

Tcells.CD8_Immunity.2013.PMID.24138885	0.91	0.60	1.39	0.67	0.93
Tcells.CD8_Nat.Methods.2015.PMID.25822800	0.84	0.55	1.26	0.40	0.83
Tcells.CD8.MCP_Nature.2020.PMID.31942075	0.95	0.61	1.47	0.81	0.98
Tcells.Cytotoxic.MCP_Nature.2020.PMID.31942075	0.89	0.58	1.38	0.61	0.91
Tcells.gammadelta_Nat.Methods.2015.PMID.25822800	0.84	0.55	1.29	0.42	0.83
Tcells.helper_Immunity.2013.PMID.24138885	0.95	0.63	1.44	0.82	0.98
Tcells.MCP_Nature.2020.PMID.31942077	0.84	0.57	1.23	0.37	0.83
Tcells.regulatory.2gene_Nature.2020.PMID.31942077	0.72	0.52	1.01	0.06	0.79
Tcells.Tregs_Nat.Methods.2015.PMID.25822800	0.77	0.51	1.16	0.21	0.79
TCGA.BRCA.1198_BASAL_JCI.2020.PMID.32573490	0.79	0.49	1.27	0.33	0.80
TCGA.BRCA.1198_Chromogranin_JCI.2020.PMID.32573490	0.88	0.57	1.35	0.56	0.88
TCGA.BRCA.1198_COLLAGEN11A_JCI.2020.PMID.32573490	0.93	0.61	1.41	0.73	0.96
TCGA.BRCA.1198_EN1_FDZ9_JCI.2020.PMID.32573490	1.16	0.75	1.80	0.50	0.86
TCGA.BRCA.1198_FGFR4_EGF_JCI.2020.PMID.32573490	0.84	0.53	1.35	0.47	0.86
TCGA.BRCA.1198_HISTONES_JCI.2020.PMID.32573490	1.04	0.68	1.60	0.85	0.98
TCGA.BRCA.1198_HOXC11_HOTAIR_SIX1_JCI.2020.PMID.32573490	1.00	0.64	1.57	>0.99	>0.99
TCGA.BRCA.1198_IL8_CCL_JCI.2020.PMID.32573490	0.85	0.54	1.36	0.50	0.86
TCGA.BRCA.1198_immune_CD19_JCI.2020.PMID.32573490	0.77	0.53	1.14	0.19	0.79
TCGA.BRCA.1198_immune_CD34_TIE1_JCI.2020.PMID.32573490	0.84	0.51	1.37	0.49	0.86
TCGA.BRCA.1198_immune_CD4_CD53_CD84_BTK_JCI.2020.PMID.32573490	0.72	0.45	1.14	0.16	0.79
TCGA.BRCA.1198_immune_CD8_GZMK_JCI.2020.PMID.32573490	0.80	0.53	1.21	0.28	0.80
TCGA.BRCA.1198_immune_CTLA4_CXCL_FOXP3_JCI.2020.PMID.32573490	0.94	0.61	1.46	0.79	0.98
TCGA.BRCA.1198_immune_FOS_JUN_IL6_JCI.2020.PMID.32573490	1.32	0.95	1.83	0.10	0.79
TCGA.BRCA.1198_immune_GIMAP_IL16_JCI.2020.PMID.32573490	0.73	0.47	1.12	0.15	0.79
TCGA.BRCA.1198_immune_HLA_A_F_JCI.2020.PMID.32573490	1.06	0.68	1.65	0.80	0.98
TCGA.BRCA.1198_immune_HLA_D_JCI.2020.PMID.32573490	0.59	0.36	0.96	0.03	0.79
TCGA.BRCA.1198_immune_INTERFERON_JCI.2020.PMID.32573490	1.45	0.97	2.18	0.07	0.79
TCGA.BRCA.1198_IMMUNE1_JCI.2020.PMID.32573490	0.85	0.56	1.27	0.43	0.83
TCGA.BRCA.1198_LUMINAL_JCI.2020.PMID.32573490	0.92	0.49	1.72	0.79	0.98
TCGA.BRCA.1198_MYBL2_APOBEC3B_JCI.2020.PMID.32573490	0.95	0.60	1.50	0.82	0.98
TCGA.BRCA.1198_NORMAL_JCI.2020.PMID.32573490	0.65	0.34	1.25	0.20	0.79
TCGA.BRCA.1198_NORMAL2_JCI.2020.PMID.32573490	0.86	0.51	1.43	0.55	0.88
TCGA.BRCA.1198_PDCHA_MANY_JCI.2020.PMID.32573490	1.04	0.68	1.57	0.87	0.99
TCGA.BRCA.1198_S100A7_8_9_JCI.2020.PMID.32573490	0.93	0.61	1.43	0.75	0.97
TCGA.BRCA.1198_TP63_JCI.2020.PMID.32573490	0.90	0.52	1.57	0.72	0.96
TCGA.BRCA.1198.IMMUNOGLOBULIN_JCI.2020.PMID.32573490	0.86	0.58	1.28	0.45	0.83
TCGA.CSF1.response_Immunity.2018.PMID.29628290	0.73	0.47	1.15	0.17	0.79
TCGA.IFN.score_Immunity.2018.PMID.29628290	1.49	1.00	2.20	0.05	0.79
TCGA.Liexpression.score_Immunity.2018.PMID.29628290	0.83	0.57	1.21	0.33	0.80
TCGA.Serum.response.up_Immunity.2018.PMID.29628290	0.82	0.52	1.30	0.41	0.83
TCGA.TFH_Immunity.2018.PMID.29628290	1.01	0.68	1.52	0.95	>0.99
TCGA.Tgd_Immunity.2018.PMID.29628290	0.97	0.60	1.58	0.91	>0.99
TCGA.TGFB.score_Immunity.2018.PMID.29628290	1.02	0.68	1.54	0.92	>0.99
Tcm_Immunity.2013.PMID.24138885	0.91	0.66	1.26	0.57	0.88
Tem_Immunity.2013.PMID.24138885	0.67	0.44	1.01	0.06	0.79
TFH_Immunity.2013.PMID.24138885	1.01	0.68	1.52	0.95	>0.99
Tgd_Immunity.2013.PMID.24138885	0.97	0.60	1.58	0.91	>0.99
Th1_cells_Immunity.2013.PMID.24138885	0.70	0.44	1.11	0.13	0.79
Th17_cells_Immunity.2013.PMID.24138885	1.17	0.80	1.69	0.42	0.83

Th2_cells_Immunity.2013.PMID.24138885	1.17	0.72	1.89	0.53	0.88
TLS.9Gene.Signature_Nature.2020.PMID.31942071	0.81	0.53	1.23	0.32	0.80
TLS.CXCL13.SingleGene_Nature.2020.PMID.31942077	0.81	0.56	1.19	0.29	0.80
TLS.Hallmark.Gene.Signature_Nature.2020.PMID.31942071	0.73	0.49	1.08	0.12	0.79
TLS.Known.Markers_Nature.2020.PMID.31942071	0.74	0.50	1.10	0.13	0.79
TLS.Structure.12chemokine_FrontImmunol.2017.PMID.28713385	0.80	0.52	1.22	0.30	0.80
TLS.tumors.wTLS.and.CD8.vs.CD8alone_Nature.2020.PMID.31942071	0.78	0.51	1.20	0.26	0.79
TNBC.good.prognosis.TNBC.230genes_BCR.2011.PMID.21978456	0.93	0.62	1.41	0.75	0.97
TNBC.good.prognosis.TNBC.26genes_BCR.2011.PMID.21978456	0.78	0.53	1.14	0.20	0.79
TNBC.metastasis.free.survival_PLoS.One.2013.PMID.24349199	0.78	0.51	1.18	0.23	0.79
TNBC.poor.prognosis.TNBC.26genes_BCR.2011.PMID.21978456	0.94	0.60	1.46	0.77	0.98
Translation.Pathway_CancerImmunolRes.2018.PMID.30266715	0.67	0.36	1.25	0.21	0.79
Tumour.hypoxia.causes.DNA.hypermethylation_Nature.2016.PMID.27533040	1.38	0.89	2.15	0.15	0.79
Type.1.T.helper.cell_CellRep.2017.PMID.28052254	0.89	0.59	1.36	0.60	0.91
Type.17.T.helper.cell_CellRep.2017.PMID.28052254	0.73	0.46	1.16	0.18	0.79
Type.2.T.helper.cell_CellRep.2017.PMID.28052254	0.91	0.57	1.45	0.68	0.93
Up.Basal.High_Nat.Cell.Biol.2014.PMID.25173976	0.70	0.46	1.06	0.09	0.79
Up.Proliferation_Nat.Cell.Biol.2014.PMID.25173976	0.94	0.59	1.50	0.81	0.98
Upregulated.by.oncogenic.NRAS.basal_Cell.Rep.2016.PMID.26166574	0.61	0.40	0.93	0.02	0.79
Upregulated.upon.NRAS.repression.basal_Cell.Rep.2017.PMID.26166574	0.72	0.45	1.17	0.18	0.79
Vascular.Content_Clin.Exp.Metastasis.2014.PMID.23975155	0.96	0.63	1.46	0.85	0.98
VEGF.13genes_BMC.Med.2009.PMID.19291283	1.08	0.68	1.72	0.74	0.97
Wirapati.Proliferation_BCR.2008.PMID.18662380	1.01	0.65	1.57	0.97	>0.99
Wound.Signature_CCR.2009.PMID.19887484	0.80	0.52	1.24	0.32	0.80
X11q13.Amplicon_BMC.Med.Genomics.2011.PMID.21214954	0.84	0.52	1.36	0.48	0.86
X12qMDM4.BMC.Med.Genomics.2011.PMID.21214954	0.84	0.54	1.32	0.45	0.83
X13q14.Amplicon_BMC.Med.Genomics.2011.PMID.21214954	1.01	0.71	1.44	0.96	>0.99
X15q25.Amplicon_BMC.Med.Genomics.2011.PMID.21214954	0.98	0.63	1.52	0.94	>0.99
X16.13.Amplicon_BMC.Med.Genomics.2011.PMID.21214954	0.84	0.56	1.27	0.41	0.83
X16q23.Amplicon_BMC.Med.Genomics.2011.PMID.21214954	0.66	0.43	1.01	0.05	0.79
X17PP13.Amplicon_BMC.Med.Genomics.2011.PMID.21214954	0.85	0.51	1.42	0.54	0.88
X17q25x.BMC.Med.Genomics.2011.PMID.21214954	1.17	0.83	1.66	0.36	0.83
X19p13.Amplicon_BMC.Med.Genomics.2011.PMID.21214954	1.09	0.74	1.61	0.66	0.92
X1p36.Amplicon_BMC.Med.Genomics.2011.PMID.21214954	0.62	0.39	0.98	0.04	0.79
X3p21.Amplicon_BMC.Med.Genomics.2011.PMID.21214954	1.04	0.59	1.82	0.90	>0.99
X4p16.Amplicon_BMC.Med.Genomics.2011.PMID.21214954	0.87	0.56	1.35	0.53	0.88
X5Q_BCRT.2012.PMID.22048815	1.15	0.75	1.77	0.51	0.87
X8p.Amplicon_BMC.Med.Genomics.2011.PMID.21214954	0.87	0.57	1.33	0.51	0.87
X8p22.Amplicon_BMC.Med.Genomics.2011.PMID.21214954	1.20	0.75	1.92	0.45	0.83
XBP1.Signature_Nature.2014.PMID.24670641	1.10	0.68	1.77	0.70	0.94
