# Supplementary material for: Tumor Intrinsic Subtypes and Gene Expression Signatures in Early-Stage ERBB2/HER2-Positive Breast Cancer: A Pooled Analysis of CALGB 40601, NeoALTTO, and NSABP B-41 Trials
Source: JAMA Oncol. 2024 Mar 28;10(5):603–11. doi: 10.1001/jamaoncol.2023.7304 (PMC10979363; doi:10.1001/jamaoncol.2023.7304)
Supplement: Supplement 2. — Data Sharing Statement [file jamaoncol-e237304-s002.pdf]

Data Sharing Statement

Fernandez-Martinez. Clinical Relevance of Tumor Intrinsic Subtypes and Gene Expression Signatures in Early-Stage ERBB2/HER2-Positive Breast Cancer. *JAMA Oncol*. Published March 28, 2024. doi:10.1001/jamaoncol.2023.7304

Data
Data available: Yes
Data types: Deidentified participant data
How to access data: CALGB 40601 RNAseq FASTQ files are available via the NCBI dbGAP repository under accession number phs001570.v3.p1. The star-salmon upper quartile normalized gene expression matrix is available in GEO under the accession number GSE116335. NSABP B-41 gene expression data is available via the NCBI dbGAP repository under accession number phs003275.v1.p1. The star-salmon upper quartile normalized gene expression matrix is available in GEO under the accession number GSE234519. The NeoALTTO RNA sequencing data at baseline and the clinical data are available upon request after submission of a research project proposal (RPP) to the RPP's administrator (alttoresearchproposals@frontier-science.co.uk). In detail, access to data for research will be granted upon review of the RPP and its endorsement by the study Steering Committee, and after entering into an appropriate data access agreement between BIG, IJB, and the investigator, subject to applicable laws. More details and documents required can be found at https://bigagainstbreastcancer.org/clinical-trials/neoaltto/ under the section "Translational Research". The policy for access to residual biological samples and data in the NeoALTTO study is a fair scientific review process set up to ensure precious biological samples or data collected in the studies are accessed appropriately, to avoid duplication of efforts and foster collaboration. The data from the study are not anonymized yet, only pseudonymized, therefore they are still considered identifiable, and cannot be made publicly available at this point. In order to ensure that they are shared in a way that preserves the privacy of patients and complies with the relevant laws and regulations including the European General Data Protection Regulation (GDPR), researchers can only access the data after they sign the data transfer agreements mentioned above, either for reproducibility or for original research purposes.
When available: With publication

Supporting Documents
Document types: None

Additional Information
Who can access the data: The data will be available for researchers whose proposed use of the data has been approved
Types of analyses: The data will be made available for research purposes only
Mechanisms of data availability: For CALGB 40601 and NSABP B-41 (dbGAP), RNA sequencing data will be made available after approval of a proposal following the dbGAP data use certification agreements. The NeoALTTO RNA sequencing data at baseline and the clinical data are available upon request after submission of a research project proposal (RPP) to the RPP's administrator (alttoresearchproposals@frontier-science.co.uk).
